# PLA2R1 promotes DNA damage and inhibits spontaneous tumor formation during aging

**DOI:** 10.1038/s41419-021-03468-3

**Published:** 2021-02-16

**Authors:** Anda Huna, Audrey Griveau, David Vindrieux, Sara Jaber, Jean-Michel Flaman, Delphine Goehrig, Lamia Azzi, Jean-Jacques Médard, Sophia Djebali, Hector Hernandez-Vargas, Robert Dante, Léa Payen, Jacqueline Marvel, Philippe Bertolino, Sébastien Aubert, Pierre Dubus, David Bernard

**Affiliations:** 1 Centre de Recherche en Cancérologie de Lyon, Inserm U1052, CNRS UMR 5286, Centre Léon Bérard, Université de Lyon, Lyon, France; 2 INSERM U1053 Bordeaux Research in Translational Oncology, University of Bordeaux, Bordeaux Cedex, France; 3 Centre International de Recherche en Infectiologie, Inserm U1111, CNRS, UMR5308, École Normale Supérieure de Lyon, Université de Lyon, Université Claude Bernard Lyon 1, Lyon, France; 4 Institut de Pathologie, Centre de Biologie Pathologie, CHRU de Lille, Faculté de Médecine, Université de Lille, Lille Cedex, France; 5 Plateau cellules tissus, CHU de Bordeaux, Pessac, France

**Keywords:** Cancer, Mechanisms of disease, DNA damage and repair, Ageing

## Abstract

Although aging is a major risk factor for most types of cancers, it is barely studied in this context. The transmembrane protein PLA2R1 (phospholipase A2 receptor) promotes cellular senescence, which can inhibit oncogene-induced tumor initiation. Functions and mechanisms of action of PLA2R1 during aging are largely unknown. In this study, we observed that old Pla2r1 knockout mice were more prone to spontaneously develop a wide spectrum of tumors compared to control littermates. Consistently, these knockout mice displayed increased Parp1, a master regulator of DNA damage repair, and decreased DNA damage, correlating with large human dataset analysis. Forced PLA2R1 expression in normal human cells decreased PARP1 expression, induced DNA damage and subsequent senescence, while the constitutive expression of PARP1 rescued cells from these PLA2R1-induced effects. Mechanistically, PARP1 expression is repressed by a ROS (reactive oxygen species)-Rb-dependent mechanism upon PLA2R1 expression. In conclusion, our results suggest that PLA2R1 suppresses aging-induced tumors by repressing PARP1, via a ROS–Rb signaling axis, and inducing DNA damage and its tumor suppressive responses.

## Introduction

Aging is a major risk factor for most human cancers^1^. It can be modeled in mice that spontaneously develop tumors during aging without prior exposure to other risk factors such as radiation, cigarette smoke, or alcohol^2^. Nevertheless, the ways in which aging impacts cancer formation has seldom been studied.

Reactive oxygen species (ROS) and subsequent DNA damage accumulates during aging^3^. They have been shown to exert both pro- or anti-tumoral effects, but still their role during aging and the increased risk of spontaneous tumor formation are largely unknown^4–9^. DNA damage is expected to mainly exert pro-tumoral effects by promoting genomic instability and aneuploidy induction^7,10–13^, still it can also exert anti-tumoral effects by fueling aneuploidy^13^ and by induction of DNA damage signaling resulting in p53 activation, senescence, and apoptosis^4–6^.

PLA2R1 belongs to the mannose receptor family that can bind several matrix components, some sugar or several sPLA2 (secretory PLA2). Though little is known about the functional role of the transmembrane protein PLA2R1 in the cancer and aging contexts^14,15^, it was shown to increase ROS production and DNA damage accumulation leading to different cellular responses like cell death and cellular senescence^16–18^, and to different organismal outcome like limiting tumor formation during RAS-induced skin carcinogenesis^19^ and tumor growth^20,21^ as well as promoting some marks of premature aging in a murine model of progeria^22^.

In this study, we aimed at deciphering and investigating the role of the PLA2R1 protein during aging-induced tumor formation using aged mice, normal human cells and large human datasets.

## Materials and methods

### Cell culture and treatments

MRC5 normal human fibroblasts (ATCC) and virus‐producing GP293 cells (Clontech) were cultured in Dulbecco′s modified Eagle′s medium containing GlutaMAX and supplemented with 10% fetal bovine serum (FBS) (Sigma‐Aldrich) and 1% penicillin/streptomycin. Cells were maintained at 37 °C under a 5% CO_2_ atmosphere and used within 10 passages after purchasing. Cells were tested for mycoplasma and if needed treated with Plasmocin (Invivogen) until they became mycoplasma‐free before performing experiments. *N*-acetyl cysteine (NAC) and Trolox were purchased from Sigma‐Aldrich and used at 500 nM and 5 µM, respectively. H_2_O_2_ was added at 250 µM for 1 h.

### siRNA transfection

For the siRNA knockdown experiments, an ON‐TARGETplus nontargeting control pool-siCtrl (sequences UGGUUUACAUGUCGACUAA, UGGUUUACAUGUUGUGUGA, UGGUUUACAUGUUUUCUGA, UGGUUUACAUGUUUUCCUA) or ON‐TARGETplus siRNA SMARTpools targeting the Rb1 (GAACAGGAGUGCACGGAUA, GGUUCAACUACGCGUGUAA, CAUUAAUGGUUCACCUUCGA, CAACCCAGCAGUUCGAUAU) (Dharmacon) were used at 15 nM. Reverse transfection was performed in six‐well plates (cell density 5 × 10^5^ per well), using a 0.1% solution of Dharmafect 1 transfection reagent (Thermo Scientific).

### Vectors, transfection, and infection

The retroviral vectors encoding human PLA2R1, pLPCX/PLA2R1 for puromycin selection or pLNCX/PLA2R1 for neomycin selection, have previously been described;^16,19,21^ pBABE/PARP1 (puromycin selection) was described in ref. ^23^; ROS reporter pLPCx‐roGFP2‐ORP1 was described in ref. ^24^.

Virus‐producing GP293 cells were transfected with vectors using the GeneJuice reagent according to the manufacturer’s recommendations (Merck Millipore). Cells were transfected with the VSVg (1 µg) and the retroviral vector of interest (5 µg). Two days after transfection, the viral supernatant was mixed with fresh medium (1/2) and hexadimethrine bromide (8 μg/ml; Sigma‐Aldrich), and was then used to infect target cells for 6 h. One day after infection, selection was started using puromycin at 500 ng/ml or/and neomycin at 100 μg/ml.

### SA‐β‐galactosidase assay, crystal violet staining and growth curves

For SA‐β‐galactosidase assay, cells were washed twice with PBS and fixed for 5 min in 2% formaldehyde/0.2% glutaraldehyde. Cells were then rinsed twice in PBS and incubated at 37 °C overnight in a SA‐β‐ Gal solution as previously described^25^. For crystal violet staining, cells were washed with PBS, fixed for 15 min in 3.7% formaldehyde, and then stained with 0.05% crystal violet solution. For growth curves, cells were seeded at the same density and counted at each passage. The population doubling was calculated at each passage using the following formula: PD = ln(number of collected cells/number of plated cells)/ln2.

### ROS detection

Cells stably expressing roGFP2‐ORP1 were washed and incubated in HBSS with calcium, magnesium, and no phenol red (14025050, Thermo Scientific) for ROS detection. Fluorescence (Ex 405 and 488 nm/Em 500–554 nm) was monitored in live cells using the Operetta system. The Columbus software was used to quantify fluorescence intensity, and the ratio between 405 nm (oxidized state) and 488 nm (reduced state) was calculated, as previously described^26^.

### Reverse transcription and real‐time quantitative PCR

Total RNA was isolated by phenol–chloroform extraction on cells. The Maxima First cDNA Synthesis Kit (Life Technologies) was used to synthesize cDNA according to the manufacturer’s instructions from 1 µg of RNA. The reverse transcription (RT) reaction mixture was used at a dilution of 1/20 as a cDNA template for quantitative PCR (qPCR) analysis. TaqMan qPCR analyses were carried out on a FX96 Thermocycler (Bio‐ Rad). The PCR mixture contained TaqMan mix (Roche), 200 nM of primers, the Universal Probe Library probe (100 µM) for the gene of interest (TaqMan Gene Expression Assays [Primers/probe]; Life technologies), and 1.67 μl cDNA template. Reactions were performed at least in technical duplicates. The relative amount of mRNA was calculated using the comparative Ct (ΔΔCT) method, following data normalization against GAPDH as a housekeeping gene. The PCR primers and UPL probes used for the qPCR are listed in Supplementary Fig. 1.

### Western blot

Cells were directly lysed in Laemmli buffer. Cell lysates were resolved by SDS‐PAGE electrophoresis and transferred to nitrocellulose membranes (Bio‐Rad). Membranes were then blocked with TBST‐Milk 5% for 1 h and incubated with primary antibodies overnight at 4 °C. Primary antibodies used are listed in Supplementary Fig. 2. Horseradish peroxidase‐conjugated donkey anti‐rabbit and sheep anti‐mouse antibodies (Interchim) were used as secondary antibodies and incubated for 1 h at room temperature. Detection was performed using the ECL kit (Amersham) and the Biorad Chemidoc system.

### Comet assay

For each condition, cells were suspended 1:10 in 0.5% low-melting point agarose at 37 °C. The suspension was immediately laid onto a Comet slide (Trevigen Inc.), 2.000 cells per well. Agarose was allowed to solidify at 4 °C for 15 min. The Comet slides were then immersed in prechilled lysis solution (1.2 M NaCl, 100 mM EDTA, 10 mM Tris, 1% Triton (pH 10.0)) at 4 °C for 120 min in the dark. After this treatment, comet slides were allowed to equilibrate in electrophoresis buffer for 2 × 15 min at 4 °C in the dark. The migration was performed in EDTA 2 mM NaOH 30 mM (pH 12.3) buffer at 40 V for 15 min. After migration, the slides were rinsed with water neutralized with 0.4 M Tris (pH 7.5) and fixed 5 min in 70% ethanol. After the slides were stained with SYBR Safe (X1000; Invitrogen). Images were acquired with a Nikon fluorescence microscope. Tail moments (¼ tail length DNA in the tail/total DNA) were analyzed using the Casplab freeware^27^.

### Immunofluorescence staining

Six days after infection in eight‐chamber tissue culture slides (Falcon, Corning), cells were fixed in ice‐cold methanol for 10 min at −20 °C and blocked in 0.05% PBS‐Tween containing 20% FBS (PBST‐FBS). Incubation with primary antibodies in PBST‐FBS was performed overnight at 4 °C. Primary antibodies and dilutions used are listed in Supplementary Fig. 2. After three washes with PBS-Tween, the slides were incubated with Alexa Fluor 488 dye‐conjugated goat anti‐rabbit antibody or chicken anti‐mouse antibody diluted in PBST‐FBS for 1 h at room temperature. The slides were then washed with PBS-Tween and mounted with DAPI Fluoromount G (SouthernBiotech). Images were acquired with a Nikon fluorescence microscope. Number of foci per nucleus was determined using the Focinator tool^28^.

### Statistical analysis

All statistical analysis and graphs, presented as mean between three independent experiments with SEM, were created with GraphPad Prism 7.03, unless indicated otherwise in figure legends. Two-tailed unpaired Student’s *t*-test was used to determine the *p* value unless indicated otherwise in the figure legends. *p < 0.05; **p < 0.01; ***p < 0.001.

### Gene set enrichment analysis

PLA2R1 co-expression correlation values and statistics for all genes of the genome were generated with the SEEK analysis tool (http://seek.princeton.edu/) using Multiple Tissue Profiling. Co-expression values were multiplied by the *p* value to obtain ranked values. This preranked file was run with the GSEA Preranked module against CGP (Chemical and Genetic Perturbation) from Curated gene sets, against BP (Biological Pathway) from GO Gene sets or against Hallmark gene sets using default parameters.

### Mice

C57/B6 PLA2R1 wild type and PLA2R1 knockout mice, both male and female, were used for experiments. Pla2r1 knockout mice were described and genotyped as in ref. ^29^. Twelve and 20-month-old mice were sacrificed and tissues of interest removed and analyzed (macroscopic lesions, skin and spleen). Mice were maintained in a specific pathogen free (SPF) animal facility platform, Anican, at the Cancer Research Center of Lyon. The experiments were conducted in accordance with the animal care guidelines of the European Union and French laws. Protocols were approved by the local Animal Ethic Evaluation Committee (CECCAPP: C2EA-15) and authorized by the French Ministry of Education and Research.

### Immunohistochemistry

Paraffin-embedded murine tissues were used. Slides were serially sectioned at 3 μm thickness. After deparaffinization and rehydration, the slides were incubated in 3% hydrogen peroxide in distilled water to block endogenous peroxidases. For heat-induced antigen retrieval, tissue sections were boiled in 10 mmol/L citrate buffer pH 6.0 in a microwave oven for 15 min. The slides were then incubated for 30 min with low-background antibody diluent (DAKO Real) to block unspecific antigen sites. They were then incubated at 4 °C overnight with the primary antibody (listed in Supplementary Fig. 2) diluted in the “low-background” antibody diluent (DAKO Real). After rinsing in PBS, the slides were incubated with a biotinylated secondary antibody bound to a streptavidin peroxidase conjugate (Dako E0468) for 1 h at room temperature. Slides were treated with Streptavidine HRP (Vector) and the bound antibody was then detected using the DAB peroxidase substrate kit (Vector). Sections were counterstained with hematoxylin and the slides were finally dehydrated and mounted. At least 500 cells taken from five independent fields were quantified per mouse. Mice were identified with a number and results were acquired without knowing the genotypes by the experimentator.

## Results

### Loss of *Pla2r1* increases spontaneous tumor formation in aged mice

**Fig. 1:**
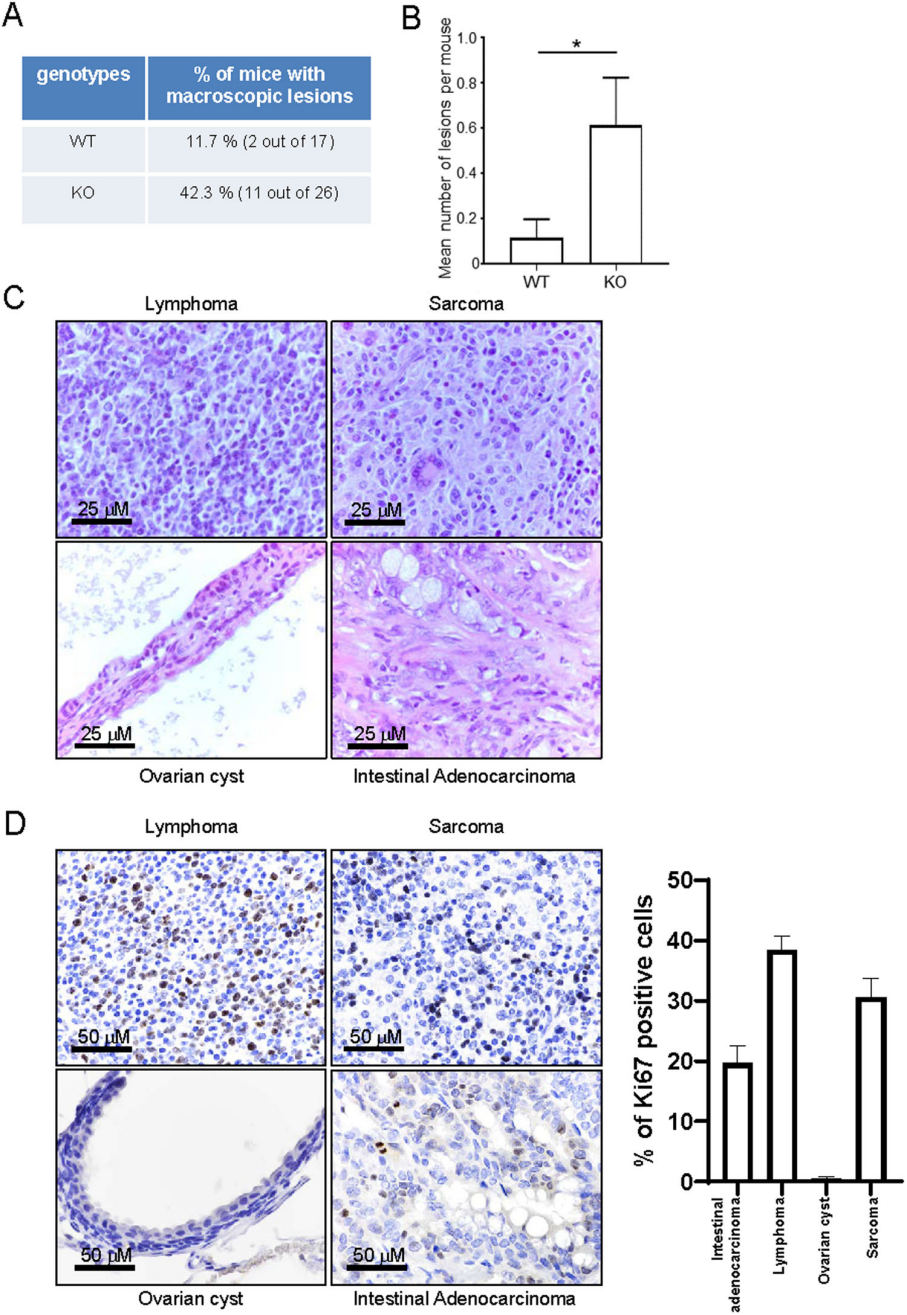
*Pla2r1* loss increases spontaneous tumor formation in old mice. **A–C** Macroscopic lesions of 20-month-old Pla2r1−/− mice (*n* = 26) and their control littermates (*n* = 17) were counted and analyzed histologically to characterize each lesion. **A** Percentage (%) of mice displaying macroscopic lesions for each genotype was calculated. *P* = 0.04 according to two-tailed Fisher exact test. **B** The mean number of lesions per mouse for each genotype was computed and presented as mean ± SEM. The *p* value was calculated using Mann–Whitney test. *p < 0.05. **C** Representative histopathological images of lesions are shown. **D** Indicated tumors were prepared and analyzed by immunohistochemistry against Ki67 proliferation marker to determine the percentage of positive cells. Representative images and histograms are shown (mean ± SD).

To investigate whether PLA2R1 regulates spontaneous tumor formation during aging, we generated mice cohorts composed of wild type (WT) and *Pla2r1* knockout littermates. We sacrificed 12-month-old mice and examined skin, pancreas, colon, kidney, lung and mammary gland tissue sections. Two *Pla2r1* KO mice displayed tumors, a lung adenoma, and a colon adenoma (Supplementary Fig. 3), suggesting an increased sensibility of *Pla2r1* KO mice to develop tumors with aging. To confirm and extend these results older mice were sacrificed at 20 months, prior to conducting macroscopic analyses. Approximately half of the *Pla2r1* KO mice displayed macroscopic lesions, whereas only 11% of the control littermates displayed such lesions (Fig. 1A). Moreover, the number of lesions per mouse was also higher in KO mice (Fig. 1B). Anatomo-pathological examination of these lesions confirmed their tumoral origin, though both benign and malignant lesions were observed (Table 1, Fig. 1C and Supplementary Fig. 4), malignant lesions displaying higher Ki67 proliferation marker staining when compared to benign lesions (Fig. 1D). Anatomo-pathological examination on tissue sections of breast, kidney, pancreas, lung, colon, liver and thymus did not reveal additional increase of other neoplastic lesions in 20-month-old KO mice when compared to WT littermates. These results suggest that *Pla2r1* protects mice from tumor formation during aging.

**Table 1:** Macroscopic lesions observed in 20-month-old mice.

Genotype and gender	Lesion type
WT—F	Benign serous ovarian cyst
WT—M	Lymphoma
KO—F	Ovarian cystadenoma
KO—F	Benign serous ovarian cyst
KO—M	Ovarian cystadenoma
	Lymphoma
	Metastatic lymphoma in the liver
KO—F	Periportal multifocal invasion by lymphoid proliferation in the liver. Probably lymphoma
KO—F	Periportal multifocal invasion by lymphoid proliferation in the liver. Probably lymphoma
KO—M	Lymphoma
KO—M	Intestinal adenocarcinoma
KO—M	Lymphoma and hystiocytic sarcoma
	Lymphoma
	Lymphoma
	Lymphoma
	Lymphoma
KO—F	Benign serous ovarian cyst
KO—F	Benign serous ovarian cyst
KO—F	Benign serous ovarian cyst

### PLA2R1 expression is correlated with loss of a DNA repair signature, decreased PARP1 expression, and increased DNA damage

**Fig. 2:**
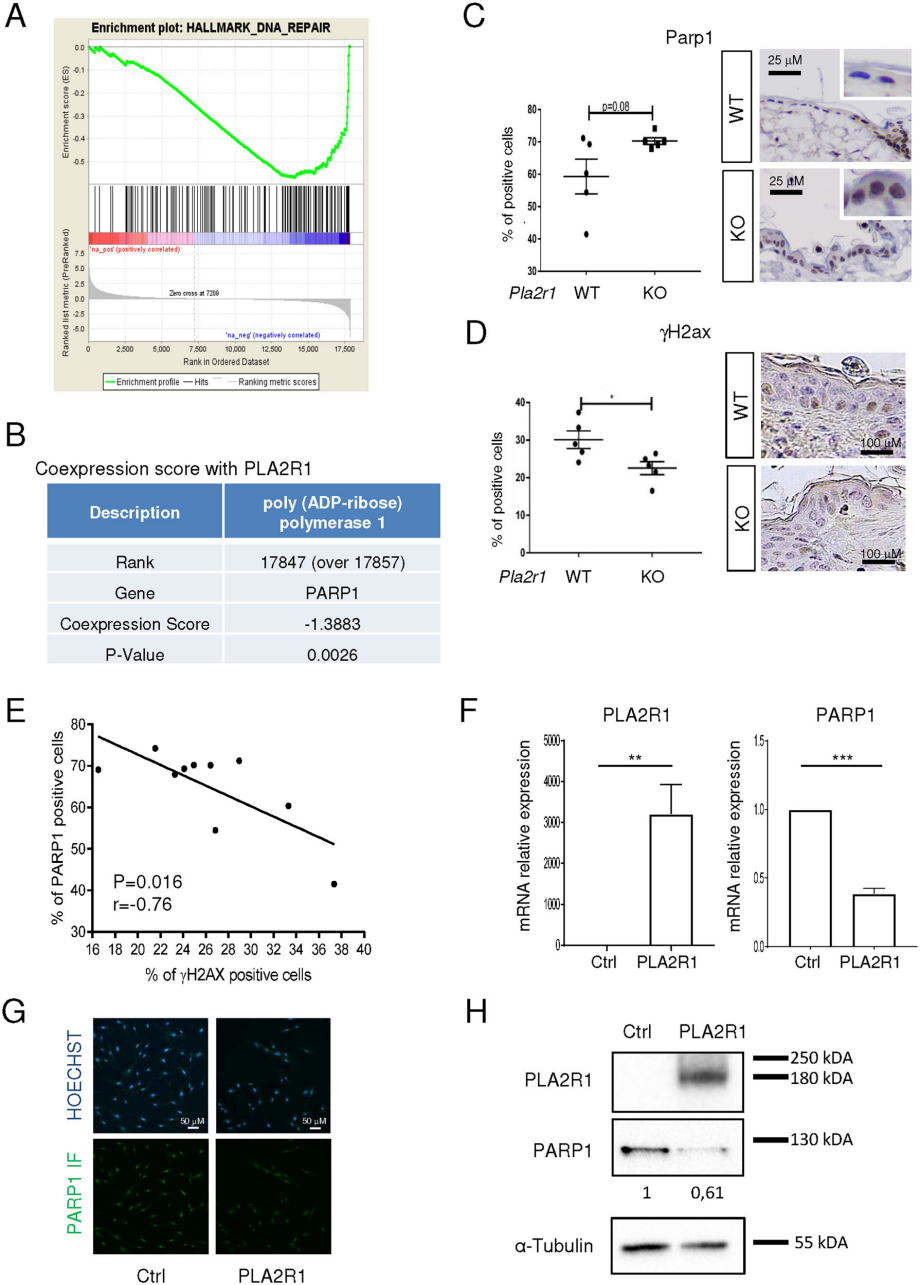
PLA2R1 represses DNA repair gene PARP1 and correlates with DNA damage. **A** Correlation between the expression of all genes of the genome and PLA2R1 in human samples was generated using SEEK analysis tool (http://seek.princeton.edu/). Gene Set Enrichment Analysis (GSEA) was used to investigate signatures showing a correlation with PLA2R1 expression. Graph displays negative enrichment of the gene set “DNA repair hallmark” in PLA2R1 correlated genes (FDR *q* val < 0.001). **B** Table displays the inverse correlation between the expressions of PARP1 and PLA2R1 using the SEEK analysis tool. **C**, **D** Skin from 20-month-old WT (*n* = 5) and Pla2r1 KO (*n* = 5) mice was prepared and analyzed by immunohistochemistry against γH2AX (**C**) or PARP1 to determine the percentage of Parp1-positive cells (**D**). Results are presented as mean ± SEM. *P* value was calculated using Student’s *t*-test. *p < 0.05. **E** Correlation between proportion of PARP1-positive cells and γH2AX-positive cells in the skin of 20-month-old mice (*n* = 10). Pearson test was used for statistical analysis. **F–H** MRC5 normal human cells were infected with a control or a PLA2R1-encoding retroviral vector and selected. Between 5 and 7 days after infection, **F** RNA was prepared and RTqPCR performed against PLA2R1 or PARP1 and normalized against GAPDH levels. Results are shown as mean ± SEM (*n* = 6). *P* value was calculated using Student’s *t*-test. **p < 0.01; ***p < 0.001. **G** Cells were fixed in methanol and immunofluorescence staining against PARP1 was performed. Nucleus counterstaining using Hoechst dye was performed. Representative images of three experiments are shown. **H** Cell extracts were prepared and western blot analysis was performed using the indicated antibodies. Quantification for PARP1 levels normalized against tubulin was performed with ImageJ. Representative images of three experiments are shown.

Accumulation of DNA damage is known to be induced by oncogenic signals where it can contribute to tumor suppression through activation of its downstream signaling resulting in p53 activation^4,30^. PLA2R1 has been shown to regulate DNA damage accumulation in vitro^16^. Still little is known about the molecular, cellular, and physiological functions of PLA2R1 in humans^14^. To gain further insight into its function, we explored the computational gene co-expression search engine SEEK (Search-Based Exploration of Expression Compendium) which contains massive human expression compendium that contains thousands of expression datasets to define genes and pathways co- or inversely expressed with PLA2R1. By this means, we obtained a full list of genes correlated or inversely correlated with PLA2R1 expression (Supplementary Table 1), and subsequently used this list to perform Gene Set Enrichment Analysis (GSEA). These analyses revealed a strong inverse correlation between PLA2R1 levels and the expression of a DNA repair signature (Fig. 2A). PARP1 was one of the top-ranking inversely correlated genes uncovered in that signature, as well as globally in the whole transcriptome (Fig. 2B and Supplementary Table 1). PARP1 is involved in DNA repair^31^. It is known to be downregulated during senescence and when ectopically expressed is able to overcome DNA damage and senescence, furthermore its inhibition promotes cell death^12,32^. PLA2R1 exerts quite opposite effects as it is induced during senescence^16^ and its downregulation decreases DNA damage and senescence^16^, as well as it promotes cell death when ectopically expressed^17^. Because of inverse correlation between these two genes and opposition in their functional effects, we further explored the link between PLA2R1 and PARP1 by examining the level of Parp1 in WT and *Pla2r1* KO mice. We found that 20-month-old *Pla2r1* KO mice had a tendency toward higher levels of Parp1 (Fig. 2C) and displayed less DNA damage according to the decreased percentage of γH2AX-positive cells (Fig. 2D). These differences were not seen in 12-month-old mice (Supplementary Fig. 5), suggesting that these differences took place with aging. In addition, an increase in Parp1 was correlated with a decrease in γH2AX levels in old mice (Fig. 2E). Next, PLA2R1 was constitutively expressed in normal human cells, where it is known to induce premature senescence and DNA damage^16^. Its constitutive expression decreased both PARP1 mRNA and protein levels (Fig. 2F–H).

Overall, these results indicate that PLA2R1 is involved in the reduction of expression of the DNA repair factor, PARP1.

### PARP1 constitutive expression rescues PLA2R1-induced DNA damage and senescence

**Fig. 3:**
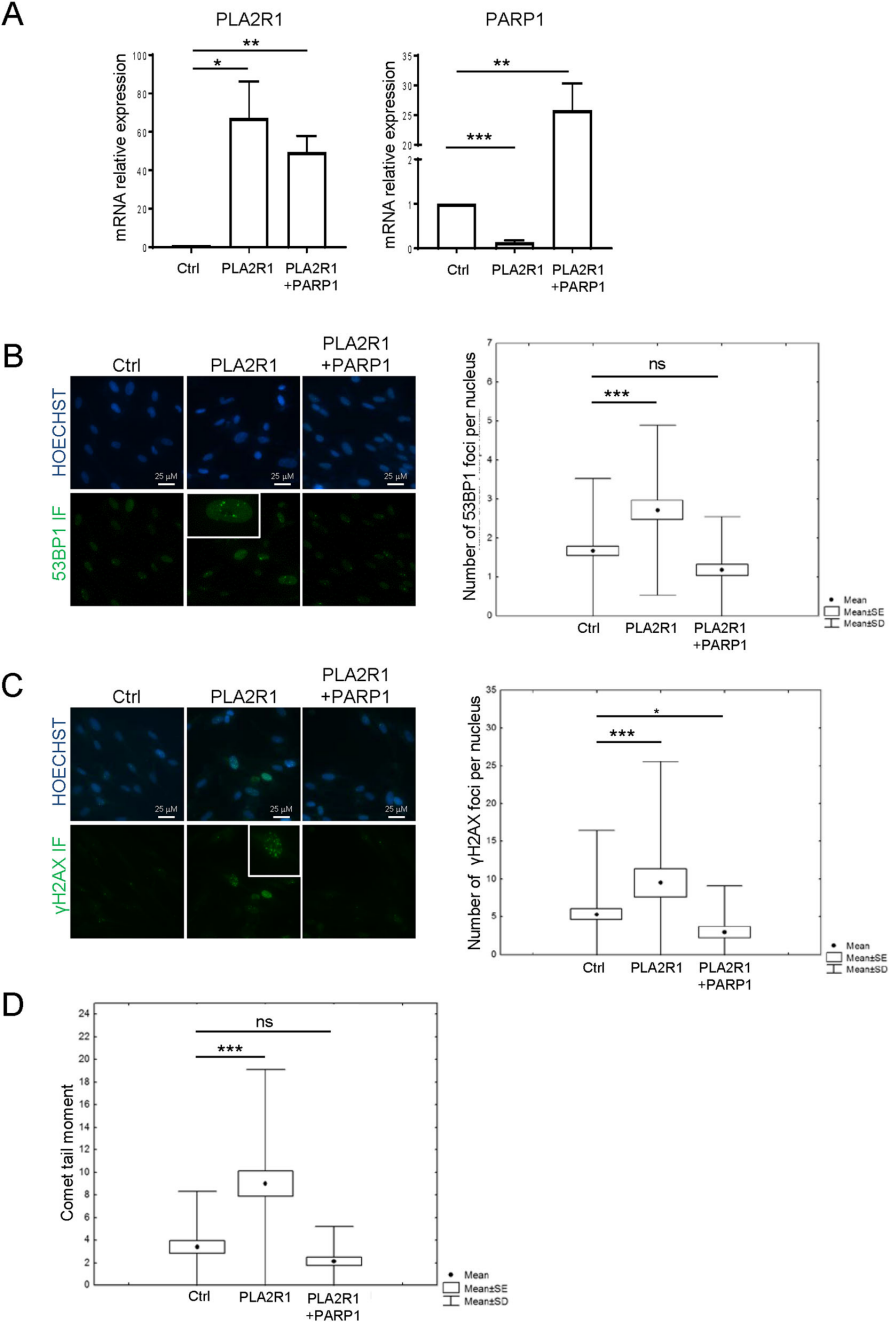
PARP1 expression abolishes DNA damage induced by PLA2R1. **A–D** MRC5 normal human cells were infected with a control, PLA2R1-, or PARP1-encoding retroviral vectors as indicated and selected. **A** RNA was prepared and RTqPCR performed against PLA2R1 or PARP1 and normalized against GAPDH levels. The results shown are mean ± SEM of three independent experiments. *P* value was calculated using Student’s *t*-test. *p < 0.05; **p < 0.01; ***p < 0.001. **B**, **C** Cells were fixed in methanol and immunofluorescence staining against 53BP1 (**B**) and γH2AX (**C**) was performed with indicated antibodies. Images were taken using a Nikon Fluorescence microscope and quantified with the Focinator software. More than 50 nuclei were analyzed per condition. The *p* value was calculated using the Mann–Whitney test. *p < 0.05; **p < 0.01; ***p < 0.001. **D** Comet assay for assessing DNA breaks was performed. After migration, the slides were stained with SYBR Safe. Tail moments (1/4 tail length DNA in the tail/total DNA) were analyzed using the Casplab software. A least 50 nuclei were analyzed per condition. The *p* value was calculated using the Mann–Whitney test, ***p < 0.001.

Having shown that expression of PARP1 is reduced when PLA2R1 is increased, we wondered what the functional consequences of this reduction were on PLA2R1-induced DNA damage and could we revert it. To address this issue, we performed rescue experiments by ectopically expressing PARP1 in PLA2R1 constitutively expressing normal human cells (Fig. 3A).

**Fig. 4:**
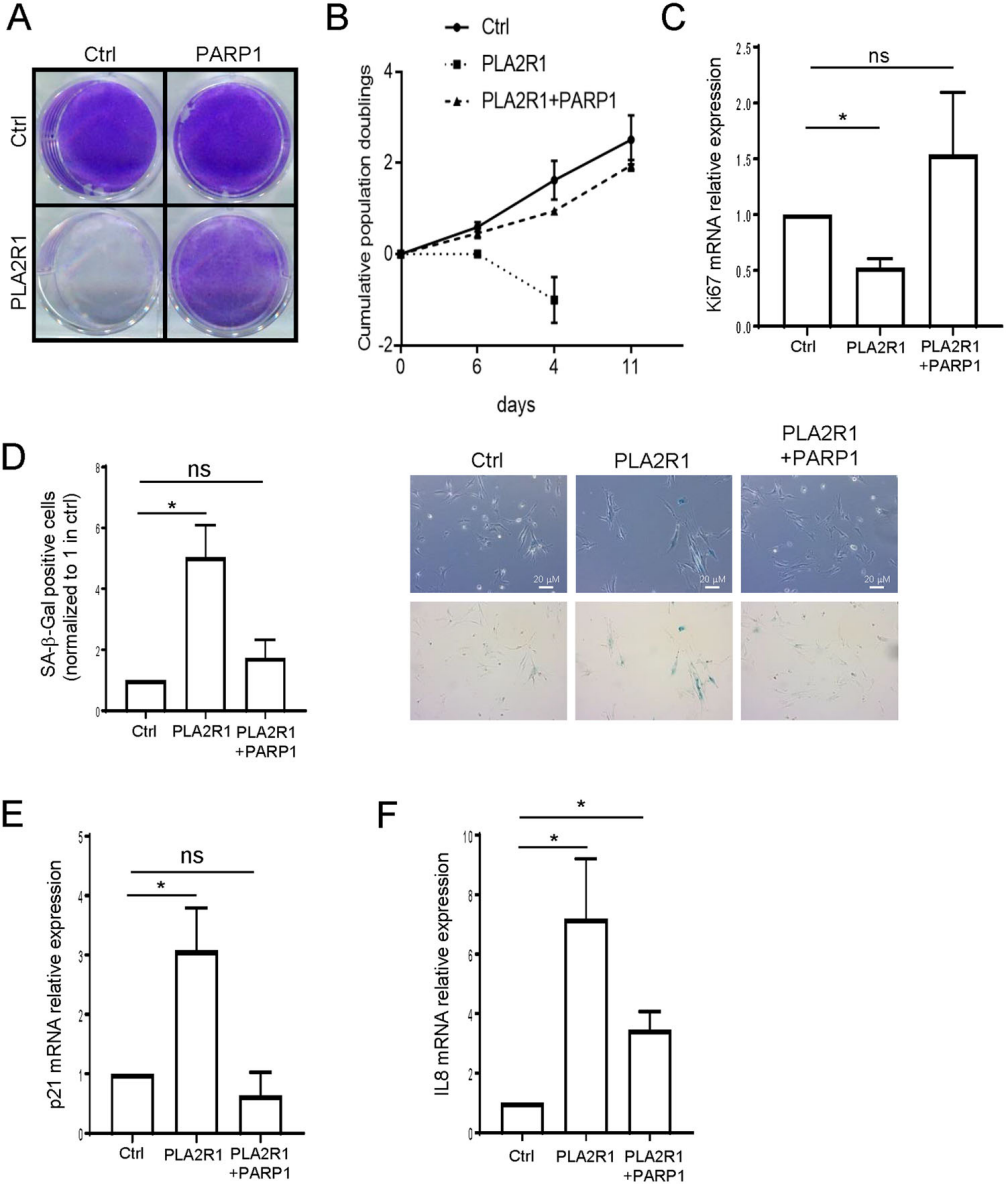
PARP1 expression inhibits senescence induced by PLA2R1. E–F MRC5 cells were infected with control, PLA2R1-, or PARP1-encoding retroviral vectors as indicated, and selected. **A** Fourteen days after seeding, cells were fixed and stained by crystal violet to visualize cell density. **B** The growth curve was performed by counting the number of cells and calculating the population doublings at each cell passage. Representative growth curves from three experiments with SD are shown. **C** RNA was prepared and RTqPCR performed against the proliferation marker Ki67 and normalized against GAPDH levels (*n* = 3). **D** Cells were fixed and a SA‐β‐galactosidase assay was performed. Percentage of SA-β-Gal positive cells was calculated and representative images are shown (*n* = 3). **E**, **F** RTqPCR performed against p21 (**E**) and IL8 (**F**) and normalized against GAPDH levels (*n* = 3). Graphs **C**–**F** show mean between three experiments ± SEM and *p* value was calculated using Student’s *t*-test. *p < 0.05.

Quantitative analysis of DNA damage was performed by examining recruitment of 53BP1 and recruitment/phosphorylation of γH2AX factors at DNA damaged sites. As expected, increased PLA2R1 expression induced an augmentation in the number of both 53BP1 and γH2AX foci, which were reverted by the ectopic expression of PARP1 (Fig. 3B, C). Furthermore, Comet assay to directly measure DNA breaks confirmed that PLA2R1 constitutive expression induced DNA damage, whereas PARP1 rescued this PLA2R1 effect (Fig. 3D). As PARP1 rescued PLA2R1-induced DNA damage, we examined whether this also rescued PLA2R1-induced cellular senescence. Indeed, the ectopic expression of PARP1 rescued PLA2R1-induced growth arrest (Fig. 4A–C) and PLA2R1-induced senescence markers, namely SA-β-Gal activity (Fig. 4D) as well as the increased expression of the p53 target, p21, and of IL8 (Fig. 4E, F).

Altogether, these results support that PARP1 decrease is critical for the mediation of the PLA2R1 effects on DNA damage and senescence.

### PARP1 transcription is repressed by a ROS–Rb pathway in PLA2R1-expressing cells

**Fig. 5:**
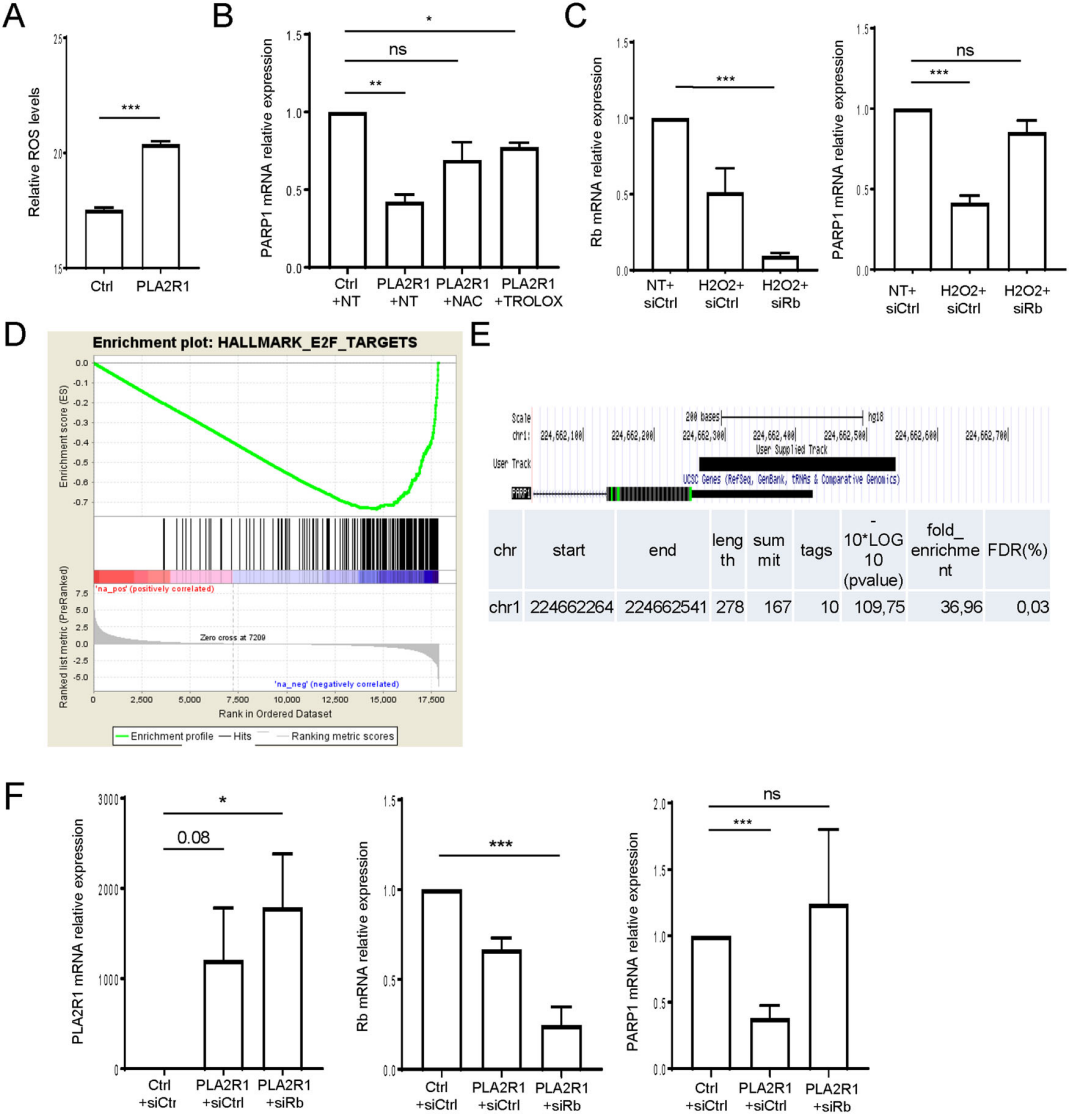
PARP1 expression is repressed by a ROS/Rb pathway. **A** MRC5 cells were infected with roGFP2-ORP1, a ratiometric ROS probe, encoding retroviral vector and a control or PLA2R1-encoding retroviral vectors and selected. Images were taken by Operetta system and quantified by Columbus software. The results are shown as representative from three experiments, mean ± SEM between three wells. *P* value was calculated using Student’s *t*-test ***p < 0.001. **B** MRC5 cells were infected with a control or a PLA2R1-encoding retroviral vector and selected. Cells were treated with NAC or Trolox daily during 3 days before analysis. Levels of PARP1 were quantified by RTqPCR and normalized against the level of GAPDH. The results are shown as mean ± SEM (*n* = 2). *P* value was calculated using Student’s *t*-test. *p < 0.05; **p < 0.01. **C** MRC5 cells where transfected with a control nontargeting pool (siCtrl) or siRNA pools targeting Rb. Two days after transfection cells were treated with H_2_O_2_; mRNA levels of Rb and PARP1 were quantified by RTqPCR 24 h later. Results were normalized against the level of GAPDH mRNA. The results shown are mean ± SEM (*n* = 4) and *p* value was calculated using Student’s *t*-test **p < 0.01; ***p < 0.001. **D** GSEA analysis was performed on PLA2R1 co-expressed genes. Graph displays negative enrichment of E2F target genes between PLA2R1 correlated genes (FDR *q* val < 0.001). **E** ChiP seq data^35^ against Rb on senescent human fibroblasts were analyzed to reveal a binding of Rb to the PARP1 promoter. **F** MRC5 cells where transfected with a control nontargeting pool (siCtrl) or siRNA pools targeting Rb. The following day, cells were infected with a control or a PLA2R1-encoding retroviral vector and selected. mRNA levels of PLA2R1 (left), Rb (middle), and PARP1 (right) were quantified by RTqPCR. Results were normalized against the level of GAPDH mRNA. Mean ± SEM (*n* = 4) are shown and *p* value was calculated using Student’s *t*-test. *p < 0.05; ***p < 0.001.

Next, we investigated the mechanism leading to a decrease in PARP1 levels following PLA2R1 signaling. ROS are key mediators of DNA damage and cellular senescence^33^. They have previously been described to be involved in the downregulation of PARP1 in the context of senescence^12^. Although how they are mediating PARP1 downregulation remains unknown. Moreover, ROS and the ensuing response are induced by PLA2R1 and are involved in PLA2R1-mediated senescence or cell death (Fig. 5A)^16,17^. In addition, human dataset analysis showed that PLA2R1 expression was strongly correlated with the GO signature “Response to oxidative stress” (Supplementary Fig. 6) further supporting a link between PLA2R1 and ROS production. To examine whether ROS induced by PLA2R1 are involved in the repression of PARP1, we treated PLA2R1 ectopically expressing cells with two different antioxidants NAC and Trolox. Both compounds decreased PARP1 downregulation induced by PLA2R1 (Fig. 5B). Furthermore, H_2_O_2_ treatment mimicked the constitutive expression of PLA2R1 as it decreased PARP1 levels (Fig. 5C). Using human datasets, we found that PLA2R1 expression was inversely correlated with the signature “E2F targets” (Fig. 5D). Rb inhibits E2F transcriptional activities and participates in PLA2R1-induced senescence^16,17^. Rb and E2F transcription factors are both master regulators of cellular senescence and of the expression of DNA repair genes^34^. We identified that Rb was bound to the PARP1 promoter in senescent cells (Fig. 5E), according to chromatin immunoprecipitation seq experiments^35^. Knockdown of Rb reverted PARP1 decrease induced by H_2_O_2_ treatment (Fig. 5C) or by the constitutive expression of PLA2R1 (Fig. 5F), further highlighting a role for Rb in repressing PARP1 expression in senescent cells.

Hence, our results support the hypothesis that Rb directly represses PARP1 expression during PLA2R1-ROS-induced senescence.

## Discussion

In this study, we demonstrated the role of PLA2R1 in tumor suppression during aging. First and foremost, loss of *Pla2r1* in mice resulted in increased tumor formation in old animals. This effect was most probably linked to decreased ROS, DNA damage, and DNA damage signaling. Mechanistically, PLA2R1 promotes repression of the PARP1 DNA repair protein, an increase in DNA damage and cellular senescence, which are dependent of a ROS–Rb pathway.

Even if we have focused on the relationship between PLA2R1 and PARP1, we can speculate that other DNA repair pathways are linked to PLA2R1 and its effects. Indeed, analysis of multiple human datasets using SEEK database support a strong anti-correlation between PLA2R1 levels and the Fanconi anemia pathway (Supplementary Table 2).

The role of DNA damage on cancer initiation is complex. According to our results, PLA2R1 could protect cells from tumor initiation during aging by promoting DNA damage and its downstream signaling such as activation of p53. In the context of PLA2R1 and beyond, p53 activation could result in cell death or/and cellular senescence^16,36^. This corroborates previous observations that DNA damage response acts as an anti-tumoral barrier^4,6^. Nevertheless this initial anti-tumoral barrier could constitute a selective process, promoted by DNA instability associated with DNA damage, to produce gene mutations promoting escape from cell death and senescence and tumor formation, such as mutations affecting the p53 pathway^7^. Consistently, mutations or loss-of-functions of multiple DNA repair genes, which promote chronic DNA damage, cell death, senescence, and instability promote long-term tumor formation^10–12,34,37^. In the balance between pro-tumoral effects of DNA damage and subsequent genetic instability, and DNA damage signaling anti-tumoral effects, the latter seems to prevail in the context of PLA2R1.

We identified ROS as critical regulators of PLA2R1-induced DNA damage. ROS are known to induce genomic instability and are often thought to exert adverse effects on tumor initiation and progression as well as on other age-related diseases^8^. These speculations support the rationale to add antioxidants in anti-aging pills, which are quite popular in today’s society. Nevertheless, these ideas have been challenged as anti-oxidant treatments might promote tumor aggressiveness^24,38,39^. As accumulation of ROS in the context of PLA2R1 induces DNA damage, cell death and cellular senescence^16,17,33^ and as DNA damage and its signaling, cell death and senescence can display tumor suppressive effects, it raises the question of whether ROS could exert tumor suppressive effects, as suggested by our data. In that case, ROS could be viewed as a messenger driving DNA damage and its downstream pathway, such as p53, and subsequent cell death and cellular senescence to suppress tumor formation. In line with this speculation, some epidemiological studies suggest deleterious effect of anti-oxidant supplements in the context of some age-related diseases, including cancer^40,41^ and one functional study suggests that NAC anti-oxidant treatment in mice promotes tumor formation during normal and accelerated aging, while it reduces some other marks of aging^5^.

ROS-induced DNA damage is largely thought to be formed due to the direct attack of DNA by ROS^42,43^. Our results suggest a more complex view where defects in DNA repair capacity by decreasing PARP1 levels largely contribute to DNA damage levels. The active role of the loss of additional DNA repair genes has already been shown in different senescence contexts^12,34^. We can speculate that decreased expression of DNA repair genes, like PARP1, reinforces and sustains DNA damage and its tumor suppressive signaling which in turn prevents damaged cells to resume their proliferation and/or induce cell death. In addition, it is possible that a decreased PARP1 avoids error prone activity of re-ligation driven by PARP1 (refs. ^44,45^), avoiding DNA instability and subsequent pro-tumoral effects.

Our findings also support that PLA2R1–ROS pathway activates Rb, which can bind and repress *PARP1* gene expression. The underlying mechanism regulating Rb activation by ROS may be mediated by the induction of cyclin-dependent kinase inhibitor such as p16 and p21 or other pathways^46,47^.

In conclusion, our work sheds light on the tumor suppressive effect of PLA2R1 during aging and its downstream effectors: ROS, PARP1, and DNA damage and its downstream tumor suppressive pathway. It also contributes to emphasizing the complex role of ROS and DNA damage that can exert both pro- and anti-tumoral effects.

## Supplementary information

Supplementary Figure and Table legends

Supplementary Table 1

Supplementary Table 2

Supplementary Figure 1

Supplementary Figure 2

Supplementary Figure 3

Supplementary Figure 4

Supplementary Figure 5

Supplementary Figure 6
