# Supplementary material for: PLA2R1 promotes DNA damage and inhibits spontaneous tumor formation during aging
Source: Cell Death Dis. 2021 Feb 16;12(2):190. doi: 10.1038/s41419-021-03468-3 (PMC7887270; doi:10.1038/s41419-021-03468-3)
Supplement: Supplementary file 1 — Supplementary Figure and Table legends [file 41419_2021_3468_MOESM1_ESM.docx]

**Supplemental Figure and Table Legends**

**Supplemental Fig. 1** List of primers and UPL probes.

**Supplemental Fig. 2** List of primary antibodies

**Supplemental Fig. 3 a** Lung Adenoma and **b** colon adenoma in 12 months old Pla2r1 KO mice. Tissues were removed, fixed and included. Sections were H&E stained and examined by a pathologist for presence of tumors. Ten WT mice and 10 KO mice were examined.

**Supplemental Fig. 4 a** Proportion of males and females in the cohorts. **b-c** Examples of tumors identified. Macroscopic lesions were removed, fixed and included. Sections were H&E stained and examined by a pathologist to define the tumor type.

**Supplemental Fig. 5** Skin from 12-month old WT (n = 6) and Pla2r1 KO (n = 6) mice was prepared and analyzed by immunohistochemistry against PARP1 or γH2AX to determine the percentage of positive cells. Results are presented as mean +/- SEM. p-value was calculated using Student’s t-test.

**Supplemental Fig. 6** GSEA analysis was performed on PLA2R1 co-expressed genes. Correlation values for all genes of the genome were generated using the SEEK analysis tool (http://seek.princeton.edu/). Graph displays positive enrichment of the GO process: response to oxidative stress. FDR q-val<0.001.

**Supplemental Table 1** List of genes correlated or inversely correlated with PLA2R1 expression.

**Supplemental Table 2** Correlation between PLA2R1 and DNA repair genes.
