# Supplementary material for: PLA2R1 promotes DNA damage and inhibits spontaneous tumor formation during aging
Source: Cell Death Dis. 2021 Feb 16;12(2):190. doi: 10.1038/s41419-021-03468-3 (PMC7887270; doi:10.1038/s41419-021-03468-3)
Supplement: Supplementary file 2 — Supplementary Table 1 [file 41419_2021_3468_MOESM2_ESM.pdf]

Supplemental Table 1

Rank	Gene	Coexpression Score	P-Value	Description
1	B3GNT9	1.9088	0	UDP-GlcNAc:betaGal beta-1,3-N-acetylglucosaminyltransferase 9
2	OLFML2A	1.8833	0	olfactomedin-like 2A
3	CASC4	1.7175	0.0002	cancer susceptibility candidate 4
4	LRIG3	1.67	0	leucine-rich repeats and immunoglobulin-like domains 3
5	TMEM245	1.6425	0	transmembrane protein 245
6	LPCAT2	1.62	0	lysophosphatidylcholine acyltransferase 2
7	VEGFC	1.6125	0.0002	vascular endothelial growth factor C
8	RHBDF1	1.5992	0.0003	rhomboid 5 homolog 1 (Drosophila)
9	ZG16B	1.5987	0	zymogen granule protein 16 homolog B (rat)
10	SCARA5	1.5825	0	scavenger receptor class A, member 5 (putative)
11	NEAT1	1.5789	0.0007	nuclear paraspeckle assembly transcript 1 (non-protein coding)
12	DOCK1	1.5725	0.0006	dedicator of cytokinesis 1
13	FNDC1	1.5662	0	fibronectin type III domain containing 1
14	RGNEF	1.5422	0	190 kDa guanine nucleotide exchange factor
15	TNFRSF11B	1.515	0	tumor necrosis factor receptor superfamily, member 11b
16	IGFBP4	1.5083	0.0001	insulin-like growth factor binding protein 4
17	RBMS3	1.5017	0.004	RNA binding motif, single stranded interacting protein 3
18	ERBB2	1.4917	0.0006	v-erb-b2 erythroblastic leukemia viral oncogene homolog 2, neuro/glioblastoma derived oncogene homolog (avian)
19	LTBP2	1.475	0.0039	latent transforming growth factor beta binding protein 2
20	MYOF	1.4692	0.0025	myoferlin
21	SPTLC3	1.465	0	serine palmitoyltransferase, long chain base subunit 3
22	SUMF1	1.4612	0.0016	sulfatase modifying factor 1
23	SGMS2	1.455	0.0015	sphingomyelin synthase 2
24	TRIM6	1.4488	0	tripartite motif containing 6
25	NPR3	1.4458	0.0001	natriuretic peptide receptor C/guanylate cyclase C (atrionatriuretic peptide receptor C)
26	NFIA	1.435	0.001	nuclear factor I/A
27	PCMTD1	1.4325	0.019	protein-L-isoaspartate (D-aspartate) O-methyltransferase domain containing 1
28	AHNAK	1.4283	0.001	AHNAK nucleoprotein
29	GPX8	1.425	0.0028	glutathione peroxidase 8 (putative)
30	CA12	1.4242	0.0002	carbonic anhydrase XII
31	UACA	1.4225	0.0002	uveal autoantigen with coiled-coil domains and ankyrin repeats
32	THSD4	1.42	0.0003	thrombospondin, type I, domain containing 4
33	MYO1C	1.4108	0.0037	myosin IC
34	LINC00472	1.405	0	long intergenic non-protein coding RNA 472
35	NBEAL1	1.4043	0.0002	neurobeachin-like 1
36	CYBRD1	1.4033	0.0031	cytochrome b reductase 1
37	PKHD1L1	1.4025	0	polycystic kidney and hepatic disease 1 (autosomal recessive)-like 1
38	CCDC80	1.4013	0.0048	coiled-coil domain containing 80
39	SPRED1	1.3987	0.0017	sprouty-related, EVH1 domain containing 1
40	ANO1	1.3967	0	anoctamin 1, calcium activated chloride channel
41	TMED4	1.3963	0.0003	transmembrane emp24 protein transport domain containing 4
42	FLJ33630	1.3963	0.0003	uncharacterized LOC644873
43	COL14A1	1.3942	0.0028	collagen, type XIV, alpha 1
44	STC1	1.3933	0	stanniocalcin 1
45	RNF135	1.3913	0.0008	ring finger protein 135
46	KCTD11	1.3887	0.0001	potassium channel tetramerisation domain containing 11
47	PDGFC	1.3858	0.0016	platelet derived growth factor C
48	SH3D19	1.38	0.0035	SH3 domain containing 19
49	HFE	1.3792	0.0031	hemochromatosis
50	FAT4	1.3783	0.004	FAT tumor suppressor homolog 4 (Drosophila)
51	PPIC	1.375	0.0029	peptidylprolyl isomerase C (cyclophilin C)
52	ITPRIPL2	1.3713	0.001	inositol 1,4,5-trisphosphate receptor interacting protein-like 2
53	TSPAN31	1.3683	0.0026	tetraspanin 31
54	PTPN14	1.365	0.0004	protein tyrosine phosphatase, non-receptor type 14
55	SLC4A11	1.3625	0.0002	solute carrier family 4, sodium borate transporter, member 11
56	ERGIC1	1.355	0.0003	endoplasmic reticulum-golgi intermediate compartment (ERGIC) 1
57	CTGF	1.3533	0.0017	connective tissue growth factor
58	NTN4	1.3513	0.0002	netrin 4
59	GABRE	1.3433	0.0002	gamma-aminobutyric acid (GABA) A receptor, epsilon
60	GLIS3	1.3425	0.0002	GLIS family zinc finger 3
61	FDCSP	1.3412	0	follicular dendritic cell secreted protein
62	LRP10	1.34	0.0308	low density lipoprotein receptor-related protein 10
63	SELM	1.3388	0.0039	selenoprotein M
64	PTGR1	1.3375	0.0005	prostaglandin reductase 1
65	EFEMP1	1.3367	0.0042	EGF containing fibulin-like extracellular matrix protein 1
66	SLC16A4	1.335	0.0004	solute carrier family 16, member 4 (monocarboxylic acid transporter 5)
67	FOXE1	1.3342	0.0003	forkhead box E1 (thyroid transcription factor 2)
68	CFH	1.3325	0.0026	complement factor H
69	SLC17A5	1.33	0.0014	solute carrier family 17 (anion/sugar transporter), member 5
70	PROM2	1.3262	0.002	prominin 2
71	LATS2	1.3225	0.0062	LATS, large tumor suppressor, homolog 2 (Drosophila)
72	RIN2	1.3192	0.0031	Ras and Rab interactor 2
73	CTNND1	1.3171	0.0013	catenin (cadherin-associated protein), delta 1
74	TCTN3	1.3142	0.0008	tectonic family member 3
75	JUP	1.3125	0.0029	junction plakoglobin
76	FCHO2	1.3125	0.0168	FCH domain only 2
77	ABI3BP	1.3088	0.0012	ABI family, member 3 (NESH) binding protein
78	GLT8D2	1.3083	0.0098	glycosyltransferase 8 domain containing 2
79	CLN5	1.3	0.0094	ceroid-lipofuscinosis, neuronal 5
80	RAB18	1.2988	0.0497	RAB18, member RAS oncogene family
81	OSR1	1.2962	0	odd-skipped related 1 (Drosophila)
82	FAM114A1	1.2944	0.0049	family with sequence similarity 114, member A1

83	KCTD10	1.29	0.0029	potassium channel tetramerisation domain containing 10
84	PRICKLE1	1.2888	0.0001	prickle homolog 1 (Drosophila)
85	GNG12	1.2883	0.0063	guanine nucleotide binding protein (G protein), gamma 12
86	LUM	1.2783	0.0041	lumican
87	FZD1	1.2767	0.0003	frizzled family receptor 1
88	CPQ	1.2758	0.0113	carboxypeptidase Q
89	DIO2	1.275	0.003	deiodinase, iodothyronine, type II
90	LMNA	1.2708	0.0163	lamin A/C
91	ITGBL1	1.2692	0.0004	integrin, beta-like 1 (with EGF-like repeat domains)
92	TMEM168	1.2667	0.0073	transmembrane protein 168
93	DISP1	1.2663	0	dispatched homolog 1 (Drosophila)
94	HSPG2	1.2658	0.0164	heparan sulfate proteoglycan 2
95	SLC26A7	1.2637	0.0002	solute carrier family 26, member 7
96	LRP11	1.2587	0.0016	low density lipoprotein receptor-related protein 11
97	DAPL1	1.2587	0	death associated protein-like 1
98	CRISPLD1	1.2575	0.001	cysteine-rich secretory protein LCCL domain containing 1
99	GPX3	1.2558	0.0008	glutathione peroxidase 3 (plasma)
100	RARB	1.2542	0.0022	retinoic acid receptor, beta
101	YAP1	1.2525	0.0024	Yes-associated protein 1
102	NPNT	1.2513	0	nephronectin
103	ATP8B1	1.2487	0.0006	ATPase, aminophospholipid transporter, class I, type 8B, member 1
104	SLC35F5	1.2463	0.0271	solute carrier family 35, member F5
105	LEPROT	1.2442	0.0096	leptin receptor overlapping transcript
106	ESYT2	1.2437	0.0039	extended synaptotagmin-like protein 2
107	MXRA5	1.2425	0.0047	matrix-remodelling associated 5
108	MUC15	1.2413	0.0002	mucin 15, cell surface associated
109	SCUBE3	1.24	0.0003	signal peptide, CUB domain, EGF-like 3
110	IGFBP6	1.2333	0.005	insulin-like growth factor binding protein 6
111	FBN1	1.2333	0.0163	fibrillin 1
112	LAMB1	1.2275	0.0055	laminin, beta 1
113	TM9SF3	1.2267	0.0375	transmembrane 9 superfamily member 3
114	SYS1	1.2263	0.0006	SYS1 Golgi-localized integral membrane protein homolog (S. cerevisiae)
115	COL3A1	1.2233	0.0093	collagen, type III, alpha 1
116	ARSD	1.2233	0.0031	arylsulfatase D
117	LOC100127888	1.2214	0.0054	uncharacterized LOC100127888
118	TNKS1BP1	1.2213	0.003	tankyrase 1 binding protein 1, 182kDa
119	SOWAHC	1.2208	0.0002	sosondowah ankyrin repeat domain family member C
120	CTSO	1.22	0.0227	cathepsin O
121	C3orf55	1.2188	0.0001	chromosome 3 open reading frame 55
122	PCYOX1	1.2167	0.02	prenylcysteine oxidase 1
123	MARVELD1	1.2143	0.0051	MARVEL domain containing 1
124	TG	1.2142	0.0011	thyroglobulin
125	VIT	1.2138	0.0004	vitrin
126	GPR133	1.2125	0.0001	G protein-coupled receptor 133
127	FHDC1	1.2112	0.0004	FH2 domain containing 1
128	SYNPO2	1.21	0.0086	synaptopodin 2
129	GINM1	1.2075	0.0099	glycoprotein integral membrane 1
130	ZNF300P1	1.2014	0.0001	zinc finger protein 300 pseudogene 1
131	PDLIM5	1.1992	0.0027	PDZ and LIM domain 5
132	KIAA1217	1.1988	0.0011	KIAA1217
133	WDR72	1.1975	0.0005	WD repeat domain 72
134	ITGB5	1.1967	0.0063	integrin, beta 5
135	FAM198B	1.1967	0.0042	family with sequence similarity 198, member B
136	C5orf28	1.195	0.0157	chromosome 5 open reading frame 28
137	IL1RL2	1.1942	0.0004	interleukin 1 receptor-like 2
138	CLDN8	1.1942	0.0001	claudin 8
139	ACVR1	1.1933	0.0281	activin A receptor, type I
140	DCN	1.1917	0.0263	decorin
141	SLC25A43	1.1888	0.0027	solute carrier family 25, member 43
142	SAMD5	1.1875	0.0003	sterile alpha motif domain containing 5
143	GLI3	1.1858	0.0071	GLI family zinc finger 3
144	SNX33	1.185	0.009	sorting nexin 33
145	PAX8	1.1842	0.0304	paired box 8
146	CD151	1.1833	0.0215	CD151 molecule (Raph blood group)
147	LAMB2	1.18	0.0307	laminin, beta 2 (laminin S)
148	IRX2	1.18	0.0001	iroquois homeobox 2
149	S100A6	1.1792	0.0095	S100 calcium binding protein A6
150	TENC1	1.1758	0.0251	tensin like C1 domain containing phosphatase (tensin 2)
151	SHC1	1.1742	0.0114	SHC (Src homology 2 domain containing) transforming protein 1
152	FAM176C	1.17	0.0001	family with sequence similarity 176, member C
153	PIP	1.1683	0.0012	prolactin-induced protein
154	ADAMTS5	1.1683	0.0022	ADAM metallopeptidase with thrombospondin type 1 motif, 5
155	RRAS	1.1675	0.034	related RAS viral (r-ras) oncogene homolog
156	NID2	1.165	0.0042	nidogen 2 (osteonidogen)
157	HRCT1	1.165	0	histidine rich carboxyl terminus 1
158	FBLN5	1.1642	0.0065	fibulin 5
159	CAV2	1.1608	0.012	caveolin 2
160	TMEM159	1.1575	0.0008	transmembrane protein 159
161	RAPH1	1.1575	0.0033	Ras association (RalGDS/AF-6) and pleckstrin homology domains 1
162	ZDHHC1	1.1563	0.0017	zinc finger, DHHC-type containing 1
163	GSTO2	1.1563	0.0007	glutathione S-transferase omega 2
164	PALMD	1.15	0.0012	palmdelphin
165	BPIFA2	1.15	0.0175	BPI fold containing family A, member 2
166	C16orf89	1.1487	0.0006	chromosome 16 open reading frame 89

167	ST3GAL1	1.1467	0.0015	ST3 beta-galactoside alpha-2,3-sialyltransferase 1
168	DNASE1L1	1.1467	0.0051	deoxyribonuclease I-like 1
169	SIAE	1.145	0.0015	sialic acid acetylesterase
170	CRIM1	1.1433	0.0088	cysteine rich transmembrane BMP regulator 1 (chordin-like)
171	ARL4D	1.1417	0.0008	ADP-ribosylation factor-like 4D
172	CD24	1.1414	0.0007	CD24 molecule
173	SLC12A8	1.1408	0	solute carrier family 12 (potassium/chloride transporters), member 8
174	RAB34	1.14	0.0036	RAB34, member RAS oncogene family
175	DSC2	1.1375	0.0029	desmocollin 2
176	RNASE4	1.1342	0.0205	ribonuclease, RNase A family, 4
177	ITGA6	1.1342	0.0021	integrin, alpha 6
178	AFAP1L2	1.1337	0.0001	actin filament associated protein 1-like 2
179	ARHGAP29	1.1333	0.0051	Rho GTPase activating protein 29
180	PERP	1.1292	0.0032	PERP, TP53 apoptosis effector
181	VASN	1.1287	0.0058	vasorin
182	EVC2	1.1275	0.0002	Ellis van Creveld syndrome 2
183	TAGLN	1.1267	0.0136	transgelin
184	ARSJ	1.1267	0.0039	arylsulfatase family, member J
185	ACTA2	1.1225	0.0114	actin, alpha 2, smooth muscle, aorta
186	ECHDC2	1.1217	0.01	enoyl CoA hydratase domain containing 2
187	MAMDC2	1.1213	0.0009	MAM domain containing 2
188	ERLEC1	1.1213	0.0346	endoplasmic reticulum lectin 1
189	FAM3D	1.12	0.0103	family with sequence similarity 3, member D
190	C1S	1.1192	0.0194	complement component 1, s subcomponent
191	TMBIM1	1.1175	0.0217	transmembrane BAX inhibitor motif containing 1
192	FOXC1	1.1175	0.0013	forkhead box C1
193	FAM129B	1.1175	0.0238	family with sequence similarity 129, member B
194	KRT7	1.1167	0.0018	keratin 7
195	ELF5	1.1167	0.0007	E74-like factor 5 (ets domain transcription factor)
196	TMEM9	1.1163	0.008	transmembrane protein 9
197	LOXL4	1.1163	0.0026	lysyl oxidase-like 4
198	TINAGL1	1.1125	0.0022	tubulointerstitial nephritis antigen-like 1
199	IQGAP1	1.1125	0.0382	IQ motif containing GTPase activating protein 1
200	FOXQ1	1.1113	0.0005	forkhead box Q1
201	PDGFRB	1.1108	0.0217	platelet-derived growth factor receptor, beta polypeptide
202	WNT5A	1.1092	0.0011	wingless-type MMTV integration site family, member 5A
203	PQLC3	1.1075	0.0304	PQ loop repeat containing 3
204	ZNF585B	1.1067	0.0063	zinc finger protein 585B
205	KCNE4	1.1067	0.0021	potassium voltage-gated channel, Isk-related family, member 4
206	LAMC1	1.1058	0.0182	laminin, gamma 1 (formerly LAMB2)
207	LINC00271	1.1043	0	long intergenic non-protein coding RNA 271
208	HIBADH	1.1037	0.0207	3-hydroxyisobutyrate dehydrogenase
209	GJB2	1.1037	0.0007	gap junction protein, beta 2, 26kDa
210	CTSB	1.1025	0.0353	cathepsin B
211	ASPH	1.1025	0.0049	aspartate beta-hydroxylase
212	MAML3	1.1013	0.0004	mastermind-like 3 (Drosophila)
213	VPS36	1.1	0.0283	vacuolar protein sorting 36 homolog (S. cerevisiae)
214	C1orf210	1.1	0.0025	chromosome 1 open reading frame 210
215	EPB41L4A	1.0992	0.0026	erythrocyte membrane protein band 4.1 like 4A
216	SH3RF1	1.0987	0.0019	SH3 domain containing ring finger 1
217	USP53	1.0975	0.0179	ubiquitin specific peptidase 53
218	UBE2Q2	1.0963	0.0335	ubiquitin-conjugating enzyme E2Q family member 2
219	S100A2	1.0958	0.0032	S100 calcium binding protein A2
220	TRPC6	1.0933	0.0018	transient receptor potential cation channel, subfamily C, member 6
221	DEPTOR	1.0933	0.0015	DEP domain containing MTOR-interacting protein
222	CHMP3	1.0929	0.0048	charged multivesicular body protein 3
223	METRNL	1.0925	0.0115	meteorin, glial cell differentiation regulator-like
224	NFIB	1.09	0.0057	nuclear factor I/B
225	KCNK2	1.09	0.0016	potassium channel, subfamily K, member 2
226	CHST14	1.0888	0.0052	carbohydrate (N-acetylgalactosamine 4-0) sulfotransferase 14
227	PTRF	1.0875	0.0387	polymerase I and transcript release factor
228	KLK10	1.0875	0.0136	kallikrein-related peptidase 10
229	GLIS2	1.0875	0.0038	GLIS family zinc finger 2
230	AHR	1.0875	0.0206	aryl hydrocarbon receptor
231	ITGB1	1.0871	0.0031	integrin, beta 1 (fibronectin receptor, beta polypeptide, antigen CD29 includes MDF2, MSK12)
232	ALDH3A2	1.0867	0.0034	aldehyde dehydrogenase 3 family, member A2
233	PARVA	1.0858	0.0204	parvin, alpha
234	TSKU	1.085	0.0031	tsukushi small leucine rich proteoglycan homolog (Xenopus laevis)
235	COL4A4	1.085	0.0009	collagen, type IV, alpha 4
236	SSH3	1.0842	0.0234	slingshot homolog 3 (Drosophila)
237	TMEM139	1.0838	0.0022	transmembrane protein 139
238	TFCP2L1	1.0833	0.0027	transcription factor CP2-like 1
239	LRRN4CL	1.0829	0.009	LRRN4 C-terminal like
240	CCL28	1.0825	0.0004	chemokine (C-C motif) ligand 28
241	SELENBP1	1.08	0.0064	selenium binding protein 1
242	COPZ2	1.08	0.0232	coatomer protein complex, subunit zeta 2
243	CDC42EP5	1.0788	0.0062	CDC42 effector protein (Rho GTPase binding) 5
244	CYR61	1.0783	0.0162	cysteine-rich, angiogenic inducer, 61
245	C15orf52	1.0775	0.0031	chromosome 15 open reading frame 52
246	LIMA1	1.0758	0.0079	LIM domain and actin binding 1
247	EGF	1.0758	0	epidermal growth factor
248	ARHGEF12	1.0758	0.0142	Rho guanine nucleotide exchange factor (GEF) 12
249	TM9SF1	1.075	0.0382	transmembrane 9 superfamily member 1
250	TANC1	1.075	0.008	tetratricopeptide repeat, ankyrin repeat and coiled-coil containing 1

251	PLXNB2	1.0742	0.0461	plexin B2
252	SLC40A1	1.0738	0.0082	solute carrier family 40 (iron-regulated transporter), member 1
253	PDE1A	1.0733	0.0051	phosphodiesterase 1A, calmodulin-dependent
254	KLF5	1.0733	0.0044	Kruppel-like factor 5 (intestinal)
255	KCNJ15	1.0725	0.0052	potassium inwardly-rectifying channel, subfamily J, member 15
256	EBF1	1.0725	0.0092	early B-cell factor 1
257	CYP1B1	1.0725	0.0027	cytochrome P450, family 1, subfamily B, polypeptide 1
258	ITPRIP	1.0712	0.0166	inositol 1,4,5-trisphosphate receptor interacting protein
259	ECM2	1.0692	0.0189	extracellular matrix protein 2, female organ and adipocyte specific
260	NOSTRIN	1.0688	0.0019	nitric oxide synthase trafficker
261	MALAT1	1.0688	0.0199	metastasis associated lung adenocarcinoma transcript 1 (non-protein coding)
262	ARRDC3	1.0675	0.0147	arrestin domain containing 3
263	SLC16A5	1.0667	0.0038	solute carrier family 16, member 5 (monocarboxylic acid transporter 6)
264	EFEMP2	1.0658	0.0484	EGF containing fibulin-like extracellular matrix protein 2
265	MIR205HG	1.0657	0.0015	MIR205 host gene (non-protein coding)
266	LINC00597	1.065	0.0085	long intergenic non-protein coding RNA 597
267	PLSCR4	1.0642	0.0395	phospholipid scramblase 4
268	EXT1	1.0633	0.0075	exostosin 1
269	MET	1.0625	0.0088	met proto-oncogene (hepatocyte growth factor receptor)
270	SLC39A1	1.0617	0.0046	solute carrier family 39 (zinc transporter), member 1
271	CDH1	1.0617	0.006	cadherin 1, type 1, E-cadherin (epithelial)
272	CCDC8	1.0612	0.0059	coiled-coil domain containing 8
273	MANBAL	1.06	0.0125	mannosidase, beta A, lysosomal-like
274	MGP	1.0583	0.0077	matrix Gla protein
275	ITGAV	1.0575	0.0676	integrin, alpha V
276	APLP2	1.0567	0.0233	amyloid beta (A4) precursor-like protein 2
277	PDE7B	1.0558	0.0065	phosphodiesterase 7B
278	FMOD	1.055	0.0168	fibromodulin
279	PAPPA	1.0542	0.0166	pregnancy-associated plasma protein A, pappalysin 1
280	ARHGEF5	1.0542	0.0029	Rho guanine nucleotide exchange factor (GEF) 5
281	ANXA2P1	1.0542	0.0098	annexin A2 pseudogene 1
282	CALCRL	1.0533	0.0098	calcitonin receptor-like
283	SH2D4A	1.0508	0.002	SH2 domain containing 4A
284	ISLR	1.0508	0.0194	immunoglobulin superfamily containing leucine-rich repeat
285	STAT6	1.0492	0.0188	signal transducer and activator of transcription 6, interleukin-4 induced
286	GSN	1.0492	0.0337	gelsolin
287	ADCY6	1.0492	0.018	adenylate cyclase 6
288	TM4SF1	1.0483	0.0073	transmembrane 4 L six family member 1
289	CA13	1.0475	0.0003	carbonic anhydrase XIII
290	SSPN	1.0467	0.0213	sarcospan
291	S100A11	1.0458	0.0382	S100 calcium binding protein A11
292	PGAP3	1.0458	0.0137	post-GPI attachment to proteins 3
293	LGALS3	1.0458	0.0132	lectin, galactoside-binding, soluble, 3
294	IL6ST	1.0458	0.0331	interleukin 6 signal transducer (gp130, oncostatin M receptor)
295	RNF39	1.045	0.0245	ring finger protein 39
296	CAPN2	1.045	0.0209	calpain 2, (m/II) large subunit
297	ABHD15	1.045	0.0024	abhydrolase domain containing 15
298	C5orf23	1.0443	0.0027	chromosome 5 open reading frame 23
299	S100A4	1.0442	0.0152	S100 calcium binding protein A4
300	TSPAN12	1.0425	0.0035	tetraspanin 12
301	COL4A2	1.0408	0.0296	collagen, type IV, alpha 2
302	TOB1	1.04	0.0123	transducer of ERBB2, 1
303	IYD	1.04	0.0011	iodotyrosine deiodinase
304	SNX13	1.0392	0.0607	sorting nexin 13
305	SAV1	1.0367	0.0061	salvador homolog 1 (Drosophila)
306	MAOA	1.0367	0.0038	monoamine oxidase A
307	IRF6	1.0367	0.0027	interferon regulatory factor 6
308	IL15	1.0367	0.0121	interleukin 15
309	KCTD14	1.0357	0.0012	potassium channel tetramerisation domain containing 14
310	PGRMC2	1.035	0.0153	progesterone receptor membrane component 2
311	TRPS1	1.0342	0.0016	trichorhinophalangeal syndrome I
312	FLNB	1.0342	0.0064	filamin B, beta
313	TRMT2B	1.0333	0.001	tRNA methyltransferase 2 homolog B (S. cerevisiae)
314	TUSC1	1.0325	0.0004	tumor suppressor candidate 1
315	GNA14	1.0325	0.0045	guanine nucleotide binding protein (G protein), alpha 14
316	ANTXR1	1.0325	0.0183	anthrax toxin receptor 1
317	SNX12	1.0313	0.0021	sorting nexin 12
318	MYL9	1.0308	0.0244	myosin, light chain 9, regulatory
319	MANSC1	1.0308	0.0081	MANSC domain containing 1
320	EGFR	1.0308	0.0285	epidermal growth factor receptor
321	EHD2	1.03	0.0761	EH-domain containing 2
322	ITM2B	1.0283	0.0677	integral membrane protein 2B
323	CALCOCO1	1.0275	0.0696	calcium binding and coiled-coil domain 1
324	C4orf34	1.0275	0.0287	chromosome 4 open reading frame 34
325	SMAD3	1.0267	0.0053	SMAD family member 3
326	OMD	1.0267	0.0128	osteomodulin
327	EMP1	1.0267	0.0202	epithelial membrane protein 1
328	PROCR	1.0258	0.0032	protein C receptor, endothelial
329	MESDC2	1.025	0.0085	mesoderm development candidate 2
330	KIAA1462	1.0244	0.0092	KIAA1462
331	FLT1	1.0229	0.0027	fms-related tyrosine kinase 1 (vascular endothelial growth factor/vascular permeability factor receptor)
332	TNFSF15	1.0225	0.0015	tumor necrosis factor (ligand) superfamily, member 15
333	SLC5A3	1.0217	0.0007	solute carrier family 5 (sodium/myo-inositol cotransporter), member 3
334	UBAC2	1.0213	0.0038	UBA domain containing 2

335	TMEM64	1.0213	0.0031	transmembrane protein 64
336	ANXA2	1.0208	0.0258	annexin A2
337	PAPSS2	1.0192	0.0057	3'-phosphoadenosine 5'-phosphosulfate synthase 2
338	ASAP3	1.0175	0.0181	ArfGAP with SH3 domain, ankyrin repeat and PH domain 3
339	AJUBA	1.0175	0.0033	ajuba LIM protein
340	RBMS2	1.0167	0.0129	RNA binding motif, single stranded interacting protein 2
341	RBM47	1.0167	0.0102	RNA binding motif protein 47
342	GABRP	1.0167	0.0023	gamma-aminobutyric acid (GABA) A receptor, pi
343	DNAJC3	1.0167	0.0188	DnaJ (Hsp40) homolog, subfamily C, member 3
344	SFRP1	1.0142	0.0047	secreted frizzled-related protein 1
345	LRP5	1.0142	0.0062	low density lipoprotein receptor-related protein 5
346	LOXL1	1.0133	0.0154	lysyl oxidase-like 1
347	GSTK1	1.0133	0.0481	glutathione S-transferase kappa 1
348	BACE2	1.0117	0.0016	beta-site APP-cleaving enzyme 2
349	VSIG2	1.01	0.0025	V-set and immunoglobulin domain containing 2
350	VSIG10L	1.0087	0.0002	V-set and immunoglobulin domain containing 10 like
351	MXRA8	1.0083	0.0477	matrix-remodelling associated 8
352	CAPS2	1.0075	0.0009	calcyphosine 2
353	KCTD18	1.0063	0.0716	potassium channel tetramerisation domain containing 18
354	GRHL3	1.0063	0.0031	grainyhead-like 3 (Drosophila)
355	COL12A1	1.0063	0.0224	collagen, type XII, alpha 1
356	KRT14	1.0058	0.002	keratin 14
357	PLXDC2	1.005	0.0099	plexin domain containing 2
358	HGSNAT	1.005	0.0157	heparan-alpha-glucosaminide N-acetyltransferase
359	CYP39A1	1.0042	0.0013	cytochrome P450, family 39, subfamily A, polypeptide 1
360	RARRES1	1.0033	0.0039	retinoic acid receptor responder (tazarotene induced) 1
361	PLA2G4A	1.0033	0.0017	phospholipase A2, group IVA (cytosolic, calcium-dependent)
362	COL8A2	1.0033	0.0124	collagen, type VIII, alpha 2
363	BDKRB2	1.0025	0.0034	bradykinin receptor B2
364	SOSTDC1	1.0008	0.0015	sclerostin domain containing 1
365	FKBP10	1.0008	0.0124	FK506 binding protein 10, 65 kDa
366	CLIC3	1.0008	0.0021	chloride intracellular channel 3
367	PDIA3	1	0.0063	protein disulfide isomerase family A, member 3
368	SORBS2	0.9967	0.0063	sorbin and SH3 domain containing 2
369	PPP1R13L	0.9967	0.0158	protein phosphatase 1, regulatory subunit 13 like
370	NPHP3	0.9962	0.0565	nephronophthisis 3 (adolescent)
371	TBC1D8B	0.995	0.0165	TBC1 domain family, member 8B (with GRAM domain)
372	RSPO3	0.995	0.0034	R-spondin 3
373	CHMP4C	0.995	0.0024	charged multivesicular body protein 4C
374	BNIPL	0.9937	0.0018	BCL2/adenovirus E1B 19kD interacting protein like
375	SLC37A3	0.9925	0.0044	solute carrier family 37 (glycerol-3-phosphate transporter), member 3
376	PPARGC1A	0.9908	0.0014	peroxisome proliferator-activated receptor gamma, coactivator 1 alpha
377	ANXA1	0.9892	0.0335	annexin A1
378	ZBTB41	0.9887	0.106	zinc finger and BTB domain containing 41
379	TNFRSF19	0.9887	0.0008	tumor necrosis factor receptor superfamily, member 19
380	USP40	0.9875	0.0109	ubiquitin specific peptidase 40
381	PDZK1IP1	0.9875	0.0072	PDZK1 interacting protein 1
382	IL20RA	0.9875	0.0026	interleukin 20 receptor, alpha
383	THBS3	0.9858	0.033	thrombospondin 3
384	FZD6	0.9858	0.0128	frizzled family receptor 6
385	LOC339535	0.9857	0	uncharacterized LOC339535
386	KIAA1191	0.985	0.0224	KIAA1191
387	IGFBP7	0.985	0.0325	insulin-like growth factor binding protein 7
388	C19orf33	0.985	0.0036	chromosome 19 open reading frame 33
389	SLC26A4	0.9842	0.0002	solute carrier family 26, member 4
390	HERPUD2	0.9837	0.0381	HERPUD family member 2
391	CFLAR	0.9833	0.0298	CASP8 and FADD-like apoptosis regulator
392	RNLS	0.9817	0.0021	renalase, FAD-dependent amine oxidase
393	MAP3K1	0.9817	0.0097	mitogen-activated protein kinase kinase kinase 1, E3 ubiquitin protein ligase
394	SLC22A18	0.9808	0.0268	solute carrier family 22, member 18
395	CAV1	0.9808	0.0207	caveolin 1, caveolae protein, 22kDa
396	SEMA3C	0.9792	0.0152	sema domain, immunoglobulin domain (Ig), short basic domain, secreted, (semaphorin) 3C
397	KCNQ1	0.9792	0.0238	potassium voltage-gated channel, KQT-like subfamily, member 1
398	PCDHGA10	0.9782	0.0018	protocadherin gamma subfamily A, 10
399	LOC550643	0.9775	0.0123	uncharacterized LOC550643
400	WWTR1	0.9767	0.0089	WW domain containing transcription regulator 1
401	TSPAN6	0.9767	0.01	tetraspanin 6
402	PRRC1	0.9767	0.0171	proline-rich coiled-coil 1
403	C5orf46	0.9762	0.0016	chromosome 5 open reading frame 46
404	C11orf70	0.9762	0.0034	chromosome 11 open reading frame 70
405	P4HA2	0.9758	0.0203	prolyl 4-hydroxylase, alpha polypeptide II
406	KLF4	0.9758	0.0107	Kruppel-like factor 4 (gut)
407	F2R	0.9758	0.0161	coagulation factor II (thrombin) receptor
408	LAMA5	0.975	0.0124	laminin, alpha 5
409	DAB2	0.975	0.0425	disabled homolog 2, mitogen-responsive phosphoprotein (Drosophila)
410	TSTD1	0.9737	0.0083	thiosulfate sulfurtransferase (rhodanese)-like domain containing 1
411	TMCO4	0.9737	0.0079	transmembrane and coiled-coil domains 4
412	KIAA1522	0.9737	0.0155	KIAA1522
413	HSBP1L1	0.9737	0.0015	heat shock factor binding protein 1-like 1
414	TPM1	0.9733	0.0154	tropomyosin 1 (alpha)
415	TMEM43	0.9725	0.0267	transmembrane protein 43
416	SUMF2	0.9725	0.0068	sulfatase modifying factor 2
417	MLPH	0.9725	0.0069	melanophilin
418	KLF8	0.9725	0.0006	Kruppel-like factor 8

419	EXOC4	0.9725	0.0122	exocyst complex component 4
420	PLD1	0.9717	0.0089	phospholipase D1, phosphatidylcholine-specific
421	PPAP2B	0.9708	0.0139	phosphatidic acid phosphatase type 2B
422	TGFBI	0.97	0.0266	transforming growth factor, beta-induced, 68kDa
423	SLC38A6	0.97	0.0303	solute carrier family 38, member 6
424	S100A16	0.97	0.0182	S100 calcium binding protein A16
425	AKAP13	0.97	0.0458	A kinase (PRKA) anchor protein 13
426	SLC10A3	0.9692	0.019	solute carrier family 10 (sodium/bile acid cotransporter family), member 3
427	SDC1	0.9683	0.0051	syndecan 1
428	ADAM9	0.9675	0.0721	ADAM metallopeptidase domain 9
429	CTNNA1	0.9667	0.0504	catenin (cadherin-associated protein), alpha 1, 102kDa
430	NUDT16P1	0.9663	0.0006	nudix (nucleoside diphosphate linked moiety X)-type motif 16 pseudogene 1
431	FNDC3B	0.9658	0.0655	fibronectin type III domain containing 3B
432	CLIP1	0.9658	0.0672	CAP-GLY domain containing linker protein 1
433	ZNF750	0.9642	0.001	zinc finger protein 750
434	CLDN4	0.9642	0.0113	claudin 4
435	C1R	0.9642	0.0372	complement component 1, r subcomponent
436	THNSL2	0.9633	0.0024	threonine synthase-like 2 (S. cerevisiae)
437	MARC2	0.9633	0.0054	mitochondrial amidoxime reducing component 2
438	ITGB4	0.9633	0.0516	integrin, beta 4
439	CD59	0.9633	0.0386	CD59 molecule, complement regulatory protein
440	CD63	0.9625	0.0513	CD63 molecule
441	COL4A3	0.9617	0.0046	collagen, type IV, alpha 3 (Goodpasture antigen)
442	ATOH8	0.9613	0.0071	atonal homolog 8 (Drosophila)
443	SMAD5	0.9608	0.0346	SMAD family member 5
444	PTPRB	0.9608	0.0214	protein tyrosine phosphatase, receptor type, B
445	GLTP	0.9608	0.003	glycolipid transfer protein
446	CNN2	0.9608	0.0165	calponin 2
447	SBSPON	0.96	0.0006	somatomedin B and thrombospondin, type 1 domain containing
448	NFAT5	0.96	0.0464	nuclear factor of activated T-cells 5, tonicity-responsive
449	LGR4	0.96	0.0043	leucine-rich repeat containing G protein-coupled receptor 4
450	EPHA3	0.9592	0.0071	EPH receptor A3
451	DHRS3	0.9583	0.0074	dehydrogenase/reductase (SDR family) member 3
452	TSPAN8	0.9575	0.0021	tetraspanin 8
453	OTOS	0.9575	0.0038	otospiralin
454	IRS1	0.9567	0.0029	insulin receptor substrate 1
455	BGN	0.9567	0.0449	biglycan
456	GGT6	0.9563	0.0023	gamma-glutamyltransferase 6
457	NOTCH3	0.955	0.023	notch 3
458	EHF	0.955	0.0078	ets homologous factor
459	ALDH3B1	0.9542	0.0394	aldehyde dehydrogenase 3 family, member B1
460	SGPP2	0.9538	0.0012	sphingosine-1-phosphate phosphatase 2
461	NQO1	0.9533	0.0038	NAD(P)H dehydrogenase, quinone 1
462	MAP3K8	0.9533	0.0269	mitogen-activated protein kinase kinase kinase 8
463	LXN	0.9533	0.0081	latexin
464	NFKBIZ	0.9525	0.0236	nuclear factor of kappa light polypeptide gene enhancer in B-cells inhibitor, zeta
465	GNG11	0.9525	0.0205	guanine nucleotide binding protein (G protein), gamma 11
466	GPRC5A	0.9517	0.0167	G protein-coupled receptor, family C, group 5, member A
467	KIAA1377	0.9513	0.0199	KIAA1377
468	PHLDA1	0.9492	0.0117	pleckstrin homology-like domain, family A, member 1
469	SEMA4B	0.9488	0.0119	sema domain, immunoglobulin domain (Ig), transmembrane domain (TM) and short cytoplasmic domain, (semaphorin) 4B
470	TTC23	0.9483	0.0044	tetratricopeptide repeat domain 23
471	CAST	0.9475	0.0677	calpastatin
472	SERPINB5	0.9467	0.0057	serpin peptidase inhibitor, clade B (ovalbumin), member 5
473	ERMP1	0.9467	0.0124	endoplasmic reticulum metallopeptidase 1
474	MSRB3	0.9463	0.0332	methionine sulfoxide reductase B3
475	PLS3	0.9458	0.0317	plastin 3
476	FSTL1	0.9458	0.0448	follistatin-like 1
477	CDH11	0.9458	0.0326	cadherin 11, type 2, OB-cadherin (osteoblast)
478	SFRP4	0.945	0.0191	secreted frizzled-related protein 4
479	PALLD	0.945	0.0326	palladin, cytoskeletal associated protein
480	LY75	0.9442	0.0116	lymphocyte antigen 75
481	EPS8L1	0.9442	0.0408	EPS8-like 1
482	PXMP4	0.9433	0.0088	peroxisomal membrane protein 4, 24kDa
483	STEAP4	0.9425	0.0052	STEAP family member 4
484	METTL21B	0.9425	0.0016	methyltransferase like 21B
485	GNS	0.9417	0.0784	glucosamine (N-acetyl)-6-sulfatase
486	COL5A2	0.9417	0.0294	collagen, type V, alpha 2
487	TMEM87B	0.9413	0.0786	transmembrane protein 87B
488	ATL2	0.9413	0.063	atlastin GTPase 2
489	ATP13A4	0.94	0.0026	ATPase type 13A4
490	CROT	0.9392	0.012	carnitine O-octanoyltransferase
491	SASH1	0.9383	0.023	SAM and SH3 domain containing 1
492	NCSTN	0.9375	0.0285	nicastrin
493	CRYBG3	0.9375	0.0183	beta-gamma crystallin domain containing 3
494	ZNF323	0.9367	0.0019	zinc finger protein 323
495	TCN1	0.9367	0.0047	transcobalamin I (vitamin B12 binding protein, R binder family)
496	TACSTD2	0.9367	0.0078	tumor-associated calcium signal transducer 2
497	SNTB2	0.9367	0.0111	syntrophin, beta 2 (dystrophin-associated protein A1, 59kDa, basic component 2)
498	CD9	0.9367	0.0128	CD9 molecule
499	ARMCX2	0.9367	0.0094	armadillo repeat containing, X-linked 2
500	MGST1	0.9362	0.0061	microsomal glutathione S-transferase 1
501	UGDH	0.9342	0.0157	UDP-glucose 6-dehydrogenase
502	KLHDC1	0.9337	0.0593	kelch domain containing 1

503	FAT1	0.9333	0.0124	FAT tumor suppressor homolog 1 (Drosophila)
504	CTSK	0.9333	0.0346	cathepsin K
505	SDC2	0.9325	0.0185	syndecan 2
506	UGT1A8	0.9308	0.0019	UDP glucuronosyltransferase 1 family, polypeptide A8
507	TRPM4	0.9308	0.0147	transient receptor potential cation channel, subfamily M, member 4
508	LPP	0.9308	0.0337	LIM domain containing preferred translocation partner in lipoma
509	RBPMS	0.93	0.0126	RNA binding protein with multiple splicing
510	PHYHD1	0.93	0.0037	phytanoyl-CoA dioxygenase domain containing 1
511	PPL	0.9292	0.0121	periplakin
512	CLDN1	0.9292	0.0076	claudin 1
513	SBF2	0.9287	0.0634	SET binding factor 2
514	HCAR1	0.9287	0.0003	hydroxycarboxylic acid receptor 1
515	MAGT1	0.9283	0.0503	magnesium transporter 1
516	LAMP1	0.9283	0.044	lysosomal-associated membrane protein 1
517	FZD7	0.9267	0.0069	frizzled family receptor 7
518	DCTD	0.9258	0.0721	dCMP deaminase
519	SLCO2A1	0.925	0.0149	solute carrier organic anion transporter family, member 2A1
520	FCGRT	0.925	0.0551	Fc fragment of IgG, receptor, transporter, alpha
521	EPHX1	0.925	0.0245	epoxide hydrolase 1, microsomal (xenobiotic)
522	AQP1	0.925	0.0292	aquaporin 1 (Colton blood group)
523	NF1	0.9242	0.0381	neurofibromin 1
524	LMAN1	0.9242	0.0323	lectin, mannose-binding, 1
525	GIPC2	0.9242	0.002	GIPC PDZ domain containing family, member 2
526	IFFO2	0.9237	0.0031	intermediate filament family orphan 2
527	ESAM	0.9237	0.0114	endothelial cell adhesion molecule
528	PRSS23	0.9233	0.0202	protease, serine, 23
529	FLJ13197	0.9233	0.0171	uncharacterized FLJ13197
530	CRTAP	0.9233	0.0246	cartilage associated protein
531	NR4A1	0.9225	0.0127	nuclear receptor subfamily 4, group A, member 1
532	LPAR6	0.9225	0.0295	lysophosphatidic acid receptor 6
533	CTTNBP2NL	0.9225	0.0355	CTTNBP2 N-terminal like
534	MMP2	0.9217	0.0471	matrix metallopeptidase 2 (gelatinase A, 72kDa gelatinase, 72kDa type IV collagenase)
535	GAS6	0.9217	0.0419	growth arrest-specific 6
536	TNS3	0.92	0.0251	tensin 3
537	IL17RC	0.9192	0.0521	interleukin 17 receptor C
538	FRMD6	0.9187	0.0347	FERM domain containing 6
539	SLC12A2	0.9183	0.0117	solute carrier family 12 (sodium/potassium/chloride transporters), member 2
540	ITGB3	0.9183	0.026	integrin, beta 3 (platelet glycoprotein IIIa, antigen CD61)
541	TMPRSS11B	0.9175	0.0011	transmembrane protease, serine 11B
542	DUOX1	0.9175	0.0016	dual oxidase 1
543	ARHGAP24	0.9167	0.0185	Rho GTPase activating protein 24
544	KDELR2	0.9158	0.0599	KDEL (Lys-Asp-Glu-Leu) endoplasmic reticulum protein retention receptor 2
545	THBD	0.915	0.016	thrombomodulin
546	CMAHP	0.9142	0.0093	cytidine monophospho-N-acetylneuraminic acid hydroxylase, pseudogene
547	sept-10	0.9133	0.0252	septin 10
548	FERMT1	0.9133	0.0085	fermitin family member 1
549	ANO6	0.9125	0.0716	anoctamin 6
550	PAQR5	0.9117	0.0009	progestin and adipoQ receptor family member V
551	TRIP6	0.9108	0.0131	thyroid hormone receptor interactor 6
552	PTPN21	0.9108	0.0317	protein tyrosine phosphatase, non-receptor type 21
553	EPS8L2	0.9108	0.0137	EPS8-like 2
554	BTC	0.9108	0.0007	betacellulin
555	NT5E	0.91	0.0145	5'-nucleotidase, ecto (CD73)
556	NRP1	0.91	0.0284	neuropilin 1
557	GGT5	0.91	0.0487	gamma-glutamyltransferase 5
558	CALCA	0.91	0.1065	calcitonin-related polypeptide alpha
559	BNC2	0.9083	0.0316	basonuclin 2
560	SLC6A6	0.9075	0.0121	solute carrier family 6 (neurotransmitter transporter, taurine), member 6
561	FAM63A	0.9075	0.0269	family with sequence similarity 63, member A
562	CAPN12	0.9075	0.0021	calpain 12
563	SUCNR1	0.9063	0.0023	succinate receptor 1
564	ITGA2	0.9058	0.0155	integrin, alpha 2 (CD49B, alpha 2 subunit of VLA-2 receptor)
565	HOXA2	0.9058	0.0056	homeobox A2
566	BMPR2	0.9058	0.0788	bone morphogenetic protein receptor, type II (serine/threonine kinase)
567	LINC00473	0.9057	0.0007	long intergenic non-protein coding RNA 473
568	MMRN2	0.905	0.012	multimerin 2
569	COL1A1	0.905	0.0401	collagen, type I, alpha 1
570	KRTCAP2	0.9038	0.0366	keratinocyte associated protein 2
571	COL23A1	0.9038	0.0047	collagen, type XXIII, alpha 1
572	SP1	0.9017	0.0213	Sp1 transcription factor
573	DPY19L4	0.9017	0.1379	dpy-19-like 4 (C. elegans)
574	POF1B	0.9008	0.006	premature ovarian failure, 1B
575	FZD4	0.9008	0.031	frizzled family receptor 4
576	EMP2	0.9	0.0058	epithelial membrane protein 2
577	DSG3	0.9	0.0141	desmoglein 3
578	CXCL12	0.9	0.0294	chemokine (C-X-C motif) ligand 12
579	BMP4	0.9	0.0053	bone morphogenetic protein 4
580	BCAR3	0.9	0.0063	breast cancer anti-estrogen resistance 3
581	RAB10	0.8988	0.065	RAB10, member RAS oncogene family
582	FRMD4B	0.8983	0.0097	FERM domain containing 4B
583	CYP24A1	0.8975	0.0009	cytochrome P450, family 24, subfamily A, polypeptide 1
584	TMED3	0.8967	0.0435	transmembrane emp24 protein transport domain containing 3
585	RNPEP	0.8958	0.0535	arginyl aminopeptidase (aminopeptidase B)
586	PTPN9	0.8958	0.0081	protein tyrosine phosphatase, non-receptor type 9

587	PPFIBP1	0.895	0.0162	PTPRF interacting protein, binding protein 1 (liprin beta 1)
588	GCM2	0.895	0.0008	glial cells missing homolog 2 (Drosophila)
589	DSG2	0.895	0.0065	desmoglein 2
590	TGFB1I1	0.8942	0.0486	transforming growth factor beta 1 induced transcript 1
591	NIPAL2	0.8942	0.0362	NIPA-like domain containing 2
592	FIBIN	0.8938	0.0279	fin bud initiation factor homolog (zebrafish)
593	VWA8	0.8933	0.022	von Willebrand factor A domain containing 8
594	PLAT	0.8933	0.0163	plasminogen activator, tissue
595	FGL2	0.8933	0.0381	fibrinogen-like 2
596	ARFIP1	0.8933	0.1024	ADP-ribosylation factor interacting protein 1
597	GALNT4	0.8929	0.0098	UDP-N-acetyl-alpha-D-galactosamine:polypeptide N-acetylgalactosaminyltransferase 4 (GalNAc-T4)
598	C6orf132	0.8929	0.0116	chromosome 6 open reading frame 132
599	TSPAN4	0.8925	0.0443	tetraspanin 4
600	RNF144B	0.8925	0.0077	ring finger protein 144B
601	OSMR	0.8925	0.033	oncostatin M receptor
602	FAM219B	0.8925	0.0267	family with sequence similarity 219, member B
603	RNASEL	0.8917	0.0493	ribonuclease L (2',5'-oligoisoadenylate synthetase-dependent)
604	COL4A1	0.8917	0.0349	collagen, type IV, alpha 1
605	ATP2C2	0.8917	0.014	ATPase, Ca++ transporting, type 2C, member 2
606	ATP10D	0.8917	0.0708	ATPase, class V, type 10D
607	SLC44A1	0.8913	0.0173	solute carrier family 44, member 1
608	TFPI	0.8908	0.0226	tissue factor pathway inhibitor (lipoprotein-associated coagulation inhibitor)
609	AGR2	0.8908	0.0041	anterior gradient 2 homolog (Xenopus laevis)
610	ZDHHC5	0.89	0.0493	zinc finger, DHHC-type containing 5
611	FAM13A	0.89	0.012	family with sequence similarity 13, member A
612	TPM2	0.8892	0.0233	tropomyosin 2 (beta)
613	ASH1L	0.8892	0.0598	ash1 (absent, small, or homeotic)-like (Drosophila)
614	ANXA4	0.8892	0.0769	annexin A4
615	BOC	0.8888	0.0323	Boc homolog (mouse)
616	BTBD7	0.8883	0.0248	BTB (POZ) domain containing 7
617	ZNF75D	0.8867	0.0292	zinc finger protein 75D
618	SLFN5	0.8863	0.0245	schlafen family member 5
619	TCF7L2	0.8858	0.0098	transcription factor 7-like 2 (T-cell specific, HMG-box)
620	LAPTM4A	0.8858	0.0965	lysosomal protein transmembrane 4 alpha
621	ATP6V1B1	0.885	0.0019	ATPase, H+ transporting, lysosomal 56/58kDa, V1 subunit B1
622	MMP14	0.8842	0.0855	matrix metallopeptidase 14 (membrane-inserted)
623	SHISA2	0.8838	0.0006	shisa homolog 2 (Xenopus laevis)
624	PRDX5	0.8838	0.0519	peroxiredoxin 5
625	SERPING1	0.8825	0.0417	serpin peptidase inhibitor, clade G (C1 inhibitor), member 1
626	MAML2	0.8825	0.0173	mastermind-like 2 (Drosophila)
627	IL1R1	0.8825	0.0361	interleukin 1 receptor, type I
628	DSC3	0.8825	0.0062	desmocollin 3
629	TCN2	0.8817	0.0279	transcobalamin II
630	CTDSP1	0.8817	0.0653	CTD (carboxy-terminal domain, RNA polymerase II, polypeptide A) small phosphatase 1
631	HTRA3	0.8813	0.0259	HtrA serine peptidase 3
632	CD109	0.8813	0.0219	CD109 molecule
633	PBX1	0.88	0.0267	pre-B-cell leukemia homeobox 1
634	KDELR3	0.88	0.0311	KDEL (Lys-Asp-Glu-Leu) endoplasmic reticulum protein retention receptor 3
635	CEBPD	0.88	0.0277	CCAAT/enhancer binding protein (C/EBP), delta
636	ECE1	0.8792	0.0874	endothelin converting enzyme 1
637	CD55	0.8792	0.0347	CD55 molecule, decay accelerating factor for complement (Cromer blood group)
638	P4HA3	0.8788	0.0269	prolyl 4-hydroxylase, alpha polypeptide III
639	ATP6V0A4	0.8783	0.0021	ATPase, H+ transporting, lysosomal V0 subunit a4
640	ASS1	0.8783	0.0042	argininosuccinate synthase 1
641	TSPAN1	0.8775	0.025	tetraspanin 1
642	TGFBR2	0.8775	0.0559	transforming growth factor, beta receptor II (70/80kDa)
643	SRXN1	0.8775	0.0204	sulfiredoxin 1
644	SH3BGRL2	0.8775	0.0182	SH3 domain binding glutamic acid-rich protein like 2
645	GPR108	0.8775	0.0762	G protein-coupled receptor 108
646	CITED2	0.8775	0.0105	Cbp/p300-interacting transactivator, with Glu/Asp-rich carboxy-terminal domain, 2
647	ABCC3	0.8775	0.02	ATP-binding cassette, sub-family C (CFTR/MRP), member 3
648	ENG	0.8767	0.0621	endoglin
649	FAM162B	0.8763	0.0087	family with sequence similarity 162, member B
650	BBS2	0.8763	0.099	Bardet-Biedl syndrome 2
651	RECK	0.8758	0.0781	reversion-inducing-cysteine-rich protein with kazal motifs
652	MRGPRF	0.8757	0.0272	MAS-related GPR, member F
653	ANXA2P2	0.8755	0.0386	annexin A2 pseudogene 2
654	LRRC32	0.8742	0.0566	leucine rich repeat containing 32
655	CLDN3	0.8742	0.0279	claudin 3
656	ASPN	0.8742	0.0132	asporin
657	PLEKHH2	0.8737	0.0242	pleckstrin homology domain containing, family H (with MyTH4 domain) member 2
658	C4orf3	0.8737	0.0604	chromosome 4 open reading frame 3
659	SERPINH1	0.8733	0.0376	serpin peptidase inhibitor, clade H (heat shock protein 47), member 1, (collagen binding protein 1)
660	ITGA3	0.8725	0.0361	integrin, alpha 3 (antigen CD49C, alpha 3 subunit of VLA-3 receptor)
661	IL22RA1	0.8725	0.0079	interleukin 22 receptor, alpha 1
662	SVIL	0.8717	0.018	supervillin
663	IGF1R	0.8717	0.0143	insulin-like growth factor 1 receptor
664	EPHA2	0.8717	0.0241	EPH receptor A2
665	C6orf89	0.8712	0.0515	chromosome 6 open reading frame 89
666	MTMR11	0.8708	0.0146	myotubularin related protein 11
667	LRP2	0.8708	0.0169	low density lipoprotein receptor-related protein 2
668	TMC4	0.87	0.0198	transmembrane channel-like 4
669	TFAP2A	0.87	0.004	transcription factor AP-2 alpha (activating enhancer binding protein 2 alpha)
670	SMR3B	0.87	0.0348	submaxillary gland androgen regulated protein 3B

671	LPHN2	0.87	0.0096	latrophilin 2
672	EXT2	0.8683	0.0361	exostosin 2
673	EMCN	0.8683	0.0276	endomucin
674	CRABP2	0.8683	0.0071	cellular retinoic acid binding protein 2
675	UBR1	0.8675	0.1461	ubiquitin protein ligase E3 component n-recognin 1
676	SULF1	0.8675	0.0383	sulfatase 1
677	PPARA	0.8667	0.0164	peroxisome proliferator-activated receptor alpha
678	PDGFRL	0.8658	0.0108	platelet-derived growth factor receptor-like
679	AKR1B10	0.8658	0.0028	aldo-keto reductase family 1, member B10 (aldose reductase)
680	TMEM106B	0.865	0.1597	transmembrane protein 106B
681	THBS1	0.865	0.0425	thrombospondin 1
682	SERINC2	0.865	0.0358	serine incorporator 2
683	LIPG	0.865	0.0029	lipase, endothelial
684	LIMK2	0.865	0.0073	LIM domain kinase 2
685	BMPR1A	0.865	0.0208	bone morphogenetic protein receptor, type IA
686	DUSP1	0.8642	0.039	dual specificity phosphatase 1
687	FAM211B	0.8637	0.03	family with sequence similarity 211, member B
688	MBTPS1	0.8633	0.0386	membrane-bound transcription factor peptidase, site 1
689	TPO	0.8625	0.0117	thyroid peroxidase
690	DPP7	0.8625	0.066	dipeptidyl-peptidase 7
691	TOM1L1	0.8617	0.0149	target of myb1 (chicken)-like 1
692	LRRC57	0.8612	0.0275	leucine rich repeat containing 57
693	ANGPTL1	0.8612	0.0142	angiopoietin-like 1
694	SDPR	0.8608	0.0113	serum deprivation response
695	RNF180	0.86	0.0134	ring finger protein 180
696	EPAS1	0.86	0.0463	endothelial PAS domain protein 1
697	ACCS	0.86	0.0219	1-aminocyclopropane-1-carboxylate synthase homolog (Arabidopsis)(non-functional)
698	TIMP3	0.8583	0.0438	TIMP metallopeptidase inhibitor 3
699	SH3YL1	0.8583	0.0474	SH3 domain containing, Ysc84-like 1 (S. cerevisiae)
700	CRYAB	0.8583	0.0298	crystallin, alpha B
701	TRIM29	0.8575	0.0321	tripartite motif containing 29
702	CYP27B1	0.8575	0.0072	cytochrome P450, family 27, subfamily B, polypeptide 1
703	CREG1	0.8575	0.0445	cellular repressor of E1A-stimulated genes 1
704	SCPEP1	0.8567	0.0428	serine carboxypeptidase 1
705	DEFB1	0.8567	0.0035	defensin, beta 1
706	SURF4	0.8562	0.0731	surfeit 4
707	ERLIN2	0.8558	0.0396	ER lipid raft associated 2
708	SEMA3F	0.855	0.0359	sema domain, immunoglobulin domain (Ig), short basic domain, secreted, (semaphorin) 3F
709	C10orf116	0.855	0.0146	chromosome 10 open reading frame 116
710	RRBP1	0.8542	0.0491	ribosome binding protein 1 homolog 180kDa (dog)
711	CTDSPL	0.8542	0.0138	CTD (carboxy-terminal domain, RNA polymerase II, polypeptide A) small phosphatase-like
712	PCDH18	0.8537	0.0362	protocadherin 18
713	SPG11	0.8533	0.1128	spastic paraplegia 11 (autosomal recessive)
714	IGFBP5	0.8533	0.0451	insulin-like growth factor binding protein 5
715	FGD6	0.8533	0.0143	FYVE, RhoGEF and PH domain containing 6
716	VWA5A	0.8517	0.0156	von Willebrand factor A domain containing 5A
717	RIPK3	0.8512	0.0225	receptor-interacting serine-threonine kinase 3
718	SLC5A1	0.85	0.0203	solute carrier family 5 (sodium/glucose cotransporter), member 1
719	MIR143HG	0.85	0.0584	MIR143 host gene (non-protein coding)
720	LEPREL4	0.85	0.0171	leprecan-like 4
721	DNAJB4	0.85	0.0699	DnaJ (Hsp40) homolog, subfamily B, member 4
722	ITGB8	0.8492	0.0139	integrin, beta 8
723	HECTD1	0.8487	0.0715	HECT domain containing E3 ubiquitin protein ligase 1
724	TLR3	0.8483	0.0226	toll-like receptor 3
725	HBP1	0.8483	0.1127	HMG-box transcription factor 1
726	TXNDC15	0.8475	0.0703	thioredoxin domain containing 15
727	TMEM179B	0.8475	0.0618	transmembrane protein 179B
728	SLC35B2	0.8475	0.0419	solute carrier family 35, member B2
729	DAPK2	0.8475	0.0473	death-associated protein kinase 2
730	TNS4	0.8458	0.0697	tensin 4
731	MID2	0.8458	0.0377	midline 2
732	SCEL	0.845	0.0066	sciellin
733	OSBPL5	0.845	0.0347	oxysterol binding protein-like 5
734	NUPR1	0.845	0.0262	nuclear protein, transcriptional regulator, 1
735	HHAT	0.845	0.0069	hedgehog acyltransferase
736	CALD1	0.845	0.0615	caldesmon 1
737	HRH1	0.8442	0.0326	histamine receptor H1
738	JAG1	0.8433	0.0174	jagged 1
739	CA6	0.8433	0.0087	carbonic anhydrase VI
740	TXNDC11	0.8425	0.0249	thioredoxin domain containing 11
741	PTGFRN	0.8425	0.0108	prostaglandin F2 receptor negative regulator
742	PRR15L	0.8425	0.0183	proline rich 15-like
743	PAM	0.8425	0.087	peptidylglycine alpha-amidating monooxygenase
744	DSTN	0.8425	0.0931	destrin (actin depolymerizing factor)
745	ADCY9	0.8417	0.0163	adenylate cyclase 9
746	TC2N	0.8413	0.0176	tandem C2 domains, nuclear
747	PARD6G	0.8413	0.0084	par-6 partitioning defective 6 homolog gamma (C. elegans)
748	CYYR1	0.8413	0.0173	cysteine/tyrosine-rich 1
749	PRRX1	0.8408	0.036	paired related homeobox 1
750	EVPL	0.8408	0.0557	envoplakin
751	TIMP1	0.84	0.0763	TIMP metallopeptidase inhibitor 1
752	OS9	0.84	0.0899	osteosarcoma amplified 9, endoplasmic reticulum lectin
753	C9orf3	0.8392	0.0053	chromosome 9 open reading frame 3
754	TOR1AIP1	0.8383	0.1407	torsin A interacting protein 1

755	APP	0.8383	0.0303	amyloid beta (A4) precursor protein
756	TFF3	0.8375	0.0036	trefoil factor 3 (intestinal)
757	NYNRIN	0.8375	0.0184	NYN domain and retroviral integrase containing
758	NUMB	0.8375	0.0756	numb homolog (Drosophila)
759	ISM1	0.8375	0.0026	isthmin 1 homolog (zebrafish)
760	DNMBP	0.8375	0.0053	dynamin binding protein
761	RBM43	0.8363	0.0305	RNA binding motif protein 43
762	IMMP2L	0.8363	0.015	IMP2 inner mitochondrial membrane peptidase-like (S. cerevisiae)
763	SLPI	0.8358	0.0216	secretory leukocyte peptidase inhibitor
764	TMEM248	0.835	0.0719	transmembrane protein 248
765	LRP1	0.8342	0.1156	low density lipoprotein receptor-related protein 1
766	ARHGAP18	0.8338	0.0425	Rho GTPase activating protein 18
767	ANXA9	0.8333	0.0167	annexin A9
768	TMTC3	0.8325	0.1214	transmembrane and tetratricopeptide repeat containing 3
769	SLIT3	0.8325	0.059	slit homolog 3 (Drosophila)
770	MON2	0.8325	0.1181	MON2 homolog (S. cerevisiae)
771	ENPP1	0.8325	0.0123	ectonucleotide pyrophosphatase/phosphodiesterase 1
772	ANXA3	0.8325	0.0152	annexin A3
773	WBP1L	0.8317	0.0641	WW domain binding protein 1-like
774	FUT2	0.8317	0.0268	fucosyltransferase 2 (secretor status included)
775	RAB20	0.8308	0.016	RAB20, member RAS oncogene family
776	PROS1	0.8308	0.04	protein S (alpha)
777	TMBIM4	0.83	0.084	transmembrane BAX inhibitor motif containing 4
778	ANO10	0.83	0.02	anoctamin 10
779	MPZL2	0.8292	0.0178	myelin protein zero-like 2
780	ELTD1	0.8292	0.0328	EGF, latrophilin and seven transmembrane domain containing 1
781	CCDC170	0.8292	0.0138	coiled-coil domain containing 170
782	VIMP	0.8288	0.0613	VCP-interacting membrane protein
783	TM2D2	0.8288	0.0727	TM2 domain containing 2
784	ZNF185	0.8283	0.0066	zinc finger protein 185 (LIM domain)
785	GRHL2	0.8283	0.0132	grainyhead-like 2 (Drosophila)
786	SOD3	0.8275	0.081	superoxide dismutase 3, extracellular
787	ODAM	0.8275	0.0019	odontogenic, ameloblast asssociated
788	SERF2	0.8273	0.0323	small EDRK-rich factor 2
789	CG030	0.8273	0.0416	uncharacterized CG030
790	FREM2	0.8271	0.0064	FRAS1 related extracellular matrix protein 2
791	LEPR	0.8267	0.048	leptin receptor
792	SPRYD3	0.8263	0.0398	SPRY domain containing 3
793	SLAIN2	0.8263	0.1022	SLAIN motif family, member 2
794	SCNN1G	0.8263	0.001	sodium channel, non-voltage-gated 1, gamma subunit
795	RASEF	0.8263	0.0045	RAS and EF-hand domain containing
796	KDELR1	0.8258	0.076	KDEL (Lys-Asp-Glu-Leu) endoplasmic reticulum protein retention receptor 1
797	INADL	0.8258	0.0245	InaD-like (Drosophila)
798	TEAD1	0.825	0.0294	TEA domain family member 1 (SV40 transcriptional enhancer factor)
799	RASSF9	0.825	0.0106	Ras association (RalGDS/AF-6) domain family (N-terminal) member 9
800	C5orf4	0.825	0.0799	chromosome 5 open reading frame 4
801	BCKDHB	0.825	0.0404	branched chain keto acid dehydrogenase E1, beta polypeptide
802	ALDH1L2	0.825	0.0089	aldehyde dehydrogenase 1 family, member L2
803	ZNF770	0.8225	0.0923	zinc finger protein 770
804	NHS	0.8225	0.0045	Nance-Horan syndrome (congenital cataracts and dental anomalies)
805	LZTS2	0.8225	0.0456	leucine zipper, putative tumor suppressor 2
806	EFNA1	0.8225	0.0129	ephrin-A1
807	ZFP36	0.8217	0.0617	zinc finger protein 36, C3H type, homolog (mouse)
808	TBC1D4	0.8217	0.0086	TBC1 domain family, member 4
809	PTPN13	0.8217	0.0209	protein tyrosine phosphatase, non-receptor type 13 (APO-1/CD95 (Fas)-associated phosphatase)
810	ENPEP	0.8217	0.0057	glutamyl aminopeptidase (aminopeptidase A)
811	DSP	0.8217	0.012	desmoplakin
812	TM9SF2	0.8208	0.1461	transmembrane 9 superfamily member 2
813	GALNT3	0.8208	0.0113	UDP-N-acetyl-alpha-D-galactosamine:polypeptide N-acetylgalactosaminyltransferase 3 (GalNAc-T3)
814	PTGER3	0.82	0.045	prostaglandin E receptor 3 (subtype EP3)
815	PPP1R3C	0.82	0.0232	protein phosphatase 1, regulatory subunit 3C
816	HTRA1	0.82	0.0648	HtrA serine peptidase 1
817	PCBP2	0.8192	0.0176	poly(rC) binding protein 2
818	PP7080	0.8188	0.0115	uncharacterized LOC25845
819	SNED1	0.8183	0.0419	sushi, nidogen and EGF-like domains 1
820	KIAA0494	0.8175	0.0979	KIAA0494
821	TRADD	0.8167	0.0664	TNFRSF1A-associated via death domain
822	TRPM7	0.8163	0.0711	transient receptor potential cation channel, subfamily M, member 7
823	ABCA9	0.8163	0.0156	ATP-binding cassette, sub-family A (ABC1), member 9
824	KLF3	0.8158	0.0422	Kruppel-like factor 3 (basic)
825	COL5A1	0.8158	0.0612	collagen, type V, alpha 1
826	S100A10	0.815	0.0399	S100 calcium binding protein A10
827	PIK3CB	0.815	0.0177	phosphatidylinositol-4,5-bisphosphate 3-kinase, catalytic subunit beta
828	C11orf92	0.815	0.0129	chromosome 11 open reading frame 92
829	PRSS8	0.8142	0.036	protease, serine, 8
830	NOX4	0.8142	0.0066	NADPH oxidase 4
831	LURAP1L	0.8138	0.0148	leucine rich adaptor protein 1-like
832	FAM109B	0.8138	0.0082	family with sequence similarity 109, member B
833	CMTM4	0.8138	0.0138	CKLF-like MARVEL transmembrane domain containing 4
834	TSPO	0.8133	0.0815	translocator protein (18kDa)
835	GALNT10	0.8133	0.0162	UDP-N-acetyl-alpha-D-galactosamine:polypeptide N-acetylgalactosaminyltransferase 10 (GalNAc-T10)
836	FBLN2	0.8133	0.0399	fibulin 2
837	P4HB	0.8125	0.1021	prolyl 4-hydroxylase, beta polypeptide
838	ITGA11	0.8125	0.0778	integrin, alpha 11

839	SERPINB13	0.8117	0.0416	serpin peptidase inhibitor, clade B (ovalbumin), member 13
840	GJA1	0.8117	0.0163	gap junction protein, alpha 1, 43kDa
841	DSC1	0.8117	0.0048	desmocollin 1
842	TCTA	0.8108	0.0426	T-cell leukemia translocation altered
843	RETSAT	0.8108	0.0445	retinol saturase (all-trans-retinol 13,14-reductase)
844	RBMS1	0.8108	0.0608	RNA binding motif, single stranded interacting protein 1
845	TMEM219	0.81	0.0918	transmembrane protein 219
846	MORC4	0.8092	0.0081	MORC family CW-type zinc finger 4
847	LTBP3	0.8092	0.0722	latent transforming growth factor beta binding protein 3
848	ITGB6	0.8092	0.0096	integrin, beta 6
849	DENND4C	0.8092	0.1384	DENN/MADD domain containing 4C
850	KRT80	0.8087	0.0076	keratin 80
851	TMED2	0.8075	0.1206	transmembrane emp24 domain trafficking protein 2
852	SPATS2L	0.8075	0.0303	spermatogenesis associated, serine-rich 2-like
853	CHP1	0.8075	0.0348	calcineurin-like EF hand protein 1
854	BARX2	0.8067	0.0306	BARX homeobox 2
855	FAM174A	0.8062	0.071	family with sequence similarity 174, member A
856	AP1S3	0.8062	0.0107	adaptor-related protein complex 1, sigma 3 subunit
857	AGPAT9	0.8062	0.0074	1-acylglycerol-3-phosphate O-acyltransferase 9
858	TBL1Y	0.8058	0.0023	transducin (beta)-like 1, Y-linked
859	ZBTB4	0.805	0.096	zinc finger and BTB domain containing 4
860	FBLN1	0.805	0.0725	fibulin 1
861	ALS2CL	0.805	0.1043	ALS2 C-terminal like
862	PBXIP1	0.8042	0.092	pre-B-cell leukemia homeobox interacting protein 1
863	AFF4	0.8042	0.0766	AF4/FMR2 family, member 4
864	PAWR	0.8033	0.0118	PRKC, apoptosis, WT1, regulator
865	VSTM4	0.8025	0.0701	V-set and transmembrane domain containing 4
866	COL15A1	0.8025	0.0273	collagen, type XV, alpha 1
867	LTBR	0.8017	0.0765	lymphotoxin beta receptor (TNFR superfamily, member 3)
868	CSRP1	0.8008	0.0537	cysteine and glycine-rich protein 1
869	AQP3	0.8	0.0068	aquaporin 3 (Gill blood group)
870	AP1M2	0.8	0.0606	adaptor-related protein complex 1, mu 2 subunit
871	REEP5	0.7992	0.096	receptor accessory protein 5
872	PAFAH2	0.7992	0.0137	platelet-activating factor acetylhydrolase 2, 40kDa
873	MPP5	0.7992	0.071	membrane protein, palmitoylated 5 (MAGUK p55 subfamily member 5)
874	HEBP2	0.7992	0.0323	heme binding protein 2
875	SPATA18	0.7987	0.0144	spermatogenesis associated 18
876	TNFRSF1A	0.7983	0.0756	tumor necrosis factor receptor superfamily, member 1A
877	TGOLN2	0.7983	0.0672	trans-golgi network protein 2
878	LAMA4	0.7983	0.0606	laminin, alpha 4
879	CCDC102B	0.7983	0.0088	coiled-coil domain containing 102B
880	ALDH3B2	0.7983	0.0163	aldehyde dehydrogenase 3 family, member B2
881	ABCA8	0.7983	0.0333	ATP-binding cassette, sub-family A (ABC1), member 8
882	TM4SF18	0.7975	0.012	transmembrane 4 L six family member 18
883	PXDC1	0.7975	0.0472	PX domain containing 1
884	NOL3	0.7975	0.0301	nucleolar protein 3 (apoptosis repressor with CARD domain)
885	HM13	0.7975	0.0583	histocompatibility (minor) 13
886	PRR5L	0.7967	0.0066	proline rich 5 like
887	GEM	0.7967	0.0399	GTP binding protein overexpressed in skeletal muscle
888	CD46	0.7967	0.1088	CD46 molecule, complement regulatory protein
889	SRPX2	0.7958	0.0296	sushi-repeat containing protein, X-linked 2
890	CLCF1	0.7958	0.071	cardiotrophin-like cytokine factor 1
891	MIA	0.7957	0.0076	melanoma inhibitory activity
892	SUSD5	0.795	0.0027	sushi domain containing 5
893	POU2F3	0.795	0.0037	POU class 2 homeobox 3
894	OLFML2B	0.795	0.037	olfactomedin-like 2B
895	AP3B1	0.795	0.0448	adaptor-related protein complex 3, beta 1 subunit
896	PLCE1	0.7942	0.0139	phospholipase C, epsilon 1
897	C1GALT1C1	0.7942	0.1059	C1GALT1-specific chaperone 1
898	ACSF2	0.7942	0.0408	acyl-CoA synthetase family member 2
899	MIR31HG	0.7937	0.0131	MIR31 host gene (non-protein coding)
900	SLC2A10	0.7933	0.0278	solute carrier family 2 (facilitated glucose transporter), member 10
901	PPIB	0.7933	0.0765	peptidylprolyl isomerase B (cyclophilin B)
902	DTWD1	0.7933	0.0893	DTW domain containing 1
903	TMEM129	0.7925	0.0517	transmembrane protein 129
904	NUAK2	0.7925	0.0074	NUAK family, SNF1-like kinase, 2
905	LCN12	0.7925	0.0009	lipocalin 12
906	HOOK3	0.7925	0.1417	hook homolog 3 (Drosophila)
907	SOCS5	0.7917	0.1438	suppressor of cytokine signaling 5
908	HTN1	0.7917	0.0058	histatin 1
909	HEXB	0.7917	0.0963	hexosaminidase B (beta polypeptide)
910	AMIGO2	0.7917	0.0137	adhesion molecule with Ig-like domain 2
911	PAPLN	0.7912	0.0136	papilin, proteoglycan-like sulfated glycoprotein
912	IGIP	0.7912	0.0844	IgA-inducing protein homolog (Bos taurus)
913	TSHR	0.7908	0.0096	thyroid stimulating hormone receptor
914	RNF128	0.7908	0.0053	ring finger protein 128, E3 ubiquitin protein ligase
915	COL6A3	0.7908	0.0401	collagen, type VI, alpha 3
916	TCF7L1	0.79	0.0259	transcription factor 7-like 1 (T-cell specific, HMG-box)
917	MATN2	0.79	0.0207	matrilin 2
918	POLD4	0.7892	0.099	polymerase (DNA-directed), delta 4, accessory subunit
919	MFGE8	0.7892	0.0398	milk fat globule-EGF factor 8 protein
920	FMO2	0.7892	0.0434	flavin containing monooxygenase 2 (non-functional)
921	DOCK5	0.7892	0.0327	dedicator of cytokinesis 5
922	MYADM	0.7887	0.0522	myeloid-associated differentiation marker

923	EOGT	0.7875	0.1071	EGF domain-specific O-linked N-acetylglucosamine (GlcNAc) transferase
924	CARD6	0.7875	0.0528	caspase recruitment domain family, member 6
925	PDCD4	0.7867	0.084	programmed cell death 4 (neoplastic transformation inhibitor)
926	OLFML1	0.7867	0.0506	olfactomedin-like 1
927	LRRC2	0.7867	0.003	leucine rich repeat containing 2
928	FLRT2	0.7867	0.0395	fibronectin leucine rich transmembrane protein 2
929	CST4	0.7864	0.0187	cystatin S
930	ZNF514	0.7862	0.0636	zinc finger protein 514
931	TMEM171	0.7862	0.0021	transmembrane protein 171
932	DMKN	0.7862	0.0059	dermokine
933	PLVAP	0.7858	0.041	plasmalemma vesicle associated protein
934	CLMP	0.785	0.019	CXADR-like membrane protein
935	ZBTB20	0.7842	0.0893	zinc finger and BTB domain containing 20
936	CCDC68	0.7842	0.0136	coiled-coil domain containing 68
937	ZNF436	0.7837	0.0403	zinc finger protein 436
938	CCDC6	0.7833	0.0573	coiled-coil domain containing 6
939	ALDH9A1	0.7833	0.0456	aldehyde dehydrogenase 9 family, member A1
940	EMB	0.7829	0.013	embigin
941	S100A14	0.7825	0.0191	S100 calcium binding protein A14
942	VANGL1	0.7817	0.0201	vang-like 1 (van gogh, Drosophila)
943	RAB25	0.7817	0.0215	RAB25, member RAS oncogene family
944	FPGT	0.7817	0.1744	fucose-1-phosphate guanylyltransferase
945	CRIP1	0.7817	0.0186	cysteine-rich protein 1 (intestinal)
946	CNGA1	0.7817	0.03	cyclic nucleotide gated channel alpha 1
947	ACOX2	0.7817	0.0101	acyl-CoA oxidase 2, branched chain
948	TPCN1	0.7808	0.0491	two pore segment channel 1
949	IGFBP3	0.7808	0.0224	insulin-like growth factor binding protein 3
950	CD34	0.7808	0.0447	CD34 molecule
951	SNX9	0.78	0.0437	sorting nexin 9
952	SCARB2	0.78	0.1184	scavenger receptor class B, member 2
953	CYP3A5	0.78	0.0194	cytochrome P450, family 3, subfamily A, polypeptide 5
954	COG6	0.78	0.1503	component of oligomeric golgi complex 6
955	PRELP	0.7792	0.1187	proline/arginine-rich end leucine-rich repeat protein
956	PKD2	0.7792	0.1148	polycystic kidney disease 2 (autosomal dominant)
957	KIAA1456	0.7792	0.0302	KIAA1456
958	RAPGEF3	0.7783	0.1437	Rap guanine nucleotide exchange factor (GEF) 3
959	EPHB3	0.7783	0.0479	EPH receptor B3
960	GJB6	0.7775	0.002	gap junction protein, beta 6, 30kDa
961	TMED10	0.7767	0.143	transmembrane emp24-like trafficking protein 10 (yeast)
962	CPED1	0.7767	0.0331	cadherin-like and PC-esterase domain containing 1
963	AZGP1	0.7767	0.0068	alpha-2-glycoprotein 1, zinc-binding
964	AR	0.7767	0.0095	androgen receptor
965	PAR5	0.7758	0.0208	Prader-Willi/Angelman syndrome-5
966	MVP	0.7758	0.1181	major vault protein
967	DYNC2H1	0.7758	0.04	dynein, cytoplasmic 2, heavy chain 1
968	ATF7	0.7758	0.0301	activating transcription factor 7
969	MYO5C	0.775	0.0223	myosin VC
970	KIAA1033	0.775	0.1645	KIAA1033
971	ARHGEF40	0.775	0.0344	Rho guanine nucleotide exchange factor (GEF) 40
972	SLC4A7	0.7742	0.0451	solute carrier family 4, sodium bicarbonate cotransporter, member 7
973	FAM20A	0.7738	0.0223	family with sequence similarity 20, member A
974	FAM199X	0.7738	0.102	family with sequence similarity 199, X-linked
975	MYH9	0.7733	0.1047	myosin, heavy chain 9, non-muscle
976	FAM46A	0.7733	0.0204	family with sequence similarity 46, member A
977	FLJ35776	0.7729	0.0085	uncharacterized LOC649446
978	SPRY1	0.7725	0.0215	sprouty homolog 1, antagonist of FGF signaling (Drosophila)
979	MPZL1	0.7725	0.0304	myelin protein zero-like 1
980	EVC	0.7725	0.0901	Ellis van Creveld syndrome
981	CDH13	0.7725	0.0227	cadherin 13, H-cadherin (heart)
982	PEAK1	0.7717	0.0547	NKF3 kinase family member
983	IL13RA1	0.7717	0.0949	interleukin 13 receptor, alpha 1
984	AKR1C3	0.7717	0.0121	aldo-keto reductase family 1, member C3 (3-alpha hydroxysteroid dehydrogenase, type II)
985	TMEM218	0.77	0.0564	transmembrane protein 218
986	SLC25A29	0.77	0.0463	solute carrier family 25 (mitochondrial carnitine/acylcarnitine carrier), member 29
987	POSTN	0.77	0.0326	periostin, osteoblast specific factor
988	PLOD2	0.77	0.039	procollagen-lysine, 2-oxoglutarate 5-dioxygenase 2
989	ACSS3	0.77	0.0128	acyl-CoA synthetase short-chain family member 3
990	TMEM30B	0.7692	0.0171	transmembrane protein 30B
991	DUOX2	0.7692	0.0187	dual oxidase 2
992	ADAM15	0.7692	0.0892	ADAM metallopeptidase domain 15
993	SHE	0.7688	0.03	Src homology 2 domain containing E
994	NR2F2	0.7683	0.0253	nuclear receptor subfamily 2, group F, member 2
995	KDR	0.7683	0.0176	kinase insert domain receptor (a type III receptor tyrosine kinase)
996	EIF4EBP2	0.7683	0.0189	eukaryotic translation initiation factor 4E binding protein 2
997	CREB3L2	0.7683	0.0331	cAMP responsive element binding protein 3-like 2
998	B2M	0.7683	0.105	beta-2-microglobulin
999	OLFML3	0.7675	0.0534	olfactomedin-like 3
1000	LMO7	0.7675	0.0167	LIM domain 7
1001	KRT19	0.7675	0.0099	keratin 19
1002	FAM26E	0.7675	0.0325	family with sequence similarity 26, member E
1003	GALNT11	0.7667	0.0409	UDP-N-acetyl-alpha-D-galactosamine:polypeptide N-acetylgalactosaminyltransferase 11 (GalNAc-T11)
1004	FAM46B	0.7663	0.0109	family with sequence similarity 46, member B
1005	MCC	0.7658	0.0284	mutated in colorectal cancers
1006	F2RL2	0.7658	0.0201	coagulation factor II (thrombin) receptor-like 2

1007	AMOTL2	0.7658	0.0247	angiomotin like 2
1008	RGL3	0.765	0.0144	ral guanine nucleotide dissociation stimulator-like 3
1009	CRIPAK	0.765	0.0551	cysteine-rich PAK1 inhibitor
1010	NOTCH2	0.7642	0.0548	notch 2
1011	SLC1A1	0.7633	0.007	solute carrier family 1 (neuronal/epithelial high affinity glutamate transporter, system Xag), member 1
1012	EFNA4	0.7633	0.0199	ephrin-A4
1013	TMTC2	0.7625	0.0157	transmembrane and tetratricopeptide repeat containing 2
1014	SPSB2	0.7625	0.0096	splA/ryanodine receptor domain and SOCS box containing 2
1015	SLC39A9	0.7625	0.0535	solute carrier family 39 (zinc transporter), member 9
1016	SLC35A3	0.7625	0.1538	solute carrier family 35 (UDP-N-acetylglucosamine (UDP-GlcNAc) transporter), member A3
1017	PPP1R3D	0.7625	0.0064	protein phosphatase 1, regulatory subunit 3D
1018	NDNL2	0.7625	0.0116	necdin-like 2
1019	NAB1	0.7625	0.0791	NGFI-A binding protein 1 (EGR1 binding protein 1)
1020	FABP4	0.7625	0.0077	fatty acid binding protein 4, adipocyte
1021	DUSP14	0.7625	0.0205	dual specificity phosphatase 14
1022	GATAD1	0.7617	0.0508	GATA zinc finger domain containing 1
1023	YIPF5	0.7608	0.1667	Yip1 domain family, member 5
1024	NFE2L2	0.7608	0.1352	nuclear factor (erythroid-derived 2)-like 2
1025	MAN2B2	0.76	0.0845	mannosidase, alpha, class 2B, member 2
1026	KIAA0754	0.76	0.0368	KIAA0754
1027	COL1A2	0.76	0.061	collagen, type I, alpha 2
1028	SLC22A5	0.7592	0.0215	solute carrier family 22 (organic cation/carnitine transporter), member 5
1029	COL17A1	0.7592	0.0178	collagen, type XVII, alpha 1
1030	BTD	0.7592	0.0304	biotinidase
1031	CPXM2	0.7588	0.0485	carboxypeptidase X (M14 family), member 2
1032	SPRR2A	0.7586	0.0173	small proline-rich protein 2A
1033	HOXA9	0.7586	0.0011	homeobox A9
1034	ITGA10	0.7583	0.0391	integrin, alpha 10
1035	COL16A1	0.7583	0.0519	collagen, type XVI, alpha 1
1036	TPD52L1	0.7575	0.0147	tumor protein D52-like 1
1037	TMEM14C	0.7575	0.1082	transmembrane protein 14C
1038	TCEA3	0.7575	0.0208	transcription elongation factor A (SII), 3
1039	SPINK6	0.7575	0.0029	serine peptidase inhibitor, Kazal type 6
1040	LOC643733	0.7575	0.0179	caspase 4, apoptosis-related cysteine peptidase pseudogene
1041	JKAMP	0.7575	0.2101	JNK1/MAPK8-associated membrane protein
1042	SYPL1	0.7567	0.1277	synaptophysin-like 1
1043	RARG	0.7567	0.1668	retinoic acid receptor, gamma
1044	TMEM37	0.7563	0.0233	transmembrane protein 37
1045	STARD13	0.7558	0.0329	StAR-related lipid transfer (START) domain containing 13
1046	SERHL2	0.7558	0.0019	serine hydrolase-like 2
1047	SEMA5A	0.7558	0.0407	sema domain, seven thrombospondin repeats (type 1 and type 1-like), transmembrane domain (TM) and short cytoplasmic domain, (semaphorin) 5
1048	WDR78	0.7542	0.0357	WD repeat domain 78
1049	SULT2B1	0.7542	0.0686	sulfotransferase family, cytosolic, 2B, member 1
1050	TMEM119	0.7538	0.0341	transmembrane protein 119
1051	KCNK6	0.7538	0.0159	potassium channel, subfamily K, member 6
1052	FBLIM1	0.7538	0.0551	filamin binding LIM protein 1
1053	C13orf33	0.7538	0.0534	chromosome 13 open reading frame 33
1054	FOXI1	0.7533	0.0385	forkhead box I1
1055	GATSL3	0.7529	0.0286	GATS protein-like 3
1056	INPP5J	0.7525	0.0825	inositol polyphosphate-5-phosphatase J
1057	HEBP1	0.7517	0.0544	heme binding protein 1
1058	FURIN	0.7517	0.1162	furin (paired basic amino acid cleaving enzyme)
1059	CCND1	0.7517	0.0156	cyclin D1
1060	WASL	0.7508	0.0408	Wiskott-Aldrich syndrome-like
1061	TEP1	0.7508	0.0219	telomerase-associated protein 1
1062	CELSR1	0.7508	0.0101	cadherin, EGF LAG seven-pass G-type receptor 1 (flamingo homolog, Drosophila)
1063	CAPG	0.7508	0.0702	capping protein (actin filament), gelsolin-like
1064	KLK1	0.75	0.0521	kallikrein 1
1065	KCNJ13	0.75	0.0167	potassium inwardly-rectifying channel, subfamily J, member 13
1066	EEF1A1	0.75	0.0392	eukaryotic translation elongation factor 1 alpha 1
1067	ATP6V0E1	0.75	0.1246	ATPase, H+ transporting, lysosomal 9kDa, V0 subunit e1
1068	DMD	0.7492	0.0165	dystrophin
1069	AVIL	0.7492	0.0239	advillin
1070	TMEM101	0.7487	0.0428	transmembrane protein 101
1071	COG3	0.7487	0.1387	component of oligomeric golgi complex 3
1072	ARHGAP31	0.7487	0.047	Rho GTPase activating protein 31
1073	RMST	0.7486	0	rhabdomyosarcoma 2 associated transcript (non-protein coding)
1074	SCN11A	0.7483	0.0032	sodium channel, voltage-gated, type XI, alpha subunit
1075	MDFIC	0.7483	0.0431	MyoD family inhibitor domain containing
1076	LGALS3BP	0.7483	0.062	lectin, galactoside-binding, soluble, 3 binding protein
1077	ARHGEF17	0.7483	0.096	Rho guanine nucleotide exchange factor (GEF) 17
1078	FAM116A	0.7475	0.125	family with sequence similarity 116, member A
1079	DOCK6	0.7475	0.0796	dedicator of cytokinesis 6
1080	UTY	0.7467	0.0059	ubiquitously transcribed tetratricopeptide repeat gene, Y-linked
1081	TPT1	0.7467	0.0566	tumor protein, translationally-controlled 1
1082	SLFN12	0.7467	0.0344	schlafen family member 12
1083	NOD1	0.7467	0.0405	nucleotide-binding oligomerization domain containing 1
1084	RHBDD2	0.7462	0.0911	rhomboid domain containing 2
1085	C1orf198	0.7462	0.0245	chromosome 1 open reading frame 198
1086	SUGT1P3	0.745	0.006	suppressor of G2 allele of SKP1 (S. cerevisiae) pseudogene 3
1087	SNX18	0.745	0.0048	sorting nexin 18
1088	SNTB1	0.745	0.0035	syntrophin, beta 1 (dystrophin-associated protein A1, 59kDa, basic component 1)
1089	EPS8	0.745	0.0692	epidermal growth factor receptor pathway substrate 8
1090	DPP4	0.745	0.0103	dipeptidyl-peptidase 4

1091	TTC28	0.7442	0.0526	tetratricopeptide repeat domain 28
1092	ADAMTS6	0.7442	0.0211	ADAM metallopeptidase with thrombospondin type 1 motif, 6
1093	PTPRJ	0.7433	0.0241	protein tyrosine phosphatase, receptor type, J
1094	HERPUD1	0.7433	0.0762	homocysteine-inducible, endoplasmic reticulum stress-inducible, ubiquitin-like domain member 1
1095	GAS1	0.7433	0.0342	growth arrest-specific 1
1096	CPZ	0.7433	0.0768	carboxypeptidase Z
1097	CCL2	0.7433	0.0477	chemokine (C-C motif) ligand 2
1098	AOX1	0.7433	0.031	aldehyde oxidase 1
1099	TMEM59	0.7425	0.1647	transmembrane protein 59
1100	ARL15	0.7425	0.0231	ADP-ribosylation factor-like 15
1101	TACC2	0.7417	0.0157	transforming, acidic coiled-coil containing protein 2
1102	MFSD1	0.7417	0.1563	major facilitator superfamily domain containing 1
1103	CSN3	0.7417	0.0547	casein kappa
1104	C17orf109	0.7414	0.0374	chromosome 17 open reading frame 109
1105	VSIG10	0.7408	0.0093	V-set and immunoglobulin domain containing 10
1106	PAMR1	0.7408	0.075	peptidase domain containing associated with muscle regeneration 1
1107	F2RL1	0.7408	0.017	coagulation factor II (thrombin) receptor-like 1
1108	C1orf116	0.7408	0.0409	chromosome 1 open reading frame 116
1109	SLITRK6	0.74	0.0067	SLIT and NTRK-like family, member 6
1110	CYFIP1	0.74	0.08	cytoplasmic FMR1 interacting protein 1
1111	BMP2	0.74	0.0124	bone morphogenetic protein 2
1112	REEP3	0.7387	0.1017	receptor accessory protein 3
1113	MFAP5	0.7383	0.0362	microfibrillar associated protein 5
1114	TP63	0.7375	0.0856	tumor protein p63
1115	TBX3	0.7375	0.0127	T-box 3
1116	PTPN12	0.7375	0.1647	protein tyrosine phosphatase, non-receptor type 12
1117	JRKL	0.7375	0.078	jerky homolog-like (mouse)
1118	COBLL1	0.7375	0.0212	COBL-like 1
1119	PDE4D	0.7367	0.0266	phosphodiesterase 4D, cAMP-specific
1120	CD248	0.7367	0.0811	CD248 molecule, endosialin
1121	CXCL14	0.7358	0.0123	chemokine (C-X-C motif) ligand 14
1122	B4GALT1	0.7358	0.0404	UDP-Gal:betaGlcNAc beta 1,4- galactosyltransferase, polypeptide 1
1123	LOC100127886	0.735	0	uncharacterized LOC100127886
1124	FCGBP	0.735	0.0136	Fc fragment of IgG binding protein
1125	DDAH2	0.735	0.0534	dimethylarginine dimethylaminohydrolase 2
1126	C6orf58	0.735	0.0069	chromosome 6 open reading frame 58
1127	THSD7A	0.7342	0.0234	thrombospondin, type I, domain containing 7A
1128	SIK1	0.7342	0.0234	salt-inducible kinase 1
1129	PVRL2	0.7342	0.0789	poliovirus receptor-related 2 (herpesvirus entry mediator B)
1130	DET1	0.7342	0.0458	de-etiolated homolog 1 (Arabidopsis)
1131	PPAPDC1B	0.7337	0.018	phosphatidic acid phosphatase type 2 domain containing 1B
1132	MYO5B	0.7337	0.017	myosin VB
1133	ZAK	0.7333	0.028	sterile alpha motif and leucine zipper containing kinase AZK
1134	TMBIM6	0.7333	0.1052	transmembrane BAX inhibitor motif containing 6
1135	LOC344887	0.7333	0	NmrA-like family domain containing 1 pseudogene
1136	LAMA2	0.7333	0.0421	laminin, alpha 2
1137	ETV1	0.7333	0.0388	ets variant 1
1138	AGA	0.7333	0.0979	aspartylglucosaminidase
1139	ZBTB7C	0.7325	0.0112	zinc finger and BTB domain containing 7C
1140	TTC17	0.7325	0.0853	tetratricopeptide repeat domain 17
1141	RPS6KA6	0.7325	0.0124	ribosomal protein S6 kinase, 90kDa, polypeptide 6
1142	MMGT1	0.7325	0.0655	membrane magnesium transporter 1
1143	GSTM4	0.7325	0.0232	glutathione S-transferase mu 4
1144	ZBTB38	0.7317	0.0672	zinc finger and BTB domain containing 38
1145	KCNJ16	0.7317	0.0145	potassium inwardly-rectifying channel, subfamily J, member 16
1146	AFAP1	0.7317	0.0444	actin filament associated protein 1
1147	PARK2	0.7308	0.0893	parkinson protein 2, E3 ubiquitin protein ligase (parkin)
1148	KANK2	0.7308	0.052	KN motif and ankyrin repeat domains 2
1149	WDR86	0.73	0.0288	WD repeat domain 86
1150	TTC22	0.73	0.0905	tetratricopeptide repeat domain 22
1151	TPBG	0.73	0.0253	trophoblast glycoprotein
1152	TNN	0.73	0.0402	tenascin N
1153	PLAC9	0.73	0.0479	placenta-specific 9
1154	IFITM3	0.73	0.0649	interferon induced transmembrane protein 3
1155	HIPK3	0.73	0.0828	homeodomain interacting protein kinase 3
1156	CYP4F8	0.73	0.0159	cytochrome P450, family 4, subfamily F, polypeptide 8
1157	CST6	0.73	0.0076	cystatin E/M
1158	GLCE	0.7292	0.0718	glucuronic acid epimerase
1159	CWH43	0.7292	0.0103	cell wall biogenesis 43 C-terminal homolog (S. cerevisiae)
1160	RDH10	0.7287	0.0103	retinol dehydrogenase 10 (all-trans)
1161	HEATR5A	0.7287	0.147	HEAT repeat containing 5A
1162	FAM73A	0.7287	0.1126	family with sequence similarity 73, member A
1163	TUFT1	0.7283	0.0296	tuftelin 1
1164	SSR3	0.7283	0.0937	signal sequence receptor, gamma (translocon-associated protein gamma)
1165	NNMT	0.7283	0.0592	nicotinamide N-methyltransferase
1166	BDH2	0.7283	0.0719	3-hydroxybutyrate dehydrogenase, type 2
1167	TBC1D19	0.7275	0.0614	TBC1 domain family, member 19
1168	PION	0.7275	0.0805	pigeon homolog (Drosophila)
1169	IQCA1	0.7275	0.023	IQ motif containing with AAA domain 1
1170	BSPRY	0.7275	0.0221	B-box and SPRY domain containing
1171	NPTN	0.7267	0.1559	neuroplastin
1172	LOX	0.7267	0.0517	lysyl oxidase
1173	FCER1A	0.7267	0.0115	Fc fragment of IgE, high affinity I, receptor for; alpha polypeptide
1174	COG5	0.7267	0.1607	component of oligomeric golgi complex 5

1175	ZNF827	0.7262	0.0343	zinc finger protein 827
1176	SEMA3D	0.7258	0.0085	sema domain, immunoglobulin domain (Ig), short basic domain, secreted, (semaphorin) 3D
1177	GSTP1	0.7258	0.0612	glutathione S-transferase pi 1
1178	CTBP2	0.7258	0.008	C-terminal binding protein 2
1179	COX16	0.7257	0.0657	COX16 cytochrome c oxidase assembly homolog (S. cerevisiae)
1180	TTC30B	0.725	0.0511	tetratricopeptide repeat domain 30B
1181	SPPL2A	0.725	0.1136	signal peptide peptidase like 2A
1182	SLC35A4	0.725	0.1135	solute carrier family 35, member A4
1183	GRAMD3	0.725	0.0537	GRAM domain containing 3
1184	TMEM204	0.7242	0.0368	transmembrane protein 204
1185	FGF7	0.7242	0.0268	fibroblast growth factor 7
1186	TMEM133	0.7237	0.0259	transmembrane protein 133
1187	TRIP10	0.7233	0.0478	thyroid hormone receptor interactor 10
1188	TMOD3	0.7233	0.1133	tropomodulin 3 (ubiquitous)
1189	PLAU	0.7233	0.0553	plasminogen activator, urokinase
1190	TCTN1	0.7225	0.0436	tectonic family member 1
1191	SLC44A3	0.7225	0.02	solute carrier family 44, member 3
1192	SLC39A13	0.7225	0.0839	solute carrier family 39 (zinc transporter), member 13
1193	PRDM5	0.7225	0.0383	PR domain containing 5
1194	PPP1R3B	0.7225	0.0196	protein phosphatase 1, regulatory subunit 3B
1195	LEPREL1	0.7225	0.0208	leprecan-like 1
1196	IDUA	0.7225	0.1077	iduronidase, alpha-L-
1197	FUT6	0.7225	0.1523	fucosyltransferase 6 (alpha (1,3) fucosyltransferase)
1198	EDNRA	0.7225	0.0487	endothelin receptor type A
1199	DUSP23	0.7225	0.0366	dual specificity phosphatase 23
1200	CALHM2	0.7225	0.0708	calcium homeostasis modulator 2
1201	C5orf24	0.7225	0.1258	chromosome 5 open reading frame 24
1202	LOC645212	0.7217	0	uncharacterized LOC645212
1203	DOCK9	0.7217	0.0368	dedicator of cytokinesis 9
1204	MRC2	0.7208	0.101	mannose receptor, C type 2
1205	VCL	0.7192	0.0535	vinculin
1206	SPRR1B	0.7192	0.0208	small proline-rich protein 1B
1207	RPS27L	0.7192	0.0791	ribosomal protein S27-like
1208	KLK11	0.7192	0.057	kallikrein-related peptidase 11
1209	CAPN1	0.7192	0.1258	calpain 1, (mu/I) large subunit
1210	TMEM184C	0.7183	0.0757	transmembrane protein 184C
1211	STATH	0.7183	0.0114	statherin
1212	SLC30A5	0.7183	0.259	solute carrier family 30 (zinc transporter), member 5
1213	ZNF280D	0.7175	0.1916	zinc finger protein 280D
1214	TMEM87A	0.7175	0.1257	transmembrane protein 87A
1215	IRF2BP2	0.7175	0.0356	interferon regulatory factor 2 binding protein 2
1216	GHDC	0.7175	0.0803	GH3 domain containing
1217	ANXA2P3	0.7175	0.0251	annexin A2 pseudogene 3
1218	TMED7	0.7167	0.2358	transmembrane emp24 protein transport domain containing 7
1219	THBS2	0.7167	0.0548	thrombospondin 2
1220	CALML5	0.7167	0.0162	calmodulin-like 5
1221	AHCYL2	0.7167	0.0329	adenosylhomocysteinase-like 2
1222	TRIP4	0.7158	0.17	thyroid hormone receptor interactor 4
1223	EFNA5	0.7142	0.0305	ephrin-A5
1224	COL4A6	0.7142	0.0216	collagen, type IV, alpha 6
1225	CLCA2	0.7142	0.0303	chloride channel accessory 2
1226	PEAR1	0.7138	0.0319	platelet endothelial aggregation receptor 1
1227	NDFIP2	0.7138	0.1016	Nedd4 family interacting protein 2
1228	SLC20A2	0.7133	0.0151	solute carrier family 20 (phosphate transporter), member 2
1229	PTGFR	0.7133	0.0193	prostaglandin F receptor (FP)
1230	TCEAL8	0.7125	0.1287	transcription elongation factor A (SII)-like 8
1231	GPR56	0.7125	0.0206	G protein-coupled receptor 56
1232	FANK1	0.7125	0.0173	fibronectin type III and ankyrin repeat domains 1
1233	ANK3	0.7125	0.0325	ankyrin 3, node of Ranvier (ankyrin G)
1234	PLSCR3	0.7114	0.0476	phospholipid scramblase 3
1235	KIAA1671	0.7114	0.0148	KIAA1671
1236	TGFB2	0.7108	0.0438	transforming growth factor, beta 2
1237	SPINT1	0.7108	0.0506	serine peptidase inhibitor, Kunitz type 1
1238	PCDHGB6	0.7108	0.0098	protocadherin gamma subfamily B, 6
1239	GULP1	0.7108	0.0328	GULP, engulfment adaptor PTB domain containing 1
1240	ADAMTS1	0.7108	0.0329	ADAM metallopeptidase with thrombospondin type 1 motif, 1
1241	KRT18	0.71	0.0152	keratin 18
1242	FOLR1	0.71	0.0167	folate receptor 1 (adult)
1243	CC2D2A	0.71	0.029	coiled-coil and C2 domain containing 2A
1244	BBS12	0.71	0.0735	Bardet-Biedl syndrome 12
1245	COL6A2	0.7092	0.1032	collagen, type VI, alpha 2
1246	SLC26A4-AS1	0.7088	0.007	SLC26A4 antisense RNA 1 (non-protein coding)
1247	PPAP2A	0.7083	0.0669	phosphatidic acid phosphatase type 2A
1248	MYOM3	0.7075	0.0572	myomesin family, member 3
1249	MAN1A2	0.7075	0.0893	mannosidase, alpha, class 1A, member 2
1250	KANSL1L	0.7075	0.0865	KAT8 regulatory NSL complex subunit 1-like
1251	ANXA5	0.7075	0.1287	annexin A5
1252	LOC100129794	0.7071	0.0128	uncharacterized LOC100129794
1253	UEVLD	0.7067	0.1528	UEV and lactate/malate dehyrogenase domains
1254	RYK	0.7067	0.11	receptor-like tyrosine kinase
1255	DUSP6	0.7067	0.0357	dual specificity phosphatase 6
1256	CTBS	0.7067	0.1537	chitobiase, di-N-acetyl-
1257	DIO1	0.7058	0.0079	deiodinase, iodothyronine, type I
1258	ANG	0.7058	0.0433	angiogenin, ribonuclease, RNase A family, 5

1259	RNF170	0.705	0.146	ring finger protein 170
1260	NEXN	0.705	0.0573	nexilin (F actin binding protein)
1261	CLPTM1L	0.705	0.12	CLPTM1-like
1262	LIMS2	0.7042	0.1197	LIM and senescent cell antigen-like domains 2
1263	ALDH3A1	0.7042	0.0309	aldehyde dehydrogenase 3 family, member A1
1264	C1orf172	0.7038	0.0294	chromosome 1 open reading frame 172
1265	ZNF214	0.7033	0.0297	zinc finger protein 214
1266	SNAI2	0.7033	0.0498	snail homolog 2 (Drosophila)
1267	PDGFD	0.7033	0.0376	platelet derived growth factor D
1268	HNMT	0.7025	0.0608	histamine N-methyltransferase
1269	FMO1	0.7025	0.0387	flavin containing monooxygenase 1
1270	ACP6	0.7025	0.026	acid phosphatase 6, lysophosphatidic
1271	SNX7	0.7017	0.0592	sorting nexin 7
1272	SCNN1A	0.7017	0.0667	sodium channel, non-voltage-gated 1 alpha subunit
1273	PDE10A	0.7017	0.0221	phosphodiesterase 10A
1274	ABCC9	0.7017	0.0464	ATP-binding cassette, sub-family C (CFTR/MRP), member 9
1275	C2orf40	0.7013	0.0178	chromosome 2 open reading frame 40
1276	SVEP1	0.7008	0.1533	sushi, von Willebrand factor type A, EGF and pentraxin domain containing 1
1277	PDE3A	0.7008	0.0089	phosphodiesterase 3A, cGMP-inhibited
1278	LHFP	0.7008	0.0792	lipoma HMGIC fusion partner
1279	IER3	0.7008	0.0533	immediate early response 3
1280	GPNMB	0.7008	0.0511	glycoprotein (transmembrane) nmb
1281	EHD4	0.7008	0.0588	EH-domain containing 4
1282	TCHH	0.7	0.0128	trichohyalin
1283	FRAS1	0.7	0.0166	Fraser syndrome 1
1284	C10orf57	0.7	0.0433	chromosome 10 open reading frame 57
1285	ARL1	0.7	0.242	ADP-ribosylation factor-like 1
1286	CPA4	0.6992	0.0252	carboxypeptidase A4
1287	SRGAP1	0.6988	0.0377	SLIT-ROBO Rho GTPase activating protein 1
1288	SLC30A7	0.6988	0.1669	solute carrier family 30 (zinc transporter), member 7
1289	JMJD8	0.6988	0.1026	jumonji domain containing 8
1290	DYX1C1	0.6988	0.0108	dyslexia susceptibility 1 candidate 1
1291	WFDC2	0.6983	0.0117	WAP four-disulfide core domain 2
1292	TGIF1	0.6983	0.0258	TGFB-induced factor homeobox 1
1293	CD207	0.6983	0.0296	CD207 molecule, langerin
1294	XIAP	0.6975	0.0518	X-linked inhibitor of apoptosis
1295	WWC2	0.6975	0.0397	WW and C2 domain containing 2
1296	TXNIP	0.6975	0.0828	thioredoxin interacting protein
1297	SULF2	0.6975	0.0343	sulfatase 2
1298	MUCL1	0.6975	0.0052	mucin-like 1
1299	CNN1	0.6975	0.0598	calponin 1, basic, smooth muscle
1300	UGGT2	0.6967	0.0402	UDP-glucose glycoprotein glucosyltransferase 2
1301	IPW	0.6967	0.0567	imprinted in Prader-Willi syndrome (non-protein coding)
1302	TRABD2B	0.6963	0.0481	TraB domain containing 2B
1303	ARHGEF19	0.6963	0.0335	Rho guanine nucleotide exchange factor (GEF) 19
1304	SMAGP	0.695	0.0163	small cell adhesion glycoprotein
1305	C1orf56	0.695	0.0068	chromosome 1 open reading frame 56
1306	NBR1	0.6942	0.1767	neighbor of BRCA1 gene 1
1307	MCFD2	0.6942	0.1514	multiple coagulation factor deficiency 2
1308	COPZ1	0.6942	0.1418	coatomer protein complex, subunit zeta 1
1309	RHOJ	0.6938	0.0776	ras homolog family member J
1310	PURB	0.6938	0.0401	purine-rich element binding protein B
1311	RHOC	0.6933	0.1261	ras homolog family member C
1312	TAX1BP3	0.6929	0.0624	Tax1 (human T-cell leukemia virus type I) binding protein 3
1313	LIF	0.6925	0.0471	leukemia inhibitory factor
1314	HECTD2	0.6925	0.0945	HECT domain containing E3 ubiquitin protein ligase 2
1315	CCPG1	0.6925	0.1698	cell cycle progression 1
1316	TJP1	0.6917	0.0676	tight junction protein 1
1317	BMP6	0.6917	0.0169	bone morphogenetic protein 6
1318	AGRN	0.6917	0.096	agrin
1319	ACAD8	0.6917	0.0746	acyl-CoA dehydrogenase family, member 8
1320	SERPINB11	0.6913	0.0051	serpin peptidase inhibitor, clade B (ovalbumin), member 11 (gene/pseudogene)
1321	VEGFA	0.6908	0.0308	vascular endothelial growth factor A
1322	PDE5A	0.6908	0.0648	phosphodiesterase 5A, cGMP-specific
1323	GALNT12	0.6908	0.008	UDP-N-acetyl-alpha-D-galactosamine:polypeptide N-acetylgalactosaminyltransferase 12 (GalNAc-T12)
1324	CTSL1	0.6908	0.0726	cathepsin L1
1325	RERGL	0.69	0.0101	RERG/RAS-like
1326	RAET1E	0.69	0.0117	retinoic acid early transcript 1E
1327	KLK8	0.69	0.103	kallikrein-related peptidase 8
1328	FBXL3	0.69	0.1963	F-box and leucine-rich repeat protein 3
1329	CLCNKB	0.69	0.1544	chloride channel, voltage-sensitive Kb
1330	CKAP4	0.69	0.0463	cytoskeleton-associated protein 4
1331	C3orf52	0.69	0.0189	chromosome 3 open reading frame 52
1332	SQSTM1	0.6892	0.0873	sequestosome 1
1333	PDIA6	0.6892	0.1601	protein disulfide isomerase family A, member 6
1334	PLCD4	0.6888	0.0141	phospholipase C, delta 4
1335	KDELC2	0.6888	0.086	KDEL (Lys-Asp-Glu-Leu) containing 2
1336	C11orf54	0.6888	0.1677	chromosome 11 open reading frame 54
1337	T	0.6883	0.0687	T, brachyury homolog (mouse)
1338	SFN	0.6883	0.0346	stratifin
1339	LOC126987	0.6883	0	tight junction protein 3 (zona occludens 3) pseudogene
1340	FHL2	0.6883	0.0373	four and a half LIM domains 2
1341	DAP	0.6883	0.0855	death-associated protein
1342	BICC1	0.6883	0.0271	bicaudal C homolog 1 (Drosophila)

1343	PHF20L1	0.6875	0.1326	PHD finger protein 20-like 1
1344	FAM84B	0.6875	0.012	family with sequence similarity 84, member B
1345	MICA	0.6871	0.0136	MHC class I polypeptide-related sequence A
1346	HLA-A	0.6867	0.0972	major histocompatibility complex, class I, A
1347	CLINT1	0.6867	0.1264	clathrin interactor 1
1348	PDLIM1	0.6858	0.0214	PDZ and LIM domain 1
1349	GPR124	0.6858	0.0873	G protein-coupled receptor 124
1350	EBF2	0.6858	0.0467	early B-cell factor 2
1351	SPG21	0.685	0.1054	spastic paraplegia 21 (autosomal recessive, Mast syndrome)
1352	SEC31A	0.685	0.1343	SEC31 homolog A (S. cerevisiae)
1353	HES1	0.685	0.013	hairy and enhancer of split 1, (Drosophila)
1354	TRAM1	0.6842	0.1895	translocation associated membrane protein 1
1355	PARP3	0.6842	0.0412	poly (ADP-ribose) polymerase family, member 3
1356	GPR116	0.6842	0.0432	G protein-coupled receptor 116
1357	C1QTNF1	0.6842	0.1218	C1q and tumor necrosis factor related protein 1
1358	C11orf93	0.6837	0.0077	chromosome 11 open reading frame 93
1359	HSD11B2	0.6833	0.0215	hydroxysteroid (11-beta) dehydrogenase 2
1360	ECM1	0.6833	0.0663	extracellular matrix protein 1
1361	CYB5A	0.6833	0.057	cytochrome b5 type A (microsomal)
1362	ALDH18A1	0.6833	0.1043	aldehyde dehydrogenase 18 family, member A1
1363	PYROXD2	0.6825	0.0373	pyridine nucleotide-disulphide oxidoreductase domain 2
1364	C2orf18	0.6825	0.0898	chromosome 2 open reading frame 18
1365	ANAPC16	0.6825	0.1362	anaphase promoting complex subunit 16
1366	PLEKHA5	0.6817	0.066	pleckstrin homology domain containing, family A member 5
1367	NEDD9	0.6817	0.0424	neural precursor cell expressed, developmentally down-regulated 9
1368	TXLNG2P	0.6812	0.004	taxilin gamma 2, pseudogene
1369	TMEM185A	0.6812	0.012	transmembrane protein 185A
1370	MCEE	0.6812	0.0888	methylmalonyl CoA epimerase
1371	DBNL	0.6812	0.1321	drebrin-like
1372	C1orf85	0.6812	0.0903	chromosome 1 open reading frame 85
1373	ANTXR2	0.6812	0.1083	anthrax toxin receptor 2
1374	LAD1	0.6808	0.0781	ladinin 1
1375	EGFLAM	0.68	0.0214	EGF-like, fibronectin type III and laminin G domains
1376	SCGB2A2	0.6792	0.0075	secretoglobin, family 2A, member 2
1377	FBP1	0.6792	0.0293	fructose-1,6-bisphosphatase 1
1378	CD302	0.6786	0.0734	CD302 molecule
1379	ZFP36L1	0.6783	0.0741	zinc finger protein 36, C3H type-like 1
1380	SLC12A4	0.6783	0.137	solute carrier family 12 (potassium/chloride transporters), member 4
1381	MMP7	0.6783	0.0105	matrix metallopeptidase 7 (matrilysin, uterine)
1382	WDR31	0.6775	0.0108	WD repeat domain 31
1383	SERTAD3	0.6775	0.0322	SERTA domain containing 3
1384	LGMN	0.6775	0.0459	legumain
1385	HLA-C	0.6775	0.1133	major histocompatibility complex, class I, C
1386	GRN	0.6775	0.1576	granulin
1387	CASP7	0.6775	0.0745	caspase 7, apoptosis-related cysteine peptidase
1388	TWSG1	0.6767	0.14	twisted gastrulation homolog 1 (Drosophila)
1389	FZD10	0.6767	0.0106	frizzled family receptor 10
1390	TTC8	0.6762	0.1038	tetratricopeptide repeat domain 8
1391	TMEM205	0.6762	0.1018	transmembrane protein 205
1392	TMEM106A	0.6762	0.0419	transmembrane protein 106A
1393	SSFA2	0.6758	0.1558	sperm specific antigen 2
1394	SLC39A7	0.675	0.0528	solute carrier family 39 (zinc transporter), member 7
1395	CHID1	0.675	0.0868	chitinase domain containing 1
1396	SMO	0.6742	0.0726	smoothened, frizzled family receptor
1397	PROL1	0.6742	0.0922	proline rich, lacrimal 1
1398	ASAH1	0.6742	0.1602	N-acylsphingosine amidohydrolase (acid ceramidase) 1
1399	CDHR5	0.6733	0.2065	cadherin-related family member 5
1400	CDCP1	0.6733	0.0353	CUB domain containing protein 1
1401	BST1	0.6733	0.0389	bone marrow stromal cell antigen 1
1402	AXL	0.6733	0.0854	AXL receptor tyrosine kinase
1403	AQR	0.6733	0.1751	aquarius homolog (mouse)
1404	FMO4	0.6725	0.0838	flavin containing monooxygenase 4
1405	EMC10	0.6725	0.0626	ER membrane protein complex subunit 10
1406	ATP7A	0.6725	0.1016	ATPase, Cu++ transporting, alpha polypeptide
1407	ALDH1A1	0.6725	0.0216	aldehyde dehydrogenase 1 family, member A1
1408	TMCO3	0.6717	0.0946	transmembrane and coiled-coil domains 3
1409	PCSK5	0.6717	0.0218	proprotein convertase subtilisin/kexin type 5
1410	LIFR	0.6717	0.0434	leukemia inhibitory factor receptor alpha
1411	HLA-B	0.6717	0.1291	major histocompatibility complex, class I, B
1412	C12orf39	0.6717	0.0683	chromosome 12 open reading frame 39
1413	AKR1C1	0.6717	0.0124	aldo-keto reductase family 1, member C1 (dihydrodiol dehydrogenase 1; 20-alpha (3-alpha)-hydroxysteroid dehydrogenase)
1414	TNS1	0.6708	0.0985	tensin 1
1415	TMEM47	0.6708	0.0686	transmembrane protein 47
1416	SLC16A7	0.6708	0.0218	solute carrier family 16, member 7 (monocarboxylic acid transporter 2)
1417	TRIM56	0.67	0.0542	tripartite motif containing 56
1418	OCIAD1	0.67	0.1673	OCIA domain containing 1
1419	PEX7	0.6692	0.0702	peroxisomal biogenesis factor 7
1420	PDK4	0.6692	0.0387	pyruvate dehydrogenase kinase, isozyme 4
1421	EREG	0.6692	0.0148	epiregulin
1422	FAM214A	0.6687	0.1861	family with sequence similarity 214, member A
1423	B3GALTL	0.6687	0.0415	beta 1,3-galactosyltransferase-like
1424	YIPF6	0.6683	0.0936	Yip1 domain family, member 6
1425	SELE	0.6683	0.0158	selectin E
1426	PRKCI	0.6683	0.0694	protein kinase C, iota

1427	ZMPSTE24	0.6675	0.2395	zinc metallopeptidase STE24 homolog (S. cerevisiae)
1428	TMX3	0.6675	0.2445	thioredoxin-related transmembrane protein 3
1429	TMEM50A	0.6675	0.1602	transmembrane protein 50A
1430	RND3	0.6675	0.0735	Rho family GTPase 3
1431	NEK7	0.6675	0.2128	NIMA (never in mitosis gene a)-related kinase 7
1432	SCNN1B	0.6667	0.0537	sodium channel, non-voltage-gated 1, beta subunit
1433	PCDH7	0.6667	0.0359	protocadherin 7
1434	FLG	0.6667	0.0074	filaggrin
1435	MCCC2	0.6658	0.0896	methylcrotonoyl-CoA carboxylase 2 (beta)
1436	LBH	0.6658	0.0371	limb bud and heart development homolog (mouse)
1437	GFPT1	0.6658	0.1648	glutamine--fructose-6-phosphate transaminase 1
1438	CALU	0.6658	0.1149	calumenin
1439	PHF11	0.665	0.1146	PHD finger protein 11
1440	OSTC	0.665	0.1333	oligosaccharyltransferase complex subunit
1441	EFCAB13	0.665	0.014	EF-hand calcium binding domain 13
1442	DNAJB14	0.665	0.1957	DnaJ (Hsp40) homolog, subfamily B, member 14
1443	BTNL9	0.665	0.0066	butyrophilin-like 9
1444	KCNK5	0.6642	0.01	potassium channel, subfamily K, member 5
1445	GLIPR1	0.6642	0.1064	GLI pathogenesis-related 1
1446	ANPEP	0.6642	0.0437	alanyl (membrane) aminopeptidase
1447	TPRG1L	0.6637	0.1016	tumor protein p63 regulated 1-like
1448	TMEM18	0.6637	0.0433	transmembrane protein 18
1449	SETD7	0.6637	0.0884	SET domain containing (lysine methyltransferase) 7
1450	PARD3B	0.6637	0.0516	par-3 partitioning defective 3 homolog B (C. elegans)
1451	FAM172A	0.6633	0.1496	family with sequence similarity 172, member A
1452	CTSC	0.6633	0.0832	cathepsin C
1453	CLEC3B	0.6633	0.0754	C-type lectin domain family 3, member B
1454	TRIM5	0.6625	0.0529	tripartite motif containing 5
1455	TMEM54	0.6625	0.05	transmembrane protein 54
1456	S100A13	0.6625	0.0475	S100 calcium binding protein A13
1457	RILPL2	0.6625	0.0526	Rab interacting lysosomal protein-like 2
1458	FUCA2	0.6625	0.0833	fucosidase, alpha-L- 2, plasma
1459	FAM120AOS	0.6625	0.0091	family with sequence similarity 120A opposite strand
1460	CCDC102A	0.6612	0.0378	coiled-coil domain containing 102A
1461	AMY2B	0.6612	0.1132	amylase, alpha 2B (pancreatic)
1462	IVD	0.6608	0.0541	isovaleryl-CoA dehydrogenase
1463	IL11RA	0.6608	0.0734	interleukin 11 receptor, alpha
1464	GATA3	0.6608	0.0089	GATA binding protein 3
1465	LRRC8E	0.66	0.024	leucine rich repeat containing 8 family, member E
1466	CTSD	0.66	0.1524	cathepsin D
1467	COL28A1	0.66	0.0073	collagen, type XXVIII, alpha 1
1468	SRPX	0.6592	0.0677	sushi-repeat containing protein, X-linked
1469	SECTM1	0.6592	0.0665	secreted and transmembrane 1
1470	LRRFIP1	0.6592	0.0855	leucine rich repeat (in FLII) interacting protein 1
1471	CHMP1B	0.6592	0.0671	charged multivesicular body protein 1B
1472	TMEM230	0.6587	0.0948	transmembrane protein 230
1473	FAM83B	0.6587	0.013	family with sequence similarity 83, member B
1474	TAS2R10	0.6583	0.0263	taste receptor, type 2, member 10
1475	TANK	0.6583	0.216	TRAF family member-associated NFKB activator
1476	CDON	0.6583	0.0469	Cdon homolog (mouse)
1477	APH1A	0.6583	0.0613	anterior pharynx defective 1 homolog A (C. elegans)
1478	STAM2	0.6575	0.2279	signal transducing adaptor molecule (SH3 domain and ITAM motif) 2
1479	SOX7	0.6575	0.011	SRY (sex determining region Y)-box 7
1480	FBXO32	0.6575	0.0558	F-box protein 32
1481	ROR2	0.6567	0.0239	receptor tyrosine kinase-like orphan receptor 2
1482	RAB1A	0.6567	0.2311	RAB1A, member RAS oncogene family
1483	FLJ11235	0.6567	0.0307	uncharacterized FLJ11235
1484	C4orf32	0.6563	0.0349	chromosome 4 open reading frame 32
1485	STK3	0.6558	0.1059	serine/threonine kinase 3
1486	IMPACT	0.6558	0.193	Impact homolog (mouse)
1487	EGR1	0.6558	0.0277	early growth response 1
1488	DEGS2	0.6557	0.0837	delta(4)-desaturase, sphingolipid 2
1489	TEAD3	0.655	0.0451	TEA domain family member 3
1490	MRAP2	0.655	0.0177	melanocortin 2 receptor accessory protein 2
1491	BRI3	0.655	0.1002	brain protein I3
1492	RHOD	0.6542	0.1229	ras homolog family member D
1493	HSPB6	0.6542	0.1468	heat shock protein, alpha-crystallin-related, B6
1494	ASAP2	0.6542	0.0879	ArfGAP with SH3 domain, ankyrin repeat and PH domain 2
1495	TRIM4	0.6538	0.098	tripartite motif containing 4
1496	DHRS4-AS1	0.6538	0.0692	DHRS4 antisense RNA 1 (non-protein coding)
1497	MYLK	0.6533	0.073	myosin light chain kinase
1498	MIPEP	0.6533	0.0726	mitochondrial intermediate peptidase
1499	MFAP3	0.6533	0.1451	microfibrillar-associated protein 3
1500	SPEF2	0.6525	0.0235	sperm flagellar 2
1501	MIR22HG	0.6525	0.0927	MIR22 host gene (non-protein coding)
1502	HSPA1A	0.6525	0.0191	heat shock 70kDa protein 1A
1503	EPHB4	0.6525	0.1007	EPH receptor B4
1504	CYP20A1	0.6525	0.1209	cytochrome P450, family 20, subfamily A, polypeptide 1
1505	USH1C	0.6517	0.1376	Usher syndrome 1C (autosomal recessive, severe)
1506	NPHP1	0.6513	0.0135	nephronophthisis 1 (juvenile)
1507	FTSJD1	0.6513	0.239	FtsJ methyltransferase domain containing 1
1508	CREBRF	0.6513	0.188	CREB3 regulatory factor
1509	TCIRG1	0.6508	0.1364	T-cell, immune regulator 1, ATPase, H+ transporting, lysosomal V0 subunit A3
1510	SPRR1A	0.6508	0.0794	small proline-rich protein 1A

1511	MYCT1	0.6508	0.0501	myc target 1
1512	HEG1	0.6508	0.1033	HEG homolog 1 (zebrafish)
1513	FKBP14	0.6508	0.0701	FK506 binding protein 14, 22 kDa
1514	MUC20	0.65	0.0272	mucin 20, cell surface associated
1515	FAM177A1	0.65	0.1244	family with sequence similarity 177, member A1
1516	ALPK2	0.65	0.0239	alpha-kinase 2
1517	PGF	0.6492	0.0445	placental growth factor
1518	NMRK1	0.6492	0.118	nicotinamide riboside kinase 1
1519	HPGDS	0.6492	0.039	hematopoietic prostaglandin D synthase
1520	GJA5	0.6492	0.0468	gap junction protein, alpha 5, 40kDa
1521	FAP	0.6492	0.0753	fibroblast activation protein, alpha
1522	ERLIN1	0.6492	0.0885	ER lipid raft associated 1
1523	BBS1	0.6492	0.1038	Bardet-Biedl syndrome 1
1524	AQP5	0.6492	0.147	aquaporin 5
1525	IFT43	0.6488	0.0834	intraflagellar transport 43 homolog (Chlamydomonas)
1526	AGR3	0.6488	0.0045	anterior gradient 3 homolog (Xenopus laevis)
1527	RPL10	0.6483	0.0108	ribosomal protein L10
1528	PRRG4	0.6483	0.0238	proline rich Gla (G-carboxyglutamic acid) 4 (transmembrane)
1529	LRBA	0.6483	0.1592	LPS-responsive vesicle trafficking, beach and anchor containing
1530	REN	0.6475	0.0552	renin
1531	PLOD1	0.6475	0.1257	procollagen-lysine, 2-oxoglutarate 5-dioxygenase 1
1532	TNNC2	0.6467	0.0776	troponin C type 2 (fast)
1533	CASK	0.6467	0.0564	calcium/calmodulin-dependent serine protein kinase (MAGUK family)
1534	C15orf40	0.6463	0.1125	chromosome 15 open reading frame 40
1535	ASB5	0.6463	0.0018	ankyrin repeat and SOCS box containing 5
1536	TNFSF10	0.6458	0.0854	tumor necrosis factor (ligand) superfamily, member 10
1537	HGD	0.6458	0.0301	homogentisate 1,2-dioxygenase
1538	GANAB	0.6458	0.1174	glucosidase, alpha; neutral AB
1539	CD44	0.6458	0.086	CD44 molecule (Indian blood group)
1540	TMEM213	0.645	0.0028	transmembrane protein 213
1541	RHCG	0.645	0.0534	Rh family, C glycoprotein
1542	TRIM2	0.6442	0.0598	tripartite motif containing 2
1543	DCBLD2	0.6442	0.0529	discoidin, CUB and LCCL domain containing 2
1544	ARHGEF16	0.6442	0.1632	Rho guanine nucleotide exchange factor (GEF) 16
1545	SLC5A5	0.6433	0.2285	solute carrier family 5 (sodium iodide symporter), member 5
1546	PDIA4	0.6433	0.0742	protein disulfide isomerase family A, member 4
1547	MANBA	0.6433	0.0985	mannosidase, beta A, lysosomal
1548	BCL3	0.6433	0.0996	B-cell CLL/lymphoma 3
1549	ZNF132	0.6425	0.015	zinc finger protein 132
1550	MFAP4	0.6425	0.079	microfibrillar-associated protein 4
1551	EMC4	0.6425	0.1785	ER membrane protein complex subunit 4
1552	OGN	0.6417	0.0446	osteoglycin
1553	PDZK1	0.6414	0.0023	PDZ domain containing 1
1554	VIPR1	0.6408	0.0509	vasoactive intestinal peptide receptor 1
1555	VAV3	0.6408	0.0191	vav 3 guanine nucleotide exchange factor
1556	TFE3	0.6408	0.1235	transcription factor binding to IGHM enhancer 3
1557	ENOSF1	0.6408	0.047	enolase superfamily member 1
1558	SDF2	0.64	0.0879	stromal cell-derived factor 2
1559	MLKL	0.64	0.0757	mixed lineage kinase domain-like
1560	HOPX	0.64	0.0158	HOP homeobox
1561	FNDC3A	0.64	0.1651	fibronectin type III domain containing 3A
1562	C4orf52	0.64	0.1304	chromosome 4 open reading frame 52
1563	TRAK1	0.6392	0.0495	trafficking protein, kinesin binding 1
1564	DHRS1	0.6392	0.0672	dehydrogenase/reductase (SDR family) member 1
1565	ZFP36L2	0.6383	0.0562	zinc finger protein 36, C3H type-like 2
1566	VTCN1	0.6383	0.0121	V-set domain containing T cell activation inhibitor 1
1567	MR1	0.6383	0.0909	major histocompatibility complex, class I-related
1568	CPD	0.6383	0.1714	carboxypeptidase D
1569	SAMD13	0.6375	0.0212	sterile alpha motif domain containing 13
1570	RAI14	0.6375	0.0595	retinoic acid induced 14
1571	EXOC8	0.6375	0.2097	exocyst complex component 8
1572	CRISPLD2	0.6375	0.0865	cysteine-rich secretory protein LCCL domain containing 2
1573	UNC93B1	0.6373	0.0845	unc-93 homolog B1 (C. elegans)
1574	TM9SF4	0.6367	0.1009	transmembrane 9 superfamily protein member 4
1575	PLXNA2	0.6367	0.0506	plexin A2
1576	BCAM	0.6367	0.2085	basal cell adhesion molecule (Lutheran blood group)
1577	TMEM127	0.6358	0.1221	transmembrane protein 127
1578	STK38L	0.6358	0.1469	serine/threonine kinase 38 like
1579	ST6GALNAC2	0.6358	0.0289	ST6 (alpha-N-acetyl-neuraminyl-2,3-beta-galactosyl-1,3)-N-acetylgalactosaminide alpha-2,6-sialyltransferase 2
1580	HSPA5	0.6358	0.0721	heat shock 70kDa protein 5 (glucose-regulated protein, 78kDa)
1581	FARP1	0.6358	0.0466	FERM, RhoGEF (ARHGEF) and pleckstrin domain protein 1 (chondrocyte-derived)
1582	TMEM115	0.635	0.1398	transmembrane protein 115
1583	SNX1	0.6342	0.153	sorting nexin 1
1584	PODXL	0.6342	0.0195	podocalyxin-like
1585	ELK3	0.6342	0.0716	ELK3, ETS-domain protein (SRF accessory protein 2)
1586	PRDM1	0.6325	0.0833	PR domain containing 1, with ZNF domain
1587	CRIP2	0.6325	0.0791	cysteine-rich protein 2
1588	CD14	0.6325	0.1	CD14 molecule
1589	ACO1	0.6325	0.0558	aconitase 1, soluble
1590	TRPV4	0.6317	0.1079	transient receptor potential cation channel, subfamily V, member 4
1591	HOXA5	0.6317	0.0103	homeobox A5
1592	CTSZ	0.6317	0.0665	cathepsin Z
1593	FOXP1	0.6313	0.0573	forkhead box P1
1594	ST14	0.6308	0.0729	suppression of tumorigenicity 14 (colon carcinoma)

1595	RCN3	0.6308	0.1108	reticulocalbin 3, EF-hand calcium binding domain
1596	LTBP4	0.6308	0.1373	latent transforming growth factor beta binding protein 4
1597	ANXA13	0.6308	0.06	annexin A13
1598	ACVR2A	0.6308	0.0833	activin A receptor, type IIA
1599	TIMP4	0.63	0.0194	TIMP metallopeptidase inhibitor 4
1600	TEK	0.63	0.1115	TEK tyrosine kinase, endothelial
1601	SMAD6	0.63	0.0572	SMAD family member 6
1602	PRRX2	0.63	0.16	paired related homeobox 2
1603	OAS1	0.63	0.0646	2'-5'-oligoadenylate synthetase 1, 40/46kDa
1604	LGR6	0.63	0.007	leucine-rich repeat containing G protein-coupled receptor 6
1605	GNPTG	0.63	0.1182	N-acetylglucosamine-1-phosphate transferase, gamma subunit
1606	COL4A5	0.63	0.0376	collagen, type IV, alpha 5
1607	ARSK	0.63	0.0475	arylsulfatase family, member K
1608	ACTN4	0.6292	0.118	actinin, alpha 4
1609	SDR16C5	0.6288	0.0084	short chain dehydrogenase/reductase family 16C, member 5
1610	OR7E24	0.6286	0.0217	olfactory receptor, family 7, subfamily E, member 24
1611	WLS	0.6283	0.0512	wntless homolog (Drosophila)
1612	SFXN3	0.6283	0.0941	sideroflexin 3
1613	MAP3K2	0.6283	0.1628	mitogen-activated protein kinase kinase kinase 2
1614	LAMC2	0.6283	0.063	laminin, gamma 2
1615	PKDCC	0.6275	0.02	protein kinase domain containing, cytoplasmic homolog (mouse)
1616	NEDD4	0.6275	0.0534	neural precursor cell expressed, developmentally down-regulated 4, E3 ubiquitin protein ligase
1617	MRPS6	0.6275	0.0465	mitochondrial ribosomal protein S6
1618	KRCC1	0.6275	0.2211	lysine-rich coiled-coil 1
1619	CDKL1	0.6275	0.0207	cyclin-dependent kinase-like 1 (CDC2-related kinase)
1620	CAPN6	0.6275	0.0486	calpain 6
1621	C8orf31	0.6275	0.0103	chromosome 8 open reading frame 31
1622	TRPC4	0.6267	0.0465	transient receptor potential cation channel, subfamily C, member 4
1623	CSGALNACT1	0.6267	0.0518	chondroitin sulfate N-acetylgalactosaminyltransferase 1
1624	B3GALNT2	0.6267	0.0637	beta-1,3-N-acetylgalactosaminyltransferase 2
1625	HLA-F-AS1	0.6264	0.0285	HLA-F antisense RNA 1 (non-protein coding)
1626	RGMB	0.6263	0.0119	RGM domain family, member B
1627	TSC22D3	0.6258	0.0767	TSC22 domain family, member 3
1628	C9orf152	0.6257	0.0221	chromosome 9 open reading frame 152
1629	TFAP2C	0.625	0.0185	transcription factor AP-2 gamma (activating enhancer binding protein 2 gamma)
1630	C1orf43	0.625	0.163	chromosome 1 open reading frame 43
1631	ADH1B	0.6245	0.06	alcohol dehydrogenase 1B (class I), beta polypeptide
1632	TMEM80	0.6242	0.0588	transmembrane protein 80
1633	SLC22A3	0.6242	0.0049	solute carrier family 22 (extraneuronal monoamine transporter), member 3
1634	GHR	0.6242	0.0469	growth hormone receptor
1635	CHD9	0.6242	0.2359	chromodomain helicase DNA binding protein 9
1636	NAGLU	0.6233	0.1248	N-acetylglucosaminidase, alpha
1637	SYNC	0.6225	0.0496	syncoilin, intermediate filament protein
1638	SOCS6	0.6225	0.1087	suppressor of cytokine signaling 6
1639	PTER	0.6225	0.1053	phosphotriesterase related
1640	PABPC4L	0.6225	0.0257	poly(A) binding protein, cytoplasmic 4-like
1641	MYO1D	0.6225	0.0444	myosin ID
1642	DDIT4L	0.6225	0.0079	DNA-damage-inducible transcript 4-like
1643	CEBPB	0.6225	0.0915	CCAAT/enhancer binding protein (C/EBP), beta
1644	C10orf32	0.6225	0.1794	chromosome 10 open reading frame 32
1645	FGF2	0.6217	0.0456	fibroblast growth factor 2 (basic)
1646	F11R	0.6217	0.0436	F11 receptor
1647	ETHE1	0.6217	0.0891	ethylmalonic encephalopathy 1
1648	EPHA1	0.6217	0.0552	EPH receptor A1
1649	XG	0.6212	0.0129	Xg blood group
1650	FKBP7	0.6212	0.0766	FK506 binding protein 7
1651	ZNF140	0.6208	0.2142	zinc finger protein 140
1652	METTL7A	0.6208	0.0728	methyltransferase like 7A
1653	ESRP1	0.6208	0.0325	epithelial splicing regulatory protein 1
1654	CHPT1	0.6208	0.0348	choline phosphotransferase 1
1655	RGS2	0.62	0.057	regulator of G-protein signaling 2, 24kDa
1656	NCKAP1	0.62	0.189	NCK-associated protein 1
1657	LNX1	0.62	0.0262	ligand of numb-protein X 1, E3 ubiquitin protein ligase
1658	KIAA0040	0.62	0.0174	KIAA0040
1659	H6PD	0.62	0.1254	hexose-6-phosphate dehydrogenase (glucose 1-dehydrogenase)
1660	GOLPH3L	0.62	0.1432	golgi phosphoprotein 3-like
1661	DUOXA1	0.62	0.1109	dual oxidase maturation factor 1
1662	PRKG1	0.6192	0.0806	protein kinase, cGMP-dependent, type I
1663	PCCA	0.6192	0.1128	propionyl CoA carboxylase, alpha polypeptide
1664	KRT83	0.6192	0.1723	keratin 83
1665	GOLGB1	0.6192	0.1687	golgin B1
1666	SH3RF2	0.6187	0.0274	SH3 domain containing ring finger 2
1667	CLDN23	0.6187	0.0136	claudin 23
1668	PPA2	0.6183	0.184	pyrophosphatase (inorganic) 2
1669	PKP1	0.6183	0.1618	plakophilin 1 (ectodermal dysplasia/skin fragility syndrome)
1670	KRT6B	0.6183	0.0334	keratin 6B
1671	ARHGEF10L	0.6183	0.0362	Rho guanine nucleotide exchange factor (GEF) 10-like
1672	SLC28A3	0.6175	0.0567	solute carrier family 28 (sodium-coupled nucleoside transporter), member 3
1673	SH3PXD2B	0.6175	0.066	SH3 and PX domains 2B
1674	PADI1	0.6175	0.2475	peptidyl arginine deiminase, type I
1675	CXorf23	0.6175	0.2199	chromosome X open reading frame 23
1676	CST5	0.6175	0.049	cystatin D
1677	SNHG8	0.6162	0.0697	small nucleolar RNA host gene 8 (non-protein coding)
1678	FAM210B	0.6162	0.038	family with sequence similarity 210, member B

1679	PRKX	0.6158	0.0301	protein kinase, X-linked
1680	KCTD3	0.6158	0.2448	potassium channel tetramerisation domain containing 3
1681	HCP5	0.6158	0.0523	HLA complex P5 (non-protein coding)
1682	FAM120A	0.6158	0.1127	family with sequence similarity 120A
1683	CDH16	0.6158	0.1354	cadherin 16, KSP-cadherin
1684	ARAF	0.6158	0.1346	v-raf murine sarcoma 3611 viral oncogene homolog
1685	VIM	0.615	0.1253	vimentin
1686	COL6A1	0.615	0.148	collagen, type VI, alpha 1
1687	AHNAK2	0.615	0.0645	AHNAK nucleoprotein 2
1688	ATRAID	0.6142	0.1664	all-trans retinoic acid-induced differentiation factor
1689	ACADL	0.6142	0.0325	acyl-CoA dehydrogenase, long chain
1690	VWDE	0.6137	0.0069	von Willebrand factor D and EGF domains
1691	LAYN	0.6137	0.0547	layilin
1692	TNFAIP2	0.6133	0.1026	tumor necrosis factor, alpha-induced protein 2
1693	TES	0.6133	0.0553	testis derived transcript (3 LIM domains)
1694	OGDH	0.6133	0.1012	oxoglutarate (alpha-ketoglutarate) dehydrogenase (lipoamide)
1695	LPO	0.6133	0.1916	lactoperoxidase
1696	PCDHGB5	0.6127	0.0768	protocadherin gamma subfamily B, 5
1697	CLIP4	0.6125	0.0895	CAP-GLY domain containing linker protein family, member 4
1698	BHLHE40	0.6117	0.0871	basic helix-loop-helix family, member e40
1699	SNX21	0.6112	0.0614	sorting nexin family member 21
1700	SFTA3	0.6112	0.0014	surfactant associated 3
1701	SLC13A1	0.6108	0.0108	solute carrier family 13 (sodium/sulfate symporters), member 1
1702	SAR1A	0.6108	0.0657	SAR1 homolog A (S. cerevisiae)
1703	PKP3	0.6108	0.1295	plakophilin 3
1704	GNPDA2	0.61	0.2378	glucosamine-6-phosphate deaminase 2
1705	ERG	0.61	0.0441	v-ets erythroblastosis virus E26 oncogene homolog (avian)
1706	UST	0.6092	0.0259	uronyl-2-sulfotransferase
1707	TULP3	0.6092	0.0522	tubby like protein 3
1708	PLGRKT	0.6092	0.1112	plasminogen receptor, C-terminal lysine transmembrane protein
1709	JUNB	0.6092	0.1029	jun B proto-oncogene
1710	DPT	0.6083	0.0965	dermatopontin
1711	CTF1	0.6083	0.1222	cardiotrophin 1
1712	PIGG	0.6075	0.115	phosphatidylinositol glycan anchor biosynthesis, class G
1713	PDE8B	0.6075	0.0397	phosphodiesterase 8B
1714	HEXA	0.6075	0.1294	hexosaminidase A (alpha polypeptide)
1715	DRAM1	0.6075	0.1187	DNA-damage regulated autophagy modulator 1
1716	CTTN	0.6075	0.0304	cortactin
1717	VGLL3	0.6067	0.0713	vestigial like 3 (Drosophila)
1718	GEMIN8	0.6067	0.0408	gem (nuclear organelle) associated protein 8
1719	FOS	0.6067	0.0448	FBJ murine osteosarcoma viral oncogene homolog
1720	MYL12A	0.6058	0.183	myosin, light chain 12A, regulatory, non-sarcomeric
1721	GSTT1	0.6058	0.0065	glutathione S-transferase theta 1
1722	EFNB1	0.6058	0.0787	ephrin-B1
1723	C8orf4	0.6058	0.0255	chromosome 8 open reading frame 4
1724	MXRA7	0.605	0.0756	matrix-remodelling associated 7
1725	LYPLAL1	0.605	0.1936	lysophospholipase-like 1
1726	CTAGE5	0.605	0.081	CTAGE family, member 5
1727	HTR2B	0.6042	0.0246	5-hydroxytryptamine (serotonin) receptor 2B, G protein-coupled
1728	FKBP2	0.6042	0.0994	FK506 binding protein 2, 13kDa
1729	ESM1	0.6042	0.0081	endothelial cell-specific molecule 1
1730	CHST3	0.6042	0.0986	carbohydrate (chondroitin 6) sulfotransferase 3
1731	ZMAT1	0.6037	0.0889	zinc finger, matrin-type 1
1732	TMEM214	0.6033	0.1338	transmembrane protein 214
1733	GPR64	0.6025	0.0166	G protein-coupled receptor 64
1734	GJB3	0.6025	0.1492	gap junction protein, beta 3, 31kDa
1735	CFD	0.6025	0.0613	complement factor D (adipsin)
1736	CDS1	0.6025	0.0501	CDP-diacylglycerol synthase (phosphatidate cytidylyltransferase) 1
1737	SQRDL	0.6017	0.1265	sulfide quinone reductase-like (yeast)
1738	SEMA3A	0.6017	0.0225	sema domain, immunoglobulin domain (Ig), short basic domain, secreted, (semaphorin) 3A
1739	RAB23	0.6017	0.1364	RAB23, member RAS oncogene family
1740	PHACTR2	0.6017	0.1145	phosphatase and actin regulator 2
1741	MSN	0.6017	0.1464	moesin
1742	H2AFJ	0.6017	0.0194	H2A histone family, member J
1743	CES2	0.6017	0.0814	carboxylesterase 2
1744	FAM83H	0.6012	0.0776	family with sequence similarity 83, member H
1745	RAB38	0.6008	0.0109	RAB38, member RAS oncogene family
1746	MYL12B	0.6008	0.1492	myosin, light chain 12B, regulatory
1747	ASAP1-IT1	0.6008	0.0816	ASAP1 intronic transcript 1 (non-protein coding)
1748	SLIT2	0.6	0.0617	slit homolog 2 (Drosophila)
1749	MGST2	0.6	0.0737	microsomal glutathione S-transferase 2
1750	LRRC70	0.6	0.0369	leucine rich repeat containing 70
1751	LDB2	0.6	0.0634	LIM domain binding 2
1752	KRT17	0.6	0.0374	keratin 17
1753	ELMO3	0.6	0.066	engulfment and cell motility 3
1754	APOA1BP	0.6	0.1749	apolipoprotein A-I binding protein
1755	TRIP11	0.5992	0.1314	thyroid hormone receptor interactor 11
1756	MUC7	0.5992	0.0053	mucin 7, secreted
1757	HEPH	0.5992	0.0445	hephaestin
1758	ACTG2	0.5992	0.0419	actin, gamma 2, smooth muscle, enteric
1759	WDFY1	0.5987	0.144	WD repeat and FYVE domain containing 1
1760	STXBP4	0.5987	0.0663	syntaxin binding protein 4
1761	EPB41L4A-AS1	0.5987	0.0949	EPB41L4A antisense RNA 1 (non-protein coding)
1762	WDFY3	0.5983	0.1615	WD repeat and FYVE domain containing 3

1763	SLC35F2	0.5983	0.0549	solute carrier family 35, member F2
1764	SLC2A9	0.5983	0.0636	solute carrier family 2 (facilitated glucose transporter), member 9
1765	PIGT	0.5983	0.1037	phosphatidylinositol glycan anchor biosynthesis, class T
1766	NPR1	0.5983	0.1943	natriuretic peptide receptor A/guanylate cyclase A (atrionatriuretic peptide receptor A)
1767	MSANTD2	0.5983	0.1529	Myb/SANT-like DNA-binding domain containing 2
1768	EMD	0.5983	0.0961	emerin
1769	DEGS1	0.5983	0.1804	delta(4)-desaturase, sphingolipid 1
1770	CST2	0.5983	0.0345	cystatin SA
1771	SLC44A2	0.5975	0.1053	solute carrier family 44, member 2
1772	PI15	0.5975	0.0381	peptidase inhibitor 15
1773	NOX1	0.5975	0.2081	NADPH oxidase 1
1774	LONRF3	0.5975	0.0251	LON peptidase N-terminal domain and ring finger 3
1775	GDAP2	0.5975	0.083	ganglioside induced differentiation associated protein 2
1776	CGREF1	0.5975	0.0297	cell growth regulator with EF-hand domain 1
1777	CD164	0.5975	0.2563	CD164 molecule, sialomucin
1778	C5orf42	0.5975	0.1056	chromosome 5 open reading frame 42
1779	WWC3	0.5967	0.0649	WWC family member 3
1780	PHKB	0.5967	0.2489	phosphorylase kinase, beta
1781	FNBP1L	0.5967	0.1484	formin binding protein 1-like
1782	ANKH	0.5967	0.024	ankylosis, progressive homolog (mouse)
1783	CCNYL1	0.5962	0.1413	cyclin Y-like 1
1784	RORA	0.5958	0.0815	RAR-related orphan receptor A
1785	RGS5	0.5958	0.0735	regulator of G-protein signaling 5
1786	FAS	0.5958	0.1187	Fas (TNF receptor superfamily, member 6)
1787	NXN	0.595	0.0354	nucleoredoxin
1788	IRX5	0.595	0.0105	iroquois homeobox 5
1789	GK5	0.595	0.1327	glycerol kinase 5 (putative)
1790	CPS1	0.595	0.0275	carbamoyl-phosphate synthase 1, mitochondrial
1791	PRR4	0.5943	0.0271	proline rich 4 (lacrimal)
1792	C1RL	0.5942	0.093	complement component 1, r subcomponent-like
1793	HAS3	0.5938	0.0052	hyaluronan synthase 3
1794	COX20	0.5938	0.0508	COX20 Cox2 chaperone homolog (S. cerevisiae)
1795	PPFIBP2	0.5933	0.039	PTPRF interacting protein, binding protein 2 (liprin beta 2)
1796	LIMD1	0.5933	0.0219	LIM domains containing 1
1797	SLC9A1	0.5925	0.103	solute carrier family 9, subfamily A (NHE1, cation proton antiporter 1), member 1
1798	GXYLT2	0.5925	0.0471	glucoside xylosyltransferase 2
1799	GPR1	0.5925	0.0181	G protein-coupled receptor 1
1800	TP53I3	0.5917	0.0635	tumor protein p53 inducible protein 3
1801	SGK1	0.5917	0.0748	serum/glucocorticoid regulated kinase 1
1802	COG7	0.5917	0.1	component of oligomeric golgi complex 7
1803	ST5	0.5908	0.0801	suppression of tumorigenicity 5
1804	CAPNS1	0.5908	0.1853	calpain, small subunit 1
1805	ABHD11	0.5908	0.0785	abhydrolase domain containing 11
1806	S1PR3	0.59	0.0338	sphingosine-1-phosphate receptor 3
1807	PEX12	0.59	0.1294	peroxisomal biogenesis factor 12
1808	PDGFRA	0.59	0.0798	platelet-derived growth factor receptor, alpha polypeptide
1809	MRGPRX3	0.59	0.0019	MAS-related GPR, member X3
1810	C15orf57	0.59	0.0551	chromosome 15 open reading frame 57
1811	PLEC	0.5892	0.139	plectin
1812	GREM2	0.5892	0.0483	gremlin 2
1813	ACADVL	0.5892	0.1335	acyl-CoA dehydrogenase, very long chain
1814	TMEM42	0.5888	0.0831	transmembrane protein 42
1815	MFSD8	0.5888	0.2159	major facilitator superfamily domain containing 8
1816	ADAM33	0.5888	0.1087	ADAM metallopeptidase domain 33
1817	SCARA3	0.5883	0.045	scavenger receptor class A, member 3
1818	ROCK2	0.5883	0.097	Rho-associated, coiled-coil containing protein kinase 2
1819	PPARG	0.5883	0.0215	peroxisome proliferator-activated receptor gamma
1820	NUCB1	0.5875	0.1845	nucleobindin 1
1821	MLL3	0.5875	0.1622	myeloid/lymphoid or mixed-lineage leukemia 3
1822	IFITM2	0.5875	0.1049	interferon induced transmembrane protein 2
1823	ABLIM1	0.5875	0.0517	actin binding LIM protein 1
1824	GNL3L	0.5867	0.0687	guanine nucleotide binding protein-like 3 (nucleolar)-like
1825	TMEM19	0.5863	0.1301	transmembrane protein 19
1826	ERMAP	0.5858	0.0463	erythroblast membrane-associated protein (Scianna blood group)
1827	STEAP2	0.585	0.0321	STEAP family member 2, metalloreductase
1828	ERC1	0.585	0.0578	ELKS/RAB6-interacting/CAST family member 1
1829	EFNB2	0.585	0.0382	ephrin-B2
1830	KIAA1609	0.5842	0.0321	KIAA1609
1831	GALC	0.5842	0.1312	galactosylceramidase
1832	FOSL2	0.5842	0.1161	FOS-like antigen 2
1833	DGKI	0.5842	0.0395	diacylglycerol kinase, iota
1834	PRTG	0.5838	0.0272	protogenin
1835	GALNT5	0.5838	0.0687	UDP-N-acetyl-alpha-D-galactosamine:polypeptide N-acetylgalactosaminyltransferase 5 (GalNAc-T5)
1836	FAM83C	0.5838	0.0265	family with sequence similarity 83, member C
1837	ELMOD2	0.5838	0.2364	ELMO/CED-12 domain containing 2
1838	HYMAI	0.5836	0.0542	hydatidiform mole associated and imprinted (non-protein coding)
1839	RAB27B	0.5833	0.0241	RAB27B, member RAS oncogene family
1840	KRT34	0.5833	0.0714	keratin 34
1841	IPP	0.5833	0.0771	intracisternal A particle-promoted polypeptide
1842	COMMD10	0.5833	0.2541	COMM domain containing 10
1843	SLC22A4	0.5825	0.0433	solute carrier family 22 (organic cation/ergothioneine transporter), member 4
1844	KHNYN	0.5825	0.0668	KH and NYN domain containing
1845	CAMK2D	0.5825	0.118	calcium/calmodulin-dependent protein kinase II delta
1846	APOL6	0.5825	0.09	apolipoprotein L, 6

1847	PARD3	0.5817	0.0393	par-3 partitioning defective 3 homolog (C. elegans)
1848	GPR110	0.5817	0.0577	G protein-coupled receptor 110
1849	VEZT	0.5813	0.2818	vezatin, adherens junctions transmembrane protein
1850	FBXO4	0.5813	0.1069	F-box protein 4
1851	TNFRSF11A	0.5808	0.0152	tumor necrosis factor receptor superfamily, member 11a, NFKB activator
1852	NR4A2	0.5808	0.049	nuclear receptor subfamily 4, group A, member 2
1853	NINJ1	0.5808	0.1134	ninjurin 1
1854	DOK4	0.5808	0.1136	docking protein 4
1855	CDH19	0.5808	0.0143	cadherin 19, type 2
1856	MEOX2	0.58	0.0235	mesenchyme homeobox 2
1857	ALG2	0.58	0.155	asparagine-linked glycosylation 2, alpha-1,3-mannosyltransferase homolog (S. cerevisiae)
1858	SYTL2	0.5792	0.056	synaptotagmin-like 2
1859	SLC24A1	0.5792	0.0511	solute carrier family 24 (sodium/potassium/calcium exchanger), member 1
1860	N4BP2L2	0.5792	0.218	NEDD4 binding protein 2-like 2
1861	TLR4	0.5783	0.1033	toll-like receptor 4
1862	SCAF11	0.5783	0.2095	SR-related CTD-associated factor 11
1863	MBNL2	0.5783	0.1361	muscleblind-like splicing regulator 2
1864	KRT15	0.5783	0.0161	keratin 15
1865	ITPR2	0.5783	0.0875	inositol 1,4,5-trisphosphate receptor, type 2
1866	SNAP23	0.5775	0.2085	synaptosomal-associated protein, 23kDa
1867	KIF16B	0.5775	0.1266	kinesin family member 16B
1868	KDSR	0.5775	0.1712	3-ketodihydrosphingosine reductase
1869	FN1	0.5775	0.1032	fibronectin 1
1870	ELL2	0.5775	0.0921	elongation factor, RNA polymerase II, 2
1871	ATAD1	0.5775	0.2835	ATPase family, AAA domain containing 1
1872	ASL	0.5775	0.1062	argininosuccinate lyase
1873	ANKRD30B	0.5771	0.0048	ankyrin repeat domain 30B
1874	STAP2	0.5767	0.044	signal transducing adaptor family member 2
1875	CYB561	0.5767	0.0443	cytochrome b-561
1876	TSHZ3	0.5763	0.0863	teashirt zinc finger homeobox 3
1877	TGFA	0.5758	0.0728	transforming growth factor, alpha
1878	KLK12	0.5758	0.1864	kallikrein-related peptidase 12
1879	CETN2	0.5758	0.184	centrin, EF-hand protein, 2
1880	CDC42EP2	0.5758	0.1171	CDC42 effector protein (Rho GTPase binding) 2
1881	ANKRD10	0.5758	0.1705	ankyrin repeat domain 10
1882	PPCS	0.575	0.1886	phosphopantothenoylcysteine synthetase
1883	PIK3R1	0.575	0.1306	phosphoinositide-3-kinase, regulatory subunit 1 (alpha)
1884	NME6	0.575	0.059	NME/NM23 nucleoside diphosphate kinase 6
1885	GALK2	0.575	0.1164	galactokinase 2
1886	CCDC115	0.575	0.1151	coiled-coil domain containing 115
1887	NBL1	0.5743	0.0805	neuroblastoma, suppression of tumorigenicity 1
1888	WNT2	0.5742	0.1261	wingless-type MMTV integration site family member 2
1889	TNFRSF12A	0.5742	0.1146	tumor necrosis factor receptor superfamily, member 12A
1890	TMEM9B	0.5742	0.1706	TMEM9 domain family, member B
1891	CANX	0.5742	0.2337	calnexin
1892	CAB39L	0.5742	0.0425	calcium binding protein 39-like
1893	ZMYND11	0.5733	0.2151	zinc finger, MYND-type containing 11
1894	MYOCD	0.5733	0.0155	myocardin
1895	LTBP1	0.5733	0.0637	latent transforming growth factor beta binding protein 1
1896	LRRC17	0.5733	0.0504	leucine rich repeat containing 17
1897	KITLG	0.5733	0.0369	KIT ligand
1898	IDE	0.5733	0.1796	insulin-degrading enzyme
1899	HOXC6	0.5733	0.0177	homeobox C6
1900	AVPI1	0.5733	0.0481	arginine vasopressin-induced 1
1901	PIEZO1	0.5725	0.1272	piezo-type mechanosensitive ion channel component 1
1902	ITPKC	0.5725	0.1199	inositol-trisphosphate 3-kinase C
1903	CASP6	0.5725	0.1638	caspase 6, apoptosis-related cysteine peptidase
1904	AIM1	0.5725	0.0493	absent in melanoma 1
1905	ZNF264	0.5717	0.0988	zinc finger protein 264
1906	PIGR	0.5717	0.1516	polymeric immunoglobulin receptor
1907	C10orf118	0.5717	0.1269	chromosome 10 open reading frame 118
1908	ZDHHC21	0.5713	0.1771	zinc finger, DHHC-type containing 21
1909	TMEM17	0.5713	0.0392	transmembrane protein 17
1910	STC2	0.5708	0.0354	stanniocalcin 2
1911	PRKAA2	0.5708	0.0433	protein kinase, AMP-activated, alpha 2 catalytic subunit
1912	NDUFA4L2	0.5708	0.0431	NADH dehydrogenase (ubiquinone) 1 alpha subcomplex, 4-like 2
1913	ANXA11	0.5708	0.1141	annexin A11
1914	ADRA2A	0.5708	0.0465	adrenoceptor alpha 2A
1915	RERG	0.57	0.0359	RAS-like, estrogen-regulated, growth inhibitor
1916	OVOL2	0.57	0.0496	ovo-like 2 (Drosophila)
1917	LINC00312	0.57	0.0715	long intergenic non-protein coding RNA 312
1918	KIAA0141	0.57	0.0599	KIAA0141
1919	IVL	0.57	0.1345	involucrin
1920	CNOT6L	0.57	0.1401	CCR4-NOT transcription complex, subunit 6-like
1921	AADAC	0.57	0.0152	arylacetamide deacetylase
1922	GTF2IRD1	0.5692	0.0406	GTF2I repeat domain containing 1
1923	FOXP2	0.5688	0.0433	forkhead box P2
1924	SP140L	0.5683	0.0787	SP140 nuclear body protein-like
1925	MGAT4B	0.5683	0.1325	mannosyl (alpha-1,3-)-glycoprotein beta-1,4-N-acetylglucosaminyltransferase, isozyme B
1926	KLK5	0.5683	0.0801	kallikrein-related peptidase 5
1927	IGF2R	0.5683	0.1299	insulin-like growth factor 2 receptor
1928	STIM2	0.5675	0.1148	stromal interaction molecule 2
1929	HNF4G	0.5675	0.0223	hepatocyte nuclear factor 4, gamma
1930	GJA4	0.5675	0.0684	gap junction protein, alpha 4, 37kDa

1931	FAM125A	0.5675	0.1464	family with sequence similarity 125, member A
1932	EPN3	0.5675	0.0701	epsin 3
1933	DPAGT1	0.5675	0.1551	dolichyl-phosphate (UDP-N-acetylglucosamine) N-acetylglucosaminephosphotransferase 1 (GlcNAc-1-P transferase)
1934	SLURP1	0.5667	0.1833	secreted LY6/PLAUR domain containing 1
1935	RALB	0.5667	0.1284	v-ral simian leukemia viral oncogene homolog B (ras related; GTP binding protein)
1936	PNPLA4	0.5667	0.061	patatin-like phospholipase domain containing 4
1937	ORAI3	0.5667	0.1237	ORAI calcium release-activated calcium modulator 3
1938	KANK1	0.5667	0.0465	KN motif and ankyrin repeat domains 1
1939	ETFDH	0.5667	0.1629	electron-transferring-flavoprotein dehydrogenase
1940	ZNF630	0.5663	0.0369	zinc finger protein 630
1941	SLC5A12	0.5658	0.0167	solute carrier family 5 (sodium/glucose cotransporter), member 12
1942	RBBP9	0.5658	0.0413	retinoblastoma binding protein 9
1943	NPY1R	0.5658	0.0203	neuropeptide Y receptor Y1
1944	HSPB8	0.5658	0.0546	heat shock 22kDa protein 8
1945	GLB1	0.5658	0.188	galactosidase, beta 1
1946	VPS13C	0.565	0.2517	vacuolar protein sorting 13 homolog C (S. cerevisiae)
1947	MFSD5	0.565	0.1584	major facilitator superfamily domain containing 5
1948	HSD17B2	0.5642	0.0218	hydroxysteroid (17-beta) dehydrogenase 2
1949	BAZ2B	0.5642	0.2383	bromodomain adjacent to zinc finger domain, 2B
1950	B3GALT5	0.5642	0.0636	UDP-Gal:betaGlcNAc beta 1,3-galactosyltransferase, polypeptide 5
1951	MACC1	0.5638	0.0247	metastasis associated in colon cancer 1
1952	ARRDC4	0.5638	0.0756	arrestin domain containing 4
1953	PHLDA2	0.5633	0.0732	pleckstrin homology-like domain, family A, member 2
1954	LYPD3	0.5633	0.0938	LY6/PLAUR domain containing 3
1955	KCTD12	0.5633	0.11	potassium channel tetramerisation domain containing 12
1956	CDC14B	0.5633	0.0593	CDC14 cell division cycle 14 homolog B (S. cerevisiae)
1957	LOC647979	0.5629	0.1957	uncharacterized LOC647979
1958	TMEM144	0.5625	0.0481	transmembrane protein 144
1959	SLFN11	0.5625	0.0853	schlafen family member 11
1960	PRELID2	0.5625	0.0116	PRELI domain containing 2
1961	PPP1CB	0.5625	0.267	protein phosphatase 1, catalytic subunit, beta isozyme
1962	NFIC	0.5625	0.0857	nuclear factor I/C (CCAAT-binding transcription factor)
1963	GPR87	0.5625	0.0205	G protein-coupled receptor 87
1964	FRY	0.5625	0.0931	furry homolog (Drosophila)
1965	CGNL1	0.5625	0.0426	cingulin-like 1
1966	KIAA1109	0.5617	0.1861	KIAA1109
1967	EXPH5	0.5617	0.0623	exophilin 5
1968	DNAJC17	0.5617	0.082	DnaJ (Hsp40) homolog, subfamily C, member 17
1969	ACOX1	0.5617	0.1294	acyl-CoA oxidase 1, palmitoyl
1970	SLC4A2	0.5608	0.1514	solute carrier family 4, anion exchanger, member 2 (erythrocyte membrane protein band 3-like 1)
1971	SIM2	0.5608	0.0562	single-minded homolog 2 (Drosophila)
1972	GAB1	0.5608	0.118	GRB2-associated binding protein 1
1973	DLST	0.5608	0.0457	dihydrolipoamide S-succinyltransferase (E2 component of 2-oxo-glutarate complex)
1974	ZNF552	0.56	0.0341	zinc finger protein 552
1975	SYNJ2BP	0.56	0.1794	synaptojanin 2 binding protein
1976	SEMA6D	0.56	0.0889	sema domain, transmembrane domain (TM), and cytoplasmic domain, (semaphorin) 6D
1977	RPN2	0.56	0.153	ribophorin II
1978	PYGB	0.56	0.1108	phosphorylase, glycogen; brain
1979	PKN2	0.56	0.2347	protein kinase N2
1980	KCNE3	0.56	0.0228	potassium voltage-gated channel, Isk-related family, member 3
1981	EBF4	0.56	0.1157	early B-cell factor 4
1982	CLIC2	0.56	0.0836	chloride intracellular channel 2
1983	BOD1	0.56	0.153	biorientation of chromosomes in cell division 1
1984	AGTRAP	0.56	0.1267	angiotensin II receptor-associated protein
1985	TNKS2	0.5592	0.2377	tankyrase, TRF1-interacting ankyrin-related ADP-ribose polymerase 2
1986	PTHLH	0.5592	0.0483	parathyroid hormone-like hormone
1987	IL1RN	0.5592	0.1246	interleukin 1 receptor antagonist
1988	HTN3	0.5592	0.0099	histatin 3
1989	CORO1B	0.5592	0.175	coronin, actin binding protein, 1B
1990	LINC00478	0.5587	0.0341	long intergenic non-protein coding RNA 478
1991	TAPBPL	0.5583	0.094	TAP binding protein-like
1992	CUBN	0.5583	0.0385	cubilin (intrinsic factor-cobalamin receptor)
1993	TAPBP	0.5575	0.1576	TAP binding protein (tapasin)
1994	SDC4	0.5575	0.0743	syndecan 4
1995	PDLIM4	0.5575	0.0782	PDZ and LIM domain 4
1996	LYZ	0.5575	0.0623	lysozyme
1997	KRT13	0.5575	0.027	keratin 13
1998	IFNE	0.5575	0.0095	interferon, epsilon
1999	LCN10	0.5571	0.1472	lipocalin 10
2000	ZMYM2	0.5567	0.2406	zinc finger, MYM-type 2
2001	SMR3A	0.5567	0.1208	submaxillary gland androgen regulated protein 3A
2002	NMB	0.5567	0.026	neuromedin B
2003	FAM129A	0.5567	0.0778	family with sequence similarity 129, member A
2004	TMPRSS4	0.5558	0.0996	transmembrane protease, serine 4
2005	SHB	0.5558	0.0922	Src homology 2 domain containing adaptor protein B
2006	MEOX1	0.5558	0.0194	mesenchyme homeobox 1
2007	CORIN	0.5558	0.0462	corin, serine peptidase
2008	GBP1P1	0.5557	0	guanylate binding protein 1, interferon-inducible pseudogene 1
2009	GBP3	0.555	0.1126	guanylate binding protein 3
2010	WBP5	0.5542	0.1281	WW domain binding protein 5
2011	PIGB	0.5542	0.2198	phosphatidylinositol glycan anchor biosynthesis, class B
2012	MYL6	0.5542	0.1539	myosin, light chain 6, alkali, smooth muscle and non-muscle
2013	CXCL1	0.5542	0.0572	chemokine (C-X-C motif) ligand 1 (melanoma growth stimulating activity, alpha)
2014	TMEM123	0.5533	0.245	transmembrane protein 123

2015	SLC44A4	0.5533	0.0439	solute carrier family 44, member 4
2016	SGMS1	0.5533	0.2493	sphingomyelin synthase 1
2017	SPDEF	0.5525	0.25	SAM pointed domain containing ets transcription factor
2018	MARVELD2	0.5525	0.0326	MARVEL domain containing 2
2019	TEX261	0.5517	0.1125	testis expressed 261
2020	CD82	0.5517	0.1044	CD82 molecule
2021	ANXA10	0.5508	0.036	annexin A10
2022	ABHD4	0.5508	0.131	abhydrolase domain containing 4
2023	TMEM167B	0.55	0.219	transmembrane protein 167B
2024	LOC92249	0.55	0.0607	uncharacterized LOC92249
2025	COX7A1	0.55	0.087	cytochrome c oxidase subunit VIIa polypeptide 1 (muscle)
2026	CDK10	0.55	0.1348	cyclin-dependent kinase 10
2027	C12orf23	0.55	0.2389	chromosome 12 open reading frame 23
2028	ABP1	0.55	0.0237	amiloride binding protein 1 (amine oxidase (copper-containing))
2029	SGSH	0.5492	0.1255	N-sulfoglucosamine sulfohydrolase
2030	PTGES	0.5492	0.0766	prostaglandin E synthase
2031	MKLN1	0.5492	0.2249	muskelin 1, intracellular mediator containing kelch motifs
2032	ATP1B1	0.5492	0.0635	ATPase, Na+/K+ transporting, beta 1 polypeptide
2033	LOC401093	0.5486	0.0406	uncharacterized LOC401093
2034	PMEPA1	0.5483	0.0563	prostate transmembrane protein, androgen induced 1
2035	MLEC	0.5483	0.1407	malectin
2036	MAGED2	0.5483	0.1381	melanoma antigen family D, 2
2037	ITPR3	0.5483	0.0867	inositol 1,4,5-trisphosphate receptor, type 3
2038	ZDHHC9	0.5475	0.0582	zinc finger, DHHC-type containing 9
2039	SPRR3	0.5475	0.0403	small proline-rich protein 3
2040	HLA-E	0.5475	0.1754	major histocompatibility complex, class I, E
2041	FHL1	0.5475	0.0721	four and a half LIM domains 1
2042	FBLN7	0.5475	0.0321	fibulin 7
2043	HOXA11	0.5467	0.0783	homeobox A11
2044	FAM63B	0.5467	0.1308	family with sequence similarity 63, member B
2045	MPP7	0.5462	0.0499	membrane protein, palmitoylated 7 (MAGUK p55 subfamily member 7)
2046	LMBRD1	0.5458	0.2513	LMBR1 domain containing 1
2047	ALDH2	0.5458	0.0764	aldehyde dehydrogenase 2 family (mitochondrial)
2048	PHPT1	0.545	0.1262	phosphohistidine phosphatase 1
2049	PCOLCE	0.545	0.115	procollagen C-endopeptidase enhancer
2050	LGSN	0.545	0.0055	lengsin, lens protein with glutamine synthetase domain
2051	FAM20C	0.545	0.0879	family with sequence similarity 20, member C
2052	BAG3	0.545	0.0812	BCL2-associated athanogene 3
2053	SYNE2	0.5442	0.0802	spectrin repeat containing, nuclear envelope 2
2054	STX17	0.5442	0.1318	syntaxin 17
2055	GLS	0.5442	0.0994	glutaminase
2056	FAM189A2	0.5442	0.0657	family with sequence similarity 189, member A2
2057	KLHDC8B	0.5437	0.083	kelch domain containing 8B
2058	HMCN1	0.5437	0.0654	hemicentin 1
2059	EIF2AK4	0.5437	0.2092	eukaryotic translation initiation factor 2 alpha kinase 4
2060	CLDN12	0.5437	0.132	claudin 12
2061	CAPN8	0.5437	0.0304	calpain 8
2062	C14orf28	0.5437	0.1564	chromosome 14 open reading frame 28
2063	HLA-J	0.5433	0.12	major histocompatibility complex, class I, J (pseudogene)
2064	ETV3	0.5433	0.1045	ets variant 3
2065	CARD10	0.5433	0.0555	caspase recruitment domain family, member 10
2066	ARFGEF2	0.5433	0.2229	ADP-ribosylation factor guanine nucleotide-exchange factor 2 (brefeldin A-inhibited)
2067	OSBPL3	0.5425	0.0837	oxysterol binding protein-like 3
2068	NAAA	0.5425	0.0545	N-acylethanolamine acid amidase
2069	MATN3	0.5425	0.0236	matrilin 3
2070	KLK7	0.5425	0.1316	kallikrein-related peptidase 7
2071	GPC6	0.5425	0.0515	glypican 6
2072	ERRFI1	0.5425	0.0775	ERBB receptor feedback inhibitor 1
2073	CYBASC3	0.5425	0.1315	cytochrome b, ascorbate dependent 3
2074	COMMD6	0.5425	0.0659	COMM domain containing 6
2075	C7orf63	0.5425	0.0584	chromosome 7 open reading frame 63
2076	ABHD14B	0.5425	0.1304	abhydrolase domain containing 14B
2077	WDR13	0.5417	0.1581	WD repeat domain 13
2078	VAMP8	0.5417	0.1334	vesicle-associated membrane protein 8 (endobrevin)
2079	PLEK2	0.5417	0.0455	pleckstrin 2
2080	IKBKB	0.5417	0.1227	inhibitor of kappa light polypeptide gene enhancer in B-cells, kinase beta
2081	DDR2	0.5417	0.1028	discoidin domain receptor tyrosine kinase 2
2082	CLCN5	0.5417	0.04	chloride channel, voltage-sensitive 5
2083	SMCR7	0.5412	0.0507	Smith-Magenis syndrome chromosome region, candidate 7
2084	TNC	0.5408	0.0782	tenascin C
2085	PHYH	0.5408	0.1371	phytanoyl-CoA 2-hydroxylase
2086	LRRC31	0.5408	0.0344	leucine rich repeat containing 31
2087	DLG3	0.5408	0.0626	discs, large homolog 3 (Drosophila)
2088	CST1	0.5408	0.005	cystatin SN
2089	TRIM47	0.54	0.0928	tripartite motif containing 47
2090	TMEM62	0.54	0.0365	transmembrane protein 62
2091	SLC26A9	0.54	0.0148	solute carrier family 26, member 9
2092	SLC25A16	0.54	0.1045	solute carrier family 25 (mitochondrial carrier; Graves disease autoantigen), member 16
2093	KRT5	0.54	0.0487	keratin 5
2094	GBP2	0.5392	0.1358	guanylate binding protein 2, interferon-inducible
2095	CACNA2D1	0.5392	0.1084	calcium channel, voltage-dependent, alpha 2/delta subunit 1
2096	FBXO30	0.5387	0.2389	F-box protein 30
2097	TMPRSS2	0.5383	0.1214	transmembrane protease, serine 2
2098	NCOA3	0.5383	0.1612	nuclear receptor coactivator 3

2099	ARHGAP12	0.5383	0.2705	Rho GTPase activating protein 12
2100	SMAD9	0.5375	0.0821	SMAD family member 9
2101	SLC10A2	0.5375	0.0622	solute carrier family 10 (sodium/bile acid cotransporter family), member 2
2102	HLA-F	0.5375	0.1438	major histocompatibility complex, class I, F
2103	DNAJC10	0.5375	0.306	DnaJ (Hsp40) homolog, subfamily C, member 10
2104	CYB5D1	0.5375	0.0433	cytochrome b5 domain containing 1
2105	MYL5	0.5367	0.0698	myosin, light chain 5, regulatory
2106	LRP6	0.5367	0.0974	low density lipoprotein receptor-related protein 6
2107	SPTY2D1	0.5362	0.2137	SPT2, Suppressor of Ty, domain containing 1 (S. cerevisiae)
2108	EFCAB4A	0.5362	0.0527	EF-hand calcium binding domain 4A
2109	C15orf48	0.5362	0.051	chromosome 15 open reading frame 48
2110	SLC35D2	0.5358	0.0877	solute carrier family 35, member D2
2111	OGFRL1	0.5358	0.0784	opioid growth factor receptor-like 1
2112	LITAF	0.5358	0.1027	lipopolysaccharide-induced TNF factor
2113	KRTAP2-4	0.5358	0.1292	keratin associated protein 2-4
2114	KRT4	0.5358	0.1261	keratin 4
2115	CEACAM6	0.5358	0.0475	carcinoembryonic antigen-related cell adhesion molecule 6 (non-specific cross reacting antigen)
2116	CA5B	0.5358	0.1243	carbonic anhydrase VB, mitochondrial
2117	ABCA1	0.5358	0.1178	ATP-binding cassette, sub-family A (ABC1), member 1
2118	ZMIZ1	0.535	0.1093	zinc finger, MIZ-type containing 1
2119	TWIST2	0.535	0.0775	twist homolog 2 (Drosophila)
2120	TMEM150A	0.535	0.0859	transmembrane protein 150A
2121	PTPRM	0.535	0.1314	protein tyrosine phosphatase, receptor type, M
2122	NEBL	0.535	0.0608	nebulette
2123	C7orf50	0.535	0.1329	chromosome 7 open reading frame 50
2124	ALG14	0.535	0.1989	asparagine-linked glycosylation 14 homolog (S. cerevisiae)
2125	SPINK5	0.5342	0.0493	serine peptidase inhibitor, Kazal type 5
2126	DCUN1D4	0.5342	0.283	DCN1, defective in cullin neddylation 1, domain containing 4 (S. cerevisiae)
2127	ANGPTL2	0.5342	0.1435	angiopoietin-like 2
2128	SDCBP	0.5333	0.2082	syndecan binding protein (syntenin)
2129	KRTAP9-9	0.5333	0.0837	keratin associated protein 9-9
2130	KIAA0196	0.5333	0.2541	KIAA0196
2131	FAM149B1	0.5333	0.1739	family with sequence similarity 149, member B1
2132	EIF2D	0.5333	0.1418	eukaryotic translation initiation factor 2D
2133	ALG12	0.5333	0.1461	asparagine-linked glycosylation 12, alpha-1,6-mannosyltransferase homolog (S. cerevisiae)
2134	ZBTB8A	0.5325	0.0995	zinc finger and BTB domain containing 8A
2135	PRB4	0.5325	0.2043	proline-rich protein BstNI subfamily 4
2136	NPHS2	0.5325	0.1525	nephrosis 2, idiopathic, steroid-resistant (podocin)
2137	NPAS2	0.5325	0.0638	neuronal PAS domain protein 2
2138	CPNE3	0.5325	0.2407	copine III
2139	ZNF37BP	0.5318	0.1279	zinc finger protein 37B, pseudogene
2140	ZFX	0.5317	0.1441	zinc finger protein, X-linked
2141	NRG1	0.5317	0.0895	neuregulin 1
2142	ZDHHC15	0.5313	0.0198	zinc finger, DHHC-type containing 15
2143	PLP2	0.5308	0.1095	proteolipid protein 2 (colonic epithelium-enriched)
2144	HSD17B8	0.5308	0.0998	hydroxysteroid (17-beta) dehydrogenase 8
2145	GPR22	0.5308	0.0489	G protein-coupled receptor 22
2146	CDH6	0.5308	0.0822	cadherin 6, type 2, K-cadherin (fetal kidney)
2147	TYSND1	0.53	0.1103	trypsin domain containing 1
2148	TRIM7	0.53	0.0502	tripartite motif containing 7
2149	PTPRF	0.53	0.0729	protein tyrosine phosphatase, receptor type, F
2150	PMP22	0.53	0.1079	peripheral myelin protein 22
2151	HDAC7	0.53	0.1577	histone deacetylase 7
2152	CIB1	0.53	0.152	calcium and integrin binding 1 (calmyrin)
2153	BMPER	0.53	0.0223	BMP binding endothelial regulator
2154	APCDD1L	0.53	0.0825	adenomatosis polyposis coli down-regulated 1-like
2155	COQ4	0.5292	0.1611	coenzyme Q4 homolog (S. cerevisiae)
2156	CHP2	0.5292	0.1159	calcineurin-like EF hand protein 2
2157	ALDH7A1	0.5292	0.0512	aldehyde dehydrogenase 7 family, member A1
2158	HSD3B7	0.5288	0.1016	hydroxy-delta-5-steroid dehydrogenase, 3 beta- and steroid delta-isomerase 7
2159	GPRIN3	0.5288	0.0276	GPRIN family member 3
2160	VPS39	0.5283	0.1696	vacuolar protein sorting 39 homolog (S. cerevisiae)
2161	AFF1	0.5283	0.0947	AF4/FMR2 family, member 1
2162	TRAK2	0.5275	0.1951	trafficking protein, kinesin binding 2
2163	EEF1D	0.5275	0.1353	eukaryotic translation elongation factor 1 delta (guanine nucleotide exchange protein)
2164	ATP8B4	0.5275	0.0755	ATPase, class I, type 8B, member 4
2165	HSPA1B	0.5273	0.0356	heat shock 70kDa protein 1B
2166	SPTLC1	0.5267	0.3191	serine palmitoyltransferase, long chain base subunit 1
2167	MLANA	0.5267	0.113	melan-A
2168	MAN2C1	0.5267	0.1382	mannosidase, alpha, class 2C, member 1
2169	GGCX	0.5267	0.0758	gamma-glutamyl carboxylase
2170	CFI	0.5267	0.0712	complement factor I
2171	CLDN2	0.5263	0.0295	claudin 2
2172	ZFYVE21	0.5258	0.1179	zinc finger, FYVE domain containing 21
2173	WISP1	0.5258	0.1552	WNT1 inducible signaling pathway protein 1
2174	RARRES3	0.5258	0.1102	retinoic acid receptor responder (tazarotene induced) 3
2175	HTATIP2	0.5258	0.1168	HIV-1 Tat interactive protein 2, 30kDa
2176	ECHDC3	0.5258	0.0178	enoyl CoA hydratase domain containing 3
2177	ADIPOQ	0.5258	0.0211	adiponectin, C1Q and collagen domain containing
2178	SPTBN1	0.525	0.0846	spectrin, beta, non-erythrocytic 1
2179	SHISA4	0.525	0.1235	shisa homolog 4 (Xenopus laevis)
2180	MED11	0.525	0.1075	mediator complex subunit 11
2181	LY6D	0.525	0.1364	lymphocyte antigen 6 complex, locus D
2182	KLHL9	0.525	0.2214	kelch-like 9 (Drosophila)

2183	S100A3	0.5242	0.0861	S100 calcium binding protein A3
2184	PTK2	0.5242	0.1578	PTK2 protein tyrosine kinase 2
2185	GCNT3	0.5242	0.0461	glucosaminyl (N-acetyl) transferase 3, mucin type
2186	VGLL4	0.5233	0.0855	vestigial like 4 (Drosophila)
2187	OTOGL	0.5233	0	otogelin-like
2188	LCN2	0.5233	0.0443	lipocalin 2
2189	KLF10	0.5233	0.1447	Kruppel-like factor 10
2190	ACAN	0.5233	0.2184	aggrecan
2191	WDR69	0.5225	0.0154	WD repeat domain 69
2192	TMEM68	0.5225	0.2651	transmembrane protein 68
2193	TFPI2	0.5225	0.0368	tissue factor pathway inhibitor 2
2194	SRPR	0.5225	0.1395	signal recognition particle receptor (docking protein)
2195	PRLR	0.5225	0.1231	prolactin receptor
2196	CBLB	0.5225	0.1604	Cbl proto-oncogene, E3 ubiquitin protein ligase B
2197	ATPBD4	0.5225	0.1824	ATP binding domain 4
2198	TMEM109	0.5217	0.1573	transmembrane protein 109
2199	TMED9	0.5217	0.1389	transmembrane emp24 protein transport domain containing 9
2200	FLNA	0.5217	0.1765	filamin A, alpha
2201	EDN3	0.5217	0.1938	endothelin 3
2202	DDR1	0.5217	0.1027	discoidin domain receptor tyrosine kinase 1
2203	WDFY3-AS2	0.5213	0.0406	WDFY3 antisense RNA 2 (non-protein coding)
2204	PRRG1	0.5208	0.1011	proline rich Gla (G-carboxyglutamic acid) 1
2205	NME3	0.5208	0.1424	NME/NM23 nucleoside diphosphate kinase 3
2206	NEK9	0.5208	0.1067	NIMA (never in mitosis gene a)- related kinase 9
2207	LMAN2L	0.5208	0.0729	lectin, mannose-binding 2-like
2208	FUCA1	0.5208	0.1453	fucosidase, alpha-L- 1, tissue
2209	SLC22A13	0.52	0.1925	solute carrier family 22 (organic anion transporter), member 13
2210	SFRP2	0.52	0.0544	secreted frizzled-related protein 2
2211	GLB1L2	0.52	0.0399	galactosidase, beta 1-like 2
2212	FAM59A	0.52	0.0446	family with sequence similarity 59, member A
2213	MSTN	0.5192	0.0114	myostatin
2214	JUN	0.5192	0.0661	jun proto-oncogene
2215	CMPK1	0.5192	0.2847	cytidine monophosphate (UMP-CMP) kinase 1, cytosolic
2216	C1QTNF7	0.5188	0.0691	C1q and tumor necrosis factor related protein 7
2217	TRIM38	0.5183	0.161	tripartite motif containing 38
2218	TRPA1	0.5175	0.1025	transient receptor potential cation channel, subfamily A, member 1
2219	PDZD11	0.5175	0.1293	PDZ domain containing 11
2220	NSMCE1	0.5175	0.1698	non-SMC element 1 homolog (S. cerevisiae)
2221	LGALS8	0.5175	0.1262	lectin, galactoside-binding, soluble, 8
2222	HYAL1	0.5175	0.1006	hyaluronoglucosaminidase 1
2223	GLG1	0.5175	0.1504	golgi glycoprotein 1
2224	CREB3L1	0.5175	0.1996	cAMP responsive element binding protein 3-like 1
2225	CERS3	0.5175	0.0742	ceramide synthase 3
2226	SOCS3	0.5167	0.1338	suppressor of cytokine signaling 3
2227	NLRX1	0.5167	0.1229	NLR family member X1
2228	FAM66D	0.5167	0	family with sequence similarity 66, member D
2229	CEACAM1	0.5167	0.0976	carcinoembryonic antigen-related cell adhesion molecule 1 (biliary glycoprotein)
2230	MIR100HG	0.5163	0.0915	mir-100-let-7a-2 cluster host gene (non-protein coding)
2231	GALNTL1	0.5163	0.0648	UDP-N-acetyl-alpha-D-galactosamine:polypeptide N-acetylgalactosaminyltransferase-like 1
2232	ZBED2	0.5158	0.0236	zinc finger, BED-type containing 2
2233	SEC61A1	0.5158	0.176	Sec61 alpha 1 subunit (S. cerevisiae)
2234	RPH3AL	0.5158	0.0213	rabphilin 3A-like (without C2 domains)
2235	ENTPD5	0.5158	0.0476	ectonucleoside triphosphate diphosphohydrolase 5
2236	FIGF	0.5157	0.053	c-fos induced growth factor (vascular endothelial growth factor D)
2237	HIAT1	0.515	0.3	hippocampus abundant transcript 1
2238	DAG1	0.515	0.1019	dystroglycan 1 (dystrophin-associated glycoprotein 1)
2239	AADACL2	0.515	0.004	arylacetamide deacetylase-like 2
2240	SYDE1	0.5142	0.2665	synapse defective 1, Rho GTPase, homolog 1 (C. elegans)
2241	SULT1C2	0.5142	0.0434	sulfotransferase family, cytosolic, 1C, member 2
2242	SCARF1	0.5142	0.0592	scavenger receptor class F, member 1
2243	ITGA5	0.5142	0.1646	integrin, alpha 5 (fibronectin receptor, alpha polypeptide)
2244	ZC3H6	0.5138	0.1874	zinc finger CCCH-type containing 6
2245	PIGN	0.5133	0.2195	phosphatidylinositol glycan anchor biosynthesis, class N
2246	PHLDB1	0.5133	0.139	pleckstrin homology-like domain, family B, member 1
2247	LOC100129917	0.5133	0	uncharacterized LOC100129917
2248	ZBTB42	0.5129	0.0453	zinc finger and BTB domain containing 42
2249	ZNF148	0.5125	0.2423	zinc finger protein 148
2250	THEM4	0.5125	0.0512	thioesterase superfamily member 4
2251	SLC4A5	0.5125	0.1008	solute carrier family 4, sodium bicarbonate cotransporter, member 5
2252	RNF150	0.5125	0.047	ring finger protein 150
2253	DST	0.5125	0.132	dystonin
2254	DNASE2B	0.5125	0.0191	deoxyribonuclease II beta
2255	ANGPT1	0.5125	0.082	angiopoietin 1
2256	IFT140	0.5117	0.1116	intraflagellar transport 140 homolog (Chlamydomonas)
2257	HPGD	0.5117	0.0293	hydroxyprostaglandin dehydrogenase 15-(NAD)
2258	FAM3A	0.5117	0.1999	family with sequence similarity 3, member A
2259	CDSN	0.5117	0.2345	corneodesmosin
2260	AMT	0.5117	0.1184	aminomethyltransferase
2261	SP100	0.5108	0.1338	SP100 nuclear antigen
2262	GPRC5C	0.5108	0.1002	G protein-coupled receptor, family C, group 5, member C
2263	FUT3	0.5108	0.1594	fucosyltransferase 3 (galactoside 3(4)-L-fucosyltransferase, Lewis blood group)
2264	EFR3A	0.5108	0.3006	EFR3 homolog A (S. cerevisiae)
2265	CSDA	0.5108	0.0548	cold shock domain protein A
2266	ZDHHC2	0.51	0.1441	zinc finger, DHHC-type containing 2

2267	VPS13B	0.51	0.2001	vacuolar protein sorting 13 homolog B (yeast)
2268	SPR	0.51	0.1025	sepiapterin reductase (7,8-dihydrobiopterin:NADP+ oxidoreductase)
2269	PVRL4	0.51	0.0832	poliovirus receptor-related 4
2270	C7orf53	0.51	0.0586	chromosome 7 open reading frame 53
2271	SLK	0.5092	0.3074	STE20-like kinase
2272	SEPP1	0.5092	0.1259	selenoprotein P, plasma, 1
2273	NT5C2	0.5092	0.1647	5'-nucleotidase, cytosolic II
2274	MECOM	0.5092	0.0293	MDS1 and EVI1 complex locus
2275	DSE	0.5092	0.1812	dermatan sulfate epimerase
2276	MUM1L1	0.5088	0.0186	melanoma associated antigen (mutated) 1-like 1
2277	AEBP2	0.5088	0.2757	AE binding protein 2
2278	FGFR2	0.5083	0.0685	fibroblast growth factor receptor 2
2279	SLC39A2	0.5075	0.1218	solute carrier family 39 (zinc transporter), member 2
2280	PXN	0.5075	0.1184	paxillin
2281	MTX3	0.5075	0.1763	metaxin 3
2282	EML4	0.5075	0.149	echinoderm microtubule associated protein like 4
2283	CBR1	0.5075	0.1074	carbonyl reductase 1
2284	FMO6P	0.5073	0.209	flavin containing monooxygenase 6 pseudogene
2285	TP53TG1	0.5067	0.1151	TP53 target 1 (non-protein coding)
2286	EDEM2	0.5067	0.1314	ER degradation enhancer, mannosidase alpha-like 2
2287	CYB5R1	0.5067	0.1519	cytochrome b5 reductase 1
2288	TMEM220	0.5063	0.0221	transmembrane protein 220
2289	PTH	0.5058	0.0131	parathyroid hormone
2290	PEX11A	0.5058	0.037	peroxisomal biogenesis factor 11 alpha
2291	KCNK15	0.5058	0.0358	potassium channel, subfamily K, member 15
2292	DBT	0.5058	0.2211	dihydrolipoamide branched chain transacylase E2
2293	S100A7	0.5057	0.0116	S100 calcium binding protein A7
2294	SELP	0.505	0.1053	selectin P (granule membrane protein 140kDa, antigen CD62)
2295	PRKAA1	0.505	0.25	protein kinase, AMP-activated, alpha 1 catalytic subunit
2296	MRPL45	0.505	0.2201	mitochondrial ribosomal protein L45
2297	CYP4V2	0.505	0.1207	cytochrome P450, family 4, subfamily V, polypeptide 2
2298	PLBD1	0.5042	0.0596	phospholipase B domain containing 1
2299	LIMCH1	0.5042	0.0929	LIM and calponin homology domains 1
2300	GOLIM4	0.5042	0.0645	golgi integral membrane protein 4
2301	CRCP	0.5042	0.0487	CGRP receptor component
2302	C4orf19	0.5042	0.0453	chromosome 4 open reading frame 19
2303	ITSN2	0.5033	0.1331	intersectin 2
2304	HYAL2	0.5033	0.1296	hyaluronoglucosaminidase 2
2305	CCNG2	0.5033	0.2285	cyclin G2
2306	C12orf49	0.5033	0.0585	chromosome 12 open reading frame 49
2307	ABHD5	0.5033	0.1258	abhydrolase domain containing 5
2308	ABCA6	0.5033	0.0839	ATP-binding cassette, sub-family A (ABC1), member 6
2309	WISP2	0.5025	0.1519	WNT1 inducible signaling pathway protein 2
2310	TNFRSF10A	0.5025	0.0519	tumor necrosis factor receptor superfamily, member 10a
2311	SSR1	0.5025	0.3136	signal sequence receptor, alpha
2312	PLA2G7	0.5025	0.0501	phospholipase A2, group VII (platelet-activating factor acetylhydrolase, plasma)
2313	KIAA0825	0.5025	0.0215	KIAA0825
2314	ATP6AP2	0.5025	0.289	ATPase, H+ transporting, lysosomal accessory protein 2
2315	TXN	0.5017	0.1972	thioredoxin
2316	SWAP70	0.5017	0.2576	SWAP switching B-cell complex 70kDa subunit
2317	PRRG2	0.5017	0.1763	proline rich Gla (G-carboxyglutamic acid) 2
2318	DMBT1	0.5017	0.151	deleted in malignant brain tumors 1
2319	MT1E	0.5014	0.031	metallothionein 1E
2320	PCDHGB7	0.5013	0.0162	protocadherin gamma subfamily B, 7
2321	MAP1LC3A	0.5013	0.1269	microtubule-associated protein 1 light chain 3 alpha
2322	CYP4X1	0.5013	0.0273	cytochrome P450, family 4, subfamily X, polypeptide 1
2323	SEL1L	0.5008	0.1935	sel-1 suppressor of lin-12-like (C. elegans)
2324	NR3C2	0.5008	0.0996	nuclear receptor subfamily 3, group C, member 2
2325	ECI1	0.5008	0.1561	enoyl-CoA delta isomerase 1
2326	CBR3	0.5008	0.0321	carbonyl reductase 3
2327	TBCK	0.5	0.2486	TBC1 domain containing kinase
2328	PRKAG1	0.5	0.1419	protein kinase, AMP-activated, gamma 1 non-catalytic subunit
2329	OSBP	0.5	0.1154	oxysterol binding protein
2330	KLF2	0.5	0.0839	Kruppel-like factor 2 (lung)
2331	ARNT	0.5	0.1003	aryl hydrocarbon receptor nuclear translocator
2332	HMGCS2	0.4992	0.089	3-hydroxy-3-methylglutaryl-CoA synthase 2 (mitochondrial)
2333	EPB41L4B	0.4992	0.05	erythrocyte membrane protein band 4.1 like 4B
2334	CRTC3	0.4992	0.1526	CREB regulated transcription coactivator 3
2335	CHPF2	0.4992	0.1655	chondroitin polymerizing factor 2
2336	SLC45A4	0.4988	0.0375	solute carrier family 45, member 4
2337	TNFSF12	0.4983	0.1653	tumor necrosis factor (ligand) superfamily, member 12
2338	STON1	0.4983	0.1203	stonin 1
2339	MYH2	0.4983	0.0267	myosin, heavy chain 2, skeletal muscle, adult
2340	LOC100287841	0.4983	0	zinc finger protein ZnFP12-like
2341	ZNF396	0.4975	0.0547	zinc finger protein 396
2342	UXS1	0.4975	0.1541	UDP-glucuronate decarboxylase 1
2343	TRIOBP	0.4975	0.1155	TRIO and F-actin binding protein
2344	TMEM181	0.4975	0.1944	transmembrane protein 181
2345	ITGA8	0.4975	0.1108	integrin, alpha 8
2346	ILK	0.4975	0.1878	integrin-linked kinase
2347	LOC150381	0.4971	0	uncharacterized LOC150381
2348	ZNF192	0.4967	0.105	zinc finger protein 192
2349	TMCO1	0.4967	0.2947	transmembrane and coiled-coil domains 1
2350	SOX9	0.4967	0.0359	SRY (sex determining region Y)-box 9

2351	RARRES2	0.4967	0.088	retinoic acid receptor responder (tazarotene induced) 2
2352	FKBP11	0.4967	0.0571	FK506 binding protein 11, 19 kDa
2353	ACOX3	0.4967	0.0479	acyl-CoA oxidase 3, pristanoyl
2354	DPY19L2	0.4963	0.0279	dpy-19-like 2 (C. elegans)
2355	DMRT3	0.4963	0.0109	doublesex and mab-3 related transcription factor 3
2356	BLID	0.4963	0.0179	BH3-like motif containing, cell death inducer
2357	WDR11	0.4958	0.3279	WD repeat domain 11
2358	PTPRK	0.4958	0.1367	protein tyrosine phosphatase, receptor type, K
2359	PPAP2C	0.4958	0.0562	phosphatidic acid phosphatase type 2C
2360	KLF9	0.4958	0.1064	Kruppel-like factor 9
2361	EYA2	0.4958	0.0192	eyes absent homolog 2 (Drosophila)
2362	EDEM3	0.4958	0.2848	ER degradation enhancer, mannosidase alpha-like 3
2363	TAGLN2	0.495	0.1549	transgelin 2
2364	SERTAD1	0.495	0.1207	SERTA domain containing 1
2365	PLTP	0.495	0.0898	phospholipid transfer protein
2366	NKX2-1	0.495	0.1593	NK2 homeobox 1
2367	ID3	0.495	0.0434	inhibitor of DNA binding 3, dominant negative helix-loop-helix protein
2368	GPX2	0.495	0.115	glutathione peroxidase 2 (gastrointestinal)
2369	COLEC11	0.495	0.0424	collectin sub-family member 11
2370	CCDC85A	0.495	0.0567	coiled-coil domain containing 85A
2371	ARHGAP42	0.4943	0.0944	Rho GTPase activating protein 42
2372	TRAM2	0.4942	0.0951	translocation associated membrane protein 2
2373	PPP1R12B	0.4942	0.1103	protein phosphatase 1, regulatory subunit 12B
2374	PLCB4	0.4942	0.0421	phospholipase C, beta 4
2375	GRB7	0.4942	0.0893	growth factor receptor-bound protein 7
2376	DDX60	0.4942	0.1905	DEAD (Asp-Glu-Ala-Asp) box polypeptide 60
2377	TNRC18	0.4938	0.0876	trinucleotide repeat containing 18
2378	OCIAD2	0.4938	0.0689	OCIA domain containing 2
2379	IFI44L	0.4933	0.0916	interferon-induced protein 44-like
2380	SLC4A4	0.4925	0.1116	solute carrier family 4, sodium bicarbonate cotransporter, member 4
2381	RGCC	0.4925	0.053	regulator of cell cycle
2382	MLL5	0.4925	0.2276	myeloid/lymphoid or mixed-lineage leukemia 5 (trithorax homolog, Drosophila)
2383	HOXA13	0.4925	0.0059	homeobox A13
2384	HCG4	0.4925	0.0077	HLA complex group 4 (non-protein coding)
2385	ESYT3	0.4925	0.0369	extended synaptotagmin-like protein 3
2386	CLN3	0.4925	0.1663	ceroid-lipofuscinosis, neuronal 3
2387	HOXA3	0.4917	0.0633	homeobox A3
2388	FSTL4	0.4917	0.2314	follistatin-like 4
2389	DNASE2	0.4917	0.1379	deoxyribonuclease II, lysosomal
2390	LOC643085	0.4914	0.0044	uncharacterized LOC643085
2391	ZBED3	0.4913	0.0472	zinc finger, BED-type containing 3
2392	MCL1	0.4908	0.1808	myeloid cell leukemia sequence 1 (BCL2-related)
2393	DENND3	0.4908	0.1416	DENN/MADD domain containing 3
2394	ANGPTL4	0.4908	0.124	angiopoietin-like 4
2395	ALG5	0.4908	0.264	asparagine-linked glycosylation 5, dolichyl-phosphate beta-glucosyltransferase homolog (S. cerevisiae)
2396	LMCD1	0.49	0.072	LIM and cysteine-rich domains 1
2397	FAM108C1	0.49	0.0307	family with sequence similarity 108, member C1
2398	UBXN4	0.4892	0.3108	UBX domain protein 4
2399	RAB2A	0.4892	0.1059	RAB2A, member RAS oncogene family
2400	KRT6A	0.4892	0.056	keratin 6A
2401	DCAF17	0.4892	0.2907	DDB1 and CUL4 associated factor 17
2402	CTSA	0.4892	0.1977	cathepsin A
2403	AGTR1	0.4892	0.0868	angiotensin II receptor, type 1
2404	C11orf74	0.4888	0.1506	chromosome 11 open reading frame 74
2405	ZC3H12A	0.4883	0.1065	zinc finger CCCH-type containing 12A
2406	SLC11A2	0.4883	0.0735	solute carrier family 11 (proton-coupled divalent metal ion transporters), member 2
2407	NR4A3	0.4883	0.1188	nuclear receptor subfamily 4, group A, member 3
2408	LRP1B	0.4883	0.0238	low density lipoprotein receptor-related protein 1B
2409	LAMP2	0.4883	0.2753	lysosomal-associated membrane protein 2
2410	C14orf101	0.4883	0.2152	chromosome 14 open reading frame 101
2411	BHLHE41	0.4883	0.0435	basic helix-loop-helix family, member e41
2412	TYRP1	0.4875	0.0269	tyrosinase-related protein 1
2413	OGT	0.4875	0.211	O-linked N-acetylglucosamine (GlcNAc) transferase
2414	MBD6	0.4875	0.1145	methyl-CpG binding domain protein 6
2415	C7orf23	0.4875	0.208	chromosome 7 open reading frame 23
2416	LUZP1	0.4867	0.0778	leucine zipper protein 1
2417	GALNT14	0.4867	0.026	UDP-N-acetyl-alpha-D-galactosamine:polypeptide N-acetylgalactosaminyltransferase 14 (GalNAc-T14)
2418	SEC14L1P1	0.4864	0.0667	SEC14-like 1 pseudogene 1
2419	BLOC1S6	0.4863	0.3001	biogenesis of lysosomal organelles complex-1, subunit 5, pallidin
2420	SUCLG2	0.4858	0.2315	succinate-CoA ligase, GDP-forming, beta subunit
2421	SDF4	0.4858	0.1961	stromal cell derived factor 4
2422	RIPK4	0.4858	0.0594	receptor-interacting serine-threonine kinase 4
2423	MFSD10	0.4858	0.1603	major facilitator superfamily domain containing 10
2424	TLR5	0.485	0.0777	toll-like receptor 5
2425	ST6GAL2	0.485	0.0232	ST6 beta-galactosamide alpha-2,6-sialyltranferase 2
2426	PRB3	0.485	0.1649	proline-rich protein BstNI subfamily 3
2427	IMPA2	0.485	0.1086	inositol(myo)-1(or 4)-monophosphatase 2
2428	ETS2	0.485	0.0846	v-ets erythroblastosis virus E26 oncogene homolog 2 (avian)
2429	CLIC5	0.485	0.0727	chloride intracellular channel 5
2430	C10orf54	0.485	0.1582	chromosome 10 open reading frame 54
2431	ZNF880	0.4843	0.0352	zinc finger protein 880
2432	XPC	0.4842	0.1362	xeroderma pigmentosum, complementation group C
2433	PLEKHA1	0.4842	0.165	pleckstrin homology domain containing, family A (phosphoinositide binding specific) member 1
2434	NUDT13	0.4842	0.0493	nudix (nucleoside diphosphate linked moiety X)-type motif 13

2435	HERC3	0.4842	0.121	HECT and RLD domain containing E3 ubiquitin protein ligase 3
2436	EGR2	0.4842	0.067	early growth response 2
2437	COPA	0.4842	0.1823	coatomer protein complex, subunit alpha
2438	FAM82A1	0.4837	0.0597	family with sequence similarity 82, member A1
2439	MUC1	0.4833	0.1098	mucin 1, cell surface associated
2440	MN1	0.4833	0.0546	meningioma (disrupted in balanced translocation) 1
2441	PTGER4	0.4825	0.0769	prostaglandin E receptor 4 (subtype EP4)
2442	MTAP	0.4825	0.1298	methylthioadenosine phosphorylase
2443	CRYZ	0.4825	0.261	crystallin, zeta (quinone reductase)
2444	CPT1A	0.4825	0.0219	carnitine palmitoyltransferase 1A (liver)
2445	BHMT2	0.4825	0.0573	betaine--homocysteine S-methyltransferase 2
2446	RCOR3	0.4817	0.2764	REST corepressor 3
2447	PART1	0.4817	0.1588	prostate androgen-regulated transcript 1 (non-protein coding)
2448	LRRC19	0.4817	0.0098	leucine rich repeat containing 19
2449	ABCA11P	0.4817	0.0855	ATP-binding cassette, sub-family A (ABC1), member 11, pseudogene
2450	ZNF780B	0.4808	0.0727	zinc finger protein 780B
2451	TGM1	0.4808	0.0914	transglutaminase 1 (K polypeptide epidermal type I, protein-glutamine-gamma-glutamyltransferase)
2452	SEC24D	0.4808	0.256	SEC24 family, member D (S. cerevisiae)
2453	NR1H3	0.4808	0.0741	nuclear receptor subfamily 1, group H, member 3
2454	WNT11	0.48	0.2228	wingless-type MMTV integration site family, member 11
2455	WDSUB1	0.48	0.2461	WD repeat, sterile alpha motif and U-box domain containing 1
2456	RBM3	0.48	0.2217	RNA binding motif (RNP1, RRM) protein 3
2457	PRCP	0.48	0.1836	prolylcarboxypeptidase (angiotensinase C)
2458	MPPE1	0.48	0.0907	metallophosphoesterase 1
2459	MCAM	0.48	0.098	melanoma cell adhesion molecule
2460	MAP4K5	0.48	0.3213	mitogen-activated protein kinase kinase kinase kinase 5
2461	HMGCL	0.48	0.1594	3-hydroxymethyl-3-methylglutaryl-CoA lyase
2462	CBLC	0.48	0.1055	Cbl proto-oncogene, E3 ubiquitin protein ligase C
2463	WDR5B	0.4792	0.1345	WD repeat domain 5B
2464	WDR45	0.4792	0.1517	WD repeat domain 45
2465	SSR2	0.4792	0.1705	signal sequence receptor, beta (translocon-associated protein beta)
2466	SLC22A2	0.4792	0.1702	solute carrier family 22 (organic cation transporter), member 2
2467	ZFAND2B	0.4787	0.1378	zinc finger, AN1-type domain 2B
2468	HIATL1	0.4787	0.2775	hippocampus abundant transcript-like 1
2469	SUOX	0.4783	0.107	sulfite oxidase
2470	SH2D3A	0.4783	0.0795	SH2 domain containing 3A
2471	SGCD	0.4783	0.1918	sarcoglycan, delta (35kDa dystrophin-associated glycoprotein)
2472	ZFYVE9	0.4775	0.0962	zinc finger, FYVE domain containing 9
2473	UBE3B	0.4775	0.1402	ubiquitin protein ligase E3B
2474	SLC16A14	0.4775	0.0282	solute carrier family 16, member 14 (monocarboxylic acid transporter 14)
2475	RNF149	0.4775	0.1788	ring finger protein 149
2476	NUMA1	0.4775	0.1899	nuclear mitotic apparatus protein 1
2477	NAPRT1	0.4775	0.0998	nicotinate phosphoribosyltransferase domain containing 1
2478	CLIC6	0.4775	0.0354	chloride intracellular channel 6
2479	ADD3	0.4775	0.237	adducin 3 (gamma)
2480	SRGN	0.4767	0.1563	serglycin
2481	LMOD1	0.4767	0.1785	leiomodin 1 (smooth muscle)
2482	ID1	0.4767	0.04	inhibitor of DNA binding 1, dominant negative helix-loop-helix protein
2483	GALNS	0.4767	0.1024	galactosamine (N-acetyl)-6-sulfate sulfatase
2484	BBS4	0.4767	0.1533	Bardet-Biedl syndrome 4
2485	ZNF655	0.4762	0.1678	zinc finger protein 655
2486	SLC30A1	0.4762	0.1159	solute carrier family 30 (zinc transporter), member 1
2487	LCA5	0.4762	0.1424	Leber congenital amaurosis 5
2488	FAM167A	0.4762	0.011	family with sequence similarity 167, member A
2489	PIAS3	0.4758	0.1137	protein inhibitor of activated STAT, 3
2490	PI3	0.4758	0.1112	peptidase inhibitor 3, skin-derived
2491	ELF1	0.4758	0.244	E74-like factor 1 (ets domain transcription factor)
2492	CRAT	0.4758	0.1372	carnitine O-acetyltransferase
2493	ALPK3	0.4758	0.0761	alpha-kinase 3
2494	TMEM98	0.475	0.0577	transmembrane protein 98
2495	SLC48A1	0.475	0.1206	solute carrier family 48 (heme transporter), member 1
2496	PRKAG2	0.475	0.0546	protein kinase, AMP-activated, gamma 2 non-catalytic subunit
2497	HOXC10	0.475	0.0295	homeobox C10
2498	FUT11	0.475	0.0597	fucosyltransferase 11 (alpha (1,3) fucosyltransferase)
2499	CRYGN	0.475	0.1278	crystallin, gamma N
2500	CHMP2A	0.475	0.198	charged multivesicular body protein 2A
2501	ATP6V1C2	0.475	0.0111	ATPase, H+ transporting, lysosomal 42kDa, V1 subunit C2
2502	PIK3IP1	0.4742	0.1655	phosphoinositide-3-kinase interacting protein 1
2503	KRT33B	0.4742	0.1627	keratin 33B
2504	ATHL1	0.4742	0.1294	ATH1, acid trehalase-like 1 (yeast)
2505	QPCT	0.4733	0.0812	glutaminyl-peptide cyclotransferase
2506	MYO15B	0.4733	0.188	myosin XVB pseudogene
2507	MFSD11	0.4733	0.0799	major facilitator superfamily domain containing 11
2508	ME3	0.4733	0.0429	malic enzyme 3, NADP(+)-dependent, mitochondrial
2509	ELOVL1	0.4733	0.2162	ELOVL fatty acid elongase 1
2510	C5orf15	0.4733	0.2432	chromosome 5 open reading frame 15
2511	AMACR	0.4729	0.0418	alpha-methylacyl-CoA racemase
2512	ZNF229	0.4725	0.0138	zinc finger protein 229
2513	UGT1A6	0.4725	0.0264	UDP glucuronosyltransferase 1 family, polypeptide A6
2514	TLR6	0.4725	0.1233	toll-like receptor 6
2515	SERPINB2	0.4725	0.0578	serpin peptidase inhibitor, clade B (ovalbumin), member 2
2516	MAPK13	0.4725	0.0502	mitogen-activated protein kinase 13
2517	FRK	0.4725	0.0433	fyn-related kinase
2518	RAB22A	0.4717	0.2829	RAB22A, member RAS oncogene family

2519	LPAR1	0.4717	0.0995	lysophosphatidic acid receptor 1
2520	KRT23	0.4717	0.0525	keratin 23 (histone deacetylase inducible)
2521	IL36G	0.4717	0.0534	interleukin 36, gamma
2522	GABARAPL1	0.4717	0.1407	GABA(A) receptor-associated protein like 1
2523	EMILIN3	0.4712	0.0822	elastin microfibril interfacer 3
2524	PIGO	0.4708	0.1239	phosphatidylinositol glycan anchor biosynthesis, class O
2525	MOCS2	0.4708	0.2815	molybdenum cofactor synthesis 2
2526	MDM2	0.4708	0.0795	Mdm2, p53 E3 ubiquitin protein ligase homolog (mouse)
2527	ACTN1	0.4708	0.1669	actinin, alpha 1
2528	ACP2	0.4708	0.1482	acid phosphatase 2, lysosomal
2529	RIPK1	0.47	0.1628	receptor (TNFRSF)-interacting serine-threonine kinase 1
2530	PF4V1	0.47	0.0081	platelet factor 4 variant 1
2531	EXOC6	0.47	0.2353	exocyst complex component 6
2532	PARD6B	0.4692	0.0596	par-6 partitioning defective 6 homolog beta (C. elegans)
2533	LPCAT3	0.4692	0.1098	lysophosphatidylcholine acyltransferase 3
2534	ITFG2	0.4692	0.0782	integrin alpha FG-GAP repeat containing 2
2535	INO80D	0.4692	0.1506	INO80 complex subunit D
2536	F8	0.4692	0.098	coagulation factor VIII, procoagulant component
2537	C14orf105	0.4692	0.0077	chromosome 14 open reading frame 105
2538	NUFIP2	0.4688	0.2818	nuclear fragile X mental retardation protein interacting protein 2
2539	LINC00085	0.4688	0.0494	long intergenic non-protein coding RNA 85
2540	C14orf149	0.4688	0.0922	chromosome 14 open reading frame 149
2541	SBSN	0.4686	0.1152	suprabasin
2542	PLD2	0.4683	0.2412	phospholipase D2
2543	MAT2B	0.4683	0.3038	methionine adenosyltransferase II, beta
2544	APOD	0.4683	0.0968	apolipoprotein D
2545	ADM	0.4683	0.0841	adrenomedullin
2546	USP51	0.4675	0.03	ubiquitin specific peptidase 51
2547	TSLP	0.4675	0.0118	thymic stromal lymphopoietin
2548	TM4SF20	0.4675	0.0429	transmembrane 4 L six family member 20
2549	RASA1	0.4675	0.3335	RAS p21 protein activator (GTPase activating protein) 1
2550	NHLRC3	0.4675	0.2366	NHL repeat containing 3
2551	IGSF10	0.4675	0.0505	immunoglobulin superfamily, member 10
2552	LOC145820	0.4671	0.1043	uncharacterized LOC145820
2553	S100A5	0.4667	0.1772	S100 calcium binding protein A5
2554	LTF	0.4667	0.0428	lactotransferrin
2555	COL7A1	0.4667	0.1526	collagen, type VII, alpha 1
2556	C13orf41	0.4667	0	chromosome 13 open reading frame 41
2557	MIOX	0.4663	0.0969	myo-inositol oxygenase
2558	MAGI3	0.4663	0.0905	membrane associated guanylate kinase, WW and PDZ domain containing 3
2559	EIF2S3	0.4663	0.1902	eukaryotic translation initiation factor 2, subunit 3 gamma, 52kDa
2560	ARRDC2	0.4663	0.1151	arrestin domain containing 2
2561	TSPAN9	0.4658	0.1401	tetraspanin 9
2562	SLITRK5	0.4658	0.0279	SLIT and NTRK-like family, member 5
2563	SLC8A1	0.4658	0.1361	solute carrier family 8 (sodium/calcium exchanger), member 1
2564	VPS13D	0.465	0.1574	vacuolar protein sorting 13 homolog D (S. cerevisiae)
2565	UBE2H	0.465	0.1313	ubiquitin-conjugating enzyme E2H
2566	TMEM185B	0.465	0.1415	transmembrane protein 185B
2567	NFKB1	0.465	0.1369	nuclear factor of kappa light polypeptide gene enhancer in B-cells 1
2568	ARFGEF1	0.465	0.3065	ADP-ribosylation factor guanine nucleotide-exchange factor 1 (brefeldin A-inhibited)
2569	REXO2	0.4642	0.2064	REX2, RNA exonuclease 2 homolog (S. cerevisiae)
2570	MYO1B	0.4642	0.1317	myosin IB
2571	MAF	0.4642	0.0691	v-maf musculoaponeurotic fibrosarcoma oncogene homolog (avian)
2572	LGALS4	0.4642	0.0761	lectin, galactoside-binding, soluble, 4
2573	DHRS7	0.4642	0.2143	dehydrogenase/reductase (SDR family) member 7
2574	CUL4A	0.4642	0.3332	cullin 4A
2575	ARMCX5	0.4642	0.2894	armadillo repeat containing, X-linked 5
2576	ZFHX3	0.4633	0.0957	zinc finger homeobox 3
2577	VAMP3	0.4633	0.2411	vesicle-associated membrane protein 3 (cellubrevin)
2578	FOXO1	0.4633	0.0857	forkhead box O1
2579	CDC73	0.4633	0.3463	cell division cycle 73, Paf1/RNA polymerase II complex component, homolog (S. cerevisiae)
2580	ANXA7	0.4633	0.3128	annexin A7
2581	UTP23	0.4625	0.2637	UTP23, small subunit (SSU) processome component, homolog (yeast)
2582	TEAD2	0.4625	0.1191	TEA domain family member 2
2583	RILPL1	0.4625	0.0999	Rab interacting lysosomal protein-like 1
2584	PODN	0.4625	0.1584	podocan
2585	PLS1	0.4625	0.1438	plastin 1
2586	PARP14	0.4625	0.1556	poly (ADP-ribose) polymerase family, member 14
2587	ITGA9	0.4625	0.1106	integrin, alpha 9
2588	CNKSR1	0.4625	0.0641	connector enhancer of kinase suppressor of Ras 1
2589	ATF6B	0.4625	0.159	activating transcription factor 6 beta
2590	TRIM8	0.4617	0.1682	tripartite motif containing 8
2591	TMEM45A	0.4617	0.1169	transmembrane protein 45A
2592	SCP2	0.4617	0.3244	sterol carrier protein 2
2593	MYO9A	0.4617	0.1338	myosin IXA
2594	ARL14	0.4617	0.0246	ADP-ribosylation factor-like 14
2595	INO80	0.4613	0.1582	INO80 homolog (S. cerevisiae)
2596	C17orf97	0.4613	0.0321	chromosome 17 open reading frame 97
2597	SSH1	0.4611	0.0972	slingshot homolog 1 (Drosophila)
2598	RUNX2	0.4608	0.1359	runt-related transcription factor 2
2599	PTK6	0.4608	0.1711	PTK6 protein tyrosine kinase 6
2600	PTGS1	0.4608	0.0798	prostaglandin-endoperoxide synthase 1 (prostaglandin G/H synthase and cyclooxygenase)
2601	NEU1	0.4608	0.1723	sialidase 1 (lysosomal sialidase)
2602	GFRA1	0.4608	0.0648	GDNF family receptor alpha 1

2603	EPYC	0.4608	0.0224	epiphycan
2604	PTPN3	0.46	0.0547	protein tyrosine phosphatase, non-receptor type 3
2605	PTK7	0.46	0.104	PTK7 protein tyrosine kinase 7
2606	MYH11	0.46	0.1837	myosin, heavy chain 11, smooth muscle
2607	DIS3	0.46	0.2926	DIS3 mitotic control homolog (S. cerevisiae)
2608	DHRS12	0.46	0.1765	dehydrogenase/reductase (SDR family) member 12
2609	CXorf26	0.46	0.279	chromosome X open reading frame 26
2610	ADI1	0.46	0.1822	acireductone dioxygenase 1
2611	WIPI1	0.4592	0.1605	WD repeat domain, phosphoinositide interacting 1
2612	PTGS2	0.4592	0.1028	prostaglandin-endoperoxide synthase 2 (prostaglandin G/H synthase and cyclooxygenase)
2613	PSMB8	0.4592	0.1796	proteasome (prosome, macropain) subunit, beta type, 8 (large multifunctional peptidase 7)
2614	MPG	0.4592	0.1704	N-methylpurine-DNA glycosylase
2615	CRABP1	0.4592	0.0391	cellular retinoic acid binding protein 1
2616	PPP1R16A	0.4588	0.1299	protein phosphatase 1, regulatory subunit 16A
2617	SERPINB7	0.4583	0.0656	serpin peptidase inhibitor, clade B (ovalbumin), member 7
2618	GCLM	0.4583	0.2128	glutamate-cysteine ligase, modifier subunit
2619	CTNNAL1	0.4583	0.1934	catenin (cadherin-associated protein), alpha-like 1
2620	ZNF334	0.4575	0.0489	zinc finger protein 334
2621	RUFY1	0.4575	0.1577	RUN and FYVE domain containing 1
2622	MSH3	0.4575	0.2546	mutS homolog 3 (E. coli)
2623	CNN3	0.4575	0.1297	calponin 3, acidic
2624	RPE	0.4567	0.3324	ribulose-5-phosphate-3-epimerase
2625	HDHD3	0.4567	0.1575	haloacid dehalogenase-like hydrolase domain containing 3
2626	ERGIC3	0.4567	0.2031	ERGIC and golgi 3
2627	ARHGAP5	0.4567	0.3115	Rho GTPase activating protein 5
2628	SRA1	0.4563	0.1579	steroid receptor RNA activator 1
2629	KRTCAP3	0.4563	0.0449	keratinocyte associated protein 3
2630	FAM175A	0.4563	0.2886	family with sequence similarity 175, member A
2631	CYP1B1-AS1	0.4563	0.0123	CYP1B1 antisense RNA 1 (non-protein coding)
2632	CTHRC1	0.4563	0.0831	collagen triple helix repeat containing 1
2633	ANKRD13A	0.4563	0.1734	ankyrin repeat domain 13A
2634	SPG20	0.4558	0.2597	spastic paraplegia 20 (Troyer syndrome)
2635	LPL	0.4558	0.0395	lipoprotein lipase
2636	ACVRL1	0.4558	0.2167	activin A receptor type II-like 1
2637	ZNF462	0.455	0.0863	zinc finger protein 462
2638	SMOC2	0.455	0.0472	SPARC related modular calcium binding 2
2639	PCDHB16	0.455	0.0511	protocadherin beta 16
2640	P2RY2	0.455	0.0756	purinergic receptor P2Y, G-protein coupled, 2
2641	ITFG3	0.455	0.1816	integrin alpha FG-GAP repeat containing 3
2642	HS3ST3A1	0.455	0.0441	heparan sulfate (glucosamine) 3-O-sulfotransferase 3A1
2643	PAPOLG	0.4542	0.2131	poly(A) polymerase gamma
2644	AMELY	0.4542	0.0489	amelogenin, Y-linked
2645	A2M	0.4542	0.1115	alpha-2-macroglobulin
2646	RHBDD1	0.4538	0.0813	rhomboid domain containing 1
2647	DNAJC14	0.4538	0.1844	DnaJ (Hsp40) homolog, subfamily C, member 14
2648	CNPY4	0.4538	0.0977	canopy 4 homolog (zebrafish)
2649	TOR4A	0.4533	0.0947	torsin family 4, member A
2650	SLC12A7	0.4533	0.0603	solute carrier family 12 (potassium/chloride transporters), member 7
2651	PON3	0.4533	0.0522	paraoxonase 3
2652	ADAMTS2	0.4533	0.2425	ADAM metallopeptidase with thrombospondin type 1 motif, 2
2653	TMEM65	0.4525	0.1625	transmembrane protein 65
2654	TIGD6	0.4525	0.0367	tigger transposable element derived 6
2655	ORMDL3	0.4525	0.086	ORM1-like 3 (S. cerevisiae)
2656	KRT38	0.4525	0.1754	keratin 38
2657	HOXA10	0.4525	0.0109	homeobox A10
2658	FST	0.4525	0.0716	follistatin
2659	TUBAL3	0.4517	0.0473	tubulin, alpha-like 3
2660	SEMA3E	0.4517	0.0227	sema domain, immunoglobulin domain (Ig), short basic domain, secreted, (semaphorin) 3E
2661	PTPRG	0.4517	0.0954	protein tyrosine phosphatase, receptor type, G
2662	MYL2	0.4517	0.0494	myosin, light chain 2, regulatory, cardiac, slow
2663	COLEC12	0.4517	0.0998	collectin sub-family member 12
2664	SYTL4	0.4512	0.0423	synaptotagmin-like 4
2665	IKBIP	0.4512	0.2314	IKBKB interacting protein
2666	CAMKK1	0.4512	0.1674	calcium/calmodulin-dependent protein kinase kinase 1, alpha
2667	TAS2R13	0.4508	0.0769	taste receptor, type 2, member 13
2668	LLGL2	0.4508	0.0803	lethal giant larvae homolog 2 (Drosophila)
2669	ATP13A3	0.4508	0.2673	ATPase type 13A3
2670	TSHZ2	0.45	0.0642	teashirt zinc finger homeobox 2
2671	SMTN	0.45	0.131	smoothelin
2672	SLC9A3	0.45	0.1611	solute carrier family 9, subfamily A (NHE3, cation proton antiporter 3), member 3
2673	SLC26A2	0.45	0.1912	solute carrier family 26 (sulfate transporter), member 2
2674	GRAMD2	0.45	0.0249	GRAM domain containing 2
2675	ACOT9	0.45	0.1792	acyl-CoA thioesterase 9
2676	SMAD4	0.4492	0.3291	SMAD family member 4
2677	PXDN	0.4492	0.1053	peroxidasin homolog (Drosophila)
2678	PBLD	0.4492	0.0998	phenazine biosynthesis-like protein domain containing
2679	GLI1	0.4492	0.1859	GLI family zinc finger 1
2680	C17orf101	0.4492	0.1028	chromosome 17 open reading frame 101
2681	BCAP31	0.4492	0.2101	B-cell receptor-associated protein 31
2682	ZNF664	0.4487	0.2779	zinc finger protein 664
2683	PEX11G	0.4487	0.1678	peroxisomal biogenesis factor 11 gamma
2684	PCDH20	0.4487	0.0185	protocadherin 20
2685	OTUD1	0.4487	0.1432	OTU domain containing 1
2686	KIF1C	0.4483	0.1511	kinesin family member 1C

2687	TBC1D10A	0.4475	0.1609	TBC1 domain family, member 10A
2688	SMAD7	0.4475	0.1065	SMAD family member 7
2689	PHLDB2	0.4475	0.1391	pleckstrin homology-like domain, family B, member 2
2690	PDCD1LG2	0.4475	0.1214	programmed cell death 1 ligand 2
2691	NOXA1	0.4475	0.1956	NADPH oxidase activator 1
2692	LRP5L	0.4475	0.0425	low density lipoprotein receptor-related protein 5-like
2693	HERC4	0.4475	0.2915	HECT and RLD domain containing E3 ubiquitin protein ligase 4
2694	CEACAM5	0.4475	0.0786	carcinoembryonic antigen-related cell adhesion molecule 5
2695	ARMCX1	0.4475	0.1465	armadillo repeat containing, X-linked 1
2696	STAT3	0.4467	0.1904	signal transducer and activator of transcription 3 (acute-phase response factor)
2697	KIT	0.4467	0.0538	v-kit Hardy-Zuckerman 4 feline sarcoma viral oncogene homolog
2698	GPR4	0.4467	0.2692	G protein-coupled receptor 4
2699	DLG5	0.4467	0.1062	discs, large homolog 5 (Drosophila)
2700	UVSSA	0.4462	0.0668	UV-stimulated scaffold protein A
2701	TINAG	0.4462	0.0817	tubulointerstitial nephritis antigen
2702	NSUN7	0.4462	0.0475	NOP2/Sun domain family, member 7
2703	BCL9L	0.4462	0.1301	B-cell CLL/lymphoma 9-like
2704	SPG7	0.4458	0.1761	spastic paraplegia 7 (pure and complicated autosomal recessive)
2705	KRT86	0.4458	0.0887	keratin 86
2706	HOOK2	0.4458	0.1017	hook homolog 2 (Drosophila)
2707	CLOCK	0.4458	0.1486	clock homolog (mouse)
2708	CLDN7	0.4458	0.0815	claudin 7
2709	CCL27	0.4458	0.1313	chemokine (C-C motif) ligand 27
2710	TMEM45B	0.445	0.0465	transmembrane protein 45B
2711	SLC16A9	0.445	0.0254	solute carrier family 16, member 9 (monocarboxylic acid transporter 9)
2712	SAMD12	0.445	0.0226	sterile alpha motif domain containing 12
2713	RNF217	0.445	0.0752	ring finger protein 217
2714	NET1	0.445	0.2134	neuroepithelial cell transforming 1
2715	LOC100130776	0.445	0.1031	uncharacterized LOC100130776
2716	IER2	0.445	0.0962	immediate early response 2
2717	HSP90B1	0.445	0.2559	heat shock protein 90kDa beta (Grp94), member 1
2718	FZD8	0.445	0.0555	frizzled family receptor 8
2719	CFTR	0.445	0.1055	cystic fibrosis transmembrane conductance regulator (ATP-binding cassette sub-family C, member 7)
2720	C12orf28	0.445	0.0217	chromosome 12 open reading frame 28
2721	ANKIB1	0.445	0.3231	ankyrin repeat and IBR domain containing 1
2722	TFAP2B	0.4442	0.1651	transcription factor AP-2 beta (activating enhancer binding protein 2 beta)
2723	SS18	0.4442	0.2024	synovial sarcoma translocation, chromosome 18
2724	PLXNB1	0.4433	0.1877	plexin B1
2725	PARM1	0.4433	0.0654	prostate androgen-regulated mucin-like protein 1
2726	LEPREL2	0.4433	0.1729	leprecan-like 2
2727	FGFBP1	0.4433	0.1439	fibroblast growth factor binding protein 1
2728	AP4E1	0.4433	0.1789	adaptor-related protein complex 4, epsilon 1 subunit
2729	TSHZ1	0.4425	0.1621	teashirt zinc finger homeobox 1
2730	LONP2	0.4425	0.2615	lon peptidase 2, peroxisomal
2731	COQ9	0.4425	0.205	coenzyme Q9 homolog (S. cerevisiae)
2732	CHMP4B	0.4425	0.1157	charged multivesicular body protein 4B
2733	BCL6	0.4425	0.1482	B-cell CLL/lymphoma 6
2734	AHSA2	0.4425	0.1849	AHA1, activator of heat shock 90kDa protein ATPase homolog 2 (yeast)
2735	PMVK	0.4417	0.1872	phosphomevalonate kinase
2736	KRTAP1-1	0.4417	0.1989	keratin associated protein 1-1
2737	APPL2	0.4417	0.2236	adaptor protein, phosphotyrosine interaction, PH domain and leucine zipper containing 2
2738	ABL1	0.4417	0.1675	c-abl oncogene 1, non-receptor tyrosine kinase
2739	TRAM1L1	0.4412	0.061	translocation associated membrane protein 1-like 1
2740	STX4	0.4408	0.1967	syntaxin 4
2741	SLC50A1	0.4408	0.119	solute carrier family 50 (sugar transporter), member 1
2742	PRSS12	0.4408	0.0702	protease, serine, 12 (neurotrypsin, motopsin)
2743	LIN7C	0.4408	0.3645	lin-7 homolog C (C. elegans)
2744	B3GNT3	0.4408	0.2344	UDP-GlcNAc:betaGal beta-1,3-N-acetylglucosaminyltransferase 3
2745	ZNF587	0.44	0.1427	zinc finger protein 587
2746	EFHC1	0.44	0.1608	EF-hand domain (C-terminal) containing 1
2747	CDH5	0.44	0.0863	cadherin 5, type 2 (vascular endothelium)
2748	C2CD2	0.44	0.0869	C2 calcium-dependent domain containing 2
2749	ARL6IP5	0.44	0.2938	ADP-ribosylation-like factor 6 interacting protein 5
2750	ANKRD50	0.44	0.2177	ankyrin repeat domain 50
2751	ZNF528	0.4392	0.0872	zinc finger protein 528
2752	ATF6	0.4392	0.1483	activating transcription factor 6
2753	SNHG5	0.4387	0.061	small nucleolar RNA host gene 5 (non-protein coding)
2754	NCOA7	0.4387	0.2153	nuclear receptor coactivator 7
2755	MAN2A1	0.4383	0.2426	mannosidase, alpha, class 2A, member 1
2756	ITFG1	0.4383	0.303	integrin alpha FG-GAP repeat containing 1
2757	PAN3	0.4378	0.262	PAN3 poly(A) specific ribonuclease subunit homolog (S. cerevisiae)
2758	SPARC	0.4375	0.1593	secreted protein, acidic, cysteine-rich (osteonectin)
2759	SGPL1	0.4375	0.1414	sphingosine-1-phosphate lyase 1
2760	RPL23AP32	0.4375	0.0391	ribosomal protein L23a pseudogene 32
2761	DHX32	0.4375	0.0838	DEAH (Asp-Glu-Ala-His) box polypeptide 32
2762	CREBL2	0.4375	0.296	cAMP responsive element binding protein-like 2
2763	CPNE8	0.4375	0.1409	copine VIII
2764	CD226	0.4375	0.0723	CD226 molecule
2765	C9orf64	0.4375	0.239	chromosome 9 open reading frame 64
2766	UFM1	0.4367	0.283	ubiquitin-fold modifier 1
2767	SETD6	0.4367	0.1318	SET domain containing 6
2768	NFE2L1	0.4367	0.1699	nuclear factor (erythroid-derived 2)-like 1
2769	IKBKE	0.4367	0.1449	inhibitor of kappa light polypeptide gene enhancer in B-cells, kinase epsilon
2770	DAD1	0.4367	0.2388	defender against cell death 1

2771	CHIC1	0.4363	0.101	cysteine-rich hydrophobic domain 1
2772	GOLPH3	0.4358	0.2802	golgi phosphoprotein 3 (coat-protein)
2773	CAMKMT	0.4358	0.073	calmodulin-lysine N-methyltransferase
2774	TNFRSF10B	0.435	0.0923	tumor necrosis factor receptor superfamily, member 10b
2775	MID1	0.435	0.08	midline 1 (Opitz/BBB syndrome)
2776	FOPNL	0.435	0.3609	FGFR1OP N-terminal like
2777	ARL10	0.435	0.0222	ADP-ribosylation factor-like 10
2778	ADH5	0.435	0.3236	alcohol dehydrogenase 5 (class III), chi polypeptide
2779	ADAM12	0.435	0.1226	ADAM metallopeptidase domain 12
2780	TYW1	0.4342	0.0942	tRNA-yW synthesizing protein 1 homolog (S. cerevisiae)
2781	TGFBR3	0.4342	0.1098	transforming growth factor, beta receptor III
2782	RABL5	0.4342	0.0969	RAB, member RAS oncogene family-like 5
2783	NFATC4	0.4342	0.2906	nuclear factor of activated T-cells, cytoplasmic, calcineurin-dependent 4
2784	GPR126	0.4342	0.0629	G protein-coupled receptor 126
2785	GLP2R	0.4342	0.1709	glucagon-like peptide 2 receptor
2786	ERP44	0.4342	0.1562	endoplasmic reticulum protein 44
2787	ERAP1	0.4342	0.2321	endoplasmic reticulum aminopeptidase 1
2788	CLUL1	0.4342	0.0669	clusterin-like 1 (retinal)
2789	ARSB	0.4342	0.1045	arylsulfatase B
2790	RSPH3	0.4338	0.0827	radial spoke 3 homolog (Chlamydomonas)
2791	NUDT12	0.4338	0.2502	nudix (nucleoside diphosphate linked moiety X)-type motif 12
2792	TBXA2R	0.4333	0.2541	thromboxane A2 receptor
2793	SLC25A24	0.4333	0.3242	solute carrier family 25 (mitochondrial carrier; phosphate carrier), member 24
2794	OCA2	0.4333	0.1399	oculocutaneous albinism II
2795	GDF5	0.4333	0.2562	growth differentiation factor 5
2796	FILIP1L	0.4333	0.1345	filamin A interacting protein 1-like
2797	ARHGAP6	0.4333	0.0896	Rho GTPase activating protein 6
2798	SMARCA1	0.4325	0.1972	SWI/SNF related, matrix associated, actin dependent regulator of chromatin, subfamily a, member 1
2799	PTGIS	0.4325	0.1748	prostaglandin I2 (prostacyclin) synthase
2800	NUMBL	0.4325	0.0551	numb homolog (Drosophila)-like
2801	MYO1E	0.4325	0.1005	myosin IE
2802	KLHDC7A	0.4325	0.1562	kelch domain containing 7A
2803	C11orf75	0.4325	0.0997	chromosome 11 open reading frame 75
2804	SH3BGRL	0.4317	0.2894	SH3 domain binding glutamic acid-rich protein like
2805	PLAC4	0.4317	0.2794	placenta-specific 4
2806	CYP4F11	0.4317	0.0242	cytochrome P450, family 4, subfamily F, polypeptide 11
2807	ACTA1	0.4317	0.0568	actin, alpha 1, skeletal muscle
2808	PABPC5	0.4313	0.0492	poly(A) binding protein, cytoplasmic 5
2809	SSR4	0.4308	0.1713	signal sequence receptor, delta
2810	TLX1	0.43	0.185	T-cell leukemia homeobox 1
2811	PHF17	0.43	0.1433	PHD finger protein 17
2812	ODF2L	0.43	0.2276	outer dense fiber of sperm tails 2-like
2813	LYSMD3	0.43	0.3474	LysM, putative peptidoglycan-binding, domain containing 3
2814	KRT20	0.43	0.076	keratin 20
2815	EGOT	0.43	0.2258	eosinophil granule ontogeny transcript (non-protein coding)
2816	CYSTM1	0.43	0.1307	cysteine-rich transmembrane module containing 1
2817	CISD3	0.43	0.1693	CDGSH iron sulfur domain 3
2818	CCBP2	0.43	0.204	chemokine binding protein 2
2819	PDE1C	0.4292	0.1694	phosphodiesterase 1C, calmodulin-dependent 70kDa
2820	LAMTOR3	0.4292	0.3582	late endosomal/lysosomal adaptor, MAPK and MTOR activator 3
2821	ZNF506	0.4288	0.2168	zinc finger protein 506
2822	MOB3C	0.4288	0.1244	MOB kinase activator 3C
2823	MBD5	0.4288	0.2045	methyl-CpG binding domain protein 5
2824	ANKRD30BL	0.4286	0.0068	ankyrin repeat domain 30B-like
2825	SIGIRR	0.4283	0.1613	single immunoglobulin and toll-interleukin 1 receptor (TIR) domain
2826	PDCD6IP	0.4283	0.3326	programmed cell death 6 interacting protein
2827	ETV6	0.4283	0.1219	ets variant 6
2828	EPHX2	0.4283	0.0961	epoxide hydrolase 2, cytoplasmic
2829	DDAH1	0.4283	0.0795	dimethylarginine dimethylaminohydrolase 1
2830	CCL21	0.4283	0.2146	chemokine (C-C motif) ligand 21
2831	MST1P9	0.4275	0.0826	macrophage stimulating 1 (hepatocyte growth factor-like) pseudogene 9
2832	ENTPD7	0.4275	0.1234	ectonucleoside triphosphate diphosphohydrolase 7
2833	CD81	0.4275	0.2101	CD81 molecule
2834	BMP1	0.4275	0.2571	bone morphogenetic protein 1
2835	ANKRD22	0.4275	0.0758	ankyrin repeat domain 22
2836	AP3S2	0.4271	0.1114	adaptor-related protein complex 3, sigma 2 subunit
2837	WDR73	0.4267	0.1378	WD repeat domain 73
2838	IL6	0.4267	0.1037	interleukin 6 (interferon, beta 2)
2839	HSPB3	0.4267	0.1324	heat shock 27kDa protein 3
2840	SUSD2	0.4263	0.1322	sushi domain containing 2
2841	SERTAD4	0.4263	0.043	SERTA domain containing 4
2842	GLB1L	0.4258	0.096	galactosidase, beta 1-like
2843	CASP4	0.4258	0.1854	caspase 4, apoptosis-related cysteine peptidase
2844	ABCG2	0.4258	0.0408	ATP-binding cassette, sub-family G (WHITE), member 2
2845	NRK	0.4257	0.043	Nik related kinase
2846	ZADH2	0.425	0.1989	zinc binding alcohol dehydrogenase domain containing 2
2847	TMEM173	0.425	0.1486	transmembrane protein 173
2848	RAMP3	0.425	0.1387	receptor (G protein-coupled) activity modifying protein 3
2849	OVOL1	0.425	0.191	ovo-like 1(Drosophila)
2850	OCRL	0.425	0.1389	oculocerebrorenal syndrome of Lowe
2851	LTC4S	0.425	0.1368	leukotriene C4 synthase
2852	BIRC6	0.425	0.3199	baculoviral IAP repeat containing 6
2853	ZBTB6	0.4242	0.2647	zinc finger and BTB domain containing 6
2854	UGT2A3	0.4242	0.0169	UDP glucuronosyltransferase 2 family, polypeptide A3

2855	TBL1XR1	0.4242	0.3304	transducin (beta)-like 1 X-linked receptor 1
2856	MXD4	0.4242	0.2147	MAX dimerization protein 4
2857	ALS2CR8	0.4242	0.245	amyotrophic lateral sclerosis 2 (juvenile) chromosome region, candidate 8
2858	FGD5	0.4238	0.1011	FYVE, RhoGEF and PH domain containing 5
2859	CYGB	0.4238	0.1643	cytoglobin
2860	C1QTNF2	0.4238	0.069	C1q and tumor necrosis factor related protein 2
2861	STAT2	0.4233	0.1687	signal transducer and activator of transcription 2, 113kDa
2862	CHRDL1	0.4233	0.0664	chordin-like 1
2863	C19orf42	0.4233	0.2464	chromosome 19 open reading frame 42
2864	AEBP1	0.4233	0.1375	AE binding protein 1
2865	MORN2	0.4229	0.2112	MORN repeat containing 2
2866	HCG26	0.4227	0.103	HLA complex group 26 (non-protein coding)
2867	SNAI1	0.4225	0.1252	snail homolog 1 (Drosophila)
2868	S100P	0.4225	0.0585	S100 calcium binding protein P
2869	PEX13	0.4225	0.3312	peroxisomal biogenesis factor 13
2870	NCKAP5	0.4225	0.0398	NCK-associated protein 5
2871	KIAA1468	0.4225	0.3149	KIAA1468
2872	ZNF124	0.4217	0.154	zinc finger protein 124
2873	TUB	0.4217	0.2594	tubby homolog (mouse)
2874	SCGB1D2	0.4217	0.0533	secretoglobin, family 1D, member 2
2875	RRAS2	0.4217	0.1102	related RAS viral (r-ras) oncogene homolog 2
2876	RPE65	0.4217	0.0171	retinal pigment epithelium-specific protein 65kDa
2877	MT1X	0.4217	0.0562	metallothionein 1X
2878	CD3EAP	0.4217	0.1038	CD3e molecule, epsilon associated protein
2879	ADAMTSL3	0.4217	0.0983	ADAMTS-like 3
2880	ACOT11	0.4217	0.1855	acyl-CoA thioesterase 11
2881	ZNF763	0.4214	0.1406	zinc finger protein 763
2882	TMEM216	0.4212	0.0819	transmembrane protein 216
2883	NUDT22	0.4212	0.1823	nudix (nucleoside diphosphate linked moiety X)-type motif 22
2884	GRTP1	0.4212	0.0503	growth hormone regulated TBC protein 1
2885	VPS54	0.4208	0.3765	vacuolar protein sorting 54 homolog (S. cerevisiae)
2886	ROR1	0.4208	0.0887	receptor tyrosine kinase-like orphan receptor 1
2887	PFKFB2	0.4208	0.0916	6-phosphofructo-2-kinase/fructose-2,6-biphosphatase 2
2888	GAN	0.4208	0.1133	gigaxonin
2889	FRZB	0.4208	0.0994	frizzled-related protein
2890	EMP3	0.4208	0.1911	epithelial membrane protein 3
2891	DDOST	0.4208	0.2562	dolichyl-diphosphooligosaccharide--protein glycosyltransferase
2892	ZNF37A	0.42	0.1559	zinc finger protein 37A
2893	SLC35B3	0.42	0.3332	solute carrier family 35, member B3
2894	PNRC1	0.42	0.1943	proline-rich nuclear receptor coactivator 1
2895	NCEH1	0.42	0.222	neutral cholesterol ester hydrolase 1
2896	LOC100128511	0.42	0.133	uncharacterized LOC100128511
2897	KCNJ8	0.42	0.1152	potassium inwardly-rectifying channel, subfamily J, member 8
2898	BCL7C	0.42	0.1945	B-cell CLL/lymphoma 7C
2899	AGXT2L2	0.42	0.1657	alanine-glyoxylate aminotransferase 2-like 2
2900	TJP3	0.4192	0.2228	tight junction protein 3
2901	SPATA20	0.4192	0.1736	spermatogenesis associated 20
2902	MYO6	0.4192	0.1927	myosin VI
2903	HSD3B1	0.4192	0.1517	hydroxy-delta-5-steroid dehydrogenase, 3 beta- and steroid delta-isomerase 1
2904	CTIF	0.4192	0.2428	CBP80/20-dependent translation initiation factor
2905	ATP6AP1	0.4192	0.2227	ATPase, H+ transporting, lysosomal accessory protein 1
2906	LOC100271722	0.4186	0.0491	uncharacterized LOC100271722
2907	NUBPL	0.4183	0.2337	nucleotide binding protein-like
2908	MAN1B1	0.4183	0.2003	mannosidase, alpha, class 1B, member 1
2909	KRT6C	0.4183	0.0234	keratin 6C
2910	DENND1B	0.4178	0.1446	DENN/MADD domain containing 1B
2911	ZNF720	0.4175	0.221	zinc finger protein 720
2912	ZHX1	0.4175	0.327	zinc fingers and homeoboxes 1
2913	ELL3	0.4175	0.0425	elongation factor RNA polymerase II-like 3
2914	BIVM	0.4175	0.2454	basic, immunoglobulin-like variable motif containing
2915	ADAM17	0.4175	0.1647	ADAM metallopeptidase domain 17
2916	ZNF287	0.4167	0.11	zinc finger protein 287
2917	TRDMT1	0.4167	0.2548	tRNA aspartic acid methyltransferase 1
2918	SMAD1	0.4167	0.1401	SMAD family member 1
2919	PRKCDBP	0.4167	0.1346	protein kinase C, delta binding protein
2920	PPP2R3A	0.4167	0.1256	protein phosphatase 2, regulatory subunit B'', alpha
2921	L3MBTL3	0.4162	0.2219	l(3)mbt-like 3 (Drosophila)
2922	ADSSL1	0.4162	0.0638	adenylosuccinate synthase like 1
2923	ZDHHC6	0.4158	0.3312	zinc finger, DHHC-type containing 6
2924	SLC38A2	0.4158	0.2821	solute carrier family 38, member 2
2925	sept-02	0.4158	0.3471	septin 2
2926	RAD51B	0.4158	0.0616	RAD51 homolog B (S. cerevisiae)
2927	IKZF2	0.4158	0.1061	IKAROS family zinc finger 2 (Helios)
2928	CYP2C18	0.4158	0.1504	cytochrome P450, family 2, subfamily C, polypeptide 18
2929	ZNRF3	0.415	0.0962	zinc and ring finger 3
2930	TRIM31	0.415	0.2511	tripartite motif containing 31
2931	TCERG1L	0.415	0.063	transcription elongation regulator 1-like
2932	PTCHD1	0.415	0.0503	patched domain containing 1
2933	NDNF	0.415	0.0803	neuron-derived neurotrophic factor
2934	MLLT6	0.415	0.1575	myeloid/lymphoid or mixed-lineage leukemia (trithorax homolog, Drosophila); translocated to, 6
2935	KLHL3	0.415	0.1226	kelch-like 3 (Drosophila)
2936	EIF4E3	0.415	0.1631	eukaryotic translation initiation factor 4E family member 3
2937	BTG2	0.415	0.1234	BTG family, member 2
2938	ARL13B	0.415	0.3492	ADP-ribosylation factor-like 13B

2939	ALOX15B	0.415	0.2533	arachidonate 15-lipoxygenase, type B
2940	STK38	0.4142	0.2554	serine/threonine kinase 38
2941	LRRC1	0.4142	0.2094	leucine rich repeat containing 1
2942	HHEX	0.4142	0.0797	hematopoietically expressed homeobox
2943	HAL	0.4142	0.064	histidine ammonia-lyase
2944	ESR1	0.4142	0.2636	estrogen receptor 1
2945	XRRA1	0.4137	0.0256	X-ray radiation resistance associated 1
2946	PLA2G4F	0.4137	0.1751	phospholipase A2, group IVF
2947	AK3	0.4137	0.2838	adenylate kinase 3
2948	SLC17A1	0.4133	0.1451	solute carrier family 17 (sodium phosphate), member 1
2949	sept-15	0.4133	0.352	15 kDa selenoprotein
2950	METTL1	0.4133	0.1996	methyltransferase like 1
2951	CLDN16	0.4133	0.1157	claudin 16
2952	C2orf54	0.4133	0.134	chromosome 2 open reading frame 54
2953	VAT1	0.4125	0.1798	vesicle amine transport protein 1 homolog (T. californica)
2954	SLC35A2	0.4125	0.1283	solute carrier family 35 (UDP-galactose transporter), member A2
2955	SEMA3G	0.4125	0.1366	sema domain, immunoglobulin domain (Ig), short basic domain, secreted, (semaphorin) 3G
2956	PLCD3	0.4125	0.1728	phospholipase C, delta 3
2957	PIK3C2G	0.4125	0.1128	phosphatidylinositol-4-phosphate 3-kinase, catalytic subunit type 2 gamma
2958	KLF6	0.4125	0.1723	Kruppel-like factor 6
2959	KIAA2026	0.4125	0.2862	KIAA2026
2960	GALNT1	0.4125	0.3271	UDP-N-acetyl-alpha-D-galactosamine:polypeptide N-acetylgalactosaminyltransferase 1 (GalNAc-T1)
2961	DDX60L	0.4125	0.1914	DEAD (Asp-Glu-Ala-Asp) box polypeptide 60-like
2962	CD40	0.4125	0.1262	CD40 molecule, TNF receptor superfamily member 5
2963	ADH1C	0.4125	0.0626	alcohol dehydrogenase 1C (class I), gamma polypeptide
2964	SYNPO	0.4117	0.22	synaptopodin
2965	MED13L	0.4117	0.2005	mediator complex subunit 13-like
2966	MARC1	0.4117	0.0661	mitochondrial amidoxime reducing component 1
2967	GALNT7	0.4117	0.3227	UDP-N-acetyl-alpha-D-galactosamine:polypeptide N-acetylgalactosaminyltransferase 7 (GalNAc-T7)
2968	CXCR7	0.4117	0.0853	chemokine (C-X-C motif) receptor 7
2969	CRISP3	0.4117	0.0109	cysteine-rich secretory protein 3
2970	KIAA1407	0.4112	0.1609	KIAA1407
2971	ZNF204P	0.4109	0.1397	zinc finger protein 204, pseudogene
2972	SPICE1	0.4108	0.1509	spindle and centriole associated protein 1
2973	CAAP1	0.4108	0.3774	caspase activity and apoptosis inhibitor 1
2974	PLK2	0.41	0.1344	polo-like kinase 2
2975	INTU	0.41	0.1361	inturned planar cell polarity effector homolog (Drosophila)
2976	BTBD11	0.41	0.015	BTB (POZ) domain containing 11
2977	AASS	0.41	0.1037	aminoadipate-semialdehyde synthase
2978	SCIN	0.4092	0.0284	scinderin
2979	RHOB	0.4092	0.1062	ras homolog family member B
2980	QSER1	0.4092	0.2627	glutamine and serine rich 1
2981	NEK11	0.4092	0.1317	NIMA (never in mitosis gene a)- related kinase 11
2982	SLC10A7	0.4087	0.2021	solute carrier family 10 (sodium/bile acid cotransporter family), member 7
2983	PPP1R15B	0.4087	0.2512	protein phosphatase 1, regulatory subunit 15B
2984	LIPH	0.4087	0.0792	lipase, member H
2985	CXCL16	0.4087	0.1368	chemokine (C-X-C motif) ligand 16
2986	ANKS6	0.4087	0.0905	ankyrin repeat and sterile alpha motif domain containing 6
2987	LOC100132707	0.4086	0	uncharacterized LOC100132707
2988	SLC7A9	0.4083	0.1308	solute carrier family 7 (glycoprotein-associated amino acid transporter light chain, bo,+ system), member 9
2989	GBP1	0.4083	0.1822	guanylate binding protein 1, interferon-inducible
2990	CRK	0.4083	0.1872	v-crk sarcoma virus CT10 oncogene homolog (avian)
2991	YES1	0.4075	0.3147	v-yes-1 Yamaguchi sarcoma viral oncogene homolog 1
2992	SLC46A3	0.4075	0.1917	solute carrier family 46, member 3
2993	PLEKHG6	0.4075	0.1924	pleckstrin homology domain containing, family G (with RhoGef domain) member 6
2994	OST4	0.4075	0.1911	oligosaccharyltransferase 4 homolog (S. cerevisiae)
2995	MAP2K3	0.4075	0.1473	mitogen-activated protein kinase kinase 3
2996	CCDC107	0.4075	0.1438	coiled-coil domain containing 107
2997	C15orf41	0.4075	0.1265	chromosome 15 open reading frame 41
2998	MGLL	0.4067	0.1172	monoglyceride lipase
2999	KAZALD1	0.4067	0.1441	Kazal-type serine peptidase inhibitor domain 1
3000	ADPGK	0.4067	0.1588	ADP-dependent glucokinase
3001	SPOPL	0.4063	0.3416	speckle-type POZ protein-like
3002	RHBDL2	0.4063	0.0567	rhomboid, veinlet-like 2 (Drosophila)
3003	KRTAP1-5	0.4063	0.108	keratin associated protein 1-5
3004	TM4SF4	0.4058	0.1675	transmembrane 4 L six family member 4
3005	SLC13A2	0.4058	0.2636	solute carrier family 13 (sodium-dependent dicarboxylate transporter), member 2
3006	OXTR	0.4058	0.0386	oxytocin receptor
3007	LNPEP	0.4058	0.1956	leucyl/cystinyl aminopeptidase
3008	KIAA0319L	0.4058	0.1838	KIAA0319-like
3009	FOSB	0.4058	0.0922	FBJ murine osteosarcoma viral oncogene homolog B
3010	DGCR11	0.4055	0.1765	DiGeorge syndrome critical region gene 11 (non-protein coding)
3011	SUN1	0.405	0.2785	Sad1 and UNC84 domain containing 1
3012	CSTA	0.405	0.0891	cystatin A (stefin A)
3013	SLC19A3	0.4042	0.0916	solute carrier family 19, member 3
3014	SCAMP1	0.4042	0.3259	secretory carrier membrane protein 1
3015	PEX2	0.4042	0.3283	peroxisomal biogenesis factor 2
3016	ANKFY1	0.4042	0.1871	ankyrin repeat and FYVE domain containing 1
3017	PPP1R1B	0.4038	0.1229	protein phosphatase 1, regulatory (inhibitor) subunit 1B
3018	OBSCN	0.4038	0.1622	obscurin, cytoskeletal calmodulin and titin-interacting RhoGEF
3019	CAPNS2	0.4038	0.0404	calpain, small subunit 2
3020	ULBP2	0.4033	0.0756	UL16 binding protein 2
3021	SPIN1	0.4033	0.3192	spindlin 1
3022	SLC6A20	0.4033	0.2033	solute carrier family 6 (proline IMINO transporter), member 20

3023	SHANK2	0.4033	0.1189	SH3 and multiple ankyrin repeat domains 2
3024	PGRMC1	0.4033	0.2783	progesterone receptor membrane component 1
3025	KL	0.4033	0.05	klotho
3026	KRT84	0.4027	0.1882	keratin 84
3027	ZNF252P	0.4025	0.278	zinc finger protein 252, pseudogene
3028	SLC16A2	0.4025	0.1075	solute carrier family 16, member 2 (thyroid hormone transporter)
3029	GRHL1	0.4025	0.039	grainyhead-like 1 (Drosophila)
3030	DENND2C	0.4025	0.0485	DENN/MADD domain containing 2C
3031	CDIPT	0.4025	0.2081	CDP-diacylglycerol--inositol 3-phosphatidyltransferase
3032	BMP8A	0.4025	0.2811	bone morphogenetic protein 8a
3033	ARHGAP21	0.4025	0.1515	Rho GTPase activating protein 21
3034	TUBD1	0.4017	0.2664	tubulin, delta 1
3035	SPRED2	0.4017	0.0939	sprouty-related, EVH1 domain containing 2
3036	KRT31	0.4017	0.2354	keratin 31
3037	DKK2	0.4017	0.0568	dickkopf 2 homolog (Xenopus laevis)
3038	FLJ23867	0.4013	0.1422	uncharacterized protein FLJ23867
3039	TMEM30A	0.4008	0.3499	transmembrane protein 30A
3040	SLC12A1	0.4008	0.0174	solute carrier family 12 (sodium/potassium/chloride transporters), member 1
3041	KCNN4	0.4008	0.0839	potassium intermediate/small conductance calcium-activated channel, subfamily N, member 4
3042	IL15RA	0.4008	0.1177	interleukin 15 receptor, alpha
3043	IBTK	0.4008	0.3714	inhibitor of Bruton agammaglobulinemia tyrosine kinase
3044	F13A1	0.4008	0.1115	coagulation factor XIII, A1 polypeptide
3045	BBIP1	0.4008	0.331	BBSome interacting protein 1
3046	ZNF626	0.4	0.0632	zinc finger protein 626
3047	STK36	0.4	0.1441	serine/threonine kinase 36
3048	RIT1	0.4	0.2546	Ras-like without CAAX 1
3049	KRTAP4-1	0.4	0.0803	keratin associated protein 4-1
3050	FOXA1	0.4	0.0386	forkhead box A1
3051	FMO5	0.4	0.1036	flavin containing monooxygenase 5
3052	CERS2	0.4	0.2204	ceramide synthase 2
3053	CDK13	0.4	0.2691	cyclin-dependent kinase 13
3054	SPINT2	0.3992	0.1076	serine peptidase inhibitor, Kunitz type, 2
3055	SERPINE1	0.3992	0.1503	serpin peptidase inhibitor, clade E (nexin, plasminogen activator inhibitor type 1), member 1
3056	QPCTL	0.3992	0.147	glutaminyl-peptide cyclotransferase-like
3057	NES	0.3992	0.1518	nestin
3058	IFNGR2	0.3992	0.1659	interferon gamma receptor 2 (interferon gamma transducer 1)
3059	ZNF521	0.3988	0.1202	zinc finger protein 521
3060	LOC729852	0.3986	0.3434	uncharacterized LOC729852
3061	VNN1	0.3983	0.0826	vanin 1
3062	TRAF3IP2	0.3983	0.0638	TRAF3 interacting protein 2
3063	SH3PXD2A	0.3983	0.1393	SH3 and PX domains 2A
3064	ATXN1	0.3983	0.2324	ataxin 1
3065	RICTOR	0.3975	0.2936	RPTOR independent companion of MTOR, complex 2
3066	MAVS	0.3975	0.138	mitochondrial antiviral signaling protein
3067	CYBA	0.3975	0.1744	cytochrome b-245, alpha polypeptide
3068	TNFAIP8	0.3967	0.2366	tumor necrosis factor, alpha-induced protein 8
3069	TCTN2	0.3967	0.0757	tectonic family member 2
3070	MOXD1	0.3967	0.1162	monooxygenase, DBH-like 1
3071	IFNGR1	0.3967	0.2866	interferon gamma receptor 1
3072	FAM54B	0.3963	0.2192	family with sequence similarity 54, member B
3073	ECSCR	0.3963	0.1157	endothelial cell surface expressed chemotaxis and apoptosis regulator
3074	DRAM2	0.3963	0.3281	DNA-damage regulated autophagy modulator 2
3075	CCNDBP1	0.3963	0.2485	cyclin D-type binding-protein 1
3076	RXRA	0.3958	0.1617	retinoid X receptor, alpha
3077	KRT2	0.3958	0.169	keratin 2
3078	IPO8	0.3958	0.2479	importin 8
3079	INHBA	0.3958	0.1078	inhibin, beta A
3080	EDA	0.3958	0.2825	ectodysplasin A
3081	CHST15	0.3958	0.1329	carbohydrate (N-acetylgalactosamine 4-sulfate 6-O) sulfotransferase 15
3082	B3GALNT1	0.3958	0.0996	beta-1,3-N-acetylgalactosaminyltransferase 1 (globoside blood group)
3083	SLC6A14	0.395	0.0316	solute carrier family 6 (amino acid transporter), member 14
3084	SLC41A1	0.395	0.0813	solute carrier family 41, member 1
3085	SKI	0.395	0.107	v-ski sarcoma viral oncogene homolog (avian)
3086	SGK2	0.395	0.0994	serum/glucocorticoid regulated kinase 2
3087	LAMTOR1	0.395	0.2216	late endosomal/lysosomal adaptor, MAPK and MTOR activator 1
3088	CALR	0.395	0.15	calreticulin
3089	GMPR2	0.3942	0.2651	guanosine monophosphate reductase 2
3090	TBC1D20	0.3938	0.1503	TBC1 domain family, member 20
3091	SPINK13	0.3938	0.0339	serine peptidase inhibitor, Kazal type 13 (putative)
3092	SLC35E4	0.3938	0.045	solute carrier family 35, member E4
3093	INMT	0.3938	0.0883	indolethylamine N-methyltransferase
3094	FUK	0.3938	0.1561	fucokinase
3095	DOCK11	0.3938	0.2485	dedicator of cytokinesis 11
3096	XBP1	0.3933	0.1691	X-box binding protein 1
3097	RABEP2	0.3933	0.2012	rabaptin, RAB GTPase binding effector protein 2
3098	APAF1	0.3933	0.1719	apoptotic peptidase activating factor 1
3099	ZFAND6	0.3929	0.3136	zinc finger, AN1-type domain 6
3100	USP9Y	0.3925	0.0262	ubiquitin specific peptidase 9, Y-linked
3101	STT3A	0.3925	0.2641	STT3, subunit of the oligosaccharyltransferase complex, homolog A (S. cerevisiae)
3102	PTPLAD2	0.3925	0.1485	protein tyrosine phosphatase-like A domain containing 2
3103	OAF	0.3925	0.1764	OAF homolog (Drosophila)
3104	AFTPH	0.3925	0.2101	aftiphilin
3105	ZNF217	0.3917	0.2979	zinc finger protein 217
3106	ZDHHC7	0.3917	0.1822	zinc finger, DHHC-type containing 7

3107	TNFAIP1	0.3917	0.1449	tumor necrosis factor, alpha-induced protein 1 (endothelial)
3108	RNF186	0.3917	0.1912	ring finger protein 186
3109	PLCD1	0.3917	0.2657	phospholipase C, delta 1
3110	MMRN1	0.3917	0.0568	multimerin 1
3111	HOXB5	0.3917	0.1348	homeobox B5
3112	CILP	0.3917	0.1708	cartilage intermediate layer protein, nucleotide pyrophosphohydrolase
3113	TMEM128	0.3913	0.3331	transmembrane protein 128
3114	SFTA2	0.3913	0.0187	surfactant associated 2
3115	UPK1B	0.3908	0.1552	uroplakin 1B
3116	IVNS1ABP	0.3908	0.2978	influenza virus NS1A binding protein
3117	GREM1	0.3908	0.0875	gremlin 1
3118	EPCAM	0.3908	0.079	epithelial cell adhesion molecule
3119	DERA	0.3908	0.3304	deoxyribose-phosphate aldolase (putative)
3120	WFDC5	0.39	0.1124	WAP four-disulfide core domain 5
3121	TIRAP	0.39	0.1	toll-interleukin 1 receptor (TIR) domain containing adaptor protein
3122	SPNS2	0.39	0.1063	spinster homolog 2 (Drosophila)
3123	RRAGB	0.39	0.1796	Ras-related GTP binding B
3124	NEGR1	0.39	0.0978	neuronal growth regulator 1
3125	JPH2	0.39	0.2948	junctophilin 2
3126	EZR	0.39	0.1779	ezrin
3127	CSN2	0.39	0.1487	casein beta
3128	CDKN2B	0.39	0.0896	cyclin-dependent kinase inhibitor 2B (p15, inhibits CDK4)
3129	TTC30A	0.3892	0.1457	tetratricopeptide repeat domain 30A
3130	ST7	0.3892	0.0683	suppression of tumorigenicity 7
3131	PID1	0.3892	0.0852	phosphotyrosine interaction domain containing 1
3132	COLEC10	0.3892	0.1576	collectin sub-family member 10 (C-type lectin)
3133	ARF4	0.3892	0.319	ADP-ribosylation factor 4
3134	UHMK1	0.3887	0.2309	U2AF homology motif (UHM) kinase 1
3135	PRICKLE2	0.3887	0.1425	prickle homolog 2 (Drosophila)
3136	IER3IP1	0.3887	0.3404	immediate early response 3 interacting protein 1
3137	ZNF223	0.3883	0.1707	zinc finger protein 223
3138	SERPINB4	0.3883	0.1097	serpin peptidase inhibitor, clade B (ovalbumin), member 4
3139	LARS	0.3883	0.3322	leucyl-tRNA synthetase
3140	GOLM1	0.3883	0.1104	golgi membrane protein 1
3141	EDAR	0.3883	0.167	ectodysplasin A receptor
3142	CLIC4	0.3883	0.2479	chloride intracellular channel 4
3143	ZNF32	0.3875	0.2488	zinc finger protein 32
3144	SSTR5-AS1	0.3875	0.142	SSTR5 antisense RNA 1 (non-protein coding)
3145	RAB7L1	0.3875	0.1527	RAB7, member RAS oncogene family-like 1
3146	NPHS1	0.3875	0.2285	nephrosis 1, congenital, Finnish type (nephrin)
3147	MEIS1	0.3875	0.1078	Meis homeobox 1
3148	MAST4	0.3875	0.1226	microtubule associated serine/threonine kinase family member 4
3149	KCNJ1	0.3875	0.2391	potassium inwardly-rectifying channel, subfamily J, member 1
3150	IL36RN	0.3875	0.2304	interleukin 36 receptor antagonist
3151	ENDOU	0.3875	0.1336	endonuclease, polyU-specific
3152	PNLIPRP3	0.3871	0.0408	pancreatic lipase-related protein 3
3153	LOC100289230	0.3871	0.1276	uncharacterized LOC100289230
3154	LOC100049716	0.3871	0.0738	uncharacterized LOC100049716
3155	TMEM40	0.3867	0.1901	transmembrane protein 40
3156	NR1D2	0.3867	0.2641	nuclear receptor subfamily 1, group D, member 2
3157	SMEK2	0.3862	0.3702	SMEK homolog 2, suppressor of mek1 (Dictyostelium)
3158	SHISA5	0.3862	0.1995	shisa homolog 5 (Xenopus laevis)
3159	LOC100129104	0.3862	0.0701	uncharacterized LOC100129104
3160	USP34	0.3858	0.3254	ubiquitin specific peptidase 34
3161	TMC5	0.3858	0.0862	transmembrane channel-like 5
3162	STAC	0.3858	0.0998	SH3 and cysteine rich domain
3163	NPR2	0.3858	0.1336	natriuretic peptide receptor B/guanylate cyclase B (atrionatriuretic peptide receptor B)
3164	LASP1	0.3858	0.2141	LIM and SH3 protein 1
3165	CST3	0.3858	0.2238	cystatin C
3166	MUC5B	0.3855	0.2363	mucin 5B, oligomeric mucus/gel-forming
3167	SHROOM3	0.385	0.0641	shroom family member 3
3168	SH3BP4	0.385	0.1013	SH3-domain binding protein 4
3169	SERTAD4-AS1	0.385	0.057	SERTAD4 antisense RNA 1 (non-protein coding)
3170	SCGB3A1	0.385	0.1631	secretoglobin, family 3A, member 1
3171	RAB3D	0.385	0.166	RAB3D, member RAS oncogene family
3172	MLLT3	0.385	0.1945	myeloid/lymphoid or mixed-lineage leukemia (trithorax homolog, Drosophila); translocated to, 3
3173	ID4	0.385	0.0754	inhibitor of DNA binding 4, dominant negative helix-loop-helix protein
3174	CACHD1	0.385	0.106	cache domain containing 1
3175	TWIST1	0.3842	0.0609	twist homolog 1 (Drosophila)
3176	RPL22	0.3842	0.2465	ribosomal protein L22
3177	GPR125	0.3842	0.1827	G protein-coupled receptor 125
3178	GPD2	0.3842	0.228	glycerol-3-phosphate dehydrogenase 2 (mitochondrial)
3179	FERMT2	0.3842	0.1927	fermitin family member 2
3180	FADD	0.3842	0.1994	Fas (TNFRSF6)-associated via death domain
3181	CNTN5	0.3842	0.054	contactin 5
3182	C7orf10	0.3842	0.0584	chromosome 7 open reading frame 10
3183	AKR7A3	0.3842	0.0965	aldo-keto reductase family 7, member A3 (aflatoxin aldehyde reductase)
3184	ACTC1	0.3842	0.0853	actin, alpha, cardiac muscle 1
3185	SYVN1	0.3837	0.1563	synovial apoptosis inhibitor 1, synoviolin
3186	SERAC1	0.3837	0.2689	serine active site containing 1
3187	HOTAIR	0.3837	0.0134	HOX transcript antisense RNA (non-protein coding)
3188	FGD4	0.3837	0.1709	FYVE, RhoGEF and PH domain containing 4
3189	DNAJC30	0.3837	0.1243	DnaJ (Hsp40) homolog, subfamily C, member 30
3190	TRIB1	0.3833	0.1043	tribbles homolog 1 (Drosophila)

3191	TNFRSF10D	0.3833	0.0793	tumor necrosis factor receptor superfamily, member 10d, decoy with truncated death domain
3192	RBM23	0.3833	0.2157	RNA binding motif protein 23
3193	MPPED2	0.3833	0.0588	metallophosphoesterase domain containing 2
3194	IHH	0.3827	0.3311	Indian hedgehog
3195	YPEL5	0.3825	0.2854	yippee-like 5 (Drosophila)
3196	TMPRSS13	0.3825	0.1014	transmembrane protease, serine 13
3197	SLC33A1	0.3825	0.371	solute carrier family 33 (acetyl-CoA transporter), member 1
3198	SEL1L3	0.3825	0.1416	sel-1 suppressor of lin-12-like 3 (C. elegans)
3199	SEC11A	0.3825	0.2813	SEC11 homolog A (S. cerevisiae)
3200	RNH1	0.3825	0.2356	ribonuclease/angiogenin inhibitor 1
3201	LAMA1	0.3825	0.0276	laminin, alpha 1
3202	CUX1	0.3825	0.1333	cut-like homeobox 1
3203	BANK1	0.3825	0.0874	B-cell scaffold protein with ankyrin repeats 1
3204	TSPAN3	0.3817	0.2815	tetraspanin 3
3205	TNFRSF14	0.3817	0.1733	tumor necrosis factor receptor superfamily, member 14
3206	HARS2	0.3817	0.2506	histidyl-tRNA synthetase 2, mitochondrial
3207	GNRH1	0.3817	0.1018	gonadotropin-releasing hormone 1 (luteinizing-releasing hormone)
3208	EPS8L3	0.3817	0.2025	EPS8-like 3
3209	AGAP11	0.3814	0.0489	ankyrin repeat and GTPase domain Arf GTPase activating protein 11
3210	MMAA	0.3812	0.2106	methylmalonic aciduria (cobalamin deficiency) cblA type
3211	FAM91A1	0.3812	0.3487	family with sequence similarity 91, member A1
3212	A2LD1	0.3812	0.0466	AIG2-like domain 1
3213	SEC63	0.3808	0.3719	SEC63 homolog (S. cerevisiae)
3214	SCGB2A1	0.3808	0.0373	secretoglobin, family 2A, member 1
3215	IFI27	0.3808	0.111	interferon, alpha-inducible protein 27
3216	TGM5	0.38	0.2356	transglutaminase 5
3217	SLC18B1	0.38	0.2115	solute carrier family 18, subfamily B, member 1
3218	PYCARD	0.38	0.1721	PYD and CARD domain containing
3219	MICALL2	0.38	0.1422	MICAL-like 2
3220	IL18	0.38	0.098	interleukin 18 (interferon-gamma-inducing factor)
3221	EML3	0.38	0.2268	echinoderm microtubule associated protein like 3
3222	DSG1	0.38	0.027	desmoglein 1
3223	C6orf170	0.38	0.2744	chromosome 6 open reading frame 170
3224	NUP62CL	0.3792	0.0707	nucleoporin 62kDa C-terminal like
3225	MMP19	0.3792	0.2881	matrix metallopeptidase 19
3226	IMPG1	0.3792	0.0514	interphotoreceptor matrix proteoglycan 1
3227	HLA-G	0.3792	0.1542	major histocompatibility complex, class I, G
3228	WDR36	0.3787	0.3789	WD repeat domain 36
3229	FAM216B	0.3787	0.0407	family with sequence similarity 216, member B
3230	C5orf43	0.3787	0.3803	chromosome 5 open reading frame 43
3231	MOB4	0.3786	0.2805	MOB family member 4, phocein
3232	ZNF234	0.3783	0.1758	zinc finger protein 234
3233	WDR1	0.3783	0.2663	WD repeat domain 1
3234	TMEM100	0.3783	0.0723	transmembrane protein 100
3235	KRT35	0.3783	0.2079	keratin 35
3236	EEA1	0.3783	0.3134	early endosome antigen 1
3237	CYP3A4	0.3783	0.2477	cytochrome P450, family 3, subfamily A, polypeptide 4
3238	ATP1A1	0.3783	0.2157	ATPase, Na+/K+ transporting, alpha 1 polypeptide
3239	ANKRA2	0.3783	0.3386	ankyrin repeat, family A (RFXANK-like), 2
3240	TMEM61	0.3775	0.1547	transmembrane protein 61
3241	TGFBR1	0.3775	0.2146	transforming growth factor, beta receptor 1
3242	SERTM1	0.3775	0.0307	serine-rich and transmembrane domain containing 1
3243	RRM2B	0.3775	0.3185	ribonucleotide reductase M2 B (TP53 inducible)
3244	PYGL	0.3775	0.1267	phosphorylase, glycogen, liver
3245	LMAN2	0.3775	0.2461	lectin, mannose-binding 2
3246	FGFRL1	0.3775	0.1789	fibroblast growth factor receptor-like 1
3247	FBXL7	0.3775	0.1191	F-box and leucine-rich repeat protein 7
3248	DLC1	0.3775	0.1292	deleted in liver cancer 1
3249	BEND7	0.3775	0.0576	BEN domain containing 7
3250	YTHDF3	0.3767	0.3709	YTH domain family, member 3
3251	WWC1	0.3767	0.0741	WW and C2 domain containing 1
3252	SPHK1	0.3767	0.1754	sphingosine kinase 1
3253	SLC47A1	0.3767	0.0252	solute carrier family 47, member 1
3254	SERPINB3	0.3767	0.0694	serpin peptidase inhibitor, clade B (ovalbumin), member 3
3255	MGAT1	0.3767	0.2217	mannosyl (alpha-1,3-)-glycoprotein beta-1,2-N-acetylglucosaminyltransferase
3256	TMTC1	0.3762	0.0983	transmembrane and tetratricopeptide repeat containing 1
3257	TMEM200A	0.3762	0.1027	transmembrane protein 200A
3258	TMEM117	0.3762	0.1424	transmembrane protein 117
3259	TMEM116	0.3762	0.2093	transmembrane protein 116
3260	SLC38A5	0.3762	0.0861	solute carrier family 38, member 5
3261	LIPI	0.3762	0.008	lipase, member I
3262	VLDLR	0.3758	0.0977	very low density lipoprotein receptor
3263	SCN7A	0.3758	0.1307	sodium channel, voltage-gated, type VII, alpha subunit
3264	RALGAPB	0.3758	0.2941	Ral GTPase activating protein, beta subunit (non-catalytic)
3265	C17orf108	0.3758	0.1656	chromosome 17 open reading frame 108
3266	ALG13	0.3758	0.3179	asparagine-linked glycosylation 13 homolog (S. cerevisiae)
3267	NCCRP1	0.3757	0.1465	non-specific cytotoxic cell receptor protein 1 homolog (zebrafish)
3268	SLC5A7	0.375	0.2111	solute carrier family 5 (choline transporter), member 7
3269	SLC31A1	0.375	0.1942	solute carrier family 31 (copper transporters), member 1
3270	SDHD	0.375	0.2824	succinate dehydrogenase complex, subunit D, integral membrane protein
3271	MTNR1A	0.375	0.142	melatonin receptor 1A
3272	HRH4	0.375	0.1298	histamine receptor H4
3273	TRIM22	0.3742	0.2275	tripartite motif containing 22
3274	TNNC1	0.3742	0.0446	troponin C type 1 (slow)

3275	CTNNB1	0.3742	0.2548	catenin (cadherin-associated protein), beta 1, 88kDa
3276	CAMSAP2	0.3742	0.3055	calmodulin regulated spectrin-associated protein family, member 2
3277	HSPA12B	0.3738	0.2301	heat shock 70kD protein 12B
3278	SECISBP2L	0.3733	0.2801	SECIS binding protein 2-like
3279	RAB3GAP2	0.3733	0.3539	RAB3 GTPase activating protein subunit 2 (non-catalytic)
3280	MSRB2	0.3733	0.18	methionine sulfoxide reductase B2
3281	MB	0.3733	0.1381	myoglobin
3282	KRT81	0.3733	0.2205	keratin 81
3283	IFI35	0.3733	0.1955	interferon-induced protein 35
3284	GNE	0.3733	0.262	glucosamine (UDP-N-acetyl)-2-epimerase/N-acetylmannosamine kinase
3285	EVI5	0.3733	0.2834	ecotropic viral integration site 5
3286	CISH	0.3733	0.0756	cytokine inducible SH2-containing protein
3287	ALDH1A3	0.3733	0.0796	aldehyde dehydrogenase 1 family, member A3
3288	ABCC1	0.3733	0.1252	ATP-binding cassette, sub-family C (CFTR/MRP), member 1
3289	UGGT1	0.3725	0.1367	UDP-glucose glycoprotein glucosyltransferase 1
3290	TP53I13	0.3725	0.191	tumor protein p53 inducible protein 13
3291	RBFOX2	0.3725	0.1326	RNA binding protein, fox-1 homolog (C. elegans) 2
3292	IRX1	0.3725	0.0457	iroquois homeobox 1
3293	HAS2	0.3725	0.0951	hyaluronan synthase 2
3294	DPEP1	0.3725	0.2608	dipeptidase 1 (renal)
3295	DCUN1D3	0.3725	0.0944	DCN1, defective in cullin neddylation 1, domain containing 3 (S. cerevisiae)
3296	SPOCK1	0.3717	0.1196	sparc/osteonectin, cwcv and kazal-like domains proteoglycan (testican) 1
3297	PLAUR	0.3717	0.1951	plasminogen activator, urokinase receptor
3298	MINA	0.3717	0.3424	MYC induced nuclear antigen
3299	LAMB4	0.3717	0.1975	laminin, beta 4
3300	HHIPL2	0.3717	0.156	HHIP-like 2
3301	FGF1	0.3717	0.1825	fibroblast growth factor 1 (acidic)
3302	MAL2	0.3713	0.0876	mal, T-cell differentiation protein 2 (gene/pseudogene)
3303	KCTD16	0.3713	0.1137	potassium channel tetramerisation domain containing 16
3304	CPNE2	0.3713	0.1615	copine II
3305	PSAP	0.3708	0.2611	prosaposin
3306	P4HA1	0.3708	0.2331	prolyl 4-hydroxylase, alpha polypeptide I
3307	NCRNA00185	0.3708	0.0347	non-protein coding RNA 185
3308	MGMT	0.3708	0.1451	O-6-methylguanine-DNA methyltransferase
3309	COL10A1	0.3708	0.1648	collagen, type X, alpha 1
3310	ATRN	0.3708	0.2254	attractin
3311	ACBD3	0.3708	0.3177	acyl-CoA binding domain containing 3
3312	ZNFX1	0.37	0.2168	zinc finger, NFX1-type containing 1
3313	ZFPM2	0.37	0.1152	zinc finger protein, multitype 2
3314	SMPDL3A	0.37	0.2037	sphingomyelin phosphodiesterase, acid-like 3A
3315	SLC25A37	0.37	0.084	solute carrier family 25 (mitochondrial iron transporter), member 37
3316	SCYL3	0.37	0.3252	SCY1-like 3 (S. cerevisiae)
3317	RGL2	0.37	0.2022	ral guanine nucleotide dissociation stimulator-like 2
3318	PURA	0.37	0.2316	purine-rich element binding protein A
3319	IGJ	0.37	0.0853	immunoglobulin J polypeptide, linker protein for immunoglobulin alpha and mu polypeptides
3320	ELF3	0.37	0.1686	E74-like factor 3 (ets domain transcription factor, epithelial-specific)
3321	EDN1	0.37	0.1159	endothelin 1
3322	CLRN3	0.37	0.0537	clarin 3
3323	NABP1	0.3692	0.1971	nucleic acid binding protein 1
3324	IL4R	0.3692	0.1691	interleukin 4 receptor
3325	COMT	0.3692	0.2073	catechol-O-methyltransferase
3326	BNC1	0.3692	0.0541	basonuclin 1
3327	TRNP1	0.3688	0.073	TMF1-regulated nuclear protein 1
3328	CNFN	0.3688	0.1048	cornifelin
3329	ADCY4	0.3688	0.2053	adenylate cyclase 4
3330	PTEN	0.3683	0.281	phosphatase and tensin homolog
3331	MT1G	0.3683	0.0598	metallothionein 1G
3332	KRTAP4-7	0.3683	0	keratin associated protein 4-7
3333	C19orf21	0.3683	0.2785	chromosome 19 open reading frame 21
3334	KCTD1	0.3675	0.0584	potassium channel tetramerisation domain containing 1
3335	IFT172	0.3675	0.132	intraflagellar transport 172 homolog (Chlamydomonas)
3336	CMTM8	0.3675	0.0483	CKLF-like MARVEL transmembrane domain containing 8
3337	CYS1	0.3671	0.0935	cystin 1
3338	TRAPPC6A	0.3667	0.1746	trafficking protein particle complex 6A
3339	TNNI2	0.3667	0.1864	troponin I type 2 (skeletal, fast)
3340	AKR1C2	0.3664	0.0527	aldo-keto reductase family 1, member C2 (dihydrodiol dehydrogenase 2; bile acid binding protein; 3-alpha hydroxysteroid dehydrogenase, type III)
3341	SLC7A8	0.3658	0.1484	solute carrier family 7 (amino acid transporter light chain, L system), member 8
3342	PGM5	0.3658	0.1768	phosphoglucomutase 5
3343	NID1	0.3658	0.1525	nidogen 1
3344	ACSL1	0.3658	0.2041	acyl-CoA synthetase long-chain family member 1
3345	ZNFX1-AS1	0.365	0.1383	ZNFX1 antisense RNA 1 (non-protein coding)
3346	YIPF4	0.365	0.348	Yip1 domain family, member 4
3347	HOXA11-AS	0.365	0.1131	HOXA11 antisense RNA (non-protein coding)
3348	FAM114A2	0.365	0.3641	family with sequence similarity 114, member A2
3349	APLF	0.365	0.1114	aprataxin and PNKP like factor
3350	C19orf10	0.3645	0.2305	chromosome 19 open reading frame 10
3351	ZNF135	0.3642	0.0712	zinc finger protein 135
3352	VASP	0.3642	0.1754	vasodilator-stimulated phosphoprotein
3353	TMEM50B	0.3642	0.3304	transmembrane protein 50B
3354	SCYL2	0.3642	0.342	SCY1-like 2 (S. cerevisiae)
3355	ROBO4	0.3642	0.2784	roundabout, axon guidance receptor, homolog 4 (Drosophila)
3356	HOXB2	0.3642	0.0436	homeobox B2
3357	WBSCR27	0.3638	0.1146	Williams Beuren syndrome chromosome region 27
3358	SLC39A11	0.3638	0.1522	solute carrier family 39 (metal ion transporter), member 11

3359	PCDHGA11	0.3636	0.1919	protocadherin gamma subfamily A, 11
3360	TBC1D1	0.3633	0.1012	TBC1 (tre-2/USP6, BUB2, cdc16) domain family, member 1
3361	PTBP3	0.3633	0.295	polypyrimidine tract binding protein 3
3362	GUCY1A2	0.3633	0.1656	guanylate cyclase 1, soluble, alpha 2
3363	ERBB3	0.3633	0.1163	v-erb-b2 erythroblastic leukemia viral oncogene homolog 3 (avian)
3364	CYP4F12	0.3633	0.2106	cytochrome P450, family 4, subfamily F, polypeptide 12
3365	BRCC3	0.3633	0.3631	BRCA1/BRCA2-containing complex, subunit 3
3366	RDH5	0.3629	0.1815	retinol dehydrogenase 5 (11-cis/9-cis)
3367	SSPO	0.3625	0.226	SCO-spondin homolog (Bos taurus)
3368	RTN4	0.3625	0.3084	reticulon 4
3369	IRF2BPL	0.3625	0.0961	interferon regulatory factor 2 binding protein-like
3370	IL33	0.3625	0.0953	interleukin 33
3371	AGTR2	0.3625	0.2233	angiotensin II receptor, type 2
3372	ZNF426	0.3617	0.2321	zinc finger protein 426
3373	TNPO1	0.3617	0.3336	transportin 1
3374	STOM	0.3617	0.1972	stomatin
3375	SLC35E3	0.3617	0.2253	solute carrier family 35, member E3
3376	SLC35A1	0.3617	0.3677	solute carrier family 35 (CMP-sialic acid transporter), member A1
3377	RSG1	0.3617	0.2551	REM2 and RAB-like small GTPase 1
3378	PROM1	0.3617	0.0386	prominin 1
3379	PPDPF	0.3617	0.1953	pancreatic progenitor cell differentiation and proliferation factor homolog (zebrafish)
3380	POLM	0.3617	0.1585	polymerase (DNA directed), mu
3381	FLRT3	0.3617	0.0894	fibronectin leucine rich transmembrane protein 3
3382	FAM106A	0.3617	0.0804	family with sequence similarity 106, member A
3383	ZNF662	0.3614	0.0728	zinc finger protein 662
3384	MTMR10	0.3613	0.2101	myotubularin related protein 10
3385	MIER1	0.3613	0.3364	mesoderm induction early response 1 homolog (Xenopus laevis)
3386	TBC1D9B	0.3608	0.2066	TBC1 domain family, member 9B (with GRAM domain)
3387	RSU1	0.3608	0.3241	Ras suppressor protein 1
3388	RASAL1	0.3608	0.2427	RAS protein activator like 1 (GAP1 like)
3389	RAD50	0.3608	0.3703	RAD50 homolog (S. cerevisiae)
3390	MME	0.3608	0.0861	membrane metallo-endopeptidase
3391	GGPS1	0.3608	0.3427	geranylgeranyl diphosphate synthase 1
3392	EXOC1	0.3608	0.3758	exocyst complex component 1
3393	CBY1	0.3608	0.1565	chibby homolog 1 (Drosophila)
3394	ANGPTL7	0.3608	0.1819	angiopoietin-like 7
3395	UNKL	0.36	0.0839	unkempt homolog (Drosophila)-like
3396	SH3BGRL3	0.36	0.2358	SH3 domain binding glutamic acid-rich protein like 3
3397	PRNP	0.36	0.266	prion protein
3398	FAM110C	0.36	0.0476	family with sequence similarity 110, member C
3399	CADPS2	0.36	0.1091	Ca++-dependent secretion activator 2
3400	TDRD3	0.3592	0.2793	tudor domain containing 3
3401	SLC3A1	0.3592	0.0353	solute carrier family 3 (cystine, dibasic and neutral amino acid transporters, activator of cystine, dibasic and neutral amino acid transport), member
3402	MFSD6	0.3592	0.2518	major facilitator superfamily domain containing 6
3403	CCL8	0.3592	0.1007	chemokine (C-C motif) ligand 8
3404	PCDH11X	0.3583	0.0921	protocadherin 11 X-linked
3405	NR2F6	0.3583	0.2281	nuclear receptor subfamily 2, group F, member 6
3406	ZFY	0.3575	0.0459	zinc finger protein, Y-linked
3407	WFDC1	0.3575	0.1338	WAP four-disulfide core domain 1
3408	TTC37	0.3575	0.4	tetratricopeptide repeat domain 37
3409	PDIA5	0.3575	0.1659	protein disulfide isomerase family A, member 5
3410	MAP4K3	0.3575	0.3855	mitogen-activated protein kinase kinase kinase kinase 3
3411	FKTN	0.3575	0.3234	fukutin
3412	DGKA	0.3575	0.1658	diacylglycerol kinase, alpha 80kDa
3413	C8orf40	0.3575	0.262	chromosome 8 open reading frame 40
3414	BCL10	0.3575	0.2788	B-cell CLL/lymphoma 10
3415	B4GALT4	0.3575	0.1752	UDP-Gal:betaGlcNAc beta 1,4- galactosyltransferase, polypeptide 4
3416	ZNF471	0.3567	0.1081	zinc finger protein 471
3417	TP53BP1	0.3567	0.1322	tumor protein p53 binding protein 1
3418	DYNC2LI1	0.3567	0.3319	dynein, cytoplasmic 2, light intermediate chain 1
3419	NAP1L5	0.3562	0.1578	nucleosome assembly protein 1-like 5
3420	PLEKHA2	0.3558	0.128	pleckstrin homology domain containing, family A (phosphoinositide binding specific) member 2
3421	TRPV6	0.355	0.209	transient receptor potential cation channel, subfamily V, member 6
3422	PRKAR1A	0.355	0.3626	protein kinase, cAMP-dependent, regulatory, type I, alpha
3423	GLI2	0.355	0.2287	GLI family zinc finger 2
3424	KRT33A	0.3543	0.0607	keratin 33A
3425	PCOLCE2	0.3542	0.0517	procollagen C-endopeptidase enhancer 2
3426	DNAJC16	0.3542	0.3279	DnaJ (Hsp40) homolog, subfamily C, member 16
3427	RELL1	0.3537	0.1307	RELT-like 1
3428	HSPB2	0.3533	0.2203	heat shock 27kDa protein 2
3429	HBB	0.3533	0.0753	hemoglobin, beta
3430	ATP9B	0.3533	0.1023	ATPase, class II, type 9B
3431	ALOX12P2	0.3533	0.2911	arachidonate 12-lipoxygenase pseudogene 2
3432	ZNF12	0.3525	0.3412	zinc finger protein 12
3433	XIRP1	0.3525	0.1992	xin actin-binding repeat containing 1
3434	RCBTB2	0.3525	0.283	regulator of chromosome condensation (RCC1) and BTB (POZ) domain containing protein 2
3435	HGF	0.3525	0.1252	hepatocyte growth factor (hepapoietin A; scatter factor)
3436	DPYD	0.3525	0.2703	dihydropyrimidine dehydrogenase
3437	CSNK1G1	0.3525	0.1655	casein kinase 1, gamma 1
3438	VPS13A	0.3517	0.3375	vacuolar protein sorting 13 homolog A (S. cerevisiae)
3439	NAGA	0.3517	0.2142	N-acetylgalactosaminidase, alpha-
3440	MYH1	0.3517	0.0617	myosin, heavy chain 1, skeletal muscle, adult
3441	HDLBP	0.3517	0.1945	high density lipoprotein binding protein
3442	EDA2R	0.3517	0.2498	ectodysplasin A2 receptor

3443	VPS25	0.3512	0.2769	vacuolar protein sorting 25 homolog (S. cerevisiae)
3444	FUNDC1	0.3512	0.3415	FUN14 domain containing 1
3445	DSEL	0.3512	0.1165	dermatan sulfate epimerase-like
3446	SAT1	0.3508	0.222	spermidine/spermine N1-acetyltransferase 1
3447	MAFK	0.3508	0.168	v-maf musculoaponeurotic fibrosarcoma oncogene homolog K (avian)
3448	FXC1	0.3508	0.1994	fracture callus 1 homolog (rat)
3449	CSNK1G3	0.3508	0.3802	casein kinase 1, gamma 3
3450	CFB	0.3508	0.1313	complement factor B
3451	ABCG1	0.3508	0.0861	ATP-binding cassette, sub-family G (WHITE), member 1
3452	SCN9A	0.35	0.0628	sodium channel, voltage-gated, type IX, alpha subunit
3453	RASA2	0.35	0.2301	RAS p21 protein activator 2
3454	PRDM6	0.35	0.1261	PR domain containing 6
3455	PIK3C2A	0.35	0.3692	phosphatidylinositol-4-phosphate 3-kinase, catalytic subunit type 2 alpha
3456	LYPLA2P1	0.35	0.1793	lysophospholipase II pseudogene 1
3457	H1F0	0.35	0.0813	H1 histone family, member 0
3458	TBX5	0.3492	0.285	T-box 5
3459	PRKD3	0.3492	0.365	protein kinase D3
3460	MBTPS2	0.3492	0.2535	membrane-bound transcription factor peptidase, site 2
3461	GLRX	0.3492	0.2031	glutaredoxin (thioltransferase)
3462	CEACAM7	0.3492	0.231	carcinoembryonic antigen-related cell adhesion molecule 7
3463	C14orf93	0.3492	0.1473	chromosome 14 open reading frame 93
3464	APBB2	0.3492	0.1601	amyloid beta (A4) precursor protein-binding, family B, member 2
3465	DCBLD1	0.3487	0.115	discoidin, CUB and LCCL domain containing 1
3466	CHTF8	0.3487	0.2076	CTF8, chromosome transmission fidelity factor 8 homolog (S. cerevisiae)
3467	ABCA13	0.3487	0.0176	ATP-binding cassette, sub-family A (ABC1), member 13
3468	ZNF41	0.3483	0.1679	zinc finger protein 41
3469	XPNPEP2	0.3483	0.2996	X-prolyl aminopeptidase (aminopeptidase P) 2, membrane-bound
3470	STARD5	0.3483	0.1097	StAR-related lipid transfer (START) domain containing 5
3471	SNX5	0.3483	0.3204	sorting nexin 5
3472	SLC39A4	0.3483	0.1395	solute carrier family 39 (zinc transporter), member 4
3473	KIFC3	0.3483	0.2474	kinesin family member C3
3474	CD58	0.3483	0.2923	CD58 molecule
3475	ATM	0.3483	0.2839	ataxia telangiectasia mutated
3476	SLC39A8	0.3475	0.1187	solute carrier family 39 (zinc transporter), member 8
3477	RNF207	0.3475	0.0575	ring finger protein 207
3478	PWWP2B	0.3475	0.2346	PWWP domain containing 2B
3479	EFS	0.3475	0.118	embryonal Fyn-associated substrate
3480	COL21A1	0.3475	0.0861	collagen, type XXI, alpha 1
3481	BLNK	0.3475	0.0975	B-cell linker
3482	TAF1B	0.3467	0.3235	TATA box binding protein (TBP)-associated factor, RNA polymerase I, B, 63kDa
3483	OLAH	0.3467	0.065	oleoyl-ACP hydrolase
3484	MEGF6	0.3467	0.1912	multiple EGF-like-domains 6
3485	DNASE1L3	0.3467	0.0969	deoxyribonuclease I-like 3
3486	DCAF10	0.3467	0.3123	DDB1 and CUL4 associated factor 10
3487	C14orf45	0.3467	0.1852	chromosome 14 open reading frame 45
3488	TYR	0.3458	0.161	tyrosinase (oculocutaneous albinism IA)
3489	SLC22A18AS	0.3458	0.2358	solute carrier family 22 (organic cation transporter), member 18 antisense
3490	NLGN4Y	0.3458	0.0498	neuroligin 4, Y-linked
3491	MEIS2	0.3458	0.1408	Meis homeobox 2
3492	IL12B	0.3458	0.0674	interleukin 12B (natural killer cell stimulatory factor 2, cytotoxic lymphocyte maturation factor 2, p40)
3493	USP43	0.345	0.0405	ubiquitin specific peptidase 43
3494	PTPN4	0.345	0.2444	protein tyrosine phosphatase, non-receptor type 4 (megakaryocyte)
3495	PCDHB7	0.345	0.0544	protocadherin beta 7
3496	FTH1	0.345	0.181	ferritin, heavy polypeptide 1
3497	FLJ43663	0.345	0.1329	uncharacterized LOC378805
3498	OSGIN2	0.3442	0.2584	oxidative stress induced growth inhibitor family member 2
3499	IRAK3	0.3442	0.1569	interleukin-1 receptor-associated kinase 3
3500	DYNC1LI2	0.3442	0.2996	dynein, cytoplasmic 1, light intermediate chain 2
3501	DLG2	0.3442	0.152	discs, large homolog 2 (Drosophila)
3502	CARD14	0.3442	0.3188	caspase recruitment domain family, member 14
3503	C16orf58	0.3442	0.2221	chromosome 16 open reading frame 58
3504	TXNDC12	0.3438	0.3065	thioredoxin domain containing 12 (endoplasmic reticulum)
3505	RBM24	0.3438	0.0751	RNA binding motif protein 24
3506	MRGPRX1	0.3438	0.0234	MAS-related GPR, member X1
3507	FONG	0.3438	0.0238	uncharacterized LOC348751
3508	C11orf45	0.3438	0.0251	chromosome 11 open reading frame 45
3509	TST	0.3433	0.1848	thiosulfate sulfurtransferase (rhodanese)
3510	LOC100288939	0.3433	0	hypothetical LOC100288939
3511	KDM6A	0.3433	0.3157	lysine (K)-specific demethylase 6A
3512	HAO2	0.3433	0.2278	hydroxyacid oxidase 2 (long chain)
3513	CALML3	0.3433	0.2356	calmodulin-like 3
3514	TPPP3	0.3425	0.107	tubulin polymerization-promoting protein family member 3
3515	SPATA7	0.3425	0.2608	spermatogenesis associated 7
3516	LRRC29	0.3425	0.0929	leucine rich repeat containing 29
3517	HYI	0.3425	0.1731	hydroxypyruvate isomerase (putative)
3518	GLTSCR2	0.3425	0.1978	glioma tumor suppressor candidate region gene 2
3519	GFPT2	0.3425	0.13	glutamine-fructose-6-phosphate transaminase 2
3520	DYNLT3	0.3425	0.3085	dynein, light chain, Tctex-type 3
3521	CYP4B1	0.3425	0.117	cytochrome P450, family 4, subfamily B, polypeptide 1
3522	ARMCX3	0.3425	0.334	armadillo repeat containing, X-linked 3
3523	RAPGEFL1	0.3417	0.0936	Rap guanine nucleotide exchange factor (GEF)-like 1
3524	PLAGL1	0.3417	0.2244	pleiomorphic adenoma gene-like 1
3525	FOXL2	0.3417	0.0597	forkhead box L2
3526	ODF3B	0.3414	0.1916	outer dense fiber of sperm tails 3B

3527	LOC400043	0.3414	0.093	uncharacterized LOC400043
3528	WDR19	0.3408	0.2766	WD repeat domain 19
3529	TMPRSS15	0.3408	0.1819	transmembrane protease, serine 15
3530	TBC1D2	0.3408	0.1627	TBC1 domain family, member 2
3531	SNX19	0.3408	0.2045	sorting nexin 19
3532	SEC22A	0.3408	0.3286	SEC22 vesicle trafficking protein homolog A (S. cerevisiae)
3533	GIPC1	0.3408	0.2332	GIPC PDZ domain containing family, member 1
3534	EPB41L1	0.3408	0.129	erythrocyte membrane protein band 4.1-like 1
3535	TMEM14B	0.34	0.3433	transmembrane protein 14B
3536	SLC39A14	0.34	0.148	solute carrier family 39 (zinc transporter), member 14
3537	LOC388692	0.34	0.1032	uncharacterized LOC388692
3538	IGBP1	0.34	0.2597	immunoglobulin (CD79A) binding protein 1
3539	GNAQ	0.34	0.1438	guanine nucleotide binding protein (G protein), q polypeptide
3540	CITED4	0.34	0.2125	Cbp/p300-interacting transactivator, with Glu/Asp-rich carboxy-terminal domain, 4
3541	BDP1	0.34	0.3149	B double prime 1, subunit of RNA polymerase III transcription initiation factor IIIB
3542	VCAM1	0.3392	0.1297	vascular cell adhesion molecule 1
3543	SAMD9	0.3392	0.2431	sterile alpha motif domain containing 9
3544	LGALSL	0.3392	0.0889	lectin, galactoside-binding-like
3545	DNAJC22	0.3392	0.0696	DnaJ (Hsp40) homolog, subfamily C, member 22
3546	CYB5D2	0.3388	0.1943	cytochrome b5 domain containing 2
3547	C2orf15	0.3388	0.098	chromosome 2 open reading frame 15
3548	SLC25A12	0.3383	0.3204	solute carrier family 25 (aspartate/glutamate carrier), member 12
3549	RPS6KA2	0.3383	0.1651	ribosomal protein S6 kinase, 90kDa, polypeptide 2
3550	PCDH1	0.3383	0.307	protocadherin 1
3551	LRRC15	0.3383	0.1954	leucine rich repeat containing 15
3552	IFI16	0.3383	0.2692	interferon, gamma-inducible protein 16
3553	AGMAT	0.3378	0.086	agmatine ureohydrolase (agmatinase)
3554	ZNF250	0.3375	0.126	zinc finger protein 250
3555	TMC7	0.3375	0.1008	transmembrane channel-like 7
3556	NAA20	0.3375	0.3333	N(alpha)-acetyltransferase 20, NatB catalytic subunit
3557	HSD17B4	0.3375	0.3488	hydroxysteroid (17-beta) dehydrogenase 4
3558	GPR176	0.3375	0.1153	G protein-coupled receptor 176
3559	FRMD8	0.3375	0.0703	FERM domain containing 8
3560	ZNF20	0.3371	0.1097	zinc finger protein 20
3561	LRRC66	0.3371	0.0295	leucine rich repeat containing 66
3562	LOC728431	0.3371	0.1298	uncharacterized LOC728431
3563	ZNF717	0.3367	0.1428	zinc finger protein 717
3564	ZNF167	0.3367	0.1134	zinc finger protein 167
3565	ZC3HAV1	0.3367	0.2229	zinc finger CCCH-type, antiviral 1
3566	TRIM68	0.3367	0.2308	tripartite motif containing 68
3567	TCEAL1	0.3367	0.3032	transcription elongation factor A (SII)-like 1
3568	SERPINB1	0.3367	0.2342	serpin peptidase inhibitor, clade B (ovalbumin), member 1
3569	OAT	0.3367	0.327	ornithine aminotransferase
3570	MUC13	0.3367	0.2112	mucin 13, cell surface associated
3571	HOXB6	0.3367	0.0249	homeobox B6
3572	HMOX1	0.3367	0.1542	heme oxygenase (decycling) 1
3573	GATA6	0.3367	0.0593	GATA binding protein 6
3574	FBXO22	0.3367	0.2863	F-box protein 22
3575	DKK3	0.3367	0.1655	dickkopf 3 homolog (Xenopus laevis)
3576	CHM	0.3367	0.3096	choroideremia (Rab escort protein 1)
3577	AUH	0.3367	0.2811	AU RNA binding protein/enoyl-CoA hydratase
3578	TCP11L2	0.3363	0.1749	t-complex 11 (mouse)-like 2
3579	PLEKHS1	0.3363	0.0141	pleckstrin homology domain containing, family S member 1
3580	SPSB1	0.3358	0.1666	splA/ryanodine receptor domain and SOCS box containing 1
3581	PGAP2	0.3358	0.1725	post-GPI attachment to proteins 2
3582	ITPR1	0.3358	0.2199	inositol 1,4,5-trisphosphate receptor, type 1
3583	FBXL5	0.3358	0.352	F-box and leucine-rich repeat protein 5
3584	CDC42BPA	0.3358	0.1747	CDC42 binding protein kinase alpha (DMPK-like)
3585	BPIFA4P	0.3357	0.1982	BPI fold containing family A, member 4, pseudogene
3586	ZNF682	0.335	0.1645	zinc finger protein 682
3587	ZNF582	0.335	0.0686	zinc finger protein 582
3588	TMF1	0.335	0.3786	TATA element modulatory factor 1
3589	RSPO1	0.335	0.1723	R-spondin 1
3590	KIF13A	0.335	0.1153	kinesin family member 13A
3591	ETV4	0.335	0.2237	ets variant 4
3592	COMP	0.335	0.1561	cartilage oligomeric matrix protein
3593	CHRNA9	0.335	0.0935	cholinergic receptor, nicotinic, alpha 9 (neuronal)
3594	C8orf42	0.335	0.0604	chromosome 8 open reading frame 42
3595	ADAMTS7	0.3343	0.2452	ADAM metallopeptidase with thrombospondin type 1 motif, 7
3596	ZC2HC1A	0.3342	0.2285	zinc finger, C2HC-type containing 1A
3597	TNFAIP3	0.3342	0.1987	tumor necrosis factor, alpha-induced protein 3
3598	PAK3	0.3342	0.2118	p21 protein (Cdc42/Rac)-activated kinase 3
3599	NGFR	0.3342	0.1596	nerve growth factor receptor
3600	ADH7	0.3342	0.1105	alcohol dehydrogenase 7 (class IV), mu or sigma polypeptide
3601	SGK223	0.3338	0.0829	homolog of rat pragma of Rnd2
3602	GDA	0.3338	0.0458	guanine deaminase
3603	C5orf62	0.3338	0.1171	chromosome 5 open reading frame 62
3604	ALPK1	0.3338	0.193	alpha-kinase 1
3605	TP53	0.3333	0.1982	tumor protein p53
3606	POLR2L	0.3333	0.2501	polymerase (RNA) II (DNA directed) polypeptide L, 7.6kDa
3607	PHLDA3	0.3333	0.2043	pleckstrin homology-like domain, family A, member 3
3608	CKMT2	0.3333	0.1049	creatine kinase, mitochondrial 2 (sarcomeric)
3609	CASQ2	0.3333	0.2408	calsequestrin 2 (cardiac muscle)
3610	XIST	0.3325	0.0557	X (inactive)-specific transcript (non-protein coding)

3611	WFS1	0.3325	0.1579	Wolfram syndrome 1 (wolframin)
3612	STX5	0.3325	0.2011	syntaxin 5
3613	PKP4	0.3325	0.2571	plakophilin 4
3614	IL10RB	0.3325	0.2637	interleukin 10 receptor, beta
3615	IGF1	0.3325	0.1749	insulin-like growth factor 1 (somatomedin C)
3616	FAM176B	0.3325	0.2293	family with sequence similarity 176, member B
3617	EPHA5	0.3325	0.0764	EPH receptor A5
3618	EML1	0.3325	0.1448	echinoderm microtubule associated protein like 1
3619	C8orf48	0.3325	0.0692	chromosome 8 open reading frame 48
3620	STARD8	0.3317	0.1994	StAR-related lipid transfer (START) domain containing 8
3621	SLC7A2	0.3317	0.0669	solute carrier family 7 (cationic amino acid transporter, y+ system), member 2
3622	RAB11A	0.3317	0.3868	RAB11A, member RAS oncogene family
3623	POFUT1	0.3317	0.1127	protein O-fucosyltransferase 1
3624	MSX2	0.3317	0.0347	msh homeobox 2
3625	MST1R	0.3317	0.2154	macrophage stimulating 1 receptor (c-met-related tyrosine kinase)
3626	CYP2B6	0.3317	0.1577	cytochrome P450, family 2, subfamily B, polypeptide 6
3627	ZDHHC16	0.3313	0.2816	zinc finger, DHHC-type containing 16
3628	TMEM67	0.3313	0.2305	transmembrane protein 67
3629	DLX3	0.3313	0.1545	distal-less homeobox 3
3630	UNC45A	0.3308	0.2356	unc-45 homolog A (C. elegans)
3631	PHB	0.3308	0.3069	prohibitin
3632	MFI2	0.3308	0.1607	antigen p97 (melanoma associated) identified by monoclonal antibodies 133.2 and 96.5
3633	LRAT	0.3308	0.0605	lecithin retinol acyltransferase (phosphatidylcholine--retinol O-acyltransferase)
3634	EFHD1	0.3308	0.1321	EF-hand domain family, member D1
3635	ZNF114	0.33	0.0472	zinc finger protein 114
3636	YOD1	0.33	0.1695	YOD1 OTU deubiquinating enzyme 1 homolog (S. cerevisiae)
3637	SSU72	0.33	0.2657	SSU72 RNA polymerase II CTD phosphatase homolog (S. cerevisiae)
3638	PRDX1	0.33	0.3217	peroxiredoxin 1
3639	POLK	0.33	0.3743	polymerase (DNA directed) kappa
3640	LINC00277	0.33	0.1432	long intergenic non-protein coding RNA 277
3641	KBTBD7	0.33	0.3461	kelch repeat and BTB (POZ) domain containing 7
3642	ERCC5	0.33	0.2672	excision repair cross-complementing rodent repair deficiency, complementation group 5
3643	EMC7	0.33	0.3345	ER membrane protein complex subunit 7
3644	CHRM3	0.33	0.0607	cholinergic receptor, muscarinic 3
3645	CFHR2	0.33	0.0846	complement factor H-related 2
3646	CCDC159	0.33	0.2237	coiled-coil domain containing 159
3647	B3GNT5	0.33	0.1875	UDP-GlcNAc:betaGal beta-1,3-N-acetylglucosaminyltransferase 5
3648	TMPRSS3	0.3292	0.0536	transmembrane protease, serine 3
3649	PNPLA3	0.3292	0.081	patatin-like phospholipase domain containing 3
3650	TMEM79	0.3288	0.0841	transmembrane protein 79
3651	TLCD1	0.3288	0.1576	TLC domain containing 1
3652	ZCCHC4	0.3283	0.2634	zinc finger, CCHC domain containing 4
3653	THEM6	0.3283	0.1781	thioesterase superfamily member 6
3654	PLA2G2A	0.3283	0.1688	phospholipase A2, group IIA (platelets, synovial fluid)
3655	PIGV	0.3283	0.2343	phosphatidylinositol glycan anchor biosynthesis, class V
3656	NPIPL2	0.3283	0.1663	nuclear pore complex interacting protein-like 2
3657	FAM179B	0.3283	0.3851	family with sequence similarity 179, member B
3658	ESRP2	0.3283	0.1704	epithelial splicing regulatory protein 2
3659	CMKLR1	0.3283	0.2375	chemokine-like receptor 1
3660	C10orf10	0.3283	0.1877	chromosome 10 open reading frame 10
3661	LOC339290	0.3282	0.1163	uncharacterized LOC339290
3662	LOC100130100	0.3282	0.175	Ig kappa chain V-I region Walker-like
3663	USO1	0.3275	0.3763	USO1 vesicle docking protein homolog (yeast)
3664	TMEM246	0.3275	0.0582	transmembrane protein 246
3665	PTPN18	0.3275	0.1985	protein tyrosine phosphatase, non-receptor type 18 (brain-derived)
3666	NAV2	0.3275	0.0931	neuron navigator 2
3667	MAN1A1	0.3275	0.2223	mannosidase, alpha, class 1A, member 1
3668	HSD17B12	0.3275	0.3597	hydroxysteroid (17-beta) dehydrogenase 12
3669	FOXF1	0.3275	0.1161	forkhead box F1
3670	C22orf29	0.3275	0.143	chromosome 22 open reading frame 29
3671	APCDD1	0.3275	0.0821	adenomatosis polyposis coli down-regulated 1
3672	ALKBH3	0.3275	0.2998	alkB, alkylation repair homolog 3 (E. coli)
3673	PLOD3	0.3267	0.2327	procollagen-lysine, 2-oxoglutarate 5-dioxygenase 3
3674	IDH1	0.3267	0.3268	isocitrate dehydrogenase 1 (NADP+), soluble
3675	CDKN1A	0.3267	0.1945	cyclin-dependent kinase inhibitor 1A (p21, Cip1)
3676	SPIRE2	0.3262	0.1443	spire homolog 2 (Drosophila)
3677	RNF213	0.3262	0.1883	ring finger protein 213
3678	TMEM165	0.3258	0.3129	transmembrane protein 165
3679	REL	0.3258	0.2318	v-rel reticuloendotheliosis viral oncogene homolog (avian)
3680	PSMD5	0.3258	0.3839	proteasome (prosome, macropain) 26S subunit, non-ATPase, 5
3681	PDPR	0.3258	0.1438	pyruvate dehydrogenase phosphatase regulatory subunit
3682	INPPL1	0.3258	0.2288	inositol polyphosphate phosphatase-like 1
3683	UBE2D4	0.325	0.1729	ubiquitin-conjugating enzyme E2D 4 (putative)
3684	SYT14	0.325	0.036	synaptotagmin XIV
3685	PIWIL4	0.325	0.0981	piwi-like 4 (Drosophila)
3686	MSR1	0.325	0.1692	macrophage scavenger receptor 1
3687	METTL9	0.325	0.2588	methyltransferase like 9
3688	KCNIP3	0.325	0.2852	Kv channel interacting protein 3, calsenilin
3689	TMEM134	0.3242	0.2075	transmembrane protein 134
3690	SIL1	0.3242	0.2081	SIL1 homolog, endoplasmic reticulum chaperone (S. cerevisiae)
3691	SERPINB6	0.3242	0.2077	serpin peptidase inhibitor, clade B (ovalbumin), member 6
3692	GOSR2	0.3242	0.2094	golgi SNAP receptor complex member 2
3693	FXYD5	0.3242	0.214	FXYD domain containing ion transport regulator 5
3694	CXorf40B	0.3242	0.1638	chromosome X open reading frame 40B

3695	ARHGAP1	0.3242	0.2252	Rho GTPase activating protein 1
3696	ABCC6	0.3242	0.2345	ATP-binding cassette, sub-family C (CFTR/MRP), member 6
3697	SLFN13	0.3237	0.0624	schlafen family member 13
3698	SLC26A11	0.3237	0.1372	solute carrier family 26, member 11
3699	SKIDA1	0.3237	0.0407	SKI/DACH domain containing 1
3700	VPS28	0.3233	0.261	vacuolar protein sorting 28 homolog (S. cerevisiae)
3701	SLC15A2	0.3233	0.0978	solute carrier family 15 (H+/peptide transporter), member 2
3702	FLT4	0.3233	0.2387	fms-related tyrosine kinase 4
3703	DIEXF	0.3233	0.338	digestive organ expansion factor homolog (zebrafish)
3704	CLIC1	0.3233	0.2887	chloride intracellular channel 1
3705	WWP1	0.3225	0.3739	WW domain containing E3 ubiquitin protein ligase 1
3706	TMEM135	0.3225	0.3858	transmembrane protein 135
3707	NOV	0.3225	0.1113	nephroblastoma overexpressed
3708	MRPL43	0.3225	0.2825	mitochondrial ribosomal protein L43
3709	IP6K3	0.3225	0.0779	inositol hexakisphosphate kinase 3
3710	HS1BP3	0.3225	0.2127	HCLS1 binding protein 3
3711	CCDC81	0.3225	0.0437	coiled-coil domain containing 81
3712	CCDC127	0.3225	0.2338	coiled-coil domain containing 127
3713	C7	0.3225	0.1407	complement component 7
3714	C14orf119	0.3225	0.3246	chromosome 14 open reading frame 119
3715	RGS16	0.3217	0.1325	regulator of G-protein signaling 16
3716	PTTG1IP	0.3217	0.2837	pituitary tumor-transforming 1 interacting protein
3717	PSKH1	0.3217	0.2031	protein serine kinase H1
3718	NDST1	0.3217	0.2365	N-deacetylase/N-sulfotransferase (heparan glucosaminyl) 1
3719	EHHADH	0.3217	0.2317	enoyl-CoA, hydratase/3-hydroxyacyl CoA dehydrogenase
3720	CREBZF	0.3217	0.3404	CREB/ATF bZIP transcription factor
3721	CASZ1	0.3217	0.2403	castor zinc finger 1
3722	PHGR1	0.3214	0.0955	proline/histidine/glycine-rich 1
3723	LOC201651	0.3214	0	arylacetamide deacetylase (esterase) pseudogene
3724	SUDS3	0.3212	0.3498	suppressor of defective silencing 3 homolog (S. cerevisiae)
3725	FBXO8	0.3212	0.3636	F-box protein 8
3726	UTP14C	0.3208	0.3967	UTP14, U3 small nucleolar ribonucleoprotein, homolog C (yeast)
3727	TUBB6	0.3208	0.2007	tubulin, beta 6 class V
3728	HOXC13	0.3208	0.1849	homeobox C13
3729	ZC3H11A	0.32	0.3215	zinc finger CCCH-type containing 11A
3730	TAF1	0.32	0.1873	TAF1 RNA polymerase II, TATA box binding protein (TBP)-associated factor, 250kDa
3731	SORBS3	0.32	0.293	sorbin and SH3 domain containing 3
3732	PKP2	0.32	0.0628	plakophilin 2
3733	PHC3	0.32	0.3541	polyhomeotic homolog 3 (Drosophila)
3734	PECR	0.32	0.1196	peroxisomal trans-2-enoyl-CoA reductase
3735	IFT88	0.32	0.3078	intraflagellar transport 88 homolog (Chlamydomonas)
3736	HAND2	0.32	0.1432	heart and neural crest derivatives expressed 2
3737	CLDN10	0.32	0.1091	claudin 10
3738	ADAMTSL1	0.32	0.2008	ADAMTS-like 1
3739	PML	0.3192	0.2756	promyelocytic leukemia
3740	MRPL24	0.3192	0.2962	mitochondrial ribosomal protein L24
3741	IRX4	0.3192	0.2059	iroquois homeobox 4
3742	GNAT3	0.3191	0.1966	guanine nucleotide binding protein, alpha transducing 3
3743	METTL10	0.3187	0.3961	methyltransferase like 10
3744	LOC100132356	0.3187	0.0545	uncharacterized LOC100132356
3745	ADAMTS9	0.3187	0.1031	ADAM metallopeptidase with thrombospondin type 1 motif, 9
3746	YIPF1	0.3183	0.2257	Yip1 domain family, member 1
3747	VKORC1	0.3183	0.2519	vitamin K epoxide reductase complex, subunit 1
3748	TOM1	0.3183	0.2492	target of myb1 (chicken)
3749	RBL2	0.3183	0.331	retinoblastoma-like 2 (p130)
3750	RAB17	0.3183	0.1724	RAB17, member RAS oncogene family
3751	KIAA0247	0.3183	0.2549	KIAA0247
3752	ITIH5	0.3183	0.1618	inter-alpha-trypsin inhibitor heavy chain family, member 5
3753	F3	0.3183	0.1454	coagulation factor III (thromboplastin, tissue factor)
3754	SWI5	0.3175	0.1875	SWI5 recombination repair homolog (yeast)
3755	STAB1	0.3175	0.2394	stabilin 1
3756	sept-11	0.3175	0.3174	septin 11
3757	PRTFDC1	0.3175	0.2491	phosphoribosyl transferase domain containing 1
3758	MMP10	0.3175	0.0682	matrix metallopeptidase 10 (stromelysin 2)
3759	MITF	0.3175	0.1388	microphthalmia-associated transcription factor
3760	COL18A1	0.3175	0.1686	collagen, type XVIII, alpha 1
3761	BLVRB	0.3175	0.1934	biliverdin reductase B (flavin reductase (NADPH))
3762	SLC38A10	0.3167	0.2333	solute carrier family 38, member 10
3763	PLK3	0.3167	0.1904	polo-like kinase 3
3764	PACSIN2	0.3167	0.1702	protein kinase C and casein kinase substrate in neurons 2
3765	KCNAB1	0.3167	0.1525	potassium voltage-gated channel, shaker-related subfamily, beta member 1
3766	C1orf27	0.3167	0.3777	chromosome 1 open reading frame 27
3767	ABHD12	0.3162	0.1898	abhydrolase domain containing 12
3768	NRIP1	0.3158	0.3279	nuclear receptor interacting protein 1
3769	NIT1	0.3158	0.2077	nitrilase 1
3770	MT2A	0.3158	0.1542	metallothionein 2A
3771	MOCOS	0.3158	0.069	molybdenum cofactor sulfurase
3772	HECTD3	0.3158	0.2163	HECT domain containing E3 ubiquitin protein ligase 3
3773	TMEM141	0.315	0.2258	transmembrane protein 141
3774	SAR1B	0.315	0.3638	SAR1 homolog B (S. cerevisiae)
3775	RAB5C	0.315	0.224	RAB5C, member RAS oncogene family
3776	QTRT1	0.315	0.2254	queuine tRNA-ribosyltransferase 1
3777	HTATSF1P2	0.315	0	HIV-1 Tat specific factor 1 pseudogene 2
3778	ARHGAP32	0.315	0.1531	Rho GTPase activating protein 32

3779	WTIP	0.3143	0.2386	Wilms tumor 1 interacting protein
3780	ACY1	0.3143	0.2226	aminoacylase 1
3781	PRUNE	0.3142	0.2181	prune homolog (Drosophila)
3782	KIRREL	0.3142	0.223	kin of IRRE like (Drosophila)
3783	UBR3	0.3137	0.4054	ubiquitin protein ligase E3 component n-recognin 3 (putative)
3784	TTC5	0.3137	0.2277	tetratricopeptide repeat domain 5
3785	RHOV	0.3137	0.1101	ras homolog family member V
3786	ADAMTSL4	0.3137	0.1299	ADAMTS-like 4
3787	R3HDM2	0.3133	0.1681	R3H domain containing 2
3788	HR	0.3133	0.3194	hairless homolog (mouse)
3789	FXYD3	0.3133	0.235	FXYD domain containing ion transport regulator 3
3790	CLCA3P	0.3133	0.0375	chloride channel accessory 3, pseudogene
3791	SYNM	0.3125	0.1053	synemin, intermediate filament protein
3792	MTIF3	0.3125	0.3277	mitochondrial translational initiation factor 3
3793	LAMB3	0.3125	0.1283	laminin, beta 3
3794	PGR	0.3117	0.1398	progesterone receptor
3795	SEPN1	0.3113	0.239	selenoprotein N, 1
3796	GLIS1	0.3113	0.1685	GLIS family zinc finger 1
3797	BOK	0.3113	0.1595	BCL2-related ovarian killer
3798	SUCLG1	0.3108	0.3145	succinate-CoA ligase, alpha subunit
3799	GOSR1	0.3108	0.3923	golgi SNAP receptor complex member 1
3800	CHI3L2	0.3108	0.0909	chitinase 3-like 2
3801	TMUB2	0.31	0.2232	transmembrane and ubiquitin-like domain containing 2
3802	TBC1D24	0.31	0.0919	TBC1 domain family, member 24
3803	HSPB7	0.31	0.2908	heat shock 27kDa protein family, member 7 (cardiovascular)
3804	CERS6	0.31	0.321	ceramide synthase 6
3805	TTC38	0.3092	0.182	tetratricopeptide repeat domain 38
3806	NFKB2	0.3092	0.2384	nuclear factor of kappa light polypeptide gene enhancer in B-cells 2 (p49/p100)
3807	CD97	0.3092	0.207	CD97 molecule
3808	CMBL	0.3088	0.103	carboxymethylenebutenolidase homolog (Pseudomonas)
3809	STK24	0.3083	0.2686	serine/threonine kinase 24
3810	RASAL2	0.3083	0.1306	RAS protein activator like 2
3811	TMEM125	0.3075	0.1412	transmembrane protein 125
3812	SMPD1	0.3075	0.2444	sphingomyelin phosphodiesterase 1, acid lysosomal
3813	PKD2L2	0.3075	0.0261	polycystic kidney disease 2-like 2
3814	NAGS	0.3075	0.0746	N-acetylglutamate synthase
3815	HSDL2	0.3075	0.3606	hydroxysteroid dehydrogenase like 2
3816	GOLGA2	0.3075	0.2025	golgin A2
3817	GNB4	0.3075	0.293	guanine nucleotide binding protein (G protein), beta polypeptide 4
3818	EIF2A	0.3075	0.3612	eukaryotic translation initiation factor 2A, 65kDa
3819	CXCL11	0.3075	0.1018	chemokine (C-X-C motif) ligand 11
3820	CCDC64B	0.3075	0.2109	coiled-coil domain containing 64B
3821	BZW2	0.3075	0.319	basic leucine zipper and W2 domains 2
3822	ARF1	0.3075	0.2715	ADP-ribosylation factor 1
3823	UNC13B	0.3067	0.134	unc-13 homolog B (C. elegans)
3824	TP53AIP1	0.3067	0.2804	tumor protein p53 regulated apoptosis inducing protein 1
3825	SLC17A3	0.3067	0.1989	solute carrier family 17 (sodium phosphate), member 3
3826	PLXNA3	0.3067	0.1937	plexin A3
3827	NPC2	0.3067	0.2531	Niemann-Pick disease, type C2
3828	HSD17B10	0.3067	0.2905	hydroxysteroid (17-beta) dehydrogenase 10
3829	DUSP3	0.3067	0.1952	dual specificity phosphatase 3
3830	OXGR1	0.3063	0.0446	oxoglutarate (alpha-ketoglutarate) receptor 1
3831	MTA3	0.3063	0.2204	metastasis associated 1 family, member 3
3832	ZNF586	0.3058	0.156	zinc finger protein 586
3833	VENTXP1	0.3058	0.146	VENT homeobox pseudogene 1
3834	TGM2	0.3058	0.1952	transglutaminase 2 (C polypeptide, protein-glutamine-gamma-glutamyltransferase)
3835	NFKBIA	0.3058	0.181	nuclear factor of kappa light polypeptide gene enhancer in B-cells inhibitor, alpha
3836	MPZ	0.3058	0.276	myelin protein zero
3837	CLEC1A	0.3058	0.2126	C-type lectin domain family 1, member A
3838	SPATA6	0.305	0.1543	spermatogenesis associated 6
3839	MMP16	0.305	0.227	matrix metallopeptidase 16 (membrane-inserted)
3840	LDOC1	0.305	0.1281	leucine zipper, down-regulated in cancer 1
3841	AKR1A1	0.305	0.2601	aldo-keto reductase family 1, member A1 (aldehyde reductase)
3842	ACACB	0.305	0.203	acetyl-CoA carboxylase beta
3843	ABLIM3	0.305	0.1619	actin binding LIM protein family, member 3
3844	MTFR1	0.3042	0.3731	mitochondrial fission regulator 1
3845	MC3R	0.3042	0.2131	melanocortin 3 receptor
3846	ZSWIM6	0.3038	0.2735	zinc finger, SWIM-type containing 6
3847	LCLAT1	0.3038	0.3905	lysocardiolipin acyltransferase 1
3848	DNAJB11	0.3038	0.3204	DnaJ (Hsp40) homolog, subfamily B, member 11
3849	TMEM2	0.3033	0.2151	transmembrane protein 2
3850	ID2	0.3033	0.1667	inhibitor of DNA binding 2, dominant negative helix-loop-helix protein
3851	HIF1A	0.3033	0.3313	hypoxia inducible factor 1, alpha subunit (basic helix-loop-helix transcription factor)
3852	ECHS1	0.3033	0.2949	enoyl CoA hydratase, short chain, 1, mitochondrial
3853	MYLK4	0.3029	0.0807	myosin light chain kinase family, member 4
3854	RPN1	0.3025	0.3075	ribophorin I
3855	PRR16	0.3025	0.1037	proline rich 16
3856	PIGY	0.3025	0.3625	phosphatidylinositol glycan anchor biosynthesis, class Y
3857	OSBPL10	0.3025	0.0672	oxysterol binding protein-like 10
3858	NLRP1	0.3025	0.1942	NLR family, pyrin domain containing 1
3859	DCAF16	0.3025	0.2781	DDB1 and CUL4 associated factor 16
3860	CRLF1	0.3025	0.1525	cytokine receptor-like factor 1
3861	BTN3A3	0.3025	0.2831	butyrophilin, subfamily 3, member A3
3862	BDKRB1	0.3025	0.1939	bradykinin receptor B1

3863	AHI1	0.3025	0.2139	Abelson helper integration site 1
3864	TAP2	0.3017	0.1577	transporter 2, ATP-binding cassette, sub-family B (MDR/TAP)
3865	PLEKHA6	0.3017	0.2099	pleckstrin homology domain containing, family A member 6
3866	PEX19	0.3017	0.3491	peroxisomal biogenesis factor 19
3867	MLX	0.3017	0.2163	MAX-like protein X
3868	GOLGA1	0.3017	0.2708	golgin A1
3869	DES	0.3017	0.2994	desmin
3870	LOC100288152	0.3014	0	uncharacterized LOC100288152
3871	TRPT1	0.3013	0.2029	tRNA phosphotransferase 1
3872	IGFN1	0.3013	0.2258	immunoglobulin-like and fibronectin type III domain containing 1
3873	FLJ42709	0.3013	0.136	uncharacterized LOC441094
3874	DUOXA2	0.3013	0.213	dual oxidase maturation factor 2
3875	CGN	0.3013	0.1263	cingulin
3876	C9orf89	0.3013	0.1707	chromosome 9 open reading frame 89
3877	UPK3A	0.3008	0.259	uroplakin 3A
3878	SPTAN1	0.3008	0.2458	spectrin, alpha, non-erythrocytic 1
3879	OSGIN1	0.3008	0.2909	oxidative stress induced growth inhibitor 1
3880	JMJD1C	0.3008	0.3145	jumonji domain containing 1C
3881	CSTB	0.3008	0.2436	cystatin B (stefin B)
3882	ZNF618	0.3	0.121	zinc finger protein 618
3883	SGCB	0.3	0.2604	sarcoglycan, beta (43kDa dystrophin-associated glycoprotein)
3884	PNPLA2	0.3	0.3457	patatin-like phospholipase domain containing 2
3885	GALNTL4	0.3	0.0719	UDP-N-acetyl-alpha-D-galactosamine:polypeptide N-acetylgalactosaminyltransferase-like 4
3886	FAM135A	0.3	0.3061	family with sequence similarity 135, member A
3887	CCL11	0.3	0.2476	chemokine (C-C motif) ligand 11
3888	PBX2	0.2992	0.1035	pre-B-cell leukemia homeobox 2
3889	FCF1	0.2992	0.3751	FCF1 small subunit (SSU) processome component homolog (S. cerevisiae)
3890	SLC38A11	0.2988	0.0574	solute carrier family 38, member 11
3891	C9orf169	0.2986	0.2635	chromosome 9 open reading frame 169
3892	TFCP2	0.2983	0.3757	transcription factor CP2
3893	SEC62	0.2983	0.3984	SEC62 homolog (S. cerevisiae)
3894	MRPL49	0.2983	0.2803	mitochondrial ribosomal protein L49
3895	JAK1	0.2983	0.3308	Janus kinase 1
3896	FZD5	0.2983	0.0853	frizzled family receptor 5
3897	CYP3A7	0.2983	0.1497	cytochrome P450, family 3, subfamily A, polypeptide 7
3898	ATF1	0.2983	0.4075	activating transcription factor 1
3899	ZNF542	0.2975	0.2282	zinc finger protein 542
3900	ROS1	0.2975	0.2612	c-ros oncogene 1 , receptor tyrosine kinase
3901	MGEA5	0.2975	0.3463	meningioma expressed antigen 5 (hyaluronidase)
3902	INSL4	0.2975	0.2121	insulin-like 4 (placenta)
3903	CXCL2	0.2975	0.1375	chemokine (C-X-C motif) ligand 2
3904	BPHL	0.2975	0.1825	biphenyl hydrolase-like (serine hydrolase)
3905	ATP6V1G3	0.2975	0.0545	ATPase, H+ transporting, lysosomal 13kDa, V1 subunit G3
3906	ARAP1	0.2975	0.2702	ArfGAP with RhoGAP domain, ankyrin repeat and PH domain 1
3907	TMEM41B	0.2967	0.352	transmembrane protein 41B
3908	MED14	0.2967	0.3967	mediator complex subunit 14
3909	SLC2A12	0.2962	0.1099	solute carrier family 2 (facilitated glucose transporter), member 12
3910	DAB2IP	0.2962	0.1798	DAB2 interacting protein
3911	SLC2A4RG	0.2958	0.2174	SLC2A4 regulator
3912	SIGMAR1	0.2958	0.2703	sigma non-opioid intracellular receptor 1
3913	SETBP1	0.2958	0.1799	SET binding protein 1
3914	RHOQ	0.2958	0.2775	ras homolog family member Q
3915	PFN2	0.2958	0.2447	profilin 2
3916	OSBPL1A	0.2958	0.2244	oxysterol binding protein-like 1A
3917	G6PD	0.2958	0.2278	glucose-6-phosphate dehydrogenase
3918	CCNG1	0.2958	0.3654	cyclin G1
3919	BMP7	0.2958	0.266	bone morphogenetic protein 7
3920	BDNF	0.2958	0.0951	brain-derived neurotrophic factor
3921	MTCH1	0.295	0.2133	mitochondrial carrier 1
3922	KRT16	0.295	0.1474	keratin 16
3923	FUOM	0.295	0.1527	fucose mutarotase
3924	FAM13C	0.295	0.1226	family with sequence similarity 13, member C
3925	BET1	0.295	0.3793	blocked early in transport 1 homolog (S. cerevisiae)
3926	PDZD3	0.2942	0.3033	PDZ domain containing 3
3927	GMDS	0.2942	0.2175	GDP-mannose 4,6-dehydratase
3928	G0S2	0.2942	0.1162	G0/G1switch 2
3929	CXCL5	0.2942	0.0675	chemokine (C-X-C motif) ligand 5
3930	TRIQK	0.2937	0.3127	triple QxxK/R motif containing
3931	PCDHB14	0.2937	0.1706	protocadherin beta 14
3932	CNTN3	0.2937	0.1012	contactin 3 (plasmacytoma associated)
3933	C22orf32	0.2937	0.2264	chromosome 22 open reading frame 32
3934	AIFM2	0.2937	0.1196	apoptosis-inducing factor, mitochondrion-associated, 2
3935	ZNF154	0.2933	0.1155	zinc finger protein 154
3936	UBR2	0.2933	0.3043	ubiquitin protein ligase E3 component n-recognin 2
3937	KLHL20	0.2933	0.3654	kelch-like 20 (Drosophila)
3938	TDO2	0.2925	0.0868	tryptophan 2,3-dioxygenase
3939	C20orf194	0.2925	0.2583	chromosome 20 open reading frame 194
3940	ALOXE3	0.2925	0.3057	arachidonate lipoxygenase 3
3941	TRAF5	0.2917	0.2806	TNF receptor-associated factor 5
3942	SNCG	0.2917	0.2777	synuclein, gamma (breast cancer-specific protein 1)
3943	SEMA4G	0.2917	0.2853	sema domain, immunoglobulin domain (Ig), transmembrane domain (TM) and short cytoplasmic domain, (semaphorin) 4G
3944	SEC24A	0.2917	0.3685	SEC24 family, member A (S. cerevisiae)
3945	PPARD	0.2917	0.1726	peroxisome proliferator-activated receptor delta
3946	PDLIM3	0.2917	0.1062	PDZ and LIM domain 3

3947	P2RX6	0.2917	0.2895	purinergic receptor P2X, ligand-gated ion channel, 6
3948	NAMPT	0.2917	0.246	nicotinamide phosphoribosyltransferase
3949	SPPL3	0.2912	0.2529	signal peptide peptidase like 3
3950	SLC25A27	0.2912	0.1635	solute carrier family 25, member 27
3951	FGF10	0.2912	0.0824	fibroblast growth factor 10
3952	BAIAP2L1	0.2912	0.11	BAI1-associated protein 2-like 1
3953	TFEB	0.2908	0.26	transcription factor EB
3954	SPRY2	0.2908	0.2057	sprouty homolog 2 (Drosophila)
3955	MPDZ	0.2908	0.2334	multiple PDZ domain protein
3956	LCN1	0.2908	0.2146	lipocalin 1
3957	GAL3ST4	0.2908	0.1379	galactose-3-O-sulfotransferase 4
3958	DAZAP2	0.2908	0.3398	DAZ associated protein 2
3959	ADIPOR1	0.2908	0.2049	adiponectin receptor 1
3960	XPNPEP1	0.29	0.3265	X-prolyl aminopeptidase (aminopeptidase P) 1, soluble
3961	SLC35D1	0.29	0.2423	solute carrier family 35 (UDP-glucuronic acid/UDP-N-acetylgalactosamine dual transporter), member D1
3962	RAI2	0.29	0.1273	retinoic acid induced 2
3963	NRAP	0.29	0.2871	nebulin-related anchoring protein
3964	LOC729678	0.29	0.1304	uncharacterized LOC729678
3965	LOC390998	0.29	0	ribosomal protein L10 pseudogene
3966	IRAK4	0.29	0.2581	interleukin-1 receptor-associated kinase 4
3967	CDC42EP1	0.29	0.2297	CDC42 effector protein (Rho GTPase binding) 1
3968	C3orf33	0.29	0.2245	chromosome 3 open reading frame 33
3969	ADAMTS3	0.29	0.0783	ADAM metallopeptidase with thrombospondin type 1 motif, 3
3970	TFG	0.2892	0.3551	TRK-fused gene
3971	SLC38A7	0.2892	0.217	solute carrier family 38, member 7
3972	PMM2	0.2892	0.2602	phosphomannomutase 2
3973	MAFF	0.2892	0.1805	v-maf musculoaponeurotic fibrosarcoma oncogene homolog F (avian)
3974	ERF	0.2892	0.1684	Ets2 repressor factor
3975	EPHX3	0.2892	0.1951	epoxide hydrolase 3
3976	C21orf7	0.2892	0.1488	chromosome 21 open reading frame 7
3977	CABLES1	0.2887	0.0698	Cdk5 and Abl enzyme substrate 1
3978	STK32B	0.2883	0.0886	serine/threonine kinase 32B
3979	SEC22B	0.2883	0.3123	SEC22 vesicle trafficking protein homolog B (S. cerevisiae) (gene/pseudogene)
3980	PCGF2	0.2883	0.2134	polycomb group ring finger 2
3981	MPHOSPH8	0.2883	0.3327	M-phase phosphoprotein 8
3982	LHFPL2	0.2883	0.1837	lipoma HMGIC fusion partner-like 2
3983	GCHFR	0.2883	0.1604	GTP cyclohydrolase I feedback regulator
3984	DPY19L3	0.2883	0.4034	dpy-19-like 3 (C. elegans)
3985	ACTN2	0.2883	0.2669	actinin, alpha 2
3986	ZMYM6	0.2875	0.3599	zinc finger, MYM-type 6
3987	TDRD9	0.2875	0.0327	tudor domain containing 9
3988	TBL2	0.2875	0.2838	transducin (beta)-like 2
3989	OXA1L	0.2875	0.2811	oxidase (cytochrome c) assembly 1-like
3990	KLHL4	0.2875	0.1511	kelch-like 4 (Drosophila)
3991	KLHL14	0.2875	0.0489	kelch-like 14 (Drosophila)
3992	HS3ST1	0.2875	0.0552	heparan sulfate (glucosamine) 3-O-sulfotransferase 1
3993	GBA3	0.2875	0.1497	glucosidase, beta, acid 3 (cytosolic)
3994	DECR1	0.2875	0.332	2,4-dienoyl CoA reductase 1, mitochondrial
3995	CMTM7	0.2875	0.1472	CKLF-like MARVEL transmembrane domain containing 7
3996	CES4A	0.2875	0.2295	carboxylesterase 4A
3997	SLC7A7	0.2867	0.1794	solute carrier family 7 (amino acid transporter light chain, y+L system), member 7
3998	KRT12	0.2867	0.1821	keratin 12
3999	GGTLC1	0.2867	0.1833	gamma-glutamyltransferase light chain 1
4000	JOSD2	0.2862	0.2397	Josephin domain containing 2
4001	CYP4Z2P	0.2862	0.0138	cytochrome P450, family 4, subfamily Z, polypeptide 2 pseudogene
4002	CREB3L4	0.2862	0.1781	cAMP responsive element binding protein 3-like 4
4003	SULT1E1	0.2858	0.0819	sulfotransferase family 1E, estrogen-preferring, member 1
4004	PC	0.2858	0.187	pyruvate carboxylase
4005	TMEM212	0.2855	0.1769	transmembrane protein 212
4006	C1orf68	0.2855	0.2907	chromosome 1 open reading frame 68
4007	ZNF518B	0.285	0.3125	zinc finger protein 518B
4008	SCUBE2	0.285	0.1356	signal peptide, CUB domain, EGF-like 2
4009	PLA2G12A	0.285	0.21	phospholipase A2, group XIIA
4010	PIGM	0.285	0.351	phosphatidylinositol glycan anchor biosynthesis, class M
4011	KLK13	0.285	0.3178	kallikrein-related peptidase 13
4012	GJB5	0.285	0.2357	gap junction protein, beta 5, 31.1kDa
4013	CDR2	0.285	0.2992	cerebellar degeneration-related protein 2, 62kDa
4014	CCDC126	0.285	0.3587	coiled-coil domain containing 126
4015	CADM2	0.285	0.0786	cell adhesion molecule 2
4016	C14orf79	0.285	0.2569	chromosome 14 open reading frame 79
4017	BTN3A1	0.285	0.2101	butyrophilin, subfamily 3, member A1
4018	SMAD5-AS1	0.2842	0.1791	SMAD5 antisense RNA 1 (non-protein coding)
4019	PEX1	0.2842	0.4021	peroxisomal biogenesis factor 1
4020	FAF2	0.2842	0.3375	Fas associated factor family member 2
4021	DNALI1	0.2842	0.114	dynein, axonemal, light intermediate chain 1
4022	CD36	0.2842	0.1307	CD36 molecule (thrombospondin receptor)
4023	BRWD1	0.2842	0.3635	bromodomain and WD repeat domain containing 1
4024	ATP1B4	0.2842	0.126	ATPase, Na+/K+ transporting, beta 4 polypeptide
4025	GOPC	0.2837	0.4072	golgi-associated PDZ and coiled-coil motif containing
4026	GGTA1P	0.2837	0.1226	glycoprotein, alpha-galactosyltransferase 1 pseudogene
4027	CXorf38	0.2837	0.2837	chromosome X open reading frame 38
4028	TGM3	0.2833	0.2817	transglutaminase 3 (E polypeptide, protein-glutamine-gamma-glutamyltransferase)
4029	LACTB2	0.2833	0.3902	lactamase, beta 2
4030	DOLK	0.2833	0.2143	dolichol kinase

4031	C1orf115	0.2833	0.1014	chromosome 1 open reading frame 115
4032	BBOX1	0.2833	0.0747	butyrobetaine (gamma), 2-oxoglutarate dioxygenase (gamma-butyrobetaine hydroxylase) 1
4033	LOC158572	0.2829	0.0592	uncharacterized LOC158572
4034	ERVMER34-1	0.2827	0.1472	endogenous retrovirus group MER34, member 1
4035	WBSCR17	0.2825	0.1414	Williams-Beuren syndrome chromosome region 17
4036	TRIM13	0.2825	0.3314	tripartite motif containing 13
4037	SSC5D	0.2825	0.2733	scavenger receptor cysteine rich domain containing (5 domains)
4038	SRSF8	0.2825	0.349	serine/arginine-rich splicing factor 8
4039	SERINC3	0.2825	0.3296	serine incorporator 3
4040	RNF145	0.2825	0.3022	ring finger protein 145
4041	REG4	0.2825	0.134	regenerating islet-derived family, member 4
4042	IFI27L2	0.2825	0.1898	interferon, alpha-inducible protein 27-like 2
4043	FAM82B	0.2825	0.3925	family with sequence similarity 82, member B
4044	TMEM39A	0.2817	0.3084	transmembrane protein 39A
4045	ESD	0.2817	0.3738	esterase D
4046	ARL5A	0.2817	0.3985	ADP-ribosylation factor-like 5A
4047	OR10H3	0.2808	0.265	olfactory receptor, family 10, subfamily H, member 3
4048	DAPP1	0.2808	0.158	dual adaptor of phosphotyrosine and 3-phosphoinositides
4049	ADAM10	0.2808	0.3751	ADAM metallopeptidase domain 10
4050	ZNF449	0.28	0.1765	zinc finger protein 449
4051	TWISTNB	0.28	0.3359	TWIST neighbor
4052	SNX29	0.28	0.201	sorting nexin 29
4053	RARS2	0.28	0.4071	arginyl-tRNA synthetase 2, mitochondrial
4054	POR	0.28	0.2611	P450 (cytochrome) oxidoreductase
4055	PGBD3	0.28	0.2239	piggyBac transposable element derived 3
4056	NLRC5	0.28	0.1675	NLR family, CARD domain containing 5
4057	GTF2I	0.28	0.2682	general transcription factor IIi
4058	GADD45B	0.28	0.1789	growth arrest and DNA-damage-inducible, beta
4059	CORO6	0.28	0.1632	coronin 6
4060	ARIH1	0.28	0.3836	ariadne homolog, ubiquitin-conjugating enzyme E2 binding protein, 1 (Drosophila)
4061	APOL3	0.28	0.1798	apolipoprotein L, 3
4062	MACROD1	0.2792	0.2114	MACRO domain containing 1
4063	PINLYP	0.2791	0.115	phospholipase A2 inhibitor and LY6/PLAUR domain containing
4064	GRIP1	0.2791	0.1291	glutamate receptor interacting protein 1
4065	ST6GALNAC3	0.2788	0.0995	ST6 (alpha-N-acetyl-neuraminyl-2,3-beta-galactosyl-1,3)-N-acetylgalactosaminide alpha-2,6-sialyltransferase 3
4066	ZDHHC3	0.2783	0.1958	zinc finger, DHHC-type containing 3
4067	SPAG16	0.2783	0.225	sperm associated antigen 16
4068	PRSS3	0.2783	0.2322	protease, serine, 3
4069	LDB1	0.2783	0.2265	LIM domain binding 1
4070	KIAA0100	0.2783	0.29	KIAA0100
4071	ADRB2	0.2783	0.1073	adrenoceptor beta 2, surface
4072	PLN	0.2775	0.1563	phospholamban
4073	MSS51	0.2775	0.0773	MSS51 mitochondrial translational activator homolog (S. cerevisiae)
4074	HKDC1	0.2775	0.2202	hexokinase domain containing 1
4075	CSN1S1	0.2775	0.1101	casein alpha s1
4076	ATP9A	0.2775	0.217	ATPase, class II, type 9A
4077	SDHC	0.2773	0.3132	succinate dehydrogenase complex, subunit C, integral membrane protein, 15kDa
4078	ELN	0.2767	0.2919	elastin
4079	CSAD	0.2767	0.2113	cysteine sulfinic acid decarboxylase
4080	ZNF219	0.2758	0.3118	zinc finger protein 219
4081	RHOA	0.2758	0.3656	ras homolog family member A
4082	PCDHGA9	0.2758	0.2872	protocadherin gamma subfamily A, 9
4083	HTR7P1	0.2758	0.1162	5-hydroxytryptamine (serotonin) receptor 7 pseudogene 1
4084	IFNA1	0.2755	0.1849	interferon, alpha 1
4085	USP54	0.275	0.1395	ubiquitin specific peptidase 54
4086	SI	0.275	0.071	sucrase-isomaltase (alpha-glucosidase)
4087	MYOZ2	0.275	0.1956	myozenin 2
4088	MAP7	0.275	0.1367	microtubule-associated protein 7
4089	LOC339803	0.275	0.1332	uncharacterized LOC339803
4090	LCOR	0.275	0.3431	ligand dependent nuclear receptor corepressor
4091	HOXB3	0.275	0.1208	homeobox B3
4092	TNNI3K	0.2743	0.1271	TNNI3 interacting kinase
4093	MACF1	0.2742	0.3149	microtubule-actin crosslinking factor 1
4094	LGALS1	0.2742	0.2496	lectin, galactoside-binding, soluble, 1
4095	INTS3	0.2742	0.2323	integrator complex subunit 3
4096	NOXO1	0.2738	0.2303	NADPH oxidase organizer 1
4097	GALM	0.2738	0.1537	galactose mutarotase (aldose 1-epimerase)
4098	TNNT2	0.2733	0.1655	troponin T type 2 (cardiac)
4099	PTPRH	0.2733	0.2297	protein tyrosine phosphatase, receptor type, H
4100	MTMR12	0.2733	0.2626	myotubularin related protein 12
4101	LYVE1	0.2733	0.2071	lymphatic vessel endothelial hyaluronan receptor 1
4102	PNRC2	0.2727	0.2889	proline-rich nuclear receptor coactivator 2
4103	ZBTB7B	0.2725	0.3452	zinc finger and BTB domain containing 7B
4104	TPM4	0.2725	0.2147	tropomyosin 4
4105	TMEM184A	0.2725	0.2963	transmembrane protein 184A
4106	RASSF6	0.2725	0.0689	Ras association (RalGDS/AF-6) domain family member 6
4107	POMGNT1	0.2725	0.2172	protein O-linked mannose beta1,2-N-acetylglucosaminyltransferase
4108	METTL7B	0.2725	0.0923	methyltransferase like 7B
4109	KRT78	0.2725	0.1694	keratin 78
4110	DLG1	0.2725	0.4114	discs, large homolog 1 (Drosophila)
4111	ABHD13	0.2725	0.3803	abhydrolase domain containing 13
4112	TRIM21	0.2717	0.2075	tripartite motif containing 21
4113	RIN1	0.2717	0.2991	Ras and Rab interactor 1
4114	RB1	0.2717	0.3877	retinoblastoma 1

4115	ERBB2IP	0.2717	0.398	erbb2 interacting protein
4116	CUEDC1	0.2717	0.1706	CUE domain containing 1
4117	CLTB	0.2717	0.2589	clathrin, light chain B
4118	WNK4	0.2713	0.1463	WNK lysine deficient protein kinase 4
4119	MGARP	0.2713	0.0748	mitochondria-localized glutamic acid-rich protein
4120	IGDCC4	0.2713	0.0921	immunoglobulin superfamily, DCC subclass, member 4
4121	GPR115	0.2713	0.07	G protein-coupled receptor 115
4122	ATL1	0.2713	0.2442	atlastin GTPase 1
4123	ZNF862	0.2708	0.241	zinc finger protein 862
4124	SENP7	0.2708	0.3655	SUMO1/sentrin specific peptidase 7
4125	RHOBTB1	0.2708	0.1889	Rho-related BTB domain containing 1
4126	RAPGEF2	0.2708	0.3164	Rap guanine nucleotide exchange factor (GEF) 2
4127	MYO10	0.2708	0.1214	myosin X
4128	GATA2	0.2708	0.1643	GATA binding protein 2
4129	CHI3L1	0.2708	0.1251	chitinase 3-like 1 (cartilage glycoprotein-39)
4130	ZNF652	0.27	0.248	zinc finger protein 652
4131	PCK1	0.27	0.0651	phosphoenolpyruvate carboxykinase 1 (soluble)
4132	GLYAT	0.27	0.2979	glycine-N-acyltransferase
4133	EFHC2	0.27	0.1563	EF-hand domain (C-terminal) containing 2
4134	DKFZP434A062	0.27	0.3659	uncharacterized LOC26102
4135	CYP2U1	0.27	0.2416	cytochrome P450, family 2, subfamily U, polypeptide 1
4136	COL11A1	0.27	0.133	collagen, type XI, alpha 1
4137	C5orf44	0.27	0.4221	chromosome 5 open reading frame 44
4138	UBA7	0.2692	0.2508	ubiquitin-like modifier activating enzyme 7
4139	RABL3	0.2692	0.3303	RAB, member of RAS oncogene family-like 3
4140	LMF1	0.2692	0.2944	lipase maturation factor 1
4141	SHISA3	0.2688	0.0416	shisa homolog 3 (Xenopus laevis)
4142	NT5C3	0.2688	0.2762	5'-nucleotidase, cytosolic III
4143	LOC100130691	0.2688	0.118	uncharacterized LOC100130691
4144	SRL	0.2686	0.2716	sarcalumenin
4145	THY1	0.2683	0.2506	Thy-1 cell surface antigen
4146	KRT32	0.2683	0.2921	keratin 32
4147	ICK	0.2683	0.3883	intestinal cell (MAK-like) kinase
4148	GSTA3	0.2683	0.2035	glutathione S-transferase alpha 3
4149	CPS1-IT1	0.2683	0.2651	CPS1 intronic transcript 1 (non-protein coding)
4150	AQP2	0.2683	0.3204	aquaporin 2 (collecting duct)
4151	AKAP10	0.2683	0.3302	A kinase (PRKA) anchor protein 10
4152	WISP3	0.2675	0.0908	WNT1 inducible signaling pathway protein 3
4153	PRKD1	0.2675	0.2015	protein kinase D1
4154	PCNX	0.2675	0.3003	pecanex homolog (Drosophila)
4155	IQGAP2	0.2675	0.2042	IQ motif containing GTPase activating protein 2
4156	IL26	0.2675	0.0441	interleukin 26
4157	GPA33	0.2675	0.208	glycoprotein A33 (transmembrane)
4158	FABP3	0.2675	0.1116	fatty acid binding protein 3, muscle and heart (mammary-derived growth inhibitor)
4159	ANKRD35	0.2675	0.096	ankyrin repeat domain 35
4160	UFL1	0.2667	0.4113	UFM1-specific ligase 1
4161	CYTIP	0.2667	0.1639	cytohesin 1 interacting protein
4162	TMEM192	0.2663	0.3682	transmembrane protein 192
4163	MSANTD3	0.2663	0.2348	Myb/SANT-like DNA-binding domain containing 3
4164	CTBP1-AS1	0.2663	0.2374	CTBP1 antisense RNA 1 (non-protein coding)
4165	XDH	0.2658	0.2691	xanthine dehydrogenase
4166	TRAPPC3	0.2658	0.3213	trafficking protein particle complex 3
4167	TAS2R1	0.2658	0.2326	taste receptor, type 2, member 1
4168	SKIL	0.2658	0.232	SKI-like oncogene
4169	LGALS2	0.2658	0.1499	lectin, galactoside-binding, soluble, 2
4170	INSL5	0.2658	0.2541	insulin-like 5
4171	EDN2	0.2658	0.2761	endothelin 2
4172	TBC1D15	0.265	0.3765	TBC1 domain family, member 15
4173	PAX9	0.265	0.215	paired box 9
4174	METTL15	0.265	0.2541	methyltransferase like 15
4175	KRT85	0.265	0.3174	keratin 85
4176	HLA-DMA	0.265	0.2154	major histocompatibility complex, class II, DM alpha
4177	EID1	0.265	0.399	EP300 interacting inhibitor of differentiation 1
4178	EDIL3	0.265	0.1893	EGF-like repeats and discoidin I-like domains 3
4179	DERL1	0.265	0.4031	derlin 1
4180	CRCT1	0.265	0.2095	cysteine-rich C-terminal 1
4181	RIPK2	0.2642	0.2397	receptor-interacting serine-threonine kinase 2
4182	LCMT2	0.2642	0.3593	leucine carboxyl methyltransferase 2
4183	LARP6	0.2642	0.1655	La ribonucleoprotein domain family, member 6
4184	GSTM3	0.2642	0.1244	glutathione S-transferase mu 3 (brain)
4185	ATG16L1	0.2637	0.248	autophagy related 16-like 1 (S. cerevisiae)
4186	ARHGAP5-AS1	0.2637	0.088	ARHGAP5 antisense RNA 1 (non-protein coding)
4187	ZNF10	0.2633	0.2476	zinc finger protein 10
4188	WWOX	0.2633	0.1175	WW domain containing oxidoreductase
4189	SLC6A13	0.2633	0.3259	solute carrier family 6 (neurotransmitter transporter, GABA), member 13
4190	LY6G6C	0.2633	0.3072	lymphocyte antigen 6 complex, locus G6C
4191	C21orf62	0.2633	0.286	chromosome 21 open reading frame 62
4192	ALDH1B1	0.2633	0.2008	aldehyde dehydrogenase 1 family, member B1
4193	SLC30A4	0.2625	0.1622	solute carrier family 30 (zinc transporter), member 4
4194	PRDM13	0.2625	0.133	PR domain containing 13
4195	MAP7D3	0.2625	0.1474	MAP7 domain containing 3
4196	IFNA16	0.2625	0.0857	interferon, alpha 16
4197	GKN1	0.2625	0.2301	gastrokine 1
4198	FLNC	0.2625	0.2047	filamin C, gamma

4199	EPB41L5	0.2625	0.2234	erythrocyte membrane protein band 4.1 like 5
4200	CLEC3A	0.2625	0.1836	C-type lectin domain family 3, member A
4201	CDC42SE1	0.2625	0.1933	CDC42 small effector 1
4202	WNT5B	0.2617	0.163	wingless-type MMTV integration site family, member 5B
4203	THADA	0.2617	0.3605	thyroid adenoma associated
4204	STRA6	0.2617	0.3104	stimulated by retinoic acid gene 6 homolog (mouse)
4205	SOS1	0.2617	0.3268	son of sevenless homolog 1 (Drosophila)
4206	RPL4	0.2617	0.3437	ribosomal protein L4
4207	RPL13P5	0.2617	0.1034	ribosomal protein L13 pseudogene 5
4208	CD93	0.2617	0.1844	CD93 molecule
4209	AUP1	0.2617	0.2918	ancient ubiquitous protein 1
4210	SYPL2	0.2614	0.2848	synaptophysin-like 2
4211	PNPLA8	0.2612	0.3831	patatin-like phospholipase domain containing 8
4212	CHSY3	0.2612	0.1323	chondroitin sulfate synthase 3
4213	C16orf74	0.2612	0.167	chromosome 16 open reading frame 74
4214	NADSYN1	0.2608	0.2387	NAD synthetase 1
4215	KDM4A	0.2608	0.1815	lysine (K)-specific demethylase 4A
4216	AGFG2	0.2608	0.2403	ArfGAP with FG repeats 2
4217	RPL23	0.26	0.2706	ribosomal protein L23
4218	RORC	0.26	0.2748	RAR-related orphan receptor C
4219	PSORS1C2	0.26	0.2999	psoriasis susceptibility 1 candidate 2
4220	MAP3K6	0.26	0.2677	mitogen-activated protein kinase kinase kinase 6
4221	DENND2D	0.26	0.1736	DENN/MADD domain containing 2D
4222	CAT	0.26	0.3141	catalase
4223	VAPB	0.2592	0.2216	VAMP (vesicle-associated membrane protein)-associated protein B and C
4224	PPP6R3	0.2592	0.343	protein phosphatase 6, regulatory subunit 3
4225	PHKA1	0.2592	0.121	phosphorylase kinase, alpha 1 (muscle)
4226	ILVBL	0.2592	0.2504	ilvB (bacterial acetolactate synthase)-like
4227	HRASLS2	0.2592	0.2078	HRAS-like suppressor 2
4228	HBEGF	0.2592	0.1745	heparin-binding EGF-like growth factor
4229	HADH	0.2592	0.3519	hydroxyacyl-CoA dehydrogenase
4230	SNORA25	0.2591	0.2646	small nucleolar RNA, H/ACA box 25
4231	KRTAP9-4	0.2587	0.1408	keratin associated protein 9-4
4232	ARHGAP23	0.2587	0.2492	Rho GTPase activating protein 23
4233	UTRN	0.2583	0.2604	utrophin
4234	GPC3	0.2583	0.086	glypican 3
4235	CALML4	0.2583	0.1187	calmodulin-like 4
4236	TMEM120A	0.2575	0.2369	transmembrane protein 120A
4237	TECPR1	0.2575	0.2115	tectonin beta-propeller repeat containing 1
4238	STAG1	0.2575	0.4211	stromal antigen 1
4239	RAMP2	0.2575	0.2284	receptor (G protein-coupled) activity modifying protein 2
4240	PTGR2	0.2575	0.3219	prostaglandin reductase 2
4241	IRF2	0.2575	0.297	interferon regulatory factor 2
4242	SPCS3	0.2567	0.3639	signal peptidase complex subunit 3 homolog (S. cerevisiae)
4243	SIX5	0.2567	0.2884	SIX homeobox 5
4244	PRSS16	0.2567	0.0972	protease, serine, 16 (thymus)
4245	PCDHB13	0.2567	0.0869	protocadherin beta 13
4246	NIT2	0.2567	0.3592	nitrilase family, member 2
4247	IQCK	0.2567	0.1684	IQ motif containing K
4248	ABCA12	0.2567	0.0662	ATP-binding cassette, sub-family A (ABC1), member 12
4249	ZNF740	0.2562	0.1254	zinc finger protein 740
4250	GORAB	0.2562	0.4013	golgin, RAB6-interacting
4251	EPDR1	0.2562	0.1664	ependymin related protein 1 (zebrafish)
4252	COX18	0.2562	0.3666	COX18 cytochrome c oxidase assembly homolog (S. cerevisiae)
4253	CHRAC1	0.2562	0.2567	chromatin accessibility complex 1
4254	NRP2	0.2558	0.274	neuropilin 2
4255	EHD3	0.2558	0.1489	EH-domain containing 3
4256	C1GALT1	0.2558	0.3382	core 1 synthase, glycoprotein-N-acetylgalactosamine 3-beta-galactosyltransferase, 1
4257	CALCB	0.2555	0.1624	calcitonin-related polypeptide beta
4258	ZNF345	0.255	0.196	zinc finger protein 345
4259	SERTAD2	0.255	0.2971	SERTA domain containing 2
4260	PON2	0.255	0.365	paraoxonase 2
4261	MOB1B	0.255	0.4122	MOB kinase activator 1B
4262	IFNA5	0.255	0.1979	interferon, alpha 5
4263	FZD2	0.255	0.1688	frizzled family receptor 2
4264	FTL	0.255	0.2511	ferritin, light polypeptide
4265	CERCAM	0.255	0.2439	cerebral endothelial cell adhesion molecule
4266	CCDC111	0.255	0.4052	coiled-coil domain containing 111
4267	SLC16A3	0.2542	0.2212	solute carrier family 16, member 3 (monocarboxylic acid transporter 4)
4268	RAB11FIP5	0.2542	0.2062	RAB11 family interacting protein 5 (class I)
4269	PER2	0.2542	0.1648	period homolog 2 (Drosophila)
4270	ISG20	0.2542	0.1774	interferon stimulated exonuclease gene 20kDa
4271	FOSL1	0.2542	0.1906	FOS-like antigen 1
4272	ENGASE	0.2542	0.2394	endo-beta-N-acetylglucosaminidase
4273	APOBEC1	0.2542	0.2515	apolipoprotein B mRNA editing enzyme, catalytic polypeptide 1
4274	ACP5	0.2542	0.1519	acid phosphatase 5, tartrate resistant
4275	SLC52A3	0.2537	0.2656	solute carrier family 52, riboflavin transporter, member 3
4276	CD276	0.2537	0.2321	CD276 molecule
4277	THUMPD2	0.2533	0.3722	THUMP domain containing 2
4278	RAB5B	0.2533	0.2977	RAB5B, member RAS oncogene family
4279	MGST3	0.2533	0.2876	microsomal glutathione S-transferase 3
4280	IL3	0.2533	0.243	interleukin 3 (colony-stimulating factor, multiple)
4281	CLU	0.2533	0.1788	clusterin
4282	FLJ90757	0.2529	0.1579	uncharacterized LOC440465

4283	MAP1LC3C	0.2527	0.1675	microtubule-associated protein 1 light chain 3 gamma
4284	ZXDA	0.2525	0.0719	zinc finger, X-linked, duplicated A
4285	ZNF565	0.2525	0.295	zinc finger protein 565
4286	SULT1B1	0.2525	0.1774	sulfotransferase family, cytosolic, 1B, member 1
4287	PRX	0.2525	0.3417	periaxin
4288	NBLA00301	0.2525	0.0699	Nbla00301
4289	LOC728613	0.2525	0.0333	programmed cell death 6 pseudogene
4290	HHLA2	0.2525	0.0967	HERV-H LTR-associating 2
4291	GXYLT1	0.2525	0.4032	glucoside xylosyltransferase 1
4292	ARAP3	0.2525	0.1615	ArfGAP with RhoGAP domain, ankyrin repeat and PH domain 3
4293	C7orf69	0.2517	0.2696	chromosome 7 open reading frame 69
4294	LOC100287896	0.2514	0.3666	uncharacterized LOC100287896
4295	MPP4	0.2512	0.04	membrane protein, palmitoylated 4 (MAGUK p55 subfamily member 4)
4296	MGC39372	0.2512	0.0873	serpin peptidase inhibitor, clade B (ovalbumin), member 9 pseudogene
4297	S100A8	0.2508	0.1455	S100 calcium binding protein A8
4298	MYOC	0.2508	0.2405	myocilin, trabecular meshwork inducible glucocorticoid response
4299	KIAA0430	0.2508	0.3615	KIAA0430
4300	EDNRB	0.2508	0.1464	endothelin receptor type B
4301	ATF3	0.2508	0.1424	activating transcription factor 3
4302	ACVR1B	0.2508	0.2136	activin A receptor, type IB
4303	ZFP2	0.25	0.1983	zinc finger protein 2 homolog (mouse)
4304	TMEM51	0.25	0.1369	transmembrane protein 51
4305	SLITRK4	0.25	0.0442	SLIT and NTRK-like family, member 4
4306	PPIL4	0.25	0.4262	peptidylprolyl isomerase (cyclophilin)-like 4
4307	NDFIP1	0.25	0.381	Nedd4 family interacting protein 1
4308	M6PR	0.25	0.3493	mannose-6-phosphate receptor (cation dependent)
4309	LARP4B	0.25	0.3595	La ribonucleoprotein domain family, member 4B
4310	HOXC11	0.25	0.2322	homeobox C11
4311	GPC1	0.25	0.2801	glypican 1
4312	DECR2	0.25	0.2041	2,4-dienoyl CoA reductase 2, peroxisomal
4313	CYP7B1	0.25	0.1141	cytochrome P450, family 7, subfamily B, polypeptide 1
4314	COPG1	0.25	0.2907	coatomer protein complex, subunit gamma 1
4315	BBS9	0.25	0.3397	Bardet-Biedl syndrome 9
4316	POLI	0.2492	0.3868	polymerase (DNA directed) iota
4317	HUS1	0.2492	0.3077	HUS1 checkpoint homolog (S. pombe)
4318	ERCC1	0.2492	0.2466	excision repair cross-complementing rodent repair deficiency, complementation group 1 (includes overlapping antisense sequence)
4319	CTSF	0.2492	0.2426	cathepsin F
4320	CALCR	0.2492	0.2467	calcitonin receptor
4321	CMTM3	0.2488	0.2253	CKLF-like MARVEL transmembrane domain containing 3
4322	ZSCAN30	0.2483	0	zinc finger and SCAN domain containing 30
4323	SRD5A3	0.2483	0.1884	steroid 5 alpha-reductase 3
4324	PCDH12	0.2483	0.2819	protocadherin 12
4325	LHCGR	0.2483	0.191	luteinizing hormone/choriogonadotropin receptor
4326	PRDM9	0.2475	0.2763	PR domain containing 9
4327	PPP1R3A	0.2475	0.1357	protein phosphatase 1, regulatory subunit 3A
4328	GPRC5D	0.2475	0.2845	G protein-coupled receptor, family C, group 5, member D
4329	FAM213A	0.2475	0.2087	family with sequence similarity 213, member A
4330	STRN3	0.2467	0.3708	striatin, calmodulin binding protein 3
4331	SLC34A1	0.2467	0.2821	solute carrier family 34 (sodium phosphate), member 1
4332	RBM12B	0.2467	0.3811	RNA binding motif protein 12B
4333	PIK3C2B	0.2467	0.1854	phosphatidylinositol-4-phosphate 3-kinase, catalytic subunit type 2 beta
4334	NEB	0.2467	0.0733	nebulin
4335	KDM5C	0.2467	0.2064	lysine (K)-specific demethylase 5C
4336	IQCH	0.2467	0.112	IQ motif containing H
4337	IFI44	0.2467	0.249	interferon-induced protein 44
4338	CAPZA2	0.2467	0.4157	capping protein (actin filament) muscle Z-line, alpha 2
4339	PLBD2	0.2463	0.2341	phospholipase B domain containing 2
4340	CDNF	0.2463	0.1064	cerebral dopamine neurotrophic factor
4341	SMARCA2	0.2458	0.3701	SWI/SNF related, matrix associated, actin dependent regulator of chromatin, subfamily a, member 2
4342	SLC35C2	0.2458	0.2538	solute carrier family 35, member C2
4343	PLEKHG3	0.2458	0.2122	pleckstrin homology domain containing, family G (with RhoGef domain) member 3
4344	PDE8A	0.2458	0.3052	phosphodiesterase 8A
4345	OPN3	0.2458	0.2695	opsin 3
4346	NR2E3	0.2458	0.3242	nuclear receptor subfamily 2, group E, member 3
4347	NPY6R	0.2458	0.2922	neuropeptide Y receptor Y6 (pseudogene)
4348	EPHA4	0.2458	0.1451	EPH receptor A4
4349	ENO3	0.2458	0.1332	enolase 3 (beta, muscle)
4350	DKFZP586I1420	0.2458	0.2576	uncharacterized protein DKFZp586I1420
4351	DIXDC1	0.2458	0.1557	DIX domain containing 1
4352	CLCN3	0.2458	0.4015	chloride channel, voltage-sensitive 3
4353	SNHG9	0.2457	0.0004	small nucleolar RNA host gene 9 (non-protein coding)
4354	SIK2	0.245	0.1875	salt-inducible kinase 2
4355	SFT2D1	0.245	0.3875	SFT2 domain containing 1
4356	NQO2	0.245	0.245	NAD(P)H dehydrogenase, quinone 2
4357	KLHL24	0.245	0.3246	kelch-like 24 (Drosophila)
4358	OR2J3	0.2445	0.2492	olfactory receptor, family 2, subfamily J, member 3
4359	TRIM15	0.2442	0.3381	tripartite motif containing 15
4360	SPRY4	0.2442	0.1447	sprouty homolog 4 (Drosophila)
4361	RBM7	0.2442	0.414	RNA binding motif protein 7
4362	NIPA2	0.2442	0.3975	non imprinted in Prader-Willi/Angelman syndrome 2
4363	FRS2	0.2442	0.2098	fibroblast growth factor receptor substrate 2
4364	DTX4	0.2442	0.1059	deltex homolog 4 (Drosophila)
4365	CPEB3	0.2442	0.2873	cytoplasmic polyadenylation element binding protein 3
4366	CPA3	0.2442	0.1593	carboxypeptidase A3 (mast cell)

4367	WSCD2	0.2438	0.2611	WSC domain containing 2
4368	MCU	0.2438	0.2853	mitochondrial calcium uniporter
4369	ZNF44	0.2433	0.2868	zinc finger protein 44
4370	NFX1	0.2433	0.3027	nuclear transcription factor, X-box binding 1
4371	ABO	0.2433	0.314	ABO blood group (transferase A, alpha 1-3-N-acetylgalactosaminyltransferase; transferase B, alpha 1-3-galactosyltransferase)
4372	UNC119B	0.2425	0.3449	unc-119 homolog B (C. elegans)
4373	TMEM33	0.2425	0.3989	transmembrane protein 33
4374	TECRL	0.2425	0.0233	trans-2,3-enoyl-CoA reductase-like
4375	TBC1D8	0.2425	0.1632	TBC1 domain family, member 8 (with GRAM domain)
4376	PPP1R12A	0.2425	0.4163	protein phosphatase 1, regulatory subunit 12A
4377	MT1F	0.2425	0.0965	metallothionein 1F
4378	MAP3K13	0.2425	0.1413	mitogen-activated protein kinase kinase kinase 13
4379	ILDR1	0.2425	0.1663	immunoglobulin-like domain containing receptor 1
4380	FAM82A2	0.2425	0.1717	family with sequence similarity 82, member A2
4381	CRB3	0.2425	0.2562	crumbs homolog 3 (Drosophila)
4382	AKR1C4	0.2425	0.0917	aldo-keto reductase family 1, member C4 (chlordecone reductase; 3-alpha hydroxysteroid dehydrogenase, type I; dihydrodiol dehydrogenase 4)
4383	ACTRT3	0.2425	0.0548	actin-related protein T3
4384	SLC9A3R1	0.2417	0.1994	solute carrier family 9, subfamily A (NHE3, cation proton antiporter 3), member 3 regulator 1
4385	PER1	0.2417	0.2844	period homolog 1 (Drosophila)
4386	KERA	0.2417	0.1128	keratocan
4387	ARHGEF15	0.2417	0.3289	Rho guanine nucleotide exchange factor (GEF) 15
4388	MIRLET7BHG	0.2412	0.2111	MIRLET7B host gene (non-protein coding)
4389	ENPP5	0.2412	0.2244	ectonucleotide pyrophosphatase/phosphodiesterase 5 (putative)
4390	CYP2S1	0.2412	0.1797	cytochrome P450, family 2, subfamily S, polypeptide 1
4391	C12orf61	0.2412	0.0649	chromosome 12 open reading frame 61
4392	SLC34A2	0.2408	0.1321	solute carrier family 34 (sodium phosphate), member 2
4393	PTPLA	0.2408	0.2089	protein tyrosine phosphatase-like (proline instead of catalytic arginine), member A
4394	PDHA1	0.2408	0.3709	pyruvate dehydrogenase (lipoamide) alpha 1
4395	PAPPA2	0.2408	0.2918	pappalysin 2
4396	KLRC4	0.2408	0.0748	killer cell lectin-like receptor subfamily C, member 4
4397	FUT4	0.2408	0.0997	fucosyltransferase 4 (alpha (1,3) fucosyltransferase, myeloid-specific)
4398	CHRM2	0.2408	0.2777	cholinergic receptor, muscarinic 2
4399	ZNF524	0.24	0.301	zinc finger protein 524
4400	ZNF14	0.24	0.3052	zinc finger protein 14
4401	UTP20	0.24	0.2862	UTP20, small subunit (SSU) processome component, homolog (yeast)
4402	TRIM45	0.24	0.1585	tripartite motif containing 45
4403	TNIP1	0.24	0.2527	TNFAIP3 interacting protein 1
4404	TMEM107	0.24	0.2233	transmembrane protein 107
4405	SPTLC2	0.24	0.2975	serine palmitoyltransferase, long chain base subunit 2
4406	SLC38A1	0.24	0.3432	solute carrier family 38, member 1
4407	PNPLA1	0.24	0.0894	patatin-like phospholipase domain containing 1
4408	CYP19A1	0.24	0.2514	cytochrome P450, family 19, subfamily A, polypeptide 1
4409	GCNT1	0.2392	0.1143	glucosaminyl (N-acetyl) transferase 1, core 2
4410	CTPS2	0.2392	0.3356	CTP synthase 2
4411	AK1	0.2392	0.2084	adenylate kinase 1
4412	ABCC4	0.2392	0.2724	ATP-binding cassette, sub-family C (CFTR/MRP), member 4
4413	TMEM200B	0.2387	0.0711	transmembrane protein 200B
4414	STXBP5	0.2387	0.3862	syntaxin binding protein 5 (tomosyn)
4415	LOC149134	0.2387	0.1837	uncharacterized LOC149134
4416	LMOD3	0.2387	0.1448	leiomodin 3 (fetal)
4417	FBXL13	0.2387	0.0715	F-box and leucine-rich repeat protein 13
4418	ZNF443	0.2386	0.3313	zinc finger protein 443
4419	ZNF137P	0.2383	0.3031	zinc finger protein 137, pseudogene
4420	ZMYM1	0.2383	0.4144	zinc finger, MYM-type 1
4421	SRD5A1	0.2383	0.212	steroid-5-alpha-reductase, alpha polypeptide 1 (3-oxo-5 alpha-steroid delta 4-dehydrogenase alpha 1)
4422	SLC1A5	0.2383	0.2326	solute carrier family 1 (neutral amino acid transporter), member 5
4423	NAT8	0.2383	0.2216	N-acetyltransferase 8 (GCN5-related, putative)
4424	MYH6	0.2383	0.2014	myosin, heavy chain 6, cardiac muscle, alpha
4425	G6PC3	0.2383	0.2755	glucose 6 phosphatase, catalytic, 3
4426	CASP8	0.2383	0.2127	caspase 8, apoptosis-related cysteine peptidase
4427	BLCAP	0.2383	0.248	bladder cancer associated protein
4428	ZSWIM5	0.2375	0.1357	zinc finger, SWIM-type containing 5
4429	TAS2R14	0.2375	0.1672	taste receptor, type 2, member 14
4430	STX6	0.2375	0.3643	syntaxin 6
4431	KIAA1430	0.2375	0.4188	KIAA1430
4432	HSF4	0.2375	0.3514	heat shock transcription factor 4
4433	GALT	0.2375	0.2336	galactose-1-phosphate uridylyltransferase
4434	FBXO28	0.2375	0.4324	F-box protein 28
4435	BATF2	0.2375	0.1732	basic leucine zipper transcription factor, ATF-like 2
4436	KCNT2	0.2371	0.1714	potassium channel, subfamily T, member 2
4437	SCAMP2	0.2367	0.2669	secretory carrier membrane protein 2
4438	PTRH1	0.2362	0.2483	peptidyl-tRNA hydrolase 1 homolog (S. cerevisiae)
4439	POC1B	0.2362	0.4092	POC1 centriolar protein homolog B (Chlamydomonas)
4440	NUDT14	0.2362	0.2339	nudix (nucleoside diphosphate linked moiety X)-type motif 14
4441	LOC440335	0.2362	0.1896	uncharacterized LOC440335
4442	FAM43A	0.2362	0.1377	family with sequence similarity 43, member A
4443	CBLN3	0.2362	0.1817	cerebellin 3 precursor
4444	ARID1B	0.2362	0.1924	AT rich interactive domain 1B (SWI1-like)
4445	VMP1	0.2358	0.369	vacuole membrane protein 1
4446	TRPC1	0.2358	0.3195	transient receptor potential cation channel, subfamily C, member 1
4447	RP2	0.2358	0.3974	retinitis pigmentosa 2 (X-linked recessive)
4448	RBKS	0.2358	0.1732	ribokinase
4449	PELI2	0.2358	0.2279	pellino E3 ubiquitin protein ligase family member 2
4450	GPR39	0.2358	0.2181	G protein-coupled receptor 39

4451	FAM190B	0.2358	0.3599	family with sequence similarity 190, member B
4452	SLC27A2	0.235	0.1452	solute carrier family 27 (fatty acid transporter), member 2
4453	FTO	0.235	0.3745	fat mass and obesity associated
4454	AMMECR1	0.235	0.3296	Alport syndrome, mental retardation, midface hypoplasia and elliptocytosis chromosomal region gene 1
4455	SAA4	0.2343	0.1452	serum amyloid A4, constitutive
4456	SLC14A1	0.2342	0.0762	solute carrier family 14 (urea transporter), member 1 (Kidd blood group)
4457	MANF	0.2342	0.2627	mesencephalic astrocyte-derived neurotrophic factor
4458	KLK6	0.2342	0.2228	kallikrein-related peptidase 6
4459	CCL20	0.2342	0.1361	chemokine (C-C motif) ligand 20
4460	OSCP1	0.2338	0.1657	organic solute carrier partner 1
4461	XPNPEP3	0.2333	0.1544	X-prolyl aminopeptidase (aminopeptidase P) 3, putative
4462	MMP27	0.2333	0.1969	matrix metallopeptidase 27
4463	FBXO9	0.2333	0.3331	F-box protein 9
4464	FABP2	0.2333	0.198	fatty acid binding protein 2, intestinal
4465	ATP2B4	0.2333	0.2611	ATPase, Ca++ transporting, plasma membrane 4
4466	TMEM106C	0.2325	0.2864	transmembrane protein 106C
4467	LRIG2	0.2325	0.2303	leucine-rich repeats and immunoglobulin-like domains 2
4468	DMGDH	0.2325	0.0849	dimethylglycine dehydrogenase
4469	CCDC30	0.2325	0.1269	coiled-coil domain containing 30
4470	VAMP4	0.2317	0.405	vesicle-associated membrane protein 4
4471	UFC1	0.2317	0.3609	ubiquitin-fold modifier conjugating enzyme 1
4472	SOX17	0.2317	0.2198	SRY (sex determining region Y)-box 17
4473	RAD51D	0.2317	0.1725	RAD51 homolog D (S. cerevisiae)
4474	GABRR2	0.2317	0.3083	gamma-aminobutyric acid (GABA) A receptor, rho 2
4475	ARSE	0.2317	0.2141	arylsulfatase E (chondrodysplasia punctata 1)
4476	MT1H	0.2314	0.0865	metallothionein 1H
4477	ZNF502	0.2313	0.1244	zinc finger protein 502
4478	SDSL	0.2313	0.1886	serine dehydratase-like
4479	LACC1	0.2313	0.2787	laccase (multicopper oxidoreductase) domain containing 1
4480	CHD6	0.2313	0.3199	chromodomain helicase DNA binding protein 6
4481	SLC52A1	0.2308	0.251	solute carrier family 52, riboflavin transporter, member 1
4482	SFT2D2	0.2308	0.284	SFT2 domain containing 2
4483	OR2B2	0.2308	0.1835	olfactory receptor, family 2, subfamily B, member 2
4484	NDUFS1	0.2308	0.4163	NADH dehydrogenase (ubiquinone) Fe-S protein 1, 75kDa (NADH-coenzyme Q reductase)
4485	ATG4A	0.2308	0.3452	autophagy related 4A, cysteine peptidase
4486	ZNF320	0.23	0.3825	zinc finger protein 320
4487	ZFP112	0.23	0.3595	zinc finger protein 112 homolog (mouse)
4488	TAOK1	0.23	0.3525	TAO kinase 1
4489	SPARCL1	0.23	0.1819	SPARC-like 1 (hevin)
4490	PTH1R	0.23	0.3202	parathyroid hormone 1 receptor
4491	OBSL1	0.23	0.2244	obscurin-like 1
4492	NUB1	0.23	0.3135	negative regulator of ubiquitin-like proteins 1
4493	NEXN-AS1	0.23	0.043	NEXN antisense RNA 1 (non-protein coding)
4494	GCOM1	0.23	0.0979	GRINL1A complex locus 1
4495	ADPRM	0.23	0.2821	ADP-ribose/CDP-alcohol diphosphatase, manganese-dependent
4496	ZBTB7A	0.2292	0.2501	zinc finger and BTB domain containing 7A
4497	SMAD2	0.2292	0.4401	SMAD family member 2
4498	SLC28A1	0.2292	0.3427	solute carrier family 28 (sodium-coupled nucleoside transporter), member 1
4499	SLC12A9	0.2292	0.3136	solute carrier family 12 (potassium/chloride transporters), member 9
4500	SFTPD	0.2292	0.2803	surfactant protein D
4501	PCBD1	0.2292	0.2686	pterin-4 alpha-carbinolamine dehydratase/dimerization cofactor of hepatocyte nuclear factor 1 alpha
4502	ITPA	0.2292	0.2612	inosine triphosphatase (nucleoside triphosphate pyrophosphatase)
4503	ELAC1	0.2292	0.232	elaC homolog 1 (E. coli)
4504	CCNL2	0.2292	0.2343	cyclin L2
4505	LINC00476	0.2288	0.0847	long intergenic non-protein coding RNA 476
4506	IL20RB	0.2288	0.0617	interleukin 20 receptor beta
4507	GPR180	0.2288	0.3927	G protein-coupled receptor 180
4508	AIG1	0.2288	0.318	androgen-induced 1
4509	UPK3B	0.2283	0.2832	uroplakin 3B
4510	UMOD	0.2283	0.29	uromodulin
4511	SLC25A20	0.2283	0.2592	solute carrier family 25 (carnitine/acylcarnitine translocase), member 20
4512	RAB30	0.2283	0.1683	RAB30, member RAS oncogene family
4513	PLAG1	0.2283	0.2304	pleiomorphic adenoma gene 1
4514	OLFM4	0.2283	0.0382	olfactomedin 4
4515	PIGK	0.2275	0.436	phosphatidylinositol glycan anchor biosynthesis, class K
4516	MMP28	0.2275	0.3197	matrix metallopeptidase 28
4517	LRRK1	0.2275	0.1426	leucine-rich repeat kinase 1
4518	KLHDC9	0.2275	0.1559	kelch domain containing 9
4519	KIF12	0.2275	0.2738	kinesin family member 12
4520	JAM2	0.2275	0.2053	junctional adhesion molecule 2
4521	GNB5	0.2275	0.1981	guanine nucleotide binding protein (G protein), beta 5
4522	DMP1	0.2275	0.2226	dentin matrix acidic phosphoprotein 1
4523	CCDC121	0.2275	0.1832	coiled-coil domain containing 121
4524	C2orf70	0.2275	0.2074	chromosome 2 open reading frame 70
4525	AMMECR1L	0.2275	0.4173	AMME chromosomal region gene 1-like
4526	ADAM28	0.2275	0.1749	ADAM metallopeptidase domain 28
4527	TAF2	0.2267	0.4101	TAF2 RNA polymerase II, TATA box binding protein (TBP)-associated factor, 150kDa
4528	SERPIND1	0.2267	0.1897	serpin peptidase inhibitor, clade D (heparin cofactor), member 1
4529	NIPBL	0.2267	0.405	Nipped-B homolog (Drosophila)
4530	MYL10	0.2267	0.3005	myosin, light chain 10, regulatory
4531	TBX22	0.2262	0.0623	T-box 22
4532	DNAH5	0.2262	0.0939	dynein, axonemal, heavy chain 5
4533	SCRN3	0.2258	0.4123	secernin 3
4534	MSRA	0.2258	0.1596	methionine sulfoxide reductase A

4535	CDH17	0.2258	0.1836	cadherin 17, LI cadherin (liver-intestine)
4536	NAALADL2	0.2257	0.1569	N-acetylated alpha-linked acidic dipeptidase-like 2
4537	ZBTB1	0.225	0.3997	zinc finger and BTB domain containing 1
4538	SPIN3	0.225	0.2354	spindlin family, member 3
4539	SCRN2	0.225	0.2465	secernin 2
4540	RELA	0.225	0.2751	v-rel reticuloendotheliosis viral oncogene homolog A (avian)
4541	PTCD2	0.225	0.4172	pentatricopeptide repeat domain 2
4542	PCDHB5	0.225	0.0917	protocadherin beta 5
4543	FAM155B	0.225	0.2756	family with sequence similarity 155, member B
4544	DYSF	0.225	0.2524	dysferlin, limb girdle muscular dystrophy 2B (autosomal recessive)
4545	CAPS	0.225	0.2821	calcyphosine
4546	C12orf26	0.225	0.3617	chromosome 12 open reading frame 26
4547	SIDT2	0.2242	0.2715	SID1 transmembrane family, member 2
4548	PLEKHA4	0.2242	0.2753	pleckstrin homology domain containing, family A (phosphoinositide binding specific) member 4
4549	NAALADL1	0.2242	0.2993	N-acetylated alpha-linked acidic dipeptidase-like 1
4550	ISCU	0.2242	0.3572	iron-sulfur cluster scaffold homolog (E. coli)
4551	DHDDS	0.2242	0.2212	dehydrodolichyl diphosphate synthase
4552	DDX58	0.2242	0.2703	DEAD (Asp-Glu-Ala-Asp) box polypeptide 58
4553	C11orf24	0.2242	0.2893	chromosome 11 open reading frame 24
4554	ATP12A	0.2242	0.3036	ATPase, H+/K+ transporting, nongastric, alpha polypeptide
4555	ALX3	0.2242	0.2745	ALX homeobox 3
4556	UBN2	0.2237	0.2831	ubinuclein 2
4557	TMEM175	0.2237	0.2621	transmembrane protein 175
4558	TMEM154	0.2237	0.1963	transmembrane protein 154
4559	THAP6	0.2237	0.4038	THAP domain containing 6
4560	EPG5	0.2237	0.2816	ectopic P-granules autophagy protein 5 homolog (C. elegans)
4561	CLEC14A	0.2237	0.2606	C-type lectin domain family 14, member A
4562	ALG1	0.2237	0.2336	asparagine-linked glycosylation 1, beta-1,4-mannosyltransferase homolog (S. cerevisiae)
4563	SREK1	0.2233	0.3992	splicing regulatory glutamine/lysine-rich protein 1
4564	RNASET2	0.2233	0.253	ribonuclease T2
4565	KIAA0146	0.2233	0.3857	KIAA0146
4566	GLT8D1	0.2233	0.429	glycosyltransferase 8 domain containing 1
4567	FABP1	0.2233	0.186	fatty acid binding protein 1, liver
4568	WDR17	0.2225	0.1616	WD repeat domain 17
4569	TUG1	0.2225	0.3879	taurine upregulated 1 (non-protein coding)
4570	SYNGR2	0.2225	0.2667	synaptogyrin 2
4571	SCARNA17	0.2225	0.1501	small Cajal body-specific RNA 17
4572	NGF	0.2225	0.3126	nerve growth factor (beta polypeptide)
4573	IL7R	0.2225	0.1942	interleukin 7 receptor
4574	IAH1	0.2225	0.3328	isoamyl acetate-hydrolyzing esterase 1 homolog (S. cerevisiae)
4575	GPAA1	0.2225	0.295	glycosylphosphatidylinositol anchor attachment protein 1 homolog (yeast)
4576	CDH3	0.2225	0.157	cadherin 3, type 1, P-cadherin (placental)
4577	ABHD2	0.2225	0.2458	abhydrolase domain containing 2
4578	STEAP3	0.2217	0.1617	STEAP family member 3, metalloreductase
4579	PTX3	0.2217	0.1769	pentraxin 3, long
4580	KRT75	0.2217	0.3006	keratin 75
4581	FOXF2	0.2217	0.1637	forkhead box F2
4582	EMILIN1	0.2217	0.2547	elastin microfibril interfacer 1
4583	CNPY2	0.2217	0.3601	canopy 2 homolog (zebrafish)
4584	C22orf26	0.2217	0.3465	chromosome 22 open reading frame 26
4585	ADAM19	0.2217	0.203	ADAM metallopeptidase domain 19
4586	LOC285095	0.2214	0	uncharacterized LOC285095
4587	CDK3	0.2214	0.2158	cyclin-dependent kinase 3
4588	SLMAP	0.2212	0.4292	sarcolemma associated protein
4589	PAG1	0.2212	0.2546	phosphoprotein associated with glycosphingolipid microdomains 1
4590	IRX3	0.2212	0.0649	iroquois homeobox 3
4591	RNF13	0.2208	0.35	ring finger protein 13
4592	PARP8	0.2208	0.2671	poly (ADP-ribose) polymerase family, member 8
4593	NMI	0.2208	0.3834	N-myc (and STAT) interactor
4594	IQCE	0.2208	0.1755	IQ motif containing E
4595	CRELD2	0.2208	0.2383	cysteine-rich with EGF-like domains 2
4596	TEX9	0.22	0.3312	testis expressed 9
4597	RNF43	0.22	0.192	ring finger protein 43
4598	PECAM1	0.22	0.2116	platelet/endothelial cell adhesion molecule 1
4599	LOC401321	0.22	0	uncharacterized LOC401321
4600	ARHGEF10	0.22	0.2042	Rho guanine nucleotide exchange factor (GEF) 10
4601	SERPINE2	0.2192	0.216	serpin peptidase inhibitor, clade E (nexin, plasminogen activator inhibitor type 1), member 2
4602	MYH8	0.2192	0.2937	myosin, heavy chain 8, skeletal muscle, perinatal
4603	ARFIP2	0.2192	0.2633	ADP-ribosylation factor interacting protein 2
4604	SERPINA12	0.2188	0.1935	serpin peptidase inhibitor, clade A (alpha-1 antiproteinase, antitrypsin), member 12
4605	PACRGL	0.2188	0.4307	PARK2 co-regulated-like
4606	CD99L2	0.2188	0.2556	CD99 molecule-like 2
4607	LOC388387	0.2186	0.299	uncharacterized LOC388387
4608	SOS2	0.2183	0.3606	son of sevenless homolog 2 (Drosophila)
4609	RUNX1T1	0.2183	0.2082	runt-related transcription factor 1; translocated to, 1 (cyclin D-related)
4610	PTGER1	0.2183	0.3616	prostaglandin E receptor 1 (subtype EP1), 42kDa
4611	POGLUT1	0.2183	0.4073	protein O-glucosyltransferase 1
4612	P4HTM	0.2183	0.2384	prolyl 4-hydroxylase, transmembrane (endoplasmic reticulum)
4613	NR2C1	0.2183	0.3701	nuclear receptor subfamily 2, group C, member 1
4614	MOB3B	0.2183	0.1434	MOB kinase activator 3B
4615	DALRD3	0.2183	0.2495	DALR anticodon binding domain containing 3
4616	ZNHIT1	0.2175	0.2928	zinc finger, HIT-type containing 1
4617	XKRX	0.2175	0.0781	XK, Kell blood group complex subunit-related, X-linked
4618	TEAD4	0.2175	0.2331	TEA domain family member 4

4619	MTR	0.2175	0.4158	5-methyltetrahydrofolate-homocysteine methyltransferase
4620	MPV17	0.2175	0.3409	MpV17 mitochondrial inner membrane protein
4621	IL1RAP	0.2175	0.2353	interleukin 1 receptor accessory protein
4622	CRB1	0.2175	0.2171	crumbs homolog 1 (Drosophila)
4623	STX19	0.2171	0.0946	syntaxin 19
4624	PSG6	0.2171	0.0893	pregnancy specific beta-1-glycoprotein 6
4625	YTHDC2	0.2167	0.4277	YTH domain containing 2
4626	TYMP	0.2167	0.2735	thymidine phosphorylase
4627	RUSC1	0.2167	0.1997	RUN and SH3 domain containing 1
4628	PRKCH	0.2167	0.2172	protein kinase C, eta
4629	PLA2G16	0.2167	0.1809	phospholipase A2, group XVI
4630	PELO	0.2167	0.3769	pelota homolog (Drosophila)
4631	FSTL3	0.2167	0.2906	follistatin-like 3 (secreted glycoprotein)
4632	CDK2AP2	0.2167	0.2826	cyclin-dependent kinase 2 associated protein 2
4633	SNAPIN	0.2163	0.3494	SNAP-associated protein
4634	ZNF155	0.2158	0.132	zinc finger protein 155
4635	ZC3H7A	0.2158	0.4283	zinc finger CCCH-type containing 7A
4636	TPST2	0.2158	0.2356	tyrosylprotein sulfotransferase 2
4637	TCF4	0.2158	0.286	transcription factor 4
4638	SUV420H1	0.2158	0.4066	suppressor of variegation 4-20 homolog 1 (Drosophila)
4639	PITX1	0.2158	0.2199	paired-like homeodomain 1
4640	MRAS	0.2158	0.2247	muscle RAS oncogene homolog
4641	KLRF1	0.2158	0.1276	killer cell lectin-like receptor subfamily F, member 1
4642	EIF2AK3	0.2158	0.3991	eukaryotic translation initiation factor 2-alpha kinase 3
4643	CDC37L1	0.2158	0.395	cell division cycle 37 homolog (S. cerevisiae)-like 1
4644	C10orf68	0.2158	0.2551	chromosome 10 open reading frame 68
4645	NFIX	0.215	0.2765	nuclear factor I/X (CCAAT-binding transcription factor)
4646	MFSD3	0.215	0.2534	major facilitator superfamily domain containing 3
4647	LPCAT4	0.215	0.2035	lysophosphatidylcholine acyltransferase 4
4648	KRTAP1-3	0.215	0.2723	keratin associated protein 1-3
4649	INSIG2	0.215	0.4049	insulin induced gene 2
4650	IFNA10	0.215	0.1152	interferon, alpha 10
4651	CLMN	0.215	0.1474	calmin (calponin-like, transmembrane)
4652	CASP10	0.215	0.3403	caspase 10, apoptosis-related cysteine peptidase
4653	VANGL2	0.2143	0.1319	vang-like 2 (van gogh, Drosophila)
4654	ZFC3H1	0.2142	0.3662	zinc finger, C3H1-type containing
4655	TCF12	0.2142	0.4354	transcription factor 12
4656	STX12	0.2142	0.3846	syntaxin 12
4657	MUC16	0.2142	0.1974	mucin 16, cell surface associated
4658	MAFB	0.2142	0.2067	v-maf musculoaponeurotic fibrosarcoma oncogene homolog B (avian)
4659	BCAP29	0.2142	0.4056	B-cell receptor-associated protein 29
4660	UBE2E2	0.2138	0.3277	ubiquitin-conjugating enzyme E2E 2
4661	ZNF442	0.2133	0.1032	zinc finger protein 442
4662	TK2	0.2133	0.2561	thymidine kinase 2, mitochondrial
4663	PYROXD1	0.2133	0.4115	pyridine nucleotide-disulphide oxidoreductase domain 1
4664	PRPSAP1	0.2133	0.3898	phosphoribosyl pyrophosphate synthetase-associated protein 1
4665	MLL	0.2133	0.2655	myeloid/lymphoid or mixed-lineage leukemia (trithorax homolog, Drosophila)
4666	EIF2AK2	0.2133	0.3226	eukaryotic translation initiation factor 2-alpha kinase 2
4667	LPAR4	0.2125	0.1142	lysophosphatidic acid receptor 4
4668	LCAT	0.2125	0.2334	lecithin-cholesterol acyltransferase
4669	KRT1	0.2125	0.205	keratin 1
4670	HOXB9	0.2125	0.2228	homeobox B9
4671	AZI2	0.2125	0.4122	5-azacytidine induced 2
4672	TOR3A	0.2117	0.2927	torsin family 3, member A
4673	SYF2	0.2117	0.3475	SYF2 homolog, RNA splicing factor (S. cerevisiae)
4674	KCTD20	0.2117	0.3269	potassium channel tetramerisation domain containing 20
4675	CYTH3	0.2117	0.2106	cytohesin 3
4676	HCG11	0.2114	0	HLA complex group 11 (non-protein coding)
4677	TTTY10	0.2113	0.1194	testis-specific transcript, Y-linked 10 (non-protein coding)
4678	CHURC1	0.2113	0.3925	churchill domain containing 1
4679	C9orf41	0.2113	0.3434	chromosome 9 open reading frame 41
4680	TM2D1	0.2108	0.4015	TM2 domain containing 1
4681	EYA4	0.2108	0.15	eyes absent homolog 4 (Drosophila)
4682	CRYL1	0.2108	0.2443	crystallin, lambda 1
4683	AP2B1	0.2108	0.3575	adaptor-related protein complex 2, beta 1 subunit
4684	ALCAM	0.2108	0.323	activated leukocyte cell adhesion molecule
4685	RCAN1	0.21	0.2305	regulator of calcineurin 1
4686	RASSF7	0.21	0.3368	Ras association (RalGDS/AF-6) domain family (N-terminal) member 7
4687	PLCB1	0.21	0.2127	phospholipase C, beta 1 (phosphoinositide-specific)
4688	PIBF1	0.21	0.44	progesterone immunomodulatory binding factor 1
4689	LY6E	0.21	0.2574	lymphocyte antigen 6 complex, locus E
4690	LOC91948	0.21	0.1165	uncharacterized LOC91948
4691	KIAA1324	0.21	0.1209	KIAA1324
4692	IL36A	0.21	0.327	interleukin 36, alpha
4693	EML2	0.21	0.2183	echinoderm microtubule associated protein like 2
4694	CKM	0.21	0.2883	creatine kinase, muscle
4695	C7orf41	0.21	0.203	chromosome 7 open reading frame 41
4696	AVPR1A	0.21	0.3296	arginine vasopressin receptor 1A
4697	ALPL	0.21	0.2717	alkaline phosphatase, liver/bone/kidney
4698	UGT8	0.2092	0.1234	UDP glycosyltransferase 8
4699	RSAD1	0.2092	0.2481	radical S-adenosyl methionine domain containing 1
4700	RIC3	0.2092	0.1918	resistance to inhibitors of cholinesterase 3 homolog (C. elegans)
4701	PLEKHO2	0.2092	0.2665	pleckstrin homology domain containing, family O member 2
4702	OSR2	0.2092	0.088	odd-skipped related 2 (Drosophila)

4703	IFIH1	0.2092	0.3227	interferon induced with helicase C domain 1
4704	GTF3C2	0.2092	0.3609	general transcription factor IIIC, polypeptide 2, beta 110kDa
4705	CTDSP2	0.2092	0.2311	CTD (carboxy-terminal domain, RNA polymerase II, polypeptide A) small phosphatase 2
4706	ZNF251	0.2087	0.2633	zinc finger protein 251
4707	TMEM99	0.2087	0.2597	transmembrane protein 99
4708	VIL1	0.2083	0.2436	villin 1
4709	PRKY	0.2083	0.0714	protein kinase, Y-linked, pseudogene
4710	OSGEPL1	0.2083	0.4001	O-sialoglycoprotein endopeptidase-like 1
4711	NXPE4	0.2083	0.3132	neurexophilin and PC-esterase domain family, member 4
4712	NT5C	0.2083	0.2599	5', 3'-nucleotidase, cytosolic
4713	LARP4	0.2083	0.4053	La ribonucleoprotein domain family, member 4
4714	FGF20	0.2083	0.1805	fibroblast growth factor 20
4715	CLSTN1	0.2083	0.2955	calsyntenin 1
4716	CLPTM1	0.2083	0.301	cleft lip and palate associated transmembrane protein 1
4717	ZBTB47	0.2075	0.3165	zinc finger and BTB domain containing 47
4718	TXLNB	0.2075	0.0967	taxilin beta
4719	TBC1D2B	0.2075	0.3205	TBC1 domain family, member 2B
4720	PCDHGC3	0.2075	0.2906	protocadherin gamma subfamily C, 3
4721	KRT79	0.2075	0.2479	keratin 79
4722	CCBE1	0.2075	0.1589	collagen and calcium binding EGF domains 1
4723	ATRX	0.2075	0.3978	alpha thalassemia/mental retardation syndrome X-linked
4724	ZFP64	0.2067	0.185	zinc finger protein 64 homolog (mouse)
4725	STAG3L4	0.2067	0.1773	stromal antigen 3-like 4
4726	PMEL	0.2067	0.2968	premelanosome protein
4727	PHACTR4	0.2067	0.3291	phosphatase and actin regulator 4
4728	KRT37	0.2067	0.2968	keratin 37
4729	KIAA0664L3	0.2067	0.2228	KIAA0664-like 3
4730	GNGT1	0.2067	0.0608	guanine nucleotide binding protein (G protein), gamma transducing activity polypeptide 1
4731	ECEL1	0.2067	0.3243	endothelin converting enzyme-like 1
4732	SEC16B	0.2062	0.1854	SEC16 homolog B (S. cerevisiae)
4733	NRBP2	0.2062	0.2229	nuclear receptor binding protein 2
4734	JDP2	0.2062	0.1699	Jun dimerization protein 2
4735	SHMT1	0.2058	0.2702	serine hydroxymethyltransferase 1 (soluble)
4736	RNPEPL1	0.2058	0.3486	arginyl aminopeptidase (aminopeptidase B)-like 1
4737	PCK2	0.2058	0.2212	phosphoenolpyruvate carboxykinase 2 (mitochondrial)
4738	MYD88	0.2058	0.2688	myeloid differentiation primary response gene (88)
4739	MILR1	0.2058	0.1755	mast cell immunoglobulin-like receptor 1
4740	KLRAP1	0.2058	0.1918	killer cell lectin-like receptor subfamily A pseudogene 1
4741	CYP2C9	0.2058	0.2778	cytochrome P450, family 2, subfamily C, polypeptide 9
4742	CA4	0.2058	0.2889	carbonic anhydrase IV
4743	HS3ST6	0.2057	0.2811	heparan sulfate (glucosamine) 3-O-sulfotransferase 6
4744	ZCCHC6	0.205	0.3508	zinc finger, CCHC domain containing 6
4745	SMC5	0.205	0.4162	structural maintenance of chromosomes 5
4746	RGN	0.205	0.1884	regucalcin (senescence marker protein-30)
4747	PDE6B	0.205	0.1344	phosphodiesterase 6B, cGMP-specific, rod, beta
4748	LCE2B	0.205	0.2024	late cornified envelope 2B
4749	FUBP3	0.205	0.4258	far upstream element (FUSE) binding protein 3
4750	EMR3	0.205	0.232	egf-like module containing, mucin-like, hormone receptor-like 3
4751	DPYSL3	0.205	0.1778	dihydropyrimidinase-like 3
4752	VIP	0.2042	0.1756	vasoactive intestinal peptide
4753	LOXL2	0.2042	0.2369	lysyl oxidase-like 2
4754	HP1BP3	0.2042	0.3527	heterochromatin protein 1, binding protein 3
4755	HNF4A	0.2042	0.3622	hepatocyte nuclear factor 4, alpha
4756	FASLG	0.2042	0.2716	Fas ligand (TNF superfamily, member 6)
4757	CDC42EP4	0.2042	0.2271	CDC42 effector protein (Rho GTPase binding) 4
4758	ZNF160	0.2037	0.2978	zinc finger protein 160
4759	TMEM138	0.2037	0.368	transmembrane protein 138
4760	ZNF333	0.2025	0.179	zinc finger protein 333
4761	TRAPPC1	0.2025	0.3105	trafficking protein particle complex 1
4762	TNFSF18	0.2025	0.263	tumor necrosis factor (ligand) superfamily, member 18
4763	SLC47A2	0.2025	0.219	solute carrier family 47, member 2
4764	KRTAP4-3	0.2025	0.1376	keratin associated protein 4-3
4765	KCNJ2	0.2025	0.1779	potassium inwardly-rectifying channel, subfamily J, member 2
4766	FAM200A	0.2025	0.4253	family with sequence similarity 200, member A
4767	SLC35E1	0.2017	0.2479	solute carrier family 35, member E1
4768	RAB36	0.2017	0.2167	RAB36, member RAS oncogene family
4769	PDGFB	0.2017	0.3474	platelet-derived growth factor beta polypeptide
4770	PAK4	0.2017	0.3479	p21 protein (Cdc42/Rac)-activated kinase 4
4771	IFNW1	0.2017	0.2728	interferon, omega 1
4772	GUCY1A3	0.2017	0.1887	guanylate cyclase 1, soluble, alpha 3
4773	F5	0.2017	0.1847	coagulation factor V (proaccelerin, labile factor)
4774	ZNF311	0.2013	0.0792	zinc finger protein 311
4775	USP46-AS1	0.2013	0.1126	USP46 antisense RNA 1 (non-protein coding)
4776	SYTL3	0.2013	0.1592	synaptotagmin-like 3
4777	MOV10	0.2013	0.257	Mov10, Moloney leukemia virus 10, homolog (mouse)
4778	KIF26A	0.2013	0.1826	kinesin family member 26A
4779	DUSP27	0.2013	0.1181	dual specificity phosphatase 27 (putative)
4780	TAS2R4	0.2008	0.2521	taste receptor, type 2, member 4
4781	PHEX	0.2008	0.1571	phosphate regulating endopeptidase homolog, X-linked
4782	PDC	0.2008	0.1912	phosducin
4783	FAT2	0.2008	0.2713	FAT tumor suppressor homolog 2 (Drosophila)
4784	CHSY1	0.2008	0.3281	chondroitin sulfate synthase 1
4785	USP30	0.2	0.3456	ubiquitin specific peptidase 30
4786	SLTM	0.2	0.4368	SAFB-like, transcription modulator

4787	RPTN	0.2	0.1392	repetin
4788	PSENEN	0.2	0.2854	presenilin enhancer 2 homolog (C. elegans)
4789	PPP2R5A	0.2	0.2841	protein phosphatase 2, regulatory subunit B', alpha
4790	PIP5K1A	0.2	0.2077	phosphatidylinositol-4-phosphate 5-kinase, type I, alpha
4791	PGLYRP4	0.2	0.3047	peptidoglycan recognition protein 4
4792	PEX11B	0.2	0.4183	peroxisomal biogenesis factor 11 beta
4793	LETMD1	0.2	0.3258	LETM1 domain containing 1
4794	FLJ35024	0.2	0.0864	uncharacterized LOC401491
4795	CDHR3	0.2	0.1197	cadherin-related family member 3
4796	CAPN9	0.2	0.2894	calpain 9
4797	C6orf15	0.2	0.3489	chromosome 6 open reading frame 15
4798	C3orf58	0.2	0.2654	chromosome 3 open reading frame 58
4799	AKAP9	0.2	0.4037	A kinase (PRKA) anchor protein (yotiao) 9
4800	YIPF3	0.1992	0.3319	Yip1 domain family, member 3
4801	TRAPPC12	0.1992	0.3157	trafficking protein particle complex 12
4802	RIOK3	0.1992	0.3471	RIO kinase 3 (yeast)
4803	PVR	0.1992	0.3182	poliovirus receptor
4804	HAUS4	0.1992	0.3338	HAUS augmin-like complex, subunit 4
4805	CD70	0.1992	0.2498	CD70 molecule
4806	CAPN7	0.1992	0.4435	calpain 7
4807	ATG2B	0.1992	0.3893	autophagy related 2B
4808	NTPCR	0.1988	0.3604	nucleoside-triphosphatase, cancer-related
4809	DCLK2	0.1988	0.18	doublecortin-like kinase 2
4810	CTTNBP2	0.1988	0.134	cortactin binding protein 2
4811	CPEB2	0.1988	0.301	cytoplasmic polyadenylation element binding protein 2
4812	AHRR	0.1988	0.057	aryl-hydrocarbon receptor repressor
4813	TMEM223	0.1983	0.2671	transmembrane protein 223
4814	SPTBN5	0.1983	0.3353	spectrin, beta, non-erythrocytic 5
4815	SLC22A6	0.1983	0.3267	solute carrier family 22 (organic anion transporter), member 6
4816	MBD2	0.1983	0.1839	methyl-CpG binding domain protein 2
4817	HSD17B14	0.1983	0.3101	hydroxysteroid (17-beta) dehydrogenase 14
4818	ESRRG	0.1983	0.1805	estrogen-related receptor gamma
4819	ZNF268	0.1975	0.3676	zinc finger protein 268
4820	RASA3	0.1975	0.2302	RAS p21 protein activator 3
4821	KLHL5	0.1975	0.4089	kelch-like 5 (Drosophila)
4822	IFT46	0.1975	0.2822	intraflagellar transport 46 homolog (Chlamydomonas)
4823	GBP6	0.1975	0.1378	guanylate binding protein family, member 6
4824	ACAA2	0.1975	0.2099	acetyl-CoA acyltransferase 2
4825	ZCWPW2	0.1971	0.1652	zinc finger, CW type with PWWP domain 2
4826	ZNF117	0.1967	0.2687	zinc finger protein 117
4827	TPD52L2	0.1967	0.3356	tumor protein D52-like 2
4828	SYNPO2L	0.1967	0.253	synaptopodin 2-like
4829	RTP4	0.1967	0.1908	receptor (chemosensory) transporter protein 4
4830	DPYS	0.1967	0.2272	dihydropyrimidinase
4831	SLC39A10	0.1963	0.4316	solute carrier family 39 (zinc transporter), member 10
4832	KRTAP3-2	0.1963	0.1745	keratin associated protein 3-2
4833	ZMYM5	0.1958	0.3933	zinc finger, MYM-type 5
4834	ST3GAL4	0.1958	0.2493	ST3 beta-galactoside alpha-2,3-sialyltransferase 4
4835	RELN	0.1958	0.1697	reelin
4836	PGGT1B	0.1958	0.4072	protein geranylgeranyltransferase type I, beta subunit
4837	OR2W1	0.1958	0.2252	olfactory receptor, family 2, subfamily W, member 1
4838	IGSF3	0.1958	0.1721	immunoglobulin superfamily, member 3
4839	HOXD9	0.1958	0.3101	homeobox D9
4840	GK	0.1958	0.1759	glycerol kinase
4841	ENTPD3	0.1958	0.2116	ectonucleoside triphosphate diphosphohydrolase 3
4842	EHMT1	0.1958	0.2418	euchromatic histone-lysine N-methyltransferase 1
4843	ZC3HAV1L	0.195	0.098	zinc finger CCCH-type, antiviral 1-like
4844	ZBED3-AS1	0.195	0.149	ZBED3 antisense RNA 1 (non-protein coding)
4845	TCF21	0.195	0.2728	transcription factor 21
4846	PRSS27	0.195	0.2628	protease, serine 27
4847	PRKAB2	0.195	0.3162	protein kinase, AMP-activated, beta 2 non-catalytic subunit
4848	PPIP5K1	0.195	0.1978	diphosphoinositol pentakisphosphate kinase 1
4849	LFNG	0.195	0.2524	LFNG O-fucosylpeptide 3-beta-N-acetylglucosaminyltransferase
4850	FAM201A	0.195	0.0669	family with sequence similarity 201, member A
4851	UAP1L1	0.1942	0.2077	UDP-N-acteylglucosamine pyrophosphorylase 1-like 1
4852	RPL31	0.1942	0.3133	ribosomal protein L31
4853	LAMC3	0.1942	0.3231	laminin, gamma 3
4854	B3GALT4	0.1942	0.264	UDP-Gal:betaGlcNAc beta 1,3-galactosyltransferase, polypeptide 4
4855	ARHGAP28	0.1942	0.202	Rho GTPase activating protein 28
4856	IER5L	0.1937	0.2281	immediate early response 5-like
4857	ASPG	0.1937	0.3313	asparaginase homolog (S. cerevisiae)
4858	TOB2	0.1933	0.1994	transducer of ERBB2, 2
4859	TMSB10	0.1933	0.314	thymosin beta 10
4860	SAMD8	0.1933	0.3584	sterile alpha motif domain containing 8
4861	PRG4	0.1933	0.1411	proteoglycan 4
4862	LOC100240726	0.1933	0	makorin ring finger protein 1 pseudogene
4863	IFITM1	0.1933	0.227	interferon induced transmembrane protein 1
4864	HPSE2	0.1933	0.3385	heparanase 2
4865	GRAMD1C	0.1933	0.249	GRAM domain containing 1C
4866	GNAI1	0.1933	0.3563	guanine nucleotide binding protein (G protein), alpha inhibiting activity polypeptide 1
4867	ATP2A2	0.1933	0.3439	ATPase, Ca++ transporting, cardiac muscle, slow twitch 2
4868	S100A9	0.1925	0.1878	S100 calcium binding protein A9
4869	PLA2G2F	0.1925	0.297	phospholipase A2, group IIF
4870	PDK2	0.1925	0.3095	pyruvate dehydrogenase kinase, isozyme 2

4871	NIPAL3	0.1925	0.2521	NIPA-like domain containing 3
4872	KCNMB1	0.1925	0.2561	potassium large conductance calcium-activated channel, subfamily M, beta member 1
4873	GNA11	0.1925	0.2575	guanine nucleotide binding protein (G protein), alpha 11 (Gq class)
4874	CPXM1	0.1925	0.1544	carboxypeptidase X (M14 family), member 1
4875	CNIH4	0.1925	0.3827	cornichon homolog 4 (Drosophila)
4876	TIE1	0.1917	0.1716	tyrosine kinase with immunoglobulin-like and EGF-like domains 1
4877	SNX16	0.1917	0.4167	sorting nexin 16
4878	PICALM	0.1917	0.4069	phosphatidylinositol binding clathrin assembly protein
4879	NCOR1	0.1917	0.3836	nuclear receptor corepressor 1
4880	N4BP3	0.1917	0.2066	NEDD4 binding protein 3
4881	IL9	0.1917	0.2657	interleukin 9
4882	FAM83E	0.1917	0.2998	family with sequence similarity 83, member E
4883	ENAH	0.1917	0.2953	enabled homolog (Drosophila)
4884	COL9A3	0.1917	0.1194	collagen, type IX, alpha 3
4885	ALOX15	0.1917	0.2406	arachidonate 15-lipoxygenase
4886	ARSG	0.1912	0.1113	arylsulfatase G
4887	ZFAND1	0.1908	0.4211	zinc finger, AN1-type domain 1
4888	MBNL1	0.1908	0.4096	muscleblind-like splicing regulator 1
4889	KHDRBS2	0.1908	0.3242	KH domain containing, RNA binding, signal transduction associated 2
4890	ISG20L2	0.1908	0.3381	interferon stimulated exonuclease gene 20kDa-like 2
4891	CHD3	0.1908	0.2639	chromodomain helicase DNA binding protein 3
4892	CH25H	0.1908	0.1767	cholesterol 25-hydroxylase
4893	WWP2	0.19	0.2207	WW domain containing E3 ubiquitin protein ligase 2
4894	WHSC1L1	0.19	0.3169	Wolf-Hirschhorn syndrome candidate 1-like 1
4895	TNFRSF10C	0.19	0.2902	tumor necrosis factor receptor superfamily, member 10c, decoy without an intracellular domain
4896	RSPH1	0.19	0.1194	radial spoke head 1 homolog (Chlamydomonas)
4897	PI16	0.19	0.2946	peptidase inhibitor 16
4898	MYOZ1	0.19	0.3333	myozenin 1
4899	MAB21L2	0.19	0.2209	mab-21-like 2 (C. elegans)
4900	KDM2A	0.19	0.2806	lysine (K)-specific demethylase 2A
4901	GRB14	0.19	0.0994	growth factor receptor-bound protein 14
4902	C2orf68	0.19	0.2942	chromosome 2 open reading frame 68
4903	ATG7	0.19	0.2652	autophagy related 7
4904	ALX1	0.19	0.2137	ALX homeobox 1
4905	ALKBH8	0.19	0.409	alkB, alkylation repair homolog 8 (E. coli)
4906	SCGB1D1	0.1892	0.2229	secretoglobin, family 1D, member 1
4907	PRB1	0.1892	0.3131	proline-rich protein BstNI subfamily 1
4908	ERCC8	0.1892	0.3892	excision repair cross-complementing rodent repair deficiency, complementation group 8
4909	DCT	0.1892	0.316	dopachrome tautomerase (dopachrome delta-isomerase, tyrosine-related protein 2)
4910	ALAD	0.1892	0.2195	aminolevulinate dehydratase
4911	SYT8	0.1887	0.2595	synaptotagmin VIII
4912	PRAP1	0.1887	0.2838	proline-rich acidic protein 1
4913	BPIFB2	0.1887	0.2931	BPI fold containing family B, member 2
4914	HNRNPU-AS1	0.1886	0.3802	HNRNPU antisense RNA 1 (non-protein coding)
4915	ZNF589	0.1883	0.2257	zinc finger protein 589
4916	XYLT1	0.1883	0.1475	xylosyltransferase I
4917	SLC25A1	0.1883	0.3112	solute carrier family 25 (mitochondrial carrier; citrate transporter), member 1
4918	NFE2L3	0.1883	0.2758	nuclear factor (erythroid-derived 2)-like 3
4919	LRCH1	0.1883	0.23	leucine-rich repeats and calponin homology (CH) domain containing 1
4920	FHL5	0.1883	0.2364	four and a half LIM domains 5
4921	DNAH7	0.1883	0.2835	dynein, axonemal, heavy chain 7
4922	DARC	0.1883	0.2664	Duffy blood group, chemokine receptor
4923	CGA	0.1883	0.2473	glycoprotein hormones, alpha polypeptide
4924	CDC14A	0.1883	0.2387	CDC14 cell division cycle 14 homolog A (S. cerevisiae)
4925	ZFYVE26	0.1875	0.3251	zinc finger, FYVE domain containing 26
4926	PGPEP1	0.1875	0.2941	pyroglutamyl-peptidase I
4927	MAOB	0.1875	0.1881	monoamine oxidase B
4928	IRF9	0.1875	0.2897	interferon regulatory factor 9
4929	FBXW4	0.1875	0.2626	F-box and WD repeat domain containing 4
4930	C12orf69	0.1875	0.1275	chromosome 12 open reading frame 69
4931	ZEB1	0.1867	0.3384	zinc finger E-box binding homeobox 1
4932	TARBP1	0.1867	0.3461	TAR (HIV-1) RNA binding protein 1
4933	ORMDL2	0.1867	0.3332	ORM1-like 2 (S. cerevisiae)
4934	NHEJ1	0.1867	0.2762	nonhomologous end-joining factor 1
4935	KIAA0556	0.1867	0.2578	KIAA0556
4936	CREB1	0.1867	0.429	cAMP responsive element binding protein 1
4937	CALCOCO2	0.1867	0.3825	calcium binding and coiled-coil domain 2
4938	ZNF800	0.1863	0.2206	zinc finger protein 800
4939	TMEM237	0.1863	0.4152	transmembrane protein 237
4940	RC3H1	0.1863	0.3778	ring finger and CCCH-type domains 1
4941	PPM1M	0.1863	0.2779	protein phosphatase, Mg2+/Mn2+ dependent, 1M
4942	TNK1	0.1858	0.3432	tyrosine kinase, non-receptor, 1
4943	PDSS2	0.1858	0.4179	prenyl (decaprenyl) diphosphate synthase, subunit 2
4944	MYL1	0.1858	0.2537	myosin, light chain 1, alkali; skeletal, fast
4945	FGFR1	0.1858	0.2117	fibroblast growth factor receptor 1
4946	FAH	0.1858	0.2743	fumarylacetoacetate hydrolase (fumarylacetoacetase)
4947	ATXN8OS	0.1855	0.3572	ATXN8 opposite strand (non-protein coding)
4948	ZFP28	0.185	0.2124	zinc finger protein 28 homolog (mouse)
4949	TNIP2	0.185	0.2675	TNFAIP3 interacting protein 2
4950	TMEM176A	0.185	0.2171	transmembrane protein 176A
4951	RNF212	0.185	0.1758	ring finger protein 212
4952	RAB3GAP1	0.185	0.4092	RAB3 GTPase activating protein subunit 1 (catalytic)
4953	NBN	0.185	0.4335	nibrin
4954	HAPLN1	0.185	0.2143	hyaluronan and proteoglycan link protein 1

4955	ESRRA	0.185	0.2957	estrogen-related receptor alpha
4956	DCSTAMP	0.185	0.3083	dendrocyte expressed seven transmembrane protein
4957	BOD1L1	0.185	0.3862	biorientation of chromosomes in cell division 1-like 1
4958	ATL3	0.185	0.3687	atlastin GTPase 3
4959	ALG10B	0.185	0.4047	asparagine-linked glycosylation 10, alpha-1,2-glucosyltransferase homolog B (yeast)
4960	ADRA1A	0.185	0.3627	adrenoceptor alpha 1A
4961	SLC30A9	0.1842	0.4617	solute carrier family 30 (zinc transporter), member 9
4962	SLC26A3	0.1842	0.1164	solute carrier family 26, member 3
4963	SLC15A1	0.1842	0.1454	solute carrier family 15 (oligopeptide transporter), member 1
4964	S100G	0.1842	0.2321	S100 calcium binding protein G
4965	PRDX3	0.1842	0.4239	peroxiredoxin 3
4966	NDUFS4	0.1842	0.3921	NADH dehydrogenase (ubiquinone) Fe-S protein 4, 18kDa (NADH-coenzyme Q reductase)
4967	MFN1	0.1842	0.4386	mitofusin 1
4968	KANK3	0.1842	0.3342	KN motif and ankyrin repeat domains 3
4969	COMMD7	0.1838	0.2674	COMM domain containing 7
4970	SLC31A2	0.1833	0.2847	solute carrier family 31 (copper transporters), member 2
4971	IL17A	0.1833	0.3126	interleukin 17A
4972	HERC6	0.1833	0.2634	HECT and RLD domain containing E3 ubiquitin protein ligase family member 6
4973	CAP2	0.1833	0.2319	CAP, adenylate cyclase-associated protein, 2 (yeast)
4974	TAS2R16	0.1825	0.2437	taste receptor, type 2, member 16
4975	PTGER2	0.1825	0.177	prostaglandin E receptor 2 (subtype EP2), 53kDa
4976	PLEKHG4B	0.1825	0.2376	pleckstrin homology domain containing, family G (with RhoGef domain) member 4B
4977	LURAP1	0.1825	0.2999	leucine rich adaptor protein 1
4978	LTA4H	0.1825	0.3973	leukotriene A4 hydrolase
4979	IL5RA	0.1825	0.3336	interleukin 5 receptor, alpha
4980	ESPNL	0.1825	0.3285	espin-like
4981	ECHDC1	0.1825	0.4295	enoyl CoA hydratase domain containing 1
4982	CYP4A11	0.1825	0.3101	cytochrome P450, family 4, subfamily A, polypeptide 11
4983	CSRP3	0.1825	0.2503	cysteine and glycine-rich protein 3 (cardiac LIM protein)
4984	C17orf79	0.1825	0.2746	chromosome 17 open reading frame 79
4985	C14orf126	0.1825	0.4284	chromosome 14 open reading frame 126
4986	LY6G6D	0.1818	0.3127	lymphocyte antigen 6 complex, locus G6D
4987	TMEM187	0.1817	0.2059	transmembrane protein 187
4988	RELB	0.1817	0.2794	v-rel reticuloendotheliosis viral oncogene homolog B
4989	RBMY2FP	0.1817	0.258	RNA binding motif protein, Y-linked, family 2, member F pseudogene
4990	RAB21	0.1817	0.429	RAB21, member RAS oncogene family
4991	IL1R2	0.1817	0.1827	interleukin 1 receptor, type II
4992	GRHPR	0.1817	0.3383	glyoxylate reductase/hydroxypyruvate reductase
4993	TAP1	0.1808	0.2856	transporter 1, ATP-binding cassette, sub-family B (MDR/TAP)
4994	SLC12A3	0.1808	0.3453	solute carrier family 12 (sodium/chloride transporters), member 3
4995	PYGM	0.1808	0.318	phosphorylase, glycogen, muscle
4996	PPP1R13B	0.1808	0.2281	protein phosphatase 1, regulatory subunit 13B
4997	NPY2R	0.1808	0.253	neuropeptide Y receptor Y2
4998	HIBCH	0.1808	0.4304	3-hydroxyisobutyryl-CoA hydrolase
4999	WDR66	0.18	0.1107	WD repeat domain 66
5000	TNIP3	0.18	0.1249	TNFAIP3 interacting protein 3
5001	TMSB4Y	0.18	0.1898	thymosin beta 4, Y-linked
5002	PRKCA	0.18	0.1792	protein kinase C, alpha
5003	NMBR	0.18	0.2264	neuromedin B receptor
5004	MED29	0.18	0.3669	mediator complex subunit 29
5005	KRIT1	0.18	0.4476	KRIT1, ankyrin repeat containing
5006	DPY30	0.18	0.4187	dpy-30 homolog (C. elegans)
5007	SMUG1	0.1792	0.2829	single-strand-selective monofunctional uracil-DNA glycosylase 1
5008	OTOR	0.1792	0.2821	otoraplin
5009	ADM2	0.1792	0.3648	adrenomedullin 2
5010	ZNF354C	0.1787	0.1151	zinc finger protein 354C
5011	RDH12	0.1787	0.209	retinol dehydrogenase 12 (all-trans/9-cis/11-cis)
5012	MRPL32	0.1787	0.3885	mitochondrial ribosomal protein L32
5013	MIB1	0.1787	0.4535	mindbomb E3 ubiquitin protein ligase 1
5014	LONRF1	0.1787	0.3768	LON peptidase N-terminal domain and ring finger 1
5015	FAM196A	0.1787	0.0451	family with sequence similarity 196, member A
5016	AKT1S1	0.1787	0.2633	AKT1 substrate 1 (proline-rich)
5017	SLN	0.1783	0.2751	sarcolipin
5018	PLSCR1	0.1783	0.3818	phospholipid scramblase 1
5019	CYTL1	0.1783	0.1397	cytokine-like 1
5020	ALDOB	0.1783	0.3319	aldolase B, fructose-bisphosphate
5021	SYNRG	0.1778	0.3926	synergin, gamma
5022	NUDT4	0.1775	0.2764	nudix (nucleoside diphosphate linked moiety X)-type motif 4
5023	GBE1	0.1775	0.4017	glucan (1,4-alpha-), branching enzyme 1
5024	GAA	0.1775	0.2913	glucosidase, alpha; acid
5025	DERL2	0.1775	0.3987	derlin 2
5026	C15orf44	0.1775	0.4038	chromosome 15 open reading frame 44
5027	RGS3	0.1767	0.1851	regulator of G-protein signaling 3
5028	MS4A2	0.1767	0.244	membrane-spanning 4-domains, subfamily A, member 2
5029	MED23	0.1767	0.4403	mediator complex subunit 23
5030	KRT10	0.1767	0.3014	keratin 10
5031	GPATCH2	0.1767	0.3682	G patch domain containing 2
5032	DDO	0.1767	0.2433	D-aspartate oxidase
5033	C1QTNF3	0.1767	0.1975	C1q and tumor necrosis factor related protein 3
5034	CCDC120	0.1762	0.2709	coiled-coil domain containing 120
5035	SEPW1	0.1758	0.2935	selenoprotein W, 1
5036	FAM204A	0.1758	0.414	family with sequence similarity 204, member A
5037	CYSLTR2	0.1758	0.272	cysteinyl leukotriene receptor 2
5038	CCL7	0.1758	0.2449	chemokine (C-C motif) ligand 7

5039	RGS13	0.175	0.1145	regulator of G-protein signaling 13
5040	LOC100130633	0.175	0	hypothetical protein LOC100130633
5041	GLYR1	0.175	0.2583	glyoxylate reductase 1 homolog (Arabidopsis)
5042	GDPD2	0.175	0.3262	glycerophosphodiester phosphodiesterase domain containing 2
5043	DNER	0.175	0.094	delta/notch-like EGF repeat containing
5044	CTSE	0.175	0.272	cathepsin E
5045	CLCA4	0.175	0.1606	chloride channel accessory 4
5046	AFAP1L1	0.175	0.144	actin filament associated protein 1-like 1
5047	ZNF385D	0.1742	0.1948	zinc finger protein 385D
5048	SLMO2	0.1742	0.4315	slowmo homolog 2 (Drosophila)
5049	RHOH	0.1742	0.1783	ras homolog family member H
5050	P2RY14	0.1742	0.1823	purinergic receptor P2Y, G-protein coupled, 14
5051	LRRC49	0.1742	0.2673	leucine rich repeat containing 49
5052	EPHA7	0.1742	0.1626	EPH receptor A7
5053	APPL1	0.1742	0.4497	adaptor protein, phosphotyrosine interaction, PH domain and leucine zipper containing 1
5054	DCDC2	0.1737	0.0836	doublecortin domain containing 2
5055	C22orf25	0.1737	0.2578	chromosome 22 open reading frame 25
5056	UBR5	0.1733	0.4333	ubiquitin protein ligase E3 component n-recognin 5
5057	TTC12	0.1733	0.2395	tetratricopeptide repeat domain 12
5058	TP73-AS1	0.1733	0.1903	TP73 antisense RNA 1 (non-protein coding)
5059	RAC1	0.1733	0.4322	ras-related C3 botulinum toxin substrate 1 (rho family, small GTP binding protein Rac1)
5060	HPD	0.1733	0.2696	4-hydroxyphenylpyruvate dioxygenase
5061	ADH1A	0.1733	0.2539	alcohol dehydrogenase 1A (class I), alpha polypeptide
5062	PIAS1	0.1725	0.3788	protein inhibitor of activated STAT, 1
5063	NT5C3L	0.1725	0.2916	5'-nucleotidase, cytosolic III-like
5064	NCOR2	0.1725	0.3496	nuclear receptor corepressor 2
5065	MTMR9LP	0.1725	0.2644	myotubularin related protein 9-like, pseudogene
5066	MIPOL1	0.1725	0.2441	mirror-image polydactyly 1
5067	IQSEC2	0.1725	0.379	IQ motif and Sec7 domain 2
5068	HTR1F	0.1725	0.2353	5-hydroxytryptamine (serotonin) receptor 1F, G protein-coupled
5069	GLYATL2	0.1725	0.0962	glycine-N-acyltransferase-like 2
5070	FKBP15	0.1725	0.3007	FK506 binding protein 15, 133kDa
5071	COQ2	0.1725	0.3887	coenzyme Q2 homolog, prenyltransferase (yeast)
5072	CCDC146	0.1725	0.2794	coiled-coil domain containing 146
5073	CC2D1B	0.1725	0.2295	coiled-coil and C2 domain containing 1B
5074	C1orf63	0.1725	0.3506	chromosome 1 open reading frame 63
5075	AFM	0.1725	0.1851	afamin
5076	ADPRH	0.1725	0.2659	ADP-ribosylarginine hydrolase
5077	XAF1	0.1717	0.2311	XIAP associated factor 1
5078	TSC22D1	0.1717	0.3095	TSC22 domain family, member 1
5079	RAP1GAP	0.1717	0.2637	RAP1 GTPase activating protein
5080	NDRG2	0.1717	0.2295	NDRG family member 2
5081	MBIP	0.1717	0.4302	MAP3K12 binding inhibitory protein 1
5082	FOXL1	0.1717	0.3434	forkhead box L1
5083	FGGY	0.1717	0.1921	FGGY carbohydrate kinase domain containing
5084	CREB3	0.1717	0.3133	cAMP responsive element binding protein 3
5085	TRIM52	0.1713	0.2938	tripartite motif containing 52
5086	STARD10	0.1713	0.2842	StAR-related lipid transfer (START) domain containing 10
5087	PLEKHA7	0.1713	0.1364	pleckstrin homology domain containing, family A member 7
5088	LHX9	0.1713	0.241	LIM homeobox 9
5089	HKR1	0.1713	0.3109	HKR1, GLI-Kruppel zinc finger family member
5090	C1orf88	0.1713	0.1853	chromosome 1 open reading frame 88
5091	ZNF221	0.1708	0.2723	zinc finger protein 221
5092	TREH	0.1708	0.2664	trehalase (brush-border membrane glycoprotein)
5093	SIKE1	0.1708	0.4214	suppressor of IKBKE 1
5094	PAX2	0.1708	0.3323	paired box 2
5095	MMP11	0.1708	0.3281	matrix metallopeptidase 11 (stromelysin 3)
5096	C11orf67	0.1708	0.2485	chromosome 11 open reading frame 67
5097	BCL2L2	0.1708	0.2527	BCL2-like 2
5098	ZNF561	0.17	0.4379	zinc finger protein 561
5099	TTC39B	0.17	0.2557	tetratricopeptide repeat domain 39B
5100	TLR2	0.17	0.2303	toll-like receptor 2
5101	TINF2	0.17	0.3256	TERF1 (TRF1)-interacting nuclear factor 2
5102	TIFA	0.17	0.3979	TRAF-interacting protein with forkhead-associated domain
5103	SYNE4	0.17	0.2252	spectrin repeat containing, nuclear envelope family member 4
5104	SLC30A6	0.17	0.4504	solute carrier family 30 (zinc transporter), member 6
5105	SLC22A11	0.17	0.336	solute carrier family 22 (organic anion/urate transporter), member 11
5106	SLC13A3	0.17	0.3516	solute carrier family 13 (sodium-dependent dicarboxylate transporter), member 3
5107	OR1G1	0.17	0.31	olfactory receptor, family 1, subfamily G, member 1
5108	MFAP2	0.17	0.1745	microfibrillar-associated protein 2
5109	LMF2	0.17	0.376	lipase maturation factor 2
5110	EEF2K	0.17	0.3005	eukaryotic elongation factor-2 kinase
5111	DDIT3	0.17	0.1934	DNA-damage-inducible transcript 3
5112	CTSH	0.17	0.2656	cathepsin H
5113	CCDC50	0.17	0.3993	coiled-coil domain containing 50
5114	APOBEC3C	0.17	0.2462	apolipoprotein B mRNA editing enzyme, catalytic polypeptide-like 3C
5115	ACAA1	0.17	0.2879	acetyl-CoA acyltransferase 1
5116	TCAP	0.1692	0.3238	titin-cap (telethonin)
5117	SMG1	0.1692	0.4006	smg-1 homolog, phosphatidylinositol 3-kinase-related kinase (C. elegans)
5118	PLAA	0.1692	0.4379	phospholipase A2-activating protein
5119	PDPN	0.1692	0.2116	podoplanin
5120	HOXD11	0.1692	0.2474	homeobox D11
5121	DIO3	0.1692	0.3357	deiodinase, iodothyronine, type III
5122	CYP7A1	0.1692	0.0677	cytochrome P450, family 7, subfamily A, polypeptide 1

5123	TRIM65	0.1688	0.1738	tripartite motif containing 65
5124	TET2	0.1688	0.3333	tet methylcytosine dioxygenase 2
5125	SRFBP1	0.1688	0.4474	serum response factor binding protein 1
5126	DZIP1L	0.1688	0.1919	DAZ interacting protein 1-like
5127	CHD2	0.1688	0.3445	chromodomain helicase DNA binding protein 2
5128	CCDC148	0.1688	0.1648	coiled-coil domain containing 148
5129	C1orf204	0.1688	0.1578	chromosome 1 open reading frame 204
5130	BRK1	0.1688	0.3923	BRICK1, SCAR/WAVE actin-nucleating complex subunit
5131	TM4SF5	0.1683	0.3442	transmembrane 4 L six family member 5
5132	RAB4A	0.1683	0.4349	RAB4A, member RAS oncogene family
5133	OAS3	0.1683	0.2192	2'-5'-oligoadenylate synthetase 3, 100kDa
5134	MGAM	0.1683	0.1143	maltase-glucoamylase (alpha-glucosidase)
5135	HOMER2	0.1683	0.1222	homer homolog 2 (Drosophila)
5136	FAM134C	0.1683	0.3327	family with sequence similarity 134, member C
5137	ZC3H10	0.1675	0.1668	zinc finger CCCH-type containing 10
5138	TMEM60	0.1675	0.3959	transmembrane protein 60
5139	SLC9A3R2	0.1675	0.3108	solute carrier family 9, subfamily A (NHE3, cation proton antiporter 3), member 3 regulator 2
5140	SAP30L	0.1675	0.4128	SAP30-like
5141	PFDN5	0.1675	0.3234	prefoldin subunit 5
5142	PDE4B	0.1675	0.2357	phosphodiesterase 4B, cAMP-specific
5143	NEIL1	0.1675	0.3121	nei endonuclease VIII-like 1 (E. coli)
5144	MAF1	0.1675	0.3104	MAF1 homolog (S. cerevisiae)
5145	HLA-DRB1	0.1675	0.2202	major histocompatibility complex, class II, DR beta 1
5146	FGFBP2	0.1675	0.0724	fibroblast growth factor binding protein 2
5147	FAM111A	0.1675	0.3712	family with sequence similarity 111, member A
5148	DHRS7B	0.1675	0.337	dehydrogenase/reductase (SDR family) member 7B
5149	CSF1	0.1675	0.376	colony stimulating factor 1 (macrophage)
5150	CLSTN2	0.1675	0.2255	calsyntenin 2
5151	RCN1	0.1671	0.3774	reticulocalbin 1, EF-hand calcium binding domain
5152	TRHDE	0.1667	0.1277	thyrotropin-releasing hormone degrading enzyme
5153	TGFB3	0.1667	0.2809	transforming growth factor, beta 3
5154	STRN	0.1667	0.3096	striatin, calmodulin binding protein
5155	OR5I1	0.1667	0.3107	olfactory receptor, family 5, subfamily I, member 1
5156	ECH1	0.1667	0.3529	enoyl CoA hydratase 1, peroxisomal
5157	COL2A1	0.1667	0.2976	collagen, type II, alpha 1
5158	VMA21	0.1663	0.4244	VMA21 vacuolar H+-ATPase homolog (S. cerevisiae)
5159	GLIPR2	0.1663	0.2887	GLI pathogenesis-related 2
5160	ZNF468	0.1658	0.4307	zinc finger protein 468
5161	ZNF33B	0.1658	0.3771	zinc finger protein 33B
5162	UGT2B17	0.1658	0.137	UDP glucuronosyltransferase 2 family, polypeptide B17
5163	UBR4	0.1658	0.3237	ubiquitin protein ligase E3 component n-recognin 4
5164	TLE2	0.1658	0.2941	transducin-like enhancer of split 2 (E(sp1) homolog, Drosophila)
5165	RYR2	0.1658	0.1997	ryanodine receptor 2 (cardiac)
5166	MOGS	0.1658	0.3184	mannosyl-oligosaccharide glucosidase
5167	KAT6B	0.1658	0.3237	K(lysine) acetyltransferase 6B
5168	GUCY2C	0.1658	0.2218	guanylate cyclase 2C (heat stable enterotoxin receptor)
5169	FBXO3	0.1658	0.4538	F-box protein 3
5170	FBN2	0.1658	0.1394	fibrillin 2
5171	COBL	0.1658	0.1648	cordon-bleu homolog (mouse)
5172	CEP112	0.1658	0.2883	centrosomal protein 112kDa
5173	BFSP1	0.1658	0.1762	beaded filament structural protein 1, filensin
5174	ZNF177	0.1657	0.2731	zinc finger protein 177
5175	ZNF549	0.165	0.1626	zinc finger protein 549
5176	TFF1	0.165	0.2485	trefoil factor 1
5177	RCAN3	0.165	0.1671	RCAN family member 3
5178	PRKG2	0.165	0.2382	protein kinase, cGMP-dependent, type II
5179	MYOM1	0.165	0.2587	myomesin 1, 185kDa
5180	HLA-DRB6	0.165	0.1956	major histocompatibility complex, class II, DR beta 6 (pseudogene)
5181	GLRA3	0.165	0.2479	glycine receptor, alpha 3
5182	GALNTL2	0.165	0.2525	UDP-N-acetyl-alpha-D-galactosamine:polypeptide N-acetylgalactosaminyltransferase-like 2
5183	GALNT6	0.165	0.1682	UDP-N-acetyl-alpha-D-galactosamine:polypeptide N-acetylgalactosaminyltransferase 6 (GalNAc-T6)
5184	FAM173B	0.165	0.4363	family with sequence similarity 173, member B
5185	FAM150B	0.165	0.0914	family with sequence similarity 150, member B
5186	FAM124B	0.165	0.2237	family with sequence similarity 124B
5187	EXD3	0.165	0.2669	exonuclease 3'-5' domain containing 3
5188	C12orf57	0.165	0.3143	chromosome 12 open reading frame 57
5189	MAEA	0.1642	0.4325	macrophage erythroblast attacher
5190	KDM5A	0.1642	0.3864	lysine (K)-specific demethylase 5A
5191	IFT52	0.1642	0.4223	intraflagellar transport 52 homolog (Chlamydomonas)
5192	HYAL4	0.1642	0.3088	hyaluronoglucosaminidase 4
5193	GDE1	0.1642	0.3838	glycerophosphodiester phosphodiesterase 1
5194	EGLN3	0.1642	0.1361	egl nine homolog 3 (C. elegans)
5195	DVL3	0.1642	0.2851	dishevelled, dsh homolog 3 (Drosophila)
5196	CPVL	0.1642	0.2233	carboxypeptidase, vitellogenic-like
5197	LINC00340	0.1637	0.1925	long intergenic non-protein coding RNA 340
5198	ZYX	0.1633	0.3217	zyxin
5199	TMX4	0.1633	0.3551	thioredoxin-related transmembrane protein 4
5200	RPS23	0.1633	0.3599	ribosomal protein S23
5201	MOSPD2	0.1633	0.3962	motile sperm domain containing 2
5202	MED8	0.1633	0.39	mediator complex subunit 8
5203	MARK2	0.1633	0.2991	MAP/microtubule affinity-regulating kinase 2
5204	FBXL4	0.1633	0.4409	F-box and leucine-rich repeat protein 4
5205	CAPN5	0.1633	0.3519	calpain 5
5206	TBX10	0.1625	0.3584	T-box 10

5207	PSMG3	0.1625	0.3535	proteasome (prosome, macropain) assembly chaperone 3
5208	PKD2L1	0.1625	0.3202	polycystic kidney disease 2-like 1
5209	PAPD4	0.1625	0.454	PAP associated domain containing 4
5210	GUSB	0.1625	0.3574	glucuronidase, beta
5211	CLN8	0.1625	0.1611	ceroid-lipofuscinosis, neuronal 8 (epilepsy, progressive with mental retardation)
5212	SMPX	0.1617	0.1859	small muscle protein, X-linked
5213	NNT	0.1617	0.4368	nicotinamide nucleotide transhydrogenase
5214	XGPY2	0.1614	0	Xg pseudogene, Y-linked 2
5215	LACRT	0.1612	0.2725	lacritin
5216	KREMEN1	0.1612	0.2981	kringle containing transmembrane protein 1
5217	IGSF22	0.1612	0.1907	immunoglobulin superfamily, member 22
5218	WBSCR22	0.1608	0.349	Williams Beuren syndrome chromosome region 22
5219	NCOA2	0.1608	0.3325	nuclear receptor coactivator 2
5220	MTHFR	0.1608	0.364	methylenetetrahydrofolate reductase (NAD(P)H)
5221	LRRC16A	0.1608	0.2505	leucine rich repeat containing 16A
5222	HOXC5	0.1608	0.2822	homeobox C5
5223	FTSJ1	0.1608	0.3747	FtsJ RNA methyltransferase homolog 1 (E. coli)
5224	FAM110B	0.1608	0.1989	family with sequence similarity 110, member B
5225	DNAJC13	0.1608	0.4429	DnaJ (Hsp40) homolog, subfamily C, member 13
5226	CCNI	0.1608	0.348	cyclin I
5227	ACSM3	0.1608	0.1809	acyl-CoA synthetase medium-chain family member 3
5228	WBP2NL	0.16	0.0624	WBP2 N-terminal like
5229	TPSAB1	0.16	0.3225	tryptase alpha/beta 1
5230	SPTSSA	0.16	0.4223	serine palmitoyltransferase, small subunit A
5231	SLC27A1	0.16	0.2953	solute carrier family 27 (fatty acid transporter), member 1
5232	PHF23	0.16	0.3533	PHD finger protein 23
5233	MLIP	0.16	0.1785	muscular LMNA-interacting protein
5234	FAM57A	0.16	0.2616	family with sequence similarity 57, member A
5235	DMPK	0.16	0.3256	dystrophia myotonica-protein kinase
5236	CATSPERB	0.16	0.1411	catsper channel auxiliary subunit beta
5237	ANKRD5	0.16	0.1936	ankyrin repeat domain 5
5238	ADAMTS15	0.16	0.2844	ADAM metallopeptidase with thrombospondin type 1 motif, 15
5239	SMPDL3B	0.1592	0.3008	sphingomyelin phosphodiesterase, acid-like 3B
5240	PODNL1	0.1592	0.2934	podocan-like 1
5241	LAMA3	0.1592	0.2927	laminin, alpha 3
5242	KLHDC10	0.1592	0.3711	kelch domain containing 10
5243	HECW1	0.1592	0.3379	HECT, C2 and WW domain containing E3 ubiquitin protein ligase 1
5244	ERO1L	0.1592	0.3994	ERO1-like (S. cerevisiae)
5245	CIDEA	0.1592	0.3051	cell death-inducing DFFA-like effector a
5246	ABCD3	0.1592	0.4545	ATP-binding cassette, sub-family D (ALD), member 3
5247	PXK	0.1587	0.3702	PX domain containing serine/threonine kinase
5248	PRPF40B	0.1587	0.2247	PRP40 pre-mRNA processing factor 40 homolog B (S. cerevisiae)
5249	C10orf47	0.1587	0.1025	chromosome 10 open reading frame 47
5250	KRTAP4-9	0.1586	0.2069	keratin associated protein 4-9
5251	ZMAT4	0.1583	0.2751	zinc finger, matrin-type 4
5252	SEMA4A	0.1583	0.2957	sema domain, immunoglobulin domain (Ig), transmembrane domain (TM) and short cytoplasmic domain, (semaphorin) 4A
5253	PITX2	0.1583	0.0553	paired-like homeodomain 2
5254	PBX3	0.1583	0.4033	pre-B-cell leukemia homeobox 3
5255	MKNK2	0.1583	0.2625	MAP kinase interacting serine/threonine kinase 2
5256	ZFP3	0.1575	0.2766	zinc finger protein 3 homolog (mouse)
5257	SH3GLB1	0.1575	0.4119	SH3-domain GRB2-like endophilin B1
5258	RRAD	0.1575	0.264	Ras-related associated with diabetes
5259	MAPK1IP1L	0.1575	0.4429	mitogen-activated protein kinase 1 interacting protein 1-like
5260	LRRC37A3	0.1575	0.2256	leucine rich repeat containing 37, member A3
5261	LNX2	0.1575	0.4252	ligand of numb-protein X 2
5262	KY	0.1575	0.303	kyphoscoliosis peptidase
5263	KDELC1	0.1575	0.3639	KDEL (Lys-Asp-Glu-Leu) containing 1
5264	GM2A	0.1575	0.2687	GM2 ganglioside activator
5265	FMO3	0.1575	0.1622	flavin containing monooxygenase 3
5266	ARFGAP3	0.1575	0.4243	ADP-ribosylation factor GTPase activating protein 3
5267	AOC3	0.1575	0.1973	amine oxidase, copper containing 3 (vascular adhesion protein 1)
5268	ZNF805	0.1571	0.4329	zinc finger protein 805
5269	TBX2	0.1567	0.364	T-box 2
5270	SERPINA6	0.1567	0.2994	serpin peptidase inhibitor, clade A (alpha-1 antiproteinase, antitrypsin), member 6
5271	PRSS22	0.1567	0.3434	protease, serine, 22
5272	GSTA4	0.1567	0.3034	glutathione S-transferase alpha 4
5273	CHRM5	0.1567	0.3438	cholinergic receptor, muscarinic 5
5274	VMO1	0.1563	0.2176	vitelline membrane outer layer 1 homolog (chicken)
5275	FBXL17	0.1563	0.1243	F-box and leucine-rich repeat protein 17
5276	DNAL1	0.1563	0.2842	dynein, axonemal, light chain 1
5277	C9orf66	0.1563	0.1346	chromosome 9 open reading frame 66
5278	ZCCHC2	0.1558	0.3199	zinc finger, CCHC domain containing 2
5279	UNC5C	0.1558	0.3058	unc-5 homolog C (C. elegans)
5280	PCDHB17	0.1558	0.3304	protocadherin beta 17 pseudogene
5281	DOK5	0.1558	0.2066	docking protein 5
5282	CXCL9	0.1558	0.2272	chemokine (C-X-C motif) ligand 9
5283	SNORD8	0.1557	0.1208	small nucleolar RNA, C/D box 8
5284	TCEAL3	0.155	0.1776	transcription elongation factor A (SII)-like 3
5285	NMRAL1	0.155	0.327	NmrA-like family domain containing 1
5286	MYH14	0.155	0.3865	myosin, heavy chain 14, non-muscle
5287	MITD1	0.155	0.4493	MIT, microtubule interacting and transport, domain containing 1
5288	IL1RL1	0.155	0.2933	interleukin 1 receptor-like 1
5289	EBF3	0.155	0.1647	early B-cell factor 3
5290	ZNF606	0.1542	0.2883	zinc finger protein 606

5291	SLC2A1	0.1542	0.1806	solute carrier family 2 (facilitated glucose transporter), member 1
5292	RRNAD1	0.1542	0.2754	ribosomal RNA adenine dimethylase domain containing 1
5293	PSG1	0.1542	0.1651	pregnancy specific beta-1-glycoprotein 1
5294	NOTCH4	0.1542	0.3183	notch 4
5295	CRNN	0.1542	0.3379	cornulin
5296	ATP8B2	0.1542	0.2405	ATPase, aminophospholipid transporter, class I, type 8B, member 2
5297	ARPC1B	0.1542	0.3469	actin related protein 2/3 complex, subunit 1B, 41kDa
5298	ABCA5	0.1542	0.3603	ATP-binding cassette, sub-family A (ABC1), member 5
5299	C12orf75	0.1538	0.3703	chromosome 12 open reading frame 75
5300	PPEF2	0.1533	0.1573	protein phosphatase, EF-hand calcium binding domain 2
5301	PEX6	0.1533	0.2122	peroxisomal biogenesis factor 6
5302	FAM206A	0.1533	0.4135	family with sequence similarity 206, member A
5303	DACT1	0.1533	0.1907	dapper, antagonist of beta-catenin, homolog 1 (Xenopus laevis)
5304	ANGPTL3	0.1533	0.239	angiopoietin-like 3
5305	TMEM63A	0.1525	0.3294	transmembrane protein 63A
5306	TBC1D9	0.1525	0.3619	TBC1 domain family, member 9 (with GRAM domain)
5307	PRR15	0.1525	0.1521	proline rich 15
5308	CREB5	0.1525	0.2241	cAMP responsive element binding protein 5
5309	CANT1	0.1525	0.3336	calcium activated nucleotidase 1
5310	C6orf57	0.1525	0.3468	chromosome 6 open reading frame 57
5311	AIF1L	0.1525	0.1741	allograft inflammatory factor 1-like
5312	ACADS	0.1525	0.3386	acyl-CoA dehydrogenase, C-2 to C-3 short chain
5313	RNF103	0.1517	0.3848	ring finger protein 103
5314	MYO19	0.1517	0.3094	myosin XIX
5315	KCNJ5	0.1517	0.386	potassium inwardly-rectifying channel, subfamily J, member 5
5316	HTR2A	0.1517	0.2954	5-hydroxytryptamine (serotonin) receptor 2A, G protein-coupled
5317	CXADR	0.1517	0.2455	coxsackie virus and adenovirus receptor
5318	ZNF474	0.1514	0.2797	zinc finger protein 474
5319	ZNF816	0.1509	0.4094	zinc finger protein 816
5320	RXRB	0.1508	0.2835	retinoid X receptor, beta
5321	OBFC1	0.1508	0.3135	oligonucleotide/oligosaccharide-binding fold containing 1
5322	GPER	0.1508	0.2897	G protein-coupled estrogen receptor 1
5323	ERN2	0.1508	0.3575	endoplasmic reticulum to nucleus signaling 2
5324	DDC	0.1508	0.2659	dopa decarboxylase (aromatic L-amino acid decarboxylase)
5325	ZFP106	0.15	0.4165	zinc finger protein 106 homolog (mouse)
5326	RNPC3	0.15	0.3331	RNA-binding region (RNP1, RRM) containing 3
5327	REM1	0.15	0.3745	RAS (RAD and GEM)-like GTP-binding 1
5328	NUDT9	0.15	0.4273	nudix (nucleoside diphosphate linked moiety X)-type motif 9
5329	EPSTI1	0.15	0.267	epithelial stromal interaction 1 (breast)
5330	DMTF1	0.15	0.4184	cyclin D binding myb-like transcription factor 1
5331	CNPPD1	0.15	0.2767	cyclin Pas1/PHO80 domain containing 1
5332	BEAN1	0.15	0.2306	brain expressed, associated with NEDD4, 1
5333	RABGAP1	0.1492	0.4232	RAB GTPase activating protein 1
5334	PIGC	0.1492	0.4285	phosphatidylinositol glycan anchor biosynthesis, class C
5335	OPLAH	0.1492	0.2673	5-oxoprolinase (ATP-hydrolysing)
5336	HOXA7	0.1492	0.161	homeobox A7
5337	DHRS11	0.1492	0.2989	dehydrogenase/reductase (SDR family) member 11
5338	CLCA1	0.1492	0.3169	chloride channel accessory 1
5339	CD74	0.1492	0.2589	CD74 molecule, major histocompatibility complex, class II invariant chain
5340	NT5DC1	0.1488	0.4425	5'-nucleotidase domain containing 1
5341	DLL1	0.1488	0.1623	delta-like 1 (Drosophila)
5342	TLR1	0.1483	0.3034	toll-like receptor 1
5343	NOP10	0.1483	0.3657	NOP10 ribonucleoprotein homolog (yeast)
5344	MPDU1	0.1483	0.3529	mannose-P-dolichol utilization defect 1
5345	EFCAB2	0.1483	0.3345	EF-hand calcium binding domain 2
5346	TRIML2	0.1475	0.2057	tripartite motif family-like 2
5347	TMEM88	0.1475	0.3137	transmembrane protein 88
5348	MOGAT2	0.1475	0.2768	monoacylglycerol O-acyltransferase 2
5349	INF2	0.1475	0.3478	inverted formin, FH2 and WH2 domain containing
5350	EFHA2	0.1475	0.3473	EF-hand domain family, member A2
5351	CUL4B	0.1475	0.4443	cullin 4B
5352	BET1L	0.1475	0.2854	blocked early in transport 1 homolog (S. cerevisiae)-like
5353	PRSS3P2	0.1473	0.3096	protease, serine, 3 pseudogene 2
5354	TPMT	0.1467	0.2977	thiopurine S-methyltransferase
5355	THG1L	0.1467	0.3433	tRNA-histidine guanylyltransferase 1-like (S. cerevisiae)
5356	SPON2	0.1467	0.2214	spondin 2, extracellular matrix protein
5357	N6AMT1	0.1467	0.3724	N-6 adenine-specific DNA methyltransferase 1 (putative)
5358	ERBB4	0.1467	0.2396	v-erb-a erythroblastic leukemia viral oncogene homolog 4 (avian)
5359	C18orf25	0.1467	0.369	chromosome 18 open reading frame 25
5360	ZNF319	0.1462	0.2093	zinc finger protein 319
5361	OSTBETA	0.1462	0.1508	organic solute transporter beta
5362	IL34	0.1462	0.3266	interleukin 34
5363	ELOVL3	0.1462	0.1608	ELOVL fatty acid elongase 3
5364	DANCR	0.1462	0.3503	differentiation antagonizing non-protein coding RNA
5365	SYNE1	0.1458	0.2818	spectrin repeat containing, nuclear envelope 1
5366	SLC5A2	0.1458	0.3757	solute carrier family 5 (sodium/glucose cotransporter), member 2
5367	RAB32	0.1458	0.2612	RAB32, member RAS oncogene family
5368	PLG	0.1458	0.261	plasminogen
5369	PDXDC1	0.1458	0.4262	pyridoxal-dependent decarboxylase domain containing 1
5370	PAX4	0.1458	0.3359	paired box 4
5371	KDM5B	0.1458	0.3845	lysine (K)-specific demethylase 5B
5372	CSRP2	0.1458	0.1709	cysteine and glycine-rich protein 2
5373	CDK12	0.1458	0.4285	cyclin-dependent kinase 12
5374	SRPRB	0.145	0.4045	signal recognition particle receptor, B subunit

5375	LGR5	0.145	0.2677	leucine-rich repeat containing G protein-coupled receptor 5
5376	HOXD10	0.145	0.294	homeobox D10
5377	GYLTL1B	0.145	0.2892	glycosyltransferase-like 1B
5378	GPRASP1	0.145	0.3093	G protein-coupled receptor associated sorting protein 1
5379	GLA	0.145	0.3279	galactosidase, alpha
5380	ARSA	0.145	0.3572	arylsulfatase A
5381	ACOT4	0.145	0.1569	acyl-CoA thioesterase 4
5382	UNC50	0.1442	0.4523	unc-50 homolog (C. elegans)
5383	TRAPPC11	0.1442	0.4816	trafficking protein particle complex 11
5384	RASSF8	0.1442	0.1952	Ras association (RalGDS/AF-6) domain family (N-terminal) member 8
5385	PPP1R14D	0.1442	0.3277	protein phosphatase 1, regulatory (inhibitor) subunit 14D
5386	IFNA8	0.1442	0.1374	interferon, alpha 8
5387	CXorf36	0.1442	0.3791	chromosome X open reading frame 36
5388	CNNM2	0.1442	0.2495	cyclin M2
5389	CD40LG	0.1442	0.3226	CD40 ligand
5390	A4GALT	0.1442	0.371	alpha 1,4-galactosyltransferase
5391	SFXN4	0.1437	0.3802	sideroflexin 4
5392	RSBN1L	0.1437	0.4192	round spermatid basic protein 1-like
5393	FNIP2	0.1437	0.3683	folliculin interacting protein 2
5394	RBM41	0.1433	0.4306	RNA binding motif protein 41
5395	HEXA-AS1	0.1433	0.2369	HEXA antisense RNA 1 (non-protein coding)
5396	COPS2	0.1433	0.447	COP9 constitutive photomorphogenic homolog subunit 2 (Arabidopsis)
5397	BCL2	0.1433	0.1976	B-cell CLL/lymphoma 2
5398	TRIM26	0.1425	0.2749	tripartite motif containing 26
5399	TEPP	0.1425	0.2851	testis, prostate and placenta expressed
5400	SALL1	0.1425	0.1043	sal-like 1 (Drosophila)
5401	PIGS	0.1425	0.3116	phosphatidylinositol glycan anchor biosynthesis, class S
5402	P2RX4	0.1425	0.2846	purinergic receptor P2X, ligand-gated ion channel, 4
5403	HCRTR2	0.1425	0.3146	hypocretin (orexin) receptor 2
5404	FAM98C	0.1425	0.2777	family with sequence similarity 98, member C
5405	DCAF12L1	0.1425	0.0963	DDB1 and CUL4 associated factor 12-like 1
5406	C5orf56	0.1425	0.1926	chromosome 5 open reading frame 56
5407	C17orf58	0.1425	0.3194	chromosome 17 open reading frame 58
5408	ASB7	0.1425	0.3183	ankyrin repeat and SOCS box containing 7
5409	ZNF701	0.1417	0.2983	zinc finger protein 701
5410	UGCG	0.1417	0.3645	UDP-glucose ceramide glucosyltransferase
5411	RFX7	0.1417	0.4361	regulatory factor X, 7
5412	PSME1	0.1417	0.3499	proteasome (prosome, macropain) activator subunit 1 (PA28 alpha)
5413	PSG4	0.1417	0.277	pregnancy specific beta-1-glycoprotein 4
5414	HNF1B	0.1417	0.361	HNF1 homeobox B
5415	GIGYF2	0.1417	0.4117	GRB10 interacting GYF protein 2
5416	ZC3H12C	0.1412	0.3943	zinc finger CCCH-type containing 12C
5417	TSPAN18	0.1412	0.2702	tetraspanin 18
5418	SPOCD1	0.1412	0.2669	SPOC domain containing 1
5419	KIAA1024	0.1409	0.1731	KIAA1024
5420	VGLL1	0.1408	0.3171	vestigial like 1 (Drosophila)
5421	TSTA3	0.1408	0.3476	tissue specific transplantation antigen P35B
5422	NR2F1	0.1408	0.2584	nuclear receptor subfamily 2, group F, member 1
5423	MASP1	0.1408	0.3673	mannan-binding lectin serine peptidase 1 (C4/C2 activating component of Ra-reactive factor)
5424	ITGB1BP2	0.1408	0.174	integrin beta 1 binding protein (melusin) 2
5425	CNTLN	0.1408	0.298	centlein, centrosomal protein
5426	TRIM54	0.14	0.246	tripartite motif containing 54
5427	TBC1D17	0.14	0.3182	TBC1 domain family, member 17
5428	TAT	0.14	0.2135	tyrosine aminotransferase
5429	SH3KBP1	0.14	0.3276	SH3-domain kinase binding protein 1
5430	SEC23IP	0.14	0.4647	SEC23 interacting protein
5431	MTM1	0.14	0.4436	myotubularin 1
5432	LRRC8A	0.14	0.2409	leucine rich repeat containing 8 family, member A
5433	KLF11	0.14	0.2824	Kruppel-like factor 11
5434	FHOD3	0.14	0.1772	formin homology 2 domain containing 3
5435	FAHD1	0.14	0.4432	fumarylacetoacetate hydrolase domain containing 1
5436	AMDHD1	0.14	0.076	amidohydrolase domain containing 1
5437	SUCO	0.1392	0.4393	SUN domain containing ossification factor
5438	RER1	0.1392	0.4074	RER1 retention in endoplasmic reticulum 1 homolog (S. cerevisiae)
5439	MUC2	0.1392	0.3535	mucin 2, oligomeric mucus/gel-forming
5440	MASP2	0.1392	0.3655	mannan-binding lectin serine peptidase 2
5441	MAK	0.1392	0.1824	male germ cell-associated kinase
5442	HSPA12A	0.1392	0.2144	heat shock 70kDa protein 12A
5443	GDF15	0.1392	0.1939	growth differentiation factor 15
5444	TMEM55A	0.1388	0.4182	transmembrane protein 55A
5445	TMEM27	0.1388	0.1777	transmembrane protein 27
5446	PLEKHG2	0.1388	0.3094	pleckstrin homology domain containing, family G (with RhoGef domain) member 2
5447	BTBD6	0.1388	0.2877	BTB (POZ) domain containing 6
5448	ZNF224	0.1383	0.3789	zinc finger protein 224
5449	FICD	0.1383	0.2179	FIC domain containing
5450	DSCR6	0.1383	0.1967	Down syndrome critical region gene 6
5451	DNAJC1	0.1383	0.3515	DnaJ (Hsp40) homolog, subfamily C, member 1
5452	CX3CL1	0.1383	0.3113	chemokine (C-X3-C motif) ligand 1
5453	ZFP90	0.1375	0.4291	zinc finger protein 90 homolog (mouse)
5454	RWDD2B	0.1375	0.3592	RWD domain containing 2B
5455	PRO1768	0.1375	0.3406	PRO1768
5456	PITPNM3	0.1375	0.2401	PITPNM family member 3
5457	MYOT	0.1375	0.1298	myotilin
5458	MAB21L1	0.1375	0.1709	mab-21-like 1 (C. elegans)

5459	IGFBP2	0.1375	0.2133	insulin-like growth factor binding protein 2, 36kDa
5460	IBSP	0.1375	0.323	integrin-binding sialoprotein
5461	FBXW5	0.1375	0.3269	F-box and WD repeat domain containing 5
5462	AMPD1	0.1375	0.2518	adenosine monophosphate deaminase 1
5463	AKT2	0.1375	0.2929	v-akt murine thymoma viral oncogene homolog 2
5464	AGPAT2	0.1375	0.3526	1-acylglycerol-3-phosphate O-acyltransferase 2 (lysophosphatidic acid acyltransferase, beta)
5465	GATC	0.1371	0.3266	glutamyl-tRNA(Gln) amidotransferase, subunit C homolog (bacterial)
5466	WDR60	0.1367	0.3017	WD repeat domain 60
5467	URGCP	0.1367	0.2853	upregulator of cell proliferation
5468	TBX1	0.1367	0.3795	T-box 1
5469	SLC22A8	0.1367	0.3677	solute carrier family 22 (organic anion transporter), member 8
5470	NR1H4	0.1367	0.2695	nuclear receptor subfamily 1, group H, member 4
5471	NPY5R	0.1367	0.2514	neuropeptide Y receptor Y5
5472	LSR	0.1367	0.2514	lipolysis stimulated lipoprotein receptor
5473	CXCL13	0.1367	0.18	chemokine (C-X-C motif) ligand 13
5474	ECRP	0.1364	0.3488	ribonuclease, RNase A family, 2 (liver, eosinophil-derived neurotoxin) pseudogene
5475	SFXN2	0.1363	0.2931	sideroflexin 2
5476	ROMO1	0.1363	0.3327	reactive oxygen species modulator 1
5477	LINC00116	0.1363	0.2384	long intergenic non-protein coding RNA 116
5478	INGX	0.1363	0.273	inhibitor of growth family, X-linked, pseudogene
5479	DNAJC21	0.1363	0.4663	DnaJ (Hsp40) homolog, subfamily C, member 21
5480	BPNT1	0.1363	0.4186	3'(2'), 5'-bisphosphate nucleotidase 1
5481	ST6GALNAC4	0.1358	0.2858	ST6 (alpha-N-acetyl-neuraminyl-2,3-beta-galactosyl-1,3)-N-acetylgalactosaminide alpha-2,6-sialyltransferase 4
5482	NARG2	0.1358	0.4595	NMDA receptor regulated 2
5483	ATG5	0.1358	0.4587	autophagy related 5
5484	ZFP62	0.1357	0.4502	zinc finger protein 62 homolog (mouse)
5485	WDR44	0.135	0.4526	WD repeat domain 44
5486	VPS4B	0.135	0.4533	vacuolar protein sorting 4 homolog B (S. cerevisiae)
5487	RBP7	0.135	0.1487	retinol binding protein 7, cellular
5488	PPIEL	0.135	0.2574	peptidylprolyl isomerase E-like pseudogene
5489	OFD1	0.135	0.3993	oral-facial-digital syndrome 1
5490	N4BP1	0.135	0.321	NEDD4 binding protein 1
5491	MEF2A	0.135	0.396	myocyte enhancer factor 2A
5492	LINS	0.135	0.4287	lines homolog (Drosophila)
5493	EDARADD	0.135	0.1754	EDAR-associated death domain
5494	DMXL1	0.135	0.4523	Dmx-like 1
5495	CEBPA	0.135	0.2324	CCAAT/enhancer binding protein (C/EBP), alpha
5496	CACNG6	0.135	0.3418	calcium channel, voltage-dependent, gamma subunit 6
5497	UBE2D3	0.1342	0.4367	ubiquitin-conjugating enzyme E2D 3
5498	TOM1L2	0.1342	0.4063	target of myb1-like 2 (chicken)
5499	TMEM209	0.1342	0.4414	transmembrane protein 209
5500	PNP	0.1342	0.3577	purine nucleoside phosphorylase
5501	PCYT1A	0.1342	0.2933	phosphate cytidylyltransferase 1, choline, alpha
5502	PACSIN3	0.1342	0.2861	protein kinase C and casein kinase substrate in neurons 3
5503	IGLL3P	0.1342	0.161	immunoglobulin lambda-like polypeptide 3, pseudogene
5504	CLK4	0.1342	0.4195	CDC-like kinase 4
5505	CHAD	0.1342	0.3376	chondroadherin
5506	SLC7A13	0.1338	0.1355	solute carrier family 7 (anionic amino acid transporter), member 13
5507	GNPNAT1	0.1338	0.4284	glucosamine-phosphate N-acetyltransferase 1
5508	DTX3L	0.1338	0.4035	deltex 3-like (Drosophila)
5509	SERINC1	0.1333	0.4308	serine incorporator 1
5510	PROC	0.1333	0.3141	protein C (inactivator of coagulation factors Va and VIIIa)
5511	MALT1	0.1333	0.433	mucosa associated lymphoid tissue lymphoma translocation gene 1
5512	IFNA7	0.1333	0.2347	interferon, alpha 7
5513	GAS8	0.1333	0.38	growth arrest-specific 8
5514	GRM6	0.1327	0.2814	glutamate receptor, metabotropic 6
5515	TAX1BP1	0.1325	0.446	Tax1 (human T-cell leukemia virus type I) binding protein 1
5516	LOC283481	0.1325	0.1696	uncharacterized LOC283481
5517	LMBRD2	0.1325	0.4432	LMBR1 domain containing 2
5518	IL7	0.1325	0.2581	interleukin 7
5519	GATM	0.1325	0.3035	glycine amidinotransferase (L-arginine:glycine amidinotransferase)
5520	CYP2A7	0.1325	0.2564	cytochrome P450, family 2, subfamily A, polypeptide 7
5521	CCNT2	0.1325	0.4292	cyclin T2
5522	C14orf43	0.1325	0.1984	chromosome 14 open reading frame 43
5523	BCL6B	0.1325	0.3435	B-cell CLL/lymphoma 6, member B
5524	MFSD7	0.1317	0.3225	major facilitator superfamily domain containing 7
5525	MAT2A	0.1317	0.3952	methionine adenosyltransferase II, alpha
5526	IFT20	0.1317	0.3893	intraflagellar transport 20 homolog (Chlamydomonas)
5527	GPD1L	0.1317	0.4176	glycerol-3-phosphate dehydrogenase 1-like
5528	CLCC1	0.1317	0.4634	chloride channel CLIC-like 1
5529	C6orf47	0.1317	0.2864	chromosome 6 open reading frame 47
5530	ALOX12	0.1317	0.2451	arachidonate 12-lipoxygenase
5531	SLC9A7	0.1312	0.1982	solute carrier family 9, subfamily A (NHE7, cation proton antiporter 7), member 7
5532	RNF183	0.1312	0.1999	ring finger protein 183
5533	CCDC149	0.1312	0.2866	coiled-coil domain containing 149
5534	RFTN1	0.1308	0.3297	raftlin, lipid raft linker 1
5535	RAB40B	0.1308	0.3176	RAB40B, member RAS oncogene family
5536	OSTM1	0.1308	0.4419	osteopetrosis associated transmembrane protein 1
5537	NTRK2	0.1308	0.3415	neurotrophic tyrosine kinase, receptor, type 2
5538	CCDC9	0.1308	0.4008	coiled-coil domain containing 9
5539	ZNF492	0.13	0.1877	zinc finger protein 492
5540	TPCN2	0.13	0.2172	two pore segment channel 2
5541	RABGEF1	0.13	0.4189	RAB guanine nucleotide exchange factor (GEF) 1
5542	MORF4L2	0.13	0.4451	mortality factor 4 like 2

5543	CCDC160	0.13	0.1223	coiled-coil domain containing 160
5544	C1orf110	0.13	0.1155	chromosome 1 open reading frame 110
5545	SND1	0.1292	0.3652	staphylococcal nuclease and tudor domain containing 1
5546	PSG2	0.1292	0.2754	pregnancy specific beta-1-glycoprotein 2
5547	MYLIP	0.1292	0.2987	myosin regulatory light chain interacting protein
5548	MIA3	0.1292	0.4371	melanoma inhibitory activity family, member 3
5549	IREB2	0.1292	0.4563	iron-responsive element binding protein 2
5550	ERCC6	0.1292	0.2877	excision repair cross-complementing rodent repair deficiency, complementation group 6
5551	DNAJB6	0.1292	0.4259	DnaJ (Hsp40) homolog, subfamily B, member 6
5552	CA3	0.1292	0.0938	carbonic anhydrase III, muscle specific
5553	KRTAP9-3	0.1287	0.1782	keratin associated protein 9-3
5554	IL17RD	0.1287	0.1919	interleukin 17 receptor D
5555	TLN2	0.1283	0.2992	talin 2
5556	PNPO	0.1283	0.3687	pyridoxamine 5'-phosphate oxidase
5557	MEST	0.1283	0.3494	mesoderm specific transcript homolog (mouse)
5558	MARCH6	0.1283	0.4251	membrane-associated ring finger (C3HC4) 6, E3 ubiquitin protein ligase
5559	LOC257396	0.1283	0	uncharacterized LOC257396
5560	HS3ST3B1	0.1283	0.2563	heparan sulfate (glucosamine) 3-O-sulfotransferase 3B1
5561	HLA-DQB2	0.1283	0.267	major histocompatibility complex, class II, DQ beta 2
5562	CYB5R3	0.1283	0.3265	cytochrome b5 reductase 3
5563	APOL1	0.1283	0.2662	apolipoprotein L, 1
5564	SRC	0.1275	0.3808	v-src sarcoma (Schmidt-Ruppin A-2) viral oncogene homolog (avian)
5565	SOST	0.1275	0.1591	sclerostin
5566	SH2D6	0.1275	0.2685	SH2 domain containing 6
5567	PLCB3	0.1275	0.3076	phospholipase C, beta 3 (phosphatidylinositol-specific)
5568	MOB2	0.1275	0.2965	MOB kinase activator 2
5569	MLH3	0.1275	0.3535	mutL homolog 3 (E. coli)
5570	LDOC1L	0.1275	0.4035	leucine zipper, down-regulated in cancer 1-like
5571	GPC4	0.1275	0.1649	glypican 4
5572	GOLT1B	0.1275	0.4463	golgi transport 1B
5573	CTDSPL2	0.1275	0.4595	CTD (carboxy-terminal domain, RNA polymerase II, polypeptide A) small phosphatase like 2
5574	CDH20	0.1275	0.274	cadherin 20, type 2
5575	CBR4	0.1275	0.4549	carbonyl reductase 4
5576	BTN3A2	0.1275	0.3615	butyrophilin, subfamily 3, member A2
5577	BMF	0.1275	0.258	Bcl2 modifying factor
5578	SLC6A9	0.1267	0.3161	solute carrier family 6 (neurotransmitter transporter, glycine), member 9
5579	PRDM16	0.1267	0.2987	PR domain containing 16
5580	NEDD4L	0.1267	0.2828	neural precursor cell expressed, developmentally down-regulated 4-like, E3 ubiquitin protein ligase
5581	MANEA	0.1267	0.4527	mannosidase, endo-alpha
5582	GP6	0.1267	0.284	glycoprotein VI (platelet)
5583	FAM160B2	0.1267	0.3099	family with sequence similarity 160, member B2
5584	METTL23	0.1262	0.4557	methyltransferase like 23
5585	ICMT	0.1258	0.3956	isoprenylcysteine carboxyl methyltransferase
5586	HOXA4	0.1258	0.1704	homeobox A4
5587	GSPT1	0.1258	0.4469	G1 to S phase transition 1
5588	AIMP1	0.1258	0.4485	aminoacyl tRNA synthetase complex-interacting multifunctional protein 1
5589	PYGO2	0.125	0.2991	pygopus homolog 2 (Drosophila)
5590	MT4	0.125	0.1657	metallothionein 4
5591	MRVI1	0.125	0.2886	murine retrovirus integration site 1 homolog
5592	LIMS1	0.125	0.2968	LIM and senescent cell antigen-like domains 1
5593	HAPLN3	0.125	0.2923	hyaluronan and proteoglycan link protein 3
5594	CIDEC	0.125	0.308	cell death-inducing DFFA-like effector c
5595	CARD18	0.125	0.1624	caspase recruitment domain family, member 18
5596	C15orf29	0.125	0.4345	chromosome 15 open reading frame 29
5597	C11orf96	0.125	0.2111	chromosome 11 open reading frame 96
5598	ASB13	0.125	0.2612	ankyrin repeat and SOCS box containing 13
5599	ARAP2	0.125	0.3693	ArfGAP with RhoGAP domain, ankyrin repeat and PH domain 2
5600	ZNF846	0.1243	0.3259	zinc finger protein 846
5601	SP110	0.1242	0.314	SP110 nuclear body protein
5602	IL8	0.1242	0.2567	interleukin 8
5603	HIP1R	0.1242	0.3742	huntingtin interacting protein 1 related
5604	GPR182	0.1242	0.3161	G protein-coupled receptor 182
5605	EPRS	0.1242	0.4462	glutamyl-prolyl-tRNA synthetase
5606	CYP2E1	0.1242	0.3125	cytochrome P450, family 2, subfamily E, polypeptide 1
5607	CNTN6	0.1242	0.2816	contactin 6
5608	ALOX12B	0.1242	0.3476	arachidonate 12-lipoxygenase, 12R type
5609	ADH6	0.1242	0.3534	alcohol dehydrogenase 6 (class V)
5610	ZNF25	0.1238	0.4398	zinc finger protein 25
5611	PVT1	0.1238	0.2341	Pvt1 oncogene (non-protein coding)
5612	C10orf11	0.1238	0.2118	chromosome 10 open reading frame 11
5613	TSPAN13	0.1233	0.3643	tetraspanin 13
5614	SPHAR	0.1233	0.4419	S-phase response (cyclin related)
5615	PER3	0.1233	0.2654	period homolog 3 (Drosophila)
5616	PDLIM2	0.1233	0.259	PDZ and LIM domain 2 (mystique)
5617	NIPSNAP3B	0.1233	0.3459	nipsnap homolog 3B (C. elegans)
5618	HIRA	0.1233	0.4112	HIR histone cell cycle regulation defective homolog A (S. cerevisiae)
5619	DLK2	0.1233	0.3225	delta-like 2 homolog (Drosophila)
5620	COASY	0.1233	0.3542	CoA synthase
5621	BARX1	0.1233	0.3257	BARX homeobox 1
5622	MTERFD3	0.1225	0.4246	MTERF domain containing 3
5623	MATN1	0.1225	0.3724	matrilin 1, cartilage matrix protein
5624	GYS2	0.1225	0.1986	glycogen synthase 2 (liver)
5625	GLP1R	0.1225	0.3903	glucagon-like peptide 1 receptor
5626	C9orf85	0.1225	0.4328	chromosome 9 open reading frame 85

5627	C2orf43	0.1225	0.4596	chromosome 2 open reading frame 43
5628	BDH1	0.1225	0.2706	3-hydroxybutyrate dehydrogenase, type 1
5629	ZNF274	0.1217	0.3675	zinc finger protein 274
5630	SLC35B1	0.1217	0.4116	solute carrier family 35, member B1
5631	PMF1	0.1217	0.3518	polyamine-modulated factor 1
5632	HMG20B	0.1217	0.3319	high mobility group 20B
5633	LOC285556	0.1214	0.1667	uncharacterized LOC285556
5634	UBXN2A	0.1213	0.4485	UBX domain protein 2A
5635	TRAF7	0.1213	0.3335	TNF receptor-associated factor 7, E3 ubiquitin protein ligase
5636	TMEM234	0.1213	0.2429	transmembrane protein 234
5637	BLOC1S2	0.1213	0.4397	biogenesis of lysosomal organelles complex-1, subunit 2
5638	ZNF81	0.1208	0.3211	zinc finger protein 81
5639	RPS6	0.1208	0.4023	ribosomal protein S6
5640	MDM4	0.1208	0.3618	Mdm4 p53 binding protein homolog (mouse)
5641	FSHR	0.1208	0.3234	follicle stimulating hormone receptor
5642	CASQ1	0.1208	0.2725	calsequestrin 1 (fast-twitch, skeletal muscle)
5643	CA2	0.1208	0.2055	carbonic anhydrase II
5644	ZC3H14	0.12	0.472	zinc finger CCCH-type containing 14
5645	TREX1	0.12	0.3074	three prime repair exonuclease 1
5646	TLK1	0.12	0.4372	tousled-like kinase 1
5647	RAB6A	0.12	0.2699	RAB6A, member RAS oncogene family
5648	OSBPL7	0.12	0.3754	oxysterol binding protein-like 7
5649	NEURL2	0.12	0.2581	neuralized homolog 2 (Drosophila)
5650	KLKB1	0.12	0.2812	kallikrein B, plasma (Fletcher factor) 1
5651	HNRNPH2	0.12	0.4382	heterogeneous nuclear ribonucleoprotein H2 (H')
5652	FYCO1	0.12	0.3222	FYVE and coiled-coil domain containing 1
5653	C1orf53	0.12	0.2208	chromosome 1 open reading frame 53
5654	BMPR1B	0.12	0.2585	bone morphogenetic protein receptor, type IB
5655	ATP10A	0.12	0.2034	ATPase, class V, type 10A
5656	ZNF493	0.1192	0.3528	zinc finger protein 493
5657	TLN1	0.1192	0.3452	talin 1
5658	P2RY6	0.1192	0.3233	pyrimidinergic receptor P2Y, G-protein coupled, 6
5659	KDM5D	0.1192	0.1011	lysine (K)-specific demethylase 5D
5660	HOXB7	0.1192	0.1185	homeobox B7
5661	EDF1	0.1192	0.3807	endothelial differentiation-related factor 1
5662	ARHGEF9	0.1192	0.3833	Cdc42 guanine nucleotide exchange factor (GEF) 9
5663	OR2F2	0.1191	0.3338	olfactory receptor, family 2, subfamily F, member 2
5664	ZNRF2	0.1187	0.3714	zinc and ring finger 2
5665	PAQR7	0.1187	0.2153	progestin and adipoQ receptor family member VII
5666	LOC100127983	0.1187	0.2879	uncharacterized LOC100127983
5667	KRTAP3-1	0.1187	0.3058	keratin associated protein 3-1
5668	GPR83	0.1187	0.145	G protein-coupled receptor 83
5669	TUBGCP2	0.1183	0.3542	tubulin, gamma complex associated protein 2
5670	OR52A1	0.1183	0.3288	olfactory receptor, family 52, subfamily A, member 1
5671	HPS5	0.1183	0.4723	Hermansky-Pudlak syndrome 5
5672	GALE	0.1183	0.3066	UDP-galactose-4-epimerase
5673	AMELX	0.1183	0.1794	amelogenin, X-linked
5674	ZNF641	0.1175	0.2793	zinc finger protein 641
5675	TACR1	0.1175	0.3848	tachykinin receptor 1
5676	SPRR4	0.1175	0.3111	small proline-rich protein 4
5677	SLC17A9	0.1175	0.3468	solute carrier family 17, member 9
5678	PPM1A	0.1175	0.4241	protein phosphatase, Mg2+/Mn2+ dependent, 1A
5679	PDCL	0.1175	0.4645	phosducin-like
5680	MED13	0.1175	0.4114	mediator complex subunit 13
5681	LRRK2	0.1175	0.3093	leucine-rich repeat kinase 2
5682	GMPPA	0.1175	0.357	GDP-mannose pyrophosphorylase A
5683	GALNT13	0.1175	0.2397	UDP-N-acetyl-alpha-D-galactosamine:polypeptide N-acetylgalactosaminyltransferase 13 (GalNAc-T13)
5684	DIRC2	0.1175	0.3796	disrupted in renal carcinoma 2
5685	ACSL5	0.1175	0.2756	acyl-CoA synthetase long-chain family member 5
5686	TMEM208	0.1167	0.3597	transmembrane protein 208
5687	TMEM176B	0.1167	0.2319	transmembrane protein 176B
5688	TCEAL4	0.1167	0.4273	transcription elongation factor A (SII)-like 4
5689	SIRT2	0.1167	0.3912	sirtuin 2
5690	RHPN1-AS1	0.1167	0.3218	RHPN1 antisense RNA 1 (non-protein coding)
5691	NUAK1	0.1167	0.2675	NUAK family, SNF1-like kinase, 1
5692	LAMTOR2	0.1167	0.346	late endosomal/lysosomal adaptor, MAPK and MTOR activator 2
5693	IL37	0.1167	0.3572	interleukin 37
5694	FBXW2	0.1167	0.4731	F-box and WD repeat domain containing 2
5695	CCKAR	0.1167	0.344	cholecystokinin A receptor
5696	ARV1	0.1163	0.444	ARV1 homolog (S. cerevisiae)
5697	ANKFN1	0.1163	0.1556	ankyrin-repeat and fibronectin type III domain containing 1
5698	TRPM1	0.1158	0.2997	transient receptor potential cation channel, subfamily M, member 1
5699	LOC282997	0.1158	0.2146	uncharacterized LOC282997
5700	CDX1	0.1158	0.3726	caudal type homeobox 1
5701	BCMO1	0.1158	0.2683	beta-carotene 15,15'-monooxygenase 1
5702	ATP5G2	0.1158	0.3503	ATP synthase, H+ transporting, mitochondrial Fo complex, subunit C2 (subunit 9)
5703	SLC25A15	0.1156	0.3441	solute carrier family 25 (mitochondrial carrier; ornithine transporter) member 15
5704	SLC6A18	0.115	0.3027	solute carrier family 6, member 18
5705	RUNDC1	0.115	0.4165	RUN domain containing 1
5706	PREPL	0.115	0.4652	prolyl endopeptidase-like
5707	PDK1	0.115	0.3046	pyruvate dehydrogenase kinase, isozyme 1
5708	OGG1	0.115	0.2737	8-oxoguanine DNA glycosylase
5709	NUDT16	0.115	0.1886	nudix (nucleoside diphosphate linked moiety X)-type motif 16
5710	LOC100287483	0.115	0	transcription elongation factor B (SIII), polypeptide 1 pseudogene

5711	LECT1	0.115	0.338	leukocyte cell derived chemotaxin 1
5712	LCT	0.115	0.2968	lactase
5713	FAM101B	0.115	0.2337	family with sequence similarity 101, member B
5714	COMMD1	0.115	0.393	copper metabolism (Murr1) domain containing 1
5715	C4orf29	0.115	0.3926	chromosome 4 open reading frame 29
5716	BROX	0.115	0.3786	BRO1 domain and CAAX motif containing
5717	ANKRD29	0.115	0.2271	ankyrin repeat domain 29
5718	ZNF665	0.1142	0.2403	zinc finger protein 665
5719	SIX2	0.1142	0.3187	SIX homeobox 2
5720	IPO7	0.1142	0.4569	importin 7
5721	EI24	0.1142	0.4267	etoposide induced 2.4 mRNA
5722	DCAF8	0.1142	0.3628	DDB1 and CUL4 associated factor 8
5723	CHST7	0.1142	0.1832	carbohydrate (N-acetylglucosamine 6-O) sulfotransferase 7
5724	VPS29	0.1138	0.4525	vacuolar protein sorting 29 homolog (S. cerevisiae)
5725	UBTD2	0.1138	0.4578	ubiquitin domain containing 2
5726	PM20D2	0.1138	0.4346	peptidase M20 domain containing 2
5727	FRMD3	0.1138	0.1985	FERM domain containing 3
5728	ATP13A5	0.1138	0.2781	ATPase type 13A5
5729	AAED1	0.1138	0.4232	AhpC/TSA antioxidant enzyme domain containing 1
5730	UTS2	0.1133	0.1887	urotensin 2
5731	PSEN1	0.1133	0.4332	presenilin 1
5732	PROZ	0.1133	0.3433	protein Z, vitamin K-dependent plasma glycoprotein
5733	PPP1CA	0.1133	0.3968	protein phosphatase 1, catalytic subunit, alpha isozyme
5734	ITSN1	0.1133	0.3632	intersectin 1 (SH3 domain protein)
5735	HOXD8	0.1133	0.1387	homeobox D8
5736	HEYL	0.1133	0.392	hairy/enhancer-of-split related with YRPW motif-like
5737	EMC1	0.1133	0.4296	ER membrane protein complex subunit 1
5738	ARPC1A	0.1133	0.4295	actin related protein 2/3 complex, subunit 1A, 41kDa
5739	ABCC2	0.1133	0.227	ATP-binding cassette, sub-family C (CFTR/MRP), member 2
5740	ZNF215	0.1125	0.2354	zinc finger protein 215
5741	SPIN4	0.1125	0.4228	spindlin family, member 4
5742	SESN3	0.1125	0.3433	sestrin 3
5743	SAT2	0.1125	0.3332	spermidine/spermine N1-acetyltransferase family member 2
5744	RAD52	0.1125	0.2975	RAD52 homolog (S. cerevisiae)
5745	IMPG2	0.1125	0.3346	interphotoreceptor matrix proteoglycan 2
5746	ELOVL4	0.1125	0.2614	ELOVL fatty acid elongase 4
5747	TMED1	0.1117	0.3109	transmembrane emp24 protein transport domain containing 1
5748	TLE6	0.1117	0.3473	transducin-like enhancer of split 6 (E(sp1) homolog, Drosophila)
5749	PTP4A1	0.1117	0.4106	protein tyrosine phosphatase type IVA, member 1
5750	EXD2	0.1117	0.3389	exonuclease 3'-5' domain containing 2
5751	DUSP5	0.1117	0.2848	dual specificity phosphatase 5
5752	CCL19	0.1117	0.2895	chemokine (C-C motif) ligand 19
5753	BCAS1	0.1117	0.2581	breast carcinoma amplified sequence 1
5754	IGFL1	0.1114	0.3146	IGF-like family member 1
5755	ZNF879	0.1112	0.3808	zinc finger protein 879
5756	SAMD9L	0.1112	0.3424	sterile alpha motif domain containing 9-like
5757	KCTD6	0.1112	0.4178	potassium channel tetramerisation domain containing 6
5758	FBXO36	0.1112	0.2039	F-box protein 36
5759	TSPAN14	0.1108	0.2819	tetraspanin 14
5760	TP53I11	0.1108	0.3511	tumor protein p53 inducible protein 11
5761	STAT1	0.1108	0.4014	signal transducer and activator of transcription 1, 91kDa
5762	RANBP17	0.1108	0.2305	RAN binding protein 17
5763	PCDH11Y	0.1108	0.0682	protocadherin 11 Y-linked
5764	LOC339524	0.1108	0.3178	uncharacterized LOC339524
5765	FHL3	0.1108	0.3511	four and a half LIM domains 3
5766	CNDP2	0.1108	0.3916	CNDP dipeptidase 2 (metallopeptidase M20 family)
5767	CCDC41	0.1108	0.4527	coiled-coil domain containing 41
5768	C9orf156	0.1108	0.3493	chromosome 9 open reading frame 156
5769	A1CF	0.1108	0.3289	APOBEC1 complementation factor
5770	ZNF814	0.11	0.3811	zinc finger protein 814
5771	WDR35	0.11	0.4178	WD repeat domain 35
5772	TMEM25	0.11	0.2644	transmembrane protein 25
5773	SLC9A8	0.11	0.2969	solute carrier family 9, subfamily A (NHE8, cation proton antiporter 8), member 8
5774	PCED1A	0.11	0.2767	PC-esterase domain containing 1A
5775	NR1D1	0.11	0.3413	nuclear receptor subfamily 1, group D, member 1
5776	NEDD1	0.11	0.4682	neural precursor cell expressed, developmentally down-regulated 1
5777	MYC	0.11	0.3495	v-myc myelocytomatosis viral oncogene homolog (avian)
5778	MAGIX	0.11	0.3637	MAGI family member, X-linked
5779	KRTDAP	0.11	0.2645	keratinocyte differentiation-associated protein
5780	COX6A2	0.11	0.3426	cytochrome c oxidase subunit VIa polypeptide 2
5781	C16orf48	0.11	0.262	chromosome 16 open reading frame 48
5782	ARMC10	0.11	0.4188	armadillo repeat containing 10
5783	ACTR10	0.11	0.4706	actin-related protein 10 homolog (S. cerevisiae)
5784	TBC1D12	0.1092	0.3925	TBC1 domain family, member 12
5785	RPA4	0.1092	0.1622	replication protein A4, 30kDa
5786	KLRD1	0.1092	0.2848	killer cell lectin-like receptor subfamily D, member 1
5787	FLVCR2	0.1092	0.2265	feline leukemia virus subgroup C cellular receptor family, member 2
5788	FER1L4	0.1092	0.3734	fer-1-like 4 (C. elegans) pseudogene
5789	HEATR7A	0.1088	0.1777	HEAT repeat containing 7A
5790	ARHGEF37	0.1088	0.2937	Rho guanine nucleotide exchange factor (GEF) 37
5791	RPS24	0.1083	0.3807	ribosomal protein S24
5792	MOSPD1	0.1083	0.4369	motile sperm domain containing 1
5793	DYNLT1	0.1083	0.4282	dynein, light chain, Tctex-type 1
5794	CACNA1D	0.1083	0.3146	calcium channel, voltage-dependent, L type, alpha 1D subunit

5795	ACTB	0.1083	0.392	actin, beta
5796	ACE2	0.1083	0.2357	angiotensin I converting enzyme (peptidyl-dipeptidase A) 2
5797	ZSCAN12	0.1082	0.3046	zinc finger and SCAN domain containing 12
5798	ZNF780A	0.1075	0.4473	zinc finger protein 780A
5799	ULBP1	0.1075	0.196	UL16 binding protein 1
5800	UBQLN2	0.1075	0.4544	ubiquilin 2
5801	TRAF6	0.1075	0.379	TNF receptor-associated factor 6, E3 ubiquitin protein ligase
5802	RPL14	0.1075	0.3625	ribosomal protein L14
5803	RAVER2	0.1075	0.4252	ribonucleoprotein, PTB-binding 2
5804	RAB1B	0.1075	0.3567	RAB1B, member RAS oncogene family
5805	PTH2R	0.1075	0.1588	parathyroid hormone 2 receptor
5806	LRP12	0.1075	0.417	low density lipoprotein receptor-related protein 12
5807	KRTAP3-3	0.1075	0.1431	keratin associated protein 3-3
5808	KCNJ3	0.1075	0.3143	potassium inwardly-rectifying channel, subfamily J, member 3
5809	GOLGA3	0.1075	0.3207	golgin A3
5810	DOPEY2	0.1075	0.3288	dopey family member 2
5811	COMTD1	0.1075	0.3154	catechol-O-methyltransferase domain containing 1
5812	CLK1	0.1075	0.4101	CDC-like kinase 1
5813	BACE1	0.1075	0.2876	beta-site APP-cleaving enzyme 1
5814	TLL1	0.1071	0.2074	tolloid-like 1
5815	PRORSD1P	0.1071	0.2491	prolyl-tRNA synthetase associated domain containing 1, pseudogene
5816	ZNF257	0.1067	0.1886	zinc finger protein 257
5817	UBA6	0.1067	0.4534	ubiquitin-like modifier activating enzyme 6
5818	SOX13	0.1067	0.3168	SRY (sex determining region Y)-box 13
5819	SCTR	0.1067	0.3752	secretin receptor
5820	LOC346329	0.1067	0	guanine nucleotide binding protein (G protein), alpha 11 (Gq class) pseudogene
5821	HILPDA	0.1067	0.2621	hypoxia inducible lipid droplet-associated
5822	ANGEL2	0.1067	0.4746	angel homolog 2 (Drosophila)
5823	AAAS	0.1067	0.358	achalasia, adrenocortical insufficiency, alacrimia
5824	PCDHGA1	0.1064	0.3554	protocadherin gamma subfamily A, 1
5825	ZNF830	0.1063	0.4509	zinc finger protein 830
5826	RFTN2	0.1063	0.2872	raftlin family member 2
5827	DOCK7	0.1063	0.475	dedicator of cytokinesis 7
5828	C15orf62	0.1063	0.3121	chromosome 15 open reading frame 62
5829	STAB2	0.1058	0.3694	stabilin 2
5830	SLC2A11	0.1058	0.2711	solute carrier family 2 (facilitated glucose transporter), member 11
5831	SERPINB10	0.1058	0.3072	serpin peptidase inhibitor, clade B (ovalbumin), member 10
5832	MYF5	0.1058	0.2939	myogenic factor 5
5833	HSPA2	0.1058	0.2487	heat shock 70kDa protein 2
5834	ETS1	0.1058	0.3152	v-ets erythroblastosis virus E26 oncogene homolog 1 (avian)
5835	EIF2B2	0.1058	0.4387	eukaryotic translation initiation factor 2B, subunit 2 beta, 39kDa
5836	EIF1AX	0.1058	0.4495	eukaryotic translation initiation factor 1A, X-linked
5837	CCDC56	0.1058	0.4151	coiled-coil domain containing 56
5838	ZKSCAN3	0.105	0.3055	zinc finger with KRAB and SCAN domains 3
5839	SKAP2	0.105	0.4389	src kinase associated phosphoprotein 2
5840	MYPN	0.105	0.263	myopalladin
5841	IDH3G	0.105	0.3613	isocitrate dehydrogenase 3 (NAD+) gamma
5842	GGA2	0.105	0.3695	golgi-associated, gamma adaptin ear containing, ARF binding protein 2
5843	FAM160B1	0.105	0.4093	family with sequence similarity 160, member B1
5844	ENDOD1	0.105	0.3592	endonuclease domain containing 1
5845	CXCR6	0.105	0.3188	chemokine (C-X-C motif) receptor 6
5846	CHST4	0.105	0.3092	carbohydrate (N-acetylglucosamine 6-O) sulfotransferase 4
5847	ATG14	0.105	0.4346	autophagy related 14
5848	ARMC9	0.105	0.2405	armadillo repeat containing 9
5849	ADAM23	0.105	0.2441	ADAM metallopeptidase domain 23
5850	STXBP6	0.1042	0.1358	syntaxin binding protein 6 (amisyn)
5851	SKP1	0.1042	0.4309	S-phase kinase-associated protein 1
5852	DEDD	0.1042	0.3397	death effector domain containing
5853	CD160	0.1042	0.2623	CD160 molecule
5854	BAG5	0.1042	0.467	BCL2-associated athanogene 5
5855	ARHGDIB	0.1042	0.3527	Rho GDP dissociation inhibitor (GDI) beta
5856	VPS37A	0.1037	0.4674	vacuolar protein sorting 37 homolog A (S. cerevisiae)
5857	SLC5A10	0.1037	0.2957	solute carrier family 5 (sodium/glucose cotransporter), member 10
5858	MTFMT	0.1037	0.453	mitochondrial methionyl-tRNA formyltransferase
5859	MIER3	0.1037	0.4568	mesoderm induction early response 1, family member 3
5860	BCDIN3D	0.1037	0.3301	BCDIN3 domain containing
5861	TPD52	0.1033	0.4169	tumor protein D52
5862	TAZ	0.1033	0.3902	tafazzin
5863	SERPINA1	0.1033	0.2626	serpin peptidase inhibitor, clade A (alpha-1 antiproteinase, antitrypsin), member 1
5864	NEK1	0.1033	0.4484	NIMA (never in mitosis gene a)-related kinase 1
5865	HECA	0.1033	0.3886	headcase homolog (Drosophila)
5866	GOLGA4	0.1033	0.4469	golgin A4
5867	C6orf108	0.1033	0.3591	chromosome 6 open reading frame 108
5868	THAP4	0.1025	0.3422	THAP domain containing 4
5869	OR7A5	0.1025	0.338	olfactory receptor, family 7, subfamily A, member 5
5870	MPST	0.1025	0.3186	mercaptopyruvate sulfurtransferase
5871	LOC100130938	0.1025	0.2751	uncharacterized LOC100130938
5872	FBXO18	0.1025	0.3951	F-box protein, helicase, 18
5873	DUSP18	0.1025	0.2938	dual specificity phosphatase 18
5874	CLDN5	0.1025	0.3515	claudin 5
5875	C2orf74	0.1025	0.3972	chromosome 2 open reading frame 74
5876	ARRDC1	0.1025	0.3297	arrestin domain containing 1
5877	QTRTD1	0.1017	0.4645	queuine tRNA-ribosyltransferase domain containing 1
5878	PPIP5K2	0.1017	0.4704	diphosphoinositol pentakisphosphate kinase 2

5879	NSMAF	0.1017	0.4677	neutral sphingomyelinase (N-SMase) activation associated factor
5880	FOLR3	0.1017	0.3408	folate receptor 3 (gamma)
5881	COPB1	0.1017	0.467	coatomer protein complex, subunit beta 1
5882	ARF6	0.1017	0.364	ADP-ribosylation factor 6
5883	SIPA1L2	0.1013	0.2886	signal-induced proliferation-associated 1 like 2
5884	RAB24	0.1013	0.3041	RAB24, member RAS oncogene family
5885	FAM213B	0.1013	0.2957	family with sequence similarity 213, member B
5886	C3orf17	0.1013	0.4796	chromosome 3 open reading frame 17
5887	ARHGAP27	0.1013	0.2814	Rho GTPase activating protein 27
5888	ZNF614	0.1008	0.4134	zinc finger protein 614
5889	PRDX6	0.1008	0.4283	peroxiredoxin 6
5890	OR1A2	0.1008	0.3438	olfactory receptor, family 1, subfamily A, member 2
5891	MCOLN3	0.1008	0.2305	mucolipin 3
5892	ZNF461	0.1	0.28	zinc finger protein 461
5893	WHAMM	0.1	0.3431	WAS protein homolog associated with actin, golgi membranes and microtubules
5894	TFEC	0.1	0.2725	transcription factor EC
5895	SNX14	0.1	0.4799	sorting nexin 14
5896	SMOC1	0.1	0.3391	SPARC related modular calcium binding 1
5897	SLC43A2	0.1	0.2937	solute carrier family 43, member 2
5898	SLC2A2	0.1	0.2206	solute carrier family 2 (facilitated glucose transporter), member 2
5899	SHF	0.1	0.2497	Src homology 2 domain containing F
5900	PPP1R18	0.1	0.3284	protein phosphatase 1, regulatory subunit 18
5901	MAPKAPK2	0.1	0.3271	mitogen-activated protein kinase-activated protein kinase 2
5902	HOXB8	0.1	0.3324	homeobox B8
5903	ARID2	0.1	0.461	AT rich interactive domain 2 (ARID, RFX-like)
5904	ANKHD1	0.1	0.4499	ankyrin repeat and KH domain containing 1
5905	WDR26	0.0992	0.445	WD repeat domain 26
5906	SLCO1B3	0.0992	0.1281	solute carrier organic anion transporter family, member 1B3
5907	SLC28A2	0.0992	0.2754	solute carrier family 28 (sodium-coupled nucleoside transporter), member 2
5908	NAT8B	0.0992	0.2928	N-acetyltransferase 8B (GCN5-related, putative, gene/pseudogene)
5909	LILRB5	0.0992	0.3132	leukocyte immunoglobulin-like receptor, subfamily B (with TM and ITIM domains), member 5
5910	KMO	0.0992	0.2381	kynurenine 3-monooxygenase (kynurenine 3-hydroxylase)
5911	KCNA1	0.0992	0.358	potassium voltage-gated channel, shaker-related subfamily, member 1 (episodic ataxia with myokymia)
5912	FBXO21	0.0992	0.4688	F-box protein 21
5913	ESYT1	0.0992	0.4208	extended synaptotagmin-like protein 1
5914	DPM3	0.0992	0.3198	dolichyl-phosphate mannosyltransferase polypeptide 3
5915	DENND4A	0.0992	0.4502	DENN/MADD domain containing 4A
5916	ZNF831	0.0988	0.1897	zinc finger protein 831
5917	TMEM167A	0.0988	0.4493	transmembrane protein 167A
5918	RANBP3L	0.0988	0.1925	RAN binding protein 3-like
5919	ARHGEF25	0.0988	0.3472	Rho guanine nucleotide exchange factor (GEF) 25
5920	NPL	0.0983	0.3185	N-acetylneuraminate pyruvate lyase (dihydrodipicolinate synthase)
5921	KYNU	0.0983	0.2854	kynureninase
5922	KRT24	0.0983	0.2603	keratin 24
5923	FGD1	0.0983	0.3296	FYVE, RhoGEF and PH domain containing 1
5924	ABL2	0.0983	0.2989	v-abl Abelson murine leukemia viral oncogene homolog 2
5925	VPS53	0.0975	0.3227	vacuolar protein sorting 53 homolog (S. cerevisiae)
5926	SYNJ2	0.0975	0.2515	synaptojanin 2
5927	HOXC8	0.0975	0.3714	homeobox C8
5928	GBF1	0.0975	0.3416	golgi brefeldin A resistant guanine nucleotide exchange factor 1
5929	FNIP1	0.0975	0.4121	folliculin interacting protein 1
5930	FGF16	0.0975	0.3445	fibroblast growth factor 16
5931	ERP29	0.0975	0.3651	endoplasmic reticulum protein 29
5932	CXorf59	0.0975	0.2634	chromosome X open reading frame 59
5933	LOC100270804	0.0971	0	uncharacterized LOC100270804
5934	UBE4B	0.0967	0.4174	ubiquitination factor E4B
5935	GADD45A	0.0967	0.3366	growth arrest and DNA-damage-inducible, alpha
5936	EXTL2	0.0967	0.4589	exostoses (multiple)-like 2
5937	FLJ39639	0.0964	0.2838	uncharacterized protein FLJ39639
5938	PRMT10	0.0962	0.4668	protein arginine methyltransferase 10 (putative)
5939	EID3	0.0962	0.2815	EP300 interacting inhibitor of differentiation 3
5940	VWA1	0.0958	0.3852	von Willebrand factor A domain containing 1
5941	RGS11	0.0958	0.4017	regulator of G-protein signaling 11
5942	PI4KB	0.0958	0.3747	phosphatidylinositol 4-kinase, catalytic, beta
5943	OTC	0.0958	0.3235	ornithine carbamoyltransferase
5944	IL1B	0.0958	0.2897	interleukin 1, beta
5945	GUCA2A	0.0958	0.3832	guanylate cyclase activator 2A (guanylin)
5946	FOXJ1	0.0958	0.3782	forkhead box J1
5947	DNAJB9	0.0958	0.4168	DnaJ (Hsp40) homolog, subfamily B, member 9
5948	CYB5R2	0.0958	0.1876	cytochrome b5 reductase 2
5949	CDADC1	0.0958	0.3626	cytidine and dCMP deaminase domain containing 1
5950	BHMT	0.0958	0.3424	betaine--homocysteine S-methyltransferase
5951	TRIL	0.095	0.2669	TLR4 interactor with leucine-rich repeats
5952	TRAPPC2	0.095	0.4463	trafficking protein particle complex 2
5953	TOMM7	0.095	0.3605	translocase of outer mitochondrial membrane 7 homolog (yeast)
5954	RBM4B	0.095	0.399	RNA binding motif protein 4B
5955	PM20D1	0.095	0.1925	peptidase M20 domain containing 1
5956	PLEKHG4	0.095	0.1793	pleckstrin homology domain containing, family G (with RhoGef domain) member 4
5957	NR3C1	0.095	0.427	nuclear receptor subfamily 3, group C, member 1 (glucocorticoid receptor)
5958	L3MBTL1	0.095	0.2825	l(3)mbt-like 1 (Drosophila)
5959	IARS2	0.095	0.4825	isoleucyl-tRNA synthetase 2, mitochondrial
5960	HRG	0.095	0.3768	histidine-rich glycoprotein
5961	GSDMC	0.095	0.2793	gasdermin C
5962	GJC2	0.095	0.3984	gap junction protein, gamma 2, 47kDa

5963	FHOD1	0.095	0.3151	formin homology 2 domain containing 1
5964	EN1	0.095	0.2226	engrailed homeobox 1
5965	CRYAA	0.095	0.3614	crystallin, alpha A
5966	CORO2A	0.095	0.2244	coronin, actin binding protein, 2A
5967	CASP14	0.095	0.2484	caspase 14, apoptosis-related cysteine peptidase
5968	TBC1D5	0.0942	0.4121	TBC1 domain family, member 5
5969	STYK1	0.0942	0.1803	serine/threonine/tyrosine kinase 1
5970	SC5DL	0.0942	0.4224	sterol-C5-desaturase (ERG3 delta-5-desaturase homolog, S. cerevisiae)-like
5971	FGF5	0.0942	0.3653	fibroblast growth factor 5
5972	FAM105A	0.0942	0.2559	family with sequence similarity 105, member A
5973	APMAP	0.0942	0.4281	adipocyte plasma membrane associated protein
5974	ANKRD49	0.0942	0.4462	ankyrin repeat domain 49
5975	XRN2	0.0938	0.476	5'-3' exoribonuclease 2
5976	SNX31	0.0938	0.1461	sorting nexin 31
5977	IDNK	0.0938	0.2857	idnK, gluconokinase homolog (E. coli)
5978	FMNL2	0.0938	0.3987	formin-like 2
5979	ZMAT3	0.0933	0.4073	zinc finger, matrin-type 3
5980	NDRG1	0.0933	0.3024	N-myc downstream regulated 1
5981	KLF17P1	0.0933	0	Kruppel-like factor 17 pseudogene 1
5982	IL2	0.0933	0.2082	interleukin 2
5983	HOXA1	0.0933	0.1204	homeobox A1
5984	GRPR	0.0933	0.3751	gastrin-releasing peptide receptor
5985	CYP4F2	0.0933	0.3297	cytochrome P450, family 4, subfamily F, polypeptide 2
5986	CDHR1	0.0933	0.3554	cadherin-related family member 1
5987	CDH8	0.0933	0.3556	cadherin 8, type 2
5988	BCKDHA	0.0933	0.3184	branched chain keto acid dehydrogenase E1, alpha polypeptide
5989	TMEM91	0.0929	0.3054	transmembrane protein 91
5990	LOC153684	0.0929	0.2253	uncharacterized LOC153684
5991	UXT	0.0925	0.4017	ubiquitously-expressed, prefoldin-like chaperone
5992	USP8	0.0925	0.4781	ubiquitin specific peptidase 8
5993	RPL8	0.0925	0.401	ribosomal protein L8
5994	LGI2	0.0925	0.3838	leucine-rich repeat LGI family, member 2
5995	HLCS	0.0925	0.2778	holocarboxylase synthetase (biotin-(proprionyl-CoA-carboxylase (ATP-hydrolysing)) ligase)
5996	GGCT	0.0925	0.4349	gamma-glutamylcyclotransferase
5997	EGFL7	0.0925	0.3749	EGF-like-domain, multiple 7
5998	CSRNP1	0.0925	0.292	cysteine-serine-rich nuclear protein 1
5999	TEF	0.0917	0.3796	thyrotrophic embryonic factor
6000	TBX4	0.0917	0.3656	T-box 4
6001	STEAP1	0.0917	0.3024	six transmembrane epithelial antigen of the prostate 1
6002	SLC43A3	0.0917	0.3047	solute carrier family 43, member 3
6003	MRPS27	0.0917	0.4631	mitochondrial ribosomal protein S27
6004	IFIT2	0.0917	0.3117	interferon-induced protein with tetratricopeptide repeats 2
6005	GABRB2	0.0917	0.3302	gamma-aminobutyric acid (GABA) A receptor, beta 2
6006	FGG	0.0917	0.1879	fibrinogen gamma chain
6007	CSGALNACT2	0.0917	0.3844	chondroitin sulfate N-acetylgalactosaminyltransferase 2
6008	ACSM5	0.0917	0.3502	acyl-CoA synthetase medium-chain family member 5
6009	VEPH1	0.0913	0.178	ventricular zone expressed PH domain homolog 1 (zebrafish)
6010	RSPRY1	0.0913	0.4725	ring finger and SPRY domain containing 1
6011	NUPL1	0.0913	0.4538	nucleoporin like 1
6012	LINC00483	0.0909	0.3115	long intergenic non-protein coding RNA 483
6013	SERPINF2	0.0908	0.3664	serpin peptidase inhibitor, clade F (alpha-2 antiplasmin, pigment epithelium derived factor), member 2
6014	SEC16A	0.0908	0.4202	SEC16 homolog A (S. cerevisiae)
6015	GIMAP4	0.0908	0.2847	GTPase, IMAP family member 4
6016	FAM8A1	0.0908	0.3913	family with sequence similarity 8, member A1
6017	AUTS2	0.0908	0.2721	autism susceptibility candidate 2
6018	ARCN1	0.0908	0.4794	archain 1
6019	ACSL3	0.0908	0.4673	acyl-CoA synthetase long-chain family member 3
6020	SRP68	0.09	0.469	signal recognition particle 68kDa
6021	SPINK1	0.09	0.2171	serine peptidase inhibitor, Kazal type 1
6022	SLC41A2	0.09	0.3545	solute carrier family 41, member 2
6023	SH2D2A	0.09	0.3072	SH2 domain containing 2A
6024	SCMH1	0.09	0.3302	sex comb on midleg homolog 1 (Drosophila)
6025	RGAG4	0.09	0.2827	retrotransposon gag domain containing 4
6026	PADI2	0.09	0.3444	peptidyl arginine deiminase, type II
6027	PABPC1L	0.09	0.242	poly(A) binding protein, cytoplasmic 1-like
6028	GABRA6	0.09	0.3202	gamma-aminobutyric acid (GABA) A receptor, alpha 6
6029	FAM155A	0.09	0.2657	family with sequence similarity 155, member A
6030	CUL5	0.09	0.4695	cullin 5
6031	CCDC3	0.09	0.2703	coiled-coil domain containing 3
6032	VN1R1	0.0892	0.3171	vomeronasal 1 receptor 1
6033	UGP2	0.0892	0.4611	UDP-glucose pyrophosphorylase 2
6034	THRB	0.0892	0.2887	thyroid hormone receptor, beta
6035	MMP13	0.0892	0.2535	matrix metallopeptidase 13 (collagenase 3)
6036	DNAJC15	0.0892	0.4432	DnaJ (Hsp40) homolog, subfamily C, member 15
6037	AICDA	0.0892	0.2905	activation-induced cytidine deaminase
6038	ZNF563	0.0887	0.1974	zinc finger protein 563
6039	UPRT	0.0887	0.489	uracil phosphoribosyltransferase (FUR1) homolog (S. cerevisiae)
6040	PARP9	0.0887	0.4134	poly (ADP-ribose) polymerase family, member 9
6041	ZNF500	0.0883	0.2888	zinc finger protein 500
6042	SLC35C1	0.0883	0.3835	solute carrier family 35, member C1
6043	PHOX2A	0.0883	0.3648	paired-like homeobox 2a
6044	NOD2	0.0883	0.2323	nucleotide-binding oligomerization domain containing 2
6045	NEO1	0.0883	0.3112	neogenin 1
6046	MST1	0.0883	0.3394	macrophage stimulating 1 (hepatocyte growth factor-like)

6047	KAL1	0.0883	0.2989	Kallmann syndrome 1 sequence
6048	CRELD1	0.0883	0.3012	cysteine-rich with EGF-like domains 1
6049	ART1	0.0883	0.3734	ADP-ribosyltransferase 1
6050	TTC39A	0.0875	0.2015	tetratricopeptide repeat domain 39A
6051	TRIO	0.0875	0.3209	trio Rho guanine nucleotide exchange factor
6052	STAG2	0.0875	0.4601	stromal antigen 2
6053	PCSK1	0.0875	0.2175	proprotein convertase subtilisin/kexin type 1
6054	NAV3	0.0875	0.2839	neuron navigator 3
6055	LINC00189	0.0875	0.1057	long intergenic non-protein coding RNA 189
6056	KIAA0125	0.0875	0.2628	KIAA0125
6057	IFRD2	0.0875	0.3676	interferon-related developmental regulator 2
6058	IFIT3	0.0875	0.3203	interferon-induced protein with tetratricopeptide repeats 3
6059	FAM127B	0.0875	0.3398	family with sequence similarity 127, member B
6060	DEFA5	0.0875	0.3282	defensin, alpha 5, Paneth cell-specific
6061	CNTN4	0.0875	0.2288	contactin 4
6062	AQP6	0.0875	0.3948	aquaporin 6, kidney specific
6063	ST7-AS2	0.0871	0.0004	ST7 antisense RNA 2 (non-protein coding)
6064	SEC23B	0.0867	0.4689	Sec23 homolog B (S. cerevisiae)
6065	PF4	0.0867	0.1533	platelet factor 4
6066	KIAA1614	0.0867	0.3783	KIAA1614
6067	ERAP2	0.0867	0.3561	endoplasmic reticulum aminopeptidase 2
6068	CSNK1A1	0.0867	0.4641	casein kinase 1, alpha 1
6069	CCNO	0.0867	0.3149	cyclin O
6070	CCDC64	0.0864	0.3745	coiled-coil domain containing 64
6071	TBX18	0.0862	0.3158	T-box 18
6072	SFTA1P	0.0862	0.2211	surfactant associated 1, pseudogene
6073	RAB42	0.0862	0.1571	RAB42, member RAS oncogene family
6074	LENG9	0.0862	0.3314	leukocyte receptor cluster (LRC) member 9
6075	FOXS1	0.0862	0.3274	forkhead box S1
6076	ZNF467	0.0858	0.3474	zinc finger protein 467
6077	SERP1	0.0858	0.4425	stress-associated endoplasmic reticulum protein 1
6078	PTPRD	0.0858	0.2721	protein tyrosine phosphatase, receptor type, D
6079	CLEC4E	0.0858	0.2838	C-type lectin domain family 4, member E
6080	APOOL	0.0858	0.4324	apolipoprotein O-like
6081	ANK2	0.0858	0.2734	ankyrin 2, neuronal
6082	ZNF629	0.085	0.2906	zinc finger protein 629
6083	SGIP1	0.085	0.2536	SH3-domain GRB2-like (endophilin) interacting protein 1
6084	QSOX1	0.085	0.3259	quiescin Q6 sulfhydryl oxidase 1
6085	PCSK7	0.085	0.3325	proprotein convertase subtilisin/kexin type 7
6086	KCTD15	0.085	0.2379	potassium channel tetramerisation domain containing 15
6087	KCNS3	0.085	0.1841	potassium voltage-gated channel, delayed-rectifier, subfamily S, member 3
6088	IL18R1	0.085	0.2729	interleukin 18 receptor 1
6089	IFNAR1	0.085	0.4405	interferon (alpha, beta and omega) receptor 1
6090	HDGF	0.085	0.3535	hepatoma-derived growth factor
6091	FASTKD2	0.085	0.4723	FAST kinase domains 2
6092	CHST5	0.085	0.3776	carbohydrate (N-acetylglucosamine 6-O) sulfotransferase 5
6093	BTNL3	0.085	0.3823	butyrophilin-like 3
6094	ANP32A-IT1	0.085	0.2117	ANP32A intronic transcript 1 (non-protein coding)
6095	UBA1	0.0842	0.4039	ubiquitin-like modifier activating enzyme 1
6096	PRKAB1	0.0842	0.334	protein kinase, AMP-activated, beta 1 non-catalytic subunit
6097	POP5	0.0842	0.4381	processing of precursor 5, ribonuclease P/MRP subunit (S. cerevisiae)
6098	NMUR1	0.0842	0.3485	neuromedin U receptor 1
6099	ABI1	0.0842	0.4624	abl-interactor 1
6100	NKAIN2	0.0838	0.1216	Na+/K+ transporting ATPase interacting 2
6101	EIF2C2	0.0838	0.3825	eukaryotic translation initiation factor 2C, 2
6102	PADI3	0.0833	0.3642	peptidyl arginine deiminase, type III
6103	COPS6	0.0833	0.4322	COP9 constitutive photomorphogenic homolog subunit 6 (Arabidopsis)
6104	CD200	0.0833	0.2627	CD200 molecule
6105	TBX19	0.0825	0.315	T-box 19
6106	SGCA	0.0825	0.3735	sarcoglycan, alpha (50kDa dystrophin-associated glycoprotein)
6107	OAS2	0.0825	0.2764	2'-5'-oligoadenylate synthetase 2, 69/71kDa
6108	NIPA1	0.0825	0.4366	non imprinted in Prader-Willi/Angelman syndrome 1
6109	MTRR	0.0825	0.4646	5-methyltetrahydrofolate-homocysteine methyltransferase reductase
6110	HIP1	0.0825	0.2939	huntingtin interacting protein 1
6111	APOH	0.0825	0.2795	apolipoprotein H (beta-2-glycoprotein I)
6112	ANO3	0.0825	0.1864	anoctamin 3
6113	ACTR2	0.0825	0.4575	ARP2 actin-related protein 2 homolog (yeast)
6114	NEK3	0.0817	0.3781	NIMA (never in mitosis gene a)-related kinase 3
6115	MYL3	0.0817	0.368	myosin, light chain 3, alkali; ventricular, skeletal, slow
6116	KIR3DL1	0.0817	0.3159	killer cell immunoglobulin-like receptor, three domains, long cytoplasmic tail, 1
6117	JTB	0.0817	0.4284	jumping translocation breakpoint
6118	GUSBP11	0.0817	0.245	glucuronidase, beta pseudogene 11
6119	GCC2	0.0817	0.4517	GRIP and coiled-coil domain containing 2
6120	C11orf16	0.0817	0.383	chromosome 11 open reading frame 16
6121	ACSM1	0.0817	0.3115	acyl-CoA synthetase medium-chain family member 1
6122	OR7E156P	0.0814	0.161	olfactory receptor, family 7, subfamily E, member 156 pseudogene
6123	XKR6	0.0812	0.2092	XK, Kell blood group complex subunit-related family, member 6
6124	SLC6A19	0.0812	0.3512	solute carrier family 6 (neutral amino acid transporter), member 19
6125	CMYA5	0.0812	0.3024	cardiomyopathy associated 5
6126	ACPL2	0.0812	0.4315	acid phosphatase-like 2
6127	LZTFL1	0.0808	0.4758	leucine zipper transcription factor-like 1
6128	KIR2DL4	0.0808	0.3592	killer cell immunoglobulin-like receptor, two domains, long cytoplasmic tail, 4
6129	IL21	0.0808	0.3163	interleukin 21
6130	GNPDA1	0.0808	0.4446	glucosamine-6-phosphate deaminase 1

6131	FAM174B	0.0808	0.283	family with sequence similarity 174, member B
6132	C4BPB	0.0808	0.2671	complement component 4 binding protein, beta
6133	TJP2	0.08	0.4288	tight junction protein 2
6134	SLC2A13	0.08	0.3754	solute carrier family 2 (facilitated glucose transporter), member 13
6135	PCDHB15	0.08	0.2483	protocadherin beta 15
6136	NPEPL1	0.08	0.3684	aminopeptidase-like 1
6137	MYOZ3	0.08	0.3841	myozenin 3
6138	JAZF1	0.08	0.393	JAZF zinc finger 1
6139	FIS1	0.08	0.3717	fission 1 (mitochondrial outer membrane) homolog (S. cerevisiae)
6140	FBXL2	0.08	0.3028	F-box and leucine-rich repeat protein 2
6141	C5orf27	0.08	0.2882	chromosome 5 open reading frame 27
6142	SCFD1	0.0792	0.4737	sec1 family domain containing 1
6143	PCMTD2	0.0792	0.4607	protein-L-isoaspartate (D-aspartate) O-methyltransferase domain containing 2
6144	NOX3	0.0792	0.2915	NADPH oxidase 3
6145	ITGA7	0.0792	0.3461	integrin, alpha 7
6146	INPP4B	0.0792	0.3975	inositol polyphosphate-4-phosphatase, type II, 105kDa
6147	HLA-DMB	0.0792	0.279	major histocompatibility complex, class II, DM beta
6148	AMFR	0.0792	0.3907	autocrine motility factor receptor, E3 ubiquitin protein ligase
6149	ACE	0.0792	0.3796	angiotensin I converting enzyme (peptidyl-dipeptidase A) 1
6150	ACBD4	0.0792	0.3894	acyl-CoA binding domain containing 4
6151	MORN4	0.0787	0.2974	MORN repeat containing 4
6152	FIGN	0.0787	0.2522	fidgetin
6153	C1orf51	0.0787	0.1804	chromosome 1 open reading frame 51
6154	C20orf24	0.0786	0.3743	chromosome 20 open reading frame 24
6155	SRRM2	0.0783	0.331	serine/arginine repetitive matrix 2
6156	SCAND2	0.0783	0.3851	SCAN domain containing 2 pseudogene
6157	RPP25	0.0783	0.3305	ribonuclease P/MRP 25kDa subunit
6158	DTHD1	0.0783	0.1348	death domain containing 1
6159	DNAJB12	0.0783	0.3615	DnaJ (Hsp40) homolog, subfamily B, member 12
6160	CCDC47	0.0783	0.4789	coiled-coil domain containing 47
6161	CAV3	0.0783	0.3813	caveolin 3
6162	ATXN7L1	0.0783	0.2567	ataxin 7-like 1
6163	TSPAN15	0.0775	0.2515	tetraspanin 15
6164	TRHR	0.0775	0.3438	thyrotropin-releasing hormone receptor
6165	RFXANK	0.0775	0.3913	regulatory factor X-associated ankyrin-containing protein
6166	PTAR1	0.0775	0.4577	protein prenyltransferase alpha subunit repeat containing 1
6167	NUP160	0.0775	0.4728	nucleoporin 160kDa
6168	NOS2	0.0775	0.3603	nitric oxide synthase 2, inducible
6169	NDUFA4	0.0775	0.4271	NADH dehydrogenase (ubiquinone) 1 alpha subcomplex, 4, 9kDa
6170	KLHL28	0.0775	0.4195	kelch-like 28 (Drosophila)
6171	GIMAP8	0.0775	0.2613	GTPase, IMAP family member 8
6172	FAM3B	0.0775	0.1608	family with sequence similarity 3, member B
6173	C16orf72	0.0775	0.4456	chromosome 16 open reading frame 72
6174	ACADSB	0.0775	0.4597	acyl-CoA dehydrogenase, short/branched chain
6175	TDRKH	0.0767	0.3309	tudor and KH domain containing
6176	SMURF2	0.0767	0.4593	SMAD specific E3 ubiquitin protein ligase 2
6177	PRKD2	0.0767	0.357	protein kinase D2
6178	POU6F1	0.0767	0.3667	POU class 6 homeobox 1
6179	GPR137B	0.0767	0.421	G protein-coupled receptor 137B
6180	ADARB2	0.0767	0.3825	adenosine deaminase, RNA-specific, B2
6181	YY2	0.0764	0.3196	YY2 transcription factor
6182	ZNF366	0.0763	0.2854	zinc finger protein 366
6183	SHC4	0.0763	0.1994	SHC (Src homology 2 domain containing) family, member 4
6184	LOC100009676	0.0763	0.2513	uncharacterized LOC100009676
6185	FAM19A5	0.0763	0.2717	family with sequence similarity 19 (chemokine (C-C motif)-like), member A5
6186	SIPA1L3	0.0758	0.2889	signal-induced proliferation-associated 1 like 3
6187	REST	0.0758	0.3482	RE1-silencing transcription factor
6188	QARS	0.0758	0.4334	glutaminyl-tRNA synthetase
6189	MMP1	0.0758	0.2426	matrix metallopeptidase 1 (interstitial collagenase)
6190	F13B	0.0758	0.2848	coagulation factor XIII, B polypeptide
6191	CSDC2	0.0758	0.3982	cold shock domain containing C2, RNA binding
6192	B3GNT2	0.0758	0.4397	UDP-GlcNAc:betaGal beta-1,3-N-acetylglucosaminyltransferase 2
6193	ZNF438	0.075	0.3144	zinc finger protein 438
6194	TPRG1	0.075	0.1436	tumor protein p63 regulated 1
6195	TMEM105	0.075	0.3576	transmembrane protein 105
6196	SGSM3	0.075	0.3517	small G protein signaling modulator 3
6197	RAB11FIP3	0.075	0.3323	RAB11 family interacting protein 3 (class II)
6198	OSM	0.075	0.3185	oncostatin M
6199	NSRP1	0.075	0.4958	nuclear speckle splicing regulatory protein 1
6200	MTERFD2	0.075	0.4053	MTERF domain containing 2
6201	MRPS21	0.075	0.4126	mitochondrial ribosomal protein S21
6202	KIAA0753	0.075	0.4332	KIAA0753
6203	IMP3	0.075	0.4364	IMP3, U3 small nucleolar ribonucleoprotein, homolog (yeast)
6204	CER1	0.075	0.3679	cerberus 1, cysteine knot superfamily, homolog (Xenopus laevis)
6205	CEP104	0.075	0.3627	centrosomal protein 104kDa
6206	ATP7B	0.075	0.2848	ATPase, Cu++ transporting, beta polypeptide
6207	TRAF3IP1	0.0742	0.4338	TNF receptor-associated factor 3 interacting protein 1
6208	TMEM251	0.0742	0.4431	transmembrane protein 251
6209	SIGLEC6	0.0742	0.34	sialic acid binding Ig-like lectin 6
6210	MAPKAP1	0.0742	0.4579	mitogen-activated protein kinase associated protein 1
6211	AGL	0.0742	0.4762	amylo-alpha-1, 6-glucosidase, 4-alpha-glucanotransferase
6212	SERPINB12	0.0737	0.308	serpin peptidase inhibitor, clade B (ovalbumin), member 12
6213	OTUD5	0.0737	0.3653	OTU domain containing 5
6214	TRMT13	0.0733	0.4739	tRNA methyltransferase 13 homolog (S. cerevisiae)

6215	STK39	0.0733	0.462	serine threonine kinase 39
6216	SLC24A6	0.0733	0.3916	solute carrier family 24 (sodium/lithium/calcium exchanger), member 6
6217	RPL15	0.0733	0.4544	ribosomal protein L15
6218	PHF10	0.0733	0.4137	PHD finger protein 10
6219	PCDHB1	0.0733	0.3626	protocadherin beta 1
6220	HLA-DPA1	0.0733	0.3235	major histocompatibility complex, class II, DP alpha 1
6221	CD68	0.0733	0.3156	CD68 molecule
6222	RPL37	0.0729	0.4051	ribosomal protein L37
6223	STBD1	0.0725	0.2579	starch binding domain 1
6224	SEC23A	0.0725	0.4718	Sec23 homolog A (S. cerevisiae)
6225	PLIN2	0.0725	0.3873	perilipin 2
6226	NPPB	0.0725	0.3717	natriuretic peptide B
6227	NACA	0.0725	0.4136	nascent polypeptide-associated complex alpha subunit
6228	LOC344595	0.0725	0.2698	uncharacterized LOC344595
6229	LINGO2	0.0725	0.2132	leucine rich repeat and Ig domain containing 2
6230	KIAA0907	0.0725	0.4526	KIAA0907
6231	HORMAD1	0.0725	0.1528	HORMA domain containing 1
6232	HLA-DPB1	0.0725	0.3067	major histocompatibility complex, class II, DP beta 1
6233	GFRA2	0.0725	0.4076	GDNF family receptor alpha 2
6234	FAM126B	0.0725	0.4396	family with sequence similarity 126, member B
6235	DRG2	0.0725	0.3902	developmentally regulated GTP binding protein 2
6236	CLEC2B	0.0725	0.3444	C-type lectin domain family 2, member B
6237	CCL5	0.0725	0.2714	chemokine (C-C motif) ligand 5
6238	BMX	0.0725	0.3273	BMX non-receptor tyrosine kinase
6239	RAB40A	0.0718	0.2792	RAB40A, member RAS oncogene family
6240	UBE3A	0.0717	0.4777	ubiquitin protein ligase E3A
6241	TRIM32	0.0717	0.4546	tripartite motif containing 32
6242	TACR3	0.0717	0.3643	tachykinin receptor 3
6243	STXBP3	0.0717	0.4712	syntaxin binding protein 3
6244	SOX15	0.0717	0.3809	SRY (sex determining region Y)-box 15
6245	SAMD4A	0.0717	0.3152	sterile alpha motif domain containing 4A
6246	PRPH2	0.0717	0.3479	peripherin 2 (retinal degeneration, slow)
6247	MPP3	0.0717	0.3459	membrane protein, palmitoylated 3 (MAGUK p55 subfamily member 3)
6248	KRT76	0.0717	0.3526	keratin 76
6249	CYP2W1	0.0717	0.334	cytochrome P450, family 2, subfamily W, polypeptide 1
6250	CDA	0.0717	0.3368	cytidine deaminase
6251	BICD2	0.0717	0.4288	bicaudal D homolog 2 (Drosophila)
6252	ANGPT2	0.0717	0.224	angiopoietin 2
6253	SLC35F3	0.0712	0.1108	solute carrier family 35, member F3
6254	KIAA1161	0.0712	0.3725	KIAA1161
6255	KBTBD3	0.0712	0.4114	kelch repeat and BTB (POZ) domain containing 3
6256	PPIL6	0.0711	0.2093	peptidylprolyl isomerase (cyclophilin)-like 6
6257	PRAMEF12	0.0709	0.3655	PRAME family member 12
6258	ZNF175	0.0708	0.4506	zinc finger protein 175
6259	SIX1	0.0708	0.2395	SIX homeobox 1
6260	ING4	0.0708	0.3484	inhibitor of growth family, member 4
6261	IL19	0.0708	0.3365	interleukin 19
6262	GORASP1	0.0708	0.3743	golgi reassembly stacking protein 1, 65kDa
6263	DHX29	0.0708	0.4888	DEAH (Asp-Glu-Ala-His) box polypeptide 29
6264	CEMP1	0.0708	0.3523	cementum protein 1
6265	CCDC93	0.0708	0.4515	coiled-coil domain containing 93
6266	C19orf24	0.0708	0.3419	chromosome 19 open reading frame 24
6267	AVEN	0.0708	0.4335	apoptosis, caspase activation inhibitor
6268	AP1S1	0.0708	0.3358	adaptor-related protein complex 1, sigma 1 subunit
6269	ZNF551	0.07	0.4088	zinc finger protein 551
6270	ZNF350	0.07	0.4226	zinc finger protein 350
6271	YAE1D1	0.07	0.4725	Yae1 domain containing 1
6272	TMEM164	0.07	0.3423	transmembrane protein 164
6273	SYNGR1	0.07	0.3817	synaptogyrin 1
6274	SNX6	0.07	0.4713	sorting nexin 6
6275	SLCO4C1	0.07	0.278	solute carrier organic anion transporter family, member 4C1
6276	PTPRE	0.07	0.3587	protein tyrosine phosphatase, receptor type, E
6277	PLEKHH1	0.07	0.2679	pleckstrin homology domain containing, family H (with MyTH4 domain) member 1
6278	OSBPL2	0.07	0.4136	oxysterol binding protein-like 2
6279	MYRIP	0.07	0.2285	myosin VIIA and Rab interacting protein
6280	ITM2A	0.07	0.3111	integral membrane protein 2A
6281	HLA-DRA	0.07	0.3149	major histocompatibility complex, class II, DR alpha
6282	HLA-DQA1	0.07	0.2227	major histocompatibility complex, class II, DQ alpha 1
6283	SLC24A3	0.0692	0.2405	solute carrier family 24 (sodium/potassium/calcium exchanger), member 3
6284	SARM1	0.0692	0.3837	sterile alpha and TIR motif containing 1
6285	MED18	0.0692	0.2417	mediator complex subunit 18
6286	ANKLE2	0.0692	0.4539	ankyrin repeat and LEM domain containing 2
6287	ABI2	0.0692	0.3821	abl-interactor 2
6288	SALL4	0.0688	0.3213	sal-like 4 (Drosophila)
6289	DNAJC25	0.0688	0.4422	DnaJ (Hsp40) homolog, subfamily C , member 25
6290	KRTAP19-3	0.0686	0.3043	keratin associated protein 19-3
6291	C7orf59	0.0686	0.3526	chromosome 7 open reading frame 59
6292	TRIM58	0.0683	0.1182	tripartite motif containing 58
6293	SPINK4	0.0683	0.3336	serine peptidase inhibitor, Kazal type 4
6294	SPAG7	0.0683	0.3443	sperm associated antigen 7
6295	SLC52A2	0.0683	0.3702	solute carrier family 52, riboflavin transporter, member 2
6296	PRICKLE3	0.0683	0.372	prickle homolog 3 (Drosophila)
6297	FLJ32255	0.0683	0.0004	uncharacterized LOC643977
6298	EDDM3A	0.0683	0.355	epididymal protein 3A

6299	CXCL6	0.0683	0.2067	chemokine (C-X-C motif) ligand 6 (granulocyte chemotactic protein 2)
6300	COPS7A	0.0683	0.4323	COP9 constitutive photomorphogenic homolog subunit 7A (Arabidopsis)
6301	ZSCAN18	0.0675	0.3039	zinc finger and SCAN domain containing 18
6302	ZNF503	0.0675	0.3274	zinc finger protein 503
6303	UBL4A	0.0675	0.3716	ubiquitin-like 4A
6304	SLC27A4	0.0675	0.3758	solute carrier family 27 (fatty acid transporter), member 4
6305	PGD	0.0675	0.4322	phosphogluconate dehydrogenase
6306	MARVELD3	0.0675	0.3372	MARVEL domain containing 3
6307	LINC00482	0.0675	0.349	long intergenic non-protein coding RNA 482
6308	KIAA1147	0.0675	0.4303	KIAA1147
6309	HOXC4	0.0675	0.2382	homeobox C4
6310	HAND1	0.0675	0.3522	heart and neural crest derivatives expressed 1
6311	EGR3	0.0675	0.2664	early growth response 3
6312	COX15	0.0675	0.4753	COX15 homolog, cytochrome c oxidase assembly protein (yeast)
6313	COL13A1	0.0675	0.331	collagen, type XIII, alpha 1
6314	CERS5	0.0675	0.4425	ceramide synthase 5
6315	AP3M1	0.0675	0.5007	adaptor-related protein complex 3, mu 1 subunit
6316	ANKRD13D	0.0675	0.3419	ankyrin repeat domain 13 family, member D
6317	ACAD10	0.0675	0.3874	acyl-CoA dehydrogenase family, member 10
6318	SCD5	0.0673	0.2437	stearoyl-CoA desaturase 5
6319	PPIA	0.0673	0.4528	peptidylprolyl isomerase A (cyclophilin A)
6320	LOC643072	0.0671	0.0004	uncharacterized LOC643072
6321	HOXA-AS3	0.0671	0.3254	HOXA cluster antisense RNA 3 (non-protein coding)
6322	VNN3	0.0667	0.351	vanin 3
6323	SLC39A6	0.0667	0.48	solute carrier family 39 (zinc transporter), member 6
6324	PIP4K2B	0.0667	0.3395	phosphatidylinositol-5-phosphate 4-kinase, type II, beta
6325	FAM175B	0.0667	0.481	family with sequence similarity 175, member B
6326	EIF4E2	0.0667	0.4358	eukaryotic translation initiation factor 4E family member 2
6327	C6orf62	0.0667	0.4698	chromosome 6 open reading frame 62
6328	BPIFA1	0.0667	0.3643	BPI fold containing family A, member 1
6329	ACN9	0.0667	0.4316	ACN9 homolog (S. cerevisiae)
6330	LRRC63	0.0662	0.1343	leucine rich repeat containing 63
6331	ZNF407	0.0658	0.3168	zinc finger protein 407
6332	TRIM10	0.0658	0.3902	tripartite motif containing 10
6333	HIC1	0.0658	0.3742	hypermethylated in cancer 1
6334	CPNE1	0.0658	0.3898	copine I
6335	ARSF	0.0658	0.3869	arylsulfatase F
6336	NIPAL4	0.0657	0.3548	NIPA-like domain containing 4
6337	NAT16	0.0657	0.3599	N-acetyltransferase 16 (GCN5-related, putative)
6338	ZNF469	0.065	0.3313	zinc finger protein 469
6339	WIPF2	0.065	0.3473	WAS/WASL interacting protein family, member 2
6340	TM2D3	0.065	0.4644	TM2 domain containing 3
6341	SLC16A11	0.065	0.3554	solute carrier family 16, member 11 (monocarboxylic acid transporter 11)
6342	PHF20	0.065	0.4425	PHD finger protein 20
6343	PCTP	0.065	0.4516	phosphatidylcholine transfer protein
6344	NAT2	0.065	0.3381	N-acetyltransferase 2 (arylamine N-acetyltransferase)
6345	LOC100131825	0.065	0	uncharacterized LOC100131825
6346	COMMD2	0.065	0.4877	COMM domain containing 2
6347	CCR4	0.065	0.3701	chemokine (C-C motif) receptor 4
6348	RBM39	0.0642	0.4558	RNA binding motif protein 39
6349	PI4K2A	0.0642	0.3452	phosphatidylinositol 4-kinase type 2 alpha
6350	PDS5A	0.0642	0.4853	PDS5, regulator of cohesion maintenance, homolog A (S. cerevisiae)
6351	NR1I2	0.0642	0.3861	nuclear receptor subfamily 1, group I, member 2
6352	HLX	0.0642	0.3029	H2.0-like homeobox
6353	GRK5	0.0642	0.3282	G protein-coupled receptor kinase 5
6354	CDKAL1	0.0642	0.4403	CDK5 regulatory subunit associated protein 1-like 1
6355	ATP6V1A	0.0642	0.4628	ATPase, H+ transporting, lysosomal 70kDa, V1 subunit A
6356	AP5M1	0.0642	0.4882	adaptor-related protein complex 5, mu 1 subunit
6357	C8orf58	0.0637	0.2944	chromosome 8 open reading frame 58
6358	TSPAN5	0.0633	0.2992	tetraspanin 5
6359	TAS2R7	0.0633	0.3632	taste receptor, type 2, member 7
6360	SYK	0.0633	0.2718	spleen tyrosine kinase
6361	SIPA1L1	0.0633	0.318	signal-induced proliferation-associated 1 like 1
6362	SERPINB9	0.0633	0.3012	serpin peptidase inhibitor, clade B (ovalbumin), member 9
6363	PFN1	0.0633	0.4119	profilin 1
6364	NEUROD4	0.0633	0.379	neuronal differentiation 4
6365	MBL1P	0.0633	0.3743	mannose-binding lectin (protein A) 1, pseudogene
6366	EEF2	0.0633	0.428	eukaryotic translation elongation factor 2
6367	CLEC11A	0.0633	0.3221	C-type lectin domain family 11, member A
6368	C20orf181	0.0629	0.3674	chromosome 20 open reading frame 181
6369	TRA2A	0.0625	0.4553	transformer 2 alpha homolog (Drosophila)
6370	SEC24B	0.0625	0.4797	SEC24 family, member B (S. cerevisiae)
6371	MECR	0.0625	0.3609	mitochondrial trans-2-enoyl-CoA reductase
6372	ETFB	0.0625	0.3688	electron-transfer-flavoprotein, beta polypeptide
6373	APLNR	0.0625	0.3792	apelin receptor
6374	ZCCHC24	0.0617	0.337	zinc finger, CCHC domain containing 24
6375	TESC	0.0617	0.2634	tescalcin
6376	SLC3A2	0.0617	0.3746	solute carrier family 3 (activators of dibasic and neutral amino acid transport), member 2
6377	RASIP1	0.0617	0.3494	Ras interacting protein 1
6378	HLF	0.0617	0.2945	hepatic leukemia factor
6379	HCG2P7	0.0617	0.0004	HLA complex group 2 pseudogene 7
6380	GPX7	0.0617	0.3484	glutathione peroxidase 7
6381	BPGM	0.0617	0.45	2,3-bisphosphoglycerate mutase
6382	ABCA4	0.0617	0.3566	ATP-binding cassette, sub-family A (ABC1), member 4

6383	SHPRH	0.0613	0.4845	SNF2 histone linker PHD RING helicase, E3 ubiquitin protein ligase
6384	SDR9C7	0.0613	0.2945	short chain dehydrogenase/reductase family 9C, member 7
6385	MGC16121	0.0613	0.2628	uncharacterized protein MGC16121
6386	MBOAT2	0.0613	0.4087	membrane bound O-acyltransferase domain containing 2
6387	LDHD	0.0613	0.3405	lactate dehydrogenase D
6388	ZC4H2	0.0608	0.2705	zinc finger, C4H2 domain containing
6389	PARP16	0.0608	0.3594	poly (ADP-ribose) polymerase family, member 16
6390	KIAA1199	0.0608	0.2845	KIAA1199
6391	GPR31	0.0608	0.3944	G protein-coupled receptor 31
6392	EIF3J	0.0608	0.4688	eukaryotic translation initiation factor 3, subunit J
6393	DDX31	0.0608	0.3566	DEAD (Asp-Glu-Ala-Asp) box polypeptide 31
6394	DCTN5	0.0608	0.4508	dynactin 5 (p25)
6395	COQ10B	0.0608	0.4525	coenzyme Q10 homolog B (S. cerevisiae)
6396	APRT	0.0608	0.3826	adenine phosphoribosyltransferase
6397	AKT3	0.0608	0.3705	v-akt murine thymoma viral oncogene homolog 3 (protein kinase B, gamma)
6398	TSEN2	0.06	0.4566	tRNA splicing endonuclease 2 homolog (S. cerevisiae)
6399	ST6GALNAC6	0.06	0.3694	ST6 (alpha-N-acetyl-neuraminyl-2,3-beta-galactosyl-1,3)-N-acetylgalactosaminide alpha-2,6-sialyltransferase 6
6400	SDS	0.06	0.3445	serine dehydratase
6401	PDP2	0.06	0.429	pyruvate dehyrogenase phosphatase catalytic subunit 2
6402	GGT7	0.06	0.3574	gamma-glutamyltransferase 7
6403	FXYD2	0.06	0.3407	FXYD domain containing ion transport regulator 2
6404	C8orf73	0.06	0.3803	chromosome 8 open reading frame 73
6405	BTBD8	0.06	0.2679	BTB (POZ) domain containing 8
6406	ATP6V1G1	0.06	0.4333	ATPase, H+ transporting, lysosomal 13kDa, V1 subunit G1
6407	GLRB	0.0592	0.3327	glycine receptor, beta
6408	FMR1	0.0592	0.4693	fragile X mental retardation 1
6409	C11orf63	0.0592	0.3135	chromosome 11 open reading frame 63
6410	SYCP2L	0.0587	0.1754	synaptonemal complex protein 2-like
6411	PPM1L	0.0587	0.2577	protein phosphatase, Mg2+/Mn2+ dependent, 1L
6412	FAM110A	0.0587	0.3024	family with sequence similarity 110, member A
6413	C7orf73	0.0587	0.4614	chromosome 7 open reading frame 73
6414	RAD21-AS1	0.0586	0.0004	RAD21 antisense RNA 1 (non-protein coding)
6415	ZNF669	0.0583	0.2876	zinc finger protein 669
6416	ZNF343	0.0583	0.3224	zinc finger protein 343
6417	UBA5	0.0583	0.4807	ubiquitin-like modifier activating enzyme 5
6418	TXNRD3NB	0.0583	0.181	thioredoxin reductase 3 neighbor
6419	REG1A	0.0583	0.3421	regenerating islet-derived 1 alpha
6420	RC3H2	0.0583	0.4759	ring finger and CCCH-type domains 2
6421	PSMB9	0.0583	0.3828	proteasome (prosome, macropain) subunit, beta type, 9 (large multifunctional peptidase 2)
6422	MYLPF	0.0583	0.3732	myosin light chain, phosphorylatable, fast skeletal muscle
6423	MRE11A	0.0583	0.4737	MRE11 meiotic recombination 11 homolog A (S. cerevisiae)
6424	KTN1	0.0583	0.4771	kinectin 1 (kinesin receptor)
6425	CXCL3	0.0583	0.2257	chemokine (C-X-C motif) ligand 3
6426	CLN6	0.0583	0.391	ceroid-lipofuscinosis, neuronal 6, late infantile, variant
6427	APOL2	0.0583	0.3848	apolipoprotein L, 2
6428	AAGAB	0.0583	0.4447	alpha- and gamma-adaptin binding protein
6429	WSB1	0.0575	0.4318	WD repeat and SOCS box containing 1
6430	TPM3	0.0575	0.2512	tropomyosin 3
6431	SVOPL	0.0575	0.1524	SVOP-like
6432	PRMT3	0.0575	0.4691	protein arginine methyltransferase 3
6433	POPDC2	0.0575	0.3022	popeye domain containing 2
6434	NUDT19	0.0575	0.4355	nudix (nucleoside diphosphate linked moiety X)-type motif 19
6435	FRMD7	0.0575	0.2286	FERM domain containing 7
6436	FCHSD1	0.0575	0.342	FCH and double SH3 domains 1
6437	DAGLB	0.0575	0.396	diacylglycerol lipase, beta
6438	CXCR2	0.0575	0.2507	chemokine (C-X-C motif) receptor 2
6439	BBX	0.0575	0.4635	bobby sox homolog (Drosophila)
6440	ANAPC13	0.0575	0.4671	anaphase promoting complex subunit 13
6441	ADTRP	0.0575	0.2194	androgen-dependent TFPI-regulating protein
6442	SHPK	0.0567	0.3533	sedoheptulokinase
6443	S100A1	0.0567	0.3731	S100 calcium binding protein A1
6444	PSD3	0.0567	0.3965	pleckstrin and Sec7 domain containing 3
6445	NYX	0.0567	0.3907	nyctalopin
6446	MIA2	0.0567	0.3385	melanoma inhibitory activity 2
6447	MAX	0.0567	0.3624	MYC associated factor X
6448	KCNG2	0.0567	0.3101	potassium voltage-gated channel, subfamily G, member 2
6449	HOXA6	0.0567	0.3737	homeobox A6
6450	GREB1L	0.0567	0.3666	growth regulation by estrogen in breast cancer-like
6451	DDX3Y	0.0567	0.2249	DEAD (Asp-Glu-Ala-Asp) box polypeptide 3, Y-linked
6452	CHUK	0.0567	0.4815	conserved helix-loop-helix ubiquitous kinase
6453	ATXN3	0.0567	0.4639	ataxin 3
6454	AHCYL1	0.0567	0.4916	adenosylhomocysteinase-like 1
6455	ZNF566	0.0562	0.4695	zinc finger protein 566
6456	HDAC8	0.0562	0.4295	histone deacetylase 8
6457	DNAJC24	0.0558	0.4755	DnaJ (Hsp40) homolog, subfamily C, member 24
6458	BCL2L10	0.0558	0.3784	BCL2-like 10 (apoptosis facilitator)
6459	ADARB1	0.0558	0.3234	adenosine deaminase, RNA-specific, B1
6460	PRR13	0.0557	0.3598	proline rich 13
6461	WIBG	0.055	0.3347	within bgcn homolog (Drosophila)
6462	RPS14	0.055	0.3944	ribosomal protein S14
6463	METTL20	0.055	0.2873	methyltransferase like 20
6464	MED31	0.055	0.3679	mediator complex subunit 31
6465	FAM214B	0.055	0.4111	family with sequence similarity 214, member B
6466	DPCR1	0.055	0.323	diffuse panbronchiolitis critical region 1

6467	CLEC10A	0.055	0.3308	C-type lectin domain family 10, member A
6468	ANKRD13C	0.055	0.4964	ankyrin repeat domain 13C
6469	ZKSCAN1	0.0542	0.4352	zinc finger with KRAB and SCAN domains 1
6470	TTPA	0.0542	0.244	tocopherol (alpha) transfer protein
6471	SNAPC3	0.0542	0.471	small nuclear RNA activating complex, polypeptide 3, 50kDa
6472	FETUB	0.0542	0.2863	fetuin B
6473	EZH1	0.0542	0.3922	enhancer of zeste homolog 1 (Drosophila)
6474	DNASE1	0.0542	0.3498	deoxyribonuclease I
6475	DIDO1	0.0542	0.4285	death inducer-obliterator 1
6476	C3	0.0542	0.3256	complement component 3
6477	ART4	0.0542	0.3389	ADP-ribosyltransferase 4 (Dombrock blood group)
6478	WDFY2	0.0538	0.2574	WD repeat and FYVE domain containing 2
6479	ZBTB33	0.0533	0.4747	zinc finger and BTB domain containing 33
6480	WDR83OS	0.0533	0.4009	WD repeat domain 83 opposite strand
6481	UGT2B15	0.0533	0.278	UDP glucuronosyltransferase 2 family, polypeptide B15
6482	PKLR	0.0533	0.3955	pyruvate kinase, liver and RBC
6483	MTHFSD	0.0533	0.3174	methenyltetrahydrofolate synthetase domain containing
6484	GPD1	0.0533	0.3988	glycerol-3-phosphate dehydrogenase 1 (soluble)
6485	AIDA	0.0529	0.4635	axin interactor, dorsalization associated
6486	ZFP161	0.0525	0.4748	zinc finger protein 161 homolog (mouse)
6487	SPRYD7	0.0525	0.3839	SPRY domain containing 7
6488	PTPRO	0.0525	0.2663	protein tyrosine phosphatase, receptor type, O
6489	MINPP1	0.0525	0.4883	multiple inositol-polyphosphate phosphatase 1
6490	EBLN2	0.0525	0.3368	endogenous Bornavirus-like nucleoprotein 2
6491	CCNB1IP1	0.0525	0.434	cyclin B1 interacting protein 1, E3 ubiquitin protein ligase
6492	TIA1	0.0517	0.4732	TIA1 cytotoxic granule-associated RNA binding protein
6493	PLEKHF1	0.0517	0.3555	pleckstrin homology domain containing, family F (with FYVE domain) member 1
6494	METTL8	0.0517	0.4526	methyltransferase like 8
6495	KCNC3	0.0517	0.384	potassium voltage-gated channel, Shaw-related subfamily, member 3
6496	GCLC	0.0517	0.4626	glutamate-cysteine ligase, catalytic subunit
6497	CDH10	0.0517	0.3227	cadherin 10, type 2 (T2-cadherin)
6498	CASP9	0.0517	0.3164	caspase 9, apoptosis-related cysteine peptidase
6499	AK2	0.0517	0.4572	adenylate kinase 2
6500	TNFRSF6B	0.0514	0.3771	tumor necrosis factor receptor superfamily, member 6b, decoy
6501	TRIM63	0.0512	0.2731	tripartite motif containing 63, E3 ubiquitin protein ligase
6502	KIF7	0.0512	0.3464	kinesin family member 7
6503	CDCA7L	0.0512	0.4469	cell division cycle associated 7-like
6504	ARSI	0.0512	0.3478	arylsulfatase family, member I
6505	VWF	0.0508	0.3623	von Willebrand factor
6506	TAAR5	0.0508	0.3693	trace amine associated receptor 5
6507	SMYD2	0.0508	0.4738	SET and MYND domain containing 2
6508	RAB9A	0.0508	0.469	RAB9A, member RAS oncogene family
6509	IRF1	0.0508	0.3472	interferon regulatory factor 1
6510	IFNA2	0.0508	0.337	interferon, alpha 2
6511	CEP290	0.0508	0.4561	centrosomal protein 290kDa
6512	ATAD2B	0.0508	0.4621	ATPase family, AAA domain containing 2B
6513	TTC18	0.05	0.3091	tetratricopeptide repeat domain 18
6514	TRIM25	0.05	0.3392	tripartite motif containing 25
6515	SGCG	0.05	0.3138	sarcoglycan, gamma (35kDa dystrophin-associated glycoprotein)
6516	PRUNE2	0.05	0.2825	prune homolog 2 (Drosophila)
6517	PANK1	0.05	0.3818	pantothenate kinase 1
6518	MATN1-AS1	0.05	0	MATN1 antisense RNA 1 (non-protein coding)
6519	HOMER3	0.05	0.3274	homer homolog 3 (Drosophila)
6520	ENPP3	0.05	0.2478	ectonucleotide pyrophosphatase/phosphodiesterase 3
6521	DPCD	0.05	0.3594	deleted in primary ciliary dyskinesia homolog (mouse)
6522	C1orf129	0.05	0.2762	chromosome 1 open reading frame 129
6523	BCAR1	0.05	0.384	breast cancer anti-estrogen resistance 1
6524	ABCB1	0.05	0.2838	ATP-binding cassette, sub-family B (MDR/TAP), member 1
6525	ZNF43	0.0492	0.447	zinc finger protein 43
6526	TMPRSS5	0.0492	0.3796	transmembrane protease, serine 5
6527	SSBP2	0.0492	0.4397	single-stranded DNA binding protein 2
6528	SP4	0.0492	0.3907	Sp4 transcription factor
6529	LDB3	0.0492	0.3985	LIM domain binding 3
6530	DKK1	0.0492	0.2715	dickkopf 1 homolog (Xenopus laevis)
6531	CSRNP3	0.0492	0.3027	cysteine-serine-rich nuclear protein 3
6532	CES1P1	0.0492	0.3653	carboxylesterase 1 pseudogene 1
6533	ST7-AS1	0.0487	0.2183	ST7 antisense RNA 1 (non-protein coding)
6534	OMA1	0.0487	0.4834	OMA1 zinc metallopeptidase homolog (S. cerevisiae)
6535	CCDC104	0.0487	0.4746	coiled-coil domain containing 104
6536	CAPSL	0.0487	0.1857	calcyphosine-like
6537	LINC00284	0.0486	0	long intergenic non-protein coding RNA 284
6538	TSPAN2	0.0483	0.3157	tetraspanin 2
6539	TBC1D13	0.0483	0.3676	TBC1 domain family, member 13
6540	SLC22A1	0.0483	0.3156	solute carrier family 22 (organic cation transporter), member 1
6541	SACM1L	0.0483	0.4879	SAC1 suppressor of actin mutations 1-like (yeast)
6542	PPP1R37	0.0483	0.4183	protein phosphatase 1, regulatory subunit 37
6543	NR5A2	0.0483	0.2722	nuclear receptor subfamily 5, group A, member 2
6544	KIAA0087	0.0483	0.2998	KIAA0087
6545	FAM182A	0.0483	0.3072	family with sequence similarity 182, member A
6546	DNPEP	0.0483	0.4064	aspartyl aminopeptidase
6547	CLEC7A	0.0483	0.2934	C-type lectin domain family 7, member A
6548	C4orf6	0.0483	0.329	chromosome 4 open reading frame 6
6549	SETX	0.0475	0.4695	senataxin
6550	RBPMS2	0.0475	0.2432	RNA binding protein with multiple splicing 2

6551	MMP25	0.0475	0.3911	matrix metallopeptidase 25
6552	C14orf118	0.0475	0.3909	chromosome 14 open reading frame 118
6553	C11orf30	0.0475	0.45	chromosome 11 open reading frame 30
6554	TMEM189	0.0471	0.3971	transmembrane protein 189
6555	VAMP5	0.0467	0.344	vesicle-associated membrane protein 5 (myobrevin)
6556	STAU1	0.0467	0.484	staufen, RNA binding protein, homolog 1 (Drosophila)
6557	NKTR	0.0467	0.421	natural killer-tumor recognition sequence
6558	METAP1	0.0467	0.4845	methionyl aminopeptidase 1
6559	GPR183	0.0467	0.3159	G protein-coupled receptor 183
6560	CD19	0.0467	0.323	CD19 molecule
6561	ULK3	0.0463	0.3572	unc-51-like kinase 3 (C. elegans)
6562	PCED1B	0.0463	0.2507	PC-esterase domain containing 1B
6563	VRTN	0.0458	0.3795	vertebrae development homolog (pig)
6564	SLC6A2	0.0458	0.4008	solute carrier family 6 (neurotransmitter transporter, noradrenalin), member 2
6565	LGALS9	0.0458	0.3538	lectin, galactoside-binding, soluble, 9
6566	HPS6	0.0458	0.3352	Hermansky-Pudlak syndrome 6
6567	GPR75	0.0458	0.3518	G protein-coupled receptor 75
6568	FPR3	0.0458	0.3343	formyl peptide receptor 3
6569	MTHFS	0.0457	0.29	5,10-methenyltetrahydrofolate synthetase (5-formyltetrahydrofolate cyclo-ligase)
6570	CXorf30	0.0457	0.3639	chromosome X open reading frame 30
6571	ZC3H12D	0.045	0	zinc finger CCCH-type containing 12D
6572	XIRP2	0.045	0.178	xin actin-binding repeat containing 2
6573	WDR59	0.045	0.3885	WD repeat domain 59
6574	UBE2W	0.045	0.4661	ubiquitin-conjugating enzyme E2W (putative)
6575	STK16	0.045	0.3152	serine/threonine kinase 16
6576	STEAP1B	0.045	0	STEAP family member 1B
6577	RFX5	0.045	0.4812	regulatory factor X, 5 (influences HLA class II expression)
6578	PPP1R14C	0.045	0.2058	protein phosphatase 1, regulatory (inhibitor) subunit 14C
6579	PLA2G3	0.045	0.3771	phospholipase A2, group III
6580	PDDC1	0.045	0.3525	Parkinson disease 7 domain containing 1
6581	NPPA	0.045	0.395	natriuretic peptide A
6582	LRCH2	0.045	0.3722	leucine-rich repeats and calponin homology (CH) domain containing 2
6583	H19	0.045	0.2356	H19, imprinted maternally expressed transcript (non-protein coding)
6584	SLC5A4	0.0442	0.3732	solute carrier family 5 (low affinity glucose cotransporter), member 4
6585	PAH	0.0442	0.2213	phenylalanine hydroxylase
6586	MMP8	0.0442	0.2857	matrix metallopeptidase 8 (neutrophil collagenase)
6587	HNRNPUL2	0.0442	0.3597	heterogeneous nuclear ribonucleoprotein U-like 2
6588	XXYLT1	0.0437	0.2881	xyloside xylosyltransferase 1
6589	TADA2B	0.0437	0.43	transcriptional adaptor 2B
6590	SYCE3	0.0437	0.3432	synaptonemal complex central element protein 3
6591	MIDN	0.0437	0.33	midnolin
6592	CEP44	0.0437	0.488	centrosomal protein 44kDa
6593	SEC61B	0.0433	0.4179	Sec61 beta subunit
6594	SCGB1C1	0.0433	0.2249	secretoglobin, family 1C, member 1
6595	RIC8B	0.0433	0.4487	resistance to inhibitors of cholinesterase 8 homolog B (C. elegans)
6596	IDS	0.0433	0.4295	iduronate 2-sulfatase
6597	HSD11B1	0.0433	0.2979	hydroxysteroid (11-beta) dehydrogenase 1
6598	HADHA	0.0433	0.4678	hydroxyacyl-CoA dehydrogenase/3-ketoacyl-CoA thiolase/enoyl-CoA hydratase (trifunctional protein), alpha subunit
6599	FBXL8	0.0433	0.3413	F-box and leucine-rich repeat protein 8
6600	DHRS9	0.0433	0.3019	dehydrogenase/reductase (SDR family) member 9
6601	CD80	0.0433	0.3092	CD80 molecule
6602	BTG1	0.0433	0.4079	B-cell translocation gene 1, anti-proliferative
6603	YIF1A	0.0425	0.4365	Yip1 interacting factor homolog A (S. cerevisiae)
6604	WDR34	0.0425	0.3725	WD repeat domain 34
6605	TRAF1	0.0425	0.3376	TNF receptor-associated factor 1
6606	SYBU	0.0425	0.2943	syntabulin (syntaxin-interacting)
6607	SLC38A4	0.0425	0.2193	solute carrier family 38, member 4
6608	SLC11A1	0.0425	0.395	solute carrier family 11 (proton-coupled divalent metal ion transporters), member 1
6609	SH2B1	0.0425	0.3889	SH2B adaptor protein 1
6610	PRODH2	0.0425	0.4008	proline dehydrogenase (oxidase) 2
6611	OR2C1	0.0425	0.379	olfactory receptor, family 2, subfamily C, member 1
6612	OR11A1	0.0425	0.3659	olfactory receptor, family 11, subfamily A, member 1
6613	NIPSNAP1	0.0425	0.3856	nipsnap homolog 1 (C. elegans)
6614	NAP1L2	0.0425	0.3998	nucleosome assembly protein 1-like 2
6615	MDK	0.0425	0.3153	midkine (neurite growth-promoting factor 2)
6616	MC2R	0.0425	0.3955	melanocortin 2 receptor (adrenocorticotropic hormone)
6617	MAPKAPK3	0.0425	0.3814	mitogen-activated protein kinase-activated protein kinase 3
6618	LRSAM1	0.0425	0.3527	leucine rich repeat and sterile alpha motif containing 1
6619	COPB2	0.0425	0.4906	coatomer protein complex, subunit beta 2 (beta prime)
6620	CACNG1	0.0425	0.3984	calcium channel, voltage-dependent, gamma subunit 1
6621	KPNA4	0.0422	0.4919	karyopherin alpha 4 (importin alpha 3)
6622	ZFAND5	0.0417	0.4547	zinc finger, AN1-type domain 5
6623	UQCRC2	0.0417	0.4744	ubiquinol-cytochrome c reductase core protein II
6624	UBE2A	0.0417	0.4825	ubiquitin-conjugating enzyme E2A
6625	TNMD	0.0417	0.3453	tenomodulin
6626	PSMC2	0.0417	0.486	proteasome (prosome, macropain) 26S subunit, ATPase, 2
6627	MEPE	0.0417	0.3946	matrix extracellular phosphoglycoprotein
6628	HEATR2	0.0417	0.4203	HEAT repeat containing 2
6629	GYS1	0.0417	0.381	glycogen synthase 1 (muscle)
6630	GIT2	0.0417	0.4567	G protein-coupled receptor kinase interacting ArfGAP 2
6631	CYP1A2	0.0417	0.3775	cytochrome P450, family 1, subfamily A, polypeptide 2
6632	BLVRA	0.0417	0.4314	biliverdin reductase A
6633	MYCBP	0.0414	0.4019	c-myc binding protein
6634	LOC387647	0.0414	0.3023	patched domain containing 3 pseudogene

6635	FAM35A	0.0414	0.4821	family with sequence similarity 35, member A
6636	TMEM232	0.0413	0.262	transmembrane protein 232
6637	HDDC3	0.0413	0.3972	HD domain containing 3
6638	ZXDB	0.0409	0.3382	zinc finger, X-linked, duplicated B
6639	TXLNG	0.0408	0.4205	taxilin gamma
6640	TMPRSS11D	0.0408	0.3362	transmembrane protease, serine 11D
6641	TAB2	0.0408	0.4757	TGF-beta activated kinase 1/MAP3K7 binding protein 2
6642	RPS6KA4	0.0408	0.4101	ribosomal protein S6 kinase, 90kDa, polypeptide 4
6643	PCIF1	0.0408	0.4104	PDX1 C-terminal inhibiting factor 1
6644	OSTF1	0.0408	0.4586	osteoclast stimulating factor 1
6645	OR2J2	0.0408	0.3328	olfactory receptor, family 2, subfamily J, member 2
6646	MARCH7	0.0408	0.4823	membrane-associated ring finger (C3HC4) 7, E3 ubiquitin protein ligase
6647	LMO4	0.0408	0.382	LIM domain only 4
6648	KIR3DL2	0.0408	0.3759	killer cell immunoglobulin-like receptor, three domains, long cytoplasmic tail, 2
6649	HSD3B2	0.0408	0.365	hydroxy-delta-5-steroid dehydrogenase, 3 beta- and steroid delta-isomerase 2
6650	HPN	0.0408	0.3572	hepsin
6651	DCLRE1C	0.0408	0.4684	DNA cross-link repair 1C
6652	AQP7	0.0408	0.3577	aquaporin 7
6653	ALDH1L1	0.0408	0.3165	aldehyde dehydrogenase 1 family, member L1
6654	ADK	0.0408	0.4639	adenosine kinase
6655	TNNI3	0.04	0.3724	troponin I type 3 (cardiac)
6656	RUFY3	0.04	0.4466	RUN and FYVE domain containing 3
6657	PFKM	0.04	0.4511	phosphofructokinase, muscle
6658	NCOA4	0.04	0.4785	nuclear receptor coactivator 4
6659	EXOSC1	0.04	0.4607	exosome component 1
6660	EPC1	0.04	0.4107	enhancer of polycomb homolog 1 (Drosophila)
6661	B9D1	0.04	0.3486	B9 protein domain 1
6662	TOMM20	0.0392	0.4695	translocase of outer mitochondrial membrane 20 homolog (yeast)
6663	RCBTB1	0.0392	0.4865	regulator of chromosome condensation (RCC1) and BTB (POZ) domain containing protein 1
6664	POLR2C	0.0392	0.44	polymerase (RNA) II (DNA directed) polypeptide C, 33kDa
6665	KIDINS220	0.0392	0.4838	kinase D-interacting substrate, 220kDa
6666	HIST1H2BK	0.0392	0.3909	histone cluster 1, H2bk
6667	GRM1	0.0392	0.4012	glutamate receptor, metabotropic 1
6668	GABRQ	0.0392	0.3916	gamma-aminobutyric acid (GABA) A receptor, theta
6669	BNIP2	0.0392	0.4749	BCL2/adenovirus E1B 19kDa interacting protein 2
6670	BACH1	0.0392	0.419	BTB and CNC homology 1, basic leucine zipper transcription factor 1
6671	TBX15	0.0388	0.2567	T-box 15
6672	STOX2	0.0388	0.2694	storkhead box 2
6673	DCAF4	0.0388	0.4315	DDB1 and CUL4 associated factor 4
6674	VEGFB	0.0383	0.3692	vascular endothelial growth factor B
6675	RABAC1	0.0383	0.3855	Rab acceptor 1 (prenylated)
6676	RAB7A	0.0383	0.4839	RAB7A, member RAS oncogene family
6677	RAB33B	0.0383	0.4549	RAB33B, member RAS oncogene family
6678	PDGFA	0.0383	0.2751	platelet-derived growth factor alpha polypeptide
6679	IKZF3	0.0383	0.3	IKAROS family zinc finger 3 (Aiolos)
6680	GSS	0.0383	0.4256	glutathione synthetase
6681	GNAT2	0.0382	0.3408	guanine nucleotide binding protein (G protein), alpha transducing activity polypeptide 2
6682	VCP	0.0375	0.4658	valosin containing protein
6683	SPNS1	0.0375	0.3772	spinster homolog 1 (Drosophila)
6684	SLC37A1	0.0375	0.3194	solute carrier family 37 (glycerol-3-phosphate transporter), member 1
6685	PUS7L	0.0375	0.464	pseudouridylate synthase 7 homolog (S. cerevisiae)-like
6686	PDLIM7	0.0375	0.3958	PDZ and LIM domain 7 (enigma)
6687	NXPH1	0.0375	0.1848	neurexophilin 1
6688	IL28RA	0.0375	0.2468	interleukin 28 receptor, alpha (interferon, lambda receptor)
6689	GLCCI1	0.0375	0.4466	glucocorticoid induced transcript 1
6690	FILIP1	0.0375	0.2764	filamin A interacting protein 1
6691	CYP26B1	0.0375	0.2947	cytochrome P450, family 26, subfamily B, polypeptide 1
6692	CCDC151	0.0375	0.3467	coiled-coil domain containing 151
6693	C8orf59	0.0375	0.4592	chromosome 8 open reading frame 59
6694	ATP10B	0.0375	0.363	ATPase, class V, type 10B
6695	AHCY	0.0375	0.4344	adenosylhomocysteinase
6696	LOC93432	0.0373	0.3686	maltase-glucoamylase (alpha-glucosidase) pseudogene
6697	LOC285419	0.0371	0	uncharacterized LOC285419
6698	LDHA	0.0371	0.4653	lactate dehydrogenase A
6699	STX8	0.0367	0.4495	syntaxin 8
6700	SLC9A2	0.0367	0.3514	solute carrier family 9, subfamily A (NHE2, cation proton antiporter 2), member 2
6701	CUL7	0.0367	0.4078	cullin 7
6702	CMTM6	0.0367	0.4801	CKLF-like MARVEL transmembrane domain containing 6
6703	CASP5	0.0367	0.3404	caspase 5, apoptosis-related cysteine peptidase
6704	UNC5B	0.0362	0.2724	unc-5 homolog B (C. elegans)
6705	PNPLA7	0.0362	0.3805	patatin-like phospholipase domain containing 7
6706	FAM65C	0.0362	0.3066	family with sequence similarity 65, member C
6707	FAM13A-AS1	0.0362	0.3227	FAM13A antisense RNA 1 (non-protein coding)
6708	ANKRD30A	0.0362	0.2488	ankyrin repeat domain 30A
6709	TMEM35	0.0358	0.2769	transmembrane protein 35
6710	SMURF1	0.0358	0.3788	SMAD specific E3 ubiquitin protein ligase 1
6711	MRPS35	0.0358	0.477	mitochondrial ribosomal protein S35
6712	MED1	0.0358	0.4795	mediator complex subunit 1
6713	LIPA	0.0358	0.4714	lipase A, lysosomal acid, cholesterol esterase
6714	KCNK1	0.0358	0.276	potassium channel, subfamily K, member 1
6715	GPR97	0.0358	0.3787	G protein-coupled receptor 97
6716	FLII	0.0358	0.4372	flightless I homolog (Drosophila)
6717	DDX52	0.0358	0.48	DEAD (Asp-Glu-Ala-Asp) box polypeptide 52
6718	BSG	0.0358	0.406	basigin (Ok blood group)

6719	ZNF470	0.0357	0.3883	zinc finger protein 470
6720	LOC100131564	0.0357	0.3084	uncharacterized LOC100131564
6721	LOC80054	0.0355	0.4173	uncharacterized LOC80054
6722	TRIT1	0.035	0.4755	tRNA isopentenyltransferase 1
6723	TMEM132B	0.035	0.1954	transmembrane protein 132B
6724	TBL1X	0.035	0.3731	transducin (beta)-like 1X-linked
6725	TACR2	0.035	0.4002	tachykinin receptor 2
6726	KCTD7	0.035	0.3067	potassium channel tetramerisation domain containing 7
6727	GOLGA5	0.035	0.4964	golgin A5
6728	CLUAP1	0.035	0.4773	clusterin associated protein 1
6729	ATP6V0D2	0.035	0.3011	ATPase, H+ transporting, lysosomal 38kDa, V0 subunit d2
6730	ATG10	0.035	0.4876	autophagy related 10
6731	SLC35E2	0.0342	0.3371	solute carrier family 35, member E2
6732	SATB1	0.0342	0.386	SATB homeobox 1
6733	PTN	0.0342	0.306	pleiotrophin
6734	PEPD	0.0342	0.4364	peptidase D
6735	OTUD4	0.0342	0.4438	OTU domain containing 4
6736	NCALD	0.0342	0.2945	neurocalcin delta
6737	MMP3	0.0342	0.2519	matrix metallopeptidase 3 (stromelysin 1, progelatinase)
6738	MGC3771	0.0342	0.367	uncharacterized LOC81854
6739	GHITM	0.0342	0.4744	growth hormone inducible transmembrane protein
6740	CRHBP	0.0342	0.2984	corticotropin releasing hormone binding protein
6741	COL5A3	0.0342	0.3941	collagen, type V, alpha 3
6742	CFHR5	0.0342	0.3257	complement factor H-related 5
6743	CAPZB	0.0342	0.4588	capping protein (actin filament) muscle Z-line, beta
6744	STARD3NL	0.0338	0.4947	STARD3 N-terminal like
6745	SETMAR	0.0333	0.4349	SET domain and mariner transposase fusion gene
6746	GPR132	0.0333	0.392	G protein-coupled receptor 132
6747	FRMD4A	0.0333	0.2735	FERM domain containing 4A
6748	FAM110D	0.0333	0.395	family with sequence similarity 110, member D
6749	CPPED1	0.0333	0.4529	calcineurin-like phosphoesterase domain containing 1
6750	ANKRD1	0.0333	0.3217	ankyrin repeat domain 1 (cardiac muscle)
6751	ZNF540	0.0325	0.3416	zinc finger protein 540
6752	TFF2	0.0325	0.3851	trefoil factor 2
6753	SLC16A6	0.0325	0.3	solute carrier family 16, member 6 (monocarboxylic acid transporter 7)
6754	NTS	0.0325	0.2454	neurotensin
6755	MKX	0.0325	0.2226	mohawk homeobox
6756	LRCH3	0.0325	0.4303	leucine-rich repeats and calponin homology (CH) domain containing 3
6757	INTS10	0.0325	0.501	integrator complex subunit 10
6758	C15orf38	0.0325	0.3761	chromosome 15 open reading frame 38
6759	ANKRD37	0.0325	0.3152	ankyrin repeat domain 37
6760	TTBK2	0.0317	0.3743	tau tubulin kinase 2
6761	SURF1	0.0317	0.3918	surfeit 1
6762	SLC27A6	0.0317	0.2215	solute carrier family 27 (fatty acid transporter), member 6
6763	POU4F2	0.0317	0.2891	POU class 4 homeobox 2
6764	MYBPC3	0.0317	0.4049	myosin binding protein C, cardiac
6765	IFT74	0.0317	0.4799	intraflagellar transport 74 homolog (Chlamydomonas)
6766	GTF2H1	0.0317	0.4891	general transcription factor IIH, polypeptide 1, 62kDa
6767	F10	0.0317	0.3926	coagulation factor X
6768	CASR	0.0317	0.4115	calcium-sensing receptor
6769	NMUR2	0.0313	0.347	neuromedin U receptor 2
6770	CCDC71L	0.0313	0.3072	coiled-coil domain containing 71-like
6771	C12orf59	0.0313	0.1744	chromosome 12 open reading frame 59
6772	BTF3P11	0.0309	0.3794	basic transcription factor 3 pseudogene 11
6773	TAF9B	0.0308	0.4863	TAF9B RNA polymerase II, TATA box binding protein (TBP)-associated factor, 31kDa
6774	SERPINF1	0.0308	0.3339	serpin peptidase inhibitor, clade F (alpha-2 antiplasmin, pigment epithelium derived factor), member 1
6775	SERPINB8	0.0308	0.3758	serpin peptidase inhibitor, clade B (ovalbumin), member 8
6776	FBXO40	0.0308	0.3917	F-box protein 40
6777	EDEM1	0.0308	0.4586	ER degradation enhancer, mannosidase alpha-like 1
6778	BBS10	0.0308	0.4946	Bardet-Biedl syndrome 10
6779	ZNF236	0.03	0.4016	zinc finger protein 236
6780	ZBTB2	0.03	0.489	zinc finger and BTB domain containing 2
6781	SDF2L1	0.03	0.3884	stromal cell-derived factor 2-like 1
6782	RAB11B	0.03	0.4139	RAB11B, member RAS oncogene family
6783	PRMT6	0.03	0.4935	protein arginine methyltransferase 6
6784	PIP4K2C	0.03	0.4159	phosphatidylinositol-5-phosphate 4-kinase, type II, gamma
6785	OR6A2	0.03	0.3151	olfactory receptor, family 6, subfamily A, member 2
6786	NAPEPLD	0.03	0.4949	N-acyl phosphatidylethanolamine phospholipase D
6787	MFAP3L	0.03	0.2905	microfibrillar-associated protein 3-like
6788	LOC400940	0.03	0.2219	uncharacterized LOC400940
6789	KCNG1	0.03	0.3367	potassium voltage-gated channel, subfamily G, member 1
6790	KBTBD6	0.03	0.4752	kelch repeat and BTB (POZ) domain containing 6
6791	FHAD1	0.03	0.3432	forkhead-associated (FHA) phosphopeptide binding domain 1
6792	CUZD1	0.03	0.3162	CUB and zona pellucida-like domains 1
6793	PPP1R11	0.0292	0.4186	protein phosphatase 1, regulatory (inhibitor) subunit 11
6794	NTF3	0.0292	0.3946	neurotrophin 3
6795	CSPG4	0.0292	0.364	chondroitin sulfate proteoglycan 4
6796	C8orf44	0.0292	0.3525	chromosome 8 open reading frame 44
6797	TTC14	0.0288	0.4719	tetratricopeptide repeat domain 14
6798	PPAPDC2	0.0288	0.4866	phosphatidic acid phosphatase type 2 domain containing 2
6799	MFSD4	0.0288	0.2315	major facilitator superfamily domain containing 4
6800	LRRC58	0.0288	0.4847	leucine rich repeat containing 58
6801	KIAA1432	0.0288	0.4778	KIAA1432
6802	HVCN1	0.0288	0.3193	hydrogen voltage-gated channel 1

6803	FBXL20	0.0288	0.4591	F-box and leucine-rich repeat protein 20
6804	ADAMTS10	0.0288	0.3923	ADAM metallopeptidase with thrombospondin type 1 motif, 10
6805	PAIP1	0.0286	0.3364	poly(A) binding protein interacting protein 1
6806	DMRTA2	0.0286	0.3406	DMRT-like family A2
6807	APOC4	0.0286	0.4009	apolipoprotein C-IV
6808	RNF146	0.0283	0.4791	ring finger protein 146
6809	HCLS1	0.0283	0.3539	hematopoietic cell-specific Lyn substrate 1
6810	ETAA1	0.0283	0.4881	Ewing tumor-associated antigen 1
6811	DPY19L1	0.0283	0.494	dpy-19-like 1 (C. elegans)
6812	DNMT3L	0.0283	0.3813	DNA (cytosine-5-)-methyltransferase 3-like
6813	ZFYVE28	0.0275	0.3793	zinc finger, FYVE domain containing 28
6814	ZFYVE1	0.0275	0.4089	zinc finger, FYVE domain containing 1
6815	WARS	0.0275	0.4362	tryptophanyl-tRNA synthetase
6816	SMYD3	0.0275	0.4701	SET and MYND domain containing 3
6817	SMCR8	0.0275	0.3271	Smith-Magenis syndrome chromosome region, candidate 8
6818	ORMDL1	0.0275	0.4964	ORM1-like 1 (S. cerevisiae)
6819	NDUFA10	0.0275	0.4616	NADH dehydrogenase (ubiquinone) 1 alpha subcomplex, 10, 42kDa
6820	MZF1	0.0275	0.3703	myeloid zinc finger 1
6821	KRT73	0.0275	0.3369	keratin 73
6822	JSRP1	0.0275	0.3504	junctional sarcoplasmic reticulum protein 1
6823	IRF3	0.0275	0.3862	interferon regulatory factor 3
6824	G6PC	0.0275	0.3238	glucose-6-phosphatase, catalytic subunit
6825	FLJ10038	0.0275	0.3821	uncharacterized protein FLJ10038
6826	CRNDE	0.0275	0.4323	colorectal neoplasia differentially expressed (non-protein coding)
6827	CNRIP1	0.0275	0.3154	cannabinoid receptor interacting protein 1
6828	AACS	0.0275	0.4407	acetoacetyl-CoA synthetase
6829	TMX1	0.0267	0.483	thioredoxin-related transmembrane protein 1
6830	SZT2	0.0267	0.3724	seizure threshold 2 homolog (mouse)
6831	SLC7A11	0.0267	0.3842	solute carrier family 7 (anionic amino acid transporter light chain, xc- system), member 11
6832	SKIV2L2	0.0267	0.4898	superkiller viralicidic activity 2-like 2 (S. cerevisiae)
6833	RNF10	0.0267	0.4256	ring finger protein 10
6834	PIR	0.0267	0.4107	pirin (iron-binding nuclear protein)
6835	FGF9	0.0267	0.2762	fibroblast growth factor 9 (glia-activating factor)
6836	ASCL3	0.0267	0.3859	achaete-scute complex homolog 3 (Drosophila)
6837	ZFYVE19	0.0262	0.3589	zinc finger, FYVE domain containing 19
6838	RPS19BP1	0.0262	0.4164	ribosomal protein S19 binding protein 1
6839	MOGAT1	0.0262	0.2902	monoacylglycerol O-acyltransferase 1
6840	ELMOD3	0.0262	0.371	ELMO/CED-12 domain containing 3
6841	ZNF556	0.0258	0.3666	zinc finger protein 556
6842	SCAMP4	0.0258	0.3937	secretory carrier membrane protein 4
6843	PPYR1	0.0258	0.3879	pancreatic polypeptide receptor 1
6844	PHOX2B	0.0258	0.3602	paired-like homeobox 2b
6845	LRRFIP2	0.0258	0.4434	leucine rich repeat (in FLII) interacting protein 2
6846	IMPAD1	0.0258	0.4834	inositol monophosphatase domain containing 1
6847	HMGN4	0.0258	0.4853	high mobility group nucleosomal binding domain 4
6848	GP2	0.0258	0.4066	glycoprotein 2 (zymogen granule membrane)
6849	CACFD1	0.0258	0.4342	calcium channel flower domain containing 1
6850	ASB9	0.0258	0.2475	ankyrin repeat and SOCS box containing 9
6851	ZNF624	0.025	0.4784	zinc finger protein 624
6852	TRIM55	0.025	0.2671	tripartite motif containing 55
6853	RNF115	0.025	0.4946	ring finger protein 115
6854	RMND1	0.025	0.4947	required for meiotic nuclear division 1 homolog (S. cerevisiae)
6855	PROSC	0.025	0.4947	proline synthetase co-transcribed homolog (bacterial)
6856	PDCD6	0.025	0.4787	programmed cell death 6
6857	NKX3-1	0.025	0.4011	NK3 homeobox 1
6858	GNAS	0.025	0.4395	GNAS complex locus
6859	ETV7	0.025	0.3872	ets variant 7
6860	CYLD	0.025	0.4516	cylindromatosis (turban tumor syndrome)
6861	AKAP12	0.025	0.3854	A kinase (PRKA) anchor protein 12
6862	A1BG-AS1	0.025	0.3746	A1BG antisense RNA 1 (non-protein coding)
6863	ZBP1	0.0242	0.3791	Z-DNA binding protein 1
6864	USPL1	0.0242	0.4735	ubiquitin specific peptidase like 1
6865	POU2AF1	0.0242	0.2392	POU class 2 associating factor 1
6866	PARP12	0.0242	0.416	poly (ADP-ribose) polymerase family, member 12
6867	C8A	0.0242	0.389	complement component 8, alpha polypeptide
6868	C14orf159	0.0242	0.4052	chromosome 14 open reading frame 159
6869	ALDOA	0.0242	0.4079	aldolase A, fructose-bisphosphate
6870	TMEM44	0.0237	0.3013	transmembrane protein 44
6871	TMEM198B	0.0237	0.3881	transmembrane protein 198B, pseudogene
6872	PEBP4	0.0237	0.3614	phosphatidylethanolamine-binding protein 4
6873	SMARCD3	0.0233	0.3603	SWI/SNF related, matrix associated, actin dependent regulator of chromatin, subfamily d, member 3
6874	RHBG	0.0233	0.4022	Rh family, B glycoprotein (gene/pseudogene)
6875	RAB14	0.0233	0.5019	RAB14, member RAS oncogene family
6876	OR51E2	0.0233	0.3788	olfactory receptor, family 51, subfamily E, member 2
6877	BPESC1	0.0233	0.2694	blepharophimosis, epicanthus inversus and ptosis, candidate 1 (non-protein coding)
6878	APOM	0.0233	0.3536	apolipoprotein M
6879	TRAF4	0.0225	0.3532	TNF receptor-associated factor 4
6880	TM7SF2	0.0225	0.3739	transmembrane 7 superfamily member 2
6881	PHKA2	0.0225	0.3793	phosphorylase kinase, alpha 2 (liver)
6882	PACS1	0.0225	0.4247	phosphofurin acidic cluster sorting protein 1
6883	MRPL53	0.0225	0.4341	mitochondrial ribosomal protein L53
6884	LRFN5	0.0225	0.2949	leucine rich repeat and fibronectin type III domain containing 5
6885	HECW2	0.0225	0.3466	HECT, C2 and WW domain containing E3 ubiquitin protein ligase 2
6886	GMEB2	0.0225	0.3383	glucocorticoid modulatory element binding protein 2

6887	GIF	0.0225	0.3378	gastric intrinsic factor (vitamin B synthesis)
6888	COX14	0.0225	0.4337	COX14 cytochrome c oxidase assembly homolog (S. cerevisiae)
6889	OR7C1	0.0218	0.3878	olfactory receptor, family 7, subfamily C, member 1
6890	TIMP2	0.0217	0.4036	TIMP metallopeptidase inhibitor 2
6891	OCEL1	0.0217	0.3874	occludin/ELL domain containing 1
6892	LIPC	0.0217	0.3095	lipase, hepatic
6893	IFNA6	0.0217	0.3602	interferon, alpha 6
6894	GABRA3	0.0217	0.3871	gamma-aminobutyric acid (GABA) A receptor, alpha 3
6895	C6orf48	0.0217	0.4128	chromosome 6 open reading frame 48
6896	TMEM132E	0.0214	0.3857	transmembrane protein 132E
6897	LPP-AS2	0.0214	0	LPP antisense RNA 2 (non-protein coding)
6898	UBQLNL	0.0213	0.3219	ubiquilin-like
6899	RASD1	0.0213	0.3212	RAS, dexamethasone-induced 1
6900	GLDN	0.0213	0.2847	gliomedin
6901	TSFM	0.0208	0.4354	Ts translation elongation factor, mitochondrial
6902	SNCA	0.0208	0.287	synuclein, alpha (non A4 component of amyloid precursor)
6903	PRKCE	0.0208	0.3884	protein kinase C, epsilon
6904	PGAP1	0.0208	0.471	post-GPI attachment to proteins 1
6905	LYPLA2	0.0208	0.3946	lysophospholipase II
6906	LAPTM4B	0.0208	0.4653	lysosomal protein transmembrane 4 beta
6907	HOXD3	0.0208	0.3939	homeobox D3
6908	GPR35	0.0208	0.4052	G protein-coupled receptor 35
6909	DEFA6	0.0208	0.3776	defensin, alpha 6, Paneth cell-specific
6910	ARPC4	0.0208	0.4279	actin related protein 2/3 complex, subunit 4, 20kDa
6911	WDR92	0.02	0.4951	WD repeat domain 92
6912	TXNDC5	0.02	0.402	thioredoxin domain containing 5 (endoplasmic reticulum)
6913	TSR2	0.02	0.4238	TSR2, 20S rRNA accumulation, homolog (S. cerevisiae)
6914	TSPYL1	0.02	0.4854	TSPY-like 1
6915	TPRXL	0.02	0.3483	tetra-peptide repeat homeobox-like
6916	SLC46A1	0.02	0.3775	solute carrier family 46 (folate transporter), member 1
6917	SLC17A4	0.02	0.3828	solute carrier family 17 (sodium phosphate), member 4
6918	SEPHS1	0.02	0.4759	selenophosphate synthetase 1
6919	PXMP2	0.02	0.3973	peroxisomal membrane protein 2, 22kDa
6920	GNB2L1	0.02	0.4663	guanine nucleotide binding protein (G protein), beta polypeptide 2-like 1
6921	FUT10	0.02	0.3755	fucosyltransferase 10 (alpha (1,3) fucosyltransferase)
6922	FAM208A	0.02	0.4951	family with sequence similarity 208, member A
6923	ARHGEF26	0.02	0.3375	Rho guanine nucleotide exchange factor (GEF) 26
6924	PPP1R10	0.0192	0.4019	protein phosphatase 1, regulatory subunit 10
6925	OR12D2	0.0192	0.386	olfactory receptor, family 12, subfamily D, member 2
6926	MSMB	0.0192	0.2687	microseminoprotein, beta-
6927	MS4A1	0.0192	0.3159	membrane-spanning 4-domains, subfamily A, member 1
6928	LRRC6	0.0192	0.3008	leucine rich repeat containing 6
6929	FBXO11	0.0192	0.4851	F-box protein 11
6930	APOBEC2	0.0192	0.3978	apolipoprotein B mRNA editing enzyme, catalytic polypeptide-like 2
6931	PCDHB10	0.0188	0.3785	protocadherin beta 10
6932	CFL2	0.0188	0.4805	cofilin 2 (muscle)
6933	ZNF239	0.0183	0.378	zinc finger protein 239
6934	SRSF5	0.0183	0.4694	serine/arginine-rich splicing factor 5
6935	PLIN3	0.0183	0.4343	perilipin 3
6936	OASL	0.0183	0.3464	2'-5'-oligoadenylate synthetase-like
6937	MMP26	0.0183	0.3871	matrix metallopeptidase 26
6938	MAPK14	0.0183	0.4791	mitogen-activated protein kinase 14
6939	LBP	0.0183	0.392	lipopolysaccharide binding protein
6940	HSPA6	0.0183	0.35	heat shock 70kDa protein 6 (HSP70B')
6941	HPSE	0.0183	0.3537	heparanase
6942	DUSP11	0.0183	0.4941	dual specificity phosphatase 11 (RNA/RNP complex 1-interacting)
6943	CALB2	0.0183	0.3926	calbindin 2
6944	TRPM6	0.0175	0.3967	transient receptor potential cation channel, subfamily M, member 6
6945	STS	0.0175	0.3789	steroid sulfatase (microsomal), isozyme S
6946	RBM20	0.0175	0.222	RNA binding motif protein 20
6947	PIK3CA	0.0175	0.4856	phosphatidylinositol-4,5-bisphosphate 3-kinase, catalytic subunit alpha
6948	MS4A6A	0.0175	0.3369	membrane-spanning 4-domains, subfamily A, member 6A
6949	METTL4	0.0175	0.4932	methyltransferase like 4
6950	MED17	0.0175	0.5038	mediator complex subunit 17
6951	JAK2	0.0175	0.478	Janus kinase 2
6952	GPR143	0.0175	0.3733	G protein-coupled receptor 143
6953	GLIS3-AS1	0.0175	0.3649	GLIS3 antisense RNA 1 (non-protein coding)
6954	FLJ20021	0.0175	0.3642	uncharacterized LOC90024
6955	ARMC2	0.0175	0.3023	armadillo repeat containing 2
6956	LOC100130502	0.0171	0	uncharacterized LOC100130502
6957	ZCCHC8	0.0167	0.4896	zinc finger, CCHC domain containing 8
6958	TMEM147	0.0167	0.447	transmembrane protein 147
6959	PCNXL4	0.0167	0.5031	pecanex-like 4 (Drosophila)
6960	IFIT5	0.0167	0.4694	interferon-induced protein with tetratricopeptide repeats 5
6961	CCNJL	0.0167	0.3742	cyclin J-like
6962	TMCC3	0.0162	0.3213	transmembrane and coiled-coil domain family 3
6963	TAF1D	0.0162	0.4592	TATA box binding protein (TBP)-associated factor, RNA polymerase I, D, 41kDa
6964	IRAK1BP1	0.0162	0.4771	interleukin-1 receptor-associated kinase 1 binding protein 1
6965	D2HGDH	0.0162	0.3338	D-2-hydroxyglutarate dehydrogenase
6966	CCNY	0.0162	0.5015	cyclin Y
6967	ADHFE1	0.0162	0.3811	alcohol dehydrogenase, iron containing, 1
6968	ABHD16A	0.0162	0.4435	abhydrolase domain containing 16A
6969	ZBTB16	0.0158	0.3279	zinc finger and BTB domain containing 16
6970	TNRC6B	0.0158	0.4392	trinucleotide repeat containing 6B

6971	SLC25A10	0.0158	0.4079	solute carrier family 25 (mitochondrial carrier; dicarboxylate transporter), member 10
6972	PGM3	0.0158	0.4861	phosphoglucomutase 3
6973	OCLM	0.0158	0.3668	oculomedin
6974	NFRKB	0.0158	0.3902	nuclear factor related to kappaB binding protein
6975	MMP12	0.0158	0.291	matrix metallopeptidase 12 (macrophage elastase)
6976	KIR2DS4	0.0158	0.3888	killer cell immunoglobulin-like receptor, two domains, short cytoplasmic tail, 4
6977	CNOT8	0.0158	0.4937	CCR4-NOT transcription complex, subunit 8
6978	ZNF677	0.0157	0.3113	zinc finger protein 677
6979	KRBA2	0.0157	0.3301	KRAB-A domain containing 2
6980	ZNF783	0.0155	0.3579	zinc finger family member 783
6981	ZNF300	0.015	0.4266	zinc finger protein 300
6982	ZNF273	0.015	0.4603	zinc finger protein 273
6983	SLC6A4	0.015	0.396	solute carrier family 6 (neurotransmitter transporter, serotonin), member 4
6984	RPS6KA1	0.015	0.3879	ribosomal protein S6 kinase, 90kDa, polypeptide 1
6985	ME1	0.015	0.4502	malic enzyme 1, NADP(+)-dependent, cytosolic
6986	GP9	0.015	0.4019	glycoprotein IX (platelet)
6987	EBAG9	0.015	0.4786	estrogen receptor binding site associated, antigen, 9
6988	C7orf60	0.015	0.4869	chromosome 7 open reading frame 60
6989	BTNL2	0.015	0.3905	butyrophilin-like 2 (MHC class II associated)
6990	APOBEC3F	0.015	0.3308	apolipoprotein B mRNA editing enzyme, catalytic polypeptide-like 3F
6991	LINC00314	0.0143	0.2574	long intergenic non-protein coding RNA 314
6992	BTN2A3P	0.0143	0.3979	butyrophilin, subfamily 2, member A3, pseudogene
6993	TCEB3B	0.0142	0.3938	transcription elongation factor B polypeptide 3B (elongin A2)
6994	TBX21	0.0142	0.3728	T-box 21
6995	PROX1	0.0142	0.344	prospero homeobox 1
6996	POM121L9P	0.0142	0.3609	POM121 transmembrane nucleoporin-like 9, pseudogene
6997	PIK3C3	0.0142	0.4992	phosphatidylinositol 3-kinase, catalytic subunit type 3
6998	GUCA2B	0.0142	0.4025	guanylate cyclase activator 2B (uroguanylin)
6999	FBXO17	0.0142	0.3832	F-box protein 17
7000	FASTK	0.0142	0.4047	Fas-activated serine/threonine kinase
7001	FAM76A	0.0142	0.4188	family with sequence similarity 76, member A
7002	AIM1L	0.0142	0.381	absent in melanoma 1-like
7003	TOP1MT	0.0137	0.4009	topoisomerase (DNA) I, mitochondrial
7004	TBC1D23	0.0137	0.4974	TBC1 domain family, member 23
7005	FAM227B	0.0137	0.2725	family with sequence similarity 227, member B
7006	C4orf33	0.0137	0.4895	chromosome 4 open reading frame 33
7007	ABHD1	0.0137	0.3525	abhydrolase domain containing 1
7008	WDR61	0.0133	0.4851	WD repeat domain 61
7009	SPAG4	0.0133	0.3048	sperm associated antigen 4
7010	PPBPP2	0.0133	0.3897	pro-platelet basic protein pseudogene 2
7011	O3FAR1	0.0133	0	omega-3 fatty acid receptor 1
7012	NSDHL	0.0133	0.4678	NAD(P) dependent steroid dehydrogenase-like
7013	NDUFC1	0.0133	0.4608	NADH dehydrogenase (ubiquinone) 1, subcomplex unknown, 1, 6kDa
7014	LAT2	0.0133	0.3052	linker for activation of T cells family, member 2
7015	CHL1-AS2	0.0129	0.0004	CHL1 antisense RNA 2 (non-protein coding)
7016	ZNF695	0.0125	0.3474	zinc finger protein 695
7017	SHROOM4	0.0125	0.3813	shroom family member 4
7018	RP11-165H20.1	0.0125	0.2875	CHIA-like pseudogene
7019	RNF121	0.0125	0.3889	ring finger protein 121
7020	RAP2C	0.0125	0.4643	RAP2C, member of RAS oncogene family
7021	NTRK3	0.0125	0.4274	neurotrophic tyrosine kinase, receptor, type 3
7022	NPFFR1	0.0125	0.4056	neuropeptide FF receptor 1
7023	LY6K	0.0125	0.3293	lymphocyte antigen 6 complex, locus K
7024	KIAA1644	0.0125	0.4158	KIAA1644
7025	KIAA0240	0.0125	0.4744	KIAA0240
7026	HLA-DQB1	0.0125	0.3352	major histocompatibility complex, class II, DQ beta 1
7027	HIVEP2	0.0125	0.4466	human immunodeficiency virus type I enhancer binding protein 2
7028	ELF2	0.0125	0.4724	E74-like factor 2 (ets domain transcription factor)
7029	DPYSL2	0.0125	0.4839	dihydropyrimidinase-like 2
7030	CX3CR1	0.0125	0.3411	chemokine (C-X3-C motif) receptor 1
7031	BCAS4	0.0125	0.3762	breast carcinoma amplified sequence 4
7032	ST8SIA4	0.0117	0.4047	ST8 alpha-N-acetyl-neuraminide alpha-2,8-sialyltransferase 4
7033	RPL3	0.0117	0.4563	ribosomal protein L3
7034	NKX3-2	0.0117	0.3753	NK3 homeobox 2
7035	MYH7	0.0117	0.305	myosin, heavy chain 7, cardiac muscle, beta
7036	KCNB1	0.0117	0.3849	potassium voltage-gated channel, Shab-related subfamily, member 1
7037	INSR	0.0117	0.3642	insulin receptor
7038	H2AFY2	0.0117	0.3469	H2A histone family, member Y2
7039	GSR	0.0117	0.459	glutathione reductase
7040	CRP	0.0117	0.3956	C-reactive protein, pentraxin-related
7041	GJB4	0.0114	0.3569	gap junction protein, beta 4, 30.3kDa
7042	FLG2	0.0114	0.2802	filaggrin family member 2
7043	ZDHHC8	0.0113	0.3547	zinc finger, DHHC-type containing 8
7044	SYTL1	0.0113	0.3201	synaptotagmin-like 1
7045	RUFY2	0.0113	0.4485	RUN and FYVE domain containing 2
7046	RILP	0.0113	0.4245	Rab interacting lysosomal protein
7047	CHCHD5	0.0113	0.421	coiled-coil-helix-coiled-coil-helix domain containing 5
7048	ZDHHC4	0.0108	0.4648	zinc finger, DHHC-type containing 4
7049	USP3	0.0108	0.4785	ubiquitin specific peptidase 3
7050	TNR	0.0108	0.4155	tenascin R
7051	SLC17A2	0.0108	0.3844	solute carrier family 17 (sodium phosphate), member 2
7052	RPGRIP1L	0.0108	0.4943	RPGRIP1-like
7053	ZNF667	0.01	0.3106	zinc finger protein 667
7054	ZNF611	0.01	0.4738	zinc finger protein 611

7055	ZNF610	0.01	0.4168	zinc finger protein 610
7056	ZNF260	0.01	0.5127	zinc finger protein 260
7057	ZBTB3	0.01	0.3683	zinc finger and BTB domain containing 3
7058	TGDS	0.01	0.4839	TDP-glucose 4,6-dehydratase
7059	SPATA5	0.01	0.483	spermatogenesis associated 5
7060	SLC9A9	0.01	0.3821	solute carrier family 9, subfamily A (NHE9, cation proton antiporter 9), member 9
7061	RTKN	0.01	0.3684	rhotekin
7062	RAG1	0.01	0.2699	recombination activating gene 1
7063	OR4D1	0.01	0.3635	olfactory receptor, family 4, subfamily D, member 1
7064	LYNX1	0.01	0.3952	Ly6/neurotoxin 1
7065	LOC400236	0.01	0.3587	uncharacterized LOC400236
7066	LOC286367	0.01	0	FP944
7067	KCNA4	0.01	0.383	potassium voltage-gated channel, shaker-related subfamily, member 4
7068	IL11	0.01	0.4051	interleukin 11
7069	DNAH11	0.01	0.3316	dynein, axonemal, heavy chain 11
7070	ADRA2C	0.01	0.4125	adrenoceptor alpha 2C
7071	AASDH	0.01	0.5045	aminoadipate-semialdehyde dehydrogenase
7072	ZNF410	0.0092	0.5028	zinc finger protein 410
7073	PSME4	0.0092	0.4856	proteasome (prosome, macropain) activator subunit 4
7074	IFNG	0.0092	0.3228	interferon, gamma
7075	FOXD2	0.0092	0.3969	forkhead box D2
7076	EIF2C4	0.0092	0.4541	eukaryotic translation initiation factor 2C, 4
7077	CNIH	0.0092	0.4714	cornichon homolog (Drosophila)
7078	CD2AP	0.0092	0.4846	CD2-associated protein
7079	B4GALNT1	0.0092	0.4103	beta-1,4-N-acetyl-galactosaminyl transferase 1
7080	AP1G1	0.0092	0.5024	adaptor-related protein complex 1, gamma 1 subunit
7081	ANKRD28	0.0092	0.492	ankyrin repeat domain 28
7082	TCEAL7	0.0088	0.312	transcription elongation factor A (SII)-like 7
7083	SPTSSB	0.0088	0.3272	serine palmitoyltransferase, small subunit B
7084	PLEKHN1	0.0088	0.3907	pleckstrin homology domain containing, family N member 1
7085	C8orf34	0.0088	0.2536	chromosome 8 open reading frame 34
7086	LOC148189	0.0086	0.4514	uncharacterized LOC148189
7087	ZNF277	0.0083	0.486	zinc finger protein 277
7088	ZC3H13	0.0083	0.4117	zinc finger CCCH-type containing 13
7089	NMRK2	0.0083	0.3946	nicotinamide riboside kinase 2
7090	INPP5A	0.0083	0.4731	inositol polyphosphate-5-phosphatase, 40kDa
7091	GRIA1	0.0083	0.4007	glutamate receptor, ionotropic, AMPA 1
7092	GPR107	0.0083	0.4971	G protein-coupled receptor 107
7093	FAM49A	0.0083	0.3883	family with sequence similarity 49, member A
7094	DLAT	0.0083	0.4798	dihydrolipoamide S-acetyltransferase
7095	CD163	0.0083	0.3252	CD163 molecule
7096	BIN1	0.0083	0.3696	bridging integrator 1
7097	ACPP	0.0083	0.3288	acid phosphatase, prostate
7098	TGFBRAP1	0.0075	0.4294	transforming growth factor, beta receptor associated protein 1
7099	SLC45A3	0.0075	0.3679	solute carrier family 45, member 3
7100	SLC25A39	0.0075	0.4286	solute carrier family 25, member 39
7101	PPP1R36	0.0075	0.3095	protein phosphatase 1, regulatory subunit 36
7102	PHF2	0.0075	0.4608	PHD finger protein 2
7103	PDZD8	0.0075	0.4803	PDZ domain containing 8
7104	NAV1	0.0075	0.3687	neuron navigator 1
7105	LOC654433	0.0075	0.3066	uncharacterized LOC654433
7106	LOC389332	0.0075	0.399	uncharacterized LOC389332
7107	KLHL21	0.0075	0.4014	kelch-like 21 (Drosophila)
7108	KCNK7	0.0075	0.3993	potassium channel, subfamily K, member 7
7109	KCNJ6	0.0075	0.3967	potassium inwardly-rectifying channel, subfamily J, member 6
7110	HIST1H2BD	0.0075	0.3728	histone cluster 1, H2bd
7111	GAL3ST3	0.0075	0.3597	galactose-3-O-sulfotransferase 3
7112	CNTNAP1	0.0075	0.4197	contactin associated protein 1
7113	B4GALT7	0.0075	0.4069	xylosylprotein beta 1,4-galactosyltransferase, polypeptide 7
7114	AGXT2	0.0075	0.3522	alanine--glyoxylate aminotransferase 2
7115	ZNF83	0.0067	0.4848	zinc finger protein 83
7116	WNT16	0.0067	0.3239	wingless-type MMTV integration site family, member 16
7117	WEE1	0.0067	0.4652	WEE1 homolog (S. pombe)
7118	TRIM23	0.0067	0.4717	tripartite motif containing 23
7119	TPRA1	0.0067	0.3872	transmembrane protein, adipocyte asscociated 1
7120	THUMPD1	0.0067	0.4998	THUMP domain containing 1
7121	PRDX4	0.0067	0.4696	peroxiredoxin 4
7122	PLLP	0.0067	0.3685	plasmolipin
7123	MPL	0.0067	0.4028	myeloproliferative leukemia virus oncogene
7124	LINC00474	0.0067	0.3741	long intergenic non-protein coding RNA 474
7125	KLHDC2	0.0067	0.4913	kelch domain containing 2
7126	AIPL1	0.0067	0.3964	aryl hydrocarbon receptor interacting protein-like 1
7127	TMEM203	0.0063	0.4992	transmembrane protein 203
7128	IFNK	0.0063	0.2293	interferon, kappa
7129	HSCB	0.0063	0.4664	HscB iron-sulfur cluster co-chaperone homolog (E. coli)
7130	C1QTNF6	0.0063	0.3898	C1q and tumor necrosis factor related protein 6
7131	SETD2	0.0058	0.491	SET domain containing 2
7132	PTPLAD1	0.0058	0.4982	protein tyrosine phosphatase-like A domain containing 1
7133	METTL18	0.0058	0.4978	methyltransferase like 18
7134	IPPK	0.0058	0.4186	inositol 1,3,4,5,6-pentakisphosphate 2-kinase
7135	GAST	0.0058	0.4003	gastrin
7136	CEBPE	0.0058	0.4099	CCAAT/enhancer binding protein (C/EBP), epsilon
7137	B3GNT4	0.0058	0.4255	UDP-GlcNAc:betaGal beta-1,3-N-acetylglucosaminyltransferase 4
7138	PGM5-AS1	0.0057	0.3553	PGM5 antisense RNA 1 (non-protein coding)

7139	WIPF1	0.005	0.4329	WAS/WASL interacting protein family, member 1
7140	USP24	0.005	0.5023	ubiquitin specific peptidase 24
7141	ULBP3	0.005	0.3304	UL16 binding protein 3
7142	SHOC2	0.005	0.4954	soc-2 suppressor of clear homolog (C. elegans)
7143	PLAC8	0.005	0.4091	placenta-specific 8
7144	PKD1L1	0.005	0.3465	polycystic kidney disease 1 like 1
7145	MICALL1	0.005	0.4092	MICAL-like 1
7146	LOC285696	0.005	0.3288	uncharacterized LOC285696
7147	HDAC11	0.005	0.45	histone deacetylase 11
7148	GSTM5	0.005	0.42	glutathione S-transferase mu 5
7149	GSDMD	0.005	0.3829	gasdermin D
7150	BCR	0.005	0.4095	breakpoint cluster region
7151	LOC338758	0.0043	0.3196	uncharacterized LOC338758
7152	NCR2	0.0042	0.4128	natural cytotoxicity triggering receptor 2
7153	MC1R	0.0042	0.4398	melanocortin 1 receptor (alpha melanocyte stimulating hormone receptor)
7154	INHBB	0.0042	0.3229	inhibin, beta B
7155	GOLGA2P5	0.0042	0.3743	golgin A2 pseudogene 5
7156	FLOT1	0.0042	0.4127	flotillin 1
7157	CALM1	0.0042	0.5061	calmodulin 1 (phosphorylase kinase, delta)
7158	ACAT1	0.0042	0.487	acetyl-CoA acetyltransferase 1
7159	ZNF501	0.0037	0.4037	zinc finger protein 501
7160	ZCCHC5	0.0037	0.3306	zinc finger, CCHC domain containing 5
7161	DTWD2	0.0037	0.4239	DTW domain containing 2
7162	C2orf63	0.0037	0.4822	chromosome 2 open reading frame 63
7163	C11orf1	0.0037	0.4527	chromosome 11 open reading frame 1
7164	TXNDC9	0.0033	0.4843	thioredoxin domain containing 9
7165	NAGK	0.0033	0.4579	N-acetylglucosamine kinase
7166	MUL1	0.0033	0.4232	mitochondrial E3 ubiquitin protein ligase 1
7167	MOCS1	0.0033	0.4208	molybdenum cofactor synthesis 1
7168	MED12	0.0033	0.4252	mediator complex subunit 12
7169	KCNK3	0.0033	0.419	potassium channel, subfamily K, member 3
7170	FER	0.0033	0.4582	fer (fps/fes related) tyrosine kinase
7171	DIRAS3	0.0033	0.3001	DIRAS family, GTP-binding RAS-like 3
7172	CNOT4	0.0033	0.4784	CCR4-NOT transcription complex, subunit 4
7173	CDH15	0.0033	0.4205	cadherin 15, type 1, M-cadherin (myotubule)
7174	ZNF731P	0.0029	0.0004	zinc finger protein 731, pseudogene
7175	BSCL2	0.0029	0.4196	Berardinelli-Seip congenital lipodystrophy 2 (seipin)
7176	ZNF84	0.0025	0.4917	zinc finger protein 84
7177	VTN	0.0025	0.3864	vitronectin
7178	TCHP	0.0025	0.4806	trichoplein, keratin filament binding
7179	SPP1	0.0025	0.3467	secreted phosphoprotein 1
7180	SIX4	0.0025	0.3075	SIX homeobox 4
7181	PKM	0.0025	0.4481	pyruvate kinase, muscle
7182	LMOD2	0.0025	0.3203	leiomodin 2 (cardiac)
7183	KNG1	0.0025	0.3999	kininogen 1
7184	IL32	0.0025	0.3523	interleukin 32
7185	FARS2	0.0025	0.4745	phenylalanyl-tRNA synthetase 2, mitochondrial
7186	F9	0.0025	0.3436	coagulation factor IX
7187	DIP2C	0.0025	0.3992	DIP2 disco-interacting protein 2 homolog C (Drosophila)
7188	BAAT	0.0025	0.4011	bile acid CoA: amino acid N-acyltransferase (glycine N-choloyltransferase)
7189	ZYG11A	0.0017	0.2519	zyg-11 homolog A (C. elegans)
7190	TRIM3	0.0017	0.443	tripartite motif containing 3
7191	ST7L	0.0017	0.421	suppression of tumorigenicity 7 like
7192	RPRD2	0.0017	0.4746	regulation of nuclear pre-mRNA domain containing 2
7193	PYCR1	0.0017	0.4146	pyrroline-5-carboxylate reductase 1
7194	PGLS	0.0017	0.4141	6-phosphogluconolactonase
7195	GNAL	0.0017	0.3208	guanine nucleotide binding protein (G protein), alpha activating activity polypeptide, olfactory type
7196	FBP2	0.0017	0.4017	fructose-1,6-bisphosphatase 2
7197	EGLN1	0.0017	0.4285	egl nine homolog 1 (C. elegans)
7198	COX7B	0.0017	0.457	cytochrome c oxidase subunit VIIb
7199	BAD	0.0017	0.3972	BCL2-associated agonist of cell death
7200	TAOK2	0.0008	0.4591	TAO kinase 2
7201	STX3	0.0008	0.4352	syntaxin 3
7202	REPS2	0.0008	0.3631	RALBP1 associated Eps domain containing 2
7203	NANS	0.0008	0.4704	N-acetylneuraminic acid synthase
7204	KLHL36	0.0008	0.3963	kelch-like 36 (Drosophila)
7205	HSD17B1	0.0008	0.3709	hydroxysteroid (17-beta) dehydrogenase 1
7206	GYPE	0.0008	0.3424	glycophorin E (MNS blood group)
7207	EMX2	0.0008	0.3404	empty spiracles homeobox 2
7208	DCLK1	0.0008	0.3487	doublecortin-like kinase 1
7209	CACNA1C	0.0008	0.4334	calcium channel, voltage-dependent, L type, alpha 1C subunit
7210	UBL3	0	0.4793	ubiquitin-like 3
7211	SGCZ	0	0.2906	sarcoglycan, zeta
7212	PPHLN1	0	0.5041	periphilin 1
7213	PLIN1	0	0.3927	perilipin 1
7214	MRPL21	0	0.4567	mitochondrial ribosomal protein L21
7215	DNM1	0	0.3657	dynamin 1
7216	C20orf166	0	0.3724	chromosome 20 open reading frame 166
7217	BMP5	0	0.3828	bone morphogenetic protein 5
7218	ARG1	0	0.3424	arginase, liver
7219	ZNF510	-0.0008	0.4832	zinc finger protein 510
7220	ULK2	-0.0008	0.4642	unc-51-like kinase 2 (C. elegans)
7221	TMED5	-0.0008	0.4887	transmembrane emp24 protein transport domain containing 5
7222	RASSF4	-0.0008	0.3845	Ras association (RalGDS/AF-6) domain family member 4

7223	PDZRN3	-0.0008	0.34	PDZ domain containing ring finger 3
7224	NOS1	-0.0008	0.4187	nitric oxide synthase 1 (neuronal)
7225	MEP1A	-0.0008	0.3412	meprin A, alpha (PABA peptide hydrolase)
7226	ENTPD1	-0.0008	0.3685	ectonucleoside triphosphate diphosphohydrolase 1
7227	CDK18	-0.0008	0.4077	cyclin-dependent kinase 18
7228	BCKDK	-0.0008	0.442	branched chain ketoacid dehydrogenase kinase
7229	ATXN7	-0.0008	0.4431	ataxin 7
7230	ABCB7	-0.0008	0.5061	ATP-binding cassette, sub-family B (MDR/TAP), member 7
7231	SIM1	-0.0012	0.3452	single-minded homolog 1 (Drosophila)
7232	ACSS1	-0.0012	0.3507	acyl-CoA synthetase short-chain family member 1
7233	ABCB10	-0.0012	0.4707	ATP-binding cassette, sub-family B (MDR/TAP), member 10
7234	ZSCAN23	-0.0014	0.3205	zinc finger and SCAN domain containing 23
7235	ZNF394	-0.0017	0.4647	zinc finger protein 394
7236	RCE1	-0.0017	0.4059	RCE1 homolog, prenyl protein protease (S. cerevisiae)
7237	MYNN	-0.0017	0.4906	myoneurin
7238	KIAA0368	-0.0017	0.508	KIAA0368
7239	CYP17A1	-0.0017	0.4072	cytochrome P450, family 17, subfamily A, polypeptide 1
7240	TRMT61B	-0.0025	0.4984	tRNA methyltransferase 61 homolog B (S. cerevisiae)
7241	RALA	-0.0025	0.4971	v-ral simian leukemia viral oncogene homolog A (ras related)
7242	NICN1	-0.0025	0.3997	nicolin 1
7243	MEFV	-0.0025	0.3411	Mediterranean fever
7244	MAN1C1	-0.0025	0.378	mannosidase, alpha, class 1C, member 1
7245	LY96	-0.0025	0.4232	lymphocyte antigen 96
7246	LCE1B	-0.0025	0.3157	late cornified envelope 1B
7247	KBTBD10	-0.0025	0.3275	kelch repeat and BTB (POZ) domain containing 10
7248	GSTO1	-0.0025	0.4594	glutathione S-transferase omega 1
7249	CHML	-0.0025	0.4637	choroideremia-like (Rab escort protein 2)
7250	BOLA1	-0.0025	0.4177	bolA homolog 1 (E. coli)
7251	ATP2A1	-0.0025	0.3776	ATPase, Ca++ transporting, cardiac muscle, fast twitch 1
7252	ACSS2	-0.0025	0.4023	acyl-CoA synthetase short-chain family member 2
7253	LOC284379	-0.0029	0.3665	solute carrier family 7 (cationic amino acid transporter, y+ system), member 3 pseudogene
7254	GJA9	-0.0029	0.3956	gap junction protein, alpha 9, 59kDa
7255	TRANK1	-0.0033	0.4174	tetratricopeptide repeat and ankyrin repeat containing 1
7256	PSCA	-0.0033	0.3373	prostate stem cell antigen
7257	MARCO	-0.0033	0.3828	macrophage receptor with collagenous structure
7258	KCNJ14	-0.0033	0.3857	potassium inwardly-rectifying channel, subfamily J, member 14
7259	GABPA	-0.0033	0.4528	GA binding protein transcription factor, alpha subunit 60kDa
7260	FSHB	-0.0033	0.384	follicle stimulating hormone, beta polypeptide
7261	ADORA2B	-0.0033	0.3431	adenosine A2b receptor
7262	ZNF776	-0.0037	0.5047	zinc finger protein 776
7263	ZNF559	-0.0037	0.5042	zinc finger protein 559
7264	TMEM241	-0.0037	0.3547	transmembrane protein 241
7265	KRTAP4-12	-0.0037	0.3338	keratin associated protein 4-12
7266	KRTAP17-1	-0.0037	0.3577	keratin associated protein 17-1
7267	CCDC61	-0.0037	0.3805	coiled-coil domain containing 61
7268	AFAP1-AS1	-0.0037	0.3933	AFAP1 antisense RNA 1 (non-protein coding)
7269	VCAN	-0.0042	0.4118	versican
7270	UBE2Z	-0.0042	0.4923	ubiquitin-conjugating enzyme E2Z
7271	TALDO1	-0.0042	0.4659	transaldolase 1
7272	STX11	-0.0042	0.3783	syntaxin 11
7273	SLC19A2	-0.0042	0.4781	solute carrier family 19 (thiamine transporter), member 2
7274	RPL13	-0.0042	0.4065	ribosomal protein L13
7275	RNF122	-0.0042	0.4525	ring finger protein 122
7276	PZP	-0.0042	0.4092	pregnancy-zone protein
7277	PLD3	-0.0042	0.4379	phospholipase D family, member 3
7278	MYL4	-0.0042	0.3545	myosin, light chain 4, alkali; atrial, embryonic
7279	HN1L	-0.0042	0.4583	hematological and neurological expressed 1-like
7280	FRYL	-0.0042	0.4949	FRY-like
7281	CCND2	-0.0042	0.3962	cyclin D2
7282	ATP6V0D1	-0.0042	0.4589	ATPase, H+ transporting, lysosomal 38kDa, V0 subunit d1
7283	APBA1	-0.0042	0.4208	amyloid beta (A4) precursor protein-binding, family A, member 1
7284	ZNF621	-0.005	0.4599	zinc finger protein 621
7285	PUM2	-0.005	0.4987	pumilio homolog 2 (Drosophila)
7286	PIEZO2	-0.005	0.3657	piezo-type mechanosensitive ion channel component 2
7287	PCNP	-0.005	0.4912	PEST proteolytic signal containing nuclear protein
7288	LRRC48	-0.005	0.4119	leucine rich repeat containing 48
7289	HLA-DOA	-0.005	0.4053	major histocompatibility complex, class II, DO alpha
7290	FOXP4	-0.005	0.4188	forkhead box P4
7291	EPOR	-0.005	0.4427	erythropoietin receptor
7292	C1orf123	-0.005	0.4712	chromosome 1 open reading frame 123
7293	INSC	-0.0057	0.3112	inscuteable homolog (Drosophila)
7294	STX7	-0.0058	0.4934	syntaxin 7
7295	SPATS2	-0.0058	0.513	spermatogenesis associated, serine-rich 2
7296	RUSC2	-0.0058	0.4489	RUN and SH3 domain containing 2
7297	MED20	-0.0058	0.4844	mediator complex subunit 20
7298	MCTP1	-0.0058	0.43	multiple C2 domains, transmembrane 1
7299	LYPLA1	-0.0058	0.4845	lysophospholipase I
7300	HSPA4L	-0.0058	0.4687	heat shock 70kDa protein 4-like
7301	FCN2	-0.0058	0.3876	ficolin (collagen/fibrinogen domain containing lectin) 2 (hucolin)
7302	CHRNB1	-0.0058	0.3256	cholinergic receptor, nicotinic, beta 1 (muscle)
7303	CDK14	-0.0058	0.4537	cyclin-dependent kinase 14
7304	C11orf57	-0.0058	0.513	chromosome 11 open reading frame 57
7305	ATP4B	-0.0058	0.3944	ATPase, H+/K+ exchanging, beta polypeptide
7306	ARID5B	-0.0058	0.4179	AT rich interactive domain 5B (MRF1-like)

7307	ZBTB40	-0.0067	0.4275	zinc finger and BTB domain containing 40
7308	TXLNA	-0.0067	0.5	taxilin alpha
7309	TTN	-0.0067	0.3622	titin
7310	ITGB7	-0.0067	0.3897	integrin, beta 7
7311	GDF10	-0.0067	0.4007	growth differentiation factor 10
7312	GAK	-0.0067	0.4104	cyclin G associated kinase
7313	ATP6V1C1	-0.0067	0.5011	ATPase, H+ transporting, lysosomal 42kDa, V1 subunit C1
7314	ASIC1	-0.0067	0.4369	acid-sensing (proton-gated) ion channel 1
7315	ABHD14A	-0.0067	0.4171	abhydrolase domain containing 14A
7316	ZNF643	-0.0075	0.4474	zinc finger protein 643
7317	SULT1A2	-0.0075	0.4078	sulfotransferase family, cytosolic, 1A, phenol-preferring, member 2
7318	SIRT3	-0.0075	0.4259	sirtuin 3
7319	SEC14L4	-0.0075	0.4055	SEC14-like 4 (S. cerevisiae)
7320	RBCK1	-0.0075	0.4254	RanBP-type and C3HC4-type zinc finger containing 1
7321	PHOSPHO2	-0.0075	0.4938	phosphatase, orphan 2
7322	MYH15	-0.0075	0.3879	myosin, heavy chain 15
7323	MGC27345	-0.0075	0.4576	uncharacterized protein MGC27345
7324	MGC13053	-0.0075	0.237	uncharacterized MGC13053
7325	LTK	-0.0075	0.4268	leukocyte receptor tyrosine kinase
7326	GDPD3	-0.0075	0.3827	glycerophosphodiester phosphodiesterase domain containing 3
7327	DCAKD	-0.0075	0.4169	dephospho-CoA kinase domain containing
7328	CTLA4	-0.0075	0.388	cytotoxic T-lymphocyte-associated protein 4
7329	CISD2	-0.0075	0.483	CDGSH iron sulfur domain 2
7330	C3orf18	-0.0075	0.4254	chromosome 3 open reading frame 18
7331	C17orf61	-0.0075	0.4207	chromosome 17 open reading frame 61
7332	ARL6	-0.0075	0.493	ADP-ribosylation factor-like 6
7333	ALDH4A1	-0.0075	0.4122	aldehyde dehydrogenase 4 family, member A1
7334	UPK1A	-0.0083	0.4019	uroplakin 1A
7335	TPK1	-0.0083	0.4878	thiamin pyrophosphokinase 1
7336	TNFAIP6	-0.0083	0.3813	tumor necrosis factor, alpha-induced protein 6
7337	STK4	-0.0083	0.4642	serine/threonine kinase 4
7338	NSUN5P1	-0.0083	0.4003	NOP2/Sun domain family, member 5 pseudogene 1
7339	NBEAL2	-0.0083	0.4015	neurobeachin-like 2
7340	METTL16	-0.0083	0.484	methyltransferase like 16
7341	ISL1	-0.0083	0.3108	ISL LIM homeobox 1
7342	FES	-0.0083	0.3943	feline sarcoma oncogene
7343	DIMT1	-0.0083	0.4888	DIM1 dimethyladenosine transferase 1 homolog (S. cerevisiae)
7344	BPIFC	-0.0083	0	BPI fold containing family C
7345	BCS1L	-0.0083	0.4613	BCS1-like (S. cerevisiae)
7346	KIF21A	-0.0088	0.4964	kinesin family member 21A
7347	C17orf103	-0.0088	0.4017	chromosome 17 open reading frame 103
7348	SFRP5	-0.0092	0.4094	secreted frizzled-related protein 5
7349	PLXDC1	-0.0092	0.3953	plexin domain containing 1
7350	MYL7	-0.0092	0.4066	myosin, light chain 7, regulatory
7351	MDFI	-0.0092	0.4208	MyoD family inhibitor
7352	IGFBP1	-0.0092	0.2929	insulin-like growth factor binding protein 1
7353	CDK7	-0.0092	0.4968	cyclin-dependent kinase 7
7354	C5	-0.0092	0.4283	complement component 5
7355	YPEL2	-0.01	0.4495	yippee-like 2 (Drosophila)
7356	TNNI1	-0.01	0.394	troponin I type 1 (skeletal, slow)
7357	SLC25A13	-0.01	0.4943	solute carrier family 25 (aspartate/glutamate carrier), member 13
7358	NLRP10	-0.01	0.3246	NLR family, pyrin domain containing 10
7359	NCR1	-0.01	0.4243	natural cytotoxicity triggering receptor 1
7360	MOB1A	-0.01	0.492	MOB kinase activator 1A
7361	MICALCL	-0.01	0.3041	MICAL C-terminal like
7362	METTL14	-0.01	0.5205	methyltransferase like 14
7363	HPS3	-0.01	0.5176	Hermansky-Pudlak syndrome 3
7364	ACRC	-0.01	0.3875	acidic repeat containing
7365	WIF1	-0.0108	0.3051	WNT inhibitory factor 1
7366	TXK	-0.0108	0.3455	TXK tyrosine kinase
7367	TTC19	-0.0108	0.5068	tetratricopeptide repeat domain 19
7368	TGFB1	-0.0108	0.4307	transforming growth factor, beta 1
7369	RHBDF2	-0.0108	0.3943	rhomboid 5 homolog 2 (Drosophila)
7370	PUS7	-0.0108	0.4843	pseudouridylate synthase 7 homolog (S. cerevisiae)
7371	PDE11A	-0.0108	0.418	phosphodiesterase 11A
7372	CYB561D2	-0.0108	0.4252	cytochrome b-561 domain containing 2
7373	BTNL8	-0.0108	0.3989	butyrophilin-like 8
7374	TRDN	-0.0113	0.2594	triadin
7375	THAP2	-0.0113	0.4851	THAP domain containing, apoptosis associated protein 2
7376	PALM2	-0.0113	0.3147	paralemmin 2
7377	KRTAP4-4	-0.0113	0.3295	keratin associated protein 4-4
7378	HTRA4	-0.0113	0.276	HtrA serine peptidase 4
7379	HPDL	-0.0113	0.3573	4-hydroxyphenylpyruvate dioxygenase-like
7380	B3GNT8	-0.0113	0.3957	UDP-GlcNAc:betaGal beta-1,3-N-acetylglucosaminyltransferase 8
7381	RPL30	-0.0117	0.4687	ribosomal protein L30
7382	PPT1	-0.0117	0.5063	palmitoyl-protein thioesterase 1
7383	MAGED1	-0.0117	0.4877	melanoma antigen family D, 1
7384	KDM4C	-0.0117	0.4941	lysine (K)-specific demethylase 4C
7385	GNRHR	-0.0117	0.387	gonadotropin-releasing hormone receptor
7386	CXCL10	-0.0117	0.3693	chemokine (C-X-C motif) ligand 10
7387	C3orf36	-0.0117	0.419	chromosome 3 open reading frame 36
7388	ZHX2	-0.0125	0.4647	zinc fingers and homeoboxes 2
7389	SRP14	-0.0125	0.4956	signal recognition particle 14kDa (homologous Alu RNA binding protein)
7390	SERBP1	-0.0125	0.4916	SERPINE1 mRNA binding protein 1

7391	ORC2	-0.0125	0.5076	origin recognition complex, subunit 2
7392	NOL10	-0.0125	0.475	nucleolar protein 10
7393	LRPAP1	-0.0125	0.4587	low density lipoprotein receptor-related protein associated protein 1
7394	GC	-0.0125	0.328	group-specific component (vitamin D binding protein)
7395	CHRDL2	-0.0125	0.3879	chordin-like 2
7396	CDH7	-0.0125	0.3974	cadherin 7, type 2
7397	ARID4A	-0.0125	0.492	AT rich interactive domain 4A (RBP1-like)
7398	SPATA1	-0.0129	0.3459	spermatogenesis associated 1
7399	FAM185A	-0.0129	0.4184	family with sequence similarity 185, member A
7400	USP9X	-0.0133	0.4996	ubiquitin specific peptidase 9, X-linked
7401	TNFSF8	-0.0133	0.4038	tumor necrosis factor (ligand) superfamily, member 8
7402	RPS3	-0.0133	0.4652	ribosomal protein S3
7403	PLXND1	-0.0133	0.4118	plexin D1
7404	PCID2	-0.0133	0.5088	PCI domain containing 2
7405	KIAA1279	-0.0133	0.5183	KIAA1279
7406	CRHR2	-0.0133	0.4004	corticotropin releasing hormone receptor 2
7407	CMA1	-0.0133	0.4066	chymase 1, mast cell
7408	ATP11A	-0.0133	0.4015	ATPase, class VI, type 11A
7409	ZNF691	-0.0137	0.4331	zinc finger protein 691
7410	SLC37A2	-0.0137	0.3848	solute carrier family 37 (glycerol-3-phosphate transporter), member 2
7411	CHCHD10	-0.0137	0.415	coiled-coil-helix-coiled-coil-helix domain containing 10
7412	C3orf78	-0.0137	0.4275	chromosome 3 open reading frame 78
7413	ADAMTS16	-0.0137	0.3597	ADAM metallopeptidase with thrombospondin type 1 motif, 16
7414	TRIM66	-0.0142	0.3751	tripartite motif containing 66
7415	RDH16	-0.0142	0.4316	retinol dehydrogenase 16 (all-trans)
7416	NECAB3	-0.0142	0.425	N-terminal EF-hand calcium binding protein 3
7417	NDST2	-0.0142	0.4205	N-deacetylase/N-sulfotransferase (heparan glucosaminyl) 2
7418	IL1A	-0.0142	0.3763	interleukin 1, alpha
7419	HTR1B	-0.0142	0.4137	5-hydroxytryptamine (serotonin) receptor 1B, G protein-coupled
7420	ERGIC2	-0.0142	0.5021	ERGIC and golgi 2
7421	EFHA1	-0.0142	0.5059	EF-hand domain family, member A1
7422	CYP2C19	-0.0142	0.3607	cytochrome P450, family 2, subfamily C, polypeptide 19
7423	C10orf95	-0.0142	0.408	chromosome 10 open reading frame 95
7424	SAPCD1	-0.0143	0.3815	suppressor APC domain containing 1
7425	GLOD5	-0.0143	0.3795	glyoxalase domain containing 5
7426	CLDN20	-0.0143	0.3525	claudin 20
7427	ZXDC	-0.015	0.4253	ZXD family zinc finger C
7428	ZSWIM7	-0.015	0.4751	zinc finger, SWIM-type containing 7
7429	SOX4	-0.015	0.4004	SRY (sex determining region Y)-box 4
7430	RNASE7	-0.015	0.337	ribonuclease, RNase A family, 7
7431	PPP1R14A	-0.015	0.3244	protein phosphatase 1, regulatory (inhibitor) subunit 14A
7432	PITPNC1	-0.015	0.4379	phosphatidylinositol transfer protein, cytoplasmic 1
7433	NHLRC2	-0.015	0.5209	NHL repeat containing 2
7434	GATA5	-0.015	0.3845	GATA binding protein 5
7435	DGAT1	-0.015	0.4146	diacylglycerol O-acyltransferase 1
7436	LOC100288911	-0.0157	0.3401	uncharacterized LOC100288911
7437	SULT1A1	-0.0158	0.357	sulfotransferase family, cytosolic, 1A, phenol-preferring, member 1
7438	SRI	-0.0158	0.5058	sorcin
7439	RPL13A	-0.0158	0.4517	ribosomal protein L13a
7440	FAR2	-0.0158	0.4654	fatty acyl CoA reductase 2
7441	EPO	-0.0158	0.4175	erythropoietin
7442	EPHB6	-0.0158	0.3941	EPH receptor B6
7443	DGKE	-0.0158	0.4406	diacylglycerol kinase, epsilon 64kDa
7444	C7orf49	-0.0158	0.4921	chromosome 7 open reading frame 49
7445	BAHD1	-0.0158	0.3953	bromo adjacent homology domain containing 1
7446	TP53RK	-0.0162	0.4959	TP53 regulating kinase
7447	IFT80	-0.0162	0.5045	intraflagellar transport 80 homolog (Chlamydomonas)
7448	FAM220A	-0.0162	0.513	family with sequence similarity 220, member A
7449	CD274	-0.0162	0.3855	CD274 molecule
7450	ST20	-0.0164	0.3827	suppressor of tumorigenicity 20
7451	RBM15B	-0.0167	0.457	RNA binding motif protein 15B
7452	PPP3CA	-0.0167	0.4996	protein phosphatase 3, catalytic subunit, alpha isozyme
7453	KDM3B	-0.0167	0.5122	lysine (K)-specific demethylase 3B
7454	HYDIN	-0.0167	0.413	HYDIN, axonemal central pair apparatus protein
7455	DFNA5	-0.0167	0.4535	deafness, autosomal dominant 5
7456	CP	-0.0167	0.3734	ceruloplasmin (ferroxidase)
7457	ARHGEF2	-0.0167	0.4277	Rho/Rac guanine nucleotide exchange factor (GEF) 2
7458	SIGLEC16	-0.0171	0.3928	sialic acid binding Ig-like lectin 16 (gene/pseudogene)
7459	GGT1	-0.0171	0.4066	gamma-glutamyltransferase 1
7460	TRPM3	-0.0175	0.4092	transient receptor potential cation channel, subfamily M, member 3
7461	STXBP2	-0.0175	0.4179	syntaxin binding protein 2
7462	SNTN	-0.0175	0.251	sentan, cilia apical structure protein
7463	SLC15A3	-0.0175	0.3929	solute carrier family 15, member 3
7464	SENP6	-0.0175	0.5015	SUMO1/sentrin specific peptidase 6
7465	PCP2	-0.0175	0.3694	Purkinje cell protein 2
7466	MVK	-0.0175	0.4566	mevalonate kinase
7467	KCNMA1	-0.0175	0.4196	potassium large conductance calcium-activated channel, subfamily M, alpha member 1
7468	ELOVL6	-0.0175	0.4812	ELOVL fatty acid elongase 6
7469	EFCAB6	-0.0175	0.4089	EF-hand calcium binding domain 6
7470	DDX3X	-0.0175	0.4861	DEAD (Asp-Glu-Ala-Asp) box polypeptide 3, X-linked
7471	CUTA	-0.0175	0.4752	cutA divalent cation tolerance homolog (E. coli)
7472	C1orf192	-0.0175	0.3561	chromosome 1 open reading frame 192
7473	ATR	-0.0175	0.5008	ataxia telangiectasia and Rad3 related
7474	ASB8	-0.0175	0.5146	ankyrin repeat and SOCS box containing 8

7475	ANKRD6	-0.0175	0.441	ankyrin repeat domain 6
7476	ABCB6	-0.0175	0.3971	ATP-binding cassette, sub-family B (MDR/TAP), member 6
7477	SNORA64	-0.0182	0.3952	small nucleolar RNA, H/ACA box 64
7478	PCDHA3	-0.0182	0.4392	protocadherin alpha 3
7479	TTC33	-0.0183	0.5036	tetratricopeptide repeat domain 33
7480	SLAMF8	-0.0183	0.3822	SLAM family member 8
7481	PPT2	-0.0183	0.462	palmitoyl-protein thioesterase 2
7482	NBAS	-0.0183	0.51	neuroblastoma amplified sequence
7483	MYO1A	-0.0183	0.4068	myosin IA
7484	MAS1	-0.0183	0.3969	MAS1 oncogene
7485	CYC1	-0.0183	0.4669	cytochrome c-1
7486	CHPF	-0.0183	0.4504	chondroitin polymerizing factor
7487	CARKD	-0.0183	0.4972	carbohydrate kinase domain containing
7488	MDS2	-0.0186	0.3613	myelodysplastic syndrome 2 translocation associated
7489	FAM53A	-0.0186	0.2734	family with sequence similarity 53, member A
7490	ZNF558	-0.0188	0.4958	zinc finger protein 558
7491	SLC44A5	-0.0188	0.3459	solute carrier family 44, member 5
7492	NEIL2	-0.0188	0.3658	nei endonuclease VIII-like 2 (E. coli)
7493	MYCBPAP	-0.0188	0.3805	MYCBP associated protein
7494	AXIN2	-0.0188	0.4084	axin 2
7495	XPA	-0.0192	0.5046	xeroderma pigmentosum, complementation group A
7496	TTC3	-0.0192	0.5013	tetratricopeptide repeat domain 3
7497	RTN3	-0.0192	0.4832	reticulon 3
7498	RBPJL	-0.0192	0.3992	recombination signal binding protein for immunoglobulin kappa J region-like
7499	PSMB3	-0.0192	0.4736	proteasome (prosome, macropain) subunit, beta type, 3
7500	PEF1	-0.0192	0.4735	penta-EF-hand domain containing 1
7501	NTSR1	-0.0192	0.4116	neurotensin receptor 1 (high affinity)
7502	DDIT4	-0.0192	0.4534	DNA-damage-inducible transcript 4
7503	ATP2B1	-0.0192	0.4938	ATPase, Ca++ transporting, plasma membrane 1
7504	ADAMTS12	-0.0192	0.4294	ADAM metallopeptidase with thrombospondin type 1 motif, 12
7505	TM7SF3	-0.02	0.5249	transmembrane 7 superfamily member 3
7506	SSTR4	-0.02	0.4133	somatostatin receptor 4
7507	SLC14A2	-0.02	0.4051	solute carrier family 14 (urea transporter), member 2
7508	SEC24C	-0.02	0.4901	SEC24 family, member C (S. cerevisiae)
7509	SCN4A	-0.02	0.4182	sodium channel, voltage-gated, type IV, alpha subunit
7510	PON1	-0.02	0.3755	paraoxonase 1
7511	PLXNA1	-0.02	0.4265	plexin A1
7512	MRFAP1	-0.02	0.5135	Morf4 family associated protein 1
7513	INPP5D	-0.02	0.3924	inositol polyphosphate-5-phosphatase, 145kDa
7514	IKZF5	-0.02	0.4673	IKAROS family zinc finger 5 (Pegasus)
7515	DHFRL1	-0.02	0.4949	dihydrofolate reductase-like 1
7516	PCSK6	-0.0208	0.4394	proprotein convertase subtilisin/kexin type 6
7517	NXPE3	-0.0208	0.508	neurexophilin and PC-esterase domain family, member 3
7518	FTCD	-0.0208	0.4175	formiminotransferase cyclodeaminase
7519	FLRT1	-0.0208	0.4035	fibronectin leucine rich transmembrane protein 1
7520	C1orf54	-0.0208	0.4114	chromosome 1 open reading frame 54
7521	BHLHB9	-0.0208	0.4574	basic helix-loop-helix domain containing, class B, 9
7522	ZNF90	-0.0213	0.2922	zinc finger protein 90
7523	LOC100128239	-0.0213	0.4094	uncharacterized LOC100128239
7524	LCE3D	-0.0213	0.3801	late cornified envelope 3D
7525	IL17RE	-0.0213	0.3748	interleukin 17 receptor E
7526	HEPACAM2	-0.0213	0.2705	HEPACAM family member 2
7527	GIMAP7	-0.0213	0.3864	GTPase, IMAP family member 7
7528	DDX26B	-0.0213	0.4788	DEAD/H (Asp-Glu-Ala-Asp/His) box polypeptide 26B
7529	CWF19L2	-0.0213	0.5121	CWF19-like 2, cell cycle control (S. pombe)
7530	CCDC13	-0.0213	0.3924	coiled-coil domain containing 13
7531	ADAMTSL5	-0.0213	0.4154	ADAMTS-like 5
7532	TPP2	-0.0217	0.5007	tripeptidyl peptidase II
7533	TAAR2	-0.0217	0.3708	trace amine associated receptor 2
7534	STIM1	-0.0217	0.4487	stromal interaction molecule 1
7535	SLC25A21	-0.0217	0.3913	solute carrier family 25 (mitochondrial oxoadipate carrier), member 21
7536	SH3BP2	-0.0217	0.4355	SH3-domain binding protein 2
7537	ACTR1B	-0.0217	0.4541	ARP1 actin-related protein 1 homolog B, centractin beta (yeast)
7538	ZNF747	-0.0225	0.4129	zinc finger protein 747
7539	VPS26A	-0.0225	0.5094	vacuolar protein sorting 26 homolog A (S. pombe)
7540	STK17B	-0.0225	0.4724	serine/threonine kinase 17b
7541	RCAN2	-0.0225	0.4168	regulator of calcineurin 2
7542	PKHD1	-0.0225	0.3747	polycystic kidney and hepatic disease 1 (autosomal recessive)
7543	PCDHB12	-0.0225	0.4237	protocadherin beta 12
7544	OR7C2	-0.0225	0.411	olfactory receptor, family 7, subfamily C, member 2
7545	NSUN3	-0.0225	0.4884	NOP2/Sun domain family, member 3
7546	NRG2	-0.0225	0.4176	neuregulin 2
7547	L1TD1	-0.0225	0.3913	LINE-1 type transposase domain containing 1
7548	ISOC1	-0.0225	0.4971	isochorismatase domain containing 1
7549	GPR32	-0.0225	0.3908	G protein-coupled receptor 32
7550	FAM65A	-0.0225	0.4602	family with sequence similarity 65, member A
7551	CTH	-0.0225	0.4583	cystathionase (cystathionine gamma-lyase)
7552	CHL1	-0.0225	0.3754	cell adhesion molecule with homology to L1CAM (close homolog of L1)
7553	CDR1	-0.0225	0.3447	cerebellar degeneration-related protein 1, 34kDa
7554	CCS	-0.0225	0.4168	copper chaperone for superoxide dismutase
7555	C4orf47	-0.0225	0.3353	chromosome 4 open reading frame 47
7556	BEGAIN	-0.0225	0.428	brain-enriched guanylate kinase-associated homolog (rat)
7557	ARL16	-0.0225	0.4015	ADP-ribosylation factor-like 16
7558	C1orf186	-0.0229	0.375	chromosome 1 open reading frame 186

7559	TOPORS	-0.0233	0.5034	topoisomerase I binding, arginine/serine-rich, E3 ubiquitin protein ligase
7560	STX10	-0.0233	0.4274	syntaxin 10
7561	OR7A17	-0.0233	0.3756	olfactory receptor, family 7, subfamily A, member 17
7562	NPAT	-0.0233	0.5018	nuclear protein, ataxia-telangiectasia locus
7563	NAA16	-0.0233	0.4949	N(alpha)-acetyltransferase 16, NatA auxiliary subunit
7564	DCAF6	-0.0233	0.5038	DDB1 and CUL4 associated factor 6
7565	CACNG5	-0.0233	0.4179	calcium channel, voltage-dependent, gamma subunit 5
7566	C2orf83	-0.0233	0.3993	chromosome 2 open reading frame 83
7567	ATP6V0A2	-0.0233	0.5105	ATPase, H+ transporting, lysosomal V0 subunit a2
7568	ZNF581	-0.0237	0.3987	zinc finger protein 581
7569	TTC32	-0.0237	0.466	tetratricopeptide repeat domain 32
7570	RNF181	-0.0237	0.4324	ring finger protein 181
7571	KBTBD12	-0.0237	0.306	kelch repeat and BTB (POZ) domain containing 12
7572	DDX59	-0.0237	0.5122	DEAD (Asp-Glu-Ala-Asp) box polypeptide 59
7573	C19orf69	-0.0237	0.3806	chromosome 19 open reading frame 69
7574	UFSP2	-0.0242	0.5261	UFM1-specific peptidase 2
7575	sept-08	-0.0242	0.4922	septin 8
7576	RPL10L	-0.0242	0.3133	ribosomal protein L10-like
7577	GDF3	-0.0242	0.4181	growth differentiation factor 3
7578	CASP1	-0.0242	0.4405	caspase 1, apoptosis-related cysteine peptidase
7579	BEST2	-0.0242	0.4146	bestrophin 2
7580	ARHGAP26	-0.0242	0.4311	Rho GTPase activating protein 26
7581	TRHDE-AS1	-0.0243	0.3702	TRHDE antisense RNA 1 (non-protein coding)
7582	MCM9	-0.0244	0.5074	minichromosome maintenance complex component 9
7583	ZNF304	-0.025	0.5169	zinc finger protein 304
7584	STAP1	-0.025	0.352	signal transducing adaptor family member 1
7585	RPLP2	-0.025	0.4693	ribosomal protein, large, P2
7586	HK1	-0.025	0.4697	hexokinase 1
7587	FAM161B	-0.025	0.353	family with sequence similarity 161, member B
7588	CEP68	-0.025	0.4936	centrosomal protein 68kDa
7589	CCR5	-0.025	0.3604	chemokine (C-C motif) receptor 5 (gene/pseudogene)
7590	C6orf120	-0.025	0.5039	chromosome 6 open reading frame 120
7591	C4BPA	-0.025	0.3947	complement component 4 binding protein, alpha
7592	BCL2L15	-0.025	0.3433	BCL2-like 15
7593	ARR3	-0.025	0.4197	arrestin 3, retinal (X-arrestin)
7594	ALDH8A1	-0.025	0.2718	aldehyde dehydrogenase 8 family, member A1
7595	PRKRIR	-0.0257	0.4874	protein-kinase, interferon-inducible double stranded RNA dependent inhibitor, repressor of (P58 repressor)
7596	NHSL1	-0.0257	0.3753	NHS-like 1
7597	VPS51	-0.0258	0.4518	vacuolar protein sorting 51 homolog (S. cerevisiae)
7598	PSEN2	-0.0258	0.4349	presenilin 2 (Alzheimer disease 4)
7599	MATN4	-0.0258	0.4095	matrilin 4
7600	MAP1A	-0.0258	0.4547	microtubule-associated protein 1A
7601	ETNK2	-0.0258	0.4485	ethanolamine kinase 2
7602	CRADD	-0.0258	0.4447	CASP2 and RIPK1 domain containing adaptor with death domain
7603	CDK20	-0.0258	0.4199	cyclin-dependent kinase 20
7604	ARHGEF1	-0.0258	0.4487	Rho guanine nucleotide exchange factor (GEF) 1
7605	TET1	-0.0262	0.4638	tet methylcytosine dioxygenase 1
7606	RRN3P2	-0.0262	0.4173	RNA polymerase I transcription factor homolog (S. cerevisiae) pseudogene 2
7607	OR51E1	-0.0262	0.35	olfactory receptor, family 51, subfamily E, member 1
7608	NOA1	-0.0262	0.5082	nitric oxide associated 1
7609	KRT74	-0.0262	0.3858	keratin 74
7610	DCD	-0.0262	0.3524	dermcidin
7611	C2orf61	-0.0262	0.3041	chromosome 2 open reading frame 61
7612	C11orf83	-0.0262	0.4361	chromosome 11 open reading frame 83
7613	SF3B1	-0.0267	0.4957	splicing factor 3b, subunit 1, 155kDa
7614	SERPINA3	-0.0267	0.407	serpin peptidase inhibitor, clade A (alpha-1 antiproteinase, antitrypsin), member 3
7615	MS4A12	-0.0267	0.3791	membrane-spanning 4-domains, subfamily A, member 12
7616	LOC646808	-0.0267	0.4118	L antigen family, member 3 pseudogene
7617	KCNN3	-0.0267	0.4326	potassium intermediate/small conductance calcium-activated channel, subfamily N, member 3
7618	CEP85L	-0.0267	0	centrosomal protein 85kDa-like
7619	LOC145474	-0.0271	0	uncharacterized LOC145474
7620	LOC100129550	-0.0271	0.4024	uncharacterized LOC100129550
7621	ZNF35	-0.0275	0.4763	zinc finger protein 35
7622	ZNF347	-0.0275	0.5062	zinc finger protein 347
7623	TSC2	-0.0275	0.4505	tuberous sclerosis 2
7624	TRPC3	-0.0275	0.4138	transient receptor potential cation channel, subfamily C, member 3
7625	TNFRSF9	-0.0275	0.4237	tumor necrosis factor receptor superfamily, member 9
7626	SMEK1	-0.0275	0.4962	SMEK homolog 1, suppressor of mek1 (Dictyostelium)
7627	SLC7A6	-0.0275	0.4896	solute carrier family 7 (amino acid transporter light chain, y+L system), member 6
7628	PTMS	-0.0275	0.4108	parathymosin
7629	PLEKHM1	-0.0275	0.4528	pleckstrin homology domain containing, family M (with RUN domain) member 1
7630	PIN4	-0.0275	0.4745	protein (peptidylprolyl cis/trans isomerase) NIMA-interacting, 4 (parvulin)
7631	PDPK1	-0.0275	0.4484	3-phosphoinositide dependent protein kinase-1
7632	MIEN1	-0.0275	0.4471	migration and invasion enhancer 1
7633	KIF3B	-0.0275	0.512	kinesin family member 3B
7634	IL22RA2	-0.0275	0.326	interleukin 22 receptor, alpha 2
7635	GRIK3	-0.0275	0.4269	glutamate receptor, ionotropic, kainate 3
7636	CAMK2G	-0.0275	0.454	calcium/calmodulin-dependent protein kinase II gamma
7637	SNX24	-0.0283	0.5225	sorting nexin 24
7638	SLC29A3	-0.0283	0.4359	solute carrier family 29 (nucleoside transporters), member 3
7639	POLR1D	-0.0283	0.4948	polymerase (RNA) I polypeptide D, 16kDa
7640	POFUT2	-0.0283	0.456	protein O-fucosyltransferase 2
7641	OLR1	-0.0283	0.3761	oxidized low density lipoprotein (lectin-like) receptor 1
7642	MYBPC2	-0.0283	0.3855	myosin binding protein C, fast type

7643	MTHFD2L	-0.0283	0.4866	methylenetetrahydrofolate dehydrogenase (NADP+ dependent) 2-like
7644	MGC4294	-0.0283	0.4232	uncharacterized MGC4294
7645	KLRB1	-0.0283	0.3748	killer cell lectin-like receptor subfamily B, member 1
7646	HTR1D	-0.0283	0.4308	5-hydroxytryptamine (serotonin) receptor 1D, G protein-coupled
7647	CXCR1	-0.0283	0.3983	chemokine (C-X-C motif) receptor 1
7648	CHRNG	-0.0283	0.3975	cholinergic receptor, nicotinic, gamma (muscle)
7649	ANKRD17	-0.0283	0.5127	ankyrin repeat domain 17
7650	LOC284023	-0.0286	0.3431	uncharacterized LOC284023
7651	EGFL8	-0.0286	0.4514	EGF-like-domain, multiple 8
7652	ZNF425	-0.0288	0.4241	zinc finger protein 425
7653	YPEL3	-0.0288	0.4405	yippee-like 3 (Drosophila)
7654	KATNAL2	-0.0288	0.3517	katanin p60 subunit A-like 2
7655	HEATR3	-0.0288	0.5072	HEAT repeat containing 3
7656	ZNF638	-0.0292	0.5018	zinc finger protein 638
7657	OPTN	-0.0292	0.4764	optineurin
7658	MEF2C	-0.0292	0.4624	myocyte enhancer factor 2C
7659	GPBP1L1	-0.0292	0.5188	GC-rich promoter binding protein 1-like 1
7660	EIF1AY	-0.0292	0.4093	eukaryotic translation initiation factor 1A, Y-linked
7661	SUCLA2	-0.03	0.5003	succinate-CoA ligase, ADP-forming, beta subunit
7662	SLC2A4	-0.03	0.4303	solute carrier family 2 (facilitated glucose transporter), member 4
7663	SESTD1	-0.03	0.5085	SEC14 and spectrin domains 1
7664	sept-05	-0.03	0.433	septin 5
7665	LRRC34	-0.03	0.4631	leucine rich repeat containing 34
7666	HPS1	-0.03	0.4466	Hermansky-Pudlak syndrome 1
7667	FLJ20712	-0.03	0.4157	uncharacterized FLJ20712
7668	DPP8	-0.03	0.5259	dipeptidyl-peptidase 8
7669	CYP2A6	-0.03	0.4343	cytochrome P450, family 2, subfamily A, polypeptide 6
7670	CAPZA3	-0.03	0.308	capping protein (actin filament) muscle Z-line, alpha 3
7671	C7orf25	-0.03	0.5134	chromosome 7 open reading frame 25
7672	BVES	-0.03	0.4123	blood vessel epicardial substance
7673	ANO5	-0.03	0.4107	anoctamin 5
7674	TIPARP	-0.0308	0.4836	TCDD-inducible poly(ADP-ribose) polymerase
7675	TBX6	-0.0308	0.4341	T-box 6
7676	SNX17	-0.0308	0.4895	sorting nexin 17
7677	PDE4DIP	-0.0308	0.45	phosphodiesterase 4D interacting protein
7678	MTUS2	-0.0308	0.3938	microtubule associated tumor suppressor candidate 2
7679	GAP43	-0.0308	0.4068	growth associated protein 43
7680	FAM134B	-0.0308	0.4651	family with sequence similarity 134, member B
7681	CHRD	-0.0308	0.4389	chordin
7682	ATF2	-0.0308	0.4967	activating transcription factor 2
7683	TSPAN11	-0.0313	0.3838	tetraspanin 11
7684	TIGD7	-0.0313	0.4977	tigger transposable element derived 7
7685	PPTC7	-0.0313	0.4953	PTC7 protein phosphatase homolog (S. cerevisiae)
7686	CYP2R1	-0.0313	0.5184	cytochrome P450, family 2, subfamily R, polypeptide 1
7687	ACBD5	-0.0313	0.5131	acyl-CoA binding domain containing 5
7688	TREM2	-0.0317	0.4208	triggering receptor expressed on myeloid cells 2
7689	TNXB	-0.0317	0.4291	tenascin XB
7690	SH3GL1	-0.0317	0.4544	SH3-domain GRB2-like 1
7691	RARA	-0.0317	0.4552	retinoic acid receptor, alpha
7692	PPBP	-0.0317	0.323	pro-platelet basic protein (chemokine (C-X-C motif) ligand 7)
7693	PGK1	-0.0317	0.4909	phosphoglycerate kinase 1
7694	ODZ4	-0.0317	0.4079	odz, odd Oz/ten-m homolog 4 (Drosophila)
7695	NUDT15	-0.0317	0.4715	nudix (nucleoside diphosphate linked moiety X)-type motif 15
7696	LAG3	-0.0317	0.3402	lymphocyte-activation gene 3
7697	HMX1	-0.0317	0.4262	H6 family homeobox 1
7698	DIAPH2	-0.0317	0.5133	diaphanous homolog 2 (Drosophila)
7699	CEPT1	-0.0317	0.5088	choline/ethanolamine phosphotransferase 1
7700	ZNF254	-0.0325	0.4867	zinc finger protein 254
7701	SLC43A1	-0.0325	0.4123	solute carrier family 43, member 1
7702	SCAMP3	-0.0325	0.474	secretory carrier membrane protein 3
7703	RNF41	-0.0325	0.5181	ring finger protein 41
7704	NME5	-0.0325	0.418	NME/NM23 family member 5
7705	MYBPH	-0.0325	0.4234	myosin binding protein H
7706	MTCP1	-0.0325	0.412	mature T-cell proliferation 1
7707	HNRPLL	-0.0325	0.5222	heterogeneous nuclear ribonucleoprotein L-like
7708	HAO1	-0.0325	0.4039	hydroxyacid oxidase (glycolate oxidase) 1
7709	GFRA4	-0.0325	0.4143	GDNF family receptor alpha 4
7710	DDX56	-0.0325	0.4748	DEAD (Asp-Glu-Ala-Asp) box helicase 56
7711	BAX	-0.0325	0.424	BCL2-associated X protein
7712	MCTP2	-0.0329	0.4292	multiple C2 domains, transmembrane 2
7713	TMA16	-0.0333	0.498	translation machinery associated 16 homolog (S. cerevisiae)
7714	ST8SIA2	-0.0333	0.419	ST8 alpha-N-acetyl-neuraminide alpha-2,8-sialyltransferase 2
7715	PSD4	-0.0333	0.4352	pleckstrin and Sec7 domain containing 4
7716	NME4	-0.0333	0.4359	NME/NM23 nucleoside diphosphate kinase 4
7717	NGRN	-0.0333	0.5154	neugrin, neurite outgrowth associated
7718	MED21	-0.0333	0.5046	mediator complex subunit 21
7719	HTR3B	-0.0333	0.4213	5-hydroxytryptamine (serotonin) receptor 3B, ionotropic
7720	GOLGA8A	-0.0333	0.4687	golgin A8 family, member A
7721	FOXD1	-0.0333	0.329	forkhead box D1
7722	C10orf76	-0.0333	0.5272	chromosome 10 open reading frame 76
7723	BECN1	-0.0333	0.5171	beclin 1, autophagy related
7724	SDR42E1	-0.0338	0.3508	short chain dehydrogenase/reductase family 42E, member 1
7725	HDX	-0.0338	0.4809	highly divergent homeobox
7726	C16orf52	-0.0338	0.4893	chromosome 16 open reading frame 52

7727	ATP1A1OS	-0.0338	0.4105	ATP1A1 opposite strand
7728	SPP2	-0.0342	0.4051	secreted phosphoprotein 2, 24kDa
7729	PSG5	-0.0342	0.3849	pregnancy specific beta-1-glycoprotein 5
7730	PIGP	-0.0342	0.4777	phosphatidylinositol glycan anchor biosynthesis, class P
7731	MX1	-0.0342	0.3993	myxovirus (influenza virus) resistance 1, interferon-inducible protein p78 (mouse)
7732	MOCS3	-0.0342	0.4667	molybdenum cofactor synthesis 3
7733	ELF4	-0.0342	0.464	E74-like factor 4 (ets domain transcription factor)
7734	DRP2	-0.0342	0.4282	dystrophin related protein 2
7735	LINC00324	-0.0343	0.3618	long intergenic non-protein coding RNA 324
7736	TUBBP5	-0.0345	0.306	tubulin, beta pseudogene 5
7737	ZNF440	-0.035	0.4989	zinc finger protein 440
7738	RALGAPA1	-0.035	0.5055	Ral GTPase activating protein, alpha subunit 1 (catalytic)
7739	RAB11FIP1	-0.035	0.4605	RAB11 family interacting protein 1 (class I)
7740	PI4K2B	-0.035	0.502	phosphatidylinositol 4-kinase type 2 beta
7741	KLHL35	-0.035	0.3931	kelch-like 35 (Drosophila)
7742	IRAK2	-0.035	0.3766	interleukin-1 receptor-associated kinase 2
7743	CLDN17	-0.035	0.4178	claudin 17
7744	CIDEB	-0.035	0.4362	cell death-inducing DFFA-like effector b
7745	C2orf47	-0.035	0.496	chromosome 2 open reading frame 47
7746	SHARPIN	-0.0358	0.4468	SHANK-associated RH domain interactor
7747	NPDC1	-0.0358	0.426	neural proliferation, differentiation and control, 1
7748	EIF3A	-0.0358	0.4991	eukaryotic translation initiation factor 3, subunit A
7749	B3GALT2	-0.0358	0.4	UDP-Gal:betaGlcNAc beta 1,3-galactosyltransferase, polypeptide 2
7750	XRN1	-0.0362	0.4972	5'-3' exoribonuclease 1
7751	C1orf86	-0.0362	0.3992	chromosome 1 open reading frame 86
7752	TRIM48	-0.0367	0.3914	tripartite motif containing 48
7753	MLXIP	-0.0367	0.4158	MLX interacting protein
7754	MAPK8	-0.0367	0.4771	mitogen-activated protein kinase 8
7755	HEY2	-0.0367	0.3903	hairy/enhancer-of-split related with YRPW motif 2
7756	FOXC2	-0.0367	0.4314	forkhead box C2 (MFH-1, mesenchyme forkhead 1)
7757	AGPAT3	-0.0367	0.4704	1-acylglycerol-3-phosphate O-acyltransferase 3
7758	ITPK1-AS1	-0.0371	0	ITPK1 antisense RNA 1 (non-protein coding)
7759	XCL1	-0.0375	0.397	chemokine (C motif) ligand 1
7760	TCL1B	-0.0375	0.4162	T-cell leukemia/lymphoma 1B
7761	SPCS1	-0.0375	0.5013	signal peptidase complex subunit 1 homolog (S. cerevisiae)
7762	SCN5A	-0.0375	0.4174	sodium channel, voltage-gated, type V, alpha subunit
7763	PRRC2C	-0.0375	0.4816	proline-rich coiled-coil 2C
7764	PBX4	-0.0375	0.3417	pre-B-cell leukemia homeobox 4
7765	INTS6	-0.0375	0.4978	integrator complex subunit 6
7766	GOT2	-0.0375	0.4947	glutamic-oxaloacetic transaminase 2, mitochondrial (aspartate aminotransferase 2)
7767	FOXK1	-0.0375	0.4328	forkhead box K1
7768	FGFBP3	-0.0375	0.3114	fibroblast growth factor binding protein 3
7769	FBXL14	-0.0375	0.3852	F-box and leucine-rich repeat protein 14
7770	ETFA	-0.0375	0.4974	electron-transfer-flavoprotein, alpha polypeptide
7771	DDX17	-0.0375	0.4774	DEAD (Asp-Glu-Ala-Asp) box helicase 17
7772	REG1P	-0.0383	0.4255	regenerating islet-derived 1 pseudogene
7773	NINL	-0.0383	0.3889	ninein-like
7774	KCNA10	-0.0383	0.4039	potassium voltage-gated channel, shaker-related subfamily, member 10
7775	ICA1	-0.0383	0.418	islet cell autoantigen 1, 69kDa
7776	EPHB2	-0.0383	0.4645	EPH receptor B2
7777	ARHGAP17	-0.0383	0.5109	Rho GTPase activating protein 17
7778	PLEKHM3	-0.0388	0.5027	pleckstrin homology domain containing, family M, member 3
7779	KIAA1549	-0.0388	0.4038	KIAA1549
7780	CDKN2B-AS1	-0.0391	0.4261	CDKN2B antisense RNA 1 (non-protein coding)
7781	PPEF1	-0.0392	0.4266	protein phosphatase, EF-hand calcium binding domain 1
7782	MARCH2	-0.0392	0.4566	membrane-associated ring finger (C3HC4) 2, E3 ubiquitin protein ligase
7783	HSD17B11	-0.0392	0.4943	hydroxysteroid (17-beta) dehydrogenase 11
7784	FANCF	-0.0392	0.5145	Fanconi anemia, complementation group F
7785	C12orf44	-0.0392	0.4691	chromosome 12 open reading frame 44
7786	ZZZ3	-0.04	0.5113	zinc finger, ZZ-type containing 3
7787	SLC23A3	-0.04	0.3841	solute carrier family 23 (nucleobase transporters), member 3
7788	PVRL1	-0.04	0.4659	poliovirus receptor-related 1 (herpesvirus entry mediator C)
7789	PGM2	-0.04	0.5112	phosphoglucomutase 2
7790	MAGEC3	-0.04	0.4053	melanoma antigen family C, 3
7791	LOC728606	-0.04	0.3495	uncharacterized LOC728606
7792	LOC400456	-0.04	0.3636	uncharacterized LOC400456
7793	LALBA	-0.04	0.4024	lactalbumin, alpha-
7794	L3MBTL4	-0.04	0.3754	l(3)mbt-like 4 (Drosophila)
7795	KIAA1328	-0.04	0.4249	KIAA1328
7796	KCTD21	-0.04	0.4199	potassium channel tetramerisation domain containing 21
7797	ING3	-0.04	0.4986	inhibitor of growth family, member 3
7798	HCG9	-0.04	0.4299	HLA complex group 9 (non-protein coding)
7799	GCGR	-0.04	0.4228	glucagon receptor
7800	C19orf66	-0.04	0.4253	chromosome 19 open reading frame 66
7801	C12orf66	-0.04	0.5073	chromosome 12 open reading frame 66
7802	ADCY3	-0.04	0.4534	adenylate cyclase 3
7803	ACTN3	-0.04	0.433	actinin, alpha 3
7804	TREM1	-0.0408	0.4285	triggering receptor expressed on myeloid cells 1
7805	SOX10	-0.0408	0.4392	SRY (sex determining region Y)-box 10
7806	SNCAIP	-0.0408	0.4345	synuclein, alpha interacting protein
7807	RAB11FIP2	-0.0408	0.5066	RAB11 family interacting protein 2 (class I)
7808	PLCL1	-0.0408	0.4563	phospholipase C-like 1
7809	LY6G6E	-0.0408	0.4005	lymphocyte antigen 6 complex, locus G6E (pseudogene)
7810	IFT57	-0.0408	0.5088	intraflagellar transport 57 homolog (Chlamydomonas)

7811	GTDC1	-0.0408	0.5176	glycosyltransferase-like domain containing 1
7812	CPLX3	-0.0408	0.4261	complexin 3
7813	CARD9	-0.0408	0.3872	caspase recruitment domain family, member 9
7814	TMEM86A	-0.0413	0.4479	transmembrane protein 86A
7815	KBTBD5	-0.0413	0.37	kelch repeat and BTB (POZ) domain containing 5
7816	FMNL3	-0.0413	0.437	formin-like 3
7817	EGLN2	-0.0414	0.4442	egl nine homolog 2 (C. elegans)
7818	DEFB121	-0.0414	0.3756	defensin, beta 121
7819	VRK2	-0.0417	0.5085	vaccinia related kinase 2
7820	RNF19A	-0.0417	0.4936	ring finger protein 19A, E3 ubiquitin protein ligase
7821	POLR3B	-0.0417	0.5075	polymerase (RNA) III (DNA directed) polypeptide B
7822	HADHB	-0.0417	0.517	hydroxyacyl-CoA dehydrogenase/3-ketoacyl-CoA thiolase/enoyl-CoA hydratase (trifunctional protein), beta subunit
7823	GNA15	-0.0417	0.405	guanine nucleotide binding protein (G protein), alpha 15 (Gq class)
7824	FLI1	-0.0417	0.4539	Friend leukemia virus integration 1
7825	FGF6	-0.0417	0.4267	fibroblast growth factor 6
7826	FGA	-0.0417	0.4087	fibrinogen alpha chain
7827	CDC42BPB	-0.0417	0.4824	CDC42 binding protein kinase beta (DMPK-like)
7828	BAI3	-0.0417	0.3992	brain-specific angiogenesis inhibitor 3
7829	SPINK7	-0.0425	0.3663	serine peptidase inhibitor, Kazal type 7 (putative)
7830	SOCS2	-0.0425	0.4848	suppressor of cytokine signaling 2
7831	SMPD2	-0.0425	0.4555	sphingomyelin phosphodiesterase 2, neutral membrane (neutral sphingomyelinase)
7832	SESN1	-0.0425	0.5092	sestrin 1
7833	MX2	-0.0425	0.4023	myxovirus (influenza virus) resistance 2 (mouse)
7834	MLLT10	-0.0425	0.5094	myeloid/lymphoid or mixed-lineage leukemia (trithorax homolog, Drosophila); translocated to, 10
7835	MARCKS	-0.0425	0.4864	myristoylated alanine-rich protein kinase C substrate
7836	MAGI1	-0.0425	0.4575	membrane associated guanylate kinase, WW and PDZ domain containing 1
7837	KLF13	-0.0425	0.476	Kruppel-like factor 13
7838	FERMT3	-0.0425	0.3903	fermitin family member 3
7839	EPHB1	-0.0425	0.4391	EPH receptor B1
7840	EMC2	-0.0425	0.5077	ER membrane protein complex subunit 2
7841	CPEB4	-0.0425	0.4915	cytoplasmic polyadenylation element binding protein 4
7842	ARHGAP10	-0.0425	0.503	Rho GTPase activating protein 10
7843	SPINT3	-0.0427	0.4301	serine peptidase inhibitor, Kunitz type, 3
7844	UGT2B28	-0.0429	0.363	UDP glucuronosyltransferase 2 family, polypeptide B28
7845	C10orf71	-0.0429	0.3895	chromosome 10 open reading frame 71
7846	ZNF197	-0.0433	0.5004	zinc finger protein 197
7847	RRN3P3	-0.0433	0	RNA polymerase I transcription factor homolog (S. cerevisiae) pseudogene 3
7848	PPM1F	-0.0433	0.4228	protein phosphatase, Mg2+/Mn2+ dependent, 1F
7849	GNAT1	-0.0433	0.4309	guanine nucleotide binding protein (G protein), alpha transducing activity polypeptide 1
7850	C5AR1	-0.0433	0.3848	complement component 5a receptor 1
7851	BMP15	-0.0433	0.4182	bone morphogenetic protein 15
7852	BCL11A	-0.0433	0.4249	B-cell CLL/lymphoma 11A (zinc finger protein)
7853	ALOX5	-0.0433	0.3941	arachidonate 5-lipoxygenase
7854	MORF4L1	-0.0436	0.5032	mortality factor 4 like 1
7855	WNT10A	-0.0437	0.3958	wingless-type MMTV integration site family, member 10A
7856	VPS18	-0.0437	0.469	vacuolar protein sorting 18 homolog (S. cerevisiae)
7857	RDH13	-0.0437	0.4313	retinol dehydrogenase 13 (all-trans/9-cis)
7858	LRRC27	-0.0437	0.4204	leucine rich repeat containing 27
7859	FITM2	-0.0437	0.4333	fat storage-inducing transmembrane protein 2
7860	CXCL17	-0.0437	0.3914	chemokine (C-X-C motif) ligand 17
7861	USP12	-0.0442	0.4246	ubiquitin specific peptidase 12
7862	TMEM231	-0.0442	0.4771	transmembrane protein 231
7863	SLC6A8	-0.0442	0.4078	solute carrier family 6 (neurotransmitter transporter, creatine), member 8
7864	SLC6A3	-0.0442	0.4264	solute carrier family 6 (neurotransmitter transporter, dopamine), member 3
7865	SLC2A3	-0.0442	0.4488	solute carrier family 2 (facilitated glucose transporter), member 3
7866	RHO	-0.0442	0.4311	rhodopsin
7867	NTM	-0.0442	0.4174	neurotrimin
7868	MTMR8	-0.0442	0.4491	myotubularin related protein 8
7869	HUNK	-0.0442	0.4399	hormonally up-regulated Neu-associated kinase
7870	GPR3	-0.0442	0.4333	G protein-coupled receptor 3
7871	ADCY7	-0.0442	0.4698	adenylate cyclase 7
7872	PTCHD4	-0.0443	0.3895	patched domain containing 4
7873	TFB2M	-0.045	0.4986	transcription factor B2, mitochondrial
7874	STX1A	-0.045	0.4438	syntaxin 1A (brain)
7875	SETD1B	-0.045	0.4125	SET domain containing 1B
7876	RNFT1	-0.045	0.5097	ring finger protein, transmembrane 1
7877	RFWD2	-0.045	0.5348	ring finger and WD repeat domain 2, E3 ubiquitin protein ligase
7878	PEX3	-0.045	0.5079	peroxisomal biogenesis factor 3
7879	OR3A2	-0.045	0.4248	olfactory receptor, family 3, subfamily A, member 2
7880	MLYCD	-0.045	0.4941	malonyl-CoA decarboxylase
7881	FAM48A	-0.045	0.5236	family with sequence similarity 48, member A
7882	CYP11B2	-0.045	0.4153	cytochrome P450, family 11, subfamily B, polypeptide 2
7883	CXCR3	-0.045	0.4325	chemokine (C-X-C motif) receptor 3
7884	CWC25	-0.045	0.4648	CWC25 spliceosome-associated protein homolog (S. cerevisiae)
7885	CCL23	-0.045	0.4018	chemokine (C-C motif) ligand 23
7886	ANKMY2	-0.045	0.5293	ankyrin repeat and MYND domain containing 2
7887	SKAP1	-0.0458	0.3883	src kinase associated phosphoprotein 1
7888	POLR3F	-0.0458	0.5211	polymerase (RNA) III (DNA directed) polypeptide F, 39 kDa
7889	MICAL2	-0.0458	0.4507	microtubule associated monoxygenase, calponin and LIM domain containing 2
7890	MEP1B	-0.0458	0.3999	meprin A, beta
7891	BAG2	-0.0458	0.5019	BCL2-associated athanogene 2
7892	SLC15A4	-0.0463	0.5248	solute carrier family 15, member 4
7893	SENP8	-0.0463	0.4842	SUMO/sentrin specific peptidase family member 8
7894	ZNF573	-0.0467	0.4957	zinc finger protein 573

7895	ZNF286A	-0.0467	0.4943	zinc finger protein 286A
7896	ZNF16	-0.0467	0.4377	zinc finger protein 16
7897	SAMHD1	-0.0467	0.5018	SAM domain and HD domain 1
7898	RREB1	-0.0467	0.4448	ras responsive element binding protein 1
7899	PLEKHF2	-0.0467	0.5095	pleckstrin homology domain containing, family F (with FYVE domain) member 2
7900	MAML1	-0.0467	0.4705	mastermind-like 1 (Drosophila)
7901	MAL	-0.0467	0.3895	mal, T-cell differentiation protein
7902	FNBP4	-0.0467	0.4886	formin binding protein 4
7903	ENSA	-0.0467	0.4953	endosulfine alpha
7904	DLX4	-0.0467	0.4322	distal-less homeobox 4
7905	CACNB1	-0.0467	0.465	calcium channel, voltage-dependent, beta 1 subunit
7906	CDRT1	-0.0471	0.3607	CMT1A duplicated region transcript 1
7907	ZNF248	-0.0475	0.5139	zinc finger protein 248
7908	UPB1	-0.0475	0.4297	ureidopropionase, beta
7909	PNISR	-0.0475	0.4889	PNN-interacting serine/arginine-rich protein
7910	NOP14-AS1	-0.0475	0.4455	NOP14 antisense RNA 1 (non-protein coding)
7911	MAGEB18	-0.0475	0.228	melanoma antigen family B, 18
7912	FAN1	-0.0475	0.5177	FANCD2/FANCI-associated nuclease 1
7913	F12	-0.0475	0.4095	coagulation factor XII (Hageman factor)
7914	DCPS	-0.0475	0.4885	decapping enzyme, scavenger
7915	CENPBD1	-0.0475	0.527	CENPB DNA-binding domains containing 1
7916	C1orf222	-0.0482	0.4391	chromosome 1 open reading frame 222
7917	UPK2	-0.0483	0.4283	uroplakin 2
7918	NUP98	-0.0483	0.5172	nucleoporin 98kDa
7919	IFIT1	-0.0483	0.4791	interferon-induced protein with tetratricopeptide repeats 1
7920	DUSP4	-0.0483	0.3748	dual specificity phosphatase 4
7921	SMTNL1	-0.0486	0.4128	smoothelin-like 1
7922	ZNF600	-0.0487	0.5057	zinc finger protein 600
7923	UBXN10	-0.0487	0.395	UBX domain protein 10
7924	SIN3A	-0.0487	0.5348	SIN3 transcription regulator homolog A (yeast)
7925	RIMKLB	-0.0487	0.5062	ribosomal modification protein rimK-like family member B
7926	FAM83A	-0.0487	0.4181	family with sequence similarity 83, member A
7927	FAM160A2	-0.0487	0.4366	family with sequence similarity 160, member A2
7928	COPG2	-0.0487	0.5391	coatomer protein complex, subunit gamma 2
7929	TMEM104	-0.0492	0.4809	transmembrane protein 104
7930	SORL1	-0.0492	0.4744	sortilin-related receptor, L(DLR class) A repeats containing
7931	PPIL2	-0.0492	0.4935	peptidylprolyl isomerase (cyclophilin)-like 2
7932	OSBPL8	-0.0492	0.5113	oxysterol binding protein-like 8
7933	LYST	-0.0492	0.4849	lysosomal trafficking regulator
7934	LHPP	-0.0492	0.4672	phospholysine phosphohistidine inorganic pyrophosphate phosphatase
7935	DUSP2	-0.0492	0.3683	dual specificity phosphatase 2
7936	CNOT1	-0.0492	0.5239	CCR4-NOT transcription complex, subunit 1
7937	APEX1	-0.0492	0.5021	APEX nuclease (multifunctional DNA repair enzyme) 1
7938	AES	-0.0492	0.478	amino-terminal enhancer of split
7939	TROVE2	-0.05	0.5194	TROVE domain family, member 2
7940	STPG2	-0.05	0.3909	sperm-tail PG-rich repeat containing 2
7941	SREK1IP1	-0.05	0.5216	SREK1-interacting protein 1
7942	RUNX1-IT1	-0.05	0.4333	RUNX1 intronic transcript 1 (non-protein coding)
7943	RHOU	-0.05	0.4584	ras homolog family member U
7944	PTGDR	-0.05	0.4103	prostaglandin D2 receptor (DP)
7945	MUC12	-0.05	0.3874	mucin 12, cell surface associated
7946	LYPD5	-0.05	0.4134	LY6/PLAUR domain containing 5
7947	KCNJ12	-0.05	0.4238	potassium inwardly-rectifying channel, subfamily J, member 12
7948	HNF1A	-0.05	0.4327	HNF1 homeobox A
7949	ERCC4	-0.05	0.5153	excision repair cross-complementing rodent repair deficiency, complementation group 4
7950	CTAGE11P	-0.05	0.4581	CTAGE family, member 11, pseudogene
7951	C9	-0.05	0.3635	complement component 9
7952	ZNF767	-0.0508	0.4481	zinc finger family member 767
7953	ZNF354A	-0.0508	0.5159	zinc finger protein 354A
7954	WNT4	-0.0508	0.44	wingless-type MMTV integration site family, member 4
7955	TSPAN7	-0.0508	0.4437	tetraspanin 7
7956	PILRB	-0.0508	0.4629	paired immunoglobin-like type 2 receptor beta
7957	PEA15	-0.0508	0.5053	phosphoprotein enriched in astrocytes 15
7958	MTUS1	-0.0508	0.4907	microtubule associated tumor suppressor 1
7959	CSNK1D	-0.0508	0.4944	casein kinase 1, delta
7960	CA9	-0.0508	0.4312	carbonic anhydrase IX
7961	C17orf76-AS1	-0.0512	0.4384	C17orf76 antisense RNA 1 (non-protein coding)
7962	FAM66C	-0.0514	0	family with sequence similarity 66, member C
7963	ZNF329	-0.0517	0.5249	zinc finger protein 329
7964	TOR1A	-0.0517	0.5227	torsin family 1, member A (torsin A)
7965	RNF11	-0.0517	0.4922	ring finger protein 11
7966	RHOT2	-0.0517	0.4573	ras homolog family member T2
7967	PORCN	-0.0517	0.4643	porcupine homolog (Drosophila)
7968	NPVF	-0.0517	0.4045	neuropeptide VF precursor
7969	MAP1B	-0.0517	0.455	microtubule-associated protein 1B
7970	LEPRE1	-0.0517	0.4888	leucine proline-enriched proteoglycan (leprecan) 1
7971	TRUB1	-0.0525	0.5158	TruB pseudouridine (psi) synthase homolog 1 (E. coli)
7972	SERPINA4	-0.0525	0.4355	serpin peptidase inhibitor, clade A (alpha-1 antiproteinase, antitrypsin), member 4
7973	RASL12	-0.0525	0.4239	RAS-like, family 12
7974	PRPF39	-0.0525	0.5087	PRP39 pre-mRNA processing factor 39 homolog (S. cerevisiae)
7975	NT5C1A	-0.0525	0.3964	5'-nucleotidase, cytosolic IA
7976	MBLAC2	-0.0525	0.507	metallo-beta-lactamase domain containing 2
7977	LINC00310	-0.0525	0.3793	long intergenic non-protein coding RNA 310
7978	IRF2BP1	-0.0525	0.4621	interferon regulatory factor 2 binding protein 1

7979	INE1	-0.0525	0.4488	inactivation escape 1 (non-protein coding)
7980	HSD17B6	-0.0525	0.4807	hydroxysteroid (17-beta) dehydrogenase 6 homolog (mouse)
7981	EIF3K	-0.0525	0.4831	eukaryotic translation initiation factor 3, subunit K
7982	CYP2A13	-0.0525	0.4353	cytochrome P450, family 2, subfamily A, polypeptide 13
7983	BCL2L13	-0.0525	0.5137	BCL2-like 13 (apoptosis facilitator)
7984	BCHE	-0.0525	0.4441	butyrylcholinesterase
7985	ASCC2	-0.0525	0.4902	activating signal cointegrator 1 complex subunit 2
7986	RFPL3	-0.0533	0.4293	ret finger protein-like 3
7987	RASGRP3	-0.0533	0.4659	RAS guanyl releasing protein 3 (calcium and DAG-regulated)
7988	PTPRS	-0.0533	0.4693	protein tyrosine phosphatase, receptor type, S
7989	PRDM14	-0.0533	0.4144	PR domain containing 14
7990	PJA2	-0.0533	0.5092	praja ring finger 2, E3 ubiquitin protein ligase
7991	OPRM1	-0.0533	0.4192	opioid receptor, mu 1
7992	MYBPC1	-0.0533	0.3996	myosin binding protein C, slow type
7993	MAP3K7	-0.0533	0.5175	mitogen-activated protein kinase kinase kinase 7
7994	LATS1	-0.0533	0.4847	LATS, large tumor suppressor, homolog 1 (Drosophila)
7995	IRF4	-0.0533	0.4333	interferon regulatory factor 4
7996	GDI2	-0.0533	0.513	GDP dissociation inhibitor 2
7997	FUT7	-0.0533	0.4412	fucosyltransferase 7 (alpha (1,3) fucosyltransferase)
7998	ERCC2	-0.0533	0.4711	excision repair cross-complementing rodent repair deficiency, complementation group 2
7999	FAM167B	-0.0538	0.4283	family with sequence similarity 167, member B
8000	ZNF26	-0.0542	0.5042	zinc finger protein 26
8001	TMEM140	-0.0542	0.4459	transmembrane protein 140
8002	SYT13	-0.0542	0.4363	synaptotagmin XIII
8003	PPP2CB	-0.0542	0.5238	protein phosphatase 2, catalytic subunit, beta isozyme
8004	POMT2	-0.0542	0.4931	protein-O-mannosyltransferase 2
8005	LARP1	-0.0542	0.5029	La ribonucleoprotein domain family, member 1
8006	GSTA1	-0.0542	0.4043	glutathione S-transferase alpha 1
8007	GRP	-0.0542	0.4299	gastrin-releasing peptide
8008	FJX1	-0.0542	0.4374	four jointed box 1 (Drosophila)
8009	DYRK2	-0.0542	0.5014	dual-specificity tyrosine-(Y)-phosphorylation regulated kinase 2
8010	TCF25	-0.055	0.4993	transcription factor 25 (basic helix-loop-helix)
8011	RABEP1	-0.055	0.5287	rabaptin, RAB GTPase binding effector protein 1
8012	POLD3	-0.055	0.5083	polymerase (DNA-directed), delta 3, accessory subunit
8013	LHFPL1	-0.055	0.4015	lipoma HMGIC fusion partner-like 1
8014	HES7	-0.055	0.3997	hairy and enhancer of split 7 (Drosophila)
8015	ENDOV	-0.055	0.4391	endonuclease V
8016	BPIFB1	-0.055	0.38	BPI fold containing family B, member 1
8017	ADAM8	-0.055	0.4337	ADAM metallopeptidase domain 8
8018	SERPINA2	-0.0555	0.425	serpin peptidase inhibitor, clade A (alpha-1 antiproteinase, antitrypsin), member 2
8019	TBCE	-0.0558	0.5166	tubulin folding cofactor E
8020	SHC2	-0.0558	0.4604	SHC (Src homology 2 domain containing) transforming protein 2
8021	SH3TC2	-0.0558	0.4191	SH3 domain and tetratricopeptide repeats 2
8022	RNF141	-0.0558	0.5199	ring finger protein 141
8023	PARG	-0.0558	0.5189	poly (ADP-ribose) glycohydrolase
8024	IFT81	-0.0558	0.5186	intraflagellar transport 81 homolog (Chlamydomonas)
8025	HRC	-0.0558	0.4343	histidine rich calcium binding protein
8026	GVINP1	-0.0558	0.4131	GTPase, very large interferon inducible pseudogene 1
8027	FDX1	-0.0558	0.4921	ferredoxin 1
8028	DUS4L	-0.0558	0.5044	dihydrouridine synthase 4-like (S. cerevisiae)
8029	ADAMDEC1	-0.0558	0.3773	ADAM-like, decysin 1
8030	RFT1	-0.0562	0.5187	RFT1 homolog (S. cerevisiae)
8031	KRT82	-0.0562	0.3996	keratin 82
8032	FAM83F	-0.0562	0.4094	family with sequence similarity 83, member F
8033	USP27X	-0.0567	0.4555	ubiquitin specific peptidase 27, X-linked
8034	STAT5B	-0.0567	0.4919	signal transducer and activator of transcription 5B
8035	PSTPIP2	-0.0567	0.5126	proline-serine-threonine phosphatase interacting protein 2
8036	NR0B2	-0.0567	0.4341	nuclear receptor subfamily 0, group B, member 2
8037	MT1M	-0.0567	0.3913	metallothionein 1M
8038	EPN2	-0.0567	0.477	epsin 2
8039	C16orf7	-0.0567	0.4592	chromosome 16 open reading frame 7
8040	BPTF	-0.0567	0.5118	bromodomain PHD finger transcription factor
8041	BIRC7	-0.0567	0.4186	baculoviral IAP repeat containing 7
8042	APH1B	-0.0567	0.4955	anterior pharynx defective 1 homolog B (C. elegans)
8043	ZNF76	-0.0575	0.4747	zinc finger protein 76
8044	PCF11	-0.0575	0.5108	PCF11, cleavage and polyadenylation factor subunit, homolog (S. cerevisiae)
8045	PCBD2	-0.0575	0.5033	pterin-4 alpha-carbinolamine dehydratase/dimerization cofactor of hepatocyte nuclear factor 1 alpha (TCF1) 2
8046	LOC155060	-0.0575	0.4327	AI894139 pseudogene
8047	KBTBD2	-0.0575	0.5222	kelch repeat and BTB (POZ) domain containing 2
8048	IL5	-0.0575	0.4171	interleukin 5 (colony-stimulating factor, eosinophil)
8049	IL2RB	-0.0575	0.3955	interleukin 2 receptor, beta
8050	HEXIM1	-0.0575	0.5285	hexamethylene bis-acetamide inducible 1
8051	FZD9	-0.0575	0.439	frizzled family receptor 9
8052	EIF3D	-0.0575	0.5063	eukaryotic translation initiation factor 3, subunit D
8053	C14orf1	-0.0575	0.5182	chromosome 14 open reading frame 1
8054	ATAD3C	-0.0575	0.4001	ATPase family, AAA domain containing 3C
8055	AKAP11	-0.0575	0.5167	A kinase (PRKA) anchor protein 11
8056	AKAP1	-0.0575	0.4998	A kinase (PRKA) anchor protein 1
8057	ZP2	-0.0583	0.4124	zona pellucida glycoprotein 2 (sperm receptor)
8058	WDR6	-0.0583	0.4891	WD repeat domain 6
8059	TRPC2	-0.0583	0.4268	transient receptor potential cation channel, subfamily C, member 2, pseudogene
8060	SH3BGR	-0.0583	0.4439	SH3 domain binding glutamic acid-rich protein
8061	RECQL5	-0.0583	0.471	RecQ protein-like 5
8062	OBP2A	-0.0583	0.4372	odorant binding protein 2A

8063	NAP1L3	-0.0583	0.4935	nucleosome assembly protein 1-like 3
8064	MRS2	-0.0583	0.5114	MRS2 magnesium homeostasis factor homolog (S. cerevisiae)
8065	LOR	-0.0583	0.4287	loricrin
8066	HMBOX1	-0.0583	0.4926	homeobox containing 1
8067	C11orf95	-0.0583	0.3675	chromosome 11 open reading frame 95
8068	ZNF420	-0.0587	0.5272	zinc finger protein 420
8069	TATDN3	-0.0587	0.5078	TatD DNase domain containing 3
8070	SCARF2	-0.0587	0.4351	scavenger receptor class F, member 2
8071	PGM2L1	-0.0587	0.5186	phosphoglucomutase 2-like 1
8072	LOC284440	-0.0587	0.4156	uncharacterized LOC284440
8073	ENKUR	-0.0587	0.3929	enkurin, TRPC channel interacting protein
8074	CPO	-0.0587	0.3829	carboxypeptidase O
8075	CHST9	-0.0587	0.3768	carbohydrate (N-acetylgalactosamine 4-0) sulfotransferase 9
8076	ANAPC7	-0.0587	0.5273	anaphase promoting complex subunit 7
8077	WASF2	-0.0589	0.4911	WAS protein family, member 2
8078	VCPIP1	-0.0592	0.4956	valosin containing protein (p97)/p47 complex interacting protein 1
8079	SH3BP5	-0.0592	0.499	SH3-domain binding protein 5 (BTK-associated)
8080	RUNX1	-0.0592	0.4743	runt-related transcription factor 1
8081	RING1	-0.0592	0.4796	ring finger protein 1
8082	MTMR2	-0.0592	0.5216	myotubularin related protein 2
8083	FLAD1	-0.0592	0.4868	FAD1 flavin adenine dinucleotide synthetase homolog (S. cerevisiae)
8084	ARL2BP	-0.0592	0.5317	ADP-ribosylation factor-like 2 binding protein
8085	ARHGEF11	-0.0592	0.4885	Rho guanine nucleotide exchange factor (GEF) 11
8086	RPGR	-0.06	0.4981	retinitis pigmentosa GTPase regulator
8087	MAPRE1	-0.06	0.5106	microtubule-associated protein, RP/EB family, member 1
8088	LECT2	-0.06	0.4088	leukocyte cell-derived chemotaxin 2
8089	KCNMB4	-0.06	0.4564	potassium large conductance calcium-activated channel, subfamily M, beta member 4
8090	HMGXB3	-0.06	0.4907	HMG box domain containing 3
8091	FLOT2	-0.06	0.4751	flotillin 2
8092	C1orf174	-0.06	0.5125	chromosome 1 open reading frame 174
8093	C17orf59	-0.06	0.4707	chromosome 17 open reading frame 59
8094	ZMYM4	-0.0608	0.5284	zinc finger, MYM-type 4
8095	UBC	-0.0608	0.5111	ubiquitin C
8096	REV1	-0.0608	0.5194	REV1, polymerase (DNA directed)
8097	LRRC41	-0.0608	0.5085	leucine rich repeat containing 41
8098	LIPF	-0.0608	0.4341	lipase, gastric
8099	EGFL6	-0.0608	0.3984	EGF-like-domain, multiple 6
8100	DYNC1H1	-0.0608	0.5297	dynein, cytoplasmic 1, heavy chain 1
8101	CNNM4	-0.0608	0.4816	cyclin M4
8102	CCRL2	-0.0608	0.4425	chemokine (C-C motif) receptor-like 2
8103	CCR3	-0.0608	0.4283	chemokine (C-C motif) receptor 3
8104	SPDYE1	-0.0613	0.3143	speedy homolog E1 (Xenopus laevis)
8105	NOB1	-0.0613	0.5044	NIN1/RPN12 binding protein 1 homolog (S. cerevisiae)
8106	NBPF3	-0.0613	0.4531	neuroblastoma breakpoint family, member 3
8107	UQCRH	-0.0617	0.4684	ubiquinol-cytochrome c reductase hinge protein
8108	RAB9B	-0.0617	0.3774	RAB9B, member RAS oncogene family
8109	PDK3	-0.0617	0.5156	pyruvate dehydrogenase kinase, isozyme 3
8110	LSM5	-0.0617	0.4991	LSM5 homolog, U6 small nuclear RNA associated (S. cerevisiae)
8111	KCNH6	-0.0617	0.434	potassium voltage-gated channel, subfamily H (eag-related), member 6
8112	GSDMB	-0.0617	0.4483	gasdermin B
8113	FOCAD	-0.0617	0.5335	focadhesin
8114	CENPB	-0.0617	0.4517	centromere protein B, 80kDa
8115	ASCC3	-0.0617	0.5164	activating signal cointegrator 1 complex subunit 3
8116	ARHGAP22	-0.0617	0.4447	Rho GTPase activating protein 22
8117	ZNF649	-0.0625	0.5131	zinc finger protein 649
8118	ZNF136	-0.0625	0.5091	zinc finger protein 136
8119	ZBTB22	-0.0625	0.4612	zinc finger and BTB domain containing 22
8120	SUGT1	-0.0625	0.5188	SGT1, suppressor of G2 allele of SKP1 (S. cerevisiae)
8121	MSX1	-0.0625	0.3898	msh homeobox 1
8122	MAP3K12	-0.0625	0.4975	mitogen-activated protein kinase kinase kinase 12
8123	LOC375295	-0.0629	0.3679	uncharacterized LOC375295
8124	SAP18	-0.0633	0.5157	Sin3A-associated protein, 18kDa
8125	QRSL1	-0.0633	0.526	glutaminyl-tRNA synthase (glutamine-hydrolyzing)-like 1
8126	PANX1	-0.0633	0.5202	pannexin 1
8127	NAT1	-0.0633	0.4961	N-acetyltransferase 1 (arylamine N-acetyltransferase)
8128	KIAA0182	-0.0633	0.4905	KIAA0182
8129	GID8	-0.0633	0.5271	GID complex subunit 8 homolog (S. cerevisiae)
8130	ATP1B3	-0.0633	0.5116	ATPase, Na+/K+ transporting, beta 3 polypeptide
8131	POLR3GL	-0.0637	0.53	polymerase (RNA) III (DNA directed) polypeptide G (32kD)-like
8132	CCDC132	-0.0637	0.5321	coiled-coil domain containing 132
8133	ZFP37	-0.0642	0.5069	zinc finger protein 37 homolog (mouse)
8134	SETD5	-0.0642	0.4979	SET domain containing 5
8135	RS1	-0.0642	0.4348	retinoschisin 1
8136	RALBP1	-0.0642	0.5361	ralA binding protein 1
8137	MXI1	-0.0642	0.4637	MAX interactor 1
8138	CRYBB2	-0.0642	0.4277	crystallin, beta B2
8139	CHIC2	-0.0642	0.5108	cysteine-rich hydrophobic domain 2
8140	CCND3	-0.0642	0.4832	cyclin D3
8141	C8G	-0.0642	0.4131	complement component 8, gamma polypeptide
8142	GCNT7	-0.0643	0.3891	glucosaminyl (N-acetyl) transferase family member 7
8143	CDK11B	-0.0643	0.4328	cyclin-dependent kinase 11B
8144	WDR70	-0.065	0.5374	WD repeat domain 70
8145	UCHL3	-0.065	0.5016	ubiquitin carboxyl-terminal esterase L3 (ubiquitin thiolesterase)
8146	TNFRSF13B	-0.065	0.4387	tumor necrosis factor receptor superfamily, member 13B

8147	RPRD1B	-0.065	0.514	regulation of nuclear pre-mRNA domain containing 1B
8148	RHOT1	-0.065	0.5246	ras homolog family member T1
8149	RFX6	-0.065	0.3227	regulatory factor X, 6
8150	PSG11	-0.065	0.3971	pregnancy specific beta-1-glycoprotein 11
8151	LIX1	-0.065	0.3904	Lix1 homolog (chicken)
8152	HDDC2	-0.065	0.5086	HD domain containing 2
8153	FAM225A	-0.065	0.4456	family with sequence similarity 225, member A (non-protein coding)
8154	CYP4Z1	-0.065	0.3564	cytochrome P450, family 4, subfamily Z, polypeptide 1
8155	C12orf68	-0.065	0.4356	chromosome 12 open reading frame 68
8156	BIRC3	-0.065	0.4818	baculoviral IAP repeat containing 3
8157	ACVR1C	-0.065	0.3999	activin A receptor, type IC
8158	ABCD4	-0.065	0.4599	ATP-binding cassette, sub-family D (ALD), member 4
8159	TRBV10-2	-0.0655	0.4643	T cell receptor beta variable 10-2
8160	CCR2	-0.0655	0.4153	chemokine (C-C motif) receptor 2
8161	ZNF480	-0.0658	0.521	zinc finger protein 480
8162	KIF13B	-0.0658	0.5236	kinesin family member 13B
8163	GPR65	-0.0658	0.4326	G protein-coupled receptor 65
8164	CD2	-0.0658	0.3868	CD2 molecule
8165	CCR6	-0.0658	0.3796	chemokine (C-C motif) receptor 6
8166	ADRB3	-0.0658	0.444	adrenoceptor beta 3
8167	CCDC7	-0.0662	0.4773	coiled-coil domain containing 7
8168	TRMT112	-0.0667	0.5126	tRNA methyltransferase 11-2 homolog (S. cerevisiae)
8169	PPP1R15A	-0.0667	0.4614	protein phosphatase 1, regulatory subunit 15A
8170	PLCG2	-0.0667	0.4549	phospholipase C, gamma 2 (phosphatidylinositol-specific)
8171	NARS	-0.0667	0.5177	asparaginyl-tRNA synthetase
8172	KSR1	-0.0667	0.4767	kinase suppressor of ras 1
8173	INVS	-0.0667	0.5383	inversin
8174	FGB	-0.0667	0.4276	fibrinogen beta chain
8175	A4GNT	-0.0667	0.4239	alpha-1,4-N-acetylglucosaminyltransferase
8176	RNF25	-0.0675	0.5117	ring finger protein 25
8177	RNF167	-0.0675	0.4999	ring finger protein 167
8178	NHLRC4	-0.0675	0.4209	NHL repeat containing 4
8179	MRI1	-0.0675	0.4732	methylthioribose-1-phosphate isomerase homolog (S. cerevisiae)
8180	MBP	-0.0675	0.4487	myelin basic protein
8181	LOC90246	-0.0675	0.4416	uncharacterized LOC90246
8182	LOC729420	-0.0675	0.3705	uncharacterized LOC729420
8183	LMLN	-0.0675	0.5131	leishmanolysin-like (metallopeptidase M8 family)
8184	GPX5	-0.0675	0.4256	glutathione peroxidase 5 (epididymal androgen-related protein)
8185	FXYD1	-0.0675	0.4534	FXYD domain containing ion transport regulator 1
8186	FAM221A	-0.0675	0.5014	family with sequence similarity 221, member A
8187	EPB41L3	-0.0675	0.4887	erythrocyte membrane protein band 4.1-like 3
8188	CD1A	-0.0675	0.4289	CD1a molecule
8189	XYLT2	-0.0683	0.4846	xylosyltransferase II
8190	TICAM1	-0.0683	0.4443	toll-like receptor adaptor molecule 1
8191	ROM1	-0.0683	0.451	retinal outer segment membrane protein 1
8192	MRPS33	-0.0683	0.4982	mitochondrial ribosomal protein S33
8193	LAP3	-0.0683	0.509	leucine aminopeptidase 3
8194	ARPC5	-0.0683	0.5052	actin related protein 2/3 complex, subunit 5, 16kDa
8195	ABCA7	-0.0683	0.4499	ATP-binding cassette, sub-family A (ABC1), member 7
8196	ST3GAL3	-0.0688	0.4779	ST3 beta-galactoside alpha-2,3-sialyltransferase 3
8197	LOC728769	-0.0688	0.4868	uncharacterized LOC728769
8198	GPR155	-0.0688	0.4948	G protein-coupled receptor 155
8199	DGKH	-0.0688	0.4979	diacylglycerol kinase, eta
8200	BEX5	-0.0688	0.4332	brain expressed, X-linked 5
8201	TRPV1	-0.0692	0.4351	transient receptor potential cation channel, subfamily V, member 1
8202	PHLPP2	-0.0692	0.5281	PH domain and leucine rich repeat protein phosphatase 2
8203	OXR1	-0.0692	0.5176	oxidation resistance 1
8204	NUDT21	-0.0692	0.5139	nudix (nucleoside diphosphate linked moiety X)-type motif 21
8205	NAB2	-0.0692	0.4663	NGFI-A binding protein 2 (EGR1 binding protein 2)
8206	GABRR1	-0.0692	0.3897	gamma-aminobutyric acid (GABA) A receptor, rho 1
8207	CERS4	-0.0692	0.4713	ceramide synthase 4
8208	UHRF2	-0.07	0.5253	ubiquitin-like with PHD and ring finger domains 2, E3 ubiquitin protein ligase
8209	SPAG9	-0.07	0.5141	sperm associated antigen 9
8210	SCN10A	-0.07	0.4278	sodium channel, voltage-gated, type X, alpha subunit
8211	RETNLB	-0.07	0.4036	resistin like beta
8212	PHAX	-0.07	0.5269	phosphorylated adaptor for RNA export
8213	NRG4	-0.07	0.317	neuregulin 4
8214	NMNAT1	-0.07	0.5299	nicotinamide nucleotide adenylyltransferase 1
8215	GTF3C1	-0.07	0.5151	general transcription factor IIIC, polypeptide 1, alpha 220kDa
8216	GP5	-0.07	0.4129	glycoprotein V (platelet)
8217	FXYD4	-0.07	0.3776	FXYD domain containing ion transport regulator 4
8218	FLYWCH1	-0.07	0.4676	FLYWCH-type zinc finger 1
8219	CHRNB4	-0.07	0.4132	cholinergic receptor, nicotinic, beta 4 (neuronal)
8220	BCL2L14	-0.07	0.4241	BCL2-like 14 (apoptosis facilitator)
8221	ATP5H	-0.07	0.501	ATP synthase, H+ transporting, mitochondrial Fo complex, subunit d
8222	AFP	-0.07	0.4068	alpha-fetoprotein
8223	ST6GAL1	-0.0708	0.5232	ST6 beta-galactosamide alpha-2,6-sialyltranferase 1
8224	MPRIP	-0.0708	0.5	myosin phosphatase Rho interacting protein
8225	KIF25	-0.0708	0.4409	kinesin family member 25
8226	KCNV1	-0.0708	0.3847	potassium channel, subfamily V, member 1
8227	GCNT4	-0.0708	0.471	glucosaminyl (N-acetyl) transferase 4, core 2
8228	ERO1LB	-0.0708	0.4986	ERO1-like beta (S. cerevisiae)
8229	DEC1	-0.0708	0.4263	deleted in esophageal cancer 1
8230	KIR2DL1	-0.0709	0.4121	killer cell immunoglobulin-like receptor, two domains, long cytoplasmic tail, 1

8231	PRELID1	-0.0712	0.5088	PRELI domain containing 1
8232	ODZ2	-0.0712	0.382	odz, odd Oz/ten-m homolog 2 (Drosophila)
8233	KDM1B	-0.0712	0.5421	lysine (K)-specific demethylase 1B
8234	COL6A6	-0.0712	0.3809	collagen, type VI, alpha 6
8235	ANKS4B	-0.0712	0.4073	ankyrin repeat and sterile alpha motif domain containing 4B
8236	LOC727944	-0.0714	0.0004	uncharacterized LOC727944
8237	TNNT1	-0.0717	0.4339	troponin T type 1 (skeletal, slow)
8238	TNFRSF21	-0.0717	0.5186	tumor necrosis factor receptor superfamily, member 21
8239	TASP1	-0.0717	0.5143	taspase, threonine aspartase, 1
8240	JAKMIP3	-0.0717	0.3489	Janus kinase and microtubule interacting protein 3
8241	GAGE1	-0.0717	0.3721	G antigen 1
8242	FLT3LG	-0.0717	0.4434	fms-related tyrosine kinase 3 ligand
8243	CORO2B	-0.0717	0.4555	coronin, actin binding protein, 2B
8244	APPBP2	-0.0717	0.5282	amyloid beta precursor protein (cytoplasmic tail) binding protein 2
8245	ZNF555	-0.0725	0.526	zinc finger protein 555
8246	ZNF544	-0.0725	0.5061	zinc finger protein 544
8247	TNNT3	-0.0725	0.4318	troponin T type 3 (skeletal, fast)
8248	TAS2R50	-0.0725	0.4041	taste receptor, type 2, member 50
8249	STYXL1	-0.0725	0.5066	serine/threonine/tyrosine interacting-like 1
8250	STK32A	-0.0725	0.3783	serine/threonine kinase 32A
8251	SNAPC1	-0.0725	0.5094	small nuclear RNA activating complex, polypeptide 1, 43kDa
8252	SEC14L2	-0.0725	0.4825	SEC14-like 2 (S. cerevisiae)
8253	RFK	-0.0725	0.4977	riboflavin kinase
8254	PELI1	-0.0725	0.5	pellino E3 ubiquitin protein ligase 1
8255	MUC3A	-0.0725	0.4493	mucin 3A, cell surface associated
8256	MALSU1	-0.0725	0.5156	mitochondrial assembly of ribosomal large subunit 1
8257	GPLD1	-0.0725	0.4594	glycosylphosphatidylinositol specific phospholipase D1
8258	GJD4	-0.0725	0.4035	gap junction protein, delta 4, 40.1kDa
8259	DZANK1	-0.0725	0.4541	double zinc ribbon and ankyrin repeat domains 1
8260	CLYBL	-0.0725	0.4804	citrate lyase beta like
8261	ACMSD	-0.0725	0.361	aminocarboxymuconate semialdehyde decarboxylase
8262	ACSL4	-0.0729	0.5232	acyl-CoA synthetase long-chain family member 4
8263	ZNF557	-0.0733	0.5182	zinc finger protein 557
8264	RECQL	-0.0733	0.5197	RecQ protein-like (DNA helicase Q1-like)
8265	PCGF3	-0.0733	0.5149	polycomb group ring finger 3
8266	OR1A1	-0.0733	0.431	olfactory receptor, family 1, subfamily A, member 1
8267	MAP3K14	-0.0733	0.4626	mitogen-activated protein kinase kinase kinase 14
8268	LMBR1L	-0.0733	0.4852	limb region 1 homolog (mouse)-like
8269	GIP	-0.0733	0.4239	gastric inhibitory polypeptide
8270	DHTKD1	-0.0733	0.5314	dehydrogenase E1 and transketolase domain containing 1
8271	CCDC22	-0.0733	0.4779	coiled-coil domain containing 22
8272	CASKIN2	-0.0733	0.4775	CASK interacting protein 2
8273	CASD1	-0.0733	0.5293	CAS1 domain containing 1
8274	AGXT	-0.0733	0.4325	alanine-glyoxylate aminotransferase
8275	LOC100128909	-0.0737	0.4033	uncharacterized LOC100128909
8276	TGM4	-0.0742	0.4372	transglutaminase 4 (prostate)
8277	SEMA3B	-0.0742	0.4755	sema domain, immunoglobulin domain (Ig), short basic domain, secreted, (semaphorin) 3B
8278	SELT	-0.0742	0.5047	selenoprotein T
8279	SCML1	-0.0742	0.5094	sex comb on midleg-like 1 (Drosophila)
8280	RAB31	-0.0742	0.5053	RAB31, member RAS oncogene family
8281	KIAA1704	-0.0742	0.517	KIAA1704
8282	GRWD1	-0.0742	0.4872	glutamate-rich WD repeat containing 1
8283	FGFR4	-0.0742	0.4605	fibroblast growth factor receptor 4
8284	CELA3B	-0.0742	0.428	chymotrypsin-like elastase family, member 3B
8285	CCL16	-0.0742	0.4406	chemokine (C-C motif) ligand 16
8286	ADRB1	-0.0742	0.4253	adrenoceptor beta 1
8287	ZBTB44	-0.075	0.5155	zinc finger and BTB domain containing 44
8288	WFDC6	-0.075	0.3805	WAP four-disulfide core domain 6
8289	SGPP1	-0.075	0.5084	sphingosine-1-phosphate phosphatase 1
8290	SCOC	-0.075	0.5105	short coiled-coil protein
8291	RARS	-0.075	0.5195	arginyl-tRNA synthetase
8292	MINOS1	-0.075	0.5034	mitochondrial inner membrane organizing system 1
8293	LOC647070	-0.075	0.0004	uncharacterized LOC647070
8294	IL36B	-0.075	0.3953	interleukin 36, beta
8295	FNDC5	-0.075	0.4359	fibronectin type III domain containing 5
8296	DDX6	-0.075	0.5225	DEAD (Asp-Glu-Ala-Asp) box helicase 6
8297	C9orf123	-0.075	0.5111	chromosome 9 open reading frame 123
8298	C1orf122	-0.075	0.4598	chromosome 1 open reading frame 122
8299	A2M-AS1	-0.0757	0	A2M antisense RNA 1 (non-protein coding)
8300	VEZF1	-0.0758	0.5194	vascular endothelial zinc finger 1
8301	PROP1	-0.0758	0.4338	PROP paired-like homeobox 1
8302	POGZ	-0.0758	0.5073	pogo transposable element with ZNF domain
8303	PAEP	-0.0758	0.4131	progestagen-associated endometrial protein
8304	KAZN	-0.0758	0.4532	kazrin, periplakin interacting protein
8305	HYOU1	-0.0758	0.5107	hypoxia up-regulated 1
8306	HCFC1R1	-0.0758	0.464	host cell factor C1 regulator 1 (XPO1 dependent)
8307	GMPPB	-0.0758	0.4683	GDP-mannose pyrophosphorylase B
8308	DOK1	-0.0758	0.4809	docking protein 1, 62kDa (downstream of tyrosine kinase 1)
8309	DLEU1	-0.0758	0.5007	deleted in lymphocytic leukemia 1 (non-protein coding)
8310	CEACAM3	-0.0758	0.4437	carcinoembryonic antigen-related cell adhesion molecule 3
8311	CCL24	-0.0758	0.4298	chemokine (C-C motif) ligand 24
8312	NALCN	-0.0763	0.4249	sodium leak channel, non-selective
8313	MRPL54	-0.0763	0.4563	mitochondrial ribosomal protein L54
8314	CTXN3	-0.0763	0.3914	cortexin 3

8315	WNT10B	-0.0767	0.4531	wingless-type MMTV integration site family, member 10B
8316	TTTY15	-0.0767	0.4462	testis-specific transcript, Y-linked 15 (non-protein coding)
8317	TNFSF4	-0.0767	0.4909	tumor necrosis factor (ligand) superfamily, member 4
8318	RBM26	-0.0767	0.521	RNA binding motif protein 26
8319	PTP4A2	-0.0767	0.5164	protein tyrosine phosphatase type IVA, member 2
8320	NRBP1	-0.0767	0.5244	nuclear receptor binding protein 1
8321	MYLK3	-0.0767	0.4163	myosin light chain kinase 3
8322	IMPDH2	-0.0767	0.504	IMP (inosine 5'-monophosphate) dehydrogenase 2
8323	HTR1E	-0.0767	0.4291	5-hydroxytryptamine (serotonin) receptor 1E, G protein-coupled
8324	CCDC48	-0.0767	0.4467	coiled-coil domain containing 48
8325	BST2	-0.0767	0.4952	bone marrow stromal cell antigen 2
8326	NCAM2	-0.0771	0.4531	neural cell adhesion molecule 2
8327	PRKCSH	-0.0775	0.4942	protein kinase C substrate 80K-H
8328	PCP4	-0.0775	0.4422	Purkinje cell protein 4
8329	MRPL40	-0.0775	0.505	mitochondrial ribosomal protein L40
8330	LOC100128164	-0.0775	0.4302	four and a half LIM domains 1 pseudogene
8331	HERC2	-0.0775	0.5149	HECT and RLD domain containing E3 ubiquitin protein ligase 2
8332	CHRNA1	-0.0775	0.4288	cholinergic receptor, nicotinic, alpha 1 (muscle)
8333	C8B	-0.0775	0.4349	complement component 8, beta polypeptide
8334	B9D2	-0.0775	0.456	B9 protein domain 2
8335	ZFP30	-0.0783	0.5244	zinc finger protein 30 homolog (mouse)
8336	RPS6KB1	-0.0783	0.5193	ribosomal protein S6 kinase, 70kDa, polypeptide 1
8337	ROBO3	-0.0783	0.4404	roundabout, axon guidance receptor, homolog 3 (Drosophila)
8338	PTGIR	-0.0783	0.4617	prostaglandin I2 (prostacyclin) receptor (IP)
8339	PDE4A	-0.0783	0.489	phosphodiesterase 4A, cAMP-specific
8340	MGC2889	-0.0783	0.4597	uncharacterized protein MGC2889
8341	FOXO3	-0.0783	0.4455	forkhead box O3
8342	FAM98A	-0.0783	0.5223	family with sequence similarity 98, member A
8343	DAK	-0.0783	0.4802	dihydroxyacetone kinase 2 homolog (S. cerevisiae)
8344	CCL1	-0.0783	0.4344	chemokine (C-C motif) ligand 1
8345	APOLD1	-0.0783	0.4958	apolipoprotein L domain containing 1
8346	LINC00092	-0.0786	0	long intergenic non-protein coding RNA 92
8347	STON2	-0.0787	0.4515	stonin 2
8348	QRFPR	-0.0787	0.3775	pyroglutamylated RFamide peptide receptor
8349	LOC100128139	-0.0787	0.4286	uncharacterized LOC100128139
8350	FAM195A	-0.0787	0.4479	family with sequence similarity 195, member A
8351	FAM176A	-0.0787	0.4064	family with sequence similarity 176, member A
8352	ABRACL	-0.0787	0.5165	ABRA C-terminal like
8353	NOX5	-0.0789	0.4148	NADPH oxidase, EF-hand calcium binding domain 5
8354	ZNF93	-0.0792	0.4894	zinc finger protein 93
8355	SRCAP	-0.0792	0.5081	Snf2-related CREBBP activator protein
8356	SBNO2	-0.0792	0.4784	strawberry notch homolog 2 (Drosophila)
8357	NDUFC2	-0.0792	0.5152	NADH dehydrogenase (ubiquinone) 1, subcomplex unknown, 2, 14.5kDa
8358	NDUFA5	-0.0792	0.51	NADH dehydrogenase (ubiquinone) 1 alpha subcomplex, 5, 13kDa
8359	HMGN5	-0.0792	0.5008	high mobility group nucleosome binding domain 5
8360	FGF21	-0.0792	0.4352	fibroblast growth factor 21
8361	FAM20B	-0.0792	0.5343	family with sequence similarity 20, member B
8362	CHCHD8	-0.0792	0.4893	coiled-coil-helix-coiled-coil-helix domain containing 8
8363	C9orf116	-0.0792	0.4781	chromosome 9 open reading frame 116
8364	ZNF596	-0.08	0.5094	zinc finger protein 596
8365	ZNF253	-0.08	0.5041	zinc finger protein 253
8366	ZNF180	-0.08	0.5098	zinc finger protein 180
8367	UBXN6	-0.08	0.489	UBX domain protein 6
8368	SORT1	-0.08	0.5506	sortilin 1
8369	SLC23A1	-0.08	0.3606	solute carrier family 23 (nucleobase transporters), member 1
8370	RAB3B	-0.08	0.4686	RAB3B, member RAS oncogene family
8371	PHF1	-0.08	0.504	PHD finger protein 1
8372	PDAP1	-0.08	0.4983	PDGFA associated protein 1
8373	LUZP2	-0.08	0.4522	leucine zipper protein 2
8374	LRRC8C	-0.08	0.5273	leucine rich repeat containing 8 family, member C
8375	ARGLU1	-0.08	0.5054	arginine and glutamate rich 1
8376	ACLY	-0.08	0.527	ATP citrate lyase
8377	ZNF423	-0.0808	0.4431	zinc finger protein 423
8378	RNF14	-0.0808	0.5237	ring finger protein 14
8379	PRKCD	-0.0808	0.5075	protein kinase C, delta
8380	GMFB	-0.0808	0.51	glia maturation factor, beta
8381	CYP51A1	-0.0808	0.5108	cytochrome P450, family 51, subfamily A, polypeptide 1
8382	CRYBA1	-0.0808	0.4195	crystallin, beta A1
8383	CHRNA10	-0.0808	0.443	cholinergic receptor, nicotinic, alpha 10 (neuronal)
8384	APOL5	-0.0808	0.4386	apolipoprotein L, 5
8385	AP1AR	-0.0808	0.5187	adaptor-related protein complex 1 associated regulatory protein
8386	SH3BP5L	-0.0812	0.4797	SH3-binding domain protein 5-like
8387	DOK6	-0.0812	0.455	docking protein 6
8388	ZNF80	-0.0817	0.3807	zinc finger protein 80
8389	YIPF2	-0.0817	0.4814	Yip1 domain family, member 2
8390	TMX2	-0.0817	0.5222	thioredoxin-related transmembrane protein 2
8391	RABGAP1L	-0.0817	0.5277	RAB GTPase activating protein 1-like
8392	MAPK10	-0.0817	0.4824	mitogen-activated protein kinase 10
8393	MAGEA8	-0.0817	0.4006	melanoma antigen family A, 8
8394	LEFTY2	-0.0817	0.4361	left-right determination factor 2
8395	FCAR	-0.0817	0.4401	Fc fragment of IgA, receptor for
8396	EIF5A2	-0.0817	0.5159	eukaryotic translation initiation factor 5A2
8397	DCTN4	-0.0817	0.5322	dynactin 4 (p62)
8398	CYP1A1	-0.0817	0.4437	cytochrome P450, family 1, subfamily A, polypeptide 1

8399	CACNB2	-0.0817	0.4433	calcium channel, voltage-dependent, beta 2 subunit
8400	ZNF518A	-0.0825	0.5244	zinc finger protein 518A
8401	ZDHHC17	-0.0825	0.5141	zinc finger, DHHC-type containing 17
8402	WNT8B	-0.0825	0.4474	wingless-type MMTV integration site family, member 8B
8403	WDR90	-0.0825	0.4374	WD repeat domain 90
8404	RBAK	-0.0825	0.5261	RB-associated KRAB zinc finger
8405	PHTF2	-0.0825	0.5206	putative homeodomain transcription factor 2
8406	NTRK1	-0.0825	0.4446	neurotrophic tyrosine kinase, receptor, type 1
8407	MAPKBP1	-0.0825	0.4948	mitogen-activated protein kinase binding protein 1
8408	IFNA21	-0.0825	0.4477	interferon, alpha 21
8409	HMX2	-0.0825	0.3733	H6 family homeobox 2
8410	GPR37	-0.0825	0.4555	G protein-coupled receptor 37 (endothelin receptor type B-like)
8411	FGF13	-0.0825	0.4429	fibroblast growth factor 13
8412	FAM84A	-0.0825	0.4304	family with sequence similarity 84, member A
8413	CDKN2A	-0.0825	0.4688	cyclin-dependent kinase inhibitor 2A
8414	CCDC169	-0.0825	0.4281	coiled-coil domain containing 169
8415	C4orf36	-0.0825	0.4152	chromosome 4 open reading frame 36
8416	B3GNT1	-0.0825	0.5231	UDP-GlcNAc:betaGal beta-1,3-N-acetylglucosaminyltransferase 1
8417	APCS	-0.0825	0.4345	amyloid P component, serum
8418	SNORD104	-0.0829	0.4587	small nucleolar RNA, C/D box 104
8419	LOC284837	-0.0829	0.4054	uncharacterized LOC284837
8420	LOC116437	-0.0829	0	uncharacterized LOC116437
8421	RBP4	-0.0833	0.4516	retinol binding protein 4, plasma
8422	NUCKS1	-0.0833	0.522	nuclear casein kinase and cyclin-dependent kinase substrate 1
8423	MRPL44	-0.0833	0.5246	mitochondrial ribosomal protein L44
8424	INPP5K	-0.0833	0.5125	inositol polyphosphate-5-phosphatase K
8425	FAM127A	-0.0833	0.5156	family with sequence similarity 127, member A
8426	ERN1	-0.0833	0.4743	endoplasmic reticulum to nucleus signaling 1
8427	CTNNBIP1	-0.0833	0.4825	catenin, beta interacting protein 1
8428	CRYBB3	-0.0833	0.4402	crystallin, beta B3
8429	CDX2	-0.0833	0.4419	caudal type homeobox 2
8430	STAC2	-0.0838	0.4385	SH3 and cysteine rich domain 2
8431	RADIL	-0.0838	0.4177	Ras association and DIL domains
8432	FAM70B	-0.0838	0.4399	family with sequence similarity 70, member B
8433	ABHD12B	-0.0838	0.3865	abhydrolase domain containing 12B
8434	NF2	-0.0842	0.5191	neurofibromin 2 (merlin)
8435	NECAP2	-0.0842	0.5375	NECAP endocytosis associated 2
8436	GPT	-0.0842	0.4335	glutamic-pyruvate transaminase (alanine aminotransferase)
8437	GLDC	-0.0842	0.4642	glycine dehydrogenase (decarboxylating)
8438	DUSP22	-0.0842	0.5127	dual specificity phosphatase 22
8439	DEXI	-0.0842	0.5289	Dexi homolog (mouse)
8440	C6	-0.0842	0.4514	complement component 6
8441	ZNF785	-0.085	0.4388	zinc finger protein 785
8442	ZNF594	-0.085	0.5257	zinc finger protein 594
8443	TIGD2	-0.085	0.5394	tigger transposable element derived 2
8444	SNX4	-0.085	0.5247	sorting nexin 4
8445	SLC6A12	-0.085	0.4601	solute carrier family 6 (neurotransmitter transporter, betaine/GABA), member 12
8446	NSMCE4A	-0.085	0.5223	non-SMC element 4 homolog A (S. cerevisiae)
8447	LOC401052	-0.085	0.4179	uncharacterized LOC401052
8448	IL1RAPL2	-0.085	0.4401	interleukin 1 receptor accessory protein-like 2
8449	HBBP1	-0.085	0.4537	hemoglobin, beta pseudogene 1
8450	GALNT8	-0.085	0.4427	UDP-N-acetyl-alpha-D-galactosamine:polypeptide N-acetylgalactosaminyltransferase 8 (GalNAc-T8)
8451	FAM132A	-0.085	0.4107	family with sequence similarity 132, member A
8452	ALG9	-0.085	0.5465	asparagine-linked glycosylation 9, alpha-1,2-mannosyltransferase homolog (S. cerevisiae)
8453	ALG3	-0.085	0.4936	asparagine-linked glycosylation 3, alpha-1,3- mannosyltransferase homolog (S. cerevisiae)
8454	RPPH1	-0.0857	0.4563	ribonuclease P RNA component H1
8455	LOC441179	-0.0857	0.3743	uncharacterized LOC441179
8456	C8orf82	-0.0857	0.4551	chromosome 8 open reading frame 82
8457	SEMG2	-0.0858	0.4203	semenogelin II
8458	RGS1	-0.0858	0.4699	regulator of G-protein signaling 1
8459	P2RY1	-0.0858	0.4521	purinergic receptor P2Y, G-protein coupled, 1
8460	DRD1	-0.0858	0.4461	dopamine receptor D1
8461	COG2	-0.0858	0.5406	component of oligomeric golgi complex 2
8462	CENPI	-0.0858	0.4847	centromere protein I
8463	PLEKHA3	-0.0862	0.5283	pleckstrin homology domain containing, family A (phosphoinositide binding specific) member 3
8464	KAAG1	-0.0862	0.4153	kidney associated antigen 1
8465	EXOC3L2	-0.0862	0.4266	exocyst complex component 3-like 2
8466	ALDH16A1	-0.0862	0.4721	aldehyde dehydrogenase 16 family, member A1
8467	UBE2J1	-0.0867	0.5266	ubiquitin-conjugating enzyme E2, J1
8468	TRH	-0.0867	0.4376	thyrotropin-releasing hormone
8469	KIF20B	-0.0867	0.5112	kinesin family member 20B
8470	IST1	-0.0867	0.5199	increased sodium tolerance 1 homolog (yeast)
8471	IL17RB	-0.0867	0.4853	interleukin 17 receptor B
8472	CCL13	-0.0867	0.4513	chemokine (C-C motif) ligand 13
8473	C8orf37	-0.0867	0.537	chromosome 8 open reading frame 37
8474	FCGR3B	-0.0873	0.4613	Fc fragment of IgG, low affinity IIIb, receptor (CD16b)
8475	VPS37C	-0.0875	0.4676	vacuolar protein sorting 37 homolog C (S. cerevisiae)
8476	UPP1	-0.0875	0.4806	uridine phosphorylase 1
8477	TOR1B	-0.0875	0.5411	torsin family 1, member B (torsin B)
8478	TMUB1	-0.0875	0.4826	transmembrane and ubiquitin-like domain containing 1
8479	SIAH3	-0.0875	0.373	siah E3 ubiquitin protein ligase family member 3
8480	SETDB2	-0.0875	0.537	SET domain, bifurcated 2
8481	NANP	-0.0875	0.5119	N-acetylneuraminic acid phosphatase
8482	KIAA1683	-0.0875	0.4451	KIAA1683

8483	IRF7	-0.0875	0.4361	interferon regulatory factor 7
8484	HTR4	-0.0875	0.4505	5-hydroxytryptamine (serotonin) receptor 4, G protein-coupled
8485	GALNT2	-0.0875	0.5385	UDP-N-acetyl-alpha-D-galactosamine:polypeptide N-acetylgalactosaminyltransferase 2 (GalNAc-T2)
8486	CR1	-0.0875	0.4306	complement component (3b/4b) receptor 1 (Knops blood group)
8487	TXNRD2	-0.0883	0.4801	thioredoxin reductase 2
8488	THRA	-0.0883	0.5198	thyroid hormone receptor, alpha
8489	RALGAPA2	-0.0883	0.5517	Ral GTPase activating protein, alpha subunit 2 (catalytic)
8490	PRKAR2A	-0.0883	0.5558	protein kinase, cAMP-dependent, regulatory, type II, alpha
8491	MAP2K6	-0.0883	0.52	mitogen-activated protein kinase kinase 6
8492	FGF23	-0.0883	0.4358	fibroblast growth factor 23
8493	ATP5G1	-0.0883	0.4863	ATP synthase, H+ transporting, mitochondrial Fo complex, subunit C1 (subunit 9)
8494	MGC45800	-0.0886	0.4366	uncharacterized LOC90768
8495	LOC255512	-0.0886	0	uncharacterized LOC255512
8496	TEDDM1	-0.0887	0.3891	transmembrane epididymal protein 1
8497	SYAP1	-0.0887	0.5336	synapse associated protein 1
8498	RALGPS2	-0.0887	0.5336	Ral GEF with PH domain and SH3 binding motif 2
8499	PRSS35	-0.0887	0.4502	protease, serine, 35
8500	KIAA1244	-0.0887	0.4766	KIAA1244
8501	FAM104B	-0.0887	0.5084	family with sequence similarity 104, member B
8502	C14orf39	-0.0887	0.4081	chromosome 14 open reading frame 39
8503	RPS6KA3	-0.0892	0.5261	ribosomal protein S6 kinase, 90kDa, polypeptide 3
8504	PAX5	-0.0892	0.4529	paired box 5
8505	KANSL3	-0.0892	0.5464	KAT8 regulatory NSL complex subunit 3
8506	FGF18	-0.0892	0.4592	fibroblast growth factor 18
8507	DUSP13	-0.0892	0.4326	dual specificity phosphatase 13
8508	CDH9	-0.0892	0.4265	cadherin 9, type 2 (T1-cadherin)
8509	BTAF1	-0.0892	0.5182	BTAF1 RNA polymerase II, B-TFIID transcription factor-associated, 170kDa (Mot1 homolog, S. cerevisiae)
8510	AKT1	-0.0892	0.5194	v-akt murine thymoma viral oncogene homolog 1
8511	TRIB2	-0.09	0.5371	tribbles homolog 2 (Drosophila)
8512	STGC3	-0.09	0.4338	uncharacterized STGC3
8513	SRSF10	-0.09	0.5113	serine/arginine-rich splicing factor 10
8514	SPATA6L	-0.09	0.4325	spermatogenesis associated 6-like
8515	SLC29A1	-0.09	0.5129	solute carrier family 29 (nucleoside transporters), member 1
8516	NXT2	-0.09	0.5088	nuclear transport factor 2-like export factor 2
8517	NMU	-0.09	0.4521	neuromedin U
8518	HEATR8	-0.09	0.4457	HEAT repeat containing 8
8519	FAM165B	-0.09	0.4985	family with sequence similarity 165, member B
8520	DNAJA4	-0.09	0.4917	DnaJ (Hsp40) homolog, subfamily A, member 4
8521	DHRS7C	-0.09	0.4095	dehydrogenase/reductase (SDR family) member 7C
8522	C8orf22	-0.09	0.3828	chromosome 8 open reading frame 22
8523	BGLAP	-0.09	0.4339	bone gamma-carboxyglutamate (gla) protein
8524	ZNF571	-0.0908	0.528	zinc finger protein 571
8525	ZNF34	-0.0908	0.4992	zinc finger protein 34
8526	TPSG1	-0.0908	0.4436	tryptase gamma 1
8527	POLR2K	-0.0908	0.51	polymerase (RNA) II (DNA directed) polypeptide K, 7.0kDa
8528	MARCH5	-0.0908	0.5324	membrane-associated ring finger (C3HC4) 5
8529	IFI6	-0.0908	0.4922	interferon, alpha-inducible protein 6
8530	GTF3C3	-0.0908	0.5337	general transcription factor IIIC, polypeptide 3, 102kDa
8531	ENC1	-0.0908	0.5296	ectodermal-neural cortex 1 (with BTB-like domain)
8532	C16orf3	-0.0908	0.4392	chromosome 16 open reading frame 3
8533	ARNTL	-0.0908	0.5402	aryl hydrocarbon receptor nuclear translocator-like
8534	ZNF608	-0.0913	0.511	zinc finger protein 608
8535	SIGLEC11	-0.0913	0.4192	sialic acid binding Ig-like lectin 11
8536	MYO3B	-0.0913	0.3918	myosin IIIB
8537	KRTAP2-1	-0.0913	0.4243	keratin associated protein 2-1
8538	KCTD4	-0.0913	0.3993	potassium channel tetramerisation domain containing 4
8539	C10orf114	-0.0913	0.4565	chromosome 10 open reading frame 114
8540	ACAD9	-0.0913	0.55	acyl-CoA dehydrogenase family, member 9
8541	KLRK1	-0.0914	0.4702	killer cell lectin-like receptor subfamily K, member 1
8542	C11orf53	-0.0914	0.4179	chromosome 11 open reading frame 53
8543	TMEM8B	-0.0917	0.4881	transmembrane protein 8B
8544	SPATA5L1	-0.0917	0.5228	spermatogenesis associated 5-like 1
8545	MKS1	-0.0917	0.527	Meckel syndrome, type 1
8546	ITK	-0.0917	0.4208	IL2-inducible T-cell kinase
8547	GYPC	-0.0917	0.47	glycophorin C (Gerbich blood group)
8548	GADD45G	-0.0917	0.459	growth arrest and DNA-damage-inducible, gamma
8549	FAM171A1	-0.0917	0.53	family with sequence similarity 171, member A1
8550	CLEC4M	-0.0917	0.447	C-type lectin domain family 4, member M
8551	C12orf29	-0.0917	0.5134	chromosome 12 open reading frame 29
8552	ZDHHC8P1	-0.0925	0.4476	zinc finger, DHHC-type containing 8 pseudogene 1
8553	ZBTB25	-0.0925	0.5391	zinc finger and BTB domain containing 25
8554	TSG101	-0.0925	0.5345	tumor susceptibility gene 101
8555	TP53INP1	-0.0925	0.5203	tumor protein p53 inducible nuclear protein 1
8556	SPRR2C	-0.0925	0.4219	small proline-rich protein 2C (pseudogene)
8557	SEC11C	-0.0925	0.532	SEC11 homolog C (S. cerevisiae)
8558	RSPH6A	-0.0925	0.4328	radial spoke head 6 homolog A (Chlamydomonas)
8559	NKX2-3	-0.0925	0.3803	NK2 homeobox 3
8560	N4BP2L1	-0.0925	0.5068	NEDD4 binding protein 2-like 1
8561	GNB2	-0.0925	0.4942	guanine nucleotide binding protein (G protein), beta polypeptide 2
8562	EMILIN2	-0.0925	0.4591	elastin microfibril interfacer 2
8563	CLEC5A	-0.0925	0.4498	C-type lectin domain family 5, member A
8564	CHCHD1	-0.0925	0.5244	coiled-coil-helix-coiled-coil-helix domain containing 1
8565	CETP	-0.0925	0.4541	cholesteryl ester transfer protein, plasma
8566	CDK6	-0.0925	0.5412	cyclin-dependent kinase 6

8567	C14orf37	-0.0925	0.5074	chromosome 14 open reading frame 37
8568	C11orf86	-0.0925	0.4344	chromosome 11 open reading frame 86
8569	ADAM6	-0.0925	0.4442	ADAM metallopeptidase domain 6 (pseudogene)
8570	ZBTB10	-0.0933	0.5036	zinc finger and BTB domain containing 10
8571	PRR5	-0.0933	0.4633	proline rich 5 (renal)
8572	MUT	-0.0933	0.5393	methylmalonyl CoA mutase
8573	LPAL2	-0.0933	0.4486	lipoprotein, Lp(a)-like 2, pseudogene
8574	HOXB1	-0.0933	0.4495	homeobox B1
8575	EBP	-0.0933	0.5078	emopamil binding protein (sterol isomerase)
8576	CRX	-0.0933	0.4408	cone-rod homeobox
8577	CPT2	-0.0933	0.5385	carnitine palmitoyltransferase 2
8578	CHMP4A	-0.0933	0.5223	charged multivesicular body protein 4A
8579	ASPA	-0.0933	0.491	aspartoacylase
8580	WWC2-AS2	-0.0938	0.4453	WWC2 antisense RNA 2 (non-protein coding)
8581	ST6GALNAC1	-0.0938	0.403	ST6 (alpha-N-acetyl-neuraminyl-2,3-beta-galactosyl-1,3)-N-acetylgalactosaminide alpha-2,6-sialyltransferase 1
8582	SCGB3A2	-0.0938	0.4119	secretoglobin, family 3A, member 2
8583	PLRG1	-0.0938	0.542	pleiotropic regulator 1
8584	ZNF639	-0.0942	0.5304	zinc finger protein 639
8585	ZDHHC24	-0.0942	0.4884	zinc finger, DHHC-type containing 24
8586	RFX3	-0.0942	0.5187	regulatory factor X, 3 (influences HLA class II expression)
8587	MKL2	-0.0942	0.5384	MKL/myocardin-like 2
8588	COMMD8	-0.0942	0.5154	COMM domain containing 8
8589	CCNL1	-0.0942	0.5008	cyclin L1
8590	ATP5E	-0.0943	0.4764	ATP synthase, H+ transporting, mitochondrial F1 complex, epsilon subunit
8591	ZNF804B	-0.095	0.3841	zinc finger protein 804B
8592	VSIG1	-0.095	0.4311	V-set and immunoglobulin domain containing 1
8593	SLC46A2	-0.095	0.4205	solute carrier family 46, member 2
8594	LDLR	-0.095	0.5219	low density lipoprotein receptor
8595	IFRD1	-0.095	0.5087	interferon-related developmental regulator 1
8596	ICAM2	-0.095	0.4733	intercellular adhesion molecule 2
8597	HNRNPCL1	-0.095	0.4307	heterogeneous nuclear ribonucleoprotein C-like 1
8598	HINT3	-0.095	0.5403	histidine triad nucleotide binding protein 3
8599	CLEC1B	-0.095	0.4367	C-type lectin domain family 1, member B
8600	CARS2	-0.095	0.5393	cysteinyl-tRNA synthetase 2, mitochondrial (putative)
8601	BRAT1	-0.095	0.4715	BRCA1-associated ATM activator 1
8602	C10orf99	-0.0957	0.4096	chromosome 10 open reading frame 99
8603	PGM1	-0.0958	0.5402	phosphoglucomutase 1
8604	MLXIPL	-0.0958	0.4617	MLX interacting protein-like
8605	MLNR	-0.0958	0.4284	motilin receptor
8606	LRRC59	-0.0958	0.5149	leucine rich repeat containing 59
8607	IL2RA	-0.0958	0.4613	interleukin 2 receptor, alpha
8608	CNOT2	-0.0958	0.536	CCR4-NOT transcription complex, subunit 2
8609	BCL9	-0.0958	0.5333	B-cell CLL/lymphoma 9
8610	ZNF22	-0.0967	0.5244	zinc finger protein 22
8611	STAT5A	-0.0967	0.5103	signal transducer and activator of transcription 5A
8612	SLCO2B1	-0.0967	0.4624	solute carrier organic anion transporter family, member 2B1
8613	SCGB1A1	-0.0967	0.4382	secretoglobin, family 1A, member 1 (uteroglobin)
8614	PHF15	-0.0967	0.5394	PHD finger protein 15
8615	NPAP1	-0.0967	0.4344	nuclear pore associated protein 1
8616	MAPRE3	-0.0967	0.5332	microtubule-associated protein, RP/EB family, member 3
8617	GLRA1	-0.0967	0.4459	glycine receptor, alpha 1
8618	FBXO38	-0.0967	0.5328	F-box protein 38
8619	CHST12	-0.0967	0.5113	carbohydrate (chondroitin 4) sulfotransferase 12
8620	XKR8	-0.0971	0.481	XK, Kell blood group complex subunit-related family, member 8
8621	LOC730236	-0.0971	0.0004	uncharacterized LOC730236
8622	TMEM161A	-0.0975	0.5062	transmembrane protein 161A
8623	TAF12	-0.0975	0.5315	TAF12 RNA polymerase II, TATA box binding protein (TBP)-associated factor, 20kDa
8624	RPS19	-0.0975	0.4981	ribosomal protein S19
8625	POPDC3	-0.0975	0.4979	popeye domain containing 3
8626	MB21D1	-0.0975	0.5182	Mab-21 domain containing 1
8627	LOC441461	-0.0975	0.4641	uncharacterized LOC441461
8628	GTF2A1	-0.0975	0.564	general transcription factor IIA, 1, 19/37kDa
8629	FLJ42875	-0.0975	0.4255	uncharacterized LOC440556
8630	FBXO34	-0.0975	0.5466	F-box protein 34
8631	TSEN34	-0.0983	0.5059	tRNA splicing endonuclease 34 homolog (S. cerevisiae)
8632	TPP1	-0.0983	0.5369	tripeptidyl peptidase I
8633	SLC18A3	-0.0983	0.4502	solute carrier family 18 (vesicular acetylcholine), member 3
8634	PLA1A	-0.0983	0.4533	phospholipase A1 member A
8635	MTMR6	-0.0983	0.5241	myotubularin related protein 6
8636	KRT9	-0.0983	0.4388	keratin 9
8637	CENPC1	-0.0983	0.5286	centromere protein C 1
8638	BIRC2	-0.0983	0.5208	baculoviral IAP repeat containing 2
8639	ZNF836	-0.0986	0.564	zinc finger protein 836
8640	CYP27C1	-0.0986	0.4209	cytochrome P450, family 27, subfamily C, polypeptide 1
8641	C20orf151	-0.0988	0.4266	chromosome 20 open reading frame 151
8642	C17orf77	-0.0988	0.3612	chromosome 17 open reading frame 77
8643	TSPO2	-0.0991	0.4449	translocator protein 2
8644	ZNF205	-0.0992	0.4526	zinc finger protein 205
8645	ZFR	-0.0992	0.534	zinc finger RNA binding protein
8646	RNASE1	-0.0992	0.4817	ribonuclease, RNase A family, 1 (pancreatic)
8647	NUPL2	-0.0992	0.5261	nucleoporin like 2
8648	NR1I3	-0.0992	0.4304	nuclear receptor subfamily 1, group I, member 3
8649	NFATC1	-0.0992	0.4914	nuclear factor of activated T-cells, cytoplasmic, calcineurin-dependent 1
8650	GPR20	-0.0992	0.4575	G protein-coupled receptor 20

8651	FBXO42	-0.0992	0.5877	F-box protein 42
8652	ETF1	-0.0992	0.5217	eukaryotic translation termination factor 1
8653	EHBP1	-0.0992	0.5403	EH domain binding protein 1
8654	DDB2	-0.0992	0.5397	damage-specific DNA binding protein 2, 48kDa
8655	BICD1	-0.0992	0.5369	bicaudal D homolog 1 (Drosophila)
8656	ZNF417	-0.1	0.5235	zinc finger protein 417
8657	TTC26	-0.1	0.5319	tetratricopeptide repeat domain 26
8658	TOX3	-0.1	0.4479	TOX high mobility group box family member 3
8659	SLC29A2	-0.1	0.4738	solute carrier family 29 (nucleoside transporters), member 2
8660	PELI3	-0.1	0.4798	pellino E3 ubiquitin protein ligase family member 3
8661	MYF6	-0.1	0.4434	myogenic factor 6 (herculin)
8662	LRP8	-0.1	0.5093	low density lipoprotein receptor-related protein 8, apolipoprotein e receptor
8663	KLHL31	-0.1	0.4378	kelch-like 31 (Drosophila)
8664	ISOC2	-0.1	0.4837	isochorismatase domain containing 2
8665	GTF3C4	-0.1	0.5341	general transcription factor IIIC, polypeptide 4, 90kDa
8666	FOLH1	-0.1	0.4415	folate hydrolase (prostate-specific membrane antigen) 1
8667	DDX42	-0.1	0.5398	DEAD (Asp-Glu-Ala-Asp) box polypeptide 42
8668	CYTH2	-0.1	0.5252	cytohesin 2
8669	APOB	-0.1	0.4482	apolipoprotein B (including Ag(x) antigen)
8670	PEMT	-0.1008	0.5121	phosphatidylethanolamine N-methyltransferase
8671	OAZ2	-0.1008	0.5464	ornithine decarboxylase antizyme 2
8672	FOXJ2	-0.1008	0.5459	forkhead box J2
8673	CYP2B7P1	-0.1008	0.456	cytochrome P450, family 2, subfamily B, polypeptide 7 pseudogene 1
8674	CCRN4L	-0.1008	0.5074	CCR4 carbon catabolite repression 4-like (S. cerevisiae)
8675	ALG8	-0.1008	0.5223	asparagine-linked glycosylation 8, alpha-1,3-glucosyltransferase homolog (S. cerevisiae)
8676	ALDH1A2	-0.1008	0.4815	aldehyde dehydrogenase 1 family, member A2
8677	ZNF786	-0.1013	0.5124	zinc finger protein 786
8678	RPL22L1	-0.1013	0.5124	ribosomal protein L22-like 1
8679	FAM151A	-0.1013	0.4056	family with sequence similarity 151, member A
8680	FLJ34503	-0.1014	0.4194	uncharacterized FLJ34503
8681	NMT1	-0.1017	0.5507	N-myristoyltransferase 1
8682	MRPL23	-0.1017	0.4913	mitochondrial ribosomal protein L23
8683	KIAA0947	-0.1017	0.528	KIAA0947
8684	HAAO	-0.1017	0.4608	3-hydroxyanthranilate 3,4-dioxygenase
8685	FAAH	-0.1017	0.4915	fatty acid amide hydrolase
8686	C6orf211	-0.1017	0.5224	chromosome 6 open reading frame 211
8687	C3orf32	-0.1017	0.4504	chromosome 3 open reading frame 32
8688	ULK4	-0.1025	0.5174	unc-51-like kinase 4 (C. elegans)
8689	TTC40	-0.1025	0.4315	tetratricopeptide repeat domain 40
8690	TDRD10	-0.1025	0.4334	tudor domain containing 10
8691	SRD5A2	-0.1025	0.4596	steroid-5-alpha-reductase, alpha polypeptide 2 (3-oxo-5 alpha-steroid delta 4-dehydrogenase alpha 2)
8692	SETD8	-0.1025	0.5149	SET domain containing (lysine methyltransferase) 8
8693	PTPN11	-0.1025	0.5297	protein tyrosine phosphatase, non-receptor type 11
8694	PROSAPIP1	-0.1025	0.4756	ProSAPiP1 protein
8695	PRDM4	-0.1025	0.5562	PR domain containing 4
8696	MYH4	-0.1025	0.4291	myosin, heavy chain 4, skeletal muscle
8697	MINK1	-0.1025	0.5173	misshapen-like kinase 1
8698	FHIT	-0.1025	0.5012	fragile histidine triad
8699	EIF3E	-0.1025	0.5164	eukaryotic translation initiation factor 3, subunit E
8700	CPAMD8	-0.1025	0.4468	C3 and PZP-like, alpha-2-macroglobulin domain containing 8
8701	CAND2	-0.1025	0.4663	cullin-associated and neddylation-dissociated 2 (putative)
8702	ATF7IP	-0.1025	0.546	activating transcription factor 7 interacting protein
8703	ALPP	-0.1025	0.4471	alkaline phosphatase, placental
8704	TKT	-0.1033	0.5192	transketolase
8705	SIGLEC1	-0.1033	0.4564	sialic acid binding Ig-like lectin 1, sialoadhesin
8706	OR2S2	-0.1033	0.4245	olfactory receptor, family 2, subfamily S, member 2
8707	OR1D2	-0.1033	0.4468	olfactory receptor, family 1, subfamily D, member 2
8708	NDP	-0.1033	0.4517	Norrie disease (pseudoglioma)
8709	MEGF9	-0.1033	0.5419	multiple EGF-like-domains 9
8710	HCFC2	-0.1033	0.5355	host cell factor C2
8711	TMEM102	-0.1037	0.4816	transmembrane protein 102
8712	RHPN2	-0.1037	0.5157	rhophilin, Rho GTPase binding protein 2
8713	CILP2	-0.1037	0.4341	cartilage intermediate layer protein 2
8714	CCDC78	-0.1037	0.4229	coiled-coil domain containing 78
8715	ASIC5	-0.1037	0.4158	acid-sensing (proton-gated) ion channel family member 5
8716	WNT2B	-0.1042	0.4832	wingless-type MMTV integration site family, member 2B
8717	SP3	-0.1042	0.5078	Sp3 transcription factor
8718	MLF1	-0.1042	0.5259	myeloid leukemia factor 1
8719	KCND1	-0.1042	0.4901	potassium voltage-gated channel, Shal-related subfamily, member 1
8720	HOXB13	-0.1042	0.4543	homeobox B13
8721	HMGA2	-0.1042	0.4728	high mobility group AT-hook 2
8722	CTSS	-0.1042	0.4866	cathepsin S
8723	ASAP1	-0.1042	0.5407	ArfGAP with SH3 domain, ankyrin repeat and PH domain 1
8724	ZNF69	-0.105	0.4445	zinc finger protein 69
8725	ZNF33A	-0.105	0.5399	zinc finger protein 33A
8726	ZC3H7B	-0.105	0.5295	zinc finger CCCH-type containing 7B
8727	SPRTN	-0.105	0.5389	SprT-like N-terminal domain
8728	SMTNL2	-0.105	0.4218	smoothelin-like 2
8729	RYR1	-0.105	0.4463	ryanodine receptor 1 (skeletal)
8730	MZB1	-0.105	0.4549	marginal zone B and B1 cell-specific protein
8731	HCCS	-0.105	0.5282	holocytochrome c synthase
8732	GRIK1	-0.105	0.4621	glutamate receptor, ionotropic, kainate 1
8733	GPR171	-0.105	0.4533	G protein-coupled receptor 171
8734	GDNF	-0.105	0.4525	glial cell derived neurotrophic factor

8735	FYTTD1	-0.105	0.5345	forty-two-three domain containing 1
8736	FUBP1	-0.105	0.5277	far upstream element (FUSE) binding protein 1
8737	FAM102B	-0.105	0.5544	family with sequence similarity 102, member B
8738	ELK4	-0.105	0.533	ELK4, ETS-domain protein (SRF accessory protein 1)
8739	EIF5A	-0.105	0.4627	eukaryotic translation initiation factor 5A
8740	EHD1	-0.105	0.5173	EH-domain containing 1
8741	ARL3	-0.105	0.5473	ADP-ribosylation factor-like 3
8742	ADAM22	-0.105	0.5073	ADAM metallopeptidase domain 22
8743	ABCG5	-0.105	0.4319	ATP-binding cassette, sub-family G (WHITE), member 5
8744	PCDHA6	-0.1055	0.4764	protocadherin alpha 6
8745	ZNF592	-0.1058	0.5471	zinc finger protein 592
8746	TRIM14	-0.1058	0.5303	tripartite motif containing 14
8747	SIRT5	-0.1058	0.5376	sirtuin 5
8748	SAGE1	-0.1058	0.4306	sarcoma antigen 1
8749	PATZ1	-0.1058	0.5324	POZ (BTB) and AT hook containing zinc finger 1
8750	LAMP3	-0.1058	0.485	lysosomal-associated membrane protein 3
8751	DGCR2	-0.1058	0.5495	DiGeorge syndrome critical region gene 2
8752	AVPR2	-0.1058	0.4556	arginine vasopressin receptor 2
8753	AP1G2	-0.1058	0.5216	adaptor-related protein complex 1, gamma 2 subunit
8754	ZNF414	-0.1063	0.4622	zinc finger protein 414
8755	TMTC4	-0.1063	0.5454	transmembrane and tetratricopeptide repeat containing 4
8756	SMARCAD1	-0.1063	0.5448	SWI/SNF-related, matrix-associated actin-dependent regulator of chromatin, subfamily a, containing DEAD/H box 1
8757	RCCD1	-0.1063	0.5	RCC1 domain containing 1
8758	PLEKHG5	-0.1063	0.4672	pleckstrin homology domain containing, family G (with RhoGef domain) member 5
8759	MRRF	-0.1063	0.5258	mitochondrial ribosome recycling factor
8760	GIMAP1	-0.1063	0.449	GTPase, IMAP family member 1
8761	DFNB59	-0.1063	0.5211	deafness, autosomal recessive 59
8762	YME1L1	-0.1067	0.5231	YME1-like 1 (S. cerevisiae)
8763	SLC37A4	-0.1067	0.5195	solute carrier family 37 (glucose-6-phosphate transporter), member 4
8764	PTPN22	-0.1067	0.5046	protein tyrosine phosphatase, non-receptor type 22 (lymphoid)
8765	NR1H2	-0.1067	0.5196	nuclear receptor subfamily 1, group H, member 2
8766	LIAS	-0.1067	0.5293	lipoic acid synthetase
8767	GNPAT	-0.1067	0.5389	glyceronephosphate O-acyltransferase
8768	ZRANB2	-0.1075	0.5277	zinc finger, RAN-binding domain containing 2
8769	ZNF644	-0.1075	0.5328	zinc finger protein 644
8770	ZGPAT	-0.1075	0.4738	zinc finger, CCCH-type with G patch domain
8771	WDR25	-0.1075	0.5302	WD repeat domain 25
8772	WARS2	-0.1075	0.5396	tryptophanyl tRNA synthetase 2, mitochondrial
8773	SPSB3	-0.1075	0.503	splA/ryanodine receptor domain and SOCS box containing 3
8774	SPAG1	-0.1075	0.5385	sperm associated antigen 1
8775	SERPINC1	-0.1075	0.456	serpin peptidase inhibitor, clade C (antithrombin), member 1
8776	N6AMT2	-0.1075	0.5386	N-6 adenine-specific DNA methyltransferase 2 (putative)
8777	MAGI2	-0.1075	0.5074	membrane associated guanylate kinase, WW and PDZ domain containing 2
8778	LYPD6B	-0.1075	0.472	LY6/PLAUR domain containing 6B
8779	LRRN3	-0.1075	0.5052	leucine rich repeat neuronal 3
8780	HLA-DOB	-0.1075	0.4657	major histocompatibility complex, class II, DO beta
8781	HIST1H1D	-0.1075	0.5082	histone cluster 1, H1d
8782	GUCY1B2	-0.1075	0.4582	guanylate cyclase 1, soluble, beta 2 (pseudogene)
8783	FAM189B	-0.1075	0.5032	family with sequence similarity 189, member B
8784	EMC3	-0.1075	0.5427	ER membrane protein complex subunit 3
8785	COMMD5	-0.1075	0.4903	COMM domain containing 5
8786	CBFB	-0.1075	0.5329	core-binding factor, beta subunit
8787	C9orf69	-0.1075	0.5239	chromosome 9 open reading frame 69
8788	ANKRD2	-0.1075	0.4528	ankyrin repeat domain 2 (stretch responsive muscle)
8789	ALB	-0.1075	0.4412	albumin
8790	ABCB11	-0.1075	0.4529	ATP-binding cassette, sub-family B (MDR/TAP), member 11
8791	STK19	-0.1083	0.5323	serine/threonine kinase 19
8792	STAT4	-0.1083	0.5153	signal transducer and activator of transcription 4
8793	RTDR1	-0.1083	0.4546	rhabdoid tumor deletion region gene 1
8794	OR1F1	-0.1083	0.4477	olfactory receptor, family 1, subfamily F, member 1
8795	NLRP2	-0.1083	0.5598	NLR family, pyrin domain containing 2
8796	MRPL13	-0.1083	0.5113	mitochondrial ribosomal protein L13
8797	CSF1R	-0.1083	0.4678	colony stimulating factor 1 receptor
8798	TRAPPC5	-0.1088	0.4967	trafficking protein particle complex 5
8799	SDE2	-0.1088	0.5383	SDE2 telomere maintenance homolog (S. pombe)
8800	ZNF302	-0.1092	0.4707	zinc finger protein 302
8801	SLC22A14	-0.1092	0.5611	solute carrier family 22, member 14
8802	SHC3	-0.1092	0.5122	SHC (Src homology 2 domain containing) transforming protein 3
8803	NDUFB1	-0.1092	0.4929	NADH dehydrogenase (ubiquinone) 1 beta subcomplex, 1, 7kDa
8804	NCS1	-0.1092	0.4699	neuronal calcium sensor 1
8805	LTN1	-0.1092	0.4697	listerin E3 ubiquitin protein ligase 1
8806	GJA8	-0.1092	0.5515	gap junction protein, alpha 8, 50kDa
8807	DNASE1L2	-0.1092	0.5352	deoxyribonuclease I-like 2
8808	CEACAM8	-0.1092	0.5574	carcinoembryonic antigen-related cell adhesion molecule 8
8809	TEX11	-0.11	0.5407	testis expressed 11
8810	SOAT1	-0.11	0.4699	sterol O-acyltransferase 1
8811	RRAGA	-0.11	0.4605	Ras-related GTP binding A
8812	NUDT7	-0.11	0.4606	nudix (nucleoside diphosphate linked moiety X)-type motif 7
8813	MYEOV	-0.11	0.566	myeloma overexpressed (in a subset of t(11;14) positive multiple myelomas)
8814	MSLN	-0.11	0.5239	mesothelin
8815	LOC51145	-0.11	0.5538	erythrocyte transmembrane protein
8816	FAM89A	-0.11	0.4909	family with sequence similarity 89, member A
8817	F7	-0.11	0.5556	coagulation factor VII (serum prothrombin conversion accelerator)
8818	EIF2S2	-0.11	0.4648	eukaryotic translation initiation factor 2, subunit 2 beta, 38kDa

8819	CLCN7	-0.11	0.4748	chloride channel, voltage-sensitive 7
8820	CD180	-0.11	0.5218	CD180 molecule
8821	CCL17	-0.11	0.5483	chemokine (C-C motif) ligand 17
8822	CABYR	-0.11	0.4896	calcium binding tyrosine-(Y)-phosphorylation regulated
8823	C5orf51	-0.11	0.4441	chromosome 5 open reading frame 51
8824	TXN2	-0.1108	0.4703	thioredoxin 2
8825	SLCO1B1	-0.1108	0.5548	solute carrier organic anion transporter family, member 1B1
8826	S100A12	-0.1108	0.5428	S100 calcium binding protein A12
8827	CDX4	-0.1108	0.5454	caudal type homeobox 4
8828	C22orf24	-0.1108	0.5374	chromosome 22 open reading frame 24
8829	ATP11B	-0.1108	0.4724	ATPase, class VI, type 11B
8830	APOF	-0.1108	0.5536	apolipoprotein F
8831	ZNF823	-0.1112	0.4468	zinc finger protein 823
8832	SLC13A5	-0.1112	0.5673	solute carrier family 13 (sodium-dependent citrate transporter), member 5
8833	POLN	-0.1112	0.5091	polymerase (DNA directed) nu
8834	METTL21A	-0.1112	0.483	methyltransferase like 21A
8835	BEND6	-0.1112	0.4627	BEN domain containing 6
8836	DIRC3	-0.1114	0.5464	disrupted in renal carcinoma 3
8837	ZMAT5	-0.1117	0.4976	zinc finger, matrin-type 5
8838	VPS11	-0.1117	0.4188	vacuolar protein sorting 11 homolog (S. cerevisiae)
8839	SCO2	-0.1117	0.5227	SCO cytochrome oxidase deficient homolog 2 (yeast)
8840	ROBO1	-0.1117	0.456	roundabout, axon guidance receptor, homolog 1 (Drosophila)
8841	PRKCQ	-0.1117	0.4816	protein kinase C, theta
8842	PRDM11	-0.1117	0.5385	PR domain containing 11
8843	NBR2	-0.1117	0.4542	neighbor of BRCA1 gene 2 (non-protein coding)
8844	HIST1H2AL	-0.1117	0.4769	histone cluster 1, H2al
8845	FPR1	-0.1117	0.5317	formyl peptide receptor 1
8846	CSPP1	-0.1117	0.4573	centrosome and spindle pole associated protein 1
8847	CAMK2N1	-0.1117	0.4476	calcium/calmodulin-dependent protein kinase II inhibitor 1
8848	THRSP	-0.1125	0.5574	thyroid hormone responsive
8849	SDHAF2	-0.1125	0.4449	succinate dehydrogenase complex assembly factor 2
8850	PMS2P1	-0.1125	0.4556	postmeiotic segregation increased 2 pseudogene 1
8851	PCDHB11	-0.1125	0.5059	protocadherin beta 11
8852	PARP11	-0.1125	0.4449	poly (ADP-ribose) polymerase family, member 11
8853	GRID2	-0.1125	0.5479	glutamate receptor, ionotropic, delta 2
8854	GPHN	-0.1125	0.4513	gephyrin
8855	GFM2	-0.1125	0.4554	G elongation factor, mitochondrial 2
8856	GABBR2	-0.1125	0.5212	gamma-aminobutyric acid (GABA) B receptor, 2
8857	FN3K	-0.1125	0.521	fructosamine 3 kinase
8858	DUSP9	-0.1125	0.5371	dual specificity phosphatase 9
8859	DDRGK1	-0.1125	0.4566	DDRGK domain containing 1
8860	CYP26A1	-0.1125	0.5409	cytochrome P450, family 26, subfamily A, polypeptide 1
8861	CRY1	-0.1125	0.4636	cryptochrome 1 (photolyase-like)
8862	CPNE7	-0.1125	0.5339	copine VII
8863	CNTD2	-0.1125	0.5465	cyclin N-terminal domain containing 2
8864	BAZ2A	-0.1125	0.4544	bromodomain adjacent to zinc finger domain, 2A
8865	ATP6V1D	-0.1125	0.4607	ATPase, H+ transporting, lysosomal 34kDa, V1 subunit D
8866	ARMC7	-0.1125	0.4814	armadillo repeat containing 7
8867	HNRNPA3P1	-0.1127	0.5123	heterogeneous nuclear ribonucleoprotein A3 pseudogene 1
8868	ZNF516	-0.1129	0.4891	zinc finger protein 516
8869	TMEM156	-0.1133	0.4676	transmembrane protein 156
8870	SRSF6	-0.1133	0.4658	serine/arginine-rich splicing factor 6
8871	SHH	-0.1133	0.5371	sonic hedgehog
8872	PLEKHB2	-0.1133	0.4611	pleckstrin homology domain containing, family B (evectins) member 2
8873	NAA11	-0.1133	0.5641	N(alpha)-acetyltransferase 11, NatA catalytic subunit
8874	INTS5	-0.1133	0.4968	integrator complex subunit 5
8875	EAPP	-0.1133	0.4663	E2F-associated phosphoprotein
8876	ATP5L	-0.1133	0.484	ATP synthase, H+ transporting, mitochondrial Fo complex, subunit G
8877	KRT25	-0.1138	0.5911	keratin 25
8878	EXOC3L1	-0.1138	0.5693	exocyst complex component 3-like 1
8879	C20orf160	-0.1138	0.5667	chromosome 20 open reading frame 160
8880	SIDT1	-0.1142	0.4911	SID1 transmembrane family, member 1
8881	SEC14L3	-0.1142	0.5534	SEC14-like 3 (S. cerevisiae)
8882	MXD1	-0.1142	0.4747	MAX dimerization protein 1
8883	MCCC1	-0.1142	0.4515	methylcrotonoyl-CoA carboxylase 1 (alpha)
8884	CDKL2	-0.1142	0.5043	cyclin-dependent kinase-like 2 (CDC2-related kinase)
8885	SCHIP1	-0.1143	0.4796	schwannomin interacting protein 1
8886	ZNF512	-0.115	0.4309	zinc finger protein 512
8887	ZNF395	-0.115	0.4675	zinc finger protein 395
8888	TSPYL5	-0.115	0.4509	TSPY-like 5
8889	TM6SF1	-0.115	0.4838	transmembrane 6 superfamily member 1
8890	RNF24	-0.115	0.4509	ring finger protein 24
8891	PMCH	-0.115	0.4966	pro-melanin-concentrating hormone
8892	PLD5	-0.115	0.5574	phospholipase D family, member 5
8893	GCN1L1	-0.115	0.4617	GCN1 general control of amino-acid synthesis 1-like 1 (yeast)
8894	FECH	-0.115	0.4459	ferrochelatase
8895	FAM163A	-0.115	0.5458	family with sequence similarity 163, member A
8896	RPS11	-0.1158	0.4704	ribosomal protein S11
8897	POLH	-0.1158	0.4238	polymerase (DNA directed), eta
8898	NRIP2	-0.1158	0.5342	nuclear receptor interacting protein 2
8899	MMADHC	-0.1158	0.4767	methylmalonic aciduria (cobalamin deficiency) cblD type, with homocystinuria
8900	GRIK4	-0.1158	0.5322	glutamate receptor, ionotropic, kainate 4
8901	DOCK10	-0.1158	0.4673	dedicator of cytokinesis 10
8902	MRO	-0.1163	0.5405	maestro

8903	JMY	-0.1163	0.4639	junction mediating and regulatory protein, p53 cofactor
8904	APOPT1	-0.1163	0.4458	apoptogenic 1, mitochondrial
8905	VPS37B	-0.1167	0.4659	vacuolar protein sorting 37 homolog B (S. cerevisiae)
8906	SEMA6A	-0.1167	0.4807	sema domain, transmembrane domain (TM), and cytoplasmic domain, (semaphorin) 6A
8907	PLA2G2D	-0.1167	0.5547	phospholipase A2, group IID
8908	GIMAP6	-0.1167	0.5139	GTPase, IMAP family member 6
8909	GID4	-0.1167	0.4396	GID complex subunit 4, VID24 homolog (S. cerevisiae)
8910	FA2H	-0.1167	0.5141	fatty acid 2-hydroxylase
8911	EIF6	-0.1167	0.4793	eukaryotic translation initiation factor 6
8912	CCL18	-0.1167	0.5121	chemokine (C-C motif) ligand 18 (pulmonary and activation-regulated)
8913	ZNF503-AS2	-0.1171	0.5408	ZNF503 antisense RNA 2 (non-protein coding)
8914	RPLP1	-0.1171	0.4944	ribosomal protein, large, P1
8915	ZNF441	-0.1175	0.4617	zinc finger protein 441
8916	ZNF211	-0.1175	0.4474	zinc finger protein 211
8917	ZHX3	-0.1175	0.4174	zinc fingers and homeoboxes 3
8918	TUBA8	-0.1175	0.5265	tubulin, alpha 8
8919	TRIB3	-0.1175	0.4895	tribbles homolog 3 (Drosophila)
8920	MYEOV2	-0.1175	0.5055	myeloma overexpressed 2
8921	MTMR1	-0.1175	0.4515	myotubularin related protein 1
8922	KRT77	-0.1175	0.5894	keratin 77
8923	KRT36	-0.1175	0.5428	keratin 36
8924	ENPP2	-0.1175	0.4566	ectonucleotide pyrophosphatase/phosphodiesterase 2
8925	DGKG	-0.1175	0.5115	diacylglycerol kinase, gamma 90kDa
8926	CBX8	-0.1175	0.5193	chromobox homolog 8
8927	CABP2	-0.1175	0.5497	calcium binding protein 2
8928	C6orf203	-0.1175	0.4632	chromosome 6 open reading frame 203
8929	ATPAF2	-0.1175	0.4539	ATP synthase mitochondrial F1 complex assembly factor 2
8930	ALG6	-0.1175	0.4623	asparagine-linked glycosylation 6, alpha-1,3-glucosyltransferase homolog (S. cerevisiae)
8931	AKAP14	-0.1175	0.577	A kinase (PRKA) anchor protein 14
8932	TMEM74B	-0.1183	0.5195	transmembrane protein 74B
8933	REG1B	-0.1183	0.5397	regenerating islet-derived 1 beta
8934	PDE2A	-0.1183	0.4968	phosphodiesterase 2A, cGMP-stimulated
8935	NAT9	-0.1183	0.4614	N-acetyltransferase 9 (GCN5-related, putative)
8936	KIAA0913	-0.1183	0.4799	KIAA0913
8937	KCMF1	-0.1183	0.4609	potassium channel modulatory factor 1
8938	CDHR2	-0.1183	0.5495	cadherin-related family member 2
8939	CCDC92	-0.1183	0.4365	coiled-coil domain containing 92
8940	ASB4	-0.1183	0.5354	ankyrin repeat and SOCS box containing 4
8941	PLCXD3	-0.1187	0.5152	phosphatidylinositol-specific phospholipase C, X domain containing 3
8942	OR8B8	-0.1187	0.5753	olfactory receptor, family 8, subfamily B, member 8
8943	LYZL4	-0.1187	0.5789	lysozyme-like 4
8944	LEO1	-0.1187	0.4516	Leo1, Paf1/RNA polymerase II complex component, homolog (S. cerevisiae)
8945	GLB1L3	-0.1187	0.5662	galactosidase, beta 1-like 3
8946	BCO2	-0.1187	0.5054	beta-carotene oxygenase 2
8947	VPS45	-0.1192	0.4499	vacuolar protein sorting 45 homolog (S. cerevisiae)
8948	TEX264	-0.1192	0.489	testis expressed 264
8949	SLC18A1	-0.1192	0.549	solute carrier family 18 (vesicular monoamine), member 1
8950	GAPVD1	-0.1192	0.4491	GTPase activating protein and VPS9 domains 1
8951	C9orf9	-0.1192	0.4794	chromosome 9 open reading frame 9
8952	ZNF513	-0.12	0.5172	zinc finger protein 513
8953	USP25	-0.12	0.4589	ubiquitin specific peptidase 25
8954	TXNDC17	-0.12	0.4736	thioredoxin domain containing 17
8955	TSGA10IP	-0.12	0.5708	testis specific, 10 interacting protein
8956	TPR	-0.12	0.4635	translocated promoter region, nuclear basket protein
8957	TAF15	-0.12	0.4403	TAF15 RNA polymerase II, TATA box binding protein (TBP)-associated factor, 68kDa
8958	PWWP2A	-0.12	0.4849	PWWP domain containing 2A
8959	PSME2	-0.12	0.4643	proteasome (prosome, macropain) activator subunit 2 (PA28 beta)
8960	PEG3	-0.12	0.4812	paternally expressed 3
8961	MTTP	-0.12	0.5154	microsomal triglyceride transfer protein
8962	MPC1	-0.12	0.4692	mitochondrial pyruvate carrier 1
8963	LONRF2	-0.12	0.4968	LON peptidase N-terminal domain and ring finger 2
8964	LOC440981	-0.12	0.9999	phospholipid scramblase-like
8965	IPO11	-0.12	0.4563	importin 11
8966	IDI2	-0.12	0.5638	isopentenyl-diphosphate delta isomerase 2
8967	FKBP1A	-0.12	0.4563	FK506 binding protein 1A, 12kDa
8968	FAM19A1	-0.12	0.5484	family with sequence similarity 19 (chemokine (C-C motif)-like), member A1
8969	CLDN14	-0.12	0.5364	claudin 14
8970	C6orf1	-0.12	0.5018	chromosome 6 open reading frame 1
8971	TSC1	-0.1208	0.4536	tuberous sclerosis 1
8972	PNLIP	-0.1208	0.539	pancreatic lipase
8973	NPY	-0.1208	0.5404	neuropeptide Y
8974	MAP9	-0.1208	0.4541	microtubule-associated protein 9
8975	EHBP1L1	-0.1208	0.4876	EH domain binding protein 1-like 1
8976	DRD3	-0.1208	0.5532	dopamine receptor D3
8977	DAAM1	-0.1208	0.4537	dishevelled associated activator of morphogenesis 1
8978	CYP3A43	-0.1208	0.5468	cytochrome P450, family 3, subfamily A, polypeptide 43
8979	CLPX	-0.1208	0.4698	ClpX caseinolytic peptidase X homolog (E. coli)
8980	CDV3	-0.1208	0.4738	CDV3 homolog (mouse)
8981	BLOC1S1	-0.1208	0.4712	biogenesis of lysosomal organelles complex-1, subunit 1
8982	OR7D2	-0.1213	0.583	olfactory receptor, family 7, subfamily D, member 2
8983	NAF1	-0.1213	0.4582	nuclear assembly factor 1 homolog (S. cerevisiae)
8984	GPCPD1	-0.1213	0.4627	glycerophosphocholine phosphodiesterase GDE1 homolog (S. cerevisiae)
8985	GBP4	-0.1213	0.4804	guanylate binding protein 4
8986	RPL24	-0.1214	0.4959	ribosomal protein L24

8987	USP29	-0.1217	0.5534	ubiquitin specific peptidase 29
8988	SHOX2	-0.1217	0.5224	short stature homeobox 2
8989	PRMT2	-0.1217	0.4235	protein arginine methyltransferase 2
8990	MFAP1	-0.1217	0.4584	microfibrillar-associated protein 1
8991	GRIN2B	-0.1217	0.5305	glutamate receptor, ionotropic, N-methyl D-aspartate 2B
8992	NACC2	-0.1222	0.4869	NACC family member 2, BEN and BTB (POZ) domain containing
8993	ZNF415	-0.1225	0.4471	zinc finger protein 415
8994	WFDC12	-0.1225	0.5735	WAP four-disulfide core domain 12
8995	TRAPPC8	-0.1225	0.4591	trafficking protein particle complex 8
8996	MBL2	-0.1225	0.5619	mannose-binding lectin (protein C) 2, soluble
8997	LGI4	-0.1225	0.5336	leucine-rich repeat LGI family, member 4
8998	IL12A	-0.1225	0.4673	interleukin 12A (natural killer cell stimulatory factor 1, cytotoxic lymphocyte maturation factor 1, p35)
8999	DPP10	-0.1225	0.5359	dipeptidyl-peptidase 10 (non-functional)
9000	DHX40	-0.1225	0.4609	DEAH (Asp-Glu-Ala-His) box polypeptide 40
9001	CNGA3	-0.1225	0.5443	cyclic nucleotide gated channel alpha 3
9002	ARHGEF7	-0.1225	0.4522	Rho guanine nucleotide exchange factor (GEF) 7
9003	ARHGAP11A	-0.1225	0.4803	Rho GTPase activating protein 11A
9004	PAN3-AS1	-0.1229	0.9999	PAN3 antisense RNA 1 (non-protein coding)
9005	STOML1	-0.1233	0.4752	stomatin (EPB72)-like 1
9006	PYCRL	-0.1233	0.4931	pyrroline-5-carboxylate reductase-like
9007	LRRC23	-0.1233	0.4657	leucine rich repeat containing 23
9008	KLK14	-0.1233	0.5523	kallikrein-related peptidase 14
9009	KBTBD4	-0.1233	0.4569	kelch repeat and BTB (POZ) domain containing 4
9010	EPB42	-0.1233	0.5509	erythrocyte membrane protein band 4.2
9011	CACNB3	-0.1233	0.4687	calcium channel, voltage-dependent, beta 3 subunit
9012	AVPR1B	-0.1233	0.5408	arginine vasopressin receptor 1B
9013	AP4M1	-0.1233	0.4449	adaptor-related protein complex 4, mu 1 subunit
9014	UCMA	-0.1238	0.5877	upper zone of growth plate and cartilage matrix associated
9015	SYT12	-0.1238	0.514	synaptotagmin XII
9016	PLIN5	-0.1238	0.5334	perilipin 5
9017	NANOS1	-0.1238	0.5207	nanos homolog 1 (Drosophila)
9018	LOC100128893	-0.1238	0.5122	uncharacterized LOC100128893
9019	DOK7	-0.1238	0.5561	docking protein 7
9020	TXNL1	-0.1242	0.4648	thioredoxin-like 1
9021	TBC1D29	-0.1242	0.5397	TBC1 domain family, member 29
9022	RRH	-0.1242	0.5276	retinal pigment epithelium-derived rhodopsin homolog
9023	NT5DC2	-0.1242	0.4585	5'-nucleotidase domain containing 2
9024	NPFF	-0.1242	0.5019	neuropeptide FF-amide peptide precursor
9025	ATXN3L	-0.1242	0.5368	ataxin 3-like
9026	MUC5AC	-0.1243	0.9999	mucin 5AC, oligomeric mucus/gel-forming
9027	TTTY11	-0.125	0.558	testis-specific transcript, Y-linked 11 (non-protein coding)
9028	TMEM182	-0.125	0.4551	transmembrane protein 182
9029	STMN2	-0.125	0.5181	stathmin-like 2
9030	PVALB	-0.125	0.5083	parvalbumin
9031	PPP2R1A	-0.125	0.4532	protein phosphatase 2, regulatory subunit A, alpha
9032	PILRA	-0.125	0.5326	paired immunoglobin-like type 2 receptor alpha
9033	HIST1H2BI	-0.125	0.472	histone cluster 1, H2bi
9034	GBAS	-0.125	0.4685	glioblastoma amplified sequence
9035	FEZ2	-0.125	0.4526	fasciculation and elongation protein zeta 2 (zygin II)
9036	ENTPD4	-0.125	0.3866	ectonucleoside triphosphate diphosphohydrolase 4
9037	CDKN2AIP	-0.125	0.4625	CDKN2A interacting protein
9038	C2orf76	-0.125	0.4605	chromosome 2 open reading frame 76
9039	C2CD4A	-0.125	0.5987	C2 calcium-dependent domain containing 4A
9040	CHEK2	-0.1257	0.4743	checkpoint kinase 2
9041	SPIB	-0.1258	0.5307	Spi-B transcription factor (Spi-1/PU.1 related)
9042	PRG3	-0.1258	0.5524	proteoglycan 3
9043	NDUFB11	-0.1258	0.4766	NADH dehydrogenase (ubiquinone) 1 beta subcomplex, 11, 17.3kDa
9044	MTNR1B	-0.1258	0.5435	melatonin receptor 1B
9045	MAP2	-0.1258	0.4373	microtubule-associated protein 2
9046	DESI2	-0.1258	0.4612	desumoylating isopeptidase 2
9047	CDC42EP3	-0.1258	0.4473	CDC42 effector protein (Rho GTPase binding) 3
9048	CCR8	-0.1258	0.5225	chemokine (C-C motif) receptor 8
9049	AGBL2	-0.1258	0.4787	ATP/GTP binding protein-like 2
9050	XPR1	-0.1262	0.4306	xenotropic and polytropic retrovirus receptor 1
9051	SRCIN1	-0.1262	0.5563	SRC kinase signaling inhibitor 1
9052	PRADC1	-0.1262	0.5015	protease-associated domain containing 1
9053	PLIN4	-0.1262	0.5499	perilipin 4
9054	FCRL1	-0.1262	0.5724	Fc receptor-like 1
9055	ATE1	-0.1262	0.4304	arginyltransferase 1
9056	AK8	-0.1262	0.5303	adenylate kinase 8
9057	ZNHIT2	-0.1267	0.4938	zinc finger, HIT-type containing 2
9058	ZNF193	-0.1267	0.4295	zinc finger protein 193
9059	PRRG3	-0.1267	0.5317	proline rich Gla (G-carboxyglutamic acid) 3 (transmembrane)
9060	MLLT11	-0.1267	0.4459	myeloid/lymphoid or mixed-lineage leukemia (trithorax homolog, Drosophila); translocated to, 11
9061	KIRREL3	-0.1267	0.579	kin of IRRE like 3 (Drosophila)
9062	KIAA1009	-0.1267	0.4452	KIAA1009
9063	HSF2BP	-0.1267	0.4976	heat shock transcription factor 2 binding protein
9064	CHRNB3	-0.1267	0.5431	cholinergic receptor, nicotinic, beta 3 (neuronal)
9065	MARCH1	-0.1271	0.4894	membrane-associated ring finger (C3HC4) 1, E3 ubiquitin protein ligase
9066	HOXA-AS2	-0.1271	0.5338	HOXA cluster antisense RNA 2 (non-protein coding)
9067	ZNF169	-0.1275	0.5188	zinc finger protein 169
9068	ZER1	-0.1275	0.4443	zer-1 homolog (C. elegans)
9069	TSR3	-0.1275	0.4701	TSR3, 20S rRNA accumulation, homolog (S. cerevisiae)
9070	TBK1	-0.1275	0.456	TANK-binding kinase 1

9071	SLC35A5	-0.1275	0.455	solute carrier family 35, member A5
9072	SLC25A46	-0.1275	0.4679	solute carrier family 25, member 46
9073	RND1	-0.1275	0.488	Rho family GTPase 1
9074	PBOV1	-0.1275	0.5494	prostate and breast cancer overexpressed 1
9075	PALM3	-0.1275	0.5499	paralemmin 3
9076	ENPP4	-0.1275	0.4658	ectonucleotide pyrophosphatase/phosphodiesterase 4 (putative)
9077	E2F5	-0.1275	0.4538	E2F transcription factor 5, p130-binding
9078	DSCR10	-0.1275	0.5695	Down syndrome critical region gene 10 (non-protein coding)
9079	DRAP1	-0.1275	0.4623	DR1-associated protein 1 (negative cofactor 2 alpha)
9080	DCP1B	-0.1275	0.4304	DCP1 decapping enzyme homolog B (S. cerevisiae)
9081	C5orf22	-0.1275	0.4567	chromosome 5 open reading frame 22
9082	USP46	-0.1283	0.455	ubiquitin specific peptidase 46
9083	PQBP1	-0.1283	0.4749	polyglutamine binding protein 1
9084	PDX1	-0.1283	0.5376	pancreatic and duodenal homeobox 1
9085	LOC541472	-0.1283	0.9996	uncharacterized LOC541472
9086	JAM3	-0.1283	0.4435	junctional adhesion molecule 3
9087	GPR18	-0.1283	0.5013	G protein-coupled receptor 18
9088	GIPR	-0.1283	0.5392	gastric inhibitory polypeptide receptor
9089	FOXP3	-0.1283	0.5319	forkhead box P3
9090	ENTPD6	-0.1283	0.4489	ectonucleoside triphosphate diphosphohydrolase 6 (putative)
9091	DSTYK	-0.1283	0.4266	dual serine/threonine and tyrosine protein kinase
9092	CYP27A1	-0.1283	0.4591	cytochrome P450, family 27, subfamily A, polypeptide 1
9093	CRYGB	-0.1283	0.527	crystallin, gamma B
9094	SLC25A34	-0.1286	0.5503	solute carrier family 25, member 34
9095	TMEM196	-0.1287	0.564	transmembrane protein 196
9096	ING5	-0.1287	0.4818	inhibitor of growth family, member 5
9097	HS6ST2	-0.1287	0.4793	heparan sulfate 6-O-sulfotransferase 2
9098	ERCC6L2	-0.1287	0.4207	excision repair cross-complementing rodent repair deficiency, complementation group 6-like 2
9099	UPF2	-0.1292	0.4571	UPF2 regulator of nonsense transcripts homolog (yeast)
9100	TXNRD1	-0.1292	0.4606	thioredoxin reductase 1
9101	SPO11	-0.1292	0.5386	SPO11 meiotic protein covalently bound to DSB homolog (S. cerevisiae)
9102	POLDIP2	-0.1292	0.4478	polymerase (DNA-directed), delta interacting protein 2
9103	PKIA	-0.1292	0.4591	protein kinase (cAMP-dependent, catalytic) inhibitor alpha
9104	MAP3K3	-0.1292	0.4438	mitogen-activated protein kinase kinase kinase 3
9105	KLRG1	-0.1292	0.4479	killer cell lectin-like receptor subfamily G, member 1
9106	HPX	-0.1292	0.5268	hemopexin
9107	HPR	-0.1292	0.5226	haptoglobin-related protein
9108	CLDN6	-0.1292	0.5189	claudin 6
9109	C5orf54	-0.1292	0.454	chromosome 5 open reading frame 54
9110	ZNF362	-0.13	0.4407	zinc finger protein 362
9111	YWHAG	-0.13	0.4645	tyrosine 3-monooxygenase/tryptophan 5-monooxygenase activation protein, gamma polypeptide
9112	THAP10	-0.13	0.4618	THAP domain containing 10
9113	SLC25A17	-0.13	0.4553	solute carrier family 25 (mitochondrial carrier; peroxisomal membrane protein, 34kDa), member 17
9114	SCCPDH	-0.13	0.4438	saccharopine dehydrogenase (putative)
9115	LSM14A	-0.13	0.4608	LSM14A, SCD6 homolog A (S. cerevisiae)
9116	HEXDC	-0.13	0.5118	hexosaminidase (glycosyl hydrolase family 20, catalytic domain) containing
9117	GORASP2	-0.13	0.452	golgi reassembly stacking protein 2, 55kDa
9118	FCGR2B	-0.13	0.488	Fc fragment of IgG, low affinity IIb, receptor (CD32)
9119	FAM18B2	-0.13	0.5181	family with sequence similarity 18, member B2
9120	DNAH3	-0.13	0.5395	dynein, axonemal, heavy chain 3
9121	CAMSAP3	-0.13	0.523	calmodulin regulated spectrin-associated protein family, member 3
9122	ZFHX4	-0.1308	0.4719	zinc finger homeobox 4
9123	PIP5K1B	-0.1308	0.4399	phosphatidylinositol-4-phosphate 5-kinase, type I, beta
9124	PCSK2	-0.1308	0.5194	proprotein convertase subtilisin/kexin type 2
9125	MSRB1	-0.1308	0.4662	methionine sulfoxide reductase B1
9126	KIF5B	-0.1308	0.4643	kinesin family member 5B
9127	GJC1	-0.1308	0.4475	gap junction protein, gamma 1, 45kDa
9128	CREBBP	-0.1308	0.4371	CREB binding protein
9129	AIFM1	-0.1308	0.464	apoptosis-inducing factor, mitochondrion-associated, 1
9130	ZSCAN29	-0.1312	0.4391	zinc finger and SCAN domain containing 29
9131	MOB3A	-0.1312	0.4583	MOB kinase activator 3A
9132	CRLS1	-0.1312	0.4562	cardiolipin synthase 1
9133	THTPA	-0.1317	0.4281	thiamine triphosphatase
9134	ST3GAL6	-0.1317	0.4485	ST3 beta-galactoside alpha-2,3-sialyltransferase 6
9135	PKIG	-0.1317	0.437	protein kinase (cAMP-dependent, catalytic) inhibitor gamma
9136	OTUD7B	-0.1317	0.4322	OTU domain containing 7B
9137	MICU1	-0.1317	0.413	mitochondrial calcium uptake 1
9138	KIAA2013	-0.1317	0.5235	KIAA2013
9139	ANO2	-0.1317	0.5055	anoctamin 2
9140	TOR1AIP2	-0.1322	0.4157	torsin A interacting protein 2
9141	ZC2HC1C	-0.1325	0.4784	zinc finger, C2HC-type containing 1C
9142	TMEM57	-0.1325	0.4418	transmembrane protein 57
9143	TLE1	-0.1325	0.3775	transducin-like enhancer of split 1 (E(sp1) homolog, Drosophila)
9144	SOX6	-0.1325	0.5072	SRY (sex determining region Y)-box 6
9145	SLC5A9	-0.1325	0.5525	solute carrier family 5 (sodium/glucose cotransporter), member 9
9146	PDE9A	-0.1325	0.4207	phosphodiesterase 9A
9147	NAPA	-0.1325	0.4414	N-ethylmaleimide-sensitive factor attachment protein, alpha
9148	MRPL55	-0.1325	0.5074	mitochondrial ribosomal protein L55
9149	HGC6.3	-0.1325	0.5449	uncharacterized LOC100128124
9150	GLYATL1	-0.1325	0.5307	glycine-N-acyltransferase-like 1
9151	FOXA2	-0.1325	0.5399	forkhead box A2
9152	CXorf21	-0.1325	0.4668	chromosome X open reading frame 21
9153	ANXA6	-0.1325	0.4215	annexin A6
9154	ACOXL	-0.1327	0.5344	acyl-CoA oxidase-like

9155	RDH14	-0.1329	0.4796	retinol dehydrogenase 14 (all-trans/9-cis/11-cis)
9156	PPP1R27	-0.1329	0.5722	protein phosphatase 1, regulatory subunit 27
9157	DKFZp434J0226	-0.1329	0.9996	uncharacterized LOC93429
9158	TAS2R9	-0.1333	0.5246	taste receptor, type 2, member 9
9159	NKX6-1	-0.1333	0.5416	NK6 homeobox 1
9160	CYTH1	-0.1333	0.4176	cytohesin 1
9161	CRTAC1	-0.1333	0.5216	cartilage acidic protein 1
9162	CHD1	-0.1333	0.4722	chromodomain helicase DNA binding protein 1
9163	CASC1	-0.1333	0.4578	cancer susceptibility candidate 1
9164	BLK	-0.1333	0.5187	B lymphoid tyrosine kinase
9165	ARPC2	-0.1333	0.4541	actin related protein 2/3 complex, subunit 2, 34kDa
9166	USHBP1	-0.1338	0.5443	Usher syndrome 1C binding protein 1
9167	MGC16275	-0.1338	0.4715	uncharacterized protein MGC16275
9168	ACER3	-0.1338	0.434	alkaline ceramidase 3
9169	ZNHIT6	-0.1342	0.4595	zinc finger, HIT-type containing 6
9170	SNUPN	-0.1342	0.4362	snurportin 1
9171	PGLYRP1	-0.1342	0.5375	peptidoglycan recognition protein 1
9172	KHK	-0.1342	0.4858	ketohexokinase (fructokinase)
9173	CTSL2	-0.1342	0.4376	cathepsin L2
9174	CNTN1	-0.1342	0.4645	contactin 1
9175	BASP1	-0.1342	0.4522	brain abundant, membrane attached signal protein 1
9176	OR2M4	-0.1343	0.5786	olfactory receptor, family 2, subfamily M, member 4
9177	TTPAL	-0.135	0.4304	tocopherol (alpha) transfer protein-like
9178	SLC5A8	-0.135	0.5503	solute carrier family 5 (iodide transporter), member 8
9179	RNASE3	-0.135	0.5231	ribonuclease, RNase A family, 3
9180	PRRC2B	-0.135	0.4373	proline-rich coiled-coil 2B
9181	PROSER1	-0.135	0.4414	proline and serine rich 1
9182	PLK1S1	-0.135	0.452	polo-like kinase 1 substrate 1
9183	NOS3	-0.135	0.5206	nitric oxide synthase 3 (endothelial cell)
9184	KRTAP4-2	-0.135	0.5772	keratin associated protein 4-2
9185	IFNB1	-0.135	0.5266	interferon, beta 1, fibroblast
9186	HIST1H1C	-0.135	0.431	histone cluster 1, H1c
9187	FEM1C	-0.135	0.46	fem-1 homolog c (C. elegans)
9188	DOPEY1	-0.135	0.4538	dopey family member 1
9189	DMC1	-0.135	0.4727	DMC1 dosage suppressor of mck1 homolog, meiosis-specific homologous recombination (yeast)
9190	CRYGS	-0.135	0.4648	crystallin, gamma S
9191	ANAPC4	-0.135	0.4454	anaphase promoting complex subunit 4
9192	AGPHD1	-0.135	0.4355	aminoglycoside phosphotransferase domain containing 1
9193	LOC91450	-0.1357	0.9996	uncharacterized LOC91450
9194	VENTX	-0.1358	0.5212	VENT homeobox
9195	SNPH	-0.1358	0.4899	syntaphilin
9196	SLC25A11	-0.1358	0.45	solute carrier family 25 (mitochondrial carrier; oxoglutarate carrier), member 11
9197	PAPSS1	-0.1358	0.4427	3'-phosphoadenosine 5'-phosphosulfate synthase 1
9198	MRPL42	-0.1358	0.4728	mitochondrial ribosomal protein L42
9199	MADCAM1	-0.1358	0.54	mucosal vascular addressin cell adhesion molecule 1
9200	GRB10	-0.1358	0.4009	growth factor receptor-bound protein 10
9201	CTSW	-0.1358	0.509	cathepsin W
9202	CHRNA5	-0.1358	0.4528	cholinergic receptor, nicotinic, alpha 5 (neuronal)
9203	CDR2L	-0.1358	0.4667	cerebellar degeneration-related protein 2-like
9204	ZBTB45	-0.1363	0.4536	zinc finger and BTB domain containing 45
9205	TBX20	-0.1363	0.5384	T-box 20
9206	PIGW	-0.1363	0.4618	phosphatidylinositol glycan anchor biosynthesis, class W
9207	NXNL2	-0.1363	0.5692	nucleoredoxin-like 2
9208	KIAA1429	-0.1363	0.4409	KIAA1429
9209	KANK4	-0.1363	0.5301	KN motif and ankyrin repeat domains 4
9210	HEATR5B	-0.1363	0.4459	HEAT repeat containing 5B
9211	FITM1	-0.1363	0.5384	fat storage-inducing transmembrane protein 1
9212	DNM1P46	-0.1363	0.5308	DNM1 pseudogene 46
9213	RANBP2	-0.1367	0.4688	RAN binding protein 2
9214	OR12D3	-0.1367	0.5509	olfactory receptor, family 12, subfamily D, member 3
9215	LRTM1	-0.1367	0.5377	leucine-rich repeats and transmembrane domains 1
9216	FAM208B	-0.1367	0.4682	family with sequence similarity 208, member B
9217	EFCAB1	-0.1367	0.5058	EF-hand calcium binding domain 1
9218	EBI3	-0.1367	0.5183	Epstein-Barr virus induced 3
9219	CDK16	-0.1367	0.438	cyclin-dependent kinase 16
9220	CASP2	-0.1367	0.4191	caspase 2, apoptosis-related cysteine peptidase
9221	CAMLG	-0.1367	0.4538	calcium modulating ligand
9222	C19orf80	-0.1367	0.5371	chromosome 19 open reading frame 80
9223	APOBEC3G	-0.1367	0.4577	apolipoprotein B mRNA editing enzyme, catalytic polypeptide-like 3G
9224	ABCF3	-0.1367	0.4242	ATP-binding cassette, sub-family F (GCN20), member 3
9225	LOC440944	-0.1371	0.4645	uncharacterized LOC440944
9226	ZNF709	-0.1375	0.4585	zinc finger protein 709
9227	ZBTB46	-0.1375	0.502	zinc finger and BTB domain containing 46
9228	TBC1D22A	-0.1375	0.4144	TBC1 domain family, member 22A
9229	SYNDIG1	-0.1375	0.5151	synapse differentiation inducing 1
9230	SLC4A9	-0.1375	0.5681	solute carrier family 4, sodium bicarbonate cotransporter, member 9
9231	SLC22A7	-0.1375	0.547	solute carrier family 22 (organic anion transporter), member 7
9232	PLA2G4C	-0.1375	0.4254	phospholipase A2, group IVC (cytosolic, calcium-independent)
9233	PIGQ	-0.1375	0.4595	phosphatidylinositol glycan anchor biosynthesis, class Q
9234	MGAT2	-0.1375	0.4637	mannosyl (alpha-1,6-)-glycoprotein beta-1,2-N-acetylglucosaminyltransferase
9235	FAM96A	-0.1375	0.4656	family with sequence similarity 96, member A
9236	BBS5	-0.1375	0.406	Bardet-Biedl syndrome 5
9237	ADGB	-0.1375	0.5365	androglobin
9238	ZSCAN16	-0.1383	0.4464	zinc finger and SCAN domain containing 16

9239	VTI1B	-0.1383	0.4465	vesicle transport through interaction with t-SNAREs homolog 1B (yeast)
9240	UCP1	-0.1383	0.5317	uncoupling protein 1 (mitochondrial, proton carrier)
9241	SPON1	-0.1383	0.4659	spondin 1, extracellular matrix protein
9242	PKNOX2	-0.1383	0.5048	PBX/knotted 1 homeobox 2
9243	PHB2	-0.1383	0.4633	prohibitin 2
9244	NUCB2	-0.1383	0.4512	nucleobindin 2
9245	KIAA0391	-0.1383	0.4468	KIAA0391
9246	FAHD2A	-0.1383	0.4514	fumarylacetoacetate hydrolase domain containing 2A
9247	AVL9	-0.1383	0.3901	AVL9 homolog (S. cerevisiase)
9248	ZNF835	-0.1386	0.5764	zinc finger protein 835
9249	LHX8	-0.1386	0.5807	LIM homeobox 8
9250	TBC1D16	-0.1388	0.4839	TBC1 domain family, member 16
9251	SYT15	-0.1388	0.5466	synaptotagmin XV
9252	CACNG7	-0.1388	0.5566	calcium channel, voltage-dependent, gamma subunit 7
9253	ADAL	-0.1388	0.409	adenosine deaminase-like
9254	OGFOD2	-0.1392	0.475	2-oxoglutarate and iron-dependent oxygenase domain containing 2
9255	GDF2	-0.1392	0.5458	growth differentiation factor 2
9256	FGF8	-0.1392	0.54	fibroblast growth factor 8 (androgen-induced)
9257	DNAH9	-0.1392	0.5377	dynein, axonemal, heavy chain 9
9258	CLDN11	-0.1392	0.5095	claudin 11
9259	CAMK2A	-0.1392	0.5273	calcium/calmodulin-dependent protein kinase II alpha
9260	AVP	-0.1392	0.5352	arginine vasopressin
9261	ZPLD1	-0.14	0.546	zona pellucida-like domain containing 1
9262	USP38	-0.14	0.4524	ubiquitin specific peptidase 38
9263	TMCO6	-0.14	0.4692	transmembrane and coiled-coil domains 6
9264	PDP1	-0.14	0.4468	pyruvate dehyrogenase phosphatase catalytic subunit 1
9265	MLLT4	-0.14	0.4008	myeloid/lymphoid or mixed-lineage leukemia (trithorax homolog, Drosophila); translocated to, 4
9266	LUC7L2	-0.14	0.4699	LUC7-like 2 (S. cerevisiae)
9267	IL13	-0.14	0.5245	interleukin 13
9268	GIPC3	-0.14	0.5412	GIPC PDZ domain containing family, member 3
9269	TX2P1-UPK3BP1-PMS2P1	-0.14	0.4909	DTX2P1-UPK3BP1-PMS2P11 readthrough (non-protein coding)
9270	DHRS2	-0.14	0.4765	dehydrogenase/reductase (SDR family) member 2
9271	CHRND	-0.14	0.5359	cholinergic receptor, nicotinic, delta (muscle)
9272	CD5L	-0.14	0.53	CD5 molecule-like
9273	ATP5SL	-0.14	0.4386	ATP5S-like
9274	WT1-AS	-0.1408	0.5233	WT1 antisense RNA (non-protein coding)
9275	TRAF2	-0.1408	0.4745	TNF receptor-associated factor 2
9276	SLC22A17	-0.1408	0.4755	solute carrier family 22, member 17
9277	SLC1A7	-0.1408	0.5226	solute carrier family 1 (glutamate transporter), member 7
9278	RTF1	-0.1408	0.4385	Rtf1, Paf1/RNA polymerase II complex component, homolog (S. cerevisiae)
9279	EYA1	-0.1408	0.4749	eyes absent homolog 1 (Drosophila)
9280	DLGAP4	-0.1408	0.4644	discs, large (Drosophila) homolog-associated protein 4
9281	C1QA	-0.1408	0.5096	complement component 1, q subcomponent, A chain
9282	ZNF585A	-0.1412	0.4374	zinc finger protein 585A
9283	RASL10B	-0.1412	0.5345	RAS-like, family 10, member B
9284	HHATL	-0.1412	0.5534	hedgehog acyltransferase-like
9285	CCDC39	-0.1412	0.4858	coiled-coil domain containing 39
9286	C1orf168	-0.1412	0.5017	chromosome 1 open reading frame 168
9287	C14orf142	-0.1412	0.4679	chromosome 14 open reading frame 142
9288	SAMD4B	-0.1417	0.4618	sterile alpha motif domain containing 4B
9289	OR2F1	-0.1417	0.5441	olfactory receptor, family 2, subfamily F, member 1
9290	OAZ3	-0.1417	0.4877	ornithine decarboxylase antizyme 3
9291	HIST2H2BE	-0.1417	0.4176	histone cluster 2, H2be
9292	GHRH	-0.1417	0.5423	growth hormone releasing hormone
9293	DLX2	-0.1417	0.5125	distal-less homeobox 2
9294	ZBTB26	-0.1425	0.4371	zinc finger and BTB domain containing 26
9295	NXF1	-0.1425	0.4421	nuclear RNA export factor 1
9296	NPEPPS	-0.1425	0.4394	aminopeptidase puromycin sensitive
9297	NFU1	-0.1425	0.4625	NFU1 iron-sulfur cluster scaffold homolog (S. cerevisiae)
9298	NDUFS8	-0.1425	0.4824	NADH dehydrogenase (ubiquinone) Fe-S protein 8, 23kDa (NADH-coenzyme Q reductase)
9299	LMAN1L	-0.1425	0.5408	lectin, mannose-binding, 1 like
9300	HDAC3	-0.1425	0.4349	histone deacetylase 3
9301	DGAT2	-0.1425	0.4723	diacylglycerol O-acyltransferase 2
9302	C6orf25	-0.1425	0.5357	chromosome 6 open reading frame 25
9303	TMEM131	-0.1433	0.4382	transmembrane protein 131
9304	TCF7	-0.1433	0.471	transcription factor 7 (T-cell specific, HMG-box)
9305	MORC3	-0.1433	0.4647	MORC family CW-type zinc finger 3
9306	MERTK	-0.1433	0.3902	c-mer proto-oncogene tyrosine kinase
9307	HSPA1L	-0.1433	0.3833	heat shock 70kDa protein 1-like
9308	GYPB	-0.1433	0.5012	glycophorin B (MNS blood group)
9309	DCTPP1	-0.1433	0.4685	dCTP pyrophosphatase 1
9310	ADSS	-0.1433	0.4595	adenylosuccinate synthase
9311	SOCS4	-0.1437	0.4561	suppressor of cytokine signaling 4
9312	PUS10	-0.1437	0.4287	pseudouridylate synthase 10
9313	KIAA1804	-0.1437	0.4721	mixed lineage kinase 4
9314	EMX2OS	-0.1437	0.5071	EMX2 opposite strand/antisense RNA (non-protein coding)
9315	CNST	-0.1437	0.4379	consortin, connexin sorting protein
9316	VILL	-0.1442	0.4776	villin-like
9317	TRPV5	-0.1442	0.5436	transient receptor potential cation channel, subfamily V, member 5
9318	SREBF1	-0.1442	0.4337	sterol regulatory element binding transcription factor 1
9319	SMOX	-0.1442	0.4811	spermine oxidase
9320	SLC12A6	-0.1442	0.4132	solute carrier family 12 (potassium/chloride transporters), member 6
9321	RNASE6	-0.1442	0.4788	ribonuclease, RNase A family, k6
9322	PRND	-0.1442	0.5214	prion protein 2 (dublet)

9323	NBEA	-0.1442	0.4382	neurobeachin
9324	NAA38	-0.1442	0.4653	N(alpha)-acetyltransferase 38, NatC auxiliary subunit
9325	FAM69A	-0.1442	0.4383	family with sequence similarity 69, member A
9326	COX7C	-0.1442	0.4693	cytochrome c oxidase subunit VIIc
9327	ZNF564	-0.145	0.4286	zinc finger protein 564
9328	ZNF419	-0.145	0.4195	zinc finger protein 419
9329	PIGF	-0.145	0.4574	phosphatidylinositol glycan anchor biosynthesis, class F
9330	NKAIN4	-0.145	0.53	Na+/K+ transporting ATPase interacting 4
9331	KLF15	-0.145	0.5171	Kruppel-like factor 15
9332	GPRIN2	-0.145	0.5414	G protein regulated inducer of neurite outgrowth 2
9333	ZNF212	-0.1458	0.4439	zinc finger protein 212
9334	TLR8	-0.1458	0.4936	toll-like receptor 8
9335	TECTA	-0.1458	0.5002	tectorin alpha
9336	SMARCE1	-0.1458	0.4571	SWI/SNF related, matrix associated, actin dependent regulator of chromatin, subfamily e, member 1
9337	SKP2	-0.1458	0.4584	S-phase kinase-associated protein 2, E3 ubiquitin protein ligase
9338	SECISBP2	-0.1458	0.4369	SECIS binding protein 2
9339	PSMD9	-0.1458	0.434	proteasome (prosome, macropain) 26S subunit, non-ATPase, 9
9340	NOTCH1	-0.1458	0.4117	notch 1
9341	KCNE2	-0.1458	0.5185	potassium voltage-gated channel, Isk-related family, member 2
9342	HEMK1	-0.1458	0.4347	HemK methyltransferase family member 1
9343	GLUD1	-0.1458	0.4466	glutamate dehydrogenase 1
9344	FRMPD1	-0.1458	0.5126	FERM and PDZ domain containing 1
9345	DLG4	-0.1458	0.4722	discs, large homolog 4 (Drosophila)
9346	BCLAF1	-0.1458	0.4688	BCL2-associated transcription factor 1
9347	ZNF599	-0.1462	0.4573	zinc finger protein 599
9348	PCP4L1	-0.1462	0.5435	Purkinje cell protein 4 like 1
9349	KIF9	-0.1462	0.4669	kinesin family member 9
9350	FAM126A	-0.1462	0.4418	family with sequence similarity 126, member A
9351	CECR5-AS1	-0.1462	0.5342	CECR5 antisense RNA 1 (non-protein coding)
9352	VAMP1	-0.1467	0.3973	vesicle-associated membrane protein 1 (synaptobrevin 1)
9353	UAP1	-0.1467	0.4594	UDP-N-acteylglucosamine pyrophosphorylase 1
9354	TUBGCP5	-0.1467	0.4409	tubulin, gamma complex associated protein 5
9355	SGCE	-0.1467	0.4392	sarcoglycan, epsilon
9356	PSORS1C1	-0.1467	0.5083	psoriasis susceptibility 1 candidate 1
9357	PRPF18	-0.1467	0.4672	PRP18 pre-mRNA processing factor 18 homolog (S. cerevisiae)
9358	PLA2G15	-0.1467	0.4266	phospholipase A2, group XV
9359	MYOG	-0.1467	0.5406	myogenin (myogenic factor 4)
9360	LIG3	-0.1467	0.4272	ligase III, DNA, ATP-dependent
9361	HIST1H2BG	-0.1467	0.4105	histone cluster 1, H2bg
9362	CAP1	-0.1467	0.4413	CAP, adenylate cyclase-associated protein 1 (yeast)
9363	BMP10	-0.1467	0.5258	bone morphogenetic protein 10
9364	ATP2A3	-0.1467	0.4612	ATPase, Ca++ transporting, ubiquitous
9365	SLC24A2	-0.1475	0.5156	solute carrier family 24 (sodium/potassium/calcium exchanger), member 2
9366	SERPINA7	-0.1475	0.5185	serpin peptidase inhibitor, clade A (alpha-1 antiproteinase, antitrypsin), member 7
9367	NCAM1	-0.1475	0.481	neural cell adhesion molecule 1
9368	MMP9	-0.1475	0.4829	matrix metallopeptidase 9 (gelatinase B, 92kDa gelatinase, 92kDa type IV collagenase)
9369	KIAA0020	-0.1475	0.4624	KIAA0020
9370	KCNJ10	-0.1475	0.5124	potassium inwardly-rectifying channel, subfamily J, member 10
9371	ITGA2B	-0.1475	0.5236	integrin, alpha 2b (platelet glycoprotein IIb of IIb/IIIa complex, antigen CD41)
9372	HSD17B3	-0.1475	0.4964	hydroxysteroid (17-beta) dehydrogenase 3
9373	GTF2H5	-0.1475	0.4588	general transcription factor IIH, polypeptide 5
9374	DAP3	-0.1475	0.4613	death associated protein 3
9375	CLTC	-0.1475	0.4522	clathrin, heavy chain (Hc)
9376	BTBD16	-0.1475	0.5433	BTB (POZ) domain containing 16
9377	BAG1	-0.1475	0.4301	BCL2-associated athanogene
9378	APOC1	-0.1475	0.4135	apolipoprotein C-I
9379	VPS52	-0.1483	0.3727	vacuolar protein sorting 52 homolog (S. cerevisiae)
9380	FP588	-0.1483	0.4772	uncharacterized LOC92973
9381	CCDC141	-0.1483	0.4832	coiled-coil domain containing 141
9382	CASS4	-0.1483	0.506	Cas scaffolding protein family member 4
9383	ALPPL2	-0.1483	0.5354	alkaline phosphatase, placental-like 2
9384	ACTR3	-0.1483	0.4695	ARP3 actin-related protein 3 homolog (yeast)
9385	SOHLH2	-0.1486	0.4644	spermatogenesis and oogenesis specific basic helix-loop-helix 2
9386	SNORA72	-0.1486	0.9999	small nucleolar RNA, H/ACA box 72
9387	ROPN1B	-0.1486	0.4356	rhophilin associated tail protein 1B
9388	UTS2R	-0.1488	0.5521	urotensin 2 receptor
9389	LENG8	-0.1488	0.474	leukocyte receptor cluster (LRC) member 8
9390	C1orf64	-0.1488	0.5431	chromosome 1 open reading frame 64
9391	AWAT1	-0.1488	0.5624	acyl-CoA wax alcohol acyltransferase 1
9392	UGT2B4	-0.1492	0.4442	UDP glucuronosyltransferase 2 family, polypeptide B4
9393	OR3A3	-0.1492	0.5332	olfactory receptor, family 3, subfamily A, member 3
9394	OR10H1	-0.1492	0.5356	olfactory receptor, family 10, subfamily H, member 1
9395	NREP	-0.1492	0.4208	neuronal regeneration related protein homolog (rat)
9396	MYCBP2	-0.1492	0.444	MYC binding protein 2, E3 ubiquitin protein ligase
9397	MMP20	-0.1492	0.5305	matrix metallopeptidase 20
9398	IRAK1	-0.1492	0.4612	interleukin-1 receptor-associated kinase 1
9399	FBXW11	-0.1492	0.4363	F-box and WD repeat domain containing 11
9400	DNAAF1	-0.1492	0.5275	dynein, axonemal, assembly factor 1
9401	ZNF718	-0.15	0.3957	zinc finger protein 718
9402	ZFYVE16	-0.15	0.4559	zinc finger, FYVE domain containing 16
9403	UTP14A	-0.15	0.4601	UTP14, U3 small nucleolar ribonucleoprotein, homolog A (yeast)
9404	TSIX	-0.15	0.5662	TSIX transcript, XIST antisense RNA (non-protein coding)
9405	SMARCC1	-0.15	0.45	SWI/SNF related, matrix associated, actin dependent regulator of chromatin, subfamily c, member 1
9406	RBP2	-0.15	0.5528	retinol binding protein 2, cellular

9407	RAB3IL1	-0.15	0.5088	RAB3A interacting protein (rabin3)-like 1
9408	OR3A1	-0.15	0.5156	olfactory receptor, family 3, subfamily A, member 1
9409	KRTAP5-9	-0.15	0.5283	keratin associated protein 5-9
9410	KIAA1737	-0.15	0.4345	KIAA1737
9411	JHDM1D	-0.15	0.4381	jumonji C domain containing histone demethylase 1 homolog D (S. cerevisiae)
9412	HGFAC	-0.15	0.525	HGF activator
9413	FKSG29	-0.15	0.4641	FKSG29
9414	DHX58	-0.15	0.4548	DEXH (Asp-Glu-X-His) box polypeptide 58
9415	CLDN15	-0.15	0.4928	claudin 15
9416	CDC42SE2	-0.15	0.4412	CDC42 small effector 2
9417	APOE	-0.15	0.4814	apolipoprotein E
9418	ABHD6	-0.15	0.3895	abhydrolase domain containing 6
9419	UBTD1	-0.1508	0.4894	ubiquitin domain containing 1
9420	PRPF6	-0.1508	0.4246	PRP6 pre-mRNA processing factor 6 homolog (S. cerevisiae)
9421	MORN1	-0.1508	0.5121	MORN repeat containing 1
9422	MEF2D	-0.1508	0.4649	myocyte enhancer factor 2D
9423	HIF3A	-0.1508	0.5256	hypoxia inducible factor 3, alpha subunit
9424	DNAJC4	-0.1508	0.4508	DnaJ (Hsp40) homolog, subfamily C, member 4
9425	CT62	-0.1508	0.4921	cancer/testis antigen 62
9426	CDC16	-0.1508	0.4412	cell division cycle 16 homolog (S. cerevisiae)
9427	ASXL2	-0.1508	0.422	additional sex combs like 2 (Drosophila)
9428	ZNF439	-0.1513	0.4435	zinc finger protein 439
9429	NEURL3	-0.1513	0.5349	neuralized homolog 3 (Drosophila) pseudogene
9430	LRRC28	-0.1513	0.3812	leucine rich repeat containing 28
9431	LOC400099	-0.1513	0.4512	uncharacterized LOC400099
9432	HMCN2	-0.1513	0.5656	hemicentin 2
9433	GSTTP1	-0.1514	0.4798	glutathione S-transferase theta pseudogene 1
9434	TAF13	-0.1517	0.4258	TAF13 RNA polymerase II, TATA box binding protein (TBP)-associated factor, 18kDa
9435	POT1	-0.1517	0.4562	protection of telomeres 1 homolog (S. pombe)
9436	PISD	-0.1517	0.4155	phosphatidylserine decarboxylase
9437	OPN1SW	-0.1517	0.5179	opsin 1 (cone pigments), short-wave-sensitive
9438	FAM193B	-0.1517	0.4776	family with sequence similarity 193, member B
9439	DLD	-0.1517	0.4691	dihydrolipoamide dehydrogenase
9440	VIPAS39	-0.1525	0.3898	VPS33B interacting protein, apical-basolateral polarity regulator, spe-39 homolog
9441	U2SURP	-0.1525	0.4699	U2 snRNP-associated SURP domain containing
9442	SCN3A	-0.1525	0.4473	sodium channel, voltage-gated, type III, alpha subunit
9443	RHEB	-0.1525	0.448	Ras homolog enriched in brain
9444	OLFM2	-0.1525	0.4942	olfactomedin 2
9445	NLRP3	-0.1525	0.4881	NLR family, pyrin domain containing 3
9446	MRPL50	-0.1525	0.4619	mitochondrial ribosomal protein L50
9447	LOC401588	-0.1525	0.4404	uncharacterized LOC401588
9448	LILRB2	-0.1525	0.5164	leukocyte immunoglobulin-like receptor, subfamily B (with TM and ITIM domains), member 2
9449	GIMAP2	-0.1525	0.4572	GTPase, IMAP family member 2
9450	ENPP6	-0.1525	0.5127	ectonucleotide pyrophosphatase/phosphodiesterase 6
9451	ELSPBP1	-0.1525	0.532	epididymal sperm binding protein 1
9452	DNAJA2	-0.1525	0.4555	DnaJ (Hsp40) homolog, subfamily A, member 2
9453	ANKS1A	-0.1525	0.4257	ankyrin repeat and sterile alpha motif domain containing 1A
9454	SHBG	-0.1533	0.5236	sex hormone-binding globulin
9455	POU3F4	-0.1533	0.5251	POU class 3 homeobox 4
9456	PJA1	-0.1533	0.4346	praja ring finger 1, E3 ubiquitin protein ligase
9457	NDRG3	-0.1533	0.4034	NDRG family member 3
9458	CTRC	-0.1533	0.529	chymotrypsin C (caldecrin)
9459	ADAP1	-0.1533	0.4783	ArfGAP with dual PH domains 1
9460	ZIM3	-0.1538	0.5565	zinc finger, imprinted 3
9461	TYW3	-0.1538	0.4506	tRNA-yW synthesizing protein 3 homolog (S. cerevisiae)
9462	SUSD1	-0.1538	0.4021	sushi domain containing 1
9463	LMBR1	-0.1538	0.4328	limb region 1 homolog (mouse)
9464	GATAD2B	-0.1538	0.4122	GATA zinc finger domain containing 2B
9465	C9orf117	-0.1538	0.5365	chromosome 9 open reading frame 117
9466	ACY3	-0.1538	0.5188	aspartoacylase (aminocyclase) 3
9467	ZNF562	-0.1542	0.4323	zinc finger protein 562
9468	PPP1R9A	-0.1542	0.4113	protein phosphatase 1, regulatory subunit 9A
9469	NFS1	-0.1542	0.4384	NFS1 nitrogen fixation 1 homolog (S. cerevisiae)
9470	MRPS18B	-0.1542	0.4563	mitochondrial ribosomal protein S18B
9471	GRINA	-0.1542	0.4446	glutamate receptor, ionotropic, N-methyl D-aspartate-associated protein 1 (glutamate binding)
9472	GML	-0.1542	0.5328	glycosylphosphatidylinositol anchored molecule like protein
9473	FAM149A	-0.1542	0.383	family with sequence similarity 149, member A
9474	DTX3	-0.1542	0.4725	deltex homolog 3 (Drosophila)
9475	CTAGE1	-0.1542	0.5031	cutaneous T-cell lymphoma-associated antigen 1
9476	CEP70	-0.1542	0.4275	centrosomal protein 70kDa
9477	AMPD3	-0.1542	0.4166	adenosine monophosphate deaminase 3
9478	LOC339788	-0.1543	0.9999	uncharacterized LOC339788
9479	PRRT2	-0.155	0.5042	proline-rich transmembrane protein 2
9480	LMTK2	-0.155	0.3888	lemur tyrosine kinase 2
9481	KAT5	-0.155	0.4137	K(lysine) acetyltransferase 5
9482	IL18RAP	-0.155	0.4875	interleukin 18 receptor accessory protein
9483	HIST1H2AE	-0.155	0.3826	histone cluster 1, H2ae
9484	CEP120	-0.155	0.449	centrosomal protein 120kDa
9485	CDK4	-0.155	0.4543	cyclin-dependent kinase 4
9486	BAIAP2	-0.155	0.468	BAI1-associated protein 2
9487	ARHGAP20	-0.155	0.4556	Rho GTPase activating protein 20
9488	AGPAT6	-0.155	0.3618	1-acylglycerol-3-phosphate O-acyltransferase 6 (lysophosphatidic acid acyltransferase, zeta)
9489	LOC100288198	-0.1557	0.9999	uncharacterized LOC100288198
9490	RPL5	-0.1558	0.4651	ribosomal protein L5

9491	NEUROG1	-0.1558	0.5415	neurogenin 1
9492	KIF26B	-0.1558	0.4987	kinesin family member 26B
9493	COL19A1	-0.1558	0.5149	collagen, type XIX, alpha 1
9494	USP44	-0.1563	0.4195	ubiquitin specific peptidase 44
9495	TPT1-AS1	-0.1563	0.4546	TPT1 antisense RNA 1 (non-protein coding)
9496	GALP	-0.1563	0.5601	galanin-like peptide
9497	FAM124A	-0.1563	0.5222	family with sequence similarity 124A
9498	AQP11	-0.1563	0.4084	aquaporin 11
9499	RBP3	-0.1564	0.5267	retinol binding protein 3, interstitial
9500	GCKR	-0.1567	0.5118	glucokinase (hexokinase 4) regulator
9501	EIF2AK1	-0.1567	0.4168	eukaryotic translation initiation factor 2-alpha kinase 1
9502	CSF2	-0.1567	0.5052	colony stimulating factor 2 (granulocyte-macrophage)
9503	C2	-0.1567	0.4812	complement component 2
9504	ZNF788	-0.1575	0.422	zinc finger family member 788
9505	ZNF627	-0.1575	0.4395	zinc finger protein 627
9506	STOML3	-0.1575	0.5441	stomatin (EPB72)-like 3
9507	SLC10A4	-0.1575	0.4647	solute carrier family 10 (sodium/bile acid cotransporter family), member 4
9508	RAPGEF1	-0.1575	0.4032	Rap guanine nucleotide exchange factor (GEF) 1
9509	RAB3C	-0.1575	0.3902	RAB3C, member RAS oncogene family
9510	NSFL1C	-0.1575	0.4191	NSFL1 (p97) cofactor (p47)
9511	HAS1	-0.1575	0.5159	hyaluronan synthase 1
9512	EIF4G2	-0.1575	0.4532	eukaryotic translation initiation factor 4 gamma, 2
9513	EFHD2	-0.1575	0.4377	EF-hand domain family, member D2
9514	COL4A3BP	-0.1575	0.4415	collagen, type IV, alpha 3 (Goodpasture antigen) binding protein
9515	COA1	-0.1575	0.4464	cytochrome C oxidase assembly factor 1 homolog (S. cerevisiae)
9516	BBC3	-0.1575	0.5079	BCL2 binding component 3
9517	ANKDD1A	-0.1575	0.4725	ankyrin repeat and death domain containing 1A
9518	AMOTL1	-0.1575	0.3957	angiomotin like 1
9519	ZNF654	-0.1583	0.4555	zinc finger protein 654
9520	RGS6	-0.1583	0.5098	regulator of G-protein signaling 6
9521	PRKDC	-0.1583	0.4658	protein kinase, DNA-activated, catalytic polypeptide
9522	PLCH2	-0.1583	0.5113	phospholipase C, eta 2
9523	NPBWR2	-0.1583	0.5406	neuropeptides B/W receptor 2
9524	EIF3H	-0.1583	0.4538	eukaryotic translation initiation factor 3, subunit H
9525	DAZL	-0.1583	0.5027	deleted in azoospermia-like
9526	CYP11B1	-0.1583	0.5427	cytochrome P450, family 11, subfamily B, polypeptide 1
9527	CLK2	-0.1583	0.4377	CDC-like kinase 2
9528	ACAP2	-0.1583	0.4605	ArfGAP with coiled-coil, ankyrin repeat and PH domains 2
9529	LOC284551	-0.1586	0.9999	uncharacterized LOC284551
9530	LOC100216545	-0.1586	0.9999	uncharacterized LOC100216545
9531	TBRG1	-0.1587	0.4057	transforming growth factor beta regulator 1
9532	PTRHD1	-0.1587	0.4385	peptidyl-tRNA hydrolase domain containing 1
9533	ESX1	-0.1587	0.5369	ESX homeobox 1
9534	ZNF7	-0.1592	0.4424	zinc finger protein 7
9535	XPO4	-0.1592	0.4398	exportin 4
9536	SLC23A2	-0.1592	0.3632	solute carrier family 23 (nucleobase transporters), member 2
9537	HABP2	-0.1592	0.5208	hyaluronan binding protein 2
9538	GYG2	-0.1592	0.4321	glycogenin 2
9539	CHD4	-0.1592	0.4049	chromodomain helicase DNA binding protein 4
9540	ASXL1	-0.1592	0.4322	additional sex combs like 1 (Drosophila)
9541	ARNTL2	-0.1592	0.4243	aryl hydrocarbon receptor nuclear translocator-like 2
9542	ZNF460	-0.16	0.367	zinc finger protein 460
9543	ZMAT2	-0.16	0.4164	zinc finger, matrin-type 2
9544	TLL2	-0.16	0.507	tolloid-like 2
9545	RNF6	-0.16	0.4637	ring finger protein (C3H2C3 type) 6
9546	PRDM12	-0.16	0.5226	PR domain containing 12
9547	PCLO	-0.16	0.4433	piccolo (presynaptic cytomatrix protein)
9548	ONECUT1	-0.16	0.538	one cut homeobox 1
9549	NECAB2	-0.16	0.5091	N-terminal EF-hand calcium binding protein 2
9550	NCK1	-0.16	0.4566	NCK adaptor protein 1
9551	IGF2-AS	-0.16	0.5345	IGF2 antisense RNA (non-protein coding)
9552	GPR15	-0.16	0.5044	G protein-coupled receptor 15
9553	FOXN1	-0.16	0.5283	forkhead box N1
9554	EPC2	-0.16	0.4337	enhancer of polycomb homolog 2 (Drosophila)
9555	CPM	-0.16	0.3914	carboxypeptidase M
9556	ABCB4	-0.16	0.364	ATP-binding cassette, sub-family B (MDR/TAP), member 4
9557	ZNF331	-0.1608	0.4139	zinc finger protein 331
9558	RHOBTB3	-0.1608	0.4214	Rho-related BTB domain containing 3
9559	RDH8	-0.1608	0.5335	retinol dehydrogenase 8 (all-trans)
9560	PGAM2	-0.1608	0.5021	phosphoglycerate mutase 2 (muscle)
9561	HPCAL1	-0.1608	0.4266	hippocalcin-like 1
9562	FRMD1	-0.1608	0.5395	FERM domain containing 1
9563	CLK3	-0.1608	0.3954	CDC-like kinase 3
9564	CEACAM21	-0.1608	0.5051	carcinoembryonic antigen-related cell adhesion molecule 21
9565	CD3E	-0.1608	0.5166	CD3e molecule, epsilon (CD3-TCR complex)
9566	SUMO4	-0.1609	0.4634	SMT3 suppressor of mif two 3 homolog 4 (S. cerevisiae)
9567	TMEM136	-0.1612	0.3586	transmembrane protein 136
9568	NADKD1	-0.1612	0.4416	NAD kinase domain containing 1
9569	FLJ43390	-0.1612	0.51	uncharacterized LOC646113
9570	FAM198A	-0.1612	0.4974	family with sequence similarity 198, member A
9571	ELP2	-0.1612	0.4273	elongation protein 2 homolog (S. cerevisiae)
9572	DIO3OS	-0.1612	0.4996	DIO3 opposite strand/antisense RNA (non-protein coding)
9573	C1orf101	-0.1612	0.443	chromosome 1 open reading frame 101
9574	RPS4X	-0.1614	0.4417	ribosomal protein S4, X-linked

9575	ZNF671	-0.1617	0.3873	zinc finger protein 671
9576	ZNF3	-0.1617	0.3912	zinc finger protein 3
9577	PEBP1	-0.1617	0.4452	phosphatidylethanolamine binding protein 1
9578	LPA	-0.1617	0.5249	lipoprotein, Lp(a)
9579	CSF3	-0.1617	0.5257	colony stimulating factor 3 (granulocyte)
9580	CDK5RAP2	-0.1617	0.402	CDK5 regulatory subunit associated protein 2
9581	CCNT1	-0.1617	0.3694	cyclin T1
9582	BTRC	-0.1617	0.3503	beta-transducin repeat containing E3 ubiquitin protein ligase
9583	THRAP3	-0.1625	0.3714	thyroid hormone receptor associated protein 3
9584	NPM1	-0.1625	0.474	nucleophosmin (nucleolar phosphoprotein B23, numatrin)
9585	MGA	-0.1625	0.4442	MAX gene associated
9586	GAPT	-0.1625	0.4546	GRB2-binding adaptor protein, transmembrane
9587	EXD1	-0.1625	0.5407	exonuclease 3'-5' domain containing 1
9588	DICER1	-0.1625	0.4507	dicer 1, ribonuclease type III
9589	BIK	-0.1625	0.4098	BCL2-interacting killer (apoptosis-inducing)
9590	AIFM3	-0.1625	0.5166	apoptosis-inducing factor, mitochondrion-associated, 3
9591	ZNF674	-0.1633	0.3588	zinc finger protein 674
9592	ZBED5	-0.1633	0.4542	zinc finger, BED-type containing 5
9593	TTC31	-0.1633	0.4316	tetratricopeptide repeat domain 31
9594	SLC26A6	-0.1633	0.4151	solute carrier family 26, member 6
9595	RPAP3	-0.1633	0.461	RNA polymerase II associated protein 3
9596	PSG9	-0.1633	0.5129	pregnancy specific beta-1-glycoprotein 9
9597	PLA2G10	-0.1633	0.361	phospholipase A2, group X
9598	PIGZ	-0.1633	0.4257	phosphatidylinositol glycan anchor biosynthesis, class Z
9599	OXSR1	-0.1633	0.4394	oxidative-stress responsive 1
9600	MS4A3	-0.1633	0.5002	membrane-spanning 4-domains, subfamily A, member 3 (hematopoietic cell-specific)
9601	MAP3K5	-0.1633	0.4275	mitogen-activated protein kinase kinase kinase 5
9602	DNAJB1	-0.1633	0.4149	DnaJ (Hsp40) homolog, subfamily B, member 1
9603	ZNF479	-0.1637	0.4982	zinc finger protein 479
9604	ZNF280C	-0.1637	0.4368	zinc finger protein 280C
9605	RAB2B	-0.1637	0.4134	RAB2B, member RAS oncogene family
9606	LOC255411	-0.1637	0.5325	uncharacterized LOC255411
9607	KLHDC5	-0.1637	0.4352	kelch domain containing 5
9608	GPR111	-0.1637	0.5292	G protein-coupled receptor 111
9609	GBGT1	-0.1637	0.4711	globoside alpha-1,3-N-acetylgalactosaminyltransferase 1
9610	FLJ40852	-0.1637	0.4718	uncharacterized LOC285962
9611	CPA6	-0.1637	0.4466	carboxypeptidase A6
9612	C14orf129	-0.1637	0.4467	chromosome 14 open reading frame 129
9613	PPOX	-0.1642	0.4368	protoporphyrinogen oxidase
9614	PCBP3	-0.1642	0.4771	poly(rC) binding protein 3
9615	LPAR3	-0.1642	0.4759	lysophosphatidic acid receptor 3
9616	FGF12	-0.1642	0.443	fibroblast growth factor 12
9617	CST8	-0.1642	0.5287	cystatin 8 (cystatin-related epididymal specific)
9618	KIR2DL3	-0.1645	0.4379	killer cell immunoglobulin-like receptor, two domains, long cytoplasmic tail, 3
9619	ZNF19	-0.165	0.445	zinc finger protein 19
9620	ZBTB12	-0.165	0.5452	zinc finger and BTB domain containing 12
9621	YWHAB	-0.165	0.4401	tyrosine 3-monooxygenase/tryptophan 5-monooxygenase activation protein, beta polypeptide
9622	TEFM	-0.165	0.456	transcription elongation factor, mitochondrial
9623	TADA2A	-0.165	0.3867	transcriptional adaptor 2A
9624	SOD2	-0.165	0.4462	superoxide dismutase 2, mitochondrial
9625	ROPN1	-0.165	0.4476	rhophilin associated tail protein 1
9626	RHPN1	-0.165	0.5224	rhophilin, Rho GTPase binding protein 1
9627	HNRNPH1	-0.165	0.4593	heterogeneous nuclear ribonucleoprotein H1 (H)
9628	GON4L	-0.165	0.4073	gon-4-like (C. elegans)
9629	FAM182B	-0.165	0.4784	family with sequence similarity 182, member B
9630	DISP2	-0.165	0.4948	dispatched homolog 2 (Drosophila)
9631	CPA1	-0.165	0.5231	carboxypeptidase A1 (pancreatic)
9632	AOC4	-0.165	0.5073	amine oxidase, copper containing 3 (vascular adhesion protein 1) pseudogene
9633	WDR52	-0.1657	0.44	WD repeat domain 52
9634	SEMG1	-0.1658	0.4825	semenogelin I
9635	RRP15	-0.1658	0.4659	ribosomal RNA processing 15 homolog (S. cerevisiae)
9636	RPGRIP1	-0.1658	0.4991	retinitis pigmentosa GTPase regulator interacting protein 1
9637	PRPF3	-0.1658	0.4512	PRP3 pre-mRNA processing factor 3 homolog (S. cerevisiae)
9638	PARN	-0.1658	0.4247	poly(A)-specific ribonuclease
9639	NCK2	-0.1658	0.3938	NCK adaptor protein 2
9640	LY9	-0.1658	0.5141	lymphocyte antigen 9
9641	IL17B	-0.1658	0.5068	interleukin 17B
9642	CROCCP3	-0.1658	0.506	ciliary rootlet coiled-coil, rootletin pseudogene 3
9643	CHST1	-0.1658	0.4711	carbohydrate (keratan sulfate Gal-6) sulfotransferase 1
9644	BPI	-0.1658	0.4943	bactericidal/permeability-increasing protein
9645	ARID4B	-0.1658	0.4604	AT rich interactive domain 4B (RBP1-like)
9646	ADCK2	-0.1658	0.3794	aarF domain containing kinase 2
9647	ZNF75A	-0.1663	0.428	zinc finger protein 75a
9648	ZDHHC12	-0.1663	0.4557	zinc finger, DHHC-type containing 12
9649	RPUSD4	-0.1663	0.4302	RNA pseudouridylate synthase domain containing 4
9650	PIK3AP1	-0.1663	0.4413	phosphoinositide-3-kinase adaptor protein 1
9651	FGF11	-0.1663	0.4939	fibroblast growth factor 11
9652	CD200R1	-0.1663	0.4539	CD200 receptor 1
9653	UBD	-0.1667	0.4321	ubiquitin D
9654	SNAPC5	-0.1667	0.3437	small nuclear RNA activating complex, polypeptide 5, 19kDa
9655	SLCO3A1	-0.1667	0.3636	solute carrier organic anion transporter family, member 3A1
9656	MAGEB4	-0.1667	0.4968	melanoma antigen family B, 4
9657	LPGAT1	-0.1667	0.443	lysophosphatidylglycerol acyltransferase 1
9658	CPB2	-0.1667	0.4756	carboxypeptidase B2 (plasma)

9659	BTN1A1	-0.1667	0.5189	butyrophilin, subfamily 1, member A1
9660	ARHGAP35	-0.1667	0.347	Rho GTPase activating protein 35
9661	ACTL10	-0.1671	0.5356	actin-like 10
9662	PCDHA2	-0.1673	0.4974	protocadherin alpha 2
9663	TGS1	-0.1675	0.4493	trimethylguanosine synthase 1
9664	PRR24	-0.1675	0.4532	proline rich 24
9665	NDUFB2	-0.1675	0.4575	NADH dehydrogenase (ubiquinone) 1 beta subcomplex, 2, 8kDa
9666	NCOA5	-0.1675	0.4171	nuclear receptor coactivator 5
9667	LRIG1	-0.1675	0.3996	leucine-rich repeats and immunoglobulin-like domains 1
9668	JAGN1	-0.1675	0.4248	jagunal homolog 1 (Drosophila)
9669	FAM171B	-0.1675	0.3236	family with sequence similarity 171, member B
9670	FAM107B	-0.1675	0.4169	family with sequence similarity 107, member B
9671	FAAH2	-0.1675	0.3927	fatty acid amide hydrolase 2
9672	COQ6	-0.1675	0.3958	coenzyme Q6 homolog, monooxygenase (S. cerevisiae)
9673	COA5	-0.1675	0.4396	cytochrome C oxidase assembly factor 5
9674	CCDC82	-0.1675	0.4472	coiled-coil domain containing 82
9675	ZNF133	-0.1683	0.4414	zinc finger protein 133
9676	TMEM8A	-0.1683	0.4281	transmembrane protein 8A
9677	SUPT3H	-0.1683	0.3943	suppressor of Ty 3 homolog (S. cerevisiae)
9678	SRPK3	-0.1683	0.5221	SRSF protein kinase 3
9679	SLAMF7	-0.1683	0.4514	SLAM family member 7
9680	RENBP	-0.1683	0.4859	renin binding protein
9681	PPFIA1	-0.1683	0.4281	protein tyrosine phosphatase, receptor type, f polypeptide (PTPRF), interacting protein (liprin), alpha 1
9682	PAFAH1B1	-0.1683	0.4373	platelet-activating factor acetylhydrolase 1b, regulatory subunit 1 (45kDa)
9683	IP6K2	-0.1683	0.4191	inositol hexakisphosphate kinase 2
9684	GPR77	-0.1683	0.496	G protein-coupled receptor 77
9685	DHX9	-0.1683	0.4582	DEAH (Asp-Glu-Ala-His) box polypeptide 9
9686	CPN2	-0.1683	0.5267	carboxypeptidase N, polypeptide 2
9687	ZNF568	-0.1688	0.4024	zinc finger protein 568
9688	IQUB	-0.1688	0.4149	IQ motif and ubiquitin domain containing
9689	FOXN4	-0.1688	0.542	forkhead box N4
9690	DNTTIP1	-0.1688	0.4247	deoxynucleotidyltransferase, terminal, interacting protein 1
9691	C8orf12	-0.1688	0.5379	chromosome 8 open reading frame 12
9692	BPIFA3	-0.1688	0.5511	BPI fold containing family A, member 3
9693	ZNF91	-0.1692	0.4366	zinc finger protein 91
9694	UBE2B	-0.1692	0.4585	ubiquitin-conjugating enzyme E2B
9695	SPINLW1	-0.1692	0.5042	serine peptidase inhibitor-like, with Kunitz and WAP domains 1 (eppin)
9696	SLC45A2	-0.1692	0.5209	solute carrier family 45, member 2
9697	PALB2	-0.1692	0.4442	partner and localizer of BRCA2
9698	MUC4	-0.1692	0.5244	mucin 4, cell surface associated
9699	MCF2L	-0.1692	0.4561	MCF.2 cell line derived transforming sequence-like
9700	MC5R	-0.1692	0.516	melanocortin 5 receptor
9701	GRIK2	-0.1692	0.4611	glutamate receptor, ionotropic, kainate 2
9702	EXTL1	-0.1692	0.5153	exostoses (multiple)-like 1
9703	YKT6	-0.17	0.4139	YKT6 v-SNARE homolog (S. cerevisiae)
9704	WDR81	-0.17	0.432	WD repeat domain 81
9705	TAF7	-0.17	0.4528	TAF7 RNA polymerase II, TATA box binding protein (TBP)-associated factor, 55kDa
9706	PKN3	-0.17	0.4341	protein kinase N3
9707	PDIK1L	-0.17	0.453	PDLIM1 interacting kinase 1 like
9708	NOVA1	-0.17	0.4179	neuro-oncological ventral antigen 1
9709	HOGA1	-0.17	0.4954	4-hydroxy-2-oxoglutarate aldolase 1
9710	GFOD2	-0.17	0.4039	glucose-fructose oxidoreductase domain containing 2
9711	F11	-0.17	0.517	coagulation factor XI
9712	DNAJB5	-0.17	0.4359	DnaJ (Hsp40) homolog, subfamily B, member 5
9713	CALML6	-0.17	0.5326	calmodulin-like 6
9714	ATP6V0E2-AS1	-0.17	0.4538	ATP6V0E2 antisense RNA 1 (non-protein coding)
9715	ARVCF	-0.17	0.4952	armadillo repeat gene deleted in velocardiofacial syndrome
9716	ARHGEF38	-0.17	0.5026	Rho guanine nucleotide exchange factor (GEF) 38
9717	AQP9	-0.17	0.4717	aquaporin 9
9718	APEH	-0.17	0.4325	N-acylaminoacyl-peptide hydrolase
9719	TNFRSF17	-0.1708	0.3955	tumor necrosis factor receptor superfamily, member 17
9720	ST3GAL5	-0.1708	0.3826	ST3 beta-galactoside alpha-2,3-sialyltransferase 5
9721	SLC25A3	-0.1708	0.4526	solute carrier family 25 (mitochondrial carrier; phosphate carrier), member 3
9722	S1PR1	-0.1708	0.4297	sphingosine-1-phosphate receptor 1
9723	RNF111	-0.1708	0.439	ring finger protein 111
9724	NMD3	-0.1708	0.4614	NMD3 homolog (S. cerevisiae)
9725	MSMO1	-0.1708	0.4399	methylsterol monooxygenase 1
9726	ICAM1	-0.1708	0.4229	intercellular adhesion molecule 1
9727	FCRL2	-0.1708	0.5093	Fc receptor-like 2
9728	CACNA2D3	-0.1708	0.3415	calcium channel, voltage-dependent, alpha 2/delta subunit 3
9729	C17orf75	-0.1708	0.4482	chromosome 17 open reading frame 75
9730	ATP6V1H	-0.1708	0.4345	ATPase, H+ transporting, lysosomal 50/57kDa, V1 subunit H
9731	AIRE	-0.1708	0.535	autoimmune regulator
9732	ZDBF2	-0.1713	0.41	zinc finger, DBF-type containing 2
9733	PPP4R1L	-0.1713	0.3246	protein phosphatase 4, regulatory subunit 1-like
9734	GPR101	-0.1713	0.5505	G protein-coupled receptor 101
9735	C11orf35	-0.1713	0.5201	chromosome 11 open reading frame 35
9736	BSND	-0.1713	0.5415	Bartter syndrome, infantile, with sensorineural deafness (Barttin)
9737	RIC8A	-0.1717	0.3996	resistance to inhibitors of cholinesterase 8 homolog A (C. elegans)
9738	RDH11	-0.1717	0.4406	retinol dehydrogenase 11 (all-trans/9-cis/11-cis)
9739	MTERF	-0.1717	0.4495	mitochondrial transcription termination factor
9740	LOC388796	-0.1717	0.4475	uncharacterized LOC388796
9741	HIVEP3	-0.1717	0.4769	human immunodeficiency virus type I enhancer binding protein 3
9742	WRNIP1	-0.1725	0.422	Werner helicase interacting protein 1

9743	TNFRSF25	-0.1725	0.4783	tumor necrosis factor receptor superfamily, member 25
9744	SLC39A5	-0.1725	0.5347	solute carrier family 39 (metal ion transporter), member 5
9745	SERP2	-0.1725	0.4277	stress-associated endoplasmic reticulum protein family member 2
9746	RFC1	-0.1725	0.4441	replication factor C (activator 1) 1, 145kDa
9747	PRPF4	-0.1725	0.4512	PRP4 pre-mRNA processing factor 4 homolog (yeast)
9748	KCNS2	-0.1725	0.5325	potassium voltage-gated channel, delayed-rectifier, subfamily S, member 2
9749	HTR1A	-0.1725	0.5145	5-hydroxytryptamine (serotonin) receptor 1A, G protein-coupled
9750	HDAC6	-0.1725	0.4137	histone deacetylase 6
9751	HCN1	-0.1725	0.5059	hyperpolarization activated cyclic nucleotide-gated potassium channel 1
9752	GLRX3	-0.1725	0.4645	glutaredoxin 3
9753	DHCR7	-0.1725	0.3968	7-dehydrocholesterol reductase
9754	ANXA2R	-0.1725	0.3911	annexin A2 receptor
9755	SLC25A40	-0.1733	0.4244	solute carrier family 25, member 40
9756	RSL24D1	-0.1733	0.471	ribosomal L24 domain containing 1
9757	PLCH1	-0.1733	0.4099	phospholipase C, eta 1
9758	PBRM1	-0.1733	0.4388	polybromo 1
9759	NDN	-0.1733	0.3702	necdin homolog (mouse)
9760	INHBC	-0.1733	0.5256	inhibin, beta C
9761	DSCR3	-0.1733	0.4097	Down syndrome critical region gene 3
9762	CPE	-0.1733	0.4046	carboxypeptidase E
9763	CD177	-0.1733	0.5011	CD177 molecule
9764	AZU1	-0.1733	0.5119	azurocidin 1
9765	SRCRB4D	-0.1737	0.525	scavenger receptor cysteine rich domain containing, group B (4 domains)
9766	PRAC	-0.1737	0.5291	prostate cancer susceptibility candidate
9767	N4BP2	-0.1737	0.4371	NEDD4 binding protein 2
9768	MB21D2	-0.1737	0.3613	Mab-21 domain containing 2
9769	DLL4	-0.1737	0.5005	delta-like 4 (Drosophila)
9770	VSIG4	-0.1742	0.4665	V-set and immunoglobulin domain containing 4
9771	TRAPPC9	-0.1742	0.4402	trafficking protein particle complex 9
9772	SIGLEC7	-0.1742	0.5011	sialic acid binding Ig-like lectin 7
9773	NUDT6	-0.1742	0.4341	nudix (nucleoside diphosphate linked moiety X)-type motif 6
9774	LMO3	-0.1742	0.4297	LIM domain only 3 (rhombotin-like 2)
9775	LILRA1	-0.1742	0.484	leukocyte immunoglobulin-like receptor, subfamily A (with TM domain), member 1
9776	KCNMB2	-0.1742	0.4912	potassium large conductance calcium-activated channel, subfamily M, beta member 2
9777	KCNA2	-0.1742	0.4939	potassium voltage-gated channel, shaker-related subfamily, member 2
9778	IL17RA	-0.1742	0.3876	interleukin 17 receptor A
9779	ICAM3	-0.1742	0.4331	intercellular adhesion molecule 3
9780	FAM102A	-0.1742	0.3571	family with sequence similarity 102, member A
9781	EDDM3B	-0.1742	0.4898	epididymal protein 3B
9782	CR2	-0.1742	0.4352	complement component (3d/Epstein Barr virus) receptor 2
9783	ADAMTS8	-0.1742	0.5102	ADAM metallopeptidase with thrombospondin type 1 motif, 8
9784	TTYH3	-0.175	0.3996	tweety homolog 3 (Drosophila)
9785	SLC25A5	-0.175	0.465	solute carrier family 25 (mitochondrial carrier; adenine nucleotide translocator), member 5
9786	RPL11	-0.175	0.4602	ribosomal protein L11
9787	MYOM2	-0.175	0.3834	myomesin (M-protein) 2, 165kDa
9788	MYO7B	-0.175	0.5178	myosin VIIB
9789	LOC100190938	-0.175	0.491	uncharacterized LOC100190938
9790	LAX1	-0.175	0.4434	lymphocyte transmembrane adaptor 1
9791	H3F3B	-0.175	0.4291	H3 histone, family 3B (H3.3B)
9792	CD209	-0.175	0.5088	CD209 molecule
9793	LOC145845	-0.1757	0.5216	uncharacterized LOC145845
9794	ZRSR2	-0.1758	0.3591	zinc finger (CCCH type), RNA-binding motif and serine/arginine rich 2
9795	ZNF365	-0.1758	0.4294	zinc finger protein 365
9796	USP21	-0.1758	0.409	ubiquitin specific peptidase 21
9797	UBE3C	-0.1758	0.443	ubiquitin protein ligase E3C
9798	PTGDS	-0.1758	0.4515	prostaglandin D2 synthase 21kDa (brain)
9799	GZMK	-0.1758	0.4634	granzyme K (granzyme 3; tryptase II)
9800	GLT25D1	-0.1758	0.3969	glycosyltransferase 25 domain containing 1
9801	FCN3	-0.1758	0.5078	ficolin (collagen/fibrinogen domain containing) 3 (Hakata antigen)
9802	CPA2	-0.1758	0.5047	carboxypeptidase A2 (pancreatic)
9803	CD5	-0.1758	0.5	CD5 molecule
9804	AMHR2	-0.1758	0.5196	anti-Mullerian hormone receptor, type II
9805	ACTL8	-0.1758	0.5041	actin-like 8
9806	SPATA9	-0.1762	0.3771	spermatogenesis associated 9
9807	MPV17L	-0.1762	0.501	MPV17 mitochondrial membrane protein-like
9808	HACL1	-0.1762	0.4244	2-hydroxyacyl-CoA lyase 1
9809	CPSF2	-0.1762	0.4403	cleavage and polyadenylation specific factor 2, 100kDa
9810	CCDC142	-0.1762	0.397	coiled-coil domain containing 142
9811	CCDC125	-0.1762	0.3958	coiled-coil domain containing 125
9812	ZNF45	-0.1767	0.4354	zinc finger protein 45
9813	TAF6L	-0.1767	0.4157	TAF6-like RNA polymerase II, p300/CBP-associated factor (PCAF)-associated factor, 65kDa
9814	SRSF1	-0.1767	0.4635	serine/arginine-rich splicing factor 1
9815	DNAH6	-0.1767	0.5042	dynein, axonemal, heavy chain 6
9816	ZNF187	-0.1775	0.4102	zinc finger protein 187
9817	USH2A	-0.1775	0.5128	Usher syndrome 2A (autosomal recessive, mild)
9818	SULT1C4	-0.1775	0.3549	sulfotransferase family, cytosolic, 1C, member 4
9819	SNTA1	-0.1775	0.4681	syntrophin, alpha 1
9820	NRXN3	-0.1775	0.43	neurexin 3
9821	GDEP	-0.1775	0.5201	gene differentially expressed in prostate
9822	FAM134A	-0.1775	0.4087	family with sequence similarity 134, member A
9823	EMX1	-0.1775	0.519	empty spiracles homeobox 1
9824	DTX2	-0.1775	0.4418	deltex homolog 2 (Drosophila)
9825	BRD7P3	-0.1775	0.4656	bromodomain containing 7 pseudogene 3
9826	APOC3	-0.1775	0.522	apolipoprotein C-III

9827	CNKSR2	-0.1778	0.4075	connector enhancer of kinase suppressor of Ras 2
9828	ZNF358	-0.1783	0.4551	zinc finger protein 358
9829	YY1	-0.1783	0.455	YY1 transcription factor
9830	PTS	-0.1783	0.4511	6-pyruvoyltetrahydropterin synthase
9831	PRDM8	-0.1783	0.4905	PR domain containing 8
9832	PPP2R1B	-0.1783	0.418	protein phosphatase 2, regulatory subunit A, beta
9833	HEATR1	-0.1783	0.4571	HEAT repeat containing 1
9834	FMN1	-0.1783	0.9999	formin 1
9835	DYRK3	-0.1783	0.3773	dual-specificity tyrosine-(Y)-phosphorylation regulated kinase 3
9836	CCDC19	-0.1783	0.4778	coiled-coil domain containing 19
9837	APOA4	-0.1783	0.5215	apolipoprotein A-IV
9838	PSMG4	-0.1786	0.4556	proteasome (prosome, macropain) assembly chaperone 4
9839	LOC145837	-0.1786	0.4663	uncharacterized LOC145837
9840	RTP1	-0.1787	0.5368	receptor (chemosensory) transporter protein 1
9841	MSANTD4	-0.1787	0.4365	Myb/SANT-like DNA-binding domain containing 4 with coiled-coils
9842	DCAF5	-0.1787	0.3974	DDB1 and CUL4 associated factor 5
9843	CD300LG	-0.1787	0.5401	CD300 molecule-like family member g
9844	CCDC27	-0.1787	0.5504	coiled-coil domain containing 27
9845	CCDC24	-0.1787	0.4724	coiled-coil domain containing 24
9846	B3GNT6	-0.1787	0.5406	UDP-GlcNAc:betaGal beta-1,3-N-acetylglucosaminyltransferase 6 (core 3 synthase)
9847	ABCC11	-0.1787	0.5322	ATP-binding cassette, sub-family C (CFTR/MRP), member 11
9848	WDR37	-0.1792	0.4145	WD repeat domain 37
9849	SLCO1C1	-0.1792	0.4787	solute carrier organic anion transporter family, member 1C1
9850	KCNE1	-0.1792	0.4999	potassium voltage-gated channel, Isk-related family, member 1
9851	FEZ1	-0.1792	0.3837	fasciculation and elongation protein zeta 1 (zygin I)
9852	ETNK1	-0.1792	0.4515	ethanolamine kinase 1
9853	ABCA2	-0.1792	0.4352	ATP-binding cassette, sub-family A (ABC1), member 2
9854	RND2	-0.18	0.4774	Rho family GTPase 2
9855	RGR	-0.18	0.5289	retinal G protein coupled receptor
9856	PTCH1	-0.18	0.3458	patched 1
9857	NOBOX	-0.18	0.5538	NOBOX oogenesis homeobox
9858	MYADML2	-0.18	0.5528	myeloid-associated differentiation marker-like 2
9859	MAPK3	-0.18	0.4182	mitogen-activated protein kinase 3
9860	IGFALS	-0.18	0.5165	insulin-like growth factor binding protein, acid labile subunit
9861	GLI4	-0.18	0.4879	GLI family zinc finger 4
9862	FAM186A	-0.18	0.535	family with sequence similarity 186, member A
9863	EFHB	-0.18	0.3892	EF-hand domain family, member B
9864	CXCR5	-0.18	0.4975	chemokine (C-X-C motif) receptor 5
9865	CIRBP	-0.18	0.4085	cold inducible RNA binding protein
9866	C3orf27	-0.18	0.5322	chromosome 3 open reading frame 27
9867	SYT2	-0.1808	0.5151	synaptotagmin II
9868	SOGA2	-0.1808	0.4045	SOGA family member 2
9869	RAD1	-0.1808	0.4507	RAD1 homolog (S. pombe)
9870	NAA50	-0.1808	0.4625	N(alpha)-acetyltransferase 50, NatE catalytic subunit
9871	KPNA1	-0.1808	0.44	karyopherin alpha 1 (importin alpha 5)
9872	GPR50	-0.1808	0.509	G protein-coupled receptor 50
9873	FAM50B	-0.1808	0.3316	family with sequence similarity 50, member B
9874	ENO1	-0.1808	0.4385	enolase 1, (alpha)
9875	ZNF511	-0.1813	0.4196	zinc finger protein 511
9876	TTTY14	-0.1813	0.5251	testis-specific transcript, Y-linked 14 (non-protein coding)
9877	MRPS10	-0.1813	0.43	mitochondrial ribosomal protein S10
9878	MAPK15	-0.1813	0.5437	mitogen-activated protein kinase 15
9879	FAM116B	-0.1813	0.5046	family with sequence similarity 116, member B
9880	C1orf65	-0.1813	0.5298	chromosome 1 open reading frame 65
9881	C12orf32	-0.1813	0.4413	chromosome 12 open reading frame 32
9882	WNT7B	-0.1817	0.4943	wingless-type MMTV integration site family, member 7B
9883	RIF1	-0.1817	0.4587	RAP1 interacting factor homolog (yeast)
9884	ITPKB	-0.1817	0.3787	inositol-trisphosphate 3-kinase B
9885	INHA	-0.1817	0.5034	inhibin, alpha
9886	CLCN2	-0.1817	0.4475	chloride channel, voltage-sensitive 2
9887	CD84	-0.1817	0.4518	CD84 molecule
9888	C9orf114	-0.1817	0.4209	chromosome 9 open reading frame 114
9889	ACTR3C	-0.1817	0.9996	ARP3 actin-related protein 3 homolog C (yeast)
9890	WDR88	-0.1825	0.5262	WD repeat domain 88
9891	VPREB3	-0.1825	0.4965	pre-B lymphocyte 3
9892	UCP2	-0.1825	0.3689	uncoupling protein 2 (mitochondrial, proton carrier)
9893	SMC1A	-0.1825	0.4327	structural maintenance of chromosomes 1A
9894	RBP5	-0.1825	0.4967	retinol binding protein 5, cellular
9895	OR1Q1	-0.1825	0.5246	olfactory receptor, family 1, subfamily Q, member 1
9896	NENF	-0.1825	0.379	neudesin neurotrophic factor
9897	HERC1	-0.1825	0.4238	HECT and RLD domain containing E3 ubiquitin protein ligase family member 1
9898	GOT1	-0.1825	0.4207	glutamic-oxaloacetic transaminase 1, soluble (aspartate aminotransferase 1)
9899	DENND1A	-0.1825	0.3314	DENN/MADD domain containing 1A
9900	ATXN1L	-0.1825	0.4282	ataxin 1-like
9901	IGSF9B	-0.1827	0.4932	immunoglobulin superfamily, member 9B
9902	RPL18	-0.1829	0.4281	ribosomal protein L18
9903	LOC100289187	-0.1829	0.9999	transmembrane protein 225-like
9904	FLJ45513	-0.1829	0.9996	uncharacterized LOC729220
9905	WAPAL	-0.1833	0.4546	wings apart-like homolog (Drosophila)
9906	TTC9	-0.1833	0.3518	tetratricopeptide repeat domain 9
9907	TPSD1	-0.1833	0.5146	tryptase delta 1
9908	TARS2	-0.1833	0.3966	threonyl-tRNA synthetase 2, mitochondrial (putative)
9909	SLCO1A2	-0.1833	0.457	solute carrier organic anion transporter family, member 1A2
9910	SF3B4	-0.1833	0.4213	splicing factor 3b, subunit 4, 49kDa

9911	RLIM	-0.1833	0.4474	ring finger protein, LIM domain interacting
9912	PADI4	-0.1833	0.5121	peptidyl arginine deiminase, type IV
9913	NDUFA1	-0.1833	0.4539	NADH dehydrogenase (ubiquinone) 1 alpha subcomplex, 1, 7.5kDa
9914	MNS1	-0.1833	0.4324	meiosis-specific nuclear structural 1
9915	LOC96610	-0.1833	0.2698	BMS1 homolog, ribosome assembly protein (yeast) pseudogene
9916	KCNAB3	-0.1833	0.4795	potassium voltage-gated channel, shaker-related subfamily, beta member 3
9917	C11orf49	-0.1833	0.3725	chromosome 11 open reading frame 49
9918	ARHGAP44	-0.1833	0.3206	Rho GTPase activating protein 44
9919	SMEK3P	-0.1838	0.5057	SMEK homolog 3, suppressor of mek1 (Dictyostelium) pseudogene
9920	SLC36A4	-0.1838	0.4145	solute carrier family 36 (proton/amino acid symporter), member 4
9921	SLC35F4	-0.1838	0.5023	solute carrier family 35, member F4
9922	MAMDC4	-0.1838	0.5018	MAM domain containing 4
9923	FAM218A	-0.1838	0.4568	family with sequence similarity 218, member A
9924	NDRG4	-0.1842	0.4265	NDRG family member 4
9925	NAGPA	-0.1842	0.4032	N-acetylglucosamine-1-phosphodiester alpha-N-acetylglucosaminidase
9926	IL13RA2	-0.1842	0.354	interleukin 13 receptor, alpha 2
9927	HS3ST2	-0.1842	0.4785	heparan sulfate (glucosamine) 3-O-sulfotransferase 2
9928	GLTPD1	-0.1842	0.4433	glycolipid transfer protein domain containing 1
9929	EIF3L	-0.1842	0.4294	eukaryotic translation initiation factor 3, subunit L
9930	FAM190A	-0.1843	0.3889	family with sequence similarity 190, member A
9931	WNT3	-0.185	0.4997	wingless-type MMTV integration site family, member 3
9932	UBXN8	-0.185	0.4491	UBX domain protein 8
9933	TMEM178A	-0.185	0.4206	transmembrane protein 178A
9934	THOC2	-0.185	0.4371	THO complex 2
9935	SPIRE1	-0.185	0.3859	spire homolog 1 (Drosophila)
9936	SPATA17	-0.185	0.3403	spermatogenesis associated 17
9937	OR1F2P	-0.185	0.5079	olfactory receptor, family 1, subfamily F, member 2
9938	NEURL1B	-0.185	0.3478	neuralized homolog 1B (Drosophila)
9939	MAP3K11	-0.185	0.4286	mitogen-activated protein kinase kinase kinase 11
9940	LOC645139	-0.185	0.9996	poly(A) binding protein interacting protein 1 pseudogene
9941	INHBE	-0.185	0.4068	inhibin, beta E
9942	EP300	-0.185	0.4234	E1A binding protein p300
9943	CBLN2	-0.185	0.4611	cerebellin 2 precursor
9944	KIAA0408	-0.1857	0.3531	KIAA0408
9945	SP2	-0.1858	0.4178	Sp2 transcription factor
9946	SIAH1	-0.1858	0.4474	siah E3 ubiquitin protein ligase 1
9947	RGS10	-0.1858	0.4329	regulator of G-protein signaling 10
9948	PITRM1	-0.1858	0.4133	pitrilysin metallopeptidase 1
9949	OTUB2	-0.1858	0.3925	OTU domain, ubiquitin aldehyde binding 2
9950	NR5A1	-0.1858	0.5223	nuclear receptor subfamily 5, group A, member 1
9951	GSPT2	-0.1858	0.4312	G1 to S phase transition 2
9952	ARL6IP4	-0.1858	0.4389	ADP-ribosylation-like factor 6 interacting protein 4
9953	ABTB2	-0.1858	0.4313	ankyrin repeat and BTB (POZ) domain containing 2
9954	ZFYVE20	-0.1863	0.3567	zinc finger, FYVE domain containing 20
9955	FBXL21	-0.1863	0.4633	F-box and leucine-rich repeat protein 21 (gene/pseudogene)
9956	EARS2	-0.1863	0.4246	glutamyl-tRNA synthetase 2, mitochondrial (putative)
9957	C6orf118	-0.1863	0.4823	chromosome 6 open reading frame 118
9958	YARS2	-0.1867	0.4433	tyrosyl-tRNA synthetase 2, mitochondrial
9959	UNC13A	-0.1867	0.4864	unc-13 homolog A (C. elegans)
9960	TEX15	-0.1867	0.4251	testis expressed 15
9961	PSPH	-0.1867	0.4405	phosphoserine phosphatase
9962	PRDM2	-0.1867	0.3856	PR domain containing 2, with ZNF domain
9963	LRRN2	-0.1867	0.5054	leucine rich repeat neuronal 2
9964	LINC00339	-0.1867	0.3886	long intergenic non-protein coding RNA 339
9965	DSCR4	-0.1867	0.5044	Down syndrome critical region gene 4
9966	ALKBH4	-0.1867	0.4306	alkB, alkylation repair homolog 4 (E. coli)
9967	CAPZA1	-0.1871	0.449	capping protein (actin filament) muscle Z-line, alpha 1
9968	TKTL2	-0.1875	0.5213	transketolase-like 2
9969	RBM48	-0.1875	0.4388	RNA binding motif protein 48
9970	DBN1	-0.1875	0.3647	drebrin 1
9971	C16orf62	-0.1875	0.3793	chromosome 16 open reading frame 62
9972	AQP4	-0.1875	0.448	aquaporin 4
9973	AGK	-0.1875	0.4322	acylglycerol kinase
9974	ZNF672	-0.1883	0.3702	zinc finger protein 672
9975	PCBP1	-0.1883	0.4219	poly(rC) binding protein 1
9976	KCNC4	-0.1883	0.472	potassium voltage-gated channel, Shaw-related subfamily, member 4
9977	IQCC	-0.1883	0.4589	IQ motif containing C
9978	FAIM	-0.1883	0.4501	Fas apoptotic inhibitory molecule
9979	EIF4G1	-0.1883	0.4206	eukaryotic translation initiation factor 4 gamma, 1
9980	VGLL2	-0.1887	0.5376	vestigial like 2 (Drosophila)
9981	RSPO2	-0.1887	0.4123	R-spondin 2
9982	C19orf70	-0.1887	0.4461	chromosome 19 open reading frame 70
9983	ANKRD44	-0.1887	0.3979	ankyrin repeat domain 44
9984	ABLIM2	-0.1887	0.4943	actin binding LIM protein family, member 2
9985	ZNF749	-0.1891	0.3222	zinc finger protein 749
9986	TSC22D2	-0.1892	0.4344	TSC22 domain family, member 2
9987	SSX3	-0.1892	0.4973	synovial sarcoma, X breakpoint 3
9988	SRP72	-0.1892	0.4585	signal recognition particle 72kDa
9989	SHMT2	-0.1892	0.4261	serine hydroxymethyltransferase 2 (mitochondrial)
9990	SATB2	-0.1892	0.3887	SATB homeobox 2
9991	RPS16	-0.1892	0.4434	ribosomal protein S16
9992	NDUFAF1	-0.1892	0.4263	NADH dehydrogenase (ubiquinone) complex I, assembly factor 1
9993	MC4R	-0.1892	0.4597	melanocortin 4 receptor
9994	MAGEB3	-0.1892	0.4884	melanoma antigen family B, 3

9995	HIST1H2AM	-0.1892	0.3703	histone cluster 1, H2am
9996	DUSP7	-0.1892	0.3098	dual specificity phosphatase 7
9997	CCNC	-0.1892	0.4616	cyclin C
9998	ZFAT	-0.19	0.3478	zinc finger and AT hook domain containing
9999	USP4	-0.19	0.3866	ubiquitin specific peptidase 4 (proto-oncogene)
10000	UPP2	-0.19	0.5208	uridine phosphorylase 2
10001	TIGD5	-0.19	0.4657	tigger transposable element derived 5
10002	SELO	-0.19	0.44	selenoprotein O
10003	RIBC1	-0.19	0.5009	RIB43A domain with coiled-coils 1
10004	R3HCC1L	-0.19	0.3773	R3H domain and coiled-coil containing 1-like
10005	OGDHL	-0.19	0.4307	oxoglutarate dehydrogenase-like
10006	NFXL1	-0.19	0.4336	nuclear transcription factor, X-box binding-like 1
10007	NDUFS2	-0.19	0.4218	NADH dehydrogenase (ubiquinone) Fe-S protein 2, 49kDa (NADH-coenzyme Q reductase)
10008	MUC6	-0.19	0.5228	mucin 6, oligomeric mucus/gel-forming
10009	MEPCE	-0.19	0.408	methylphosphate capping enzyme
10010	IL6R	-0.19	0.3749	interleukin 6 receptor
10011	GTPBP5	-0.19	0.3967	GTP binding protein 5 (putative)
10012	GSC2	-0.19	0.5056	goosecoid homeobox 2
10013	FOLR2	-0.19	0.4816	folate receptor 2 (fetal)
10014	FAM5C	-0.19	0.3499	family with sequence similarity 5, member C
10015	CXorf56	-0.19	0.387	chromosome X open reading frame 56
10016	CRY2	-0.19	0.4063	cryptochrome 2 (photolyase-like)
10017	ADAP2	-0.19	0.4228	ArfGAP with dual PH domains 2
10018	ADAM21	-0.19	0.4461	ADAM metallopeptidase domain 21
10019	TMEM53	-0.1908	0.3817	transmembrane protein 53
10020	STXBP1	-0.1908	0.369	syntaxin binding protein 1
10021	LAIR2	-0.1908	0.4496	leukocyte-associated immunoglobulin-like receptor 2
10022	KCNA3	-0.1908	0.4092	potassium voltage-gated channel, shaker-related subfamily, member 3
10023	HTR7	-0.1908	0.4668	5-hydroxytryptamine (serotonin) receptor 7, adenylate cyclase-coupled
10024	GUK1	-0.1908	0.4229	guanylate kinase 1
10025	GP1BA	-0.1908	0.4993	glycoprotein Ib (platelet), alpha polypeptide
10026	CRISP2	-0.1908	0.4782	cysteine-rich secretory protein 2
10027	CCR7	-0.1908	0.4513	chemokine (C-C motif) receptor 7
10028	PLK5	-0.1912	0.5424	polo-like kinase 5
10029	GALNT9	-0.1912	0.5266	UDP-N-acetyl-alpha-D-galactosamine:polypeptide N-acetylgalactosaminyltransferase 9 (GalNAc-T9)
10030	RNF130	-0.1917	0.398	ring finger protein 130
10031	OSBPL9	-0.1917	0.4328	oxysterol binding protein-like 9
10032	LOC654780	-0.1917	0.9996	SFPQ
10033	ICOS	-0.1917	0.4218	inducible T-cell co-stimulator
10034	GABARAP	-0.1917	0.4031	GABA(A) receptor-associated protein
10035	CCDC28A	-0.1917	0.4333	coiled-coil domain containing 28A
10036	C17orf72	-0.1917	0.9996	chromosome 17 open reading frame 72
10037	ZFP82	-0.1925	0.4048	zinc finger protein 82 homolog (mouse)
10038	SPTA1	-0.1925	0.4607	spectrin, alpha, erythrocytic 1 (elliptocytosis 2)
10039	SMARCD2	-0.1925	0.3984	SWI/SNF related, matrix associated, actin dependent regulator of chromatin, subfamily d, member 2
10040	SCN8A	-0.1925	0.4417	sodium channel, voltage gated, type VIII, alpha subunit
10041	SCN1A	-0.1925	0.4603	sodium channel, voltage-gated, type I, alpha subunit
10042	RGS4	-0.1925	0.4199	regulator of G-protein signaling 4
10043	PDE6G	-0.1925	0.5036	phosphodiesterase 6G, cGMP-specific, rod, gamma
10044	MRPS18C	-0.1925	0.4233	mitochondrial ribosomal protein S18C
10045	LOC154822	-0.1925	0.4747	uncharacterized LOC154822
10046	ITIH4	-0.1925	0.4791	inter-alpha-trypsin inhibitor heavy chain family, member 4
10047	INO80E	-0.1925	0.4302	INO80 complex subunit E
10048	FRA10AC1	-0.1925	0.4312	fragile site, folic acid type, rare, fra(10)(q23.3) or fra(10)(q24.2) candidate 1
10049	FLJ10661	-0.1925	0.4746	family with sequence similarity 86, member A pseudogene
10050	CKB	-0.1925	0.3309	creatine kinase, brain
10051	CD1B	-0.1925	0.4899	CD1b molecule
10052	C9orf135	-0.1925	0.4209	chromosome 9 open reading frame 135
10053	C17orf65	-0.1925	0.3704	chromosome 17 open reading frame 65
10054	LINC00493	-0.1929	0.9999	long intergenic non-protein coding RNA 493
10055	STOML2	-0.1933	0.4504	stomatin (EPB72)-like 2
10056	MEGF8	-0.1933	0.4082	multiple EGF-like-domains 8
10057	KCNIP1	-0.1933	0.4678	Kv channel interacting protein 1
10058	ITIH1	-0.1933	0.5086	inter-alpha-trypsin inhibitor heavy chain 1
10059	FZD3	-0.1933	0.4004	frizzled family receptor 3
10060	CAB39	-0.1933	0.4369	calcium binding protein 39
10061	CA5A	-0.1933	0.4709	carbonic anhydrase VA, mitochondrial
10062	C1orf35	-0.1933	0.4436	chromosome 1 open reading frame 35
10063	AIM2	-0.1933	0.4015	absent in melanoma 2
10064	CCDC33	-0.1936	0.5271	coiled-coil domain containing 33
10065	LOC283143	-0.1937	0.388	uncharacterized LOC283143
10066	GABPB1-AS1	-0.1937	0.4129	GABPB1 antisense RNA 1 (non-protein coding)
10067	C15orf56	-0.1937	0.5187	chromosome 15 open reading frame 56
10068	BLZF1	-0.1937	0.4088	basic leucine zipper nuclear factor 1
10069	SYCP2	-0.1942	0.3753	synaptonemal complex protein 2
10070	SH3BP1	-0.1942	0.4794	SH3-domain binding protein 1
10071	PNLIPRP1	-0.1942	0.513	pancreatic lipase-related protein 1
10072	HMGCS1	-0.1942	0.4181	3-hydroxy-3-methylglutaryl-CoA synthase 1 (soluble)
10073	BRE	-0.1942	0.3397	brain and reproductive organ-expressed (TNFRSF1A modulator)
10074	ASCL2	-0.1942	0.4474	achaete-scute complex homolog 2 (Drosophila)
10075	ADAM5P	-0.1942	0.5079	ADAM metallopeptidase domain 5, pseudogene
10076	WNT8A	-0.195	0.5016	wingless-type MMTV integration site family, member 8A
10077	VPS41	-0.195	0.4222	vacuolar protein sorting 41 homolog (S. cerevisiae)
10078	RBM27	-0.195	0.4115	RNA binding motif protein 27

10079	RAB8A	-0.195	0.4147	RAB8A, member RAS oncogene family
10080	PPP2R2C	-0.195	0.4645	protein phosphatase 2, regulatory subunit B, gamma
10081	PPP1R26	-0.195	0.344	protein phosphatase 1, regulatory subunit 26
10082	PITX3	-0.195	0.5155	paired-like homeodomain 3
10083	PAPOLB	-0.195	0.4782	poly(A) polymerase beta (testis specific)
10084	NXPH3	-0.195	0.5009	neurexophilin 3
10085	KIF19	-0.195	0.5085	kinesin family member 19
10086	GRK1	-0.195	0.5099	G protein-coupled receptor kinase 1
10087	GCNT2	-0.195	0.3019	glucosaminyl (N-acetyl) transferase 2, I-branching enzyme (I blood group)
10088	FEM1B	-0.195	0.3758	fem-1 homolog b (C. elegans)
10089	FAM98B	-0.195	0.4286	family with sequence similarity 98, member B
10090	FAM184B	-0.195	0.493	family with sequence similarity 184, member B
10091	DEM1	-0.195	0.3795	defects in morphology 1 homolog (S. cerevisiae)
10092	DCAF11	-0.195	0.3447	DDB1 and CUL4 associated factor 11
10093	CHDH	-0.195	0.4567	choline dehydrogenase
10094	CCBL2	-0.195	0.4343	cysteine conjugate-beta lyase 2
10095	C6orf70	-0.195	0.4006	chromosome 6 open reading frame 70
10096	ANP32C	-0.195	0.4664	acidic (leucine-rich) nuclear phosphoprotein 32 family, member C
10097	ALS2	-0.195	0.4174	amyotrophic lateral sclerosis 2 (juvenile)
10098	ADRA2B	-0.195	0.5079	adrenoceptor alpha 2B
10099	WT1	-0.1958	0.4594	Wilms tumor 1
10100	SIRPA	-0.1958	0.38	signal-regulatory protein alpha
10101	SETD3	-0.1958	0.371	SET domain containing 3
10102	RAX	-0.1958	0.5156	retina and anterior neural fold homeobox
10103	PANK3	-0.1958	0.4007	pantothenate kinase 3
10104	PAK1	-0.1958	0.343	p21 protein (Cdc42/Rac)-activated kinase 1
10105	NONO	-0.1958	0.4314	non-POU domain containing, octamer-binding
10106	MYH13	-0.1958	0.511	myosin, heavy chain 13, skeletal muscle
10107	LMX1B	-0.1958	0.5015	LIM homeobox transcription factor 1, beta
10108	KCNQ4	-0.1958	0.5003	potassium voltage-gated channel, KQT-like subfamily, member 4
10109	ITIH3	-0.1958	0.4894	inter-alpha-trypsin inhibitor heavy chain 3
10110	IFI30	-0.1958	0.4016	interferon, gamma-inducible protein 30
10111	CACNB4	-0.1958	0.4617	calcium channel, voltage-dependent, beta 4 subunit
10112	XKR4	-0.1963	0.447	XK, Kell blood group complex subunit-related family, member 4
10113	TMEM71	-0.1963	0.3916	transmembrane protein 71
10114	PPP1R35	-0.1963	0.4101	protein phosphatase 1, regulatory subunit 35
10115	KIAA1841	-0.1963	0.4191	KIAA1841
10116	ANO9	-0.1963	0.473	anoctamin 9
10117	RPS6KB2	-0.1967	0.411	ribosomal protein S6 kinase, 70kDa, polypeptide 2
10118	RFX1	-0.1967	0.4435	regulatory factor X, 1 (influences HLA class II expression)
10119	KCNH4	-0.1967	0.5227	potassium voltage-gated channel, subfamily H (eag-related), member 4
10120	GJD2	-0.1967	0.5123	gap junction protein, delta 2, 36kDa
10121	CAPN10	-0.1967	0.4904	calpain 10
10122	ZNF107	-0.1975	0.443	zinc finger protein 107
10123	TUBB1	-0.1975	0.4335	tubulin, beta 1 class VI
10124	SZRD1	-0.1975	0.3576	SUZ RNA binding domain containing 1
10125	POGK	-0.1975	0.4283	pogo transposable element with KRAB domain
10126	OXSM	-0.1975	0.4306	3-oxoacyl-ACP synthase, mitochondrial
10127	ONECUT2	-0.1975	0.4699	one cut homeobox 2
10128	LIME1	-0.1975	0.4938	Lck interacting transmembrane adaptor 1
10129	LILRA4	-0.1975	0.4898	leukocyte immunoglobulin-like receptor, subfamily A (with TM domain), member 4
10130	FAM81B	-0.1975	0.4201	family with sequence similarity 81, member B
10131	BMP3	-0.1975	0.4685	bone morphogenetic protein 3
10132	ATP6V0C	-0.1975	0.4058	ATPase, H+ transporting, lysosomal 16kDa, V0 subunit c
10133	ARF3	-0.1975	0.3789	ADP-ribosylation factor 3
10134	SCML2	-0.1983	0.3638	sex comb on midleg-like 2 (Drosophila)
10135	PDZD2	-0.1983	0.3457	PDZ domain containing 2
10136	NAA10	-0.1983	0.4218	N(alpha)-acetyltransferase 10, NatA catalytic subunit
10137	MRPL17	-0.1983	0.4414	mitochondrial ribosomal protein L17
10138	IL25	-0.1983	0.5146	interleukin 25
10139	HMG20A	-0.1983	0.4324	high mobility group 20A
10140	CSTF2T	-0.1983	0.433	cleavage stimulation factor, 3' pre-RNA, subunit 2, 64kDa, tau variant
10141	CBX7	-0.1983	0.3895	chromobox homolog 7
10142	ASTN2	-0.1983	0.3873	astrotactin 2
10143	ZNF765	-0.1988	0.4275	zinc finger protein 765
10144	ZNF330	-0.1992	0.437	zinc finger protein 330
10145	TTC27	-0.1992	0.4449	tetratricopeptide repeat domain 27
10146	TPPP	-0.1992	0.4323	tubulin polymerization promoting protein
10147	SDCCAG3	-0.1992	0.4101	serologically defined colon cancer antigen 3
10148	RPP14	-0.1992	0.4172	ribonuclease P/MRP 14kDa subunit
10149	QPRT	-0.1992	0.341	quinolinate phosphoribosyltransferase
10150	POLDIP3	-0.1992	0.3829	polymerase (DNA-directed), delta interacting protein 3
10151	HIGD2A	-0.1992	0.3997	HIG1 hypoxia inducible domain family, member 2A
10152	FOXN3	-0.1992	0.3505	forkhead box N3
10153	FAM5B	-0.1992	0.4817	family with sequence similarity 5, member B
10154	CDKN1C	-0.1992	0.4014	cyclin-dependent kinase inhibitor 1C (p57, Kip2)
10155	SNX8	-0.2	0.4051	sorting nexin 8
10156	PLEKHA8P1	-0.2	0.3317	pleckstrin homology domain containing, family A member 8 pseudogene 1
10157	MECP2	-0.2	0.3566	methyl CpG binding protein 2 (Rett syndrome)
10158	FAM45B	-0.2	0.3844	family with sequence similarity 45, member A pseudogene
10159	DAO	-0.2	0.5006	D-amino-acid oxidase
10160	C4orf22	-0.2	0.4128	chromosome 4 open reading frame 22
10161	C19orf6	-0.2	0.3999	chromosome 19 open reading frame 6
10162	C10orf67	-0.2	0.4891	chromosome 10 open reading frame 67

10163	ANKRD32	-0.2	0.4355	ankyrin repeat domain 32
10164	USP15	-0.2008	0.4392	ubiquitin specific peptidase 15
10165	TMEM5	-0.2008	0.4283	transmembrane protein 5
10166	SERPINA10	-0.2008	0.499	serpin peptidase inhibitor, clade A (alpha-1 antiproteinase, antitrypsin), member 10
10167	SEC13	-0.2008	0.428	SEC13 homolog (S. cerevisiae)
10168	PAX3	-0.2008	0.4951	paired box 3
10169	MOK	-0.2008	0.258	MOK protein kinase
10170	MMP15	-0.2008	0.4538	matrix metallopeptidase 15 (membrane-inserted)
10171	MAGEA10	-0.2008	0.506	melanoma antigen family A, 10
10172	HNRNPL	-0.2008	0.332	heterogeneous nuclear ribonucleoprotein L
10173	GYPA	-0.2008	0.479	glycophorin A (MNS blood group)
10174	C10orf12	-0.2008	0.2988	chromosome 10 open reading frame 12
10175	BFAR	-0.2008	0.4156	bifunctional apoptosis regulator
10176	WFDC13	-0.2013	0.4987	WAP four-disulfide core domain 13
10177	UBE2F	-0.2013	0.41	ubiquitin-conjugating enzyme E2F (putative)
10178	TAS2R5	-0.2013	0.4113	taste receptor, type 2, member 5
10179	LINC00477	-0.2013	0.5137	long intergenic non-protein coding RNA 477
10180	TUBG2	-0.2017	0.3094	tubulin, gamma 2
10181	TSPAN32	-0.2017	0.4949	tetraspanin 32
10182	SIGLEC9	-0.2017	0.4756	sialic acid binding Ig-like lectin 9
10183	RBM12B-AS1	-0.2017	0.475	RBM12B antisense RNA 1 (non-protein coding)
10184	RBBP5	-0.2017	0.4166	retinoblastoma binding protein 5
10185	COX8A	-0.2017	0.4326	cytochrome c oxidase subunit VIIIA (ubiquitous)
10186	CCDC53	-0.2017	0.4323	coiled-coil domain containing 53
10187	UBASH3B	-0.2025	0.3702	ubiquitin associated and SH3 domain containing B
10188	TP53INP2	-0.2025	0.3783	tumor protein p53 inducible nuclear protein 2
10189	TNFSF14	-0.2025	0.4821	tumor necrosis factor (ligand) superfamily, member 14
10190	SORBS1	-0.2025	0.3734	sorbin and SH3 domain containing 1
10191	RNF112	-0.2025	0.4777	ring finger protein 112
10192	PHF3	-0.2025	0.4465	PHD finger protein 3
10193	KIAA0355	-0.2025	0.4078	KIAA0355
10194	HRSP12	-0.2025	0.4358	heat-responsive protein 12
10195	GRIN2C	-0.2025	0.5034	glutamate receptor, ionotropic, N-methyl D-aspartate 2C
10196	DUSP15	-0.2025	0.5123	dual specificity phosphatase 15
10197	CBL	-0.2025	0.3295	Cbl proto-oncogene, E3 ubiquitin protein ligase
10198	LINC00094	-0.2033	0.4239	long intergenic non-protein coding RNA 94
10199	KCNS1	-0.2033	0.4923	potassium voltage-gated channel, delayed-rectifier, subfamily S, member 1
10200	ITGA4	-0.2033	0.4181	integrin, alpha 4 (antigen CD49D, alpha 4 subunit of VLA-4 receptor)
10201	GCC1	-0.2033	0.3041	GRIP and coiled-coil domain containing 1
10202	DARS	-0.2033	0.4408	aspartyl-tRNA synthetase
10203	ALDH6A1	-0.2033	0.3939	aldehyde dehydrogenase 6 family, member A1
10204	AKR1D1	-0.2033	0.4312	aldo-keto reductase family 1, member D1 (delta 4-3-ketosteroid-5-beta-reductase)
10205	USP45	-0.2037	0.4232	ubiquitin specific peptidase 45
10206	SDCCAG8	-0.2037	0.3898	serologically defined colon cancer antigen 8
10207	LYSMD1	-0.2037	0.3136	LysM, putative peptidoglycan-binding, domain containing 1
10208	LRG1	-0.2037	0.4534	leucine-rich alpha-2-glycoprotein 1
10209	SMARCAL1	-0.2042	0.4012	SWI/SNF related, matrix associated, actin dependent regulator of chromatin, subfamily a-like 1
10210	PPP2R5B	-0.2042	0.4234	protein phosphatase 2, regulatory subunit B', beta
10211	PITPNA	-0.2042	0.3924	phosphatidylinositol transfer protein, alpha
10212	MYH3	-0.2042	0.3703	myosin, heavy chain 3, skeletal muscle, embryonic
10213	MCOLN1	-0.2042	0.3661	mucolipin 1
10214	FMNL1	-0.2042	0.4482	formin-like 1
10215	ATP6V0A1	-0.2042	0.3167	ATPase, H+ transporting, lysosomal V0 subunit a1
10216	UNC45B	-0.205	0.4995	unc-45 homolog B (C. elegans)
10217	TAC1	-0.205	0.3486	tachykinin, precursor 1
10218	STAMBPL1	-0.205	0.4063	STAM binding protein-like 1
10219	NUDT5	-0.205	0.4245	nudix (nucleoside diphosphate linked moiety X)-type motif 5
10220	LEFTY1	-0.205	0.4696	left-right determination factor 1
10221	GBX2	-0.205	0.4962	gastrulation brain homeobox 2
10222	COX4I2	-0.205	0.5046	cytochrome c oxidase subunit IV isoform 2 (lung)
10223	CD101	-0.205	0.4511	CD101 molecule
10224	APBB3	-0.205	0.3946	amyloid beta (A4) precursor protein-binding, family B, member 3
10225	ADCY10P1	-0.205	0.3762	adenylate cyclase 10 (soluble) pseudogene 1
10226	ITGB1BP1	-0.2058	0.427	integrin beta 1 binding protein 1
10227	INSRR	-0.2058	0.5089	insulin receptor-related receptor
10228	CENPJ	-0.2058	0.4067	centromere protein J
10229	ZNF404	-0.2062	0.4116	zinc finger protein 404
10230	TLR10	-0.2062	0.4032	toll-like receptor 10
10231	LOC389458	-0.2062	0.5007	uncharacterized LOC389458
10232	AXDND1	-0.2062	0.4296	axonemal dynein light chain domain containing 1
10233	STX2	-0.2067	0.3665	syntaxin 2
10234	MAU2	-0.2067	0.3622	MAU2 chromatid cohesion factor homolog (C. elegans)
10235	FAM50A	-0.2067	0.3987	family with sequence similarity 50, member A
10236	EPN1	-0.2067	0.4707	epsin 1
10237	B4GALT3	-0.2067	0.342	UDP-Gal:betaGlcNAc beta 1,4- galactosyltransferase, polypeptide 3
10238	WNK1	-0.2075	0.3779	WNK lysine deficient protein kinase 1
10239	ULK1	-0.2075	0.4021	unc-51-like kinase 1 (C. elegans)
10240	TPH2	-0.2075	0.49	tryptophan hydroxylase 2
10241	TP73	-0.2075	0.4996	tumor protein p73
10242	TIGIT	-0.2075	0.4116	T cell immunoreceptor with Ig and ITIM domains
10243	TAS2R8	-0.2075	0.4569	taste receptor, type 2, member 8
10244	PPIL3	-0.2075	0.4236	peptidylprolyl isomerase (cyclophilin)-like 3
10245	PLEKHH3	-0.2075	0.4606	pleckstrin homology domain containing, family H (with MyTH4 domain) member 3
10246	PIM3	-0.2075	0.3906	pim-3 oncogene

10247	MRPL38	-0.2075	0.4216	mitochondrial ribosomal protein L38
10248	LSP1	-0.2075	0.4457	lymphocyte-specific protein 1
10249	LOC158696	-0.2075	0.4206	uncharacterized LOC158696
10250	LILRB4	-0.2075	0.4512	leukocyte immunoglobulin-like receptor, subfamily B (with TM and ITIM domains), member 4
10251	FRAT2	-0.2075	0.4152	frequently rearranged in advanced T-cell lymphomas 2
10252	FAM122A	-0.2075	0.3961	family with sequence similarity 122A
10253	CPT1C	-0.2075	0.4044	carnitine palmitoyltransferase 1C
10254	C20orf112	-0.2075	0.2927	chromosome 20 open reading frame 112
10255	TOX4	-0.2083	0.3862	TOX high mobility group box family member 4
10256	PNLIPRP2	-0.2083	0.4565	pancreatic lipase-related protein 2
10257	NRSN2	-0.2083	0.4151	neurensin 2
10258	NFATC3	-0.2083	0.3884	nuclear factor of activated T-cells, cytoplasmic, calcineurin-dependent 3
10259	MLLT4-AS1	-0.2083	0.4592	MLLT4 antisense RNA 1 (non-protein coding)
10260	MARK4	-0.2083	0.3738	MAP/microtubule affinity-regulating kinase 4
10261	IQCG	-0.2083	0.3706	IQ motif containing G
10262	FADS3	-0.2083	0.3501	fatty acid desaturase 3
10263	DPH5	-0.2083	0.4309	DPH5 homolog (S. cerevisiae)
10264	DFFA	-0.2083	0.3781	DNA fragmentation factor, 45kDa, alpha polypeptide
10265	C1orf21	-0.2083	0.2772	chromosome 1 open reading frame 21
10266	C11orf10	-0.2083	0.433	chromosome 11 open reading frame 10
10267	AKTIP	-0.2083	0.4095	AKT interacting protein
10268	AHCTF1	-0.2083	0.4357	AT hook containing transcription factor 1
10269	ADAM20	-0.2083	0.4888	ADAM metallopeptidase domain 20
10270	ACTG1	-0.2083	0.4097	actin, gamma 1
10271	ABCD2	-0.2083	0.3374	ATP-binding cassette, sub-family D (ALD), member 2
10272	LOC220077	-0.2086	0.9999	dedicator of cytokinesis 1 pseudogene
10273	ZNF792	-0.2087	0.3649	zinc finger protein 792
10274	NMNAT3	-0.2087	0.3975	nicotinamide nucleotide adenylyltransferase 3
10275	NEK6	-0.2087	0.348	NIMA (never in mitosis gene a)-related kinase 6
10276	FSIP1	-0.2087	0.2984	fibrous sheath interacting protein 1
10277	FAM200B	-0.2087	0.4136	family with sequence similarity 200, member B
10278	EGFEM1P	-0.2087	0.3066	EGF-like and EMI domain containing 1, pseudogene
10279	AKD1	-0.2087	0.3683	adenylate kinase domain containing 1
10280	TMEM120B	-0.2092	0.3822	transmembrane protein 120B
10281	SOX18	-0.2092	0.4497	SRY (sex determining region Y)-box 18
10282	RSF1	-0.2092	0.4238	remodeling and spacing factor 1
10283	MKNK1	-0.2092	0.3537	MAP kinase interacting serine/threonine kinase 1
10284	B3GALT1	-0.2092	0.4542	UDP-Gal:betaGlcNAc beta 1,3-galactosyltransferase, polypeptide 1
10285	ATOH1	-0.2092	0.4899	atonal homolog 1 (Drosophila)
10286	ADCK3	-0.2092	0.3535	aarF domain containing kinase 3
10287	VSX1	-0.21	0.5237	visual system homeobox 1
10288	STX16	-0.21	0.4187	syntaxin 16
10289	SAA3P	-0.21	0.4654	serum amyloid A3 pseudogene
10290	RANBP10	-0.21	0.3487	RAN binding protein 10
10291	RAB27A	-0.21	0.4137	RAB27A, member RAS oncogene family
10292	P2RX1	-0.21	0.471	purinergic receptor P2X, ligand-gated ion channel, 1
10293	NXPH4	-0.21	0.4865	neurexophilin 4
10294	MCF2	-0.21	0.3936	MCF.2 cell line derived transforming sequence
10295	LOC284578	-0.21	0.9999	uncharacterized LOC284578
10296	JUND	-0.21	0.3681	jun D proto-oncogene
10297	JMJD4	-0.21	0.3985	jumonji domain containing 4
10298	GAS2L1	-0.21	0.4093	growth arrest-specific 2 like 1
10299	CYP2C8	-0.21	0.2911	cytochrome P450, family 2, subfamily C, polypeptide 8
10300	CRH	-0.21	0.4788	corticotropin releasing hormone
10301	CDH26	-0.21	0.4719	cadherin 26
10302	TANC2	-0.2108	0.3295	tetratricopeptide repeat, ankyrin repeat and coiled-coil containing 2
10303	SPOCK3	-0.2108	0.3977	sparc/osteonectin, cwcv and kazal-like domains proteoglycan (testican) 3
10304	SERPINA5	-0.2108	0.415	serpin peptidase inhibitor, clade A (alpha-1 antiproteinase, antitrypsin), member 5
10305	RSAD2	-0.2108	0.3796	radical S-adenosyl methionine domain containing 2
10306	RCVRN	-0.2108	0.4896	recoverin
10307	PTPRR	-0.2108	0.3663	protein tyrosine phosphatase, receptor type, R
10308	LANCL1	-0.2108	0.4235	LanC lantibiotic synthetase component C-like 1 (bacterial)
10309	KIAA1598	-0.2108	0.4129	KIAA1598
10310	DYNLRB1	-0.2108	0.4004	dynein, light chain, roadblock-type 1
10311	CLCN1	-0.2108	0.5097	chloride channel, voltage-sensitive 1
10312	ALPI	-0.2108	0.5151	alkaline phosphatase, intestinal
10313	ABCE1	-0.2108	0.457	ATP-binding cassette, sub-family E (OABP), member 1
10314	TREML4	-0.2113	0.4691	triggering receptor expressed on myeloid cells-like 4
10315	NGEF	-0.2113	0.4463	neuronal guanine nucleotide exchange factor
10316	KRTAP13-1	-0.2113	0.5217	keratin associated protein 13-1
10317	KLHL34	-0.2113	0.4785	kelch-like 34 (Drosophila)
10318	ABRA	-0.2113	0.4763	actin-binding Rho activating protein
10319	SUGT1P1	-0.2114	0.2431	suppressor of G2 allele of SKP1 (S. cerevisiae) pseudogene 1
10320	XPOT	-0.2117	0.4485	exportin, tRNA (nuclear export receptor for tRNAs)
10321	TTLL1	-0.2117	0.317	tubulin tyrosine ligase-like family, member 1
10322	TSC22D4	-0.2117	0.4166	TSC22 domain family, member 4
10323	TNFSF11	-0.2117	0.454	tumor necrosis factor (ligand) superfamily, member 11
10324	SLC6A7	-0.2117	0.5012	solute carrier family 6 (neurotransmitter transporter, L-proline), member 7
10325	RAPGEF4	-0.2117	0.3347	Rap guanine nucleotide exchange factor (GEF) 4
10326	POLR3E	-0.2117	0.4353	polymerase (RNA) III (DNA directed) polypeptide E (80kD)
10327	PAX7	-0.2117	0.5104	paired box 7
10328	PARP6	-0.2117	0.3466	poly (ADP-ribose) polymerase family, member 6
10329	LGALS14	-0.2117	0.4801	lectin, galactoside-binding, soluble, 14
10330	C12orf35	-0.2117	0.4274	chromosome 12 open reading frame 35

10331	C11orf68	-0.2117	0.3908	chromosome 11 open reading frame 68
10332	VPREB1	-0.2125	0.4707	pre-B lymphocyte 1
10333	TTC7A	-0.2125	0.3432	tetratricopeptide repeat domain 7A
10334	TMEM252	-0.2125	0.5176	transmembrane protein 252
10335	SLC6A5	-0.2125	0.5078	solute carrier family 6 (neurotransmitter transporter, glycine), member 5
10336	SLC25A51	-0.2125	0.3736	solute carrier family 25, member 51
10337	REG3A	-0.2125	0.4849	regenerating islet-derived 3 alpha
10338	PSAPL1	-0.2125	0.5298	prosaposin-like 1 (gene/pseudogene)
10339	PRPF40A	-0.2125	0.4433	PRP40 pre-mRNA processing factor 40 homolog A (S. cerevisiae)
10340	PCDHA9	-0.2125	0.4497	protocadherin alpha 9
10341	ORAI1	-0.2125	0.3808	ORAI calcium release-activated calcium modulator 1
10342	MRPS16	-0.2125	0.4326	mitochondrial ribosomal protein S16
10343	MRPL10	-0.2125	0.4104	mitochondrial ribosomal protein L10
10344	MOSPD3	-0.2125	0.4312	motile sperm domain containing 3
10345	KLRC3	-0.2125	0.2607	killer cell lectin-like receptor subfamily C, member 3
10346	IL29	-0.2125	0.5317	interleukin 29 (interferon, lambda 1)
10347	IGHMBP2	-0.2125	0.4544	immunoglobulin mu binding protein 2
10348	CRISP1	-0.2125	0.4855	cysteine-rich secretory protein 1
10349	BAI2	-0.2125	0.4346	brain-specific angiogenesis inhibitor 2
10350	OR10A3	-0.2129	0.5048	olfactory receptor, family 10, subfamily A, member 3
10351	CNPY1	-0.2129	0.5037	canopy 1 homolog (zebrafish)
10352	WDR96	-0.2133	0.388	WD repeat domain 96
10353	SULT2A1	-0.2133	0.4791	sulfotransferase family, cytosolic, 2A, dehydroepiandrosterone (DHEA)-preferring, member 1
10354	PPP1R1A	-0.2133	0.4671	protein phosphatase 1, regulatory (inhibitor) subunit 1A
10355	MGAT5	-0.2133	0.3431	mannosyl (alpha-1,6-)-glycoprotein beta-1,6-N-acetyl-glucosaminyltransferase
10356	IPO9	-0.2133	0.3975	importin 9
10357	FGL1	-0.2133	0.4636	fibrinogen-like 1
10358	CNGB3	-0.2133	0.5064	cyclic nucleotide gated channel beta 3
10359	CHMP5	-0.2133	0.449	charged multivesicular body protein 5
10360	CA1	-0.2133	0.4771	carbonic anhydrase I
10361	BAIAP2L2	-0.2138	0.4867	BAI1-associated protein 2-like 2
10362	ZNF131	-0.2142	0.4313	zinc finger protein 131
10363	UBA3	-0.2142	0.4494	ubiquitin-like modifier activating enzyme 3
10364	SLC10A1	-0.2142	0.5047	solute carrier family 10 (sodium/bile acid cotransporter family), member 1
10365	S1PR2	-0.2142	0.4821	sphingosine-1-phosphate receptor 2
10366	NDUFV1	-0.2142	0.4134	NADH dehydrogenase (ubiquinone) flavoprotein 1, 51kDa
10367	KIAA0284	-0.2142	0.4079	KIAA0284
10368	GNA12	-0.2142	0.3281	guanine nucleotide binding protein (G protein) alpha 12
10369	C1orf109	-0.2142	0.444	chromosome 1 open reading frame 109
10370	LOC642852	-0.2143	0.3685	uncharacterized LOC642852
10371	SPEG	-0.2144	0.4953	SPEG complex locus
10372	PRAMEF11	-0.2145	0.4971	PRAME family member 11
10373	SLC7A10	-0.215	0.4859	solute carrier family 7 (neutral amino acid transporter light chain, asc system), member 10
10374	SAMD14	-0.215	0.4823	sterile alpha motif domain containing 14
10375	S1PR5	-0.215	0.4782	sphingosine-1-phosphate receptor 5
10376	RTKN2	-0.215	0.3953	rhotekin 2
10377	RP9P	-0.215	0.268	retinitis pigmentosa 9 pseudogene
10378	PTDSS2	-0.215	0.3815	phosphatidylserine synthase 2
10379	NXPE1	-0.215	0.5088	neurexophilin and PC-esterase domain family, member 1
10380	MMAB	-0.215	0.3547	methylmalonic aciduria (cobalamin deficiency) cblB type
10381	KLHL13	-0.215	0.3593	kelch-like 13 (Drosophila)
10382	HDAC1	-0.215	0.4189	histone deacetylase 1
10383	DHX35	-0.215	0.3992	DEAH (Asp-Glu-Ala-His) box polypeptide 35
10384	CHRNB2	-0.215	0.5083	cholinergic receptor, nicotinic, beta 2 (neuronal)
10385	C1QC	-0.215	0.4347	complement component 1, q subcomponent, C chain
10386	BRMS1	-0.215	0.3951	breast cancer metastasis suppressor 1
10387	AMN	-0.215	0.5287	amnionless homolog (mouse)
10388	ALKBH5	-0.215	0.3219	alkB, alkylation repair homolog 5 (E. coli)
10389	AGPS	-0.215	0.4408	alkylglycerone phosphate synthase
10390	AGBL5	-0.215	0.3619	ATP/GTP binding protein-like 5
10391	ZNF292	-0.2158	0.44	zinc finger protein 292
10392	UBOX5	-0.2158	0.4093	U-box domain containing 5
10393	THAP11	-0.2158	0.4098	THAP domain containing 11
10394	SRRM1	-0.2158	0.423	serine/arginine repetitive matrix 1
10395	RORB	-0.2158	0.3548	RAR-related orphan receptor B
10396	NPTX1	-0.2158	0.4101	neuronal pentraxin I
10397	IRS2	-0.2158	0.3623	insulin receptor substrate 2
10398	IFT27	-0.2158	0.3838	intraflagellar transport 27 homolog (Chlamydomonas)
10399	CSF2RB	-0.2158	0.431	colony stimulating factor 2 receptor, beta, low-affinity (granulocyte-macrophage)
10400	CDKL5	-0.2158	0.3935	cyclin-dependent kinase-like 5
10401	CCDC51	-0.2158	0.401	coiled-coil domain containing 51
10402	C12orf10	-0.2158	0.4193	chromosome 12 open reading frame 10
10403	ABCC10	-0.2158	0.3565	ATP-binding cassette, sub-family C (CFTR/MRP), member 10
10404	USP35	-0.2163	0.3869	ubiquitin specific peptidase 35
10405	SMYD1	-0.2163	0.5162	SET and MYND domain containing 1
10406	PPAPDC3	-0.2163	0.445	phosphatidic acid phosphatase type 2 domain containing 3
10407	POLE4	-0.2163	0.4083	polymerase (DNA-directed), epsilon 4, accessory subunit
10408	MRPL30	-0.2163	0.4285	mitochondrial ribosomal protein L30
10409	GUSBP5	-0.2163	0.3168	glucuronidase, beta pseudogene 5
10410	C3orf23	-0.2163	0.4197	chromosome 3 open reading frame 23
10411	C1QTNF9	-0.2163	0.3819	C1q and tumor necrosis factor related protein 9
10412	LOC100130331	-0.2164	0.4779	POTE ankyrin domain family, member F pseudogene
10413	ZNF225	-0.2167	0.3955	zinc finger protein 225
10414	SPATA13	-0.2167	0.3461	spermatogenesis associated 13

10415	SP140	-0.2167	0.4066	SP140 nuclear body protein
10416	PRDX2	-0.2167	0.4026	peroxiredoxin 2
10417	PIK3R3	-0.2167	0.3097	phosphoinositide-3-kinase, regulatory subunit 3 (gamma)
10418	NPM3	-0.2167	0.4256	nucleophosmin/nucleoplasmin 3
10419	MAP3K4	-0.2167	0.42	mitogen-activated protein kinase kinase kinase 4
10420	IRF5	-0.2167	0.4453	interferon regulatory factor 5
10421	GPC5	-0.2167	0.3697	glypican 5
10422	C6orf162	-0.2167	0.4068	chromosome 6 open reading frame 162
10423	LINGO3	-0.2171	0.5263	leucine rich repeat and Ig domain containing 3
10424	LINC00452	-0.2171	0.5337	long intergenic non-protein coding RNA 452
10425	ZNF704	-0.2175	0.3339	zinc finger protein 704
10426	ZNF673	-0.2175	0.4254	zinc finger family member 673
10427	TOLLIP	-0.2175	0.366	toll interacting protein
10428	OR2B6	-0.2175	0.4433	olfactory receptor, family 2, subfamily B, member 6
10429	NKD2	-0.2175	0.4643	naked cuticle homolog 2 (Drosophila)
10430	NDUFS6	-0.2175	0.4304	NADH dehydrogenase (ubiquinone) Fe-S protein 6, 13kDa (NADH-coenzyme Q reductase)
10431	MCMBP	-0.2175	0.4378	minichromosome maintenance complex binding protein
10432	MARCH3	-0.2175	0.2281	membrane-associated ring finger (C3HC4) 3, E3 ubiquitin protein ligase
10433	LRFN4	-0.2175	0.4145	leucine rich repeat and fibronectin type III domain containing 4
10434	EIF3B	-0.2175	0.4255	eukaryotic translation initiation factor 3, subunit B
10435	DUSP12	-0.2175	0.4386	dual specificity phosphatase 12
10436	DEFB118	-0.2175	0.5217	defensin, beta 118
10437	COG1	-0.2175	0.3585	component of oligomeric golgi complex 1
10438	ZCCHC11	-0.2183	0.4194	zinc finger, CCHC domain containing 11
10439	ZBBX	-0.2183	0.3734	zinc finger, B-box domain containing
10440	SNRNP35	-0.2183	0.368	small nuclear ribonucleoprotein 35kDa (U11/U12)
10441	PRSS1	-0.2183	0.4506	protease, serine, 1 (trypsin 1)
10442	MUSK	-0.2183	0.4968	muscle, skeletal, receptor tyrosine kinase
10443	LDHB	-0.2183	0.4379	lactate dehydrogenase B
10444	GPR21	-0.2183	0.4385	G protein-coupled receptor 21
10445	ECD	-0.2183	0.4189	ecdysoneless homolog (Drosophila)
10446	BBS7	-0.2183	0.4216	Bardet-Biedl syndrome 7
10447	BATF	-0.2183	0.4262	basic leucine zipper transcription factor, ATF-like
10448	MAGEA5	-0.2186	0.4814	melanoma antigen family A, 5
10449	LOC284276	-0.2186	0.9999	uncharacterized LOC284276
10450	SLC25A30	-0.2188	0.3707	solute carrier family 25, member 30
10451	RBM11	-0.2188	0.3215	RNA binding motif protein 11
10452	PFN4	-0.2188	0.2781	profilin family, member 4
10453	HSDL1	-0.2188	0.3972	hydroxysteroid dehydrogenase like 1
10454	C1orf31	-0.2188	0.4312	chromosome 1 open reading frame 31
10455	ADAM3A	-0.2191	0.46	ADAM metallopeptidase domain 3A (pseudogene)
10456	ZEB2	-0.2192	0.4142	zinc finger E-box binding homeobox 2
10457	UCP3	-0.2192	0.493	uncoupling protein 3 (mitochondrial, proton carrier)
10458	RBM25	-0.2192	0.442	RNA binding motif protein 25
10459	CYP2J2	-0.2192	0.2739	cytochrome P450, family 2, subfamily J, polypeptide 2
10460	CTNS	-0.2192	0.3255	cystinosin, lysosomal cystine transporter
10461	ZNF182	-0.22	0.3992	zinc finger protein 182
10462	SPIC	-0.22	0.2004	Spi-C transcription factor (Spi-1/PU.1 related)
10463	SPATA4	-0.22	0.4488	spermatogenesis associated 4
10464	SCARB1	-0.22	0.3414	scavenger receptor class B, member 1
10465	PRKAG3	-0.22	0.5244	protein kinase, AMP-activated, gamma 3 non-catalytic subunit
10466	OLIG3	-0.22	0.4805	oligodendrocyte transcription factor 3
10467	OLFM1	-0.22	0.2672	olfactomedin 1
10468	MRPL36	-0.22	0.4317	mitochondrial ribosomal protein L36
10469	LHX4	-0.22	0.4823	LIM homeobox 4
10470	IARS	-0.22	0.4384	isoleucyl-tRNA synthetase
10471	HSPB9	-0.22	0.5029	heat shock protein, alpha-crystallin-related, B9
10472	HOOK1	-0.22	0.355	hook homolog 1 (Drosophila)
10473	FFAR1	-0.22	0.5256	free fatty acid receptor 1
10474	FAM118A	-0.22	0.2931	family with sequence similarity 118, member A
10475	EMR1	-0.22	0.4165	egf-like module containing, mucin-like, hormone receptor-like 1
10476	C3orf70	-0.22	0.3247	chromosome 3 open reading frame 70
10477	C15orf60	-0.22	0.5031	chromosome 15 open reading frame 60
10478	ANKRD18A	-0.22	0.9999	ankyrin repeat domain 18A
10479	AGFG1	-0.22	0.4309	ArfGAP with FG repeats 1
10480	POU4F1	-0.2208	0.4109	POU class 4 homeobox 1
10481	POMT1	-0.2208	0.343	protein-O-mannosyltransferase 1
10482	NGFRAP1	-0.2208	0.3814	nerve growth factor receptor (TNFRSF16) associated protein 1
10483	KAT2B	-0.2208	0.4161	K(lysine) acetyltransferase 2B
10484	FAM131B	-0.2208	0.4373	family with sequence similarity 131, member B
10485	SCFD2	-0.2212	0.3656	sec1 family domain containing 2
10486	ORAOV1	-0.2212	0.3338	oral cancer overexpressed 1
10487	INIP	-0.2212	0.4266	INTS3 and NABP interacting protein
10488	ADPRHL1	-0.2212	0.4638	ADP-ribosylhydrolase like 1
10489	SH3RF3-AS1	-0.2214	0.3102	SH3RF3 antisense RNA 1 (non-protein coding)
10490	LOC100129935	-0.2214	0.4915	lectin, galactoside-binding, soluble, 14 pseudogene
10491	SCAPER	-0.2217	0.4069	S-phase cyclin A-associated protein in the ER
10492	RTN1	-0.2217	0.3193	reticulon 1
10493	LRRC8B	-0.2217	0.3987	leucine rich repeat containing 8 family, member B
10494	HIST1H2BL	-0.2217	0.302	histone cluster 1, H2bl
10495	HCG4B	-0.2217	0.4113	HLA complex group 4B (non-protein coding)
10496	FBXL18	-0.2217	0.377	F-box and leucine-rich repeat protein 18
10497	WFIKKN1	-0.2225	0.5091	WAP, follistatin/kazal, immunoglobulin, kunitz and netrin domain containing 1
10498	RAD9A	-0.2225	0.4027	RAD9 homolog A (S. pombe)

10499	RAB8B	-0.2225	0.4128	RAB8B, member RAS oncogene family
10500	PRLHR	-0.2225	0.509	prolactin releasing hormone receptor
10501	PPP2R5C	-0.2225	0.4368	protein phosphatase 2, regulatory subunit B', gamma
10502	LILRB1	-0.2225	0.4478	leukocyte immunoglobulin-like receptor, subfamily B (with TM and ITIM domains), member 1
10503	KLHL12	-0.2225	0.413	kelch-like 12 (Drosophila)
10504	KIAA1908	-0.2225	0.3446	uncharacterized LOC114796
10505	FGR	-0.2225	0.4453	Gardner-Rasheed feline sarcoma viral (v-fgr) oncogene homolog
10506	EFNA3	-0.2225	0.4174	ephrin-A3
10507	DMBX1	-0.2225	0.4893	diencephalon/mesencephalon homeobox 1
10508	DAAM2	-0.2225	0.3901	dishevelled associated activator of morphogenesis 2
10509	CSDE1	-0.2225	0.4267	cold shock domain containing E1, RNA-binding
10510	CENPT	-0.2225	0.3765	centromere protein T
10511	CCDC87	-0.2225	0.4708	coiled-coil domain containing 87
10512	PET100	-0.2229	0.4184	PET100 homolog (S. cerevisiae)
10513	ZNF230	-0.2233	0.3931	zinc finger protein 230
10514	SUZ12	-0.2233	0.4394	suppressor of zeste 12 homolog (Drosophila)
10515	SLC27A3	-0.2233	0.3139	solute carrier family 27 (fatty acid transporter), member 3
10516	PPIE	-0.2233	0.4038	peptidylprolyl isomerase E (cyclophilin E)
10517	POU3F1	-0.2233	0.4888	POU class 3 homeobox 1
10518	PIWIL1	-0.2233	0.4414	piwi-like 1 (Drosophila)
10519	KCNF1	-0.2233	0.4871	potassium voltage-gated channel, subfamily F, member 1
10520	HPYR1	-0.2233	0.9996	Helicobacter pylori responsive 1 (non-protein coding)
10521	GRM8	-0.2233	0.4464	glutamate receptor, metabotropic 8
10522	GHRHR	-0.2233	0.5006	growth hormone releasing hormone receptor
10523	FASTKD3	-0.2233	0.4477	FAST kinase domains 3
10524	CHMP2B	-0.2233	0.4455	charged multivesicular body protein 2B
10525	MAPT-AS1	-0.2237	0.5138	MAPT antisense RNA 1 (non-protein coding)
10526	LOC441666	-0.2237	0.308	zinc finger protein 91 pseudogene
10527	ANKRD16	-0.2237	0.3654	ankyrin repeat domain 16
10528	A2ML1	-0.2237	0.4934	alpha-2-macroglobulin-like 1
10529	SMYD5	-0.2242	0.3732	SMYD family member 5
10530	SMARCC2	-0.2242	0.348	SWI/SNF related, matrix associated, actin dependent regulator of chromatin, subfamily c, member 2
10531	RGS14	-0.2242	0.4306	regulator of G-protein signaling 14
10532	PP14571	-0.2242	0.5013	uncharacterized LOC100130449
10533	HS2ST1	-0.2242	0.4374	heparan sulfate 2-O-sulfotransferase 1
10534	GUF1	-0.2242	0.4308	GUF1 GTPase homolog (S. cerevisiae)
10535	F2	-0.2242	0.4937	coagulation factor II (thrombin)
10536	BCAT1	-0.2242	0.3643	branched chain amino-acid transaminase 1, cytosolic
10537	ZNF804A	-0.225	0.2848	zinc finger protein 804A
10538	SPAG8	-0.225	0.4595	sperm associated antigen 8
10539	MRPL19	-0.225	0.4494	mitochondrial ribosomal protein L19
10540	HIPK2	-0.225	0.2694	homeodomain interacting protein kinase 2
10541	ENY2	-0.225	0.4285	enhancer of yellow 2 homolog (Drosophila)
10542	C3orf14	-0.225	0.3969	chromosome 3 open reading frame 14
10543	C20orf111	-0.225	0.4018	chromosome 20 open reading frame 111
10544	BCAS3	-0.225	0.2751	breast carcinoma amplified sequence 3
10545	TMEM222	-0.2258	0.3824	transmembrane protein 222
10546	NACAP1	-0.2258	0.4461	nascent-polypeptide-associated complex alpha polypeptide pseudogene 1
10547	MGAT4A	-0.2258	0.3653	mannosyl (alpha-1,3-)-glycoprotein beta-1,4-N-acetylglucosaminyltransferase, isozyme A
10548	DDX5	-0.2258	0.4332	DEAD (Asp-Glu-Ala-Asp) box helicase 5
10549	AMBP	-0.2258	0.4633	alpha-1-microglobulin/bikunin precursor
10550	TIMMDC1	-0.2262	0.4032	translocase of inner mitochondrial membrane domain containing 1
10551	SPAG17	-0.2262	0.3693	sperm associated antigen 17
10552	SORCS2	-0.2262	0.4089	sortilin-related VPS10 domain containing receptor 2
10553	PCDHAC2	-0.2262	0.4857	protocadherin alpha subfamily C, 2
10554	PAPD5	-0.2262	0.3981	PAP associated domain containing 5
10555	CEP89	-0.2262	0.2627	centrosomal protein 89kDa
10556	ZCCHC14	-0.2267	0.3253	zinc finger, CCHC domain containing 14
10557	TSNAX	-0.2267	0.4435	translin-associated factor X
10558	TLR7	-0.2267	0.357	toll-like receptor 7
10559	NUP133	-0.2267	0.4276	nucleoporin 133kDa
10560	EIF3I	-0.2267	0.4258	eukaryotic translation initiation factor 3, subunit I
10561	DESI1	-0.2267	0.3674	desumoylating isopeptidase 1
10562	CRYBB1	-0.2267	0.4872	crystallin, beta B1
10563	CD79A	-0.2267	0.4739	CD79a molecule, immunoglobulin-associated alpha
10564	CCL4	-0.2267	0.3835	chemokine (C-C motif) ligand 4
10565	MAGEA12	-0.2271	0.3568	melanoma antigen family A, 12
10566	GRIP2	-0.2271	0.5161	glutamate receptor interacting protein 2
10567	WNT7A	-0.2275	0.4483	wingless-type MMTV integration site family, member 7A
10568	TTLL2	-0.2275	0.5118	tubulin tyrosine ligase-like family, member 2
10569	TNRC6A	-0.2275	0.4019	trinucleotide repeat containing 6A
10570	STARD7	-0.2275	0.429	StAR-related lipid transfer (START) domain containing 7
10571	SLC27A5	-0.2275	0.4063	solute carrier family 27 (fatty acid transporter), member 5
10572	PIM2	-0.2275	0.2999	pim-2 oncogene
10573	MPLKIP	-0.2275	0.4284	M-phase specific PLK1 interacting protein
10574	HOXD4	-0.2275	0.4715	homeobox D4
10575	FAM89B	-0.2275	0.3663	family with sequence similarity 89, member B
10576	EXOC3	-0.2275	0.3432	exocyst complex component 3
10577	COX11	-0.2275	0.4299	COX11 cytochrome c oxidase assembly homolog (yeast)
10578	BPIFB4	-0.2275	0.5183	BPI fold containing family B, member 4
10579	MAGEH1	-0.2283	0.3982	melanoma antigen family H, 1
10580	INPP1	-0.2283	0.3753	inositol polyphosphate-1-phosphatase
10581	HP	-0.2283	0.341	haptoglobin
10582	BRIX1	-0.2283	0.4476	BRX1, biogenesis of ribosomes, homolog (S. cerevisiae)

10583	ATP2C1	-0.2283	0.4304	ATPase, Ca++ transporting, type 2C, member 1
10584	NRARP	-0.2286	0.3403	NOTCH-regulated ankyrin repeat protein
10585	FAM228B	-0.2286	0.9999	family with sequence similarity 228, member B
10586	TMEM155	-0.2288	0.4172	transmembrane protein 155
10587	OR5P3	-0.2288	0.5016	olfactory receptor, family 5, subfamily P, member 3
10588	ZCWPW1	-0.2292	0.4176	zinc finger, CW type with PWWP domain 1
10589	ST13	-0.2292	0.4332	suppression of tumorigenicity 13 (colon carcinoma) (Hsp70 interacting protein)
10590	SOAT2	-0.2292	0.4878	sterol O-acyltransferase 2
10591	SDC3	-0.2292	0.3436	syndecan 3
10592	RUNX3	-0.2292	0.3653	runt-related transcription factor 3
10593	RNF32	-0.2292	0.2843	ring finger protein 32
10594	RDX	-0.2292	0.4206	radixin
10595	PPWD1	-0.2292	0.4392	peptidylprolyl isomerase domain and WD repeat containing 1
10596	HSBP1	-0.2292	0.4143	heat shock factor binding protein 1
10597	DUS1L	-0.2292	0.3964	dihydrouridine synthase 1-like (S. cerevisiae)
10598	DOCK4	-0.2292	0.3893	dedicator of cytokinesis 4
10599	CD47	-0.2292	0.4177	CD47 molecule
10600	CARD8	-0.2292	0.3986	caspase recruitment domain family, member 8
10601	ZNF837	-0.23	0.4898	zinc finger protein 837
10602	ZNF434	-0.23	0.2878	zinc finger protein 434
10603	WFDC10B	-0.23	0.5121	WAP four-disulfide core domain 10B
10604	USP47	-0.23	0.4197	ubiquitin specific peptidase 47
10605	TDRD6	-0.23	0.3407	tudor domain containing 6
10606	PDE6A	-0.23	0.4763	phosphodiesterase 6A, cGMP-specific, rod, alpha
10607	OR5P2	-0.23	0.4708	olfactory receptor, family 5, subfamily P, member 2
10608	MRPL27	-0.23	0.4257	mitochondrial ribosomal protein L27
10609	MFF	-0.23	0.4269	mitochondrial fission factor
10610	KLK15	-0.23	0.5137	kallikrein-related peptidase 15
10611	KCNQ1DN	-0.23	0.5076	KCNQ1 downstream neighbor (non-protein coding)
10612	GMIP	-0.23	0.4111	GEM interacting protein
10613	GHRL	-0.23	0.481	ghrelin/obestatin prepropeptide
10614	FBXO46	-0.23	0.3554	F-box protein 46
10615	EIF4G3	-0.23	0.4015	eukaryotic translation initiation factor 4 gamma, 3
10616	DQX1	-0.23	0.471	DEAQ box RNA-dependent ATPase 1
10617	DPP3	-0.23	0.3815	dipeptidyl-peptidase 3
10618	CLDN19	-0.23	0.5197	claudin 19
10619	CDS2	-0.23	0.3702	CDP-diacylglycerol synthase (phosphatidate cytidylyltransferase) 2
10620	CAPN11	-0.23	0.4627	calpain 11
10621	BCOR	-0.23	0.4056	BCL6 corepressor
10622	ZNF208	-0.2308	0.2879	zinc finger protein 208
10623	XCR1	-0.2308	0.5002	chemokine (C motif) receptor 1
10624	TTC13	-0.2308	0.4092	tetratricopeptide repeat domain 13
10625	SALL2	-0.2308	0.3323	sal-like 2 (Drosophila)
10626	PRSS21	-0.2308	0.4473	protease, serine, 21 (testisin)
10627	PAFAH1B2	-0.2308	0.2299	platelet-activating factor acetylhydrolase 1b, catalytic subunit 2 (30kDa)
10628	NDUFA9	-0.2308	0.4076	NADH dehydrogenase (ubiquinone) 1 alpha subcomplex, 9, 39kDa
10629	IMPDH1	-0.2308	0.3843	IMP (inosine 5'-monophosphate) dehydrogenase 1
10630	ENOX1	-0.2308	0.2475	ecto-NOX disulfide-thiol exchanger 1
10631	EMG1	-0.2308	0.4407	EMG1 nucleolar protein homolog (S. cerevisiae)
10632	ABCB8	-0.2308	0.4294	ATP-binding cassette, sub-family B (MDR/TAP), member 8
10633	TNFSF13B	-0.2313	0.41	tumor necrosis factor (ligand) superfamily, member 13b
10634	RSPH4A	-0.2313	0.2913	radial spoke head 4 homolog A (Chlamydomonas)
10635	RNF169	-0.2313	0.3885	ring finger protein 169
10636	PCGF5	-0.2313	0.4132	polycomb group ring finger 5
10637	LOC100134229	-0.2313	0.2128	uncharacterized LOC100134229
10638	EPHA8	-0.2313	0.525	EPH receptor A8
10639	ELOVL7	-0.2313	0.3464	ELOVL fatty acid elongase 7
10640	LOC283050	-0.2314	0.4431	uncharacterized LOC283050
10641	LINC00173	-0.2314	0.4143	long intergenic non-protein coding RNA 173
10642	CCDC129	-0.2314	0.5054	coiled-coil domain containing 129
10643	RWDD3	-0.2317	0.4445	RWD domain containing 3
10644	PDE6C	-0.2317	0.3813	phosphodiesterase 6C, cGMP-specific, cone, alpha prime
10645	PAK6	-0.2317	0.3564	p21 protein (Cdc42/Rac)-activated kinase 6
10646	MZT2B	-0.2317	0.3822	mitotic spindle organizing protein 2B
10647	MRPS31	-0.2317	0.4339	mitochondrial ribosomal protein S31
10648	HNRNPA3	-0.2317	0.4401	heterogeneous nuclear ribonucleoprotein A3
10649	GALR1	-0.2317	0.4371	galanin receptor 1
10650	CD1E	-0.2317	0.4098	CD1e molecule
10651	CCR9	-0.2317	0.4018	chemokine (C-C motif) receptor 9
10652	C16orf45	-0.2317	0.2721	chromosome 16 open reading frame 45
10653	ZNF678	-0.2325	0.3952	zinc finger protein 678
10654	SLCO5A1	-0.2325	0.3991	solute carrier organic anion transporter family, member 5A1
10655	PTPN6	-0.2325	0.3736	protein tyrosine phosphatase, non-receptor type 6
10656	LEP	-0.2325	0.4615	leptin
10657	KLHL29	-0.2325	0.2925	kelch-like 29 (Drosophila)
10658	GABBR1	-0.2325	0.3524	gamma-aminobutyric acid (GABA) B receptor, 1
10659	EFTUD1	-0.2325	0.4107	elongation factor Tu GTP binding domain containing 1
10660	DENND5B	-0.2325	0.3439	DENN/MADD domain containing 5B
10661	ABHD10	-0.2325	0.4188	abhydrolase domain containing 10
10662	UCKL1	-0.2333	0.3863	uridine-cytidine kinase 1-like 1
10663	TGIF2	-0.2333	0.3456	TGFB-induced factor homeobox 2
10664	TDP2	-0.2333	0.4438	tyrosyl-DNA phosphodiesterase 2
10665	RFNG	-0.2333	0.4082	RFNG O-fucosylpeptide 3-beta-N-acetylglucosaminyltransferase
10666	RAP2A	-0.2333	0.3952	RAP2A, member of RAS oncogene family

10667	MTCH2	-0.2333	0.4237	mitochondrial carrier 2
10668	KCND3	-0.2333	0.4739	potassium voltage-gated channel, Shal-related subfamily, member 3
10669	GCM1	-0.2333	0.4797	glial cells missing homolog 1 (Drosophila)
10670	CCT6B	-0.2333	0.3623	chaperonin containing TCP1, subunit 6B (zeta 2)
10671	WNK2	-0.2338	0.4711	WNK lysine deficient protein kinase 2
10672	WDR63	-0.2338	0.2375	WD repeat domain 63
10673	TAAR1	-0.2338	0.3559	trace amine associated receptor 1
10674	SEC22C	-0.2338	0.3958	SEC22 vesicle trafficking protein homolog C (S. cerevisiae)
10675	RAX2	-0.2338	0.5241	retina and anterior neural fold homeobox 2
10676	OTUD6B	-0.2338	0.4444	OTU domain containing 6B
10677	LYRM5	-0.2338	0.4148	LYR motif containing 5
10678	LRRN1	-0.2338	0.2968	leucine rich repeat neuronal 1
10679	LOC283588	-0.2338	0.3477	uncharacterized LOC283588
10680	CCDC84	-0.2338	0.3699	coiled-coil domain containing 84
10681	ANKRD9	-0.2338	0.3541	ankyrin repeat domain 9
10682	UQCC	-0.2342	0.3428	ubiquinol-cytochrome c reductase complex chaperone
10683	RASSF1	-0.2342	0.3303	Ras association (RalGDS/AF-6) domain family member 1
10684	PIGL	-0.2342	0.2798	phosphatidylinositol glycan anchor biosynthesis, class L
10685	MLN	-0.2342	0.4937	motilin
10686	KRAS	-0.2342	0.4132	v-Ki-ras2 Kirsten rat sarcoma viral oncogene homolog
10687	CLCN4	-0.2342	0.2231	chloride channel, voltage-sensitive 4
10688	CBS	-0.2342	0.2816	cystathionine-beta-synthase
10689	BMP8B	-0.2342	0.4592	bone morphogenetic protein 8b
10690	AK5	-0.2342	0.2696	adenylate kinase 5
10691	AGER	-0.2342	0.471	advanced glycosylation end product-specific receptor
10692	NSUN5P2	-0.2343	0.396	NOP2/Sun domain family, member 5 pseudogene 2
10693	LOC100287813	-0.2343	0.3637	uncharacterized LOC100287813
10694	ZNF85	-0.235	0.3887	zinc finger protein 85
10695	ZCCHC7	-0.235	0.4264	zinc finger, CCHC domain containing 7
10696	TMEM174	-0.235	0.5149	transmembrane protein 174
10697	RPS4Y1	-0.235	0.2469	ribosomal protein S4, Y-linked 1
10698	RPAP2	-0.235	0.3627	RNA polymerase II associated protein 2
10699	PPP4R1	-0.235	0.411	protein phosphatase 4, regulatory subunit 1
10700	MRPS30	-0.235	0.433	mitochondrial ribosomal protein S30
10701	KLC4	-0.235	0.3823	kinesin light chain 4
10702	HTR6	-0.235	0.5007	5-hydroxytryptamine (serotonin) receptor 6, G protein-coupled
10703	DZIP1	-0.235	0.3312	DAZ interacting protein 1
10704	CLEC4F	-0.235	0.4806	C-type lectin domain family 4, member F
10705	CA7	-0.235	0.5018	carbonic anhydrase VII
10706	ADORA3	-0.235	0.4235	adenosine A3 receptor
10707	YBX1	-0.2357	0.4347	Y box binding protein 1
10708	LOC100133308	-0.2357	0.9999	Ras suppressor protein 1 pseudogene
10709	CLEC4GP1	-0.2357	0.5155	C-type lectin domain family 4, member G pseudogene 1
10710	TRIP12	-0.2358	0.4236	thyroid hormone receptor interactor 12
10711	PTAFR	-0.2358	0.4222	platelet-activating factor receptor
10712	CCT8L2	-0.2358	0.4857	chaperonin containing TCP1, subunit 8 (theta)-like 2
10713	C2orf49	-0.2358	0.3909	chromosome 2 open reading frame 49
10714	ATXN7L3B	-0.2358	0.3525	ataxin 7-like 3B
10715	TSPAN33	-0.2362	0.2812	tetraspanin 33
10716	DCUN1D5	-0.2362	0.4292	DCN1, defective in cullin neddylation 1, domain containing 5 (S. cerevisiae)
10717	C7orf55	-0.2362	0.4239	chromosome 7 open reading frame 55
10718	TYROBP	-0.2367	0.4283	TYRO protein tyrosine kinase binding protein
10719	TRRAP	-0.2367	0.4068	transformation/transcription domain-associated protein
10720	P2RY10	-0.2367	0.3506	purinergic receptor P2Y, G-protein coupled, 10
10721	GLOD4	-0.2367	0.4299	glyoxalase domain containing 4
10722	FGFR3	-0.2367	0.3412	fibroblast growth factor receptor 3
10723	CPB1	-0.2367	0.4079	carboxypeptidase B1 (tissue)
10724	TRBV7-8	-0.2371	0.9999	T cell receptor beta variable 7-8
10725	ZNF134	-0.2375	0.363	zinc finger protein 134
10726	YWHAZ	-0.2375	0.4298	tyrosine 3-monooxygenase/tryptophan 5-monooxygenase activation protein, zeta polypeptide
10727	STPG1	-0.2375	0.1748	sperm-tail PG-rich repeat containing 1
10728	SEPHS2	-0.2375	0.429	selenophosphate synthetase 2
10729	NUDT11	-0.2375	0.3074	nudix (nucleoside diphosphate linked moiety X)-type motif 11
10730	IL10RA	-0.2375	0.4117	interleukin 10 receptor, alpha
10731	IDO1	-0.2375	0.3391	indoleamine 2,3-dioxygenase 1
10732	HELQ	-0.2375	0.4114	helicase, POLQ-like
10733	FOXO4	-0.2375	0.3569	forkhead box O4
10734	EIF4EBP1	-0.2375	0.3737	eukaryotic translation initiation factor 4E binding protein 1
10735	DRD4	-0.2375	0.4816	dopamine receptor D4
10736	CACNA1H	-0.2375	0.4722	calcium channel, voltage-dependent, T type, alpha 1H subunit
10737	C1orf105	-0.2375	0.4692	chromosome 1 open reading frame 105
10738	UQCRB	-0.2383	0.4253	ubiquinol-cytochrome c reductase binding protein
10739	UBXN7	-0.2383	0.4104	UBX domain protein 7
10740	TECPR2	-0.2383	0.2864	tectonin beta-propeller repeat containing 2
10741	RIOK2	-0.2383	0.4348	RIO kinase 2 (yeast)
10742	PIWIL2	-0.2383	0.4547	piwi-like 2 (Drosophila)
10743	KLF12	-0.2383	0.3195	Kruppel-like factor 12
10744	CNOT6	-0.2383	0.4303	CCR4-NOT transcription complex, subunit 6
10745	CLTCL1	-0.2383	0.3722	clathrin, heavy chain-like 1
10746	C9orf62	-0.2383	0.9999	chromosome 9 open reading frame 62
10747	BMP2K	-0.2383	0.3983	BMP2 inducible kinase
10748	ASIP	-0.2383	0.4782	agouti signaling protein
10749	GPR153	-0.2386	0.4975	G protein-coupled receptor 153
10750	FAM71F2	-0.2386	0.4563	family with sequence similarity 71, member F2

10751	ZNF385A	-0.2387	0.432	zinc finger protein 385A
10752	PGBD2	-0.2387	0.3556	piggyBac transposable element derived 2
10753	LRTOMT	-0.2387	0.3148	leucine rich transmembrane and 0-methyltransferase domain containing
10754	LOC283663	-0.2387	0.409	uncharacterized LOC283663
10755	IGSF9	-0.2387	0.4484	immunoglobulin superfamily, member 9
10756	FMO9P	-0.2387	0.4673	flavin containing monooxygenase 9 pseudogene
10757	DMRTA1	-0.2387	0.436	DMRT-like family A1
10758	ZZEF1	-0.2392	0.2929	zinc finger, ZZ-type with EF-hand domain 1
10759	SH3D21	-0.2392	0.4701	SH3 domain containing 21
10760	PTDSS1	-0.2392	0.4018	phosphatidylserine synthase 1
10761	MTG1	-0.2392	0.3591	mitochondrial GTPase 1 homolog (S. cerevisiae)
10762	METTL17	-0.2392	0.4003	methyltransferase like 17
10763	GCK	-0.2392	0.4848	glucokinase (hexokinase 4)
10764	CLDN9	-0.2392	0.4885	claudin 9
10765	ZNF609	-0.24	0.2956	zinc finger protein 609
10766	PTPRN	-0.24	0.4594	protein tyrosine phosphatase, receptor type, N
10767	PPY2	-0.24	0.4961	pancreatic polypeptide 2
10768	NEK8	-0.24	0.4297	NIMA (never in mitosis gene a)- related kinase 8
10769	CELA3A	-0.24	0.488	chymotrypsin-like elastase family, member 3A
10770	YTHDF1	-0.2408	0.4011	YTH domain family, member 1
10771	TOMM6	-0.2408	0.4235	translocase of outer mitochondrial membrane 6 homolog (yeast)
10772	RAG2	-0.2408	0.3632	recombination activating gene 2
10773	RAC2	-0.2408	0.3739	ras-related C3 botulinum toxin substrate 2 (rho family, small GTP binding protein Rac2)
10774	NRN1	-0.2408	0.2348	neuritin 1
10775	NDUFS5	-0.2408	0.4153	NADH dehydrogenase (ubiquinone) Fe-S protein 5, 15kDa (NADH-coenzyme Q reductase)
10776	NCF2	-0.2408	0.3787	neutrophil cytosolic factor 2
10777	METRN	-0.2408	0.4119	meteorin, glial cell differentiation regulator
10778	LUZP4	-0.2408	0.4885	leucine zipper protein 4
10779	LHX5	-0.2408	0.4798	LIM homeobox 5
10780	HNRNPU	-0.2408	0.4199	heterogeneous nuclear ribonucleoprotein U (scaffold attachment factor A)
10781	HBD	-0.2408	0.4164	hemoglobin, delta
10782	DLGAP1	-0.2408	0.4539	discs, large (Drosophila) homolog-associated protein 1
10783	CD6	-0.2408	0.4813	CD6 molecule
10784	CD48	-0.2408	0.3928	CD48 molecule
10785	MCF2L-AS1	-0.2409	0.3474	MCF2L antisense RNA 1 (non-protein coding)
10786	TEKT1	-0.2412	0.48	tektin 1
10787	EPHX4	-0.2412	0.2706	epoxide hydrolase 4
10788	HIST1H2AG	-0.2414	0.3222	histone cluster 1, H2ag
10789	C6orf163	-0.2414	0.3374	chromosome 6 open reading frame 163
10790	BTBD17	-0.2414	0.4503	BTB (POZ) domain containing 17
10791	ZW10	-0.2417	0.4095	ZW10, kinetochore associated, homolog (Drosophila)
10792	TMEM126B	-0.2417	0.4449	transmembrane protein 126B
10793	TEX2	-0.2417	0.3722	testis expressed 2
10794	NRAS	-0.2417	0.4293	neuroblastoma RAS viral (v-ras) oncogene homolog
10795	FAM65B	-0.2417	0.3241	family with sequence similarity 65, member B
10796	DOLPP1	-0.2417	0.3049	dolichyl pyrophosphate phosphatase 1
10797	ATP5C1	-0.2417	0.4322	ATP synthase, H+ transporting, mitochondrial F1 complex, gamma polypeptide 1
10798	ZPBP	-0.2425	0.4499	zona pellucida binding protein
10799	TMSB15B	-0.2425	0.3325	thymosin beta 15B
10800	RPL34-AS1	-0.2425	0.415	RPL34 antisense RNA 1 (non-protein coding)
10801	IDH2	-0.2425	0.357	isocitrate dehydrogenase 2 (NADP+), mitochondrial
10802	GNMT	-0.2425	0.43	glycine N-methyltransferase
10803	FAR1	-0.2425	0.4196	fatty acyl CoA reductase 1
10804	FAM188A	-0.2425	0.4178	family with sequence similarity 188, member A
10805	DTNA	-0.2425	0.3905	dystrobrevin, alpha
10806	DDX19A	-0.2425	0.4104	DEAD (Asp-Glu-Ala-Asp) box polypeptide 19A
10807	DCTN1-AS1	-0.2425	0.3217	DCTN1 antisense RNA 1 (non-protein coding)
10808	DARS2	-0.2425	0.3665	aspartyl-tRNA synthetase 2, mitochondrial
10809	CAMKK2	-0.2425	0.2499	calcium/calmodulin-dependent protein kinase kinase 2, beta
10810	SARS	-0.2433	0.3934	seryl-tRNA synthetase
10811	RNF17	-0.2433	0.4753	ring finger protein 17
10812	ODZ3	-0.2433	0.3138	odz, odd Oz/ten-m homolog 3 (Drosophila)
10813	MS4A4A	-0.2433	0.3587	membrane-spanning 4-domains, subfamily A, member 4A
10814	MFSD12	-0.2433	0.371	major facilitator superfamily domain containing 12
10815	ITCH	-0.2433	0.3757	itchy E3 ubiquitin protein ligase
10816	GLUL	-0.2433	0.3658	glutamate-ammonia ligase
10817	FAM212B	-0.2433	0.4681	family with sequence similarity 212, member B
10818	ELAVL2	-0.2433	0.3333	ELAV (embryonic lethal, abnormal vision, Drosophila)-like 2 (Hu antigen B)
10819	CTC1	-0.2433	0.2899	CTS telomere maintenance complex component 1
10820	PCDH9	-0.2442	0.2677	protocadherin 9
10821	OR10H2	-0.2442	0.4893	olfactory receptor, family 10, subfamily H, member 2
10822	NAPG	-0.2442	0.4102	N-ethylmaleimide-sensitive factor attachment protein, gamma
10823	LENEP	-0.2442	0.4853	lens epithelial protein
10824	KLHL22	-0.2442	0.3358	kelch-like 22 (Drosophila)
10825	HIST1H2AK	-0.2442	0.3212	histone cluster 1, H2ak
10826	FKBP5	-0.2442	0.3457	FK506 binding protein 5
10827	FAM192A	-0.2442	0.367	family with sequence similarity 192, member A
10828	CHRNA2	-0.2442	0.5048	cholinergic receptor, nicotinic, alpha 2 (neuronal)
10829	BTF3	-0.2442	0.4318	basic transcription factor 3
10830	ARID5A	-0.2442	0.3842	AT rich interactive domain 5A (MRF1-like)
10831	ANP32D	-0.2442	0.3753	acidic (leucine-rich) nuclear phosphoprotein 32 family, member D
10832	SLC6A15	-0.245	0.3459	solute carrier family 6 (neutral amino acid transporter), member 15
10833	SEMA6C	-0.245	0.4736	sema domain, transmembrane domain (TM), and cytoplasmic domain, (semaphorin) 6C
10834	RHOF	-0.245	0.3117	ras homolog family member F (in filopodia)

10835	MTMR3	-0.245	0.3091	myotubularin related protein 3
10836	MARK1	-0.245	0.2647	MAP/microtubule affinity-regulating kinase 1
10837	LCP1	-0.245	0.3598	lymphocyte cytosolic protein 1 (L-plastin)
10838	KIAA0895	-0.245	0.3639	KIAA0895
10839	ISCA2	-0.245	0.3977	iron-sulfur cluster assembly 2 homolog (S. cerevisiae)
10840	IGSF6	-0.245	0.3652	immunoglobulin superfamily, member 6
10841	HESX1	-0.245	0.2655	HESX homeobox 1
10842	CLEC12A	-0.245	0.3229	C-type lectin domain family 12, member A
10843	C19orf59	-0.245	0.4195	chromosome 19 open reading frame 59
10844	C17orf28	-0.245	0.4433	chromosome 17 open reading frame 28
10845	ZNF793	-0.2457	0.2945	zinc finger protein 793
10846	PTPRC	-0.2458	0.4165	protein tyrosine phosphatase, receptor type, C
10847	PRTN3	-0.2458	0.4747	proteinase 3
10848	PAN2	-0.2458	0.3786	PAN2 poly(A) specific ribonuclease subunit homolog (S. cerevisiae)
10849	LYN	-0.2458	0.371	v-yes-1 Yamaguchi sarcoma viral related oncogene homolog
10850	KLHL2	-0.2458	0.4148	kelch-like 2, Mayven (Drosophila)
10851	KCND2	-0.2458	0.2809	potassium voltage-gated channel, Shal-related subfamily, member 2
10852	HELZ	-0.2458	0.3995	helicase with zinc finger
10853	FUT8	-0.2458	0.3737	fucosyltransferase 8 (alpha (1,6) fucosyltransferase)
10854	DENR	-0.2458	0.444	density-regulated protein
10855	DDX10	-0.2458	0.4138	DEAD (Asp-Glu-Ala-Asp) box polypeptide 10
10856	BFSP2	-0.2458	0.4483	beaded filament structural protein 2, phakinin
10857	ABCD1	-0.2458	0.4267	ATP-binding cassette, sub-family D (ALD), member 1
10858	SCYL1	-0.2463	0.3349	SCY1-like 1 (S. cerevisiae)
10859	NLN	-0.2463	0.3863	neurolysin (metallopeptidase M3 family)
10860	MAGEE1	-0.2463	0.2938	melanoma antigen family E, 1
10861	GPR114	-0.2463	0.4238	G protein-coupled receptor 114
10862	DMAP1	-0.2463	0.3698	DNA methyltransferase 1 associated protein 1
10863	DACT2	-0.2463	0.4401	dapper, antagonist of beta-catenin, homolog 2 (Xenopus laevis)
10864	C3orf15	-0.2463	0.4118	chromosome 3 open reading frame 15
10865	ART5	-0.2463	0.4953	ADP-ribosyltransferase 5
10866	XYLB	-0.2467	0.2794	xylulokinase homolog (H. influenzae)
10867	XRCC2	-0.2467	0.3223	X-ray repair complementing defective repair in Chinese hamster cells 2
10868	VAPA	-0.2467	0.4149	VAMP (vesicle-associated membrane protein)-associated protein A, 33kDa
10869	TPST1	-0.2467	0.3117	tyrosylprotein sulfotransferase 1
10870	SIGLEC8	-0.2467	0.4792	sialic acid binding Ig-like lectin 8
10871	sept-07	-0.2467	0.4367	septin 7
10872	PTCH2	-0.2467	0.4855	patched 2
10873	NDST4	-0.2467	0.422	N-deacetylase/N-sulfotransferase (heparan glucosaminyl) 4
10874	JAG2	-0.2467	0.3799	jagged 2
10875	ITIH2	-0.2467	0.4416	inter-alpha-trypsin inhibitor heavy chain 2
10876	FASTKD1	-0.2467	0.4209	FAST kinase domains 1
10877	CXCR4	-0.2467	0.3335	chemokine (C-X-C motif) receptor 4
10878	CRYGA	-0.2467	0.4819	crystallin, gamma A
10879	TMEM239	-0.2471	0.5063	transmembrane protein 239
10880	RPL12	-0.2471	0.3928	ribosomal protein L12
10881	MAPK9	-0.2475	0.4123	mitogen-activated protein kinase 9
10882	GZMA	-0.2475	0.3391	granzyme A (granzyme 1, cytotoxic T-lymphocyte-associated serine esterase 3)
10883	EIF3M	-0.2475	0.4476	eukaryotic translation initiation factor 3, subunit M
10884	DNAJC5	-0.2475	0.3071	DnaJ (Hsp40) homolog, subfamily C, member 5
10885	CYSLTR1	-0.2475	0.3173	cysteinyl leukotriene receptor 1
10886	CHD8	-0.2475	0.3439	chromodomain helicase DNA binding protein 8
10887	CDH2	-0.2475	0.2349	cadherin 2, type 1, N-cadherin (neuronal)
10888	ZNF548	-0.2478	0.335	zinc finger protein 548
10889	VPS16	-0.2483	0.3381	vacuolar protein sorting 16 homolog (S. cerevisiae)
10890	TIMM22	-0.2483	0.3066	translocase of inner mitochondrial membrane 22 homolog (yeast)
10891	SLC6A16	-0.2483	0.3069	solute carrier family 6, member 16
10892	PENK	-0.2483	0.3516	proenkephalin
10893	MYCL1	-0.2483	0.4299	v-myc myelocytomatosis viral oncogene homolog 1, lung carcinoma derived (avian)
10894	MRPL9	-0.2483	0.4249	mitochondrial ribosomal protein L9
10895	LPPR2	-0.2483	0.4193	lipid phosphate phosphatase-related protein type 2
10896	LOC392555	-0.2483	0.9999	melanoma antigen family C, 2 pseudogene
10897	ECI2	-0.2483	0.4101	enoyl-CoA delta isomerase 2
10898	DMXL2	-0.2483	0.4134	Dmx-like 2
10899	CD33	-0.2483	0.4208	CD33 molecule
10900	EML5	-0.2486	0.3314	echinoderm microtubule associated protein like 5
10901	ZNF680	-0.2488	0.4121	zinc finger protein 680
10902	ZFAND2A	-0.2488	0.3436	zinc finger, AN1-type domain 2A
10903	ZDHHC20	-0.2488	0.3753	zinc finger, DHHC-type containing 20
10904	TMEM56	-0.2488	0.3228	transmembrane protein 56
10905	VAV2	-0.2492	0.3271	vav 2 guanine nucleotide exchange factor
10906	NKX2-5	-0.2492	0.489	NK2 homeobox 5
10907	MYH10	-0.2492	0.3331	myosin, heavy chain 10, non-muscle
10908	LIN28A	-0.2492	0.3998	lin-28 homolog A (C. elegans)
10909	LDHAL6B	-0.2492	0.4207	lactate dehydrogenase A-like 6B
10910	HNRNPF	-0.2492	0.415	heterogeneous nuclear ribonucleoprotein F
10911	AGAP1	-0.2492	0.3136	ArfGAP with GTPase domain, ankyrin repeat and PH domain 1
10912	ZNF852	-0.25	0.337	zinc finger protein 852
10913	TMEM161B	-0.25	0.4182	transmembrane protein 161B
10914	SARDH	-0.25	0.4773	sarcosine dehydrogenase
10915	NXPH2	-0.25	0.4441	neurexophilin 2
10916	NFKBID	-0.25	0.3889	nuclear factor of kappa light polypeptide gene enhancer in B-cells inhibitor, delta
10917	LOC284100	-0.25	0.4921	tyrosine 3-monooxygenase/tryptophan 5-monooxygenase activation protein, epsilon polypeptide pseudogene
10918	GRIA4	-0.25	0.4163	glutamate receptor, ionotropic, AMPA 4

10919	FNDC4	-0.25	0.4343	fibronectin type III domain containing 4
10920	DCHS1	-0.25	0.3814	dachsous 1 (Drosophila)
10921	C5orf55	-0.25	0.2865	chromosome 5 open reading frame 55
10922	ALS2CR11	-0.25	0.3496	amyotrophic lateral sclerosis 2 (juvenile) chromosome region, candidate 11
10923	POU1F1	-0.2508	0.3756	POU class 1 homeobox 1
10924	LDLRAP1	-0.2508	0.2748	low density lipoprotein receptor adaptor protein 1
10925	KLHDC8A	-0.2508	0.4502	kelch domain containing 8A
10926	KAT6A	-0.2508	0.3687	K(lysine) acetyltransferase 6A
10927	INTS12	-0.2508	0.4158	integrator complex subunit 12
10928	FAM162A	-0.2508	0.4022	family with sequence similarity 162, member A
10929	DTNB	-0.2508	0.3763	dystrobrevin, beta
10930	ADAM2	-0.2508	0.3759	ADAM metallopeptidase domain 2
10931	ACTR8	-0.2508	0.3381	ARP8 actin-related protein 8 homolog (yeast)
10932	ZIK1	-0.2512	0.2349	zinc finger protein interacting with K protein 1 homolog (mouse)
10933	STYX	-0.2512	0.4054	serine/threonine/tyrosine interacting protein
10934	PGLYRP2	-0.2512	0.4564	peptidoglycan recognition protein 2
10935	NTNG1	-0.2512	0.3314	netrin G1
10936	FAM58A	-0.2512	0.3692	family with sequence similarity 58, member A
10937	C6orf123	-0.2514	0.4855	chromosome 6 open reading frame 123
10938	TRPC7	-0.2517	0.4552	transient receptor potential cation channel, subfamily C, member 7
10939	TOP1	-0.2517	0.3896	topoisomerase (DNA) I
10940	OSGEP	-0.2517	0.3641	O-sialoglycoprotein endopeptidase
10941	GATA1	-0.2517	0.492	GATA binding protein 1 (globin transcription factor 1)
10942	EXOC7	-0.2517	0.3283	exocyst complex component 7
10943	CYB5B	-0.2517	0.4162	cytochrome b5 type B (outer mitochondrial membrane)
10944	CLC	-0.2517	0.3521	Charcot-Leyden crystal protein
10945	CHMP1A	-0.2517	0.3534	charged multivesicular body protein 1A
10946	ZNF283	-0.2525	0.389	zinc finger protein 283
10947	PNKP	-0.2525	0.3868	polynucleotide kinase 3'-phosphatase
10948	MEG3	-0.2525	0.4108	maternally expressed 3 (non-protein coding)
10949	IL17D	-0.2525	0.3445	interleukin 17D
10950	GAL	-0.2525	0.339	galanin prepropeptide
10951	FGF14	-0.2525	0.3884	fibroblast growth factor 14
10952	CXXC5	-0.2525	0.2376	CXXC finger protein 5
10953	TTC36	-0.2529	0.4959	tetratricopeptide repeat domain 36
10954	LINC00323	-0.2529	0.9999	long intergenic non-protein coding RNA 323
10955	TUBA4A	-0.2533	0.3345	tubulin, alpha 4a
10956	SCT	-0.2533	0.4557	secretin
10957	RBM5	-0.2533	0.3846	RNA binding motif protein 5
10958	NUP54	-0.2533	0.4425	nucleoporin 54kDa
10959	MYO7A	-0.2533	0.4656	myosin VIIA
10960	MNDA	-0.2533	0.3739	myeloid cell nuclear differentiation antigen
10961	CDK5RAP3	-0.2533	0.3607	CDK5 regulatory subunit associated protein 3
10962	CAMK1D	-0.2533	0.2031	calcium/calmodulin-dependent protein kinase ID
10963	AKR7A2	-0.2533	0.3855	aldo-keto reductase family 7, member A2 (aflatoxin aldehyde reductase)
10964	RXFP1	-0.2537	0.3085	relaxin/insulin-like family peptide receptor 1
10965	RGS8	-0.2537	0.4753	regulator of G-protein signaling 8
10966	MAS1L	-0.2537	0.4875	MAS1 oncogene-like
10967	LOH12CR2	-0.2537	0.274	loss of heterozygosity, 12, chromosomal region 2 (non-protein coding)
10968	LOC148413	-0.2537	0.315	uncharacterized LOC148413
10969	ZNF324	-0.2542	0.3427	zinc finger protein 324
10970	SARS2	-0.2542	0.3542	seryl-tRNA synthetase 2, mitochondrial
10971	PRPF4B	-0.2542	0.4418	PRP4 pre-mRNA processing factor 4 homolog B (yeast)
10972	PDZD7	-0.2542	0.4802	PDZ domain containing 7
10973	MAP4	-0.2542	0.2798	microtubule-associated protein 4
10974	LINC00588	-0.2542	0.3977	long intergenic non-protein coding RNA 588
10975	HAUS7	-0.2542	0.3899	HAUS augmin-like complex, subunit 7
10976	DUT	-0.2542	0.4428	deoxyuridine triphosphatase
10977	ATG4B	-0.2542	0.3065	autophagy related 4B, cysteine peptidase
10978	ARTN	-0.2542	0.4835	artemin
10979	ZNF574	-0.255	0.3428	zinc finger protein 574
10980	TUBE1	-0.255	0.4215	tubulin, epsilon 1
10981	TMC2	-0.255	0.5052	transmembrane channel-like 2
10982	THNSL1	-0.255	0.369	threonine synthase-like 1 (S. cerevisiae)
10983	THAP9	-0.255	0.3925	THAP domain containing 9
10984	SAMD11	-0.255	0.4525	sterile alpha motif domain containing 11
10985	PIK3R2	-0.255	0.3897	phosphoinositide-3-kinase, regulatory subunit 2 (beta)
10986	PDE3B	-0.255	0.3105	phosphodiesterase 3B, cGMP-inhibited
10987	PCNXL2	-0.255	0.283	pecanex-like 2 (Drosophila)
10988	PCDHA5	-0.255	0.4096	protocadherin alpha 5
10989	MYSM1	-0.255	0.4077	Myb-like, SWIRM and MPN domains 1
10990	MCRS1	-0.255	0.3667	microspherule protein 1
10991	MAGEB2	-0.255	0.3727	melanoma antigen family B, 2
10992	KLHDC7B	-0.255	0.4489	kelch domain containing 7B
10993	HDHD2	-0.255	0.4052	haloacid dehalogenase-like hydrolase domain containing 2
10994	GPT2	-0.255	0.2896	glutamic pyruvate transaminase (alanine aminotransferase) 2
10995	CYHR1	-0.255	0.3676	cysteine/histidine-rich 1
10996	CXorf69	-0.255	0.2415	chromosome X open reading frame 69
10997	BRWD3	-0.255	0.3524	bromodomain and WD repeat domain containing 3
10998	APOBR	-0.255	0.4286	apolipoprotein B receptor
10999	APLN	-0.255	0.4616	apelin
11000	SERINC5	-0.2557	0.3449	serine incorporator 5
11001	GANC	-0.2557	0.2487	glucosidase, alpha; neutral C
11002	WDR41	-0.2558	0.4023	WD repeat domain 41

11003	TRIM27	-0.2558	0.3848	tripartite motif containing 27
11004	STK25	-0.2558	0.3724	serine/threonine kinase 25
11005	PTCRA	-0.2558	0.5023	pre T-cell antigen receptor alpha
11006	PSAT1	-0.2558	0.3476	phosphoserine aminotransferase 1
11007	PIK3CG	-0.2558	0.3142	phosphatidylinositol-4,5-bisphosphate 3-kinase, catalytic subunit gamma
11008	HBQ1	-0.2558	0.4738	hemoglobin, theta 1
11009	ELK1	-0.2558	0.3844	ELK1, member of ETS oncogene family
11010	DNM3	-0.2558	0.2544	dynamin 3
11011	CNNM3	-0.2558	0.3084	cyclin M3
11012	CACNA1F	-0.2558	0.4935	calcium channel, voltage-dependent, L type, alpha 1F subunit
11013	ADNP	-0.2558	0.4263	activity-dependent neuroprotector homeobox
11014	ZNF483	-0.2562	0.2907	zinc finger protein 483
11015	TIMD4	-0.2562	0.3503	T-cell immunoglobulin and mucin domain containing 4
11016	RP9	-0.2562	0.3879	retinitis pigmentosa 9 (autosomal dominant)
11017	NDUFB9	-0.2562	0.3819	NADH dehydrogenase (ubiquinone) 1 beta subcomplex, 9, 22kDa
11018	LRRC43	-0.2562	0.4881	leucine rich repeat containing 43
11019	ZNF337	-0.2567	0.3354	zinc finger protein 337
11020	TREML2	-0.2567	0.4622	triggering receptor expressed on myeloid cells-like 2
11021	MRPS2	-0.2567	0.4081	mitochondrial ribosomal protein S2
11022	DAB1	-0.2567	0.4793	disabled homolog 1 (Drosophila)
11023	CD320	-0.2567	0.3834	CD320 molecule
11024	THAP3	-0.2571	0.3722	THAP domain containing, apoptosis associated protein 3
11025	LOC100130452	-0.2571	0.9999	uncharacterized LOC100130452
11026	C1orf220	-0.2571	0.3412	chromosome 1 open reading frame 220
11027	TMEM55B	-0.2575	0.3091	transmembrane protein 55B
11028	SPESP1	-0.2575	0.2132	sperm equatorial segment protein 1
11029	PIPOX	-0.2575	0.4009	pipecolic acid oxidase
11030	NUDT16L1	-0.2575	0.3635	nudix (nucleoside diphosphate linked moiety X)-type motif 16-like 1
11031	ESRRB	-0.2575	0.4781	estrogen-related receptor beta
11032	ARL8A	-0.2575	0.2475	ADP-ribosylation factor-like 8A
11033	WASF3	-0.2583	0.3327	WAS protein family, member 3
11034	VPS35	-0.2583	0.4044	vacuolar protein sorting 35 homolog (S. cerevisiae)
11035	SETD4	-0.2583	0.3324	SET domain containing 4
11036	RTN2	-0.2583	0.3381	reticulon 2
11037	RHAG	-0.2583	0.4748	Rh-associated glycoprotein
11038	NRBF2	-0.2583	0.4269	nuclear receptor binding factor 2
11039	NEMF	-0.2583	0.4249	nuclear export mediator factor
11040	LTB4R	-0.2583	0.3768	leukotriene B4 receptor
11041	HCRTR1	-0.2583	0.4818	hypocretin (orexin) receptor 1
11042	GIN1	-0.2583	0.4283	gypsy retrotransposon integrase 1
11043	FEV	-0.2583	0.4938	FEV (ETS oncogene family)
11044	DBC1	-0.2583	0.3949	deleted in bladder cancer 1
11045	CHRNA4	-0.2583	0.4974	cholinergic receptor, nicotinic, alpha 4 (neuronal)
11046	TTC39C	-0.2587	0.3374	tetratricopeptide repeat domain 39C
11047	OIT3	-0.2587	0.4165	oncoprotein induced transcript 3
11048	KRT27	-0.2587	0.3779	keratin 27
11049	FCAMR	-0.2587	0.4586	Fc receptor, IgA, IgM, high affinity
11050	SH2B3	-0.2592	0.3583	SH2B adaptor protein 3
11051	OGFOD1	-0.2592	0.4074	2-oxoglutarate and iron-dependent oxygenase domain containing 1
11052	LTB	-0.2592	0.4098	lymphotoxin beta (TNF superfamily, member 3)
11053	CSH2	-0.2592	0.4688	chorionic somatomammotropin hormone 2
11054	ATRNL1	-0.2592	0.3178	attractin-like 1
11055	AKAP7	-0.2592	0.3002	A kinase (PRKA) anchor protein 7
11056	ACOT13	-0.2592	0.4039	acyl-CoA thioesterase 13
11057	ZNF660	-0.26	0.282	zinc finger protein 660
11058	UBE3D	-0.26	0.3942	ubiquitin protein ligase E3D
11059	TACO1	-0.26	0.3633	translational activator of mitochondrially encoded cytochrome c oxidase I
11060	RPL28	-0.26	0.3533	ribosomal protein L28
11061	RAD21L1	-0.26	0.3794	RAD21-like 1 (S. pombe)
11062	NR2C2	-0.26	0.389	nuclear receptor subfamily 2, group C, member 2
11063	LMO2	-0.26	0.3306	LIM domain only 2 (rhombotin-like 1)
11064	LINC00341	-0.26	0.3359	long intergenic non-protein coding RNA 341
11065	GKN2	-0.26	0.4173	gastrokine 2
11066	GET4	-0.26	0.3505	golgi to ER traffic protein 4 homolog (S. cerevisiae)
11067	FAM170A	-0.26	0.4769	family with sequence similarity 170, member A
11068	EVX1	-0.26	0.4905	even-skipped homeobox 1
11069	CYBB	-0.26	0.3559	cytochrome b-245, beta polypeptide
11070	ACADM	-0.26	0.4332	acyl-CoA dehydrogenase, C-4 to C-12 straight chain
11071	ZNF146	-0.2608	0.4321	zinc finger protein 146
11072	SCN2A	-0.2608	0.3599	sodium channel, voltage-gated, type II, alpha subunit
11073	RNF125	-0.2608	0.2984	ring finger protein 125, E3 ubiquitin protein ligase
11074	POMP	-0.2608	0.4233	proteasome maturation protein
11075	HAP1	-0.2608	0.4875	huntingtin-associated protein 1
11076	ANK1	-0.2608	0.4404	ankyrin 1, erythrocytic
11077	VPS37D	-0.2612	0.4699	vacuolar protein sorting 37 homolog D (S. cerevisiae)
11078	TPGS1	-0.2612	0.3426	tubulin polyglutamylase complex subunit 1
11079	THAP5	-0.2612	0.4253	THAP domain containing 5
11080	PDCD7	-0.2612	0.4096	programmed cell death 7
11081	HFM1	-0.2612	0.2634	HFM1, ATP-dependent DNA helicase homolog (S. cerevisiae)
11082	BLOC1S4	-0.2612	0.3969	biogenesis of lysosomal organelles complex-1, subunit 4, cappuccino
11083	PCDHB3	-0.2614	0.209	protocadherin beta 3
11084	LOC283867	-0.2614	0.4369	uncharacterized LOC283867
11085	C3orf43	-0.2614	0.4993	chromosome 3 open reading frame 43
11086	SNRK	-0.2617	0.4042	SNF related kinase

11087	GZMH	-0.2617	0.3989	granzyme H (cathepsin G-like 2, protein h-CCPX)
11088	GNLY	-0.2617	0.3762	granulysin
11089	C11orf20	-0.2617	0.47	chromosome 11 open reading frame 20
11090	ARFRP1	-0.2617	0.3986	ADP-ribosylation factor related protein 1
11091	ZNF580	-0.2625	0.3608	zinc finger protein 580
11092	TH1L	-0.2625	0.3922	TH1-like (Drosophila)
11093	OBP2B	-0.2625	0.4143	odorant binding protein 2B
11094	MGC2752	-0.2625	0.2722	CENPB DNA-binding domains containing 1 pseudogene
11095	LGI1	-0.2625	0.3296	leucine-rich, glioma inactivated 1
11096	FPR2	-0.2625	0.3952	formyl peptide receptor 2
11097	FGF19	-0.2625	0.4566	fibroblast growth factor 19
11098	FAM71F1	-0.2625	0.469	family with sequence similarity 71, member F1
11099	DNAJC11	-0.2625	0.3291	DnaJ (Hsp40) homolog, subfamily C, member 11
11100	CIB2	-0.2625	0.3592	calcium and integrin binding family member 2
11101	C1orf170	-0.2625	0.5023	chromosome 1 open reading frame 170
11102	BCAT2	-0.2625	0.3305	branched chain amino-acid transaminase 2, mitochondrial
11103	OSTM1-AS1	-0.2629	0.9999	OSTM1 antisense RNA 1 (non-protein coding)
11104	WDR33	-0.2633	0.3733	WD repeat domain 33
11105	UNG	-0.2633	0.4187	uracil-DNA glycosylase
11106	TEC	-0.2633	0.2601	tec protein tyrosine kinase
11107	TCF15	-0.2633	0.4561	transcription factor 15 (basic helix-loop-helix)
11108	SSTR1	-0.2633	0.39	somatostatin receptor 1
11109	SNX27	-0.2633	0.2607	sorting nexin family member 27
11110	PNMT	-0.2633	0.4478	phenylethanolamine N-methyltransferase
11111	GJB1	-0.2633	0.4468	gap junction protein, beta 1, 32kDa
11112	CSTF2	-0.2633	0.3677	cleavage stimulation factor, 3' pre-RNA, subunit 2, 64kDa
11113	B4GALT2	-0.2633	0.3565	UDP-Gal:betaGlcNAc beta 1,4- galactosyltransferase, polypeptide 2
11114	AQP8	-0.2633	0.4803	aquaporin 8
11115	AAR2	-0.2633	0.3227	AAR2 splicing factor homolog (S. cerevisiae)
11116	METTL12	-0.2637	0.2811	methyltransferase like 12
11117	CEACAM19	-0.2637	0.488	carcinoembryonic antigen-related cell adhesion molecule 19
11118	CDKN2AIPNL	-0.2637	0.3458	CDKN2A interacting protein N-terminal like
11119	HOMER1	-0.2642	0.3788	homer homolog 1 (Drosophila)
11120	HDAC4	-0.2642	0.3251	histone deacetylase 4
11121	GCFC2	-0.2642	0.4112	GC-rich sequence DNA-binding factor 2
11122	FOXN2	-0.2642	0.3353	forkhead box N2
11123	SNORD114-3	-0.2643	0.1982	small nucleolar RNA, C/D box 114-3
11124	C14orf182	-0.2643	0.29	chromosome 14 open reading frame 182
11125	MEF2BNB	-0.2645	0.28	MEF2B neighbor
11126	UBE2D1	-0.265	0.4195	ubiquitin-conjugating enzyme E2D 1
11127	TRPV2	-0.265	0.3344	transient receptor potential cation channel, subfamily V, member 2
11128	PSTPIP1	-0.265	0.4178	proline-serine-threonine phosphatase interacting protein 1
11129	PKNOX1	-0.265	0.2808	PBX/knotted 1 homeobox 1
11130	NHLH1	-0.265	0.47	nescient helix loop helix 1
11131	MTSS1L	-0.265	0.3963	metastasis suppressor 1-like
11132	LIPT1	-0.265	0.417	lipoyltransferase 1
11133	GPR88	-0.265	0.3716	G protein-coupled receptor 88
11134	FBXO6	-0.265	0.3306	F-box protein 6
11135	DAPK3	-0.265	0.324	death-associated protein kinase 3
11136	AXIN1	-0.265	0.3568	axin 1
11137	C15orf63	-0.2657	0.407	chromosome 15 open reading frame 63
11138	UBFD1	-0.2658	0.3802	ubiquitin family domain containing 1
11139	TUSC3	-0.2658	0.3093	tumor suppressor candidate 3
11140	TSGA10	-0.2658	0.2594	testis specific, 10
11141	NOL8	-0.2658	0.4162	nucleolar protein 8
11142	MRP63	-0.2658	0.3981	mitochondrial ribosomal protein 63
11143	KIN	-0.2658	0.4296	KIN, antigenic determinant of recA protein homolog (mouse)
11144	KCNA5	-0.2658	0.4177	potassium voltage-gated channel, shaker-related subfamily, member 5
11145	DEK	-0.2658	0.4473	DEK oncogene
11146	CEP164	-0.2658	0.29	centrosomal protein 164kDa
11147	CDC27	-0.2658	0.4247	cell division cycle 27 homolog (S. cerevisiae)
11148	ANKRD12	-0.2658	0.414	ankyrin repeat domain 12
11149	VSTM2L	-0.2663	0.4397	V-set and transmembrane domain containing 2 like
11150	NKAPL	-0.2663	0.3396	NFKB activating protein-like
11151	IL17C	-0.2663	0.506	interleukin 17C
11152	C7orf33	-0.2663	0.4494	chromosome 7 open reading frame 33
11153	C3orf35	-0.2663	0.3505	chromosome 3 open reading frame 35
11154	ANKUB1	-0.2663	0.4423	ankyrin repeat and ubiquitin domain containing 1
11155	TBR1	-0.2667	0.4356	T-box, brain, 1
11156	SLC7A4	-0.2667	0.4623	solute carrier family 7 (orphan transporter), member 4
11157	SFTPB	-0.2667	0.4911	surfactant protein B
11158	RANBP6	-0.2667	0.4209	RAN binding protein 6
11159	PABPC3	-0.2667	0.4096	poly(A) binding protein, cytoplasmic 3
11160	NOL9	-0.2667	0.357	nucleolar protein 9
11161	MIER2	-0.2667	0.386	mesoderm induction early response 1, family member 2
11162	LILRB3	-0.2667	0.458	leukocyte immunoglobulin-like receptor, subfamily B (with TM and ITIM domains), member 3
11163	DLEC1	-0.2667	0.4755	deleted in lung and esophageal cancer 1
11164	DDHD2	-0.2671	0.3781	DDHD domain containing 2
11165	ZNF2	-0.2675	0.2975	zinc finger protein 2
11166	WHSC2	-0.2675	0.3707	Wolf-Hirschhorn syndrome candidate 2
11167	TSPEAR	-0.2675	0.4979	thrombospondin-type laminin G domain and EAR repeats
11168	SLCO4A1	-0.2675	0.285	solute carrier organic anion transporter family, member 4A1
11169	SLC25A36	-0.2675	0.4039	solute carrier family 25 (pyrimidine nucleotide carrier), member 36
11170	SLC1A6	-0.2675	0.4639	solute carrier family 1 (high affinity aspartate/glutamate transporter), member 6

11171	RGS19	-0.2675	0.3603	regulator of G-protein signaling 19
11172	NLRP13	-0.2675	0.4856	NLR family, pyrin domain containing 13
11173	MRPL11	-0.2675	0.4174	mitochondrial ribosomal protein L11
11174	MAGEC2	-0.2675	0.4024	melanoma antigen family C, 2
11175	LOC90834	-0.2675	0.2947	uncharacterized protein BC001742
11176	L1CAM	-0.2675	0.4052	L1 cell adhesion molecule
11177	FOXH1	-0.2675	0.4821	forkhead box H1
11178	EXOG	-0.2675	0.2825	endo/exonuclease (5'-3'), endonuclease G-like
11179	CYP21A2	-0.2675	0.4487	cytochrome P450, family 21, subfamily A, polypeptide 2
11180	CKLF	-0.2675	0.3966	chemokine-like factor
11181	B3GNT7	-0.2675	0.4113	UDP-GlcNAc:betaGal beta-1,3-N-acetylglucosaminyltransferase 7
11182	ATP6V1G2	-0.2675	0.3236	ATPase, H+ transporting, lysosomal 13kDa, V1 subunit G2
11183	ZNF430	-0.2683	0.395	zinc finger protein 430
11184	URI1	-0.2683	0.4241	URI1, prefoldin-like chaperone
11185	PFKFB1	-0.2683	0.4322	6-phosphofructo-2-kinase/fructose-2,6-biphosphatase 1
11186	NARFL	-0.2683	0.3685	nuclear prelamin A recognition factor-like
11187	KLF1	-0.2683	0.4717	Kruppel-like factor 1 (erythroid)
11188	HAUS5	-0.2683	0.3398	HAUS augmin-like complex, subunit 5
11189	DCP1A	-0.2683	0.342	DCP1 decapping enzyme homolog A (S. cerevisiae)
11190	C11orf80	-0.2683	0.2156	chromosome 11 open reading frame 80
11191	BNIP3L	-0.2683	0.3724	BCL2/adenovirus E1B 19kDa interacting protein 3-like
11192	ZYG11B	-0.2688	0.392	zyg-11 homolog B (C. elegans)
11193	SOGA1	-0.2688	0.226	suppressor of glucose, autophagy associated 1
11194	SEPSECS	-0.2688	0.3984	Sep (O-phosphoserine) tRNA:Sec (selenocysteine) tRNA synthase
11195	PCDHGC5	-0.2688	0.4831	protocadherin gamma subfamily C, 5
11196	GRIPAP1	-0.2688	0.2875	GRIP1 associated protein 1
11197	AP5B1	-0.2688	0.2762	adaptor-related protein complex 5, beta 1 subunit
11198	KDM4B	-0.2691	0.3283	lysine (K)-specific demethylase 4B
11199	UTP3	-0.2692	0.4255	UTP3, small subunit (SSU) processome component, homolog (S. cerevisiae)
11200	RERE	-0.2692	0.2794	arginine-glutamic acid dipeptide (RE) repeats
11201	PTPN1	-0.2692	0.3112	protein tyrosine phosphatase, non-receptor type 1
11202	PTGES2	-0.2692	0.411	prostaglandin E synthase 2
11203	PCDHB8	-0.2692	0.1837	protocadherin beta 8
11204	NCBP1	-0.2692	0.4198	nuclear cap binding protein subunit 1, 80kDa
11205	KRR1	-0.2692	0.4323	KRR1, small subunit (SSU) processome component, homolog (yeast)
11206	DKC1	-0.2692	0.4338	dyskeratosis congenita 1, dyskerin
11207	CUX2	-0.2692	0.3453	cut-like homeobox 2
11208	ADRA1D	-0.2692	0.4786	adrenoceptor alpha 1D
11209	WFDC10A	-0.27	0.4252	WAP four-disulfide core domain 10A
11210	TRMT11	-0.27	0.4268	tRNA methyltransferase 11 homolog (S. cerevisiae)
11211	SAYSD1	-0.27	0.3521	SAYSVFN motif domain containing 1
11212	RPS21	-0.27	0.4102	ribosomal protein S21
11213	RASSF3	-0.27	0.3033	Ras association (RalGDS/AF-6) domain family member 3
11214	PNMA1	-0.27	0.3935	paraneoplastic Ma antigen 1
11215	MRAP	-0.27	0.4954	melanocortin 2 receptor accessory protein
11216	MDM1	-0.27	0.4196	Mdm1 nuclear protein homolog (mouse)
11217	MAFG-AS1	-0.27	0.4673	MAFG antisense RNA 1 (non-protein coding)
11218	LRRC73	-0.27	0.4327	leucine rich repeat containing 73
11219	LOC100134259	-0.27	0.4273	uncharacterized LOC100134259
11220	KRTAP11-1	-0.27	0.4893	keratin associated protein 11-1
11221	KCNK17	-0.27	0.4334	potassium channel, subfamily K, member 17
11222	HCRT	-0.27	0.49	hypocretin (orexin) neuropeptide precursor
11223	DEDD2	-0.27	0.3019	death effector domain containing 2
11224	CSHL1	-0.27	0.4896	chorionic somatomammotropin hormone-like 1
11225	BEX4	-0.27	0.3754	brain expressed, X-linked 4
11226	ADAM32	-0.27	0.2447	ADAM metallopeptidase domain 32
11227	UBTF	-0.2708	0.3398	upstream binding transcription factor, RNA polymerase I
11228	RASL11B	-0.2708	0.2763	RAS-like, family 11, member B
11229	PURG	-0.2708	0.4079	purine-rich element binding protein G
11230	IDH3A	-0.2708	0.4173	isocitrate dehydrogenase 3 (NAD+) alpha
11231	ARHGAP25	-0.2708	0.3276	Rho GTPase activating protein 25
11232	ADD1	-0.2708	0.3119	adducin 1 (alpha)
11233	SWT1	-0.2713	0.2895	SWT1 RNA endoribonuclease homolog (S. cerevisiae)
11234	RNF215	-0.2713	0.4452	ring finger protein 215
11235	PHF12	-0.2713	0.2627	PHD finger protein 12
11236	GAS2L2	-0.2713	0.5056	growth arrest-specific 2 like 2
11237	ANKS3	-0.2713	0.3984	ankyrin repeat and sterile alpha motif domain containing 3
11238	FKSG2	-0.2714	0.2819	tumor protein, translationally-controlled 1 pseudogene
11239	ROGDI	-0.2717	0.3064	rogdi homolog (Drosophila)
11240	PTBP1	-0.2717	0.3981	polypyrimidine tract binding protein 1
11241	PPP2R4	-0.2717	0.3218	protein phosphatase 2A activator, regulatory subunit 4
11242	HIST1H1A	-0.2717	0.3988	histone cluster 1, H1a
11243	HHLA3	-0.2717	0.2564	HERV-H LTR-associating 3
11244	GPR135	-0.2717	0.4726	G protein-coupled receptor 135
11245	BTBD2	-0.2717	0.3485	BTB (POZ) domain containing 2
11246	ZNF280A	-0.2725	0.4117	zinc finger protein 280A
11247	ZNF226	-0.2725	0.3987	zinc finger protein 226
11248	SPRYD4	-0.2725	0.2999	SPRY domain containing 4
11249	SLC26A10	-0.2725	0.4155	solute carrier family 26, member 10
11250	NSMCE2	-0.2725	0.378	non-SMC element 2, MMS21 homolog (S. cerevisiae)
11251	MIB2	-0.2725	0.4077	mindbomb E3 ubiquitin protein ligase 2
11252	LRP4	-0.2725	0.2275	low density lipoprotein receptor-related protein 4
11253	LOC113230	-0.2725	0.4435	uncharacterized LOC113230
11254	KIAA0895L	-0.2725	0.2627	KIAA0895-like

11255	JAK3	-0.2725	0.4268	Janus kinase 3
11256	FH	-0.2725	0.4195	fumarate hydratase
11257	FBXO39	-0.2725	0.4378	F-box protein 39
11258	ENTPD2	-0.2725	0.4802	ectonucleoside triphosphate diphosphohydrolase 2
11259	DIS3L2	-0.2725	0.3092	DIS3 mitotic control homolog (S. cerevisiae)-like 2
11260	CTAG2	-0.2725	0.4674	cancer/testis antigen 2
11261	CELSR2	-0.2725	0.2678	cadherin, EGF LAG seven-pass G-type receptor 2 (flamingo homolog, Drosophila)
11262	BRCA2	-0.2725	0.3952	breast cancer 2, early onset
11263	TEX13A	-0.2733	0.489	testis expressed 13A
11264	POLR2J	-0.2733	0.3469	polymerase (RNA) II (DNA directed) polypeptide J, 13.3kDa
11265	NFATC2IP	-0.2733	0.3738	nuclear factor of activated T-cells, cytoplasmic, calcineurin-dependent 2 interacting protein
11266	NEIL3	-0.2733	0.3861	nei endonuclease VIII-like 3 (E. coli)
11267	MTDH	-0.2733	0.4254	metadherin
11268	KCNIP2	-0.2733	0.4398	Kv channel interacting protein 2
11269	IFT122	-0.2733	0.259	intraflagellar transport 122 homolog (Chlamydomonas)
11270	GSTZ1	-0.2733	0.3115	glutathione S-transferase zeta 1
11271	ZNF781	-0.2738	0.2778	zinc finger protein 781
11272	NDUFB10	-0.2738	0.3722	NADH dehydrogenase (ubiquinone) 1 beta subcomplex, 10, 22kDa
11273	DHDH	-0.2738	0.4378	dihydrodiol dehydrogenase (dimeric)
11274	C3orf49	-0.2738	0.4413	chromosome 3 open reading frame 49
11275	TRPC4AP	-0.2742	0.2802	transient receptor potential cation channel, subfamily C, member 4 associated protein
11276	SIRT4	-0.2742	0.2666	sirtuin 4
11277	RXFP3	-0.2742	0.4858	relaxin/insulin-like family peptide receptor 3
11278	PMPCB	-0.2742	0.4192	peptidase (mitochondrial processing) beta
11279	PAX1	-0.2742	0.4863	paired box 1
11280	NFKBIL1	-0.2742	0.4002	nuclear factor of kappa light polypeptide gene enhancer in B-cells inhibitor-like 1
11281	GPR52	-0.2742	0.4392	G protein-coupled receptor 52
11282	EIF2C3	-0.2742	0.3683	eukaryotic translation initiation factor 2C, 3
11283	CACNG4	-0.2742	0.4693	calcium channel, voltage-dependent, gamma subunit 4
11284	INO80B	-0.2743	0.3434	INO80 complex subunit B
11285	TEX29	-0.275	0.4657	testis expressed 29
11286	SPA17	-0.275	0.3479	sperm autoantigenic protein 17
11287	PPP1R32	-0.275	0.4412	protein phosphatase 1, regulatory subunit 32
11288	OPCML	-0.275	0.4211	opioid binding protein/cell adhesion molecule-like
11289	MAST2	-0.275	0.3121	microtubule associated serine/threonine kinase 2
11290	LOC643037	-0.275	0.2313	uncharacterized LOC643037
11291	KIAA0319	-0.275	0.3592	KIAA0319
11292	HK3	-0.275	0.4401	hexokinase 3 (white cell)
11293	GZMM	-0.275	0.4654	granzyme M (lymphocyte met-ase 1)
11294	FAM109A	-0.275	0.4516	family with sequence similarity 109, member A
11295	DDX18	-0.275	0.4352	DEAD (Asp-Glu-Ala-Asp) box polypeptide 18
11296	GRIK1-AS1	-0.2757	0.4056	GRIK1 antisense RNA 1 (non-protein coding)
11297	ZIC1	-0.2758	0.1986	Zic family member 1
11298	TUBGCP4	-0.2758	0.3858	tubulin, gamma complex associated protein 4
11299	ROCK1	-0.2758	0.3839	Rho-associated, coiled-coil containing protein kinase 1
11300	PDCD1	-0.2758	0.4776	programmed cell death 1
11301	LAT	-0.2758	0.3611	linker for activation of T cells
11302	HSPBAP1	-0.2758	0.3792	HSPB (heat shock 27kDa) associated protein 1
11303	DLK1	-0.2758	0.3306	delta-like 1 homolog (Drosophila)
11304	CDK5RAP1	-0.2758	0.3564	CDK5 regulatory subunit associated protein 1
11305	RAB12	-0.2763	0.2875	RAB12, member RAS oncogene family
11306	OSTCP1	-0.2763	0.3096	oligosaccharyltransferase complex subunit pseudogene 1
11307	DYM	-0.2763	0.3811	dymeclin
11308	DKFZP434I0714	-0.2763	0.3764	uncharacterized protein DKFZP434I0714
11309	STK17A	-0.2767	0.373	serine/threonine kinase 17a
11310	SNHG3	-0.2767	0.9996	small nucleolar RNA host gene 3 (non-protein coding)
11311	SIPA1	-0.2767	0.3477	signal-induced proliferation-associated 1
11312	RAP1A	-0.2767	0.4007	RAP1A, member of RAS oncogene family
11313	NUP205	-0.2767	0.4226	nucleoporin 205kDa
11314	ITGAM	-0.2767	0.3521	integrin, alpha M (complement component 3 receptor 3 subunit)
11315	GDPD5	-0.2767	0.3567	glycerophosphodiester phosphodiesterase domain containing 5
11316	DENND2A	-0.2767	0.391	DENN/MADD domain containing 2A
11317	CWF19L1	-0.2767	0.2863	CWF19-like 1, cell cycle control (S. pombe)
11318	CLIP3	-0.2767	0.4081	CAP-GLY domain containing linker protein 3
11319	CDO1	-0.2767	0.2498	cysteine dioxygenase, type I
11320	CAPRIN2	-0.2767	0.3544	caprin family member 2
11321	ATP6V0E2	-0.2767	0.303	ATPase, H+ transporting V0 subunit e2
11322	ARRB1	-0.2767	0.1882	arrestin, beta 1
11323	LINC00319	-0.2771	0.4891	long intergenic non-protein coding RNA 319
11324	KIR3DL3	-0.2771	0.4676	killer cell immunoglobulin-like receptor, three domains, long cytoplasmic tail, 3
11325	FNTB	-0.2771	0.2174	farnesyltransferase, CAAX box, beta
11326	TSPAN17	-0.2775	0.2863	tetraspanin 17
11327	TEX12	-0.2775	0.168	testis expressed 12
11328	SSTR3	-0.2775	0.5005	somatostatin receptor 3
11329	SLC30A2	-0.2775	0.4964	solute carrier family 30 (zinc transporter), member 2
11330	RAB26	-0.2775	0.4254	RAB26, member RAS oncogene family
11331	NDUFB8	-0.2775	0.3786	NADH dehydrogenase (ubiquinone) 1 beta subcomplex, 8, 19kDa
11332	MREG	-0.2775	0.3723	melanoregulin
11333	IDH3B	-0.2775	0.3819	isocitrate dehydrogenase 3 (NAD+) beta
11334	HOXC9	-0.2775	0.192	homeobox C9
11335	HMGCLL1	-0.2775	0.3644	3-hydroxymethyl-3-methylglutaryl-CoA lyase-like 1
11336	GPAM	-0.2775	0.387	glycerol-3-phosphate acyltransferase, mitochondrial
11337	FCRLB	-0.2775	0.2783	Fc receptor-like B
11338	FANCC	-0.2775	0.2862	Fanconi anemia, complementation group C

11339	DDI1	-0.2775	0.4229	DNA-damage inducible 1 homolog 1 (S. cerevisiae)
11340	C12orf52	-0.2775	0.3489	chromosome 12 open reading frame 52
11341	AGPAT1	-0.2775	0.2846	1-acylglycerol-3-phosphate O-acyltransferase 1 (lysophosphatidic acid acyltransferase, alpha)
11342	ZNF195	-0.2783	0.4101	zinc finger protein 195
11343	USP2	-0.2783	0.4099	ubiquitin specific peptidase 2
11344	GLRA2	-0.2783	0.4545	glycine receptor, alpha 2
11345	FSCN2	-0.2783	0.4279	fascin homolog 2, actin-bundling protein, retinal (Strongylocentrotus purpuratus)
11346	FPGS	-0.2783	0.3362	folylpolyglutamate synthase
11347	COX4I1	-0.2783	0.3814	cytochrome c oxidase subunit IV isoform 1
11348	CLIP2	-0.2783	0.2927	CAP-GLY domain containing linker protein 2
11349	CDC23	-0.2783	0.4261	cell division cycle 23 homolog (S. cerevisiae)
11350	APEX2	-0.2783	0.3183	APEX nuclease (apurinic/apyrimidinic endonuclease) 2
11351	TMEM207	-0.2786	0.4905	transmembrane protein 207
11352	OR2K2	-0.2786	0.4698	olfactory receptor, family 2, subfamily K, member 2
11353	LOC645188	-0.2786	0.4254	uncharacterized LOC645188
11354	LINC00410	-0.2786	0.9999	long intergenic non-protein coding RNA 410
11355	GNAS-AS1	-0.2786	0.9999	GNAS antisense RNA 1 (non-protein coding)
11356	C12orf55	-0.2786	0.2007	chromosome 12 open reading frame 55
11357	ZNF876P	-0.2788	0.1892	zinc finger protein 876, pseudogene
11358	ZMYND12	-0.2788	0.2506	zinc finger, MYND-type containing 12
11359	RSPH9	-0.2788	0.3819	radial spoke head 9 homolog (Chlamydomonas)
11360	LOC100131138	-0.2788	0.4195	uncharacterized LOC100131138
11361	CASC2	-0.2788	0.3253	cancer susceptibility candidate 2 (non-protein coding)
11362	C3P1	-0.2788	0.5009	complement component 3 precursor pseudogene
11363	SLC7A1	-0.2792	0.2854	solute carrier family 7 (cationic amino acid transporter, y+ system), member 1
11364	PTPRCAP	-0.2792	0.4105	protein tyrosine phosphatase, receptor type, C-associated protein
11365	OR2H2	-0.2792	0.4292	olfactory receptor, family 2, subfamily H, member 2
11366	LRMP	-0.2792	0.2975	lymphoid-restricted membrane protein
11367	KIAA1107	-0.2792	0.3326	KIAA1107
11368	ALX4	-0.2792	0.4661	ALX homeobox 4
11369	ZNF207	-0.28	0.4127	zinc finger protein 207
11370	RBBP8	-0.28	0.4184	retinoblastoma binding protein 8
11371	PRSS30P	-0.28	0.4722	protease, serine, 30 homolog (mouse), pseudogene
11372	PABPC4	-0.28	0.387	poly(A) binding protein, cytoplasmic 4 (inducible form)
11373	MTSS1	-0.28	0.1962	metastasis suppressor 1
11374	MCMDC2	-0.28	0.2332	minichromosome maintenance domain containing 2
11375	KCNV2	-0.28	0.4741	potassium channel, subfamily V, member 2
11376	HCG18	-0.28	0.3393	HLA complex group 18 (non-protein coding)
11377	FUT5	-0.28	0.4642	fucosyltransferase 5 (alpha (1,3) fucosyltransferase)
11378	DCTN6	-0.28	0.4131	dynactin 6
11379	CD247	-0.28	0.3512	CD247 molecule
11380	CCDC153	-0.28	0.3914	coiled-coil domain containing 153
11381	C14orf180	-0.28	0.5092	chromosome 14 open reading frame 180
11382	ASB3	-0.28	0.4	ankyrin repeat and SOCS box containing 3
11383	ARHGEF6	-0.28	0.3427	Rac/Cdc42 guanine nucleotide exchange factor (GEF) 6
11384	SORCS3	-0.2808	0.3853	sortilin-related VPS10 domain containing receptor 3
11385	SLITRK3	-0.2808	0.3767	SLIT and NTRK-like family, member 3
11386	SLC41A3	-0.2808	0.2042	solute carrier family 41, member 3
11387	RNF114	-0.2808	0.4014	ring finger protein 114
11388	NIP7	-0.2808	0.42	nuclear import 7 homolog (S. cerevisiae)
11389	MPP1	-0.2808	0.2314	membrane protein, palmitoylated 1, 55kDa
11390	MLF2	-0.2808	0.3302	myeloid leukemia factor 2
11391	MAT1A	-0.2808	0.4605	methionine adenosyltransferase I, alpha
11392	GTF2F2	-0.2808	0.3948	general transcription factor IIF, polypeptide 2, 30kDa
11393	GNG13	-0.2808	0.4745	guanine nucleotide binding protein (G protein), gamma 13
11394	DNAJC28	-0.2808	0.1803	DnaJ (Hsp40) homolog, subfamily C, member 28
11395	CLDN18	-0.2808	0.4719	claudin 18
11396	WDR27	-0.2813	0.3324	WD repeat domain 27
11397	PLEKHG1	-0.2813	0.2959	pleckstrin homology domain containing, family G (with RhoGef domain) member 1
11398	MRPS26	-0.2813	0.3848	mitochondrial ribosomal protein S26
11399	FBXO15	-0.2813	0.2787	F-box protein 15
11400	APOL4	-0.2813	0.2765	apolipoprotein L, 4
11401	TNFRSF4	-0.2817	0.4407	tumor necrosis factor receptor superfamily, member 4
11402	TAL1	-0.2817	0.447	T-cell acute lymphocytic leukemia 1
11403	RBMXL2	-0.2817	0.4357	RNA binding motif protein, X-linked-like 2
11404	NDUFAF3	-0.2817	0.3662	NADH dehydrogenase (ubiquinone) complex I, assembly factor 3
11405	NCR3	-0.2817	0.4547	natural cytotoxicity triggering receptor 3
11406	HIST1H2BH	-0.2817	0.2477	histone cluster 1, H2bh
11407	GNPTAB	-0.2817	0.3893	N-acetylglucosamine-1-phosphate transferase, alpha and beta subunits
11408	CRYBA4	-0.2817	0.4554	crystallin, beta A4
11409	CPSF6	-0.2817	0.4249	cleavage and polyadenylation specific factor 6, 68kDa
11410	CD79B	-0.2817	0.3828	CD79b molecule, immunoglobulin-associated beta
11411	CD69	-0.2817	0.3379	CD69 molecule
11412	BTN2A2	-0.2817	0.2914	butyrophilin, subfamily 2, member A2
11413	STAU2	-0.2825	0.3887	staufen, RNA binding protein, homolog 2 (Drosophila)
11414	SRY	-0.2825	0.4275	sex determining region Y
11415	PIGA	-0.2825	0.4039	phosphatidylinositol glycan anchor biosynthesis, class A
11416	NMT2	-0.2825	0.3802	N-myristoyltransferase 2
11417	NFYB	-0.2825	0.4245	nuclear transcription factor Y, beta
11418	NEU2	-0.2825	0.4721	sialidase 2 (cytosolic sialidase)
11419	MNX1	-0.2825	0.4055	motor neuron and pancreas homeobox 1
11420	MGC27382	-0.2825	0.2455	uncharacterized MGC27382
11421	ITGAX	-0.2825	0.4027	integrin, alpha X (complement component 3 receptor 4 subunit)
11422	FCRL5	-0.2825	0.3945	Fc receptor-like 5

11423	FAM166B	-0.2825	0.4008	family with sequence similarity 166, member B
11424	ERP27	-0.2825	0.2055	endoplasmic reticulum protein 27
11425	COX6B1	-0.2825	0.3715	cytochrome c oxidase subunit VIb polypeptide 1 (ubiquitous)
11426	C6orf225	-0.2825	0.2749	chromosome 6 open reading frame 225
11427	TAF4B	-0.2833	0.3184	TAF4b RNA polymerase II, TATA box binding protein (TBP)-associated factor, 105kDa
11428	CLEC4A	-0.2833	0.3363	C-type lectin domain family 4, member A
11429	CDK17	-0.2833	0.4008	cyclin-dependent kinase 17
11430	CD3G	-0.2833	0.3297	CD3g molecule, gamma (CD3-TCR complex)
11431	CACNA1S	-0.2833	0.4716	calcium channel, voltage-dependent, L type, alpha 1S subunit
11432	BMS1	-0.2833	0.3992	BMS1 homolog, ribosome assembly protein (yeast)
11433	ATP8B3	-0.2833	0.4407	ATPase, aminophospholipid transporter, class I, type 8B, member 3
11434	ZNF766	-0.2837	0.3761	zinc finger protein 766
11435	SESN2	-0.2837	0.2462	sestrin 2
11436	DCAF13	-0.2837	0.4333	DDB1 and CUL4 associated factor 13
11437	SUPT16H	-0.2842	0.3947	suppressor of Ty 16 homolog (S. cerevisiae)
11438	POLR2H	-0.2842	0.3827	polymerase (RNA) II (DNA directed) polypeptide H
11439	LRRTM4	-0.2842	0.2437	leucine rich repeat transmembrane neuronal 4
11440	FSCN1	-0.2842	0.2753	fascin homolog 1, actin-bundling protein (Strongylocentrotus purpuratus)
11441	C11orf41	-0.2842	0.3273	chromosome 11 open reading frame 41
11442	WWTR1-AS1	-0.2843	0.9999	WWTR1 antisense RNA 1 (non-protein coding)
11443	STL	-0.2843	0.9996	six-twelve leukemia
11444	STUB1	-0.285	0.3572	STIP1 homology and U-box containing protein 1, E3 ubiquitin protein ligase
11445	SEMA5B	-0.285	0.4349	sema domain, seven thrombospondin repeats (type 1 and type 1-like), transmembrane domain (TM) and short cytoplasmic domain, (semaphorin) 5
11446	MPC2	-0.285	0.403	mitochondrial pyruvate carrier 2
11447	LMO1	-0.285	0.4176	LIM domain only 1 (rhombotin 1)
11448	HELB	-0.285	0.2579	helicase (DNA) B
11449	CHN1	-0.285	0.2768	chimerin (chimaerin) 1
11450	C9orf50	-0.285	0.4865	chromosome 9 open reading frame 50
11451	ABHD3	-0.285	0.3974	abhydrolase domain containing 3
11452	C10orf108	-0.2857	0.4946	chromosome 10 open reading frame 108
11453	VPS8	-0.2858	0.351	vacuolar protein sorting 8 homolog (S. cerevisiae)
11454	SIX3	-0.2858	0.4353	SIX homeobox 3
11455	RAB5A	-0.2858	0.3978	RAB5A, member RAS oncogene family
11456	PSMD11	-0.2858	0.4048	proteasome (prosome, macropain) 26S subunit, non-ATPase, 11
11457	POU6F2	-0.2858	0.4599	POU class 6 homeobox 2
11458	PFKP	-0.2858	0.3372	phosphofructokinase, platelet
11459	KLK3	-0.2858	0.488	kallikrein-related peptidase 3
11460	HAX1	-0.2858	0.3761	HCLS1 associated protein X-1
11461	CYP11A1	-0.2858	0.4015	cytochrome P450, family 11, subfamily A, polypeptide 1
11462	CTRL	-0.2858	0.4109	chymotrypsin-like
11463	ZNF18	-0.2862	0.2908	zinc finger protein 18
11464	PDE7A	-0.2862	0.3723	phosphodiesterase 7A
11465	MBTD1	-0.2862	0.4062	mbt domain containing 1
11466	KLC3	-0.2862	0.4561	kinesin light chain 3
11467	GBA2	-0.2862	0.2435	glucosidase, beta (bile acid) 2
11468	EXOSC6	-0.2862	0.3244	exosome component 6
11469	DIS3L	-0.2862	0.3681	DIS3 mitotic control homolog (S. cerevisiae)-like
11470	C16orf70	-0.2862	0.1782	chromosome 16 open reading frame 70
11471	RPL36	-0.2867	0.3783	ribosomal protein L36
11472	PTK2B	-0.2867	0.3466	PTK2B protein tyrosine kinase 2 beta
11473	PDHA2	-0.2867	0.4522	pyruvate dehydrogenase (lipoamide) alpha 2
11474	MYOD1	-0.2867	0.486	myogenic differentiation 1
11475	MIP	-0.2867	0.4595	major intrinsic protein of lens fiber
11476	GJA3	-0.2867	0.4319	gap junction protein, alpha 3, 46kDa
11477	GAL3ST1	-0.2867	0.4037	galactose-3-O-sulfotransferase 1
11478	FYB	-0.2867	0.3273	FYN binding protein
11479	FDPS	-0.2867	0.3953	farnesyl diphosphate synthase
11480	ATG9A	-0.2867	0.2909	autophagy related 9A
11481	C17orf69	-0.2871	0.3085	chromosome 17 open reading frame 69
11482	ANHX	-0.2871	0.449	anomalous homeobox
11483	ZNRF4	-0.2875	0.4848	zinc and ring finger 4
11484	TNP2	-0.2875	0.4545	transition protein 2 (during histone to protamine replacement)
11485	NCF4	-0.2875	0.3606	neutrophil cytosolic factor 4, 40kDa
11486	MEIS3	-0.2875	0.3809	Meis homeobox 3
11487	LRRC39	-0.2875	0.2516	leucine rich repeat containing 39
11488	CCDC17	-0.2875	0.4146	coiled-coil domain containing 17
11489	BCL2L1	-0.2875	0.2581	BCL2-like 1
11490	BCL11B	-0.2875	0.2461	B-cell CLL/lymphoma 11B (zinc finger protein)
11491	SMC3	-0.2883	0.4092	structural maintenance of chromosomes 3
11492	RHCE	-0.2883	0.2983	Rh blood group, CcEe antigens
11493	PRSS50	-0.2883	0.4611	protease, serine, 50
11494	PPP4R2	-0.2883	0.3583	protein phosphatase 4, regulatory subunit 2
11495	PLA2G5	-0.2883	0.4238	phospholipase A2, group V
11496	NUBP1	-0.2883	0.3674	nucleotide binding protein 1
11497	MMP17	-0.2883	0.4597	matrix metallopeptidase 17 (membrane-inserted)
11498	L2HGDH	-0.2883	0.3239	L-2-hydroxyglutarate dehydrogenase
11499	KIAA0317	-0.2883	0.3776	KIAA0317
11500	GZMB	-0.2883	0.3229	granzyme B (granzyme 2, cytotoxic T-lymphocyte-associated serine esterase 1)
11501	DBH	-0.2883	0.4611	dopamine beta-hydroxylase (dopamine beta-monooxygenase)
11502	COQ7	-0.2883	0.2849	coenzyme Q7 homolog, ubiquinone (yeast)
11503	CEP350	-0.2883	0.3995	centrosomal protein 350kDa
11504	LRRTM1	-0.2887	0.4109	leucine rich repeat transmembrane neuronal 1
11505	KCNA7	-0.2887	0.458	potassium voltage-gated channel, shaker-related subfamily, member 7
11506	ADAMTS19	-0.2887	0.2879	ADAM metallopeptidase with thrombospondin type 1 motif, 19

11507	SRBD1	-0.2892	0.3918	S1 RNA binding domain 1
11508	POLR1E	-0.2892	0.3633	polymerase (RNA) I polypeptide E, 53kDa
11509	PINK1	-0.2892	0.2868	PTEN induced putative kinase 1
11510	NME8	-0.2892	0.3166	NME/NM23 family member 8
11511	MCHR1	-0.2892	0.4444	melanin-concentrating hormone receptor 1
11512	CD28	-0.2892	0.3097	CD28 molecule
11513	BCL2L11	-0.2892	0.1943	BCL2-like 11 (apoptosis facilitator)
11514	ATAD5	-0.2892	0.3332	ATPase family, AAA domain containing 5
11515	AP5S1	-0.2892	0.2512	adaptor-related protein complex 5, sigma 1 subunit
11516	ZNF711	-0.29	0.3234	zinc finger protein 711
11517	ZBTB43	-0.29	0.2895	zinc finger and BTB domain containing 43
11518	TNFRSF1B	-0.29	0.3258	tumor necrosis factor receptor superfamily, member 1B
11519	SLC25A45	-0.29	0.2821	solute carrier family 25, member 45
11520	RBM18	-0.29	0.407	RNA binding motif protein 18
11521	RABGGTA	-0.29	0.3072	Rab geranylgeranyltransferase, alpha subunit
11522	PYY	-0.29	0.4646	peptide YY
11523	PPP1R21	-0.29	0.3792	protein phosphatase 1, regulatory subunit 21
11524	PLAC2	-0.29	0.4315	placenta-specific 2 (non-protein coding)
11525	MED4	-0.29	0.4225	mediator complex subunit 4
11526	MAN2B1	-0.29	0.3163	mannosidase, alpha, class 2B, member 1
11527	GPR27	-0.29	0.455	G protein-coupled receptor 27
11528	GOLGA6L2	-0.29	0.4878	golgin A6 family-like 2
11529	CTDNEP1	-0.29	0.3249	CTD nuclear envelope phosphatase 1
11530	CGGBP1	-0.29	0.3915	CGG triplet repeat binding protein 1
11531	C20orf118	-0.29	0.4681	chromosome 20 open reading frame 118
11532	ALMS1	-0.29	0.3953	Alstrom syndrome 1
11533	ZNF235	-0.2908	0.2963	zinc finger protein 235
11534	UPF3A	-0.2908	0.3607	UPF3 regulator of nonsense transcripts homolog A (yeast)
11535	RPRM	-0.2908	0.3795	reprimo, TP53 dependent G2 arrest mediator candidate
11536	RFPL1-AS1	-0.2908	0.1086	RFPL1 antisense RNA 1 (non-protein coding)
11537	MRPS14	-0.2908	0.3907	mitochondrial ribosomal protein S14
11538	MRFAP1L1	-0.2908	0.4176	Morf4 family associated protein 1-like 1
11539	HPCA	-0.2908	0.4403	hippocalcin
11540	CHIA	-0.2908	0.4701	chitinase, acidic
11541	AHDC1	-0.2908	0.388	AT hook, DNA binding motif, containing 1
11542	ZFP14	-0.2912	0.2511	zinc finger protein 14 homolog (mouse)
11543	TUBGCP6	-0.2912	0.3356	tubulin, gamma complex associated protein 6
11544	PTH2	-0.2912	0.512	parathyroid hormone 2
11545	C3orf45	-0.2912	0.4673	chromosome 3 open reading frame 45
11546	C1orf74	-0.2912	0.2461	chromosome 1 open reading frame 74
11547	SP6	-0.2914	0.4533	Sp6 transcription factor
11548	LOC374443	-0.2914	0.3737	C-type lectin domain family 2, member D pseudogene
11549	SLC2A5	-0.2917	0.3024	solute carrier family 2 (facilitated glucose/fructose transporter), member 5
11550	RWDD1	-0.2917	0.3874	RWD domain containing 1
11551	RALGPS1	-0.2917	0.2569	Ral GEF with PH domain and SH3 binding motif 1
11552	PSMA2	-0.2917	0.4303	proteasome (prosome, macropain) subunit, alpha type, 2
11553	PNMA3	-0.2917	0.4678	paraneoplastic Ma antigen 3
11554	HIST1H4E	-0.2917	0.1935	histone cluster 1, H4e
11555	CD27	-0.2917	0.3632	CD27 molecule
11556	AAMP	-0.2917	0.3297	angio-associated, migratory cell protein
11557	STRA13	-0.2925	0.3739	stimulated by retinoic acid 13 homolog (mouse)
11558	S100A7A	-0.2925	0.3834	S100 calcium binding protein A7A
11559	RCN2	-0.2925	0.421	reticulocalbin 2, EF-hand calcium binding domain
11560	PUM1	-0.2925	0.3946	pumilio homolog 1 (Drosophila)
11561	MORC2-AS1	-0.2925	0.2545	MORC2 antisense RNA 1 (non-protein coding)
11562	LOC283174	-0.2925	0.2726	uncharacterized LOC283174
11563	H2AFV	-0.2925	0.4135	H2A histone family, member V
11564	GPATCH8	-0.2925	0.3175	G patch domain containing 8
11565	EPM2A	-0.2925	0.2432	epilepsy, progressive myoclonus type 2A, Lafora disease (laforin)
11566	CHAC1	-0.2925	0.3315	ChaC, cation transport regulator homolog 1 (E. coli)
11567	CDH24	-0.2925	0.4067	cadherin 24, type 2
11568	ASNSD1	-0.2925	0.4223	asparagine synthetase domain containing 1
11569	TUBB7P	-0.2933	0.9996	tubulin, beta 7, pseudogene
11570	SYCP1	-0.2933	0.3728	synaptonemal complex protein 1
11571	SSTR5	-0.2933	0.4354	somatostatin receptor 5
11572	PIK3R4	-0.2933	0.4023	phosphoinositide-3-kinase, regulatory subunit 4
11573	PFDN1	-0.2933	0.3158	prefoldin subunit 1
11574	KCNH1	-0.2933	0.4351	potassium voltage-gated channel, subfamily H (eag-related), member 1
11575	FLT3	-0.2933	0.3219	fms-related tyrosine kinase 3
11576	AP2M1	-0.2933	0.3587	adaptor-related protein complex 2, mu 1 subunit
11577	RTN4R	-0.2937	0.3669	reticulon 4 receptor
11578	C20orf72	-0.2937	0.4013	chromosome 20 open reading frame 72
11579	PHIP	-0.2942	0.4131	pleckstrin homology domain interacting protein
11580	FTSJ3	-0.2942	0.3482	FtsJ homolog 3 (E. coli)
11581	FAM107A	-0.2942	0.427	family with sequence similarity 107, member A
11582	COX10	-0.2942	0.2585	COX10 homolog, cytochrome c oxidase assembly protein, heme A: farnesyltransferase (yeast)
11583	CLSTN3	-0.2942	0.2998	calsyntenin 3
11584	ATN1	-0.2942	0.4188	atrophin 1
11585	LOC285627	-0.2943	0.9999	uncharacterized LOC285627
11586	TAS2R38	-0.295	0.4264	taste receptor, type 2, member 38
11587	SHKBP1	-0.295	0.3167	SH3KBP1 binding protein 1
11588	SCUBE1	-0.295	0.4332	signal peptide, CUB domain, EGF-like 1
11589	RPP25L	-0.295	0.2889	ribonuclease P/MRP 25kDa subunit-like
11590	PYDC1	-0.295	0.3239	PYD (pyrin domain) containing 1

11591	PANX3	-0.295	0.4543	pannexin 3
11592	NPLOC4	-0.295	0.2829	nuclear protein localization 4 homolog (S. cerevisiae)
11593	MDH1B	-0.295	0.2788	malate dehydrogenase 1B, NAD (soluble)
11594	LOC151121	-0.295	0.9999	uncharacterized LOC151121
11595	IL22	-0.295	0.4439	interleukin 22
11596	HS6ST3	-0.295	0.4106	heparan sulfate 6-O-sulfotransferase 3
11597	HRH2	-0.295	0.4562	histamine receptor H2
11598	HDC	-0.295	0.3803	histidine decarboxylase
11599	DDX23	-0.295	0.3864	DEAD (Asp-Glu-Ala-Asp) box polypeptide 23
11600	CDC20B	-0.295	0.4401	cell division cycle 20 homolog B (S. cerevisiae)
11601	AGPAT4	-0.295	0.1838	1-acylglycerol-3-phosphate O-acyltransferase 4 (lysophosphatidic acid acyltransferase, delta)
11602	ADCY10	-0.295	0.4187	adenylate cyclase 10 (soluble)
11603	RBM4	-0.2955	0.3712	RNA binding motif protein 4
11604	LOC646762	-0.2957	0.1939	uncharacterized LOC646762
11605	FLJ35816	-0.2957	0.4322	FLJ35816 protein
11606	STAMBP	-0.2958	0.4036	STAM binding protein
11607	SLC13A4	-0.2958	0.4573	solute carrier family 13 (sodium/sulfate symporters), member 4
11608	PPP4C	-0.2958	0.3491	protein phosphatase 4, catalytic subunit
11609	NOLC1	-0.2958	0.4002	nucleolar and coiled-body phosphoprotein 1
11610	GDI1	-0.2958	0.2556	GDP dissociation inhibitor 1
11611	CCDC91	-0.2958	0.419	coiled-coil domain containing 91
11612	CCDC40	-0.2958	0.4336	coiled-coil domain containing 40
11613	APTX	-0.2958	0.3954	aprataxin
11614	VN1R5	-0.2962	0.4272	vomeronasal 1 receptor 5 (gene/pseudogene)
11615	SPRR2G	-0.2962	0.455	small proline-rich protein 2G
11616	PLEKHA8	-0.2962	0.1599	pleckstrin homology domain containing, family A (phosphoinositide binding specific) member 8
11617	OTX2	-0.2962	0.4128	orthodenticle homeobox 2
11618	NSUN4	-0.2962	0.2834	NOP2/Sun domain family, member 4
11619	NOG	-0.2962	0.1405	noggin
11620	FMR1NB	-0.2962	0.3892	fragile X mental retardation 1 neighbor
11621	C3orf19	-0.2962	0.3125	chromosome 3 open reading frame 19
11622	TMEM194A	-0.2967	0.398	transmembrane protein 194A
11623	PHF16	-0.2967	0.3681	PHD finger protein 16
11624	PAPOLA	-0.2967	0.4275	poly(A) polymerase alpha
11625	MORC1	-0.2967	0.372	MORC family CW-type zinc finger 1
11626	LGALS13	-0.2967	0.3762	lectin, galactoside-binding, soluble, 13
11627	HIF1AN	-0.2967	0.2872	hypoxia inducible factor 1, alpha subunit inhibitor
11628	CRTAM	-0.2967	0.2588	cytotoxic and regulatory T cell molecule
11629	CDKN2C	-0.2967	0.2736	cyclin-dependent kinase inhibitor 2C (p18, inhibits CDK4)
11630	MSANTD1	-0.2971	0.4363	Myb/SANT-like DNA-binding domain containing 1
11631	UBQLN1	-0.2975	0.3963	ubiquilin 1
11632	SCN1B	-0.2975	0.3935	sodium channel, voltage-gated, type I, beta subunit
11633	SAMD3	-0.2975	0.259	sterile alpha motif domain containing 3
11634	RPL3L	-0.2975	0.4469	ribosomal protein L3-like
11635	RETN	-0.2975	0.4255	resistin
11636	RAI1	-0.2975	0.2527	retinoic acid induced 1
11637	NEFM	-0.2975	0.2233	neurofilament, medium polypeptide
11638	MRPS12	-0.2975	0.37	mitochondrial ribosomal protein S12
11639	LRRC46	-0.2975	0.3373	leucine rich repeat containing 46
11640	KIAA1755	-0.2975	0.4473	KIAA1755
11641	HNRPDL	-0.2975	0.4159	heterogeneous nuclear ribonucleoprotein D-like
11642	GSTCD	-0.2975	0.348	glutathione S-transferase, C-terminal domain containing
11643	GALR3	-0.2975	0.4804	galanin receptor 3
11644	FGFR1OP2	-0.2975	0.4001	FGFR1 oncogene partner 2
11645	FCER2	-0.2975	0.4268	Fc fragment of IgE, low affinity II, receptor for (CD23)
11646	ERC2-IT1	-0.2975	0.4632	ERC2 intronic transcript 1 (non-protein coding)
11647	C12orf54	-0.2975	0.409	chromosome 12 open reading frame 54
11648	ATP5D	-0.2975	0.366	ATP synthase, H+ transporting, mitochondrial F1 complex, delta subunit
11649	KIF17	-0.2983	0.4297	kinesin family member 17
11650	KDM6B	-0.2983	0.3668	lysine (K)-specific demethylase 6B
11651	GRM7	-0.2983	0.4573	glutamate receptor, metabotropic 7
11652	EPX	-0.2983	0.4614	eosinophil peroxidase
11653	DHODH	-0.2983	0.3114	dihydroorotate dehydrogenase (quinone)
11654	CCIN	-0.2983	0.4354	calicin
11655	ATP5G3	-0.2983	0.414	ATP synthase, H+ transporting, mitochondrial Fo complex, subunit C3 (subunit 9)
11656	AOAH	-0.2983	0.3018	acyloxyacyl hydrolase (neutrophil)
11657	SNORA61	-0.2986	0.3567	small nucleolar RNA, H/ACA box 61
11658	WDR38	-0.2988	0.4657	WD repeat domain 38
11659	RIPPLY1	-0.2988	0.4347	ripply1 homolog (zebrafish)
11660	NS3BP	-0.2988	0.4417	NS3BP
11661	CLDND2	-0.2988	0.4486	claudin domain containing 2
11662	C2orf55	-0.2988	0.4012	chromosome 2 open reading frame 55
11663	C20orf94	-0.2988	0.2384	chromosome 20 open reading frame 94
11664	C1orf213	-0.2988	0.3126	chromosome 1 open reading frame 213
11665	UBE4A	-0.2992	0.4077	ubiquitination factor E4A
11666	TFDP3	-0.2992	0.4014	transcription factor Dp family, member 3
11667	SLC4A1	-0.2992	0.4725	solute carrier family 4, anion exchanger, member 1 (erythrocyte membrane protein band 3, Diego blood group)
11668	SCAF8	-0.2992	0.415	SR-related CTD-associated factor 8
11669	RIMS1	-0.2992	0.3752	regulating synaptic membrane exocytosis 1
11670	NFYA	-0.2992	0.3493	nuclear transcription factor Y, alpha
11671	MAP1LC3B	-0.2992	0.3555	microtubule-associated protein 1 light chain 3 beta
11672	FBXL6	-0.2992	0.3523	F-box and leucine-rich repeat protein 6
11673	DRD5	-0.2992	0.4586	dopamine receptor D5
11674	DEFA4	-0.2992	0.3927	defensin, alpha 4, corticostatin

11675	ARG2	-0.2992	0.2091	arginase, type II
11676	SNHG4	-0.3	0.9996	small nucleolar RNA host gene 4 (non-protein coding)
11677	SMIM1	-0.3	0.3868	small integral membrane protein 1
11678	SF3B14	-0.3	0.4064	splicing factor 3B, 14 kDa subunit
11679	SEMA4C	-0.3	0.1915	sema domain, immunoglobulin domain (Ig), transmembrane domain (TM) and short cytoplasmic domain, (semaphorin) 4C
11680	SEL1L2	-0.3	0.4318	sel-1 suppressor of lin-12-like 2 (C. elegans)
11681	RAB15	-0.3	0.1428	RAB15, member RAS oncogene family
11682	NPHP4	-0.3	0.2455	nephronophthisis 4
11683	MED7	-0.3	0.4199	mediator complex subunit 7
11684	LRRC7	-0.3	0.2245	leucine rich repeat containing 7
11685	LOC284632	-0.3	0.9999	uncharacterized LOC284632
11686	GPR45	-0.3	0.4262	G protein-coupled receptor 45
11687	DDX27	-0.3	0.3253	DEAD (Asp-Glu-Ala-Asp) box polypeptide 27
11688	CRHR1	-0.3	0.4812	corticotropin releasing hormone receptor 1
11689	CHST8	-0.3	0.4488	carbohydrate (N-acetylgalactosamine 4-0) sulfotransferase 8
11690	CD3D	-0.3	0.291	CD3d molecule, delta (CD3-TCR complex)
11691	ASPRV1	-0.3	0.3658	aspartic peptidase, retroviral-like 1
11692	UBE2K	-0.3008	0.4253	ubiquitin-conjugating enzyme E2K
11693	SPAG11B	-0.3008	0.3333	sperm associated antigen 11B
11694	PDE1B	-0.3008	0.4267	phosphodiesterase 1B, calmodulin-dependent
11695	LZTR1	-0.3008	0.3076	leucine-zipper-like transcription regulator 1
11696	LSAMP	-0.3008	0.3218	limbic system-associated membrane protein
11697	KLHL1	-0.3008	0.4241	kelch-like 1 (Drosophila)
11698	DNAJA3	-0.3008	0.3732	DnaJ (Hsp40) homolog, subfamily A, member 3
11699	CCBL1	-0.3008	0.27	cysteine conjugate-beta lyase, cytoplasmic
11700	ASIC3	-0.3008	0.4036	acid-sensing (proton-gated) ion channel 3
11701	ZSWIM2	-0.3013	0.3766	zinc finger, SWIM-type containing 2
11702	ZFPM1	-0.3013	0.415	zinc finger protein, multitype 1
11703	PROK1	-0.3013	0.4607	prokineticin 1
11704	POLR2J4	-0.3013	0.3252	polymerase (RNA) II (DNA directed) polypeptide J4, pseudogene
11705	ANKAR	-0.3013	0.2549	ankyrin and armadillo repeat containing
11706	ZDHHC18	-0.3017	0.2756	zinc finger, DHHC-type containing 18
11707	TFAP4	-0.3017	0.3595	transcription factor AP-4 (activating enhancer binding protein 4)
11708	PSMB10	-0.3017	0.3573	proteasome (prosome, macropain) subunit, beta type, 10
11709	PPME1	-0.3017	0.2122	protein phosphatase methylesterase 1
11710	MTA2	-0.3017	0.2196	metastasis associated 1 family, member 2
11711	HIST1H2BE	-0.3017	0.1719	histone cluster 1, H2be
11712	GABRA2	-0.3017	0.1826	gamma-aminobutyric acid (GABA) A receptor, alpha 2
11713	DLGAP2	-0.3017	0.4589	discs, large (Drosophila) homolog-associated protein 2
11714	CHODL	-0.3017	0.3067	chondrolectin
11715	TMEM242	-0.3025	0.1842	transmembrane protein 242
11716	sept-09	-0.3025	0.2413	septin 9
11717	RB1CC1	-0.3025	0.4137	RB1-inducible coiled-coil 1
11718	PXDNL	-0.3025	0.207	peroxidasin homolog (Drosophila)-like
11719	LRRC56	-0.3025	0.4332	leucine rich repeat containing 56
11720	LPPR4	-0.3025	0.2436	lipid phosphate phosphatase-related protein type 4
11721	FAM195B	-0.3025	0.275	family with sequence similarity 195, member B
11722	DNAL4	-0.3025	0.2347	dynein, axonemal, light chain 4
11723	DDX46	-0.3025	0.4155	DEAD (Asp-Glu-Ala-Asp) box polypeptide 46
11724	DDB1	-0.3025	0.3427	damage-specific DNA binding protein 1, 127kDa
11725	DACH1	-0.3025	0.1762	dachshund homolog 1 (Drosophila)
11726	C9orf16	-0.3025	0.3418	chromosome 9 open reading frame 16
11727	VDAC1	-0.3029	0.4133	voltage-dependent anion channel 1
11728	ESRG	-0.3029	0.9996	embryonic stem cell related (non-protein coding)
11729	NARS2	-0.3033	0.3964	asparaginyl-tRNA synthetase 2, mitochondrial (putative)
11730	LOC100287590	-0.3033	0.9999	uncharacterized LOC100287590
11731	KAT2A	-0.3033	0.2895	K(lysine) acetyltransferase 2A
11732	FDXR	-0.3033	0.2723	ferredoxin reductase
11733	DVL1	-0.3033	0.3045	dishevelled, dsh homolog 1 (Drosophila)
11734	DNAJB2	-0.3033	0.2968	DnaJ (Hsp40) homolog, subfamily B, member 2
11735	CDC37	-0.3033	0.3507	cell division cycle 37 homolog (S. cerevisiae)
11736	ATF5	-0.3033	0.2752	activating transcription factor 5
11737	ALK	-0.3033	0.4198	anaplastic lymphoma receptor tyrosine kinase
11738	ZNF683	-0.3038	0.4376	zinc finger protein 683
11739	TEKT5	-0.3038	0.4712	tektin 5
11740	SLC34A3	-0.3038	0.5074	solute carrier family 34 (sodium phosphate), member 3
11741	PYHIN1	-0.3038	0.2882	pyrin and HIN domain family, member 1
11742	PCDHB4	-0.3038	0.2386	protocadherin beta 4
11743	FREM3	-0.3038	0.2914	FRAS1 related extracellular matrix 3
11744	TRIM17	-0.3042	0.4392	tripartite motif containing 17
11745	RPS6KC1	-0.3042	0.3811	ribosomal protein S6 kinase, 52kDa, polypeptide 1
11746	RAPSN	-0.3042	0.4231	receptor-associated protein of the synapse
11747	RAP2B	-0.3042	0.292	RAP2B, member of RAS oncogene family
11748	RAB40C	-0.3042	0.2692	RAB40C, member RAS oncogene family
11749	PLEKHB1	-0.3042	0.1857	pleckstrin homology domain containing, family B (evectins) member 1
11750	MFN2	-0.3042	0.2724	mitofusin 2
11751	DBP	-0.3042	0.2913	D site of albumin promoter (albumin D-box) binding protein
11752	ART3	-0.3042	0.149	ADP-ribosyltransferase 3
11753	AHSP	-0.3042	0.4209	alpha hemoglobin stabilizing protein
11754	LOC100132354	-0.3043	0.4912	uncharacterized LOC100132354
11755	WDR89	-0.305	0.3815	WD repeat domain 89
11756	SPACA7	-0.305	0.4196	sperm acrosome associated 7
11757	SNX3	-0.305	0.3784	sorting nexin 3
11758	OTX1	-0.305	0.45	orthodenticle homeobox 1

11759	NCKAP5L	-0.305	0.377	NCK-associated protein 5-like
11760	MTIF2	-0.305	0.4133	mitochondrial translational initiation factor 2
11761	MMEL1	-0.305	0.3837	membrane metallo-endopeptidase-like 1
11762	LOC554206	-0.305	0.1654	leucine carboxyl methyltransferase 1 pseudogene
11763	KLK2	-0.305	0.4752	kallikrein-related peptidase 2
11764	IL2RG	-0.305	0.3235	interleukin 2 receptor, gamma
11765	DUSP19	-0.305	0.2687	dual specificity phosphatase 19
11766	CIC	-0.305	0.3599	capicua homolog (Drosophila)
11767	CHCHD3	-0.305	0.4095	coiled-coil-helix-coiled-coil-helix domain containing 3
11768	CD8B	-0.305	0.3408	CD8b molecule
11769	CCR1	-0.305	0.3076	chemokine (C-C motif) receptor 1
11770	BZRAP1	-0.305	0.3545	benzodiazapine receptor (peripheral) associated protein 1
11771	LOC158376	-0.3057	0.3962	uncharacterized LOC158376
11772	PRIM2	-0.3058	0.4022	primase, DNA, polypeptide 2 (58kDa)
11773	MYO1F	-0.3058	0.3576	myosin IF
11774	MRPL22	-0.3058	0.4004	mitochondrial ribosomal protein L22
11775	IL4	-0.3058	0.4099	interleukin 4
11776	HIST1H1B	-0.3058	0.2859	histone cluster 1, H1b
11777	G6PC2	-0.3058	0.4626	glucose-6-phosphatase, catalytic, 2
11778	FAM13B	-0.3058	0.4033	family with sequence similarity 13, member B
11779	E4F1	-0.3058	0.3419	E4F transcription factor 1
11780	CTNND2	-0.3058	0.3066	catenin (cadherin-associated protein), delta 2 (neural plakophilin-related arm-repeat protein)
11781	CEP72	-0.3058	0.3193	centrosomal protein 72kDa
11782	APOA2	-0.3058	0.4024	apolipoprotein A-II
11783	TUSC5	-0.3063	0.4505	tumor suppressor candidate 5
11784	EXOC3L4	-0.3063	0.4277	exocyst complex component 3-like 4
11785	YAF2	-0.3067	0.3776	YY1 associated factor 2
11786	TAF8	-0.3067	0.1546	TAF8 RNA polymerase II, TATA box binding protein (TBP)-associated factor, 43kDa
11787	SNX11	-0.3067	0.2539	sorting nexin 11
11788	S1PR4	-0.3067	0.4217	sphingosine-1-phosphate receptor 4
11789	NDEL1	-0.3067	0.3046	nudE nuclear distribution E homolog (A. nidulans)-like 1
11790	AKAP5	-0.3067	0.1815	A kinase (PRKA) anchor protein 5
11791	ZFR2	-0.3075	0.464	zinc finger RNA binding protein 2
11792	UCN2	-0.3075	0.428	urocortin 2
11793	TSC22D1-AS1	-0.3075	0.17	TSC22D1 antisense RNA 1 (non-protein coding)
11794	SPCS2	-0.3075	0.2696	signal peptidase complex subunit 2 homolog (S. cerevisiae)
11795	PDCD2	-0.3075	0.4153	programmed cell death 2
11796	NLGN2	-0.3075	0.4327	neuroligin 2
11797	FBXL19-AS1	-0.3075	0.4126	FBXL19 antisense RNA 1 (non-protein coding)
11798	FAU	-0.3075	0.3636	Finkel-Biskis-Reilly murine sarcoma virus (FBR-MuSV) ubiquitously expressed
11799	EPB41L2	-0.3075	0.3422	erythrocyte membrane protein band 4.1-like 2
11800	DSPP	-0.3075	0.2269	dentin sialophosphoprotein
11801	C5orf45	-0.3075	0.2635	chromosome 5 open reading frame 45
11802	BEST3	-0.3075	0.3703	bestrophin 3
11803	BACH2	-0.3075	0.2528	BTB and CNC homology 1, basic leucine zipper transcription factor 2
11804	SFMBT2	-0.3083	0.2201	Scm-like with four mbt domains 2
11805	NOC3L	-0.3083	0.4188	nucleolar complex associated 3 homolog (S. cerevisiae)
11806	MYO3A	-0.3083	0.4043	myosin IIIA
11807	MAPK7	-0.3083	0.2629	mitogen-activated protein kinase 7
11808	HDHD1	-0.3083	0.3535	haloacid dehalogenase-like hydrolase domain containing 1
11809	FAM46C	-0.3083	0.1602	family with sequence similarity 46, member C
11810	UBE2U	-0.3088	0.1083	ubiquitin-conjugating enzyme E2U (putative)
11811	SELK	-0.3088	0.3144	selenoprotein K
11812	PPM1K	-0.3088	0.3196	protein phosphatase, Mg2+/Mn2+ dependent, 1K
11813	PATE1	-0.3088	0.4426	prostate and testis expressed 1
11814	KANSL1	-0.3088	0.3331	KAT8 regulatory NSL complex subunit 1
11815	ZFPL1	-0.3092	0.3014	zinc finger protein-like 1
11816	WSB2	-0.3092	0.3748	WD repeat and SOCS box containing 2
11817	RLN2	-0.3092	0.201	relaxin 2
11818	HSF1	-0.3092	0.3136	heat shock transcription factor 1
11819	CCDC134	-0.3092	0.3409	coiled-coil domain containing 134
11820	C21orf33	-0.3092	0.3366	chromosome 21 open reading frame 33
11821	C1D	-0.3092	0.4136	C1D nuclear receptor corepressor
11822	AGXT2L1	-0.3092	0.1981	alanine-glyoxylate aminotransferase 2-like 1
11823	WNT9B	-0.31	0.4659	wingless-type MMTV integration site family, member 9B
11824	TMEM66	-0.31	0.39	transmembrane protein 66
11825	THBS4	-0.31	0.2316	thrombospondin 4
11826	TAS2R41	-0.31	0.3796	taste receptor, type 2, member 41
11827	SLC25A26	-0.31	0.2951	solute carrier family 25 (S-adenosylmethionine carrier), member 26
11828	SHROOM1	-0.31	0.3831	shroom family member 1
11829	RNASEH2B	-0.31	0.3797	ribonuclease H2, subunit B
11830	REG3G	-0.31	0.4269	regenerating islet-derived 3 gamma
11831	PNLDC1	-0.31	0.3326	poly(A)-specific ribonuclease (PARN)-like domain containing 1
11832	PHF8	-0.31	0.2236	PHD finger protein 8
11833	OPRD1	-0.31	0.4604	opioid receptor, delta 1
11834	IQCF6	-0.31	0.3618	IQ motif containing F6
11835	GPIHBP1	-0.31	0.458	glycosylphosphatidylinositol anchored high density lipoprotein binding protein 1
11836	ARL4C	-0.31	0.244	ADP-ribosylation factor-like 4C
11837	ARFGAP2	-0.31	0.3106	ADP-ribosylation factor GTPase activating protein 2
11838	APITD1	-0.31	0.3805	apoptosis-inducing, TAF9-like domain 1
11839	ACIN1	-0.31	0.2958	apoptotic chromatin condensation inducer 1
11840	ABCA3	-0.31	0.2348	ATP-binding cassette, sub-family A (ABC1), member 3
11841	TH	-0.3108	0.4215	tyrosine hydroxylase
11842	SLC36A1	-0.3108	0.154	solute carrier family 36 (proton/amino acid symporter), member 1

11843	PLXNB3	-0.3108	0.4286	plexin B3
11844	PET112	-0.3108	0.3107	PET112 homolog (yeast)
11845	MAGEA4	-0.3108	0.2218	melanoma antigen family A, 4
11846	LPXN	-0.3108	0.3259	leupaxin
11847	CFL1	-0.3109	0.332	cofilin 1 (non-muscle)
11848	ZNF397	-0.3113	0.3084	zinc finger protein 397
11849	RNF20	-0.3113	0.3848	ring finger protein 20, E3 ubiquitin protein ligase
11850	PCDHB2	-0.3113	0.1389	protocadherin beta 2
11851	GSX2	-0.3113	0.4631	GS homeobox 2
11852	FAM217A	-0.3113	0.4398	family with sequence similarity 217, member A
11853	CSTT	-0.3113	0.3722	cystatin pseudogene
11854	C5orf58	-0.3113	0.1855	chromosome 5 open reading frame 58
11855	PRSS55	-0.3114	0.4731	protease, serine, 55
11856	PER4	-0.3114	0.9999	period homolog 3 (Drosophila) pseudogene
11857	LOC285501	-0.3114	0.9999	uncharacterized LOC285501
11858	ZNF692	-0.3117	0.2989	zinc finger protein 692
11859	LRIT1	-0.3117	0.4452	leucine-rich repeat, immunoglobulin-like and transmembrane domains 1
11860	KEL	-0.3117	0.4308	Kell blood group, metallo-endopeptidase
11861	EXOC2	-0.3117	0.3774	exocyst complex component 2
11862	DHX15	-0.3117	0.4265	DEAH (Asp-Glu-Ala-His) box polypeptide 15
11863	CD8A	-0.3117	0.3185	CD8a molecule
11864	ASGR1	-0.3117	0.3259	asialoglycoprotein receptor 1
11865	ZBTB5	-0.3125	0.2864	zinc finger and BTB domain containing 5
11866	PRKACA	-0.3125	0.25	protein kinase, cAMP-dependent, catalytic, alpha
11867	PDS5B	-0.3125	0.4079	PDS5, regulator of cohesion maintenance, homolog B (S. cerevisiae)
11868	MOAP1	-0.3125	0.3662	modulator of apoptosis 1
11869	IRF8	-0.3125	0.2519	interferon regulatory factor 8
11870	FUT1	-0.3125	0.3583	fucosyltransferase 1 (galactoside 2-alpha-L-fucosyltransferase, H blood group)
11871	EFNB3	-0.3125	0.4222	ephrin-B3
11872	CRMP1	-0.3125	0.3938	collapsin response mediator protein 1
11873	CXorf64	-0.3129	0.4515	chromosome X open reading frame 64
11874	ZNF271	-0.3133	0.3908	zinc finger protein 271
11875	WDTC1	-0.3133	0.3584	WD and tetratricopeptide repeats 1
11876	VSNL1	-0.3133	0.1982	visinin-like 1
11877	SGSM2	-0.3133	0.3048	small G protein signaling modulator 2
11878	RPL35A	-0.3133	0.3814	ribosomal protein L35a
11879	RGL1	-0.3133	0.2569	ral guanine nucleotide dissociation stimulator-like 1
11880	PTPRT	-0.3133	0.4243	protein tyrosine phosphatase, receptor type, T
11881	NUP43	-0.3133	0.3924	nucleoporin 43kDa
11882	MAPK4	-0.3133	0.3868	mitogen-activated protein kinase 4
11883	IL10	-0.3133	0.324	interleukin 10
11884	GALK1	-0.3133	0.3442	galactokinase 1
11885	CCNJ	-0.3133	0.3773	cyclin J
11886	CACNA1I	-0.3133	0.4651	calcium channel, voltage-dependent, T type, alpha 1I subunit
11887	TCTEX1D2	-0.3137	0.3369	Tctex1 domain containing 2
11888	STK32C	-0.3137	0.364	serine/threonine kinase 32C
11889	PCDH15	-0.3137	0.2007	protocadherin-related 15
11890	MGC34034	-0.3137	0.3942	uncharacterized protein MGC34034
11891	LACTB	-0.3137	0.3628	lactamase, beta
11892	CYP4F22	-0.3137	0.3771	cytochrome P450, family 4, subfamily F, polypeptide 22
11893	CD163L1	-0.3137	0.1794	CD163 molecule-like 1
11894	UBASH3A	-0.3142	0.3163	ubiquitin associated and SH3 domain containing A
11895	RBPJ	-0.3142	0.3256	recombination signal binding protein for immunoglobulin kappa J region
11896	PMS1	-0.3142	0.4149	PMS1 postmeiotic segregation increased 1 (S. cerevisiae)
11897	PCGF1	-0.3142	0.2766	polycomb group ring finger 1
11898	GRIN2A	-0.3142	0.3858	glutamate receptor, ionotropic, N-methyl D-aspartate 2A
11899	GALR2	-0.3142	0.3886	galanin receptor 2
11900	CCDC90A	-0.3142	0.3881	coiled-coil domain containing 90A
11901	BTBD3	-0.3142	0.3845	BTB (POZ) domain containing 3
11902	ATP4A	-0.3142	0.4548	ATPase, H+/K+ exchanging, alpha polypeptide
11903	UFSP1	-0.3143	0.4114	UFM1-specific peptidase 1 (non-functional)
11904	SGK3	-0.3143	0.3695	serum/glucocorticoid regulated kinase family, member 3
11905	PTMA	-0.3143	0.3678	prothymosin, alpha
11906	ZNF498	-0.315	0.2255	zinc finger protein 498
11907	ZNF346	-0.315	0.2254	zinc finger protein 346
11908	THSD7B	-0.315	0.2173	thrombospondin, type I, domain containing 7B
11909	TBC1D14	-0.315	0.2634	TBC1 domain family, member 14
11910	SFXN5	-0.315	0.3039	sideroflexin 5
11911	PRSS53	-0.315	0.4242	protease, serine, 53
11912	NIPSNAP3A	-0.315	0.3887	nipsnap homolog 3A (C. elegans)
11913	MRPL48	-0.315	0.3686	mitochondrial ribosomal protein L48
11914	GGNBP2	-0.315	0.3873	gametogenetin binding protein 2
11915	CECR7	-0.315	0.2772	cat eye syndrome chromosome region, candidate 7 (non-protein coding)
11916	CCDC109B	-0.315	0.363	coiled-coil domain containing 109B
11917	CABP4	-0.315	0.4868	calcium binding protein 4
11918	B4GALNT3	-0.315	0.4227	beta-1,4-N-acetyl-galactosaminyl transferase 3
11919	USP19	-0.3158	0.2546	ubiquitin specific peptidase 19
11920	SOX30	-0.3158	0.2102	SRY (sex determining region Y)-box 30
11921	PRMT5	-0.3158	0.3913	protein arginine methyltransferase 5
11922	NOL6	-0.3158	0.3009	nucleolar protein family 6 (RNA-associated)
11923	CTPS1	-0.3158	0.3866	CTP synthase 1
11924	ASF1A	-0.3158	0.4125	ASF1 anti-silencing function 1 homolog A (S. cerevisiae)
11925	ZRANB1	-0.3162	0.3477	zinc finger, RAN-binding domain containing 1
11926	EPT1	-0.3162	0.3759	ethanolaminephosphotransferase 1 (CDP-ethanolamine-specific)

11927	DGCR9	-0.3164	0.4364	DiGeorge syndrome critical region gene 9
11928	TNFSF9	-0.3167	0.2484	tumor necrosis factor (ligand) superfamily, member 9
11929	NAA40	-0.3167	0.2722	N(alpha)-acetyltransferase 40, NatD catalytic subunit, homolog (S. cerevisiae)
11930	CUL3	-0.3167	0.3963	cullin 3
11931	CD96	-0.3167	0.2606	CD96 molecule
11932	BTBD1	-0.3167	0.4086	BTB (POZ) domain containing 1
11933	ZNF276	-0.3175	0.3244	zinc finger protein 276
11934	WDR20	-0.3175	0.3848	WD repeat domain 20
11935	TNF	-0.3175	0.3433	tumor necrosis factor
11936	TACC1	-0.3175	0.3243	transforming, acidic coiled-coil containing protein 1
11937	PLEKHJ1	-0.3175	0.3121	pleckstrin homology domain containing, family J member 1
11938	OR2H1	-0.3175	0.4737	olfactory receptor, family 2, subfamily H, member 1
11939	NME9	-0.3175	0.3691	NME/NM23 family member 9
11940	LEMD2	-0.3175	0.2599	LEM domain containing 2
11941	CNIH3	-0.3175	0.13	cornichon homolog 3 (Drosophila)
11942	UBE2G2	-0.3183	0.3431	ubiquitin-conjugating enzyme E2G 2
11943	PSMA5	-0.3183	0.4059	proteasome (prosome, macropain) subunit, alpha type, 5
11944	OVOL3	-0.3183	0.4671	ovo-like 3 (Drosophila)
11945	EVI2B	-0.3183	0.3318	ecotropic viral integration site 2B
11946	DNAI2	-0.3183	0.442	dynein, axonemal, intermediate chain 2
11947	CHCHD7	-0.3183	0.372	coiled-coil-helix-coiled-coil-helix domain containing 7
11948	SPEM1	-0.3187	0.48	spermatid maturation 1
11949	KIAA2018	-0.3187	0.3089	KIAA2018
11950	GFM1	-0.3187	0.4071	G elongation factor, mitochondrial 1
11951	C16orf13	-0.3187	0.3304	chromosome 16 open reading frame 13
11952	TCL6	-0.3192	0.4594	T-cell leukemia/lymphoma 6 (non-protein coding)
11953	MEX3D	-0.3192	0.2416	mex-3 homolog D (C. elegans)
11954	IRS4	-0.3192	0.4202	insulin receptor substrate 4
11955	HIST1H3G	-0.3192	0.1823	histone cluster 1, H3g
11956	DROSHA	-0.3192	0.3912	drosha, ribonuclease type III
11957	CFP	-0.3192	0.3894	complement factor properdin
11958	CCK	-0.3192	0.3999	cholecystokinin
11959	AHSG	-0.3192	0.4387	alpha-2-HS-glycoprotein
11960	ZDHHC11	-0.32	0.2676	zinc finger, DHHC-type containing 11
11961	TBXAS1	-0.32	0.2766	thromboxane A synthase 1 (platelet)
11962	SYNDIG1L	-0.32	0.4661	synapse differentiation inducing 1-like
11963	SORCS1	-0.32	0.2533	sortilin-related VPS10 domain containing receptor 1
11964	SNHG11	-0.32	0.2602	small nucleolar RNA host gene 11 (non-protein coding)
11965	SETD1A	-0.32	0.3242	SET domain containing 1A
11966	RPUSD2	-0.32	0.2913	RNA pseudouridylate synthase domain containing 2
11967	PMPCA	-0.32	0.3374	peptidase (mitochondrial processing) alpha
11968	PIH1D2	-0.32	0.2032	PIH1 domain containing 2
11969	MEX3C	-0.32	0.3895	mex-3 homolog C (C. elegans)
11970	KPNA6	-0.32	0.2629	karyopherin alpha 6 (importin alpha 7)
11971	ITPK1	-0.32	0.2785	inositol-tetrakisphosphate 1-kinase
11972	HCST	-0.32	0.3174	hematopoietic cell signal transducer
11973	GCG	-0.32	0.3239	glucagon
11974	FLYWCH2	-0.32	0.3069	FLYWCH family member 2
11975	FAM189A1	-0.32	0.4097	family with sequence similarity 189, member A1
11976	FABP7	-0.32	0.2056	fatty acid binding protein 7, brain
11977	ETV5	-0.32	0.125	ets variant 5
11978	CCDC113	-0.32	0.2517	coiled-coil domain containing 113
11979	C14orf64	-0.32	0.9996	chromosome 14 open reading frame 64
11980	AMZ1	-0.32	0.4739	archaelysin family metallopeptidase 1
11981	PREB	-0.3208	0.3316	prolactin regulatory element binding
11982	HBS1L	-0.3208	0.3625	HBS1-like (S. cerevisiae)
11983	GNA13	-0.3208	0.3555	guanine nucleotide binding protein (G protein), alpha 13
11984	CRYGC	-0.3208	0.4416	crystallin, gamma C
11985	C20orf43	-0.3208	0.3495	chromosome 20 open reading frame 43
11986	AIP	-0.3208	0.2999	aryl hydrocarbon receptor interacting protein
11987	NFASC	-0.3209	0.3626	neurofascin
11988	MTCP1NB	-0.3209	0.3531	mature T-cell proliferation 1 neighbor
11989	FAM9A	-0.3212	0.1607	family with sequence similarity 9, member A
11990	RPL27	-0.3214	0.3685	ribosomal protein L27
11991	CDRT15L2	-0.3214	0.9999	CMT1A duplicated region transcript 15-like 2
11992	TCEB2	-0.3217	0.2889	transcription elongation factor B (SIII), polypeptide 2 (18kDa, elongin B)
11993	SNTG2	-0.3217	0.3377	syntrophin, gamma 2
11994	RNF2	-0.3217	0.3385	ring finger protein 2
11995	LRRC42	-0.3217	0.331	leucine rich repeat containing 42
11996	LAPTM5	-0.3217	0.3082	lysosomal protein transmembrane 5
11997	EMR2	-0.3217	0.2405	egf-like module containing, mucin-like, hormone receptor-like 2
11998	YTHDC1	-0.3225	0.3948	YTH domain containing 1
11999	WIPI2	-0.3225	0.2007	WD repeat domain, phosphoinositide interacting 2
12000	UNK	-0.3225	0.2133	unkempt homolog (Drosophila)
12001	PROX2	-0.3225	0.1531	prospero homeobox 2
12002	P2RX5	-0.3225	0.1781	purinergic receptor P2X, ligand-gated ion channel, 5
12003	MPO	-0.3225	0.4141	myeloperoxidase
12004	MAGEA1	-0.3225	0.231	melanoma antigen family A, 1 (directs expression of antigen MZ2-E)
12005	LOC100128098	-0.3225	0.3882	uncharacterized LOC100128098
12006	LGALS12	-0.3225	0.4144	lectin, galactoside-binding, soluble, 12
12007	GPR161	-0.3225	0.3003	G protein-coupled receptor 161
12008	FBXO24	-0.3225	0.4358	F-box protein 24
12009	DKFZP434H168	-0.3225	0.4222	uncharacterized LOC26077
12010	CYLC2	-0.3225	0.3717	cylicin, basic protein of sperm head cytoskeleton 2

12011	ATF7IP2	-0.3225	0.1903	activating transcription factor 7 interacting protein 2
12012	ADCYAP1R1	-0.3225	0.4217	adenylate cyclase activating polypeptide 1 (pituitary) receptor type I
12013	THPO	-0.3233	0.4447	thrombopoietin
12014	TFB1M	-0.3233	0.3543	transcription factor B1, mitochondrial
12015	RINT1	-0.3233	0.4049	RAD50 interactor 1
12016	NVL	-0.3233	0.3523	nuclear VCP-like
12017	LPHN3	-0.3233	0.2176	latrophilin 3
12018	IPO5	-0.3233	0.4155	importin 5
12019	GNAI3	-0.3233	0.4157	guanine nucleotide binding protein (G protein), alpha inhibiting activity polypeptide 3
12020	CELA2A	-0.3233	0.4266	chymotrypsin-like elastase family, member 2A
12021	BTN2A1	-0.3233	0.3024	butyrophilin, subfamily 2, member A1
12022	ASTE1	-0.3233	0.283	asteroid homolog 1 (Drosophila)
12023	ZNF583	-0.3237	0.2297	zinc finger protein 583
12024	MIF4GD	-0.3237	0.2693	MIF4G domain containing
12025	LOC286359	-0.3237	0.4287	uncharacterized LOC286359
12026	KCNH5	-0.3237	0.4025	potassium voltage-gated channel, subfamily H (eag-related), member 5
12027	ITLN1	-0.3237	0.2868	intelectin 1 (galactofuranose binding)
12028	FBXO16	-0.3237	0.1618	F-box protein 16
12029	CEP41	-0.3237	0.3538	centrosomal protein 41kDa
12030	UBQLN3	-0.3242	0.4463	ubiquilin 3
12031	TSTD2	-0.3242	0.3797	thiosulfate sulfurtransferase (rhodanese)-like domain containing 2
12032	TMEM48	-0.3242	0.4117	transmembrane protein 48
12033	SH3GLB2	-0.3242	0.2938	SH3-domain GRB2-like endophilin B2
12034	PUS3	-0.3242	0.3358	pseudouridylate synthase 3
12035	PRKACG	-0.3242	0.4632	protein kinase, cAMP-dependent, catalytic, gamma
12036	PPP1R17	-0.3242	0.4023	protein phosphatase 1, regulatory subunit 17
12037	MED15	-0.3242	0.2993	mediator complex subunit 15
12038	DCHS2	-0.3242	0.4305	dachsous 2 (Drosophila)
12039	COL9A1	-0.3242	0.4257	collagen, type IX, alpha 1
12040	RPL19	-0.3243	0.3357	ribosomal protein L19
12041	TSPY26P	-0.325	0.4165	testis specific protein, Y-linked 26, pseudogene
12042	TET3	-0.325	0.2189	tet methylcytosine dioxygenase 3
12043	SYT7	-0.325	0.428	synaptotagmin VII
12044	SIRT7	-0.325	0.2759	sirtuin 7
12045	PROCA1	-0.325	0.4268	protein interacting with cyclin A1
12046	NLE1	-0.325	0.3215	notchless homolog 1 (Drosophila)
12047	NFYC	-0.325	0.3211	nuclear transcription factor Y, gamma
12048	NEFH	-0.325	0.1138	neurofilament, heavy polypeptide
12049	LYSMD4	-0.325	0.1507	LysM, putative peptidoglycan-binding, domain containing 4
12050	LOC284648	-0.325	0.4152	uncharacterized LOC284648
12051	FAM26F	-0.325	0.2826	family with sequence similarity 26, member F
12052	CSTF1	-0.325	0.3964	cleavage stimulation factor, 3' pre-RNA, subunit 1, 50kDa
12053	C2orf88	-0.325	0.1287	chromosome 2 open reading frame 88
12054	ASZ1	-0.325	0.2286	ankyrin repeat, SAM and basic leucine zipper domain containing 1
12055	ANKRD7	-0.325	0.1889	ankyrin repeat domain 7
12056	AFF3	-0.325	0.2907	AF4/FMR2 family, member 3
12057	PSMC6	-0.3257	0.4231	proteasome (prosome, macropain) 26S subunit, ATPase, 6
12058	ZNF384	-0.3258	0.2443	zinc finger protein 384
12059	TMCC1	-0.3258	0.3098	transmembrane and coiled-coil domain family 1
12060	INSIG1	-0.3258	0.3108	insulin induced gene 1
12061	DCXR	-0.3258	0.309	dicarbonyl/L-xylulose reductase
12062	ZNF418	-0.3262	0.2385	zinc finger protein 418
12063	ZIC4	-0.3262	0.402	Zic family member 4
12064	UMODL1	-0.3262	0.1944	uromodulin-like 1
12065	GNGT2	-0.3262	0.2993	guanine nucleotide binding protein (G protein), gamma transducing activity polypeptide 2
12066	RBMX2	-0.3267	0.3507	RNA binding motif protein, X-linked 2
12067	PLA2G1B	-0.3267	0.3527	phospholipase A2, group IB (pancreas)
12068	NADK	-0.3267	0.2705	NAD kinase
12069	MPPED1	-0.3267	0.4473	metallophosphoesterase domain containing 1
12070	LCK	-0.3267	0.3066	lymphocyte-specific protein tyrosine kinase
12071	LARP7	-0.3267	0.4024	La ribonucleoprotein domain family, member 7
12072	FCER1G	-0.3267	0.3029	Fc fragment of IgE, high affinity I, receptor for; gamma polypeptide
12073	DKKL1	-0.3267	0.4141	dickkopf-like 1
12074	COX5B	-0.3267	0.3574	cytochrome c oxidase subunit Vb
12075	CAMK1G	-0.3267	0.4259	calcium/calmodulin-dependent protein kinase IG
12076	ADRA1B	-0.3267	0.3635	adrenoceptor alpha 1B
12077	ZNF560	-0.3275	0.1372	zinc finger protein 560
12078	ZNF252P-AS1	-0.3275	0.3352	ZNF252P antisense RNA 1 (non-protein coding)
12079	SUB1	-0.3275	0.4122	SUB1 homolog (S. cerevisiae)
12080	SLC30A10	-0.3275	0.294	solute carrier family 30, member 10
12081	RTCA	-0.3275	0.41	RNA 3'-terminal phosphate cyclase
12082	MRPS11	-0.3275	0.3675	mitochondrial ribosomal protein S11
12083	LOC400027	-0.3275	0.2945	uncharacterized LOC400027
12084	FAM26D	-0.3275	0.21	family with sequence similarity 26, member D
12085	CD1C	-0.3275	0.3001	CD1c molecule
12086	ABCG8	-0.3275	0.4201	ATP-binding cassette, sub-family G (WHITE), member 8
12087	UBE2G1	-0.3283	0.3982	ubiquitin-conjugating enzyme E2G 1
12088	TBC1D22B	-0.3283	0.1424	TBC1 domain family, member 22B
12089	SMAP1	-0.3283	0.3815	small ArfGAP 1
12090	PMP2	-0.3283	0.2902	peripheral myelin protein 2
12091	PHF14	-0.3283	0.3829	PHD finger protein 14
12092	PDE6H	-0.3283	0.2941	phosphodiesterase 6H, cGMP-specific, cone, gamma
12093	NDUFB5	-0.3283	0.4037	NADH dehydrogenase (ubiquinone) 1 beta subcomplex, 5, 16kDa
12094	GK2	-0.3283	0.4055	glycerol kinase 2

12095	BAMBI	-0.3283	0.1408	BMP and activin membrane-bound inhibitor homolog (Xenopus laevis)
12096	AKIP1	-0.3283	0.2915	A kinase (PRKA) interacting protein 1
12097	TMC1	-0.3288	0.3549	transmembrane channel-like 1
12098	TEX26	-0.3288	0.264	testis expressed 26
12099	SLC25A35	-0.3288	0.2577	solute carrier family 25, member 35
12100	MFSD2A	-0.3288	0.2027	major facilitator superfamily domain containing 2A
12101	LOC338799	-0.3288	0.3009	uncharacterized LOC338799
12102	HMGB1	-0.3288	0.3899	high mobility group box 1
12103	HDAC10	-0.3288	0.4215	histone deacetylase 10
12104	SUSD4	-0.3292	0.2229	sushi domain containing 4
12105	PALM	-0.3292	0.3551	paralemmin
12106	NDUFB7	-0.3292	0.367	NADH dehydrogenase (ubiquinone) 1 beta subcomplex, 7, 18kDa
12107	EPM2AIP1	-0.3292	0.3742	EPM2A (laforin) interacting protein 1
12108	CHN2	-0.3292	0.134	chimerin (chimaerin) 2
12109	CAND1	-0.3292	0.4199	cullin-associated and neddylation-dissociated 1
12110	CALB1	-0.3292	0.2134	calbindin 1, 28kDa
12111	ATP13A2	-0.3292	0.2859	ATPase type 13A2
12112	RBM22	-0.33	0.3802	RNA binding motif protein 22
12113	PNKD	-0.33	0.2741	paroxysmal nonkinesigenic dyskinesia
12114	KIAA1751	-0.33	0.4472	KIAA1751
12115	KIAA0528	-0.33	0.3974	KIAA0528
12116	GAPDH	-0.33	0.3373	glyceraldehyde-3-phosphate dehydrogenase
12117	FBXW7	-0.33	0.306	F-box and WD repeat domain containing 7, E3 ubiquitin protein ligase
12118	EXOSC7	-0.33	0.319	exosome component 7
12119	DYRK4	-0.33	0.2146	dual-specificity tyrosine-(Y)-phosphorylation regulated kinase 4
12120	DNAJC19	-0.33	0.3672	DnaJ (Hsp40) homolog, subfamily C, member 19
12121	C12orf5	-0.33	0.3792	chromosome 12 open reading frame 5
12122	ZNF768	-0.3308	0.2571	zinc finger protein 768
12123	SSTR2	-0.3308	0.3852	somatostatin receptor 2
12124	SPTBN4	-0.3308	0.4664	spectrin, beta, non-erythrocytic 4
12125	PRKCZ	-0.3308	0.2039	protein kinase C, zeta
12126	PICK1	-0.3308	0.337	protein interacting with PRKCA 1
12127	NSD1	-0.3308	0.1564	nuclear receptor binding SET domain protein 1
12128	MAN2A2	-0.3308	0.2112	mannosidase, alpha, class 2A, member 2
12129	CDK2	-0.3308	0.3038	cyclin-dependent kinase 2
12130	ACYP2	-0.3308	0.3225	acylphosphatase 2, muscle type
12131	ACO2	-0.3308	0.3132	aconitase 2, mitochondrial
12132	USP42	-0.3313	0.2865	ubiquitin specific peptidase 42
12133	SLC25A47	-0.3313	0.4847	solute carrier family 25, member 47
12134	FAM19A4	-0.3313	0.2329	family with sequence similarity 19 (chemokine (C-C motif)-like), member A4
12135	DERL3	-0.3313	0.3779	derlin 3
12136	ACBD6	-0.3313	0.3448	acyl-CoA binding domain containing 6
12137	NPHP3-AS1	-0.3314	0.9999	NPHP3 antisense RNA 1 (non-protein coding)
12138	WDR77	-0.3317	0.344	WD repeat domain 77
12139	TSHB	-0.3317	0.4171	thyroid stimulating hormone, beta
12140	TRO	-0.3317	0.2032	trophinin
12141	SRSF11	-0.3317	0.4064	serine/arginine-rich splicing factor 11
12142	SPACA1	-0.3317	0.415	sperm acrosome associated 1
12143	RXRG	-0.3317	0.3757	retinoid X receptor, gamma
12144	PTPRU	-0.3317	0.3249	protein tyrosine phosphatase, receptor type, U
12145	LEMD3	-0.3317	0.3975	LEM domain containing 3
12146	HSP90AB1	-0.3317	0.3865	heat shock protein 90kDa alpha (cytosolic), class B member 1
12147	DLX5	-0.3317	0.2501	distal-less homeobox 5
12148	DDX43	-0.3317	0.0656	DEAD (Asp-Glu-Ala-Asp) box polypeptide 43
12149	ASB1	-0.3317	0.2098	ankyrin repeat and SOCS box containing 1
12150	C1orf95	-0.3322	0.4416	chromosome 1 open reading frame 95
12151	ZNF576	-0.3325	0.2841	zinc finger protein 576
12152	WAC	-0.3325	0.3854	WW domain containing adaptor with coiled-coil
12153	TSPAN16	-0.3325	0.4146	tetraspanin 16
12154	TIMM8A	-0.3325	0.365	translocase of inner mitochondrial membrane 8 homolog A (yeast)
12155	TFRC	-0.3325	0.3527	transferrin receptor (p90, CD71)
12156	SMC6	-0.3325	0.4003	structural maintenance of chromosomes 6
12157	RAPGEF5	-0.3325	0.1959	Rap guanine nucleotide exchange factor (GEF) 5
12158	P2RY4	-0.3325	0.4422	pyrimidinergic receptor P2Y, G-protein coupled, 4
12159	ORC4	-0.3325	0.3953	origin recognition complex, subunit 4
12160	NKPD1	-0.3325	0.4045	NTPase, KAP family P-loop domain containing 1
12161	LINC00598	-0.3325	0.4497	long intergenic non-protein coding RNA 598
12162	FAM120C	-0.3325	0.1279	family with sequence similarity 120C
12163	DPEP2	-0.3325	0.3349	dipeptidase 2
12164	CYCS	-0.3325	0.3764	cytochrome c, somatic
12165	C11orf21	-0.3325	0.427	chromosome 11 open reading frame 21
12166	TFDP1	-0.3333	0.3745	transcription factor Dp-1
12167	SUPT6H	-0.3333	0.2496	suppressor of Ty 6 homolog (S. cerevisiae)
12168	SLC25A31	-0.3333	0.1639	solute carrier family 25 (mitochondrial carrier; adenine nucleotide translocator), member 31
12169	RBM34	-0.3333	0.4104	RNA binding motif protein 34
12170	PPA1	-0.3333	0.3896	pyrophosphatase (inorganic) 1
12171	KCNQ3	-0.3333	0.4463	potassium voltage-gated channel, KQT-like subfamily, member 3
12172	GUCA1A	-0.3333	0.4338	guanylate cyclase activator 1A (retina)
12173	FTSJ2	-0.3333	0.3846	FtsJ RNA methyltransferase homolog 2 (E. coli)
12174	C21orf2	-0.3333	0.4066	chromosome 21 open reading frame 2
12175	TIAM2	-0.3338	0.0988	T-cell lymphoma invasion and metastasis 2
12176	TFAP2D	-0.3338	0.4384	transcription factor AP-2 delta (activating enhancer binding protein 2 delta)
12177	RAB39B	-0.3338	0.231	RAB39B, member RAS oncogene family
12178	OR5V1	-0.3338	0.4136	olfactory receptor, family 5, subfamily V, member 1

12179	DYNLRB2	-0.3338	0.2095	dynein, light chain, roadblock-type 2
12180	DLX1	-0.3338	0.2731	distal-less homeobox 1
12181	TARS	-0.3342	0.3975	threonyl-tRNA synthetase
12182	R3HDM4	-0.3342	0.2865	R3H domain containing 4
12183	PRLH	-0.3342	0.4687	prolactin releasing hormone
12184	KDM8	-0.3342	0.3253	lysine (K)-specific demethylase 8
12185	DFNB31	-0.3342	0.3281	deafness, autosomal recessive 31
12186	CRNKL1	-0.3342	0.4017	crooked neck pre-mRNA splicing factor-like 1 (Drosophila)
12187	CCL25	-0.3342	0.4115	chemokine (C-C motif) ligand 25
12188	BRD2	-0.3342	0.2681	bromodomain containing 2
12189	APOC2	-0.3343	0.2727	apolipoprotein C-II
12190	ZNF575	-0.335	0.3574	zinc finger protein 575
12191	SOX21	-0.335	0.4443	SRY (sex determining region Y)-box 21
12192	SH2D3C	-0.335	0.3135	SH2 domain containing 3C
12193	PIGH	-0.335	0.3417	phosphatidylinositol glycan anchor biosynthesis, class H
12194	MICB	-0.335	0.295	MHC class I polypeptide-related sequence B
12195	LOC441601	-0.335	0.3412	septin 7 pseudogene
12196	LOC100128822	-0.335	0.3504	uncharacterized LOC100128822
12197	CST11	-0.335	0.4367	cystatin 11
12198	COL9A2	-0.335	0.3271	collagen, type IX, alpha 2
12199	CD83	-0.335	0.197	CD83 molecule
12200	CCDC85C	-0.3358	0.194	coiled-coil domain containing 85C
12201	AP2A2	-0.3358	0.2425	adaptor-related protein complex 2, alpha 2 subunit
12202	KCNQ5	-0.3363	0.1248	potassium voltage-gated channel, KQT-like subfamily, member 5
12203	C9orf24	-0.3363	0.3564	chromosome 9 open reading frame 24
12204	ZMIZ2	-0.3367	0.3304	zinc finger, MIZ-type containing 2
12205	PRAF2	-0.3367	0.2532	PRA1 domain family, member 2
12206	KPNA5	-0.3367	0.2922	karyopherin alpha 5 (importin alpha 6)
12207	IK	-0.3367	0.3761	IK cytokine, down-regulator of HLA II
12208	CD244	-0.3367	0.3296	CD244 molecule, natural killer cell receptor 2B4
12209	ASIC4	-0.3367	0.4552	acid-sensing (proton-gated) ion channel family member 4
12210	LOC644456	-0.3371	0.9996	IK cytokine, down-regulator of HLA II pseudogene
12211	SCN4B	-0.3375	0.2557	sodium channel, voltage-gated, type IV, beta subunit
12212	RPP30	-0.3375	0.3621	ribonuclease P/MRP 30kDa subunit
12213	RGS17	-0.3375	0.1922	regulator of G-protein signaling 17
12214	NRIP3	-0.3375	0.1733	nuclear receptor interacting protein 3
12215	METTL21C	-0.3375	0.2609	methyltransferase like 21C
12216	G3BP1	-0.3375	0.3843	GTPase activating protein (SH3 domain) binding protein 1
12217	FREM1	-0.3375	0.2001	FRAS1 related extracellular matrix 1
12218	FCRL4	-0.3375	0.404	Fc receptor-like 4
12219	FBXO44	-0.3375	0.3689	F-box protein 44
12220	FASN	-0.3375	0.2689	fatty acid synthase
12221	CD52	-0.3375	0.2729	CD52 molecule
12222	CA11	-0.3375	0.2376	carbonic anhydrase XI
12223	C20orf26	-0.3375	0.3881	chromosome 20 open reading frame 26
12224	C17orf78	-0.3375	0.3337	chromosome 17 open reading frame 78
12225	C12orf4	-0.3375	0.3761	chromosome 12 open reading frame 4
12226	ANGPT4	-0.3375	0.4548	angiopoietin 4
12227	RBM8A	-0.3382	0.3938	RNA binding motif protein 8A
12228	UBE2Q1	-0.3383	0.3211	ubiquitin-conjugating enzyme E2Q family member 1
12229	TRBV20-1	-0.3383	0.9999	T cell receptor beta variable 20-1
12230	TERF1	-0.3383	0.367	telomeric repeat binding factor (NIMA-interacting) 1
12231	OPRL1	-0.3383	0.438	opiate receptor-like 1
12232	IDI2-AS1	-0.3383	0.213	IDI2 antisense RNA 1 (non-protein coding)
12233	HTR3A	-0.3383	0.4414	5-hydroxytryptamine (serotonin) receptor 3A, ionotropic
12234	GOLGA8B	-0.3383	0.3327	golgin A8 family, member B
12235	FAM99B	-0.3383	0.9999	family with sequence similarity 99, member B (non-protein coding)
12236	CTR9	-0.3383	0.4033	Ctr9, Paf1/RNA polymerase II complex component, homolog (S. cerevisiae)
12237	CLASRP	-0.3383	0.2767	CLK4-associating serine/arginine rich protein
12238	C15orf39	-0.3383	0.2743	chromosome 15 open reading frame 39
12239	BIN3	-0.3383	0.2096	bridging integrator 3
12240	BAHCC1	-0.3383	0.368	BAH domain and coiled-coil containing 1
12241	ATG4C	-0.3383	0.3648	autophagy related 4C, cysteine peptidase
12242	ZFP57	-0.3388	0.3342	zinc finger protein 57 homolog (mouse)
12243	SCLT1	-0.3388	0.3646	sodium channel and clathrin linker 1
12244	RASSF5	-0.3388	0.2299	Ras association (RalGDS/AF-6) domain family member 5
12245	LRRCC1	-0.3388	0.3411	leucine rich repeat and coiled-coil centrosomal protein 1
12246	LRRC71	-0.3388	0.4281	leucine rich repeat containing 71
12247	LOC145783	-0.3388	0.0999	uncharacterized LOC145783
12248	IDO2	-0.3388	0.2519	indoleamine 2,3-dioxygenase 2
12249	GABRR3	-0.3388	0.3839	gamma-aminobutyric acid (GABA) A receptor, rho 3
12250	ARMC5	-0.3388	0.4024	armadillo repeat containing 5
12251	RET	-0.3392	0.3606	ret proto-oncogene
12252	POLG	-0.3392	0.2458	polymerase (DNA directed), gamma
12253	NOVA2	-0.3392	0.4428	neuro-oncological ventral antigen 2
12254	GABRA4	-0.3392	0.3483	gamma-aminobutyric acid (GABA) A receptor, alpha 4
12255	DGKD	-0.3392	0.1771	diacylglycerol kinase, delta 130kDa
12256	DAPK1	-0.3392	0.1812	death-associated protein kinase 1
12257	C1QB	-0.3392	0.2784	complement component 1, q subcomponent, B chain
12258	AATK	-0.3392	0.4302	apoptosis-associated tyrosine kinase
12259	ZSCAN4	-0.34	0.3004	zinc finger and SCAN domain containing 4
12260	TMEM52	-0.34	0.3375	transmembrane protein 52
12261	SPATC1	-0.34	0.4693	spermatogenesis and centriole associated 1
12262	RAB9BP1	-0.34	0.125	RAB9B, member RAS oncogene family pseudogene 1

12263	PLXNA4	-0.34	0.3266	plexin A4
12264	PGC	-0.34	0.441	progastricsin (pepsinogen C)
12265	NTN1	-0.34	0.3729	netrin 1
12266	LUC7L3	-0.34	0.4059	LUC7-like 3 (S. cerevisiae)
12267	GOLT1A	-0.34	0.3212	golgi transport 1A
12268	CPNE4	-0.34	0.1302	copine IV
12269	C20orf166-AS1	-0.34	0.4671	C20orf166 antisense RNA 1 (non-protein coding)
12270	C12orf74	-0.34	0.3092	chromosome 12 open reading frame 74
12271	ATOX1	-0.34	0.3012	ATX1 antioxidant protein 1 homolog (yeast)
12272	USP6NL	-0.3408	0.3634	USP6 N-terminal like
12273	TUBGCP3	-0.3408	0.4024	tubulin, gamma complex associated protein 3
12274	SUMO1	-0.3408	0.4101	SMT3 suppressor of mif two 3 homolog 1 (S. cerevisiae)
12275	PA2G4	-0.3408	0.3734	proliferation-associated 2G4, 38kDa
12276	OVGP1	-0.3408	0.3495	oviductal glycoprotein 1, 120kDa
12277	GRIA3	-0.3408	0.3425	glutamate receptor, ionotropic, AMPA 3
12278	CDYL	-0.3408	0.3379	chromodomain protein, Y-like
12279	CARTPT	-0.3408	0.3811	CART prepropeptide
12280	ASTN1	-0.3408	0.2865	astrotactin 1
12281	TRIM50	-0.3413	0.4256	tripartite motif containing 50
12282	SCML4	-0.3413	0.2838	sex comb on midleg-like 4 (Drosophila)
12283	OR1C1	-0.3413	0.3818	olfactory receptor, family 1, subfamily C, member 1
12284	LOC84856	-0.3413	0.2982	uncharacterized LOC84856
12285	FSCB	-0.3413	0.3951	fibrous sheath CABYR binding protein
12286	CDPF1	-0.3413	0.2046	cysteine-rich, DPF motif domain containing 1
12287	CACNG8	-0.3413	0.4683	calcium channel, voltage-dependent, gamma subunit 8
12288	TMC6	-0.3417	0.2773	transmembrane channel-like 6
12289	POLL	-0.3417	0.3442	polymerase (DNA directed), lambda
12290	HSPB11	-0.3417	0.3963	heat shock protein family B (small), member 11
12291	GPR25	-0.3417	0.4309	G protein-coupled receptor 25
12292	DCC	-0.3417	0.3729	deleted in colorectal carcinoma
12293	CSH1	-0.3417	0.3693	chorionic somatomammotropin hormone 1 (placental lactogen)
12294	CNBP	-0.3417	0.4019	CCHC-type zinc finger, nucleic acid binding protein
12295	C11orf9	-0.3417	0.3832	chromosome 11 open reading frame 9
12296	ARL2	-0.3417	0.2665	ADP-ribosylation factor-like 2
12297	ARC	-0.3417	0.392	activity-regulated cytoskeleton-associated protein
12298	ZNF282	-0.3425	0.2813	zinc finger protein 282
12299	ZDHHC13	-0.3425	0.3701	zinc finger, DHHC-type containing 13
12300	ST8SIA5	-0.3425	0.3262	ST8 alpha-N-acetyl-neuraminide alpha-2,8-sialyltransferase 5
12301	SLC22A12	-0.3425	0.483	solute carrier family 22 (organic anion/urate transporter), member 12
12302	SIRT1	-0.3425	0.3891	sirtuin 1
12303	RAD23B	-0.3425	0.4045	RAD23 homolog B (S. cerevisiae)
12304	NFAM1	-0.3425	0.432	NFAT activating protein with ITAM motif 1
12305	MED19	-0.3425	0.2306	mediator complex subunit 19
12306	MDH2	-0.3425	0.3668	malate dehydrogenase 2, NAD (mitochondrial)
12307	GYG1	-0.3425	0.3484	glycogenin 1
12308	GPR160	-0.3425	0.288	G protein-coupled receptor 160
12309	EXOC5	-0.3425	0.3991	exocyst complex component 5
12310	EPB41	-0.3425	0.155	erythrocyte membrane protein band 4.1 (elliptocytosis 1, RH-linked)
12311	E2F4	-0.3425	0.3328	E2F transcription factor 4, p107/p130-binding
12312	CNTN2	-0.3425	0.454	contactin 2 (axonal)
12313	C2orf56	-0.3425	0.3819	chromosome 2 open reading frame 56
12314	ALAS2	-0.3425	0.4033	aminolevulinate, delta-, synthase 2
12315	RPS9	-0.3429	0.3213	ribosomal protein S9
12316	LOC100289495	-0.3429	0.9999	uncharacterized LOC100289495
12317	GPX1	-0.3429	0.2803	glutathione peroxidase 1
12318	DUSP28	-0.3429	0.2772	dual specificity phosphatase 28
12319	UVRAG	-0.3433	0.3209	UV radiation resistance associated gene
12320	UQCRQ	-0.3433	0.3667	ubiquinol-cytochrome c reductase, complex III subunit VII, 9.5kDa
12321	TBCCD1	-0.3433	0.3726	TBCC domain containing 1
12322	RPS27A	-0.3433	0.3736	ribosomal protein S27a
12323	RMND5B	-0.3433	0.25	required for meiotic nuclear division 5 homolog B (S. cerevisiae)
12324	MYCNOS	-0.3433	0.4327	MYCN opposite strand/antisense RNA (non-protein coding)
12325	KCTD17	-0.3433	0.3896	potassium channel tetramerisation domain containing 17
12326	KBTBD11	-0.3433	0.1374	kelch repeat and BTB (POZ) domain containing 11
12327	HARS	-0.3433	0.3534	histidyl-tRNA synthetase
12328	GNAI2	-0.3433	0.2777	guanine nucleotide binding protein (G protein), alpha inhibiting activity polypeptide 2
12329	DIAPH1	-0.3433	0.207	diaphanous homolog 1 (Drosophila)
12330	C5orf30	-0.3433	0.3352	chromosome 5 open reading frame 30
12331	BRD3	-0.3433	0.284	bromodomain containing 3
12332	ZNF285	-0.3438	0.2183	zinc finger protein 285
12333	SPATA16	-0.3438	0.425	spermatogenesis associated 16
12334	SLC25A42	-0.3438	0.3828	solute carrier family 25, member 42
12335	EVI5L	-0.3438	0.3967	ecotropic viral integration site 5-like
12336	EFCAB7	-0.3438	0.3826	EF-hand calcium binding domain 7
12337	CSN1S2AP	-0.3438	0.4042	casein alpha s2-like A, pseudogene
12338	ZNF24	-0.3442	0.3683	zinc finger protein 24
12339	UCHL1	-0.3442	0.1354	ubiquitin carboxyl-terminal esterase L1 (ubiquitin thiolesterase)
12340	POU2F2	-0.3442	0.3906	POU class 2 homeobox 2
12341	ORM2	-0.3442	0.1443	orosomucoid 2
12342	MSI1	-0.3442	0.4081	musashi homolog 1 (Drosophila)
12343	MED6	-0.3442	0.3951	mediator complex subunit 6
12344	IGSF1	-0.3442	0.3379	immunoglobulin superfamily, member 1
12345	GPR12	-0.3442	0.4422	G protein-coupled receptor 12
12346	AAK1	-0.3442	0.2306	AP2 associated kinase 1

12347	CCDC147	-0.3443	0.2416	coiled-coil domain containing 147
12348	C2orf81	-0.3443	0.4295	chromosome 2 open reading frame 81
12349	XPO7	-0.345	0.2923	exportin 7
12350	WSCD1	-0.345	0.3638	WSC domain containing 1
12351	VIPR2	-0.345	0.4266	vasoactive intestinal peptide receptor 2
12352	SRF	-0.345	0.1973	serum response factor (c-fos serum response element-binding transcription factor)
12353	PPM1J	-0.345	0.2933	protein phosphatase, Mg2+/Mn2+ dependent, 1J
12354	POLA1	-0.345	0.3801	polymerase (DNA directed), alpha 1, catalytic subunit
12355	PIK3R5	-0.345	0.369	phosphoinositide-3-kinase, regulatory subunit 5
12356	PFKFB3	-0.345	0.2207	6-phosphofructo-2-kinase/fructose-2,6-biphosphatase 3
12357	MGC23270	-0.345	0.4041	uncharacterized LOC196872
12358	LRRC16B	-0.345	0.4451	leucine rich repeat containing 16B
12359	GRSF1	-0.345	0.4074	G-rich RNA sequence binding factor 1
12360	FAM215A	-0.345	0.4404	family with sequence similarity 215, member A (non-protein coding)
12361	CTRB2	-0.345	0.4471	chymotrypsinogen B2
12362	COL27A1	-0.345	0.4369	collagen, type XXVII, alpha 1
12363	CHADL	-0.345	0.4244	chondroadherin-like
12364	CCDC106	-0.345	0.2862	coiled-coil domain containing 106
12365	CCAR1	-0.345	0.3848	cell division cycle and apoptosis regulator 1
12366	CALHM3	-0.345	0.3765	calcium homeostasis modulator 3
12367	AJAP1	-0.345	0.3723	adherens junctions associated protein 1
12368	DCAF4L1	-0.3457	0.1908	DDB1 and CUL4 associated factor 4-like 1
12369	CEP170	-0.3457	0.3518	centrosomal protein 170kDa
12370	XRCC4	-0.3458	0.3906	X-ray repair complementing defective repair in Chinese hamster cells 4
12371	RYR3	-0.3458	0.2002	ryanodine receptor 3
12372	PPP3CB	-0.3458	0.3481	protein phosphatase 3, catalytic subunit, beta isozyme
12373	MRPS34	-0.3458	0.3381	mitochondrial ribosomal protein S34
12374	MMP24	-0.3458	0.4179	matrix metallopeptidase 24 (membrane-inserted)
12375	LHX3	-0.3458	0.4561	LIM homeobox 3
12376	DGKB	-0.3458	0.2486	diacylglycerol kinase, beta 90kDa
12377	CELA2B	-0.3458	0.4254	chymotrypsin-like elastase family, member 2B
12378	ZNF597	-0.3462	0.183	zinc finger protein 597
12379	TM6SF2	-0.3462	0.4375	transmembrane 6 superfamily member 2
12380	FAM221B	-0.3462	0.383	family with sequence similarity 221, member B
12381	TAS2R3	-0.3467	0.2992	taste receptor, type 2, member 3
12382	SLC6A11	-0.3467	0.3865	solute carrier family 6 (neurotransmitter transporter, GABA), member 11
12383	NTSR2	-0.3467	0.4337	neurotensin receptor 2
12384	MS4A5	-0.3467	0.4103	membrane-spanning 4-domains, subfamily A, member 5
12385	LINC00574	-0.3467	0.4335	long intergenic non-protein coding RNA 574
12386	TTTY5	-0.3475	0.4615	testis-specific transcript, Y-linked 5 (non-protein coding)
12387	TRIM59	-0.3475	0.3436	tripartite motif containing 59
12388	TRIM41	-0.3475	0.2266	tripartite motif containing 41
12389	SCO1	-0.3475	0.3538	SCO cytochrome oxidase deficient homolog 1 (yeast)
12390	RGS12	-0.3475	0.3523	regulator of G-protein signaling 12
12391	NPAS3	-0.3475	0.3838	neuronal PAS domain protein 3
12392	LPIN3	-0.3475	0.4098	lipin 3
12393	HDAC9	-0.3475	0.165	histone deacetylase 9
12394	FAM71E2	-0.3475	0.4666	family with sequence similarity 71, member E2
12395	DBR1	-0.3475	0.3977	debranching enzyme homolog 1 (S. cerevisiae)
12396	CWC22	-0.3475	0.3829	CWC22 spliceosome-associated protein homolog (S. cerevisiae)
12397	CSF3R	-0.3475	0.3667	colony stimulating factor 3 receptor (granulocyte)
12398	CBX4	-0.3475	0.2042	chromobox homolog 4
12399	C9orf78	-0.3475	0.3391	chromosome 9 open reading frame 78
12400	ATG16L2	-0.3475	0.2742	autophagy related 16-like 2 (S. cerevisiae)
12401	ASGR2	-0.3475	0.4071	asialoglycoprotein receptor 2
12402	PPCDC	-0.3483	0.2393	phosphopantothenoylcysteine decarboxylase
12403	PIP5K1C	-0.3483	0.3053	phosphatidylinositol-4-phosphate 5-kinase, type I, gamma
12404	PIAS2	-0.3483	0.3337	protein inhibitor of activated STAT, 2
12405	MXD3	-0.3483	0.3728	MAX dimerization protein 3
12406	FKBP8	-0.3483	0.3363	FK506 binding protein 8, 38kDa
12407	CDK2AP1	-0.3483	0.3456	cyclin-dependent kinase 2 associated protein 1
12408	ARF5	-0.3483	0.2825	ADP-ribosylation factor 5
12409	LOC100130992	-0.3486	0.9999	uncharacterized LOC100130992
12410	VPS26B	-0.3487	0.2313	vacuolar protein sorting 26 homolog B (S. pombe)
12411	SWSAP1	-0.3487	0.2376	SWIM-type zinc finger 7 associated protein 1
12412	LOC253039	-0.3487	0.2694	uncharacterized LOC253039
12413	FAM47B	-0.3487	0.3345	family with sequence similarity 47, member B
12414	CCDC37	-0.3487	0.3867	coiled-coil domain containing 37
12415	REV3L	-0.3492	0.3891	REV3-like, polymerase (DNA directed), zeta, catalytic subunit
12416	PEX10	-0.3492	0.269	peroxisomal biogenesis factor 10
12417	LAS1L	-0.3492	0.303	LAS1-like (S. cerevisiae)
12418	CHAT	-0.3492	0.4492	choline O-acetyltransferase
12419	BEND5	-0.3492	0.1544	BEN domain containing 5
12420	ZNF746	-0.35	0.2304	zinc finger protein 746
12421	RPAP1	-0.35	0.2277	RNA polymerase II associated protein 1
12422	RNF139	-0.35	0.3707	ring finger protein 139
12423	PEX26	-0.35	0.1177	peroxisomal biogenesis factor 26
12424	NPAS4	-0.35	0.3529	neuronal PAS domain protein 4
12425	MYO15A	-0.35	0.4465	myosin XVA
12426	MGC15885	-0.35	0.3225	uncharacterized protein MGC15885
12427	LOC84931	-0.35	0.4425	uncharacterized LOC84931
12428	LINC00628	-0.35	0.9999	long intergenic non-protein coding RNA 628
12429	LHB	-0.35	0.428	luteinizing hormone beta polypeptide
12430	HIST1H2BM	-0.35	0.2107	histone cluster 1, H2bm

12431	CATSPER2	-0.35	0.1544	cation channel, sperm associated 2
12432	C17orf82	-0.35	0.4721	chromosome 17 open reading frame 82
12433	AQPEP	-0.35	0.2575	laeverin
12434	ZC3H4	-0.3508	0.3155	zinc finger CCCH-type containing 4
12435	TRIM44	-0.3508	0.344	tripartite motif containing 44
12436	TCL1A	-0.3508	0.3514	T-cell leukemia/lymphoma 1A
12437	FCN1	-0.3508	0.3471	ficolin (collagen/fibrinogen domain containing) 1
12438	COLQ	-0.3508	0.3303	collagen-like tail subunit (single strand of homotrimer) of asymmetric acetylcholinesterase
12439	ZNF256	-0.3512	0.2535	zinc finger protein 256
12440	WFDC8	-0.3512	0.3874	WAP four-disulfide core domain 8
12441	MRPL37	-0.3512	0.3409	mitochondrial ribosomal protein L37
12442	HIPK4	-0.3512	0.4399	homeodomain interacting protein kinase 4
12443	C9orf72	-0.3512	0.3228	chromosome 9 open reading frame 72
12444	C6orf226	-0.3512	0.2264	chromosome 6 open reading frame 226
12445	C19orf18	-0.3512	0.1688	chromosome 19 open reading frame 18
12446	TRBV7-3	-0.3514	0.9999	T cell receptor beta variable 7-3
12447	LOC148145	-0.3514	0.9999	uncharacterized LOC148145
12448	PEX16	-0.3517	0.3696	peroxisomal biogenesis factor 16
12449	KCTD5	-0.3517	0.286	potassium channel tetramerisation domain containing 5
12450	IL24	-0.3517	0.2454	interleukin 24
12451	FNDC8	-0.3517	0.4168	fibronectin type III domain containing 8
12452	COMMD4	-0.3517	0.329	COMM domain containing 4
12453	CNR2	-0.3517	0.4201	cannabinoid receptor 2 (macrophage)
12454	AOC2	-0.3517	0.2119	amine oxidase, copper containing 2 (retina-specific)
12455	UNC5CL	-0.3525	0.1877	unc-5 homolog C (C. elegans)-like
12456	THOC1	-0.3525	0.3635	THO complex 1
12457	SOWAHA	-0.3525	0.2716	sosondowah ankyrin repeat domain family member A
12458	RPUSD1	-0.3525	0.3525	RNA pseudouridylate synthase domain containing 1
12459	RAF1	-0.3525	0.3278	v-raf-1 murine leukemia viral oncogene homolog 1
12460	PIH1D3	-0.3525	0.3703	PIH1 domain containing 3
12461	PEX5L	-0.3525	0.3616	peroxisomal biogenesis factor 5-like
12462	PCDP1	-0.3525	0.3358	primary ciliary dyskinesia protein 1
12463	LRRC4	-0.3525	0.2422	leucine rich repeat containing 4
12464	LENG1	-0.3525	0.25	leukocyte receptor cluster (LRC) member 1
12465	HHLA1	-0.3525	0.4652	HERV-H LTR-associating 1
12466	GATA4	-0.3525	0.429	GATA binding protein 4
12467	DCP2	-0.3525	0.3827	DCP2 decapping enzyme homolog (S. cerevisiae)
12468	CHIT1	-0.3525	0.3512	chitinase 1 (chitotriosidase)
12469	C6orf10	-0.3525	0.3239	chromosome 6 open reading frame 10
12470	ATG13	-0.3525	0.1858	autophagy related 13
12471	LOC100130987	-0.3529	0.9999	uncharacterized LOC100130987
12472	TUBA1A	-0.3533	0.2683	tubulin, alpha 1a
12473	STMN3	-0.3533	0.1543	stathmin-like 3
12474	OR7A10	-0.3533	0.2335	olfactory receptor, family 7, subfamily A, member 10
12475	MSH4	-0.3533	0.2499	mutS homolog 4 (E. coli)
12476	MAGEA11	-0.3533	0.2088	melanoma antigen family A, 11
12477	GPKOW	-0.3533	0.2747	G patch domain and KOW motifs
12478	ZNF490	-0.3537	0.1862	zinc finger protein 490
12479	PPP1R3F	-0.3537	0.4163	protein phosphatase 1, regulatory subunit 3F
12480	OR5L2	-0.3537	0.3193	olfactory receptor, family 5, subfamily L, member 2
12481	LOC100128288	-0.3537	0.2157	uncharacterized LOC100128288
12482	FAM71B	-0.3537	0.4605	family with sequence similarity 71, member B
12483	CPNE5	-0.3537	0.2358	copine V
12484	CCDC89	-0.3537	0.1128	coiled-coil domain containing 89
12485	ALG10	-0.3537	0.2983	asparagine-linked glycosylation 10, alpha-1,2-glucosyltransferase homolog (S. pombe)
12486	ABCB5	-0.3537	0.39	ATP-binding cassette, sub-family B (MDR/TAP), member 5
12487	SDHAF1	-0.3542	0.2501	succinate dehydrogenase complex assembly factor 1
12488	RPS6KA5	-0.3542	0.2928	ribosomal protein S6 kinase, 90kDa, polypeptide 5
12489	MON1B	-0.3542	0.2051	MON1 homolog B (yeast)
12490	LHX1	-0.3542	0.3919	LIM homeobox 1
12491	KPNA3	-0.3542	0.36	karyopherin alpha 3 (importin alpha 4)
12492	GPR68	-0.3542	0.3339	G protein-coupled receptor 68
12493	CD86	-0.3542	0.2828	CD86 molecule
12494	CD4	-0.3542	0.326	CD4 molecule
12495	SCARNA2	-0.3543	0.9996	small Cajal body-specific RNA 2
12496	C15orf54	-0.3543	0.22	chromosome 15 open reading frame 54
12497	ST8SIA1	-0.355	0.1224	ST8 alpha-N-acetyl-neuraminide alpha-2,8-sialyltransferase 1
12498	PTBP2	-0.355	0.3816	polypyrimidine tract binding protein 2
12499	PLB1	-0.355	0.3542	phospholipase B1
12500	NUDCD1	-0.355	0.3921	NudC domain containing 1
12501	MYO5A	-0.355	0.3211	myosin VA (heavy chain 12, myoxin)
12502	LOC151658	-0.355	0.2784	uncharacterized LOC151658
12503	ISG15	-0.355	0.2542	ISG15 ubiquitin-like modifier
12504	HIST1H2BF	-0.355	0.1259	histone cluster 1, H2bf
12505	CAMP	-0.355	0.3239	cathelicidin antimicrobial peptide
12506	B3GALT6	-0.355	0.2367	UDP-Gal:betaGal beta 1,3-galactosyltransferase polypeptide 6
12507	ACER1	-0.355	0.4165	alkaline ceramidase 1
12508	LOC644794	-0.3557	0.9996	uncharacterized LOC644794
12509	TTLL5	-0.3558	0.2686	tubulin tyrosine ligase-like family, member 5
12510	TMEM186	-0.3558	0.3256	transmembrane protein 186
12511	SPATA2	-0.3558	0.1574	spermatogenesis associated 2
12512	MATR3	-0.3558	0.416	matrin 3
12513	KRT3	-0.3558	0.4118	keratin 3
12514	BAG4	-0.3558	0.3052	BCL2-associated athanogene 4

12515	TMEM126A	-0.3562	0.3843	transmembrane protein 126A
12516	SRMS	-0.3562	0.3502	src-related kinase lacking C-terminal regulatory tyrosine and N-terminal myristylation sites
12517	SERPINA9	-0.3562	0.4451	serpin peptidase inhibitor, clade A (alpha-1 antiproteinase, antitrypsin), member 9
12518	MESDC1	-0.3562	0.2035	mesoderm development candidate 1
12519	LINC00589	-0.3562	0.373	long intergenic non-protein coding RNA 589
12520	CDK15	-0.3562	0.3763	cyclin-dependent kinase 15
12521	C15orf23	-0.3562	0.347	chromosome 15 open reading frame 23
12522	ANO4	-0.3562	0.1288	anoctamin 4
12523	AKR1E2	-0.3562	0.2045	aldo-keto reductase family 1, member E2
12524	AFMID	-0.3562	0.2011	arylformamidase
12525	SNF8	-0.3567	0.3013	SNF8, ESCRT-II complex subunit, homolog (S. cerevisiae)
12526	PPP6C	-0.3567	0.3754	protein phosphatase 6, catalytic subunit
12527	NFIL3	-0.3567	0.2902	nuclear factor, interleukin 3 regulated
12528	LOC100130815	-0.3567	0.9999	uncharacterized LOC100130815
12529	HAUS2	-0.3567	0.3008	HAUS augmin-like complex, subunit 2
12530	DDX21	-0.3567	0.3832	DEAD (Asp-Glu-Ala-Asp) box helicase 21
12531	BAZ1A	-0.3567	0.3851	bromodomain adjacent to zinc finger domain, 1A
12532	ATG2A	-0.3567	0.2655	autophagy related 2A
12533	ARHGAP15	-0.3567	0.2547	Rho GTPase activating protein 15
12534	ZNF79	-0.3575	0.1209	zinc finger protein 79
12535	REPS1	-0.3575	0.3347	RALBP1 associated Eps domain containing 1
12536	PLAC1L	-0.3575	0.3485	placenta-specific 1-like
12537	NCOA6	-0.3575	0.3353	nuclear receptor coactivator 6
12538	LRRC36	-0.3575	0.368	leucine rich repeat containing 36
12539	GLE1	-0.3575	0.2082	GLE1 RNA export mediator homolog (yeast)
12540	CHRM1	-0.3575	0.3686	cholinergic receptor, muscarinic 1
12541	C17orf64	-0.3575	0.3483	chromosome 17 open reading frame 64
12542	KHDC1L	-0.3582	0.3589	KH homology domain containing 1-like
12543	ETV2	-0.3582	0.3636	ets variant 2
12544	ZNF267	-0.3583	0.3731	zinc finger protein 267
12545	TMEM38B	-0.3583	0.3727	transmembrane protein 38B
12546	TIPRL	-0.3583	0.3843	TIP41, TOR signaling pathway regulator-like (S. cerevisiae)
12547	TIMM17A	-0.3583	0.3957	translocase of inner mitochondrial membrane 17 homolog A (yeast)
12548	LIPE	-0.3583	0.388	lipase, hormone-sensitive
12549	LAMP5	-0.3583	0.1296	lysosomal-associated membrane protein family, member 5
12550	GABRB3	-0.3583	0.2247	gamma-aminobutyric acid (GABA) A receptor, beta 3
12551	DVL2	-0.3583	0.2313	dishevelled, dsh homolog 2 (Drosophila)
12552	BRD9	-0.3583	0.2222	bromodomain containing 9
12553	ACTL6A	-0.3583	0.412	actin-like 6A
12554	SNORA68	-0.3586	0.1698	small nucleolar RNA, H/ACA box 68
12555	SPACA4	-0.3587	0.4182	sperm acrosome associated 4
12556	PDILT	-0.3587	0.3508	protein disulfide isomerase-like, testis expressed
12557	LPAR5	-0.3587	0.1782	lysophosphatidic acid receptor 5
12558	CXorf61	-0.3587	0.1423	chromosome X open reading frame 61
12559	C19orf12	-0.3587	0.1858	chromosome 19 open reading frame 12
12560	LRCH4	-0.3591	0.3817	leucine-rich repeats and calponin homology (CH) domain containing 4
12561	WDR43	-0.3592	0.3766	WD repeat domain 43
12562	RBP1	-0.3592	0.12	retinol binding protein 1, cellular
12563	PSME3	-0.3592	0.3452	proteasome (prosome, macropain) activator subunit 3 (PA28 gamma; Ki)
12564	GPM6B	-0.3592	0.187	glycoprotein M6B
12565	EED	-0.3592	0.3908	embryonic ectoderm development
12566	C3AR1	-0.3592	0.2561	complement component 3a receptor 1
12567	ZNF646	-0.36	0.3122	zinc finger protein 646
12568	YIF1B	-0.36	0.3666	Yip1 interacting factor homolog B (S. cerevisiae)
12569	TCEB3	-0.36	0.1918	transcription elongation factor B (SIII), polypeptide 3 (110kDa, elongin A)
12570	PNPLA5	-0.36	0.4262	patatin-like phospholipase domain containing 5
12571	PLAC1	-0.36	0.2296	placenta-specific 1
12572	PCSK9	-0.36	0.2793	proprotein convertase subtilisin/kexin type 9
12573	NDE1	-0.36	0.1465	nudE nuclear distribution E homolog 1 (A. nidulans)
12574	MLL2	-0.36	0.2336	myeloid/lymphoid or mixed-lineage leukemia 2
12575	MIS18BP1	-0.36	0.3679	MIS18 binding protein 1
12576	LRRN4	-0.36	0.3627	leucine rich repeat neuronal 4
12577	LARP1B	-0.36	0.336	La ribonucleoprotein domain family, member 1B
12578	HAMP	-0.36	0.3035	hepcidin antimicrobial peptide
12579	GABRA5	-0.36	0.3492	gamma-aminobutyric acid (GABA) A receptor, alpha 5
12580	FBL	-0.36	0.3704	fibrillarin
12581	CIAO1	-0.36	0.3272	cytosolic iron-sulfur protein assembly 1
12582	AMZ2P1	-0.36	0.3294	archaelysin family metallopeptidase 2 pseudogene 1
12583	ABAT	-0.36	0.1946	4-aminobutyrate aminotransferase
12584	TLE4	-0.3608	0.2226	transducin-like enhancer of split 4 (E(sp1) homolog, Drosophila)
12585	SNX2	-0.3608	0.3918	sorting nexin 2
12586	MYBBP1A	-0.3608	0.2909	MYB binding protein (P160) 1a
12587	METTL3	-0.3608	0.3718	methyltransferase like 3
12588	KREMEN2	-0.3608	0.4079	kringle containing transmembrane protein 2
12589	KEAP1	-0.3608	0.3013	kelch-like ECH-associated protein 1
12590	HK2	-0.3608	0.2205	hexokinase 2
12591	GRM4	-0.3608	0.4296	glutamate receptor, metabotropic 4
12592	DDX54	-0.3608	0.2896	DEAD (Asp-Glu-Ala-Asp) box polypeptide 54
12593	SPDYA	-0.3613	0.1557	speedy homolog A (Xenopus laevis)
12594	PLCZ1	-0.3613	0.1269	phospholipase C, zeta 1
12595	MEGF10	-0.3613	0.1844	multiple EGF-like-domains 10
12596	KLHL8	-0.3613	0.3265	kelch-like 8 (Drosophila)
12597	CHST9-AS1	-0.3613	0.1684	CHST9 antisense RNA 1 (non-protein coding)
12598	C6orf165	-0.3613	0.2551	chromosome 6 open reading frame 165

12599	FLJ16779	-0.3614	0.9999	uncharacterized LOC100192386
12600	FIGLA	-0.3614	0.3494	folliculogenesis specific basic helix-loop-helix
12601	URB1	-0.3617	0.1814	URB1 ribosome biogenesis 1 homolog (S. cerevisiae)
12602	OPRK1	-0.3617	0.3719	opioid receptor, kappa 1
12603	MYB	-0.3617	0.2398	v-myb myeloblastosis viral oncogene homolog (avian)
12604	MRPL33	-0.3617	0.3428	mitochondrial ribosomal protein L33
12605	HOXB-AS3	-0.3617	0.9996	HOXB cluster antisense RNA 3 (non-protein coding)
12606	GABPB1	-0.3617	0.3741	GA binding protein transcription factor, beta subunit 1
12607	FAM70A	-0.3617	0.1413	family with sequence similarity 70, member A
12608	BRD1	-0.3617	0.3033	bromodomain containing 1
12609	ATP1A2	-0.3617	0.2988	ATPase, Na+/K+ transporting, alpha 2 polypeptide
12610	TDRD1	-0.3625	0.0801	tudor domain containing 1
12611	SLC2A8	-0.3625	0.2736	solute carrier family 2 (facilitated glucose transporter), member 8
12612	NPC1L1	-0.3625	0.4283	NPC1 (Niemann-Pick disease, type C1, gene)-like 1
12613	NLRP9	-0.3625	0.3082	NLR family, pyrin domain containing 9
12614	NABP2	-0.3625	0.265	nucleic acid binding protein 2
12615	KISS1	-0.3625	0.4215	KiSS-1 metastasis-suppressor
12616	KCNK10	-0.3625	0.3835	potassium channel, subfamily K, member 10
12617	FAM136A	-0.3625	0.3582	family with sequence similarity 136, member A
12618	C1orf177	-0.3625	0.4185	chromosome 1 open reading frame 177
12619	ADAM29	-0.3625	0.4057	ADAM metallopeptidase domain 29
12620	UQCRFS1	-0.3629	0.3493	ubiquinol-cytochrome c reductase, Rieske iron-sulfur polypeptide 1
12621	C2orf71	-0.3629	0.4446	chromosome 2 open reading frame 71
12622	PUS1	-0.3633	0.3462	pseudouridylate synthase 1
12623	MRM1	-0.3633	0.3015	mitochondrial rRNA methyltransferase 1 homolog (S. cerevisiae)
12624	GUCY2D	-0.3633	0.4127	guanylate cyclase 2D, membrane (retina-specific)
12625	CELF1	-0.3633	0.2743	CUGBP, Elav-like family member 1
12626	BTK	-0.3633	0.2087	Bruton agammaglobulinemia tyrosine kinase
12627	BCAN	-0.3633	0.4599	brevican
12628	UBE2R2	-0.3638	0.2078	ubiquitin-conjugating enzyme E2R 2
12629	TIGD1	-0.3638	0.3009	tigger transposable element derived 1
12630	SUV420H2	-0.3638	0.4038	suppressor of variegation 4-20 homolog 2 (Drosophila)
12631	CCDC117	-0.3638	0.3213	coiled-coil domain containing 117
12632	C21orf88	-0.3638	0.4078	chromosome 21 open reading frame 88
12633	VPS33B	-0.3642	0.2608	vacuolar protein sorting 33 homolog B (yeast)
12634	PSMF1	-0.3642	0.3138	proteasome (prosome, macropain) inhibitor subunit 1 (PI31)
12635	NXT1	-0.3642	0.3029	NTF2-like export factor 1
12636	NRF1	-0.3642	0.2198	nuclear respiratory factor 1
12637	METTL21D	-0.3642	0.3884	methyltransferase like 21D
12638	DPPA4	-0.3642	0.1929	developmental pluripotency associated 4
12639	DNAH17	-0.3642	0.4187	dynein, axonemal, heavy chain 17
12640	ANKZF1	-0.3642	0.232	ankyrin repeat and zinc finger domain containing 1
12641	SNX15	-0.3643	0.164	sorting nexin 15
12642	VNN2	-0.365	0.2299	vanin 2
12643	TRPC5	-0.365	0.2294	transient receptor potential cation channel, subfamily C, member 5
12644	TMPRSS6	-0.365	0.4622	transmembrane protease, serine 6
12645	NR0B1	-0.365	0.242	nuclear receptor subfamily 0, group B, member 1
12646	LOC645431	-0.365	0.074	uncharacterized LOC645431
12647	LILRA3	-0.365	0.2803	leukocyte immunoglobulin-like receptor, subfamily A (without TM domain), member 3
12648	C2orf72	-0.365	0.3713	chromosome 2 open reading frame 72
12649	LOC340357	-0.3657	0.9996	uncharacterized LOC340357
12650	TRAT1	-0.3658	0.207	T cell receptor associated transmembrane adaptor 1
12651	SQLE	-0.3658	0.2732	squalene epoxidase
12652	SNRNP70	-0.3658	0.2952	small nuclear ribonucleoprotein 70kDa (U1)
12653	KIF3C	-0.3658	0.1621	kinesin family member 3C
12654	HIST1H2BB	-0.3658	0.1165	histone cluster 1, H2bb
12655	ELANE	-0.3658	0.3765	elastase, neutrophil expressed
12656	BTG4	-0.3658	0.3168	B-cell translocation gene 4
12657	BRD8	-0.3658	0.3019	bromodomain containing 8
12658	ATP8A1	-0.3658	0.23	ATPase, aminophospholipid transporter (APLT), class I, type 8A, member 1
12659	ATP5B	-0.3658	0.3516	ATP synthase, H+ transporting, mitochondrial F1 complex, beta polypeptide
12660	RASL11A	-0.3663	0.1864	RAS-like, family 11, member A
12661	GTSF1L	-0.3663	0.4221	gametocyte specific factor 1-like
12662	GGN	-0.3663	0.4409	gametogenetin
12663	ADCK4	-0.3663	0.3011	aarF domain containing kinase 4
12664	UBAP2L	-0.3667	0.2468	ubiquitin associated protein 2-like
12665	SHFM1	-0.3667	0.3429	split hand/foot malformation (ectrodactyly) type 1
12666	RPL18AP3	-0.3667	0.9996	ribosomal protein L18a pseudogene 3
12667	PDYN	-0.3667	0.3931	prodynorphin
12668	MAPK12	-0.3667	0.2349	mitogen-activated protein kinase 12
12669	HMHB1	-0.3667	0.402	histocompatibility (minor) HB-1
12670	GRAMD4	-0.3667	0.1804	GRAM domain containing 4
12671	GPR85	-0.3667	0.1203	G protein-coupled receptor 85
12672	DGKQ	-0.3667	0.3249	diacylglycerol kinase, theta 110kDa
12673	ACACA	-0.3667	0.25	acetyl-CoA carboxylase alpha
12674	ZSCAN2	-0.3675	0.1747	zinc finger and SCAN domain containing 2
12675	TYRO3	-0.3675	0.23	TYRO3 protein tyrosine kinase
12676	TARSL2	-0.3675	0.1706	threonyl-tRNA synthetase-like 2
12677	PVRIG	-0.3675	0.292	poliovirus receptor related immunoglobulin domain containing
12678	PRF1	-0.3675	0.2841	perforin 1 (pore forming protein)
12679	OXCT1	-0.3675	0.3444	3-oxoacid CoA transferase 1
12680	OR10C1	-0.3675	0.4255	olfactory receptor, family 10, subfamily C, member 1
12681	HOXD13	-0.3675	0.3736	homeobox D13
12682	FAM19A2	-0.3675	0.142	family with sequence similarity 19 (chemokine (C-C motif)-like), member A2

12683	CWC15	-0.3675	0.3164	CWC15 spliceosome-associated protein homolog (S. cerevisiae)
12684	CHRM4	-0.3675	0.3577	cholinergic receptor, muscarinic 4
12685	BCL7B	-0.3675	0.2273	B-cell CLL/lymphoma 7B
12686	ARPP21	-0.3675	0.3104	cAMP-regulated phosphoprotein, 21kDa
12687	AP5Z1	-0.3675	0.2828	adaptor-related protein complex 5, zeta 1 subunit
12688	ACAP1	-0.3675	0.3288	ArfGAP with coiled-coil, ankyrin repeat and PH domains 1
12689	YBX2	-0.3683	0.4062	Y box binding protein 2
12690	SIT1	-0.3683	0.3488	signaling threshold regulating transmembrane adaptor 1
12691	PQLC1	-0.3683	0.1962	PQ loop repeat containing 1
12692	MTHFD1	-0.3683	0.3673	methylenetetrahydrofolate dehydrogenase (NADP+ dependent) 1, methenyltetrahydrofolate cyclohydrolase, formyltetrahydrofolate synthetase
12693	MRPL34	-0.3683	0.3242	mitochondrial ribosomal protein L34
12694	MLLT1	-0.3683	0.215	myeloid/lymphoid or mixed-lineage leukemia (trithorax homolog, Drosophila); translocated to, 1
12695	HIST1H3I	-0.3683	0.151	histone cluster 1, H3i
12696	FSCN3	-0.3683	0.4302	fascin homolog 3, actin-bundling protein, testicular (Strongylocentrotus purpuratus)
12697	DNTTIP2	-0.3683	0.3786	deoxynucleotidyltransferase, terminal, interacting protein 2
12698	DIRAS2	-0.3683	0.1011	DIRAS family, GTP-binding RAS-like 2
12699	SNAP47	-0.3688	0.2639	synaptosomal-associated protein, 47kDa
12700	ERVW-1	-0.3688	0.2555	endogenous retrovirus group W, member 1
12701	C16orf73	-0.3688	0.1947	chromosome 16 open reading frame 73
12702	ACAP3	-0.3688	0.2789	ArfGAP with coiled-coil, ankyrin repeat and PH domains 3
12703	TIMM8B	-0.3691	0.3318	translocase of inner mitochondrial membrane 8 homolog B (yeast)
12704	ZNF263	-0.3692	0.2461	zinc finger protein 263
12705	WRN	-0.3692	0.3555	Werner syndrome, RecQ helicase-like
12706	SOX14	-0.3692	0.3637	SRY (sex determining region Y)-box 14
12707	SOX12	-0.3692	0.246	SRY (sex determining region Y)-box 12
12708	RIBC2	-0.3692	0.2162	RIB43A domain with coiled-coils 2
12709	PIM1	-0.3692	0.1382	pim-1 oncogene
12710	PIH1D1	-0.3692	0.2898	PIH1 domain containing 1
12711	KAT8	-0.3692	0.1797	K(lysine) acetyltransferase 8
12712	GTF3A	-0.3692	0.3583	general transcription factor IIIA
12713	USP10	-0.37	0.3229	ubiquitin specific peptidase 10
12714	TULP1	-0.37	0.4029	tubby like protein 1
12715	SOCS7	-0.37	0.1699	suppressor of cytokine signaling 7
12716	PPP2R5E	-0.37	0.3752	protein phosphatase 2, regulatory subunit B', epsilon isoform
12717	NKAIN1	-0.37	0.3649	Na+/K+ transporting ATPase interacting 1
12718	NCL	-0.37	0.3867	nucleolin
12719	MRPL41	-0.37	0.2981	mitochondrial ribosomal protein L41
12720	LYPD6	-0.37	0.1425	LY6/PLAUR domain containing 6
12721	DNAJC7	-0.37	0.3199	DnaJ (Hsp40) homolog, subfamily C, member 7
12722	COQ10A	-0.37	0.1868	coenzyme Q10 homolog A (S. cerevisiae)
12723	C9orf173	-0.37	0.4287	chromosome 9 open reading frame 173
12724	C1orf87	-0.37	0.3247	chromosome 1 open reading frame 87
12725	BTBD10	-0.37	0.334	BTB (POZ) domain containing 10
12726	APOBEC3B	-0.37	0.2607	apolipoprotein B mRNA editing enzyme, catalytic polypeptide-like 3B
12727	ACOT6	-0.37	0.3458	acyl-CoA thioesterase 6
12728	ZBTB39	-0.3708	0.1845	zinc finger and BTB domain containing 39
12729	UTP6	-0.3708	0.3802	UTP6, small subunit (SSU) processome component, homolog (yeast)
12730	OSBP2	-0.3708	0.3783	oxysterol binding protein 2
12731	MMS19	-0.3708	0.2922	MMS19 nucleotide excision repair homolog (S. cerevisiae)
12732	INS	-0.3708	0.3284	insulin
12733	ABCF2	-0.3708	0.2711	ATP-binding cassette, sub-family F (GCN20), member 2
12734	TBC1D10C	-0.3713	0.3328	TBC1 domain family, member 10C
12735	PDE12	-0.3713	0.3592	phosphodiesterase 12
12736	EFR3B	-0.3713	0.1651	EFR3 homolog B (S. cerevisiae)
12737	TRABD	-0.3717	0.3182	TraB domain containing
12738	PSMA3	-0.3717	0.4071	proteasome (prosome, macropain) subunit, alpha type, 3
12739	NTHL1	-0.3717	0.3003	nth endonuclease III-like 1 (E. coli)
12740	COX7A2L	-0.3717	0.3284	cytochrome c oxidase subunit VIIa polypeptide 2 like
12741	API5	-0.3717	0.3735	apoptosis inhibitor 5
12742	VAX2	-0.3725	0.3906	ventral anterior homeobox 2
12743	TAC4	-0.3725	0.3494	tachykinin 4 (hemokinin)
12744	SYCN	-0.3725	0.4121	syncollin
12745	SPNS3	-0.3725	0.3657	spinster homolog 3 (Drosophila)
12746	SCG2	-0.3725	0.109	secretogranin II
12747	RASGEF1B	-0.3725	0.1302	RasGEF domain family, member 1B
12748	PTTG2	-0.3725	0.1423	pituitary tumor-transforming 2
12749	PITPNM1	-0.3725	0.2602	phosphatidylinositol transfer protein, membrane-associated 1
12750	ORAI2	-0.3725	0.1979	ORAI calcium release-activated calcium modulator 2
12751	MNAT1	-0.3725	0.353	menage a trois homolog 1, cyclin H assembly factor (Xenopus laevis)
12752	MIR155HG	-0.3725	0.2281	MIR155 host gene (non-protein coding)
12753	GNB3	-0.3725	0.3772	guanine nucleotide binding protein (G protein), beta polypeptide 3
12754	C8orf76	-0.3725	0.3517	chromosome 8 open reading frame 76
12755	LOC154872	-0.3729	0.3969	uncharacterized protein LOC154872
12756	HSPE1	-0.3729	0.3813	heat shock 10kDa protein 1 (chaperonin 10)
12757	HEATR7B1	-0.3729	0.3985	HEAT repeat containing 7B1
12758	WNT1	-0.3733	0.4128	wingless-type MMTV integration site family, member 1
12759	VASH2	-0.3733	0.1615	vasohibin 2
12760	NUP37	-0.3733	0.4098	nucleoporin 37kDa
12761	LRRC55	-0.3733	0.3416	leucine rich repeat containing 55
12762	JRK	-0.3733	0.3051	jerky homolog (mouse)
12763	ICOSLG	-0.3733	0.4106	inducible T-cell co-stimulator ligand
12764	B4GALT6	-0.3733	0.2351	UDP-Gal:betaGlcNAc beta 1,4- galactosyltransferase, polypeptide 6
12765	ATPIF1	-0.3733	0.3263	ATPase inhibitory factor 1
12766	TMEM69	-0.3738	0.3481	transmembrane protein 69

12767	RNF175	-0.3738	0.1078	ring finger protein 175
12768	FAM160A1	-0.3738	0.2963	family with sequence similarity 160, member A1
12769	TIMM9	-0.3742	0.3519	translocase of inner mitochondrial membrane 9 homolog (yeast)
12770	ST3GAL2	-0.3742	0.2031	ST3 beta-galactoside alpha-2,3-sialyltransferase 2
12771	SERPINI1	-0.3742	0.2371	serpin peptidase inhibitor, clade I (neuroserpin), member 1
12772	PSMD13	-0.3742	0.3334	proteasome (prosome, macropain) 26S subunit, non-ATPase, 13
12773	PRPF38B	-0.3742	0.3895	PRP38 pre-mRNA processing factor 38 (yeast) domain containing B
12774	MIS12	-0.3742	0.3874	MIS12, MIND kinetochore complex component, homolog (S. pombe)
12775	ESR2	-0.3742	0.395	estrogen receptor 2 (ER beta)
12776	CTBP1	-0.3742	0.2854	C-terminal binding protein 1
12777	CBLN1	-0.3742	0.2912	cerebellin 1 precursor
12778	HMHA1	-0.3745	0.2693	histocompatibility (minor) HA-1
12779	U2AF1L4	-0.375	0.1436	U2 small nuclear RNA auxiliary factor 1-like 4
12780	TOMM70A	-0.375	0.3935	translocase of outer mitochondrial membrane 70 homolog A (S. cerevisiae)
12781	TMSB15A	-0.375	0.1746	thymosin beta 15a
12782	NELL1	-0.375	0.2829	NEL-like 1 (chicken)
12783	NDUFB3	-0.375	0.3562	NADH dehydrogenase (ubiquinone) 1 beta subcomplex, 3, 12kDa
12784	LRRIQ3	-0.375	0.1662	leucine-rich repeats and IQ motif containing 3
12785	ELP4	-0.375	0.3133	elongation protein 4 homolog (S. cerevisiae)
12786	DDX51	-0.375	0.2612	DEAD (Asp-Glu-Ala-Asp) box polypeptide 51
12787	CNGB1	-0.375	0.4583	cyclic nucleotide gated channel beta 1
12788	UBE2L6	-0.3758	0.2522	ubiquitin-conjugating enzyme E2L 6
12789	PCDH8	-0.3758	0.1169	protocadherin 8
12790	PAQR3	-0.3758	0.3887	progestin and adipoQ receptor family member III
12791	NUDT18	-0.3758	0.2484	nudix (nucleoside diphosphate linked moiety X)-type motif 18
12792	MUS81	-0.3758	0.237	MUS81 endonuclease homolog (S. cerevisiae)
12793	MRPL15	-0.3758	0.3841	mitochondrial ribosomal protein L15
12794	KIAA0664	-0.3758	0.284	KIAA0664
12795	HES2	-0.3758	0.3913	hairy and enhancer of split 2 (Drosophila)
12796	GUCY2F	-0.3758	0.3751	guanylate cyclase 2F, retinal
12797	GPR37L1	-0.3758	0.4235	G protein-coupled receptor 37 like 1
12798	CIR1	-0.3758	0.2802	corepressor interacting with RBPJ, 1
12799	AIMP2	-0.3758	0.354	aminoacyl tRNA synthetase complex-interacting multifunctional protein 2
12800	UGT3A1	-0.3762	0.3804	UDP glycosyltransferase 3 family, polypeptide A1
12801	PATL1	-0.3762	0.2353	protein associated with topoisomerase II homolog 1 (yeast)
12802	MDGA1	-0.3762	0.4036	MAM domain containing glycosylphosphatidylinositol anchor 1
12803	LEMD1	-0.3762	0.127	LEM domain containing 1
12804	KCNIP4	-0.3762	0.2655	Kv channel interacting protein 4
12805	IL1F10	-0.3762	0.4297	interleukin 1 family, member 10 (theta)
12806	FGD3	-0.3762	0.2506	FYVE, RhoGEF and PH domain containing 3
12807	CCDC43	-0.3762	0.3585	coiled-coil domain containing 43
12808	C11orf88	-0.3762	0.2793	chromosome 11 open reading frame 88
12809	ARL5B	-0.3762	0.3247	ADP-ribosylation factor-like 5B
12810	RAMP1	-0.3767	0.2181	receptor (G protein-coupled) activity modifying protein 1
12811	IFNAR2	-0.3767	0.1352	interferon (alpha, beta and omega) receptor 2
12812	GEMIN4	-0.3767	0.2973	gem (nuclear organelle) associated protein 4
12813	LOC285205	-0.3771	0.9999	uncharacterized LOC285205
12814	ARL9	-0.3771	0.167	ADP-ribosylation factor-like 9
12815	USP28	-0.3775	0.2781	ubiquitin specific peptidase 28
12816	TSSK1B	-0.3775	0.4097	testis-specific serine kinase 1B
12817	TFR2	-0.3775	0.3139	transferrin receptor 2
12818	MPI	-0.3775	0.2458	mannose phosphate isomerase
12819	IGSF8	-0.3775	0.2547	immunoglobulin superfamily, member 8
12820	GATAD2A	-0.3775	0.2346	GATA zinc finger domain containing 2A
12821	DTD1	-0.3775	0.27	D-tyrosyl-tRNA deacylase 1 homolog (S. cerevisiae)
12822	CD300C	-0.3775	0.3375	CD300c molecule
12823	C1orf233	-0.3775	0.2969	chromosome 1 open reading frame 233
12824	BEX2	-0.3775	0.197	brain expressed X-linked 2
12825	SYN3	-0.3783	0.3846	synapsin III
12826	SSB	-0.3783	0.3948	Sjogren syndrome antigen B (autoantigen La)
12827	HIVEP1	-0.3783	0.3103	human immunodeficiency virus type I enhancer binding protein 1
12828	CCNK	-0.3783	0.1238	cyclin K
12829	BORA	-0.3783	0.3663	bora, aurora kinase A activator
12830	BCL7A	-0.3783	0.1641	B-cell CLL/lymphoma 7A
12831	ANKRD26	-0.3783	0.2623	ankyrin repeat domain 26
12832	TOMM40	-0.3786	0.3242	translocase of outer mitochondrial membrane 40 homolog (yeast)
12833	PATE2	-0.3786	0.4024	prostate and testis expressed 2
12834	LOC340017	-0.3786	0.9996	uncharacterized LOC340017
12835	FAM171A2	-0.3786	0.4148	family with sequence similarity 171, member A2
12836	ZFHX4-AS1	-0.3787	0.1749	ZFHX4 antisense RNA 1 (non-protein coding)
12837	ZBTB37	-0.3787	0.1674	zinc finger and BTB domain containing 37
12838	SLC25A18	-0.3787	0.216	solute carrier family 25 (glutamate carrier), member 18
12839	MORN5	-0.3787	0.3612	MORN repeat containing 5
12840	WAS	-0.3792	0.389	Wiskott-Aldrich syndrome
12841	SLC25A28	-0.3792	0.1886	solute carrier family 25 (mitochondrial iron transporter), member 28
12842	RBM12	-0.3792	0.3936	RNA binding motif protein 12
12843	MAPK11	-0.3792	0.3993	mitogen-activated protein kinase 11
12844	KIAA0232	-0.3792	0.3336	KIAA0232
12845	EN2	-0.3792	0.3294	engrailed homeobox 2
12846	EMID1	-0.3792	0.4031	EMI domain containing 1
12847	DPM2	-0.3792	0.258	dolichyl-phosphate mannosyltransferase polypeptide 2, regulatory subunit
12848	CRLF3	-0.3792	0.3562	cytokine receptor-like factor 3
12849	ZNF668	-0.38	0.252	zinc finger protein 668
12850	UROD	-0.38	0.2905	uroporphyrinogen decarboxylase

12851	TNKS	-0.38	0.2867	tankyrase, TRF1-interacting ankyrin-related ADP-ribose polymerase
12852	REPIN1	-0.38	0.204	replication initiator 1
12853	PCDHA10	-0.38	0.3681	protocadherin alpha 10
12854	LY6G5C	-0.38	0.1653	lymphocyte antigen 6 complex, locus G5C
12855	LOC728175	-0.38	0.3724	uncharacterized LOC728175
12856	IL28A	-0.38	0.4331	interleukin 28A (interferon, lambda 2)
12857	DUSP16	-0.38	0.101	dual specificity phosphatase 16
12858	DEFB132	-0.38	0.4189	defensin, beta 132
12859	CDH18	-0.38	0.322	cadherin 18, type 2
12860	C3orf71	-0.38	0.1962	chromosome 3 open reading frame 71
12861	C21orf90	-0.38	0.3991	chromosome 21 open reading frame 90
12862	SEC14L5	-0.3808	0.2823	SEC14-like 5 (S. cerevisiae)
12863	OR10J1	-0.3808	0.4071	olfactory receptor, family 10, subfamily J, member 1
12864	NACA2	-0.3808	0.1636	nascent polypeptide-associated complex alpha subunit 2
12865	ECT2	-0.3808	0.3968	epithelial cell transforming sequence 2 oncogene
12866	CCDC25	-0.3808	0.3166	coiled-coil domain containing 25
12867	BSDC1	-0.3808	0.2316	BSD domain containing 1
12868	ZNF784	-0.3812	0.3392	zinc finger protein 784
12869	TSSK6	-0.3812	0.3672	testis-specific serine kinase 6
12870	TIMM21	-0.3812	0.3779	translocase of inner mitochondrial membrane 21 homolog (yeast)
12871	SFR1	-0.3812	0.352	SWI5-dependent recombination repair 1
12872	NKAP	-0.3812	0.331	NFKB activating protein
12873	LY86-AS1	-0.3812	0.3247	LY86 antisense RNA 1 (non-protein coding)
12874	CCDC105	-0.3812	0.4493	coiled-coil domain containing 105
12875	TIFAB	-0.3814	0.3368	TRAF-interacting protein with forkhead-associated domain, family member B
12876	PLEK	-0.3817	0.253	pleckstrin
12877	OTOF	-0.3817	0.4295	otoferlin
12878	MAMLD1	-0.3817	0.1342	mastermind-like domain containing 1
12879	FOXE3	-0.3817	0.4112	forkhead box E3
12880	CECR1	-0.3817	0.2016	cat eye syndrome chromosome region, candidate 1
12881	ARL8B	-0.3817	0.3694	ADP-ribosylation factor-like 8B
12882	ANKRD46	-0.3817	0.3611	ankyrin repeat domain 46
12883	SHANK3	-0.3825	0.256	SH3 and multiple ankyrin repeat domains 3
12884	SH3TC1	-0.3825	0.2065	SH3 domain and tetratricopeptide repeats 1
12885	RFPL2	-0.3825	0.1197	ret finger protein-like 2
12886	PRL	-0.3825	0.3244	prolactin
12887	NRM	-0.3825	0.2186	nurim (nuclear envelope membrane protein)
12888	MRPS36	-0.3825	0.3189	mitochondrial ribosomal protein S36
12889	KRT71	-0.3825	0.4149	keratin 71
12890	HMGB3P1	-0.3825	0.222	high mobility group box 3 pseudogene 1
12891	GH1	-0.3825	0.4121	growth hormone 1
12892	DDX1	-0.3825	0.3892	DEAD (Asp-Glu-Ala-Asp) box helicase 1
12893	CRTC1	-0.3825	0.4224	CREB regulated transcription coactivator 1
12894	CDK8	-0.3825	0.2713	cyclin-dependent kinase 8
12895	CD1D	-0.3825	0.1599	CD1d molecule
12896	AP1B1	-0.3825	0.2287	adaptor-related protein complex 1, beta 1 subunit
12897	ABR	-0.3825	0.1692	active BCR-related
12898	SNORA74A	-0.3829	0.2492	small nucleolar RNA, H/ACA box 74A
12899	LINC00028	-0.3829	0.4685	long intergenic non-protein coding RNA 28
12900	DEFB124	-0.3829	0.4346	defensin, beta 124
12901	UBXN2B	-0.3833	0.3484	UBX domain protein 2B
12902	TAB1	-0.3833	0.2635	TGF-beta activated kinase 1/MAP3K7 binding protein 1
12903	SHCBP1L	-0.3833	0.3519	SHC SH2-domain binding protein 1-like
12904	PLSCR2	-0.3833	0.3052	phospholipid scramblase 2
12905	HUWE1	-0.3833	0.1503	HECT, UBA and WWE domain containing 1, E3 ubiquitin protein ligase
12906	HTR5A	-0.3833	0.4262	5-hydroxytryptamine (serotonin) receptor 5A, G protein-coupled
12907	GSK3B	-0.3833	0.2556	glycogen synthase kinase 3 beta
12908	DPM1	-0.3833	0.4037	dolichyl-phosphate mannosyltransferase polypeptide 1, catalytic subunit
12909	CST7	-0.3833	0.2782	cystatin F (leukocystatin)
12910	ARID1A	-0.3833	0.2256	AT rich interactive domain 1A (SWI-like)
12911	ARHGEF4	-0.3833	0.261	Rho guanine nucleotide exchange factor (GEF) 4
12912	ABCC8	-0.3833	0.3655	ATP-binding cassette, sub-family C (CFTR/MRP), member 8
12913	TMEM41A	-0.3837	0.205	transmembrane protein 41A
12914	GRIN3B	-0.3837	0.3783	glutamate receptor, ionotropic, N-methyl-D-aspartate 3B
12915	GARNL3	-0.3837	0.1528	GTPase activating Rap/RanGAP domain-like 3
12916	CHST6	-0.3837	0.1731	carbohydrate (N-acetylglucosamine 6-O) sulfotransferase 6
12917	ATPAF1-AS1	-0.3837	0.4118	ATPAF1 antisense RNA 1 (non-protein coding)
12918	RNASE2	-0.3842	0.2051	ribonuclease, RNase A family, 2 (liver, eosinophil-derived neurotoxin)
12919	PAPD7	-0.3842	0.3118	PAP associated domain containing 7
12920	KIAA0101	-0.3842	0.3603	KIAA0101
12921	GFER	-0.3842	0.2821	growth factor, augmenter of liver regeneration
12922	FABP6	-0.3842	0.2619	fatty acid binding protein 6, ileal
12923	CIITA	-0.3842	0.3146	class II, major histocompatibility complex, transactivator
12924	ZEB1-AS1	-0.3843	0.9999	ZEB1 antisense RNA 1 (non-protein coding)
12925	LOC100289341	-0.3843	0.1088	uncharacterized LOC100289341
12926	ZNF622	-0.385	0.242	zinc finger protein 622
12927	REEP4	-0.385	0.2051	receptor accessory protein 4
12928	MPHOSPH10	-0.385	0.3792	M-phase phosphoprotein 10 (U3 small nucleolar ribonucleoprotein)
12929	MACROD2	-0.385	0.1513	MACRO domain containing 2
12930	KIAA0513	-0.385	0.2008	KIAA0513
12931	IER5	-0.385	0.2413	immediate early response 5
12932	HIST1H3A	-0.385	0.0884	histone cluster 1, H3a
12933	DNTT	-0.385	0.2866	deoxynucleotidyltransferase, terminal
12934	COMMD9	-0.385	0.2481	COMM domain containing 9

12935	CNEP1R1	-0.385	0.3378	CTD nuclear envelope phosphatase 1 regulatory subunit 1
12936	LOC202781	-0.3857	0.3453	uncharacterized LOC202781
12937	IRGM	-0.3857	0.0351	immunity-related GTPase family, M
12938	DCDC2B	-0.3857	0.4225	doublecortin domain containing 2B
12939	SUV39H2	-0.3858	0.3352	suppressor of variegation 3-9 homolog 2 (Drosophila)
12940	SUPT7L	-0.3858	0.3379	suppressor of Ty 7 (S. cerevisiae)-like
12941	NUP214	-0.3858	0.1712	nucleoporin 214kDa
12942	DNAAF2	-0.3858	0.3881	dynein, axonemal, assembly factor 2
12943	CRYGD	-0.3858	0.3538	crystallin, gamma D
12944	ALAS1	-0.3858	0.2767	aminolevulinate, delta-, synthase 1
12945	RBM45	-0.3862	0.3254	RNA binding motif protein 45
12946	ANKRD52	-0.3862	0.1679	ankyrin repeat domain 52
12947	ADARB2-AS1	-0.3862	0.3503	ADARB2 antisense RNA 1 (non-protein coding)
12948	ABCC13	-0.3862	0.3562	ATP-binding cassette, sub-family C (CFTR/MRP), member 13, pseudogene
12949	WDR45L	-0.3867	0.3052	WDR45-like
12950	UBAP1	-0.3867	0.1566	ubiquitin associated protein 1
12951	TRIAP1	-0.3867	0.3422	TP53 regulated inhibitor of apoptosis 1
12952	SDHB	-0.3867	0.3541	succinate dehydrogenase complex, subunit B, iron sulfur (Ip)
12953	MSL3	-0.3867	0.1705	male-specific lethal 3 homolog (Drosophila)
12954	KLHL25	-0.3867	0.3047	kelch-like 25 (Drosophila)
12955	KIF1B	-0.3867	0.2499	kinesin family member 1B
12956	HOXD1	-0.3867	0.2614	homeobox D1
12957	FAIM3	-0.3867	0.2147	Fas apoptotic inhibitory molecule 3
12958	CD38	-0.3867	0.1547	CD38 molecule
12959	CD22	-0.3867	0.3245	CD22 molecule
12960	AKR1B1	-0.3867	0.2641	aldo-keto reductase family 1, member B1 (aldose reductase)
12961	WIPF3	-0.3871	0.3537	WAS/WASL interacting protein family, member 3
12962	LOC730098	-0.3871	0.3775	uncharacterized LOC730098
12963	EML6	-0.3871	0.1049	echinoderm microtubule associated protein like 6
12964	ZNF547	-0.3875	0.1321	zinc finger protein 547
12965	WTAP	-0.3875	0.3564	Wilms tumor 1 associated protein
12966	TTLL10	-0.3875	0.4596	tubulin tyrosine ligase-like family, member 10
12967	PRR14L	-0.3875	0.1667	proline rich 14-like
12968	NEUROG3	-0.3875	0.3891	neurogenin 3
12969	LRRC8D	-0.3875	0.2908	leucine rich repeat containing 8 family, member D
12970	KRT72	-0.3875	0.3741	keratin 72
12971	INPP5B	-0.3875	0.1222	inositol polyphosphate-5-phosphatase, 75kDa
12972	FLJ13224	-0.3875	0.3348	uncharacterized LOC79857
12973	FEZF2	-0.3875	0.3775	FEZ family zinc finger 2
12974	CHRNE	-0.3875	0.3965	cholinergic receptor, nicotinic, epsilon (muscle)
12975	ATP5I	-0.3875	0.2731	ATP synthase, H+ transporting, mitochondrial Fo complex, subunit E
12976	MBOAT7	-0.3883	0.236	membrane bound O-acyltransferase domain containing 7
12977	IPCEF1	-0.3883	0.154	interaction protein for cytohesin exchange factors 1
12978	GH2	-0.3883	0.4206	growth hormone 2
12979	EP400	-0.3883	0.2084	E1A binding protein p400
12980	CCDC69	-0.3883	0.1639	coiled-coil domain containing 69
12981	LOC388948	-0.3886	0.9996	uncharacterized LOC388948
12982	ZNF567	-0.3887	0.3546	zinc finger protein 567
12983	FGD2	-0.3887	0.3452	FYVE, RhoGEF and PH domain containing 2
12984	BANF2	-0.3887	0.4046	barrier to autointegration factor 2
12985	ANKRD65	-0.3887	0.3521	ankyrin repeat domain 65
12986	TMEM14A	-0.3892	0.3574	transmembrane protein 14A
12987	PSG3	-0.3892	0.2653	pregnancy specific beta-1-glycoprotein 3
12988	PCDH17	-0.3892	0.2011	protocadherin 17
12989	HSPA9	-0.3892	0.3787	heat shock 70kDa protein 9 (mortalin)
12990	DEFB126	-0.3892	0.1719	defensin, beta 126
12991	ZNF620	-0.39	0.1082	zinc finger protein 620
12992	ZNF335	-0.39	0.3212	zinc finger protein 335
12993	WDR49	-0.39	0.1116	WD repeat domain 49
12994	TEKT2	-0.39	0.3105	tektin 2 (testicular)
12995	SLC45A1	-0.39	0.3144	solute carrier family 45, member 1
12996	RNF113A	-0.39	0.2527	ring finger protein 113A
12997	RLBP1	-0.39	0.4089	retinaldehyde binding protein 1
12998	PRPH	-0.39	0.3903	peripherin
12999	NLGN1	-0.39	0.1185	neuroligin 1
13000	LOC389043	-0.39	0.3108	uncharacterized LOC389043
13001	LINC00294	-0.39	0.9999	long intergenic non-protein coding RNA 294
13002	HRAS	-0.39	0.2902	v-Ha-ras Harvey rat sarcoma viral oncogene homolog
13003	CCDC164	-0.39	0.3775	coiled-coil domain containing 164
13004	C11orf36	-0.39	0.4366	chromosome 11 open reading frame 36
13005	B4GALNT2	-0.39	0.3798	beta-1,4-N-acetyl-galactosaminyl transferase 2
13006	ABTB1	-0.39	0.3105	ankyrin repeat and BTB (POZ) domain containing 1
13007	ST6GALNAC5	-0.3908	0.1135	ST6 (alpha-N-acetyl-neuraminyl-2,3-beta-galactosyl-1,3)-N-acetylgalactosaminide alpha-2,6-sialyltransferase 5
13008	KCNC1	-0.3908	0.373	potassium voltage-gated channel, Shaw-related subfamily, member 1
13009	FKRP	-0.3908	0.1727	fukutin related protein
13010	SDK1	-0.3913	0.1318	sidekick cell adhesion molecule 1
13011	PIGU	-0.3913	0.2796	phosphatidylinositol glycan anchor biosynthesis, class U
13012	NSUN2	-0.3913	0.338	NOP2/Sun RNA methyltransferase family, member 2
13013	NAPSA	-0.3913	0.3696	napsin A aspartic peptidase
13014	KLHL10	-0.3913	0.2398	kelch-like 10 (Drosophila)
13015	HTR3C	-0.3913	0.4006	5-hydroxytryptamine (serotonin) receptor 3C, ionotropic
13016	HOMEZ	-0.3913	0.0431	homeobox and leucine zipper encoding
13017	HACE1	-0.3913	0.3518	HECT domain and ankyrin repeat containing E3 ubiquitin protein ligase 1
13018	H1FOO	-0.3913	0.4338	H1 histone family, member O, oocyte-specific

13019	CLVS1	-0.3913	0.2711	clavesin 1
13020	C15orf26	-0.3913	0.0948	chromosome 15 open reading frame 26
13021	DUS2L	-0.3917	0.1882	dihydrouridine synthase 2-like, SMM1 homolog (S. cerevisiae)
13022	DKK4	-0.3917	0.3463	dickkopf homolog 4 (Xenopus laevis)
13023	CETN1	-0.3917	0.4056	centrin, EF-hand protein, 1
13024	ARHGDIA	-0.3917	0.2769	Rho GDP dissociation inhibitor (GDI) alpha
13025	AMDHD2	-0.3917	0.2992	amidohydrolase domain containing 2
13026	SHQ1	-0.3925	0.3289	SHQ1 homolog (S. cerevisiae)
13027	SH3RF3	-0.3925	0.1782	SH3 domain containing ring finger 3
13028	RP1	-0.3925	0.1268	retinitis pigmentosa 1 (autosomal dominant)
13029	MCF2L2	-0.3925	0.0746	MCF.2 cell line derived transforming sequence-like 2
13030	HIST1H2BO	-0.3925	0.081	histone cluster 1, H2bo
13031	GNG5	-0.3925	0.2367	guanine nucleotide binding protein (G protein), gamma 5
13032	DOCK3	-0.3925	0.1322	dedicator of cytokinesis 3
13033	DMWD	-0.3925	0.3921	dystrophia myotonica, WD repeat containing
13034	DKFZp779M0652	-0.3925	0.4396	uncharacterized DKFZp779M0652
13035	CRIPT	-0.3925	0.3581	cysteine-rich PDZ-binding protein
13036	CHAMP1	-0.3925	0.313	chromosome alignment maintaining phosphoprotein 1
13037	CEND1	-0.3925	0.443	cell cycle exit and neuronal differentiation 1
13038	C2orf16	-0.3925	0.4109	chromosome 2 open reading frame 16
13039	C19orf77	-0.3925	0.2295	chromosome 19 open reading frame 77
13040	ARMC3	-0.3925	0.2387	armadillo repeat containing 3
13041	DISC1	-0.3927	0.2063	disrupted in schizophrenia 1
13042	FLJ45983	-0.3929	0.4139	uncharacterized LOC399717
13043	CLRN1-AS1	-0.3929	0.9999	CLRN1 antisense RNA 1 (non-protein coding)
13044	UQCRC1	-0.3933	0.3099	ubiquinol-cytochrome c reductase core protein I
13045	SRGAP3	-0.3933	0.1755	SLIT-ROBO Rho GTPase activating protein 3
13046	SFTPC	-0.3933	0.4478	surfactant protein C
13047	SERPINI2	-0.3933	0.1382	serpin peptidase inhibitor, clade I (pancpin), member 2
13048	PRPF31	-0.3933	0.321	PRP31 pre-mRNA processing factor 31 homolog (S. cerevisiae)
13049	NUDCD3	-0.3933	0.1696	NudC domain containing 3
13050	MTF1	-0.3933	0.1624	metal-regulatory transcription factor 1
13051	MKL1	-0.3933	0.2723	megakaryoblastic leukemia (translocation) 1
13052	LPIN1	-0.3933	0.1867	lipin 1
13053	DCTN1	-0.3933	0.2468	dynactin 1
13054	CIZ1	-0.3933	0.2598	CDKN1A interacting zinc finger protein 1
13055	ROBO2	-0.3938	0.1225	roundabout, axon guidance receptor, homolog 2 (Drosophila)
13056	MOV10L1	-0.3938	0.3527	Mov10l1, Moloney leukemia virus 10-like 1, homolog (mouse)
13057	LYG1	-0.3938	0.1288	lysozyme G-like 1
13058	FBXO33	-0.3938	0.2896	F-box protein 33
13059	CYB561D1	-0.3938	0.1987	cytochrome b-561 domain containing 1
13060	COL6A5	-0.3938	0.199	collagen, type VI, alpha 5
13061	RTEL1	-0.3942	0.3535	regulator of telomere elongation helicase 1
13062	RBBP6	-0.3942	0.3025	retinoblastoma binding protein 6
13063	OXT	-0.3942	0.422	oxytocin, prepropeptide
13064	MBD1	-0.3942	0.1246	methyl-CpG binding domain protein 1
13065	HRASLS	-0.3942	0.089	HRAS-like suppressor
13066	APOBEC3A	-0.3942	0.1563	apolipoprotein B mRNA editing enzyme, catalytic polypeptide-like 3A
13067	TRIM61	-0.3943	0.345	tripartite motif containing 61
13068	TGM6	-0.3943	0.4672	transglutaminase 6
13069	LOC283914	-0.3943	0.9999	uncharacterized LOC283914
13070	LINC00606	-0.3943	0.9999	long intergenic non-protein coding RNA 606
13071	ZFP91	-0.395	0.3079	zinc finger protein 91 homolog (mouse)
13072	TRIM51	-0.395	0.0721	tripartite motif-containing 51
13073	SNHG7	-0.395	0.204	small nucleolar RNA host gene 7 (non-protein coding)
13074	SH2B2	-0.395	0.2816	SH2B adaptor protein 2
13075	MAP2K5	-0.395	0.1476	mitogen-activated protein kinase kinase 5
13076	FMN2	-0.395	0.1725	formin 2
13077	BANF1	-0.395	0.327	barrier to autointegration factor 1
13078	AGT	-0.395	0.1618	angiotensinogen (serpin peptidase inhibitor, clade A, member 8)
13079	NDUFV2	-0.3957	0.328	NADH dehydrogenase (ubiquinone) flavoprotein 2, 24kDa
13080	FRMPD4	-0.3957	0.3393	FERM and PDZ domain containing 4
13081	TAC3	-0.3958	0.3444	tachykinin 3
13082	RHOBTB2	-0.3958	0.1818	Rho-related BTB domain containing 2
13083	NAALAD2	-0.3958	0.1015	N-acetylated alpha-linked acidic dipeptidase 2
13084	DUSP10	-0.3958	0.1483	dual specificity phosphatase 10
13085	AEN	-0.3958	0.1932	apoptosis enhancing nuclease
13086	SPATA22	-0.3963	0.0923	spermatogenesis associated 22
13087	GPR156	-0.3963	0.3496	G protein-coupled receptor 156
13088	DYDC1	-0.3963	0.0848	DPY30 domain containing 1
13089	C7orf29	-0.3963	0.0886	chromosome 7 open reading frame 29
13090	ANKRD53	-0.3963	0.4131	ankyrin repeat domain 53
13091	IRGQ	-0.3964	0.1361	immunity-related GTPase family, Q
13092	ZC3H15	-0.3967	0.3978	zinc finger CCCH-type containing 15
13093	ZBTB24	-0.3967	0.298	zinc finger and BTB domain containing 24
13094	STXBP5L	-0.3975	0.2167	syntaxin binding protein 5-like
13095	RNF123	-0.3975	0.2015	ring finger protein 123
13096	PRKACB	-0.3975	0.3469	protein kinase, cAMP-dependent, catalytic, beta
13097	NEFL	-0.3975	0.0925	neurofilament, light polypeptide
13098	NAA35	-0.3975	0.3776	N(alpha)-acetyltransferase 35, NatC auxiliary subunit
13099	HTR2C	-0.3975	0.2049	5-hydroxytryptamine (serotonin) receptor 2C, G protein-coupled
13100	HFE2	-0.3975	0.2699	hemochromatosis type 2 (juvenile)
13101	DLX6	-0.3975	0.2996	distal-less homeobox 6
13102	CHCHD6	-0.3975	0.235	coiled-coil-helix-coiled-coil-helix domain containing 6

13103	CCDC88B	-0.3975	0.3747	coiled-coil domain containing 88B
13104	ZNF74	-0.3983	0.204	zinc finger protein 74
13105	PRCC	-0.3983	0.2858	papillary renal cell carcinoma (translocation-associated)
13106	LIN7A	-0.3983	0.0612	lin-7 homolog A (C. elegans)
13107	LETM1	-0.3983	0.2133	leucine zipper-EF-hand containing transmembrane protein 1
13108	GREB1	-0.3983	0.208	growth regulation by estrogen in breast cancer 1
13109	LINC00313	-0.3986	0.371	long intergenic non-protein coding RNA 313
13110	CCDC152	-0.3986	0.1896	coiled-coil domain containing 152
13111	ZSCAN20	-0.3988	0.1323	zinc finger and SCAN domain containing 20
13112	ZMYND15	-0.3988	0.3529	zinc finger, MYND-type containing 15
13113	TMEM225	-0.3988	0.3265	transmembrane protein 225
13114	NTN5	-0.3988	0.3709	netrin 5
13115	MUC17	-0.3988	0.2959	mucin 17, cell surface associated
13116	CCDC12	-0.3988	0.2176	coiled-coil domain containing 12
13117	C16orf54	-0.3988	0.1911	chromosome 16 open reading frame 54
13118	C12orf45	-0.3988	0.2529	chromosome 12 open reading frame 45
13119	XRCC5	-0.3992	0.392	X-ray repair complementing defective repair in Chinese hamster cells 5 (double-strand-break rejoining)
13120	SON	-0.3992	0.3495	SON DNA binding protein
13121	SIRPG	-0.3992	0.2502	signal-regulatory protein gamma
13122	RBFA	-0.3992	0.2377	ribosome binding factor A (putative)
13123	HOXD12	-0.3992	0.2966	homeobox D12
13124	GSG1	-0.3992	0.3763	germ cell associated 1
13125	AZIN1	-0.3992	0.3821	antizyme inhibitor 1
13126	ZSCAN21	-0.4	0.2393	zinc finger and SCAN domain containing 21
13127	STK35	-0.4	0.2223	serine/threonine kinase 35
13128	SCAI	-0.4	0.2686	suppressor of cancer cell invasion
13129	PKIB	-0.4	0.1288	protein kinase (cAMP-dependent, catalytic) inhibitor beta
13130	PDZD4	-0.4	0.3297	PDZ domain containing 4
13131	MMACHC	-0.4	0.1979	methylmalonic aciduria (cobalamin deficiency) cblC type, with homocystinuria
13132	KCNJ9	-0.4	0.4161	potassium inwardly-rectifying channel, subfamily J, member 9
13133	GPR82	-0.4	0.1161	G protein-coupled receptor 82
13134	FLJ26850	-0.4	0.2846	FLJ26850 protein
13135	DGCR10	-0.4	0.9999	DiGeorge syndrome critical region gene 10 (non-protein coding)
13136	CSNK1A1P1	-0.4	0.2229	casein kinase 1, alpha 1 pseudogene 1
13137	CNTFR	-0.4	0.3751	ciliary neurotrophic factor receptor
13138	CAPN14	-0.4	0.037	calpain 14
13139	C14orf132	-0.4	0.1864	chromosome 14 open reading frame 132
13140	BCL2A1	-0.4	0.2167	BCL2-related protein A1
13141	ADCY8	-0.4	0.3084	adenylate cyclase 8 (brain)
13142	ZNF227	-0.4008	0.3599	zinc finger protein 227
13143	TREX2	-0.4008	0.3614	three prime repair exonuclease 2
13144	TCP11	-0.4008	0.3418	t-complex 11 homolog (mouse)
13145	SFXN1	-0.4008	0.1759	sideroflexin 1
13146	RNF31	-0.4008	0.1954	ring finger protein 31
13147	CELF3	-0.4008	0.413	CUGBP, Elav-like family member 3
13148	BTG3	-0.4008	0.3057	BTG family, member 3
13149	AP4S1	-0.4008	0.1192	adaptor-related protein complex 4, sigma 1 subunit
13150	TUBA4B	-0.4009	0.3171	tubulin, alpha 4b (pseudogene)
13151	TYW5	-0.4013	0.2612	tRNA-yW synthesizing protein 5
13152	IL31RA	-0.4013	0.22	interleukin 31 receptor A
13153	CNKSR3	-0.4013	0.0893	CNKSR family member 3
13154	TRMT61A	-0.4017	0.3765	tRNA methyltransferase 61 homolog A (S. cerevisiae)
13155	TIMM17B	-0.4017	0.263	translocase of inner mitochondrial membrane 17 homolog B (yeast)
13156	OPA3	-0.4017	0.1478	optic atrophy 3 (autosomal recessive, with chorea and spastic paraplegia)
13157	FANCL	-0.4017	0.3796	Fanconi anemia, complementation group L
13158	ZNF326	-0.4025	0.3228	zinc finger protein 326
13159	UHRF1BP1L	-0.4025	0.3336	UHRF1 binding protein 1-like
13160	UCHL5	-0.4025	0.399	ubiquitin carboxyl-terminal hydrolase L5
13161	NETO2	-0.4025	0.2787	neuropilin (NRP) and tolloid (TLL)-like 2
13162	HSPH1	-0.4025	0.3364	heat shock 105kDa/110kDa protein 1
13163	GGA3	-0.4025	0.1754	golgi-associated, gamma adaptin ear containing, ARF binding protein 3
13164	EMID2	-0.4025	0.4198	EMI domain containing 2
13165	DNMT3A	-0.4025	0.1281	DNA (cytosine-5-)-methyltransferase 3 alpha
13166	CACUL1	-0.4025	0.166	CDK2-associated, cullin domain 1
13167	C15orf61	-0.4025	0.2511	chromosome 15 open reading frame 61
13168	ZNF451	-0.4033	0.367	zinc finger protein 451
13169	UFD1L	-0.4033	0.3195	ubiquitin fusion degradation 1 like (yeast)
13170	SDR39U1	-0.4033	0.229	short chain dehydrogenase/reductase family 39U, member 1
13171	RPH3A	-0.4033	0.3243	rabphilin 3A homolog (mouse)
13172	NMNAT2	-0.4033	0.1912	nicotinamide nucleotide adenylyltransferase 2
13173	DUSP26	-0.4033	0.3659	dual specificity phosphatase 26 (putative)
13174	MZT2A	-0.4036	0.2448	mitotic spindle organizing protein 2A
13175	TMEM63B	-0.4038	0.2565	transmembrane protein 63B
13176	TLE3	-0.4042	0.1581	transducin-like enhancer of split 3 (E(sp1) homolog, Drosophila)
13177	PARL	-0.4042	0.265	presenilin associated, rhomboid-like
13178	NOS1AP	-0.4042	0.3609	nitric oxide synthase 1 (neuronal) adaptor protein
13179	MKRN1	-0.4042	0.2699	makorin ring finger protein 1
13180	CREM	-0.4042	0.2526	cAMP responsive element modulator
13181	CEP57	-0.4042	0.3844	centrosomal protein 57kDa
13182	CADM1	-0.4042	0.0825	cell adhesion molecule 1
13183	UROS	-0.405	0.2323	uroporphyrinogen III synthase
13184	TMEM217	-0.405	0.2023	transmembrane protein 217
13185	REEP6	-0.405	0.2232	receptor accessory protein 6
13186	LIMK1	-0.405	0.2062	LIM domain kinase 1

13187	HMGCR	-0.405	0.3496	3-hydroxy-3-methylglutaryl-CoA reductase
13188	HIST1H2BA	-0.405	0.0365	histone cluster 1, H2ba
13189	GNL1	-0.405	0.1561	guanine nucleotide binding protein-like 1
13190	CEACAM4	-0.405	0.3623	carcinoembryonic antigen-related cell adhesion molecule 4
13191	CCDC15	-0.405	0.2216	coiled-coil domain containing 15
13192	C20orf195	-0.405	0.3624	chromosome 20 open reading frame 195
13193	BLMH	-0.405	0.3012	bleomycin hydrolase
13194	BIN2	-0.405	0.2117	bridging integrator 2
13195	BEST4	-0.405	0.2169	bestrophin 4
13196	BAALC	-0.405	0.1137	brain and acute leukemia, cytoplasmic
13197	RAPGEF6	-0.4058	0.3213	Rap guanine nucleotide exchange factor (GEF) 6
13198	PCBP4	-0.4058	0.2028	poly(rC) binding protein 4
13199	MAPKAPK5	-0.4058	0.3559	mitogen-activated protein kinase-activated protein kinase 5
13200	CHFR	-0.4058	0.1817	checkpoint with forkhead and ring finger domains, E3 ubiquitin protein ligase
13201	CBX5	-0.4058	0.2658	chromobox homolog 5
13202	ADORA1	-0.4058	0.3759	adenosine A1 receptor
13203	ADCY2	-0.4058	0.3477	adenylate cyclase 2 (brain)
13204	WFDC9	-0.4063	0.3994	WAP four-disulfide core domain 9
13205	KRT40	-0.4063	0.189	keratin 40
13206	HCG22	-0.4063	0.3064	HLA complex group 22 (non-protein coding)
13207	ENPP7	-0.4063	0.3752	ectonucleotide pyrophosphatase/phosphodiesterase 7
13208	BCORP1	-0.4063	0.1128	BCL6 corepressor pseudogene 1
13209	ALS2CR12	-0.4063	0.1044	amyotrophic lateral sclerosis 2 (juvenile) chromosome region, candidate 12
13210	PSMA4	-0.4067	0.3845	proteasome (prosome, macropain) subunit, alpha type, 4
13211	NPTX2	-0.4067	0.0789	neuronal pentraxin II
13212	CHGA	-0.4067	0.3661	chromogranin A (parathyroid secretory protein 1)
13213	APOBEC4	-0.4071	0.3277	apolipoprotein B mRNA editing enzyme, catalytic polypeptide-like 4 (putative)
13214	UBN1	-0.4075	0.1409	ubinuclein 1
13215	PTPDC1	-0.4075	0.1485	protein tyrosine phosphatase domain containing 1
13216	MSC	-0.4075	0.2126	musculin
13217	LPAR2	-0.4075	0.239	lysophosphatidic acid receptor 2
13218	GNG8	-0.4075	0.2809	guanine nucleotide binding protein (G protein), gamma 8
13219	GAS2L3	-0.4075	0.1453	growth arrest-specific 2 like 3
13220	FAM184A	-0.4075	0.1597	family with sequence similarity 184, member A
13221	CTNNA3	-0.4075	0.3023	catenin (cadherin-associated protein), alpha 3
13222	CCDC96	-0.4075	0.1274	coiled-coil domain containing 96
13223	ASB14	-0.4075	0.2731	ankyrin repeat and SOCS box containing 14
13224	SIGLEC5	-0.4083	0.3375	sialic acid binding Ig-like lectin 5
13225	RUNDC3B	-0.4083	0.1234	RUN domain containing 3B
13226	PSD	-0.4083	0.3605	pleckstrin and Sec7 domain containing
13227	MYBL1	-0.4083	0.2081	v-myb myeloblastosis viral oncogene homolog (avian)-like 1
13228	LTB4R2	-0.4083	0.1902	leukotriene B4 receptor 2
13229	FOXB1	-0.4083	0.3783	forkhead box B1
13230	FKBP1B	-0.4083	0.1141	FK506 binding protein 1B, 12.6 kDa
13231	TTC23L	-0.4087	0.171	tetratricopeptide repeat domain 23-like
13232	SRRM3	-0.4087	0.4203	serine/arginine repetitive matrix 3
13233	PRR12	-0.4087	0.2052	proline rich 12
13234	PCDHB18	-0.4087	0.1047	protocadherin beta 18 pseudogene
13235	SLC38A3	-0.4092	0.3645	solute carrier family 38, member 3
13236	POLG2	-0.4092	0.3025	polymerase (DNA directed), gamma 2, accessory subunit
13237	P2RX2	-0.4092	0.4572	purinergic receptor P2X, ligand-gated ion channel, 2
13238	KIAA0922	-0.4092	0.1562	KIAA0922
13239	ING2	-0.4092	0.3014	inhibitor of growth family, member 2
13240	DUSP21	-0.4092	0.3873	dual specificity phosphatase 21
13241	DOM3Z	-0.4092	0.2068	dom-3 homolog Z (C. elegans)
13242	CA10	-0.4092	0.2292	carbonic anhydrase X
13243	UNC119	-0.41	0.2297	unc-119 homolog (C. elegans)
13244	TAF5L	-0.41	0.2198	TAF5-like RNA polymerase II, p300/CBP-associated factor (PCAF)-associated factor, 65kDa
13245	SRP54	-0.41	0.3694	signal recognition particle 54kDa
13246	PRODH	-0.41	0.2487	proline dehydrogenase (oxidase) 1
13247	MYO18B	-0.41	0.3142	myosin XVIIIB
13248	ICT1	-0.41	0.3359	immature colon carcinoma transcript 1
13249	GUSBP1	-0.41	0.1405	glucuronidase, beta pseudogene 1
13250	COCH	-0.41	0.1042	coagulation factor C homolog, cochlin (Limulus polyphemus)
13251	CLPS	-0.41	0.4165	colipase, pancreatic
13252	CEP95	-0.41	0.3176	centrosomal protein 95kDa
13253	CA8	-0.41	0.0676	carbonic anhydrase VIII
13254	C9orf53	-0.41	0.4006	chromosome 9 open reading frame 53
13255	C10orf55	-0.41	0.1116	chromosome 10 open reading frame 55
13256	ARHGAP36	-0.41	0.1374	Rho GTPase activating protein 36
13257	ANO7	-0.41	0.3964	anoctamin 7
13258	AKNAD1	-0.41	0.1267	AKNA domain containing 1
13259	ADAR	-0.41	0.2742	adenosine deaminase, RNA-specific
13260	ADAMTS13	-0.41	0.4154	ADAM metallopeptidase with thrombospondin type 1 motif, 13
13261	ZNF593	-0.4108	0.2862	zinc finger protein 593
13262	PHGDH	-0.4108	0.1552	phosphoglycerate dehydrogenase
13263	NAA15	-0.4108	0.3758	N(alpha)-acetyltransferase 15, NatA auxiliary subunit
13264	KIAA0195	-0.4108	0.2038	KIAA0195
13265	CXorf27	-0.4108	0.3647	chromosome X open reading frame 27
13266	CPN1	-0.4108	0.3751	carboxypeptidase N, polypeptide 1
13267	CCL22	-0.4108	0.3467	chemokine (C-C motif) ligand 22
13268	ZSWIM1	-0.4112	0.1089	zinc finger, SWIM-type containing 1
13269	RASGEF1A	-0.4112	0.0601	RasGEF domain family, member 1A
13270	PARP10	-0.4112	0.3541	poly (ADP-ribose) polymerase family, member 10

13271	ITIH6	-0.4112	0.422	inter-alpha-trypsin inhibitor heavy chain family, member 6
13272	IGFL2	-0.4112	0.2167	IGF-like family member 2
13273	GPR146	-0.4112	0.1373	G protein-coupled receptor 146
13274	GPR123	-0.4112	0.3631	G protein-coupled receptor 123
13275	EFCAB4B	-0.4112	0.1453	EF-hand calcium binding domain 4B
13276	ATOH7	-0.4112	0.1968	atonal homolog 7 (Drosophila)
13277	RIT2	-0.4117	0.2426	Ras-like without CAAX 2
13278	PHYHIP	-0.4117	0.3425	phytanoyl-CoA 2-hydroxylase interacting protein
13279	ANKRD11	-0.4117	0.1659	ankyrin repeat domain 11
13280	ZCRB1	-0.4125	0.3062	zinc finger CCHC-type and RNA binding motif 1
13281	USP5	-0.4125	0.2257	ubiquitin specific peptidase 5 (isopeptidase T)
13282	TMEM59L	-0.4125	0.3909	transmembrane protein 59-like
13283	SLC22A24	-0.4125	0.2127	solute carrier family 22, member 24
13284	PSPC1	-0.4125	0.342	paraspeckle component 1
13285	PPP6R2	-0.4125	0.2064	protein phosphatase 6, regulatory subunit 2
13286	MYO1H	-0.4125	0.1326	myosin IH
13287	FAM76B	-0.4125	0.3594	family with sequence similarity 76, member B
13288	ECE2	-0.4125	0.3611	endothelin converting enzyme 2
13289	DAOA	-0.4125	0.3092	D-amino acid oxidase activator
13290	ABCB9	-0.4125	0.2811	ATP-binding cassette, sub-family B (MDR/TAP), member 9
13291	BAK1	-0.4129	0.1627	BCL2-antagonist/killer 1
13292	TYK2	-0.4133	0.2317	tyrosine kinase 2
13293	SPPL2B	-0.4133	0.3182	signal peptide peptidase like 2B
13294	PSMD3	-0.4133	0.2816	proteasome (prosome, macropain) 26S subunit, non-ATPase, 3
13295	HAT1	-0.4133	0.4106	histone acetyltransferase 1
13296	GPR137	-0.4133	0.3419	G protein-coupled receptor 137
13297	GPM6A	-0.4133	0.124	glycoprotein M6A
13298	DBI	-0.4133	0.3051	diazepam binding inhibitor (GABA receptor modulator, acyl-CoA binding protein)
13299	CDKN1B	-0.4133	0.3398	cyclin-dependent kinase inhibitor 1B (p27, Kip1)
13300	CAPN3	-0.4133	0.1622	calpain 3, (p94)
13301	AARSD1	-0.4133	0.2003	alanyl-tRNA synthetase domain containing 1
13302	WDR87	-0.4137	0.3832	WD repeat domain 87
13303	USP49	-0.4137	0.2208	ubiquitin specific peptidase 49
13304	PCSK4	-0.4137	0.3976	proprotein convertase subtilisin/kexin type 4
13305	IGF2BP2	-0.4137	0.0827	insulin-like growth factor 2 mRNA binding protein 2
13306	ATP11C	-0.4137	0.3062	ATPase, class VI, type 11C
13307	ABCA17P	-0.4137	0.1738	ATP-binding cassette, sub-family A (ABC1), member 17, pseudogene
13308	TUT1	-0.4142	0.2684	terminal uridylyl transferase 1, U6 snRNA-specific
13309	TRMT2A	-0.4142	0.2975	tRNA methyltransferase 2 homolog A (S. cerevisiae)
13310	TNP1	-0.4142	0.3717	transition protein 1 (during histone to protamine replacement)
13311	SDK2	-0.4142	0.3583	sidekick cell adhesion molecule 2
13312	CDH4	-0.4142	0.2813	cadherin 4, type 1, R-cadherin (retinal)
13313	RPS5	-0.4143	0.2794	ribosomal protein S5
13314	OR8B2	-0.4143	0.0595	olfactory receptor, family 8, subfamily B, member 2
13315	LRRC10B	-0.4143	0.2396	leucine rich repeat containing 10B
13316	C8orf49	-0.4143	0.1763	chromosome 8 open reading frame 49
13317	TRPM5	-0.415	0.4101	transient receptor potential cation channel, subfamily M, member 5
13318	TPGS2	-0.415	0.2743	tubulin polyglutamylase complex subunit 2
13319	SCNN1D	-0.415	0.3874	sodium channel, non-voltage-gated 1, delta subunit
13320	NT5C1B	-0.415	0.2716	5'-nucleotidase, cytosolic IB
13321	NDUFA11	-0.415	0.2866	NADH dehydrogenase (ubiquinone) 1 alpha subcomplex, 11, 14.7kDa
13322	MICAL1	-0.415	0.2086	microtubule associated monoxygenase, calponin and LIM domain containing 1
13323	KRTAP4-5	-0.415	0.3655	keratin associated protein 4-5
13324	KCNAB2	-0.415	0.1525	potassium voltage-gated channel, shaker-related subfamily, beta member 2
13325	GLTPD2	-0.415	0.3733	glycolipid transfer protein domain containing 2
13326	FKBP6	-0.415	0.3669	FK506 binding protein 6, 36kDa
13327	DNAJB7	-0.415	0.0548	DnaJ (Hsp40) homolog, subfamily B, member 7
13328	CSMD2	-0.415	0.2196	CUB and Sushi multiple domains 2
13329	RAPGEF4-AS1	-0.4157	0.9996	RAPGEF4 antisense RNA 1 (non-protein coding)
13330	RRAGD	-0.4158	0.1435	Ras-related GTP binding D
13331	LTA	-0.4158	0.3481	lymphotoxin alpha (TNF superfamily, member 1)
13332	HAPLN2	-0.4158	0.4083	hyaluronan and proteoglycan link protein 2
13333	EAF2	-0.4158	0.2049	ELL associated factor 2
13334	BLM	-0.4158	0.2885	Bloom syndrome, RecQ helicase-like
13335	ANAPC10	-0.4158	0.385	anaphase promoting complex subunit 10
13336	WDR75	-0.4162	0.3545	WD repeat domain 75
13337	TMEM92	-0.4162	0.3365	transmembrane protein 92
13338	SGK494	-0.4162	0.224	uncharacterized serine/threonine-protein kinase SgK494
13339	PPP1R12C	-0.4162	0.2752	protein phosphatase 1, regulatory subunit 12C
13340	NLRP12	-0.4162	0.3389	NLR family, pyrin domain containing 12
13341	MAP6	-0.4162	0.329	microtubule-associated protein 6
13342	YY1AP1	-0.4167	0.2352	YY1 associated protein 1
13343	YLPM1	-0.4167	0.3251	YLP motif containing 1
13344	STARD3	-0.4167	0.2187	StAR-related lipid transfer (START) domain containing 3
13345	PPP1R2P9	-0.4167	0.4	protein phosphatase 1, regulatory (inhibitor) subunit 2 pseudogene 9
13346	MTO1	-0.4167	0.373	mitochondrial translation optimization 1 homolog (S. cerevisiae)
13347	LLGL1	-0.4167	0.3076	lethal giant larvae homolog 1 (Drosophila)
13348	IPO13	-0.4167	0.1873	importin 13
13349	FUZ	-0.4167	0.2485	fuzzy homolog (Drosophila)
13350	COG4	-0.4167	0.1953	component of oligomeric golgi complex 4
13351	CABP1	-0.4167	0.3812	calcium binding protein 1
13352	ANKRD55	-0.4167	0.1377	ankyrin repeat domain 55
13353	UBE2E3	-0.4171	0.3104	ubiquitin-conjugating enzyme E2E 3
13354	OR1J4	-0.4171	0.2954	olfactory receptor, family 1, subfamily J, member 4

13355	NRG3	-0.4171	0.1127	neuregulin 3
13356	LOC157381	-0.4171	0.9999	uncharacterized LOC157381
13357	LOC150577	-0.4171	0.9999	uncharacterized LOC150577
13358	SLU7	-0.4175	0.3395	SLU7 splicing factor homolog (S. cerevisiae)
13359	SENP3	-0.4175	0.1858	SUMO1/sentrin/SMT3 specific peptidase 3
13360	SCN2B	-0.4175	0.4029	sodium channel, voltage-gated, type II, beta subunit
13361	PLA2G2E	-0.4175	0.3913	phospholipase A2, group IIE
13362	NOP16	-0.4175	0.3505	NOP16 nucleolar protein homolog (yeast)
13363	LBR	-0.4175	0.3612	lamin B receptor
13364	KIFAP3	-0.4175	0.3139	kinesin-associated protein 3
13365	KIAA1045	-0.4175	0.3837	KIAA1045
13366	HINT2	-0.4175	0.2226	histidine triad nucleotide binding protein 2
13367	G3BP2	-0.4175	0.371	GTPase activating protein (SH3 domain) binding protein 2
13368	FLJ33360	-0.4175	0.2734	FLJ33360 protein
13369	FBXL15	-0.4175	0.2529	F-box and leucine-rich repeat protein 15
13370	C9orf47	-0.4175	0.2828	chromosome 9 open reading frame 47
13371	ARHGAP4	-0.4175	0.2657	Rho GTPase activating protein 4
13372	USP33	-0.4183	0.366	ubiquitin specific peptidase 33
13373	SPTBN2	-0.4183	0.2757	spectrin, beta, non-erythrocytic 2
13374	POLR1C	-0.4183	0.2955	polymerase (RNA) I polypeptide C, 30kDa
13375	HNRNPA1	-0.4183	0.3488	heterogeneous nuclear ribonucleoprotein A1
13376	HIGD1A	-0.4183	0.3554	HIG1 hypoxia inducible domain family, member 1A
13377	EEF1A2	-0.4183	0.1363	eukaryotic translation elongation factor 1 alpha 2
13378	DDX50	-0.4183	0.3493	DEAD (Asp-Glu-Ala-Asp) box polypeptide 50
13379	AFF2	-0.4183	0.3535	AF4/FMR2 family, member 2
13380	CBX1	-0.4186	0.3572	chromobox homolog 1
13381	ZNF398	-0.4187	0.2204	zinc finger protein 398
13382	TMEM95	-0.4187	0.4345	transmembrane protein 95
13383	ISL2	-0.4187	0.1222	ISL LIM homeobox 2
13384	GTPBP10	-0.4187	0.3188	GTP-binding protein 10 (putative)
13385	MARK3	-0.4192	0.3066	MAP/microtubule affinity-regulating kinase 3
13386	GTF2H4	-0.4192	0.2544	general transcription factor IIH, polypeptide 4, 52kDa
13387	FOXRED2	-0.4192	0.1798	FAD-dependent oxidoreductase domain containing 2
13388	ZNF202	-0.42	0.2033	zinc finger protein 202
13389	SLC35G3	-0.42	0.0432	solute carrier family 35, member G3
13390	RBM42	-0.42	0.2661	RNA binding motif protein 42
13391	PPAPDC1A	-0.42	0.1172	phosphatidic acid phosphatase type 2 domain containing 1A
13392	INTS4	-0.42	0.233	integrator complex subunit 4
13393	FXR2	-0.42	0.1909	fragile X mental retardation, autosomal homolog 2
13394	FBRS	-0.42	0.3212	fibrosin
13395	FAM81A	-0.42	0.103	family with sequence similarity 81, member A
13396	C14orf166B	-0.42	0.4145	chromosome 14 open reading frame 166B
13397	RBM6	-0.4208	0.2613	RNA binding motif protein 6
13398	NME7	-0.4208	0.3424	NME/NM23 family member 7
13399	CSTF3	-0.4208	0.3509	cleavage stimulation factor, 3' pre-RNA, subunit 3, 77kDa
13400	CDKL3	-0.4208	0.1696	cyclin-dependent kinase-like 3
13401	SPRN	-0.4212	0.4282	shadow of prion protein homolog (zebrafish)
13402	P2RY12	-0.4212	0.1226	purinergic receptor P2Y, G-protein coupled, 12
13403	LEAP2	-0.4212	0.1498	liver expressed antimicrobial peptide 2
13404	KIAA1239	-0.4212	0.1349	KIAA1239
13405	UBXN1	-0.4217	0.2108	UBX domain protein 1
13406	SOCS1	-0.4217	0.2101	suppressor of cytokine signaling 1
13407	LRRC3	-0.4217	0.332	leucine rich repeat containing 3
13408	GRK4	-0.4217	0.1648	G protein-coupled receptor kinase 4
13409	DSN1	-0.4217	0.316	DSN1, MIND kinetochore complex component, homolog (S. cerevisiae)
13410	CBFA2T2	-0.4217	0.1609	core-binding factor, runt domain, alpha subunit 2; translocated to, 2
13411	WDR54	-0.4225	0.1969	WD repeat domain 54
13412	TTYH2	-0.4225	0.0946	tweety homolog 2 (Drosophila)
13413	TSNAXIP1	-0.4225	0.3399	translin-associated factor X interacting protein 1
13414	TOMM34	-0.4225	0.1915	translocase of outer mitochondrial membrane 34
13415	TAF6	-0.4225	0.178	TAF6 RNA polymerase II, TATA box binding protein (TBP)-associated factor, 80kDa
13416	SLC9A6	-0.4225	0.3411	solute carrier family 9, subfamily A (NHE6, cation proton antiporter 6), member 6
13417	RIPPLY2	-0.4225	0.2195	ripply2 homolog (zebrafish)
13418	RFXAP	-0.4225	0.2408	regulatory factor X-associated protein
13419	NOP14	-0.4225	0.2774	NOP14 nucleolar protein homolog (yeast)
13420	LYPD1	-0.4225	0.1016	LY6/PLAUR domain containing 1
13421	LSM1	-0.4225	0.3072	LSM1 homolog, U6 small nuclear RNA associated (S. cerevisiae)
13422	IKZF4	-0.4225	0.1595	IKAROS family zinc finger 4 (Eos)
13423	FAM108B1	-0.4225	0.2579	family with sequence similarity 108, member B1
13424	LOC643923	-0.4229	0.3849	uncharacterized LOC643923
13425	LOC100287177	-0.4229	0.3605	uncharacterized LOC100287177
13426	VPS4A	-0.4233	0.211	vacuolar protein sorting 4 homolog A (S. cerevisiae)
13427	RLN1	-0.4233	0.1068	relaxin 1
13428	LRRTM2	-0.4233	0.153	leucine rich repeat transmembrane neuronal 2
13429	FRG1	-0.4233	0.3423	FSHD region gene 1
13430	FGF17	-0.4233	0.3767	fibroblast growth factor 17
13431	APC	-0.4233	0.2981	adenomatous polyposis coli
13432	TRERF1	-0.4238	0.0902	transcriptional regulating factor 1
13433	SLC9C2	-0.4238	0.08	solute carrier family 9, member C2 (putative)
13434	ODF3	-0.4238	0.4423	outer dense fiber of sperm tails 3
13435	FOXR1	-0.4238	0.3268	forkhead box R1
13436	FAM111B	-0.4238	0.1929	family with sequence similarity 111, member B
13437	C1orf150	-0.4238	0.0331	chromosome 1 open reading frame 150
13438	C17orf51	-0.4238	0.1311	chromosome 17 open reading frame 51

13439	BRPF3	-0.4238	0.0966	bromodomain and PHD finger containing, 3
13440	ASB11	-0.4238	0.2913	ankyrin repeat and SOCS box containing 11
13441	ZAP70	-0.4242	0.2685	zeta-chain (TCR) associated protein kinase 70kDa
13442	PLCG1	-0.4242	0.1802	phospholipase C, gamma 1
13443	PIKFYVE	-0.4242	0.3092	phosphoinositide kinase, FYVE finger containing
13444	NISCH	-0.4242	0.218	nischarin
13445	MOS	-0.4242	0.3723	v-mos Moloney murine sarcoma viral oncogene homolog
13446	KLHL11	-0.4242	0.1382	kelch-like 11 (Drosophila)
13447	ITGAE	-0.4242	0.2794	integrin, alpha E (antigen CD103, human mucosal lymphocyte antigen 1; alpha polypeptide)
13448	HIST1H4H	-0.4242	0.0543	histone cluster 1, H4h
13449	ATP6V1F	-0.4242	0.221	ATPase, H+ transporting, lysosomal 14kDa, V1 subunit F
13450	SNX25	-0.425	0.2224	sorting nexin 25
13451	SLC22A15	-0.425	0.1586	solute carrier family 22, member 15
13452	SETD9	-0.425	0.2979	SET domain containing 9
13453	RRN3	-0.425	0.3726	RRN3 RNA polymerase I transcription factor homolog (S. cerevisiae)
13454	FAM73B	-0.425	0.1947	family with sequence similarity 73, member B
13455	DGCR5	-0.425	0.3687	DiGeorge syndrome critical region gene 5 (non-protein coding)
13456	C7orf31	-0.425	0.156	chromosome 7 open reading frame 31
13457	C16orf5	-0.425	0.1419	chromosome 16 open reading frame 5
13458	AP2A1	-0.425	0.2438	adaptor-related protein complex 2, alpha 1 subunit
13459	LOC100134368	-0.4257	0.9999	uncharacterized LOC100134368
13460	CCDC42B	-0.4257	0.4239	coiled-coil domain containing 42B
13461	ZIC3	-0.4258	0.2352	Zic family member 3
13462	TWF2	-0.4258	0.222	twinfilin, actin-binding protein, homolog 2 (Drosophila)
13463	RASGRP1	-0.4258	0.1381	RAS guanyl releasing protein 1 (calcium and DAG-regulated)
13464	MRPS7	-0.4258	0.3229	mitochondrial ribosomal protein S7
13465	LYZL6	-0.4258	0.3315	lysozyme-like 6
13466	HCN4	-0.4258	0.4205	hyperpolarization activated cyclic nucleotide-gated potassium channel 4
13467	CBLL1	-0.4258	0.3597	Cbl proto-oncogene, E3 ubiquitin protein ligase-like 1
13468	B3GNTL1	-0.4258	0.1437	UDP-GlcNAc:betaGal beta-1,3-N-acetylglucosaminyltransferase-like 1
13469	ATP5A1	-0.4258	0.3518	ATP synthase, H+ transporting, mitochondrial F1 complex, alpha subunit 1, cardiac muscle
13470	ZP4	-0.4263	0.3186	zona pellucida glycoprotein 4
13471	ZBTB34	-0.4263	0.2788	zinc finger and BTB domain containing 34
13472	KRBA1	-0.4263	0.3746	KRAB-A domain containing 1
13473	KLK4	-0.4263	0.4259	kallikrein-related peptidase 4
13474	ISX	-0.4263	0.2883	intestine-specific homeobox
13475	GRASP	-0.4263	0.3349	GRP1 (general receptor for phosphoinositides 1)-associated scaffold protein
13476	FAM169B	-0.4263	0.3189	family with sequence similarity 169, member B
13477	COL22A1	-0.4263	0.2453	collagen, type XXII, alpha 1
13478	CDC42BPG	-0.4263	0.3303	CDC42 binding protein kinase gamma (DMPK-like)
13479	C22orf23	-0.4263	0.2238	chromosome 22 open reading frame 23
13480	SSX5	-0.4267	0.3248	synovial sarcoma, X breakpoint 5
13481	OTUD3	-0.4267	0.1794	OTU domain containing 3
13482	MIOS	-0.4267	0.3415	missing oocyte, meiosis regulator, homolog (Drosophila)
13483	LY86	-0.4267	0.2136	lymphocyte antigen 86
13484	HEATR6	-0.4267	0.1289	HEAT repeat containing 6
13485	GTF2E2	-0.4267	0.3395	general transcription factor IIE, polypeptide 2, beta 34kDa
13486	DNAI1	-0.4267	0.3736	dynein, axonemal, intermediate chain 1
13487	AKAP4	-0.4267	0.3317	A kinase (PRKA) anchor protein 4
13488	UBE2I	-0.4275	0.309	ubiquitin-conjugating enzyme E2I
13489	TSPYL2	-0.4275	0.1755	TSPY-like 2
13490	TSNARE1	-0.4275	0.3244	t-SNARE domain containing 1
13491	RHBDL1	-0.4275	0.3351	rhomboid, veinlet-like 1 (Drosophila)
13492	PHTF1	-0.4275	0.2681	putative homeodomain transcription factor 1
13493	NEU3	-0.4275	0.2662	sialidase 3 (membrane sialidase)
13494	MOBP	-0.4275	0.4041	myelin-associated oligodendrocyte basic protein
13495	FUS	-0.4275	0.25	fused in sarcoma
13496	EIF5	-0.4275	0.3402	eukaryotic translation initiation factor 5
13497	CXorf1	-0.4275	0.193	chromosome X open reading frame 1
13498	COQ5	-0.4275	0.3141	coenzyme Q5 homolog, methyltransferase (S. cerevisiae)
13499	CDAN1	-0.4275	0.1648	codanin 1
13500	RIN3	-0.4283	0.2499	Ras and Rab interactor 3
13501	MLST8	-0.4283	0.2722	MTOR associated protein, LST8 homolog (S. cerevisiae)
13502	ARFGAP1	-0.4283	0.269	ADP-ribosylation factor GTPase activating protein 1
13503	NAT14	-0.4288	0.2202	N-acetyltransferase 14 (GCN5-related, putative)
13504	MOGAT3	-0.4288	0.3561	monoacylglycerol O-acyltransferase 3
13505	CPXCR1	-0.4288	0.0731	CPX chromosome region, candidate 1
13506	SNRPB	-0.4292	0.3288	small nuclear ribonucleoprotein polypeptides B and B1
13507	PKDREJ	-0.4292	0.2536	polycystic kidney disease (polycystin) and REJ homolog (sperm receptor for egg jelly homolog, sea urchin)
13508	NRD1	-0.4292	0.268	nardilysin (N-arginine dibasic convertase)
13509	IGF2BP3	-0.4292	0.1696	insulin-like growth factor 2 mRNA binding protein 3
13510	CD53	-0.4292	0.2381	CD53 molecule
13511	CCDC85B	-0.4292	0.2672	coiled-coil domain containing 85B
13512	C14orf169	-0.4292	0.2283	chromosome 14 open reading frame 169
13513	BRF2	-0.4292	0.1218	BRF2, subunit of RNA polymerase III transcription initiation factor, BRF1-like
13514	AMBN	-0.4292	0.3982	ameloblastin (enamel matrix protein)
13515	ZNF721	-0.43	0.3392	zinc finger protein 721
13516	WDR4	-0.43	0.2229	WD repeat domain 4
13517	WBP4	-0.43	0.3459	WW domain binding protein 4 (formin binding protein 21)
13518	VAT1L	-0.43	0.0998	vesicle amine transport protein 1 homolog (T. californica)-like
13519	TMEM184B	-0.43	0.1559	transmembrane protein 184B
13520	SARNP	-0.43	0.2365	SAP domain containing ribonucleoprotein
13521	RFX2	-0.43	0.2168	regulatory factor X, 2 (influences HLA class II expression)
13522	PANX2	-0.43	0.3885	pannexin 2

13523	OFCC1	-0.43	0.3454	orofacial cleft 1 candidate 1
13524	ODF3L1	-0.43	0.2076	outer dense fiber of sperm tails 3-like 1
13525	METTL21CP1	-0.43	0.2542	methyltransferase like 21C pseudogene 1
13526	LOC400891	-0.43	0.2082	chromosome 14 open reading frame 166B pseudogene
13527	LINC00161	-0.43	0.3378	long intergenic non-protein coding RNA 161
13528	KDM3A	-0.43	0.3252	lysine (K)-specific demethylase 3A
13529	KCP	-0.43	0.4187	kielin/chordin-like protein
13530	FAM96B	-0.43	0.27	family with sequence similarity 96, member B
13531	ENAM	-0.43	0.2811	enamelin
13532	CDH12	-0.43	0.0877	cadherin 12, type 2 (N-cadherin 2)
13533	ATG12	-0.43	0.3191	autophagy related 12
13534	ANKRD34C	-0.43	0.9996	ankyrin repeat domain 34C
13535	AGGF1	-0.43	0.3668	angiogenic factor with G patch and FHA domains 1
13536	LHX2	-0.4308	0.1169	LIM homeobox 2
13537	ITGB2	-0.4308	0.2326	integrin, beta 2 (complement component 3 receptor 3 and 4 subunit)
13538	ALLC	-0.4308	0.3438	allantoicase
13539	ZNF615	-0.4313	0.192	zinc finger protein 615
13540	STARD6	-0.4313	0.0573	StAR-related lipid transfer (START) domain containing 6
13541	KCNC2	-0.4313	0.3089	potassium voltage-gated channel, Shaw-related subfamily, member 2
13542	FCRLA	-0.4313	0.1345	Fc receptor-like A
13543	DNLZ	-0.4313	0.2296	DNL-type zinc finger
13544	ADIG	-0.4313	0.3999	adipogenin
13545	LINC00582	-0.4314	0.9999	long intergenic non-protein coding RNA 582
13546	RGSL1	-0.4317	0.3277	regulator of G-protein signaling like 1
13547	PSMA7	-0.4317	0.3333	proteasome (prosome, macropain) subunit, alpha type, 7
13548	PPIF	-0.4317	0.2398	peptidylprolyl isomerase F
13549	NR2E1	-0.4317	0.3171	nuclear receptor subfamily 2, group E, member 1
13550	MAP2K4	-0.4317	0.2941	mitogen-activated protein kinase kinase 4
13551	DTYMK	-0.4317	0.3106	deoxythymidylate kinase (thymidylate kinase)
13552	DSCAM	-0.4317	0.4057	Down syndrome cell adhesion molecule
13553	DRD2	-0.4317	0.439	dopamine receptor D2
13554	CEP85	-0.4317	0.1575	centrosomal protein 85kDa
13555	C8orf33	-0.4317	0.3134	chromosome 8 open reading frame 33
13556	ATMIN	-0.4317	0.3564	ATM interactor
13557	SF1	-0.4325	0.1904	splicing factor 1
13558	sept-12	-0.4325	0.4048	septin 12
13559	PARVB	-0.4325	0.1579	parvin, beta
13560	OMG	-0.4325	0.2274	oligodendrocyte myelin glycoprotein
13561	MYH7B	-0.4325	0.3092	myosin, heavy chain 7B, cardiac muscle, beta
13562	LOC729121	-0.4325	0.2205	uncharacterized LOC729121
13563	LOC400752	-0.4325	0.2806	uncharacterized LOC400752
13564	HABP4	-0.4325	0.0776	hyaluronan binding protein 4
13565	GCA	-0.4325	0.2881	grancalcin, EF-hand calcium binding protein
13566	FOXD3	-0.4325	0.3179	forkhead box D3
13567	EOMES	-0.4325	0.1251	eomesodermin
13568	CEP192	-0.4325	0.352	centrosomal protein 192kDa
13569	C21orf49	-0.4325	0.1157	chromosome 21 open reading frame 49
13570	C20orf79	-0.4325	0.3464	chromosome 20 open reading frame 79
13571	RPL36AL	-0.4329	0.291	ribosomal protein L36a-like
13572	LCNL1	-0.4329	0.4403	lipocalin-like 1
13573	SSX1	-0.4333	0.0477	synovial sarcoma, X breakpoint 1
13574	DHX57	-0.4333	0.192	DEAH (Asp-Glu-Ala-Asp/His) box polypeptide 57
13575	C22orf28	-0.4333	0.3043	chromosome 22 open reading frame 28
13576	ZNF853	-0.4338	0.1597	zinc finger protein 853
13577	TMEM190	-0.4338	0.3991	transmembrane protein 190
13578	TBCEL	-0.4338	0.2077	tubulin folding cofactor E-like
13579	IZUMO2	-0.4338	0.3367	IZUMO family member 2
13580	DMRTB1	-0.4338	0.4166	DMRT-like family B with proline-rich C-terminal, 1
13581	ZNF512B	-0.4342	0.2074	zinc finger protein 512B
13582	ZNF189	-0.4342	0.3073	zinc finger protein 189
13583	TRIM62	-0.4342	0.2225	tripartite motif containing 62
13584	SPI1	-0.4342	0.3025	spleen focus forming virus (SFFV) proviral integration oncogene spi1
13585	PRM1	-0.4342	0.3794	protamine 1
13586	PDXK	-0.4342	0.1508	pyridoxal (pyridoxine, vitamin B6) kinase
13587	LAIR1	-0.4342	0.2364	leukocyte-associated immunoglobulin-like receptor 1
13588	KCNK13	-0.4342	0.1891	potassium channel, subfamily K, member 13
13589	HNRNPA0	-0.4342	0.311	heterogeneous nuclear ribonucleoprotein A0
13590	DHX38	-0.4342	0.1574	DEAH (Asp-Glu-Ala-His) box polypeptide 38
13591	CD72	-0.4342	0.1587	CD72 molecule
13592	ZNF174	-0.435	0.115	zinc finger protein 174
13593	TMEM170B	-0.435	0.2685	transmembrane protein 170B
13594	SMU1	-0.435	0.1804	smu-1 suppressor of mec-8 and unc-52 homolog (C. elegans)
13595	SCAND1	-0.435	0.2614	SCAN domain containing 1
13596	PSMG2	-0.435	0.347	proteasome (prosome, macropain) assembly chaperone 2
13597	PLD4	-0.435	0.2387	phospholipase D family, member 4
13598	PDZRN4	-0.435	0.1102	PDZ domain containing ring finger 4
13599	OGFR	-0.435	0.3212	opioid growth factor receptor
13600	MYCN	-0.435	0.3154	v-myc myelocytomatosis viral related oncogene, neuroblastoma derived (avian)
13601	LRRC3B	-0.435	0.1588	leucine rich repeat containing 3B
13602	IL12RB2	-0.435	0.0618	interleukin 12 receptor, beta 2
13603	FAM194A	-0.435	0.2537	family with sequence similarity 194, member A
13604	DEFB127	-0.435	0.1003	defensin, beta 127
13605	C8orf47	-0.435	0.0517	chromosome 8 open reading frame 47
13606	C17orf70	-0.435	0.2638	chromosome 17 open reading frame 70

13607	ATP6AP1L	-0.435	0.1197	ATPase, H+ transporting, lysosomal accessory protein 1-like
13608	ARL11	-0.435	0.1052	ADP-ribosylation factor-like 11
13609	ACSBG2	-0.435	0.3328	acyl-CoA synthetase bubblegum family member 2
13610	LOC286135	-0.4357	0.9996	uncharacterized LOC286135
13611	LOC100130503	-0.4357	0.1461	uncharacterized LOC100130503
13612	LOC100129098	-0.4357	0.9999	uncharacterized LOC100129098
13613	TP53BP2	-0.4358	0.2861	tumor protein p53 binding protein, 2
13614	NOL12	-0.4358	0.2156	nucleolar protein 12
13615	CXorf57	-0.4358	0.169	chromosome X open reading frame 57
13616	LOC100133991	-0.4363	0.1903	uncharacterized LOC100133991
13617	NPRL2	-0.4367	0.2427	nitrogen permease regulator-like 2 (S. cerevisiae)
13618	LPHN1	-0.4367	0.1552	latrophilin 1
13619	LILRA5	-0.4367	0.2866	leukocyte immunoglobulin-like receptor, subfamily A (with TM domain), member 5
13620	LHX6	-0.4367	0.2662	LIM homeobox 6
13621	KIF3A	-0.4367	0.2973	kinesin family member 3A
13622	H2AFY	-0.4367	0.2847	H2A histone family, member Y
13623	EIF5B	-0.4367	0.333	eukaryotic translation initiation factor 5B
13624	C19orf60	-0.4367	0.2475	chromosome 19 open reading frame 60
13625	AURKA	-0.4367	0.3175	aurora kinase A
13626	ARHGEF3	-0.4367	0.2253	Rho guanine nucleotide exchange factor (GEF) 3
13627	GIMAP5	-0.4371	0.2056	GTPase, IMAP family member 5
13628	EIF4A1	-0.4371	0.3167	eukaryotic translation initiation factor 4A1
13629	TMEM130	-0.4375	0.2424	transmembrane protein 130
13630	SYT1	-0.4375	0.0937	synaptotagmin I
13631	SIX6	-0.4375	0.3558	SIX homeobox 6
13632	RSRC2	-0.4375	0.3523	arginine/serine-rich coiled-coil 2
13633	PNO1	-0.4375	0.3362	partner of NOB1 homolog (S. cerevisiae)
13634	PKD1	-0.4375	0.3559	polycystic kidney disease 1 (autosomal dominant)
13635	MUTYH	-0.4375	0.2066	mutY homolog (E. coli)
13636	MRPS15	-0.4375	0.298	mitochondrial ribosomal protein S15
13637	IL21R	-0.4375	0.2072	interleukin 21 receptor
13638	HCN2	-0.4375	0.3265	hyperpolarization activated cyclic nucleotide-gated potassium channel 2
13639	GMFG	-0.4375	0.2064	glia maturation factor, gamma
13640	FGF22	-0.4375	0.2438	fibroblast growth factor 22
13641	CHRNA3	-0.4375	0.347	cholinergic receptor, nicotinic, alpha 3 (neuronal)
13642	CD7	-0.4375	0.3303	CD7 molecule
13643	C12orf51	-0.4375	0.2139	chromosome 12 open reading frame 51
13644	C11orf58	-0.4375	0.3771	chromosome 11 open reading frame 58
13645	AGAP2	-0.4375	0.3448	ArfGAP with GTPase domain, ankyrin repeat and PH domain 2
13646	ADAT1	-0.4375	0.2688	adenosine deaminase, tRNA-specific 1
13647	SRP9	-0.4382	0.3879	signal recognition particle 9kDa
13648	DEPDC1	-0.4382	0.2904	DEP domain containing 1
13649	SRSF4	-0.4383	0.2623	serine/arginine-rich splicing factor 4
13650	SPN	-0.4383	0.2736	sialophorin
13651	GPATCH1	-0.4383	0.247	G patch domain containing 1
13652	FBXO7	-0.4383	0.1581	F-box protein 7
13653	DHCR24	-0.4383	0.1587	24-dehydrocholesterol reductase
13654	CLP1	-0.4383	0.2647	CLP1, cleavage and polyadenylation factor I subunit, homolog (S. cerevisiae)
13655	BRDT	-0.4383	0.0902	bromodomain, testis-specific
13656	SNHG6	-0.4386	0.9996	small nucleolar RNA host gene 6 (non-protein coding)
13657	PATL2	-0.4386	0.2271	protein associated with topoisomerase II homolog 2 (yeast)
13658	BLOC1S3	-0.4386	0.172	biogenesis of lysosomal organelles complex-1, subunit 3
13659	STOX1	-0.4387	0.0841	storkhead box 1
13660	RIMS4	-0.4387	0.3704	regulating synaptic membrane exocytosis 4
13661	OSCAR	-0.4387	0.2426	osteoclast associated, immunoglobulin-like receptor
13662	NUP35	-0.4387	0.3681	nucleoporin 35kDa
13663	G2E3	-0.4387	0.3584	G2/M-phase specific E3 ubiquitin protein ligase
13664	FTHL17	-0.4387	0.246	ferritin, heavy polypeptide-like 17
13665	ABI3	-0.4387	0.3114	ABI family, member 3
13666	ZFAND3	-0.4392	0.1624	zinc finger, AN1-type domain 3
13667	sept-04	-0.4392	0.2841	septin 4
13668	UBE2NL	-0.44	0.1759	ubiquitin-conjugating enzyme E2N-like
13669	SST	-0.44	0.2139	somatostatin
13670	SLC25A25	-0.44	0.0665	solute carrier family 25 (mitochondrial carrier; phosphate carrier), member 25
13671	PTCD1	-0.44	0.1677	pentatricopeptide repeat domain 1
13672	PPP1R2	-0.44	0.352	protein phosphatase 1, regulatory (inhibitor) subunit 2
13673	NPPC	-0.44	0.2458	natriuretic peptide C
13674	NKG7	-0.44	0.2079	natural killer cell group 7 sequence
13675	MTHFD2	-0.44	0.3301	methylenetetrahydrofolate dehydrogenase (NADP+ dependent) 2, methenyltetrahydrofolate cyclohydrolase
13676	LCN8	-0.44	0.3528	lipocalin 8
13677	DCX	-0.44	0.2686	doublecortin
13678	CXorf67	-0.44	0.4112	chromosome X open reading frame 67
13679	CIB4	-0.44	0.3015	calcium and integrin binding family member 4
13680	CCDC140	-0.44	0.3066	coiled-coil domain containing 140
13681	C7orf57	-0.44	0.0577	chromosome 7 open reading frame 57
13682	C2orf73	-0.44	0.2475	chromosome 2 open reading frame 73
13683	TNFRSF8	-0.4408	0.2782	tumor necrosis factor receptor superfamily, member 8
13684	TMPO	-0.4408	0.3518	thymopoietin
13685	TAF1A	-0.4408	0.3539	TATA box binding protein (TBP)-associated factor, RNA polymerase I, A, 48kDa
13686	SHROOM2	-0.4408	0.085	shroom family member 2
13687	NCLN	-0.4408	0.2567	nicalin
13688	MSL2	-0.4408	0.2803	male-specific lethal 2 homolog (Drosophila)
13689	MED22	-0.4408	0.1995	mediator complex subunit 22
13690	TAS2R40	-0.4412	0.1373	taste receptor, type 2, member 40

13691	QRICH2	-0.4412	0.1574	glutamine rich 2
13692	INO80C	-0.4412	0.2276	INO80 complex subunit C
13693	C4orf46	-0.4412	0.3221	chromosome 4 open reading frame 46
13694	TTF2	-0.4417	0.349	transcription termination factor, RNA polymerase II
13695	SPAM1	-0.4417	0.3543	sperm adhesion molecule 1 (PH-20 hyaluronidase, zona pellucida binding)
13696	RHD	-0.4417	0.2549	Rh blood group, D antigen
13697	RBM14	-0.4417	0.2265	RNA binding motif protein 14
13698	MAGEC1	-0.4417	0.2344	melanoma antigen family C, 1
13699	KIAA0226L	-0.4417	0.1039	KIAA0226-like
13700	ZNF232	-0.4425	0.2482	zinc finger protein 232
13701	VTI1A	-0.4425	0.0688	vesicle transport through interaction with t-SNAREs homolog 1A (yeast)
13702	TRIM33	-0.4425	0.3187	tripartite motif containing 33
13703	TP53TG5	-0.4425	0.3768	TP53 target 5
13704	TCTEX1D1	-0.4425	0.0463	Tctex1 domain containing 1
13705	RGS18	-0.4425	0.1691	regulator of G-protein signaling 18
13706	RAB35	-0.4425	0.1548	RAB35, member RAS oncogene family
13707	NDUFA13	-0.4425	0.2879	NADH dehydrogenase (ubiquinone) 1 alpha subcomplex, 13
13708	LPIN2	-0.4425	0.1913	lipin 2
13709	GHRLOS2	-0.4425	0.0647	ghrelin opposite strand RNA 2 (non-protein coding)
13710	ELMO2	-0.4425	0.2257	engulfment and cell motility 2
13711	CRAMP1L	-0.4425	0.1793	Crm, cramped-like (Drosophila)
13712	C2CD4B	-0.4425	0.3292	C2 calcium-dependent domain containing 4B
13713	ANKRD19P	-0.4425	0.0736	ankyrin repeat domain 19, pseudogene
13714	AMOT	-0.4425	0.1099	angiomotin
13715	TULP4	-0.4433	0.1723	tubby like protein 4
13716	MAP4K2	-0.4433	0.2019	mitogen-activated protein kinase kinase kinase kinase 2
13717	KIAA0930	-0.4433	0.144	KIAA0930
13718	HYAL3	-0.4433	0.1675	hyaluronoglucosaminidase 3
13719	CTSG	-0.4433	0.2669	cathepsin G
13720	ASB6	-0.4433	0.1862	ankyrin repeat and SOCS box containing 6
13721	ABCG4	-0.4433	0.354	ATP-binding cassette, sub-family G (WHITE), member 4
13722	TNFRSF18	-0.4437	0.2908	tumor necrosis factor receptor superfamily, member 18
13723	MAEL	-0.4437	0.0355	maelstrom homolog (Drosophila)
13724	KIAA1715	-0.4437	0.3055	KIAA1715
13725	C11orf87	-0.4437	0.0823	chromosome 11 open reading frame 87
13726	STMN4	-0.4442	0.3065	stathmin-like 4
13727	RNF44	-0.4442	0.1941	ring finger protein 44
13728	KIF5A	-0.4442	0.2382	kinesin family member 5A
13729	IPO4	-0.4442	0.249	importin 4
13730	DGCR8	-0.4442	0.161	DiGeorge syndrome critical region gene 8
13731	LOC727924	-0.4443	0.9996	uncharacterized LOC727924
13732	LOC100130111	-0.4443	0.9999	uncharacterized LOC100130111
13733	WDR76	-0.445	0.2301	WD repeat domain 76
13734	SLC9A5	-0.445	0.274	solute carrier family 9, subfamily A (NHE5, cation proton antiporter 5), member 5
13735	PHKG1	-0.445	0.3086	phosphorylase kinase, gamma 1 (muscle)
13736	GCH1	-0.445	0.2681	GTP cyclohydrolase 1
13737	DBNDD1	-0.445	0.2197	dysbindin (dystrobrevin binding protein 1) domain containing 1
13738	CYTH4	-0.445	0.304	cytohesin 4
13739	C21orf119	-0.445	0.1381	chromosome 21 open reading frame 119
13740	C20orf85	-0.445	0.263	chromosome 20 open reading frame 85
13741	ADC	-0.445	0.2019	arginine decarboxylase
13742	LOC152225	-0.4457	0.9999	uncharacterized LOC152225
13743	ZNF787	-0.4458	0.2778	zinc finger protein 787
13744	PTGES3	-0.4458	0.375	prostaglandin E synthase 3 (cytosolic)
13745	ORM1	-0.4458	0.0385	orosomucoid 1
13746	LPPR3	-0.4458	0.3181	lipid phosphate phosphatase-related protein type 3
13747	PHACTR3	-0.4462	0.0649	phosphatase and actin regulator 3
13748	LRRC4C	-0.4462	0.1601	leucine rich repeat containing 4C
13749	DNAJC27	-0.4462	0.1863	DnaJ (Hsp40) homolog, subfamily C, member 27
13750	ZNF391	-0.4464	0.0876	zinc finger protein 391
13751	SYT17	-0.4467	0.0965	synaptotagmin XVII
13752	RAD17	-0.4467	0.3413	RAD17 homolog (S. pombe)
13753	IGLL1	-0.4467	0.0927	immunoglobulin lambda-like polypeptide 1
13754	HIST1H3F	-0.4467	0.1011	histone cluster 1, H3f
13755	EXOSC2	-0.4467	0.3114	exosome component 2
13756	CROCC	-0.4467	0.2677	ciliary rootlet coiled-coil, rootletin
13757	C16orf71	-0.4467	0.3826	chromosome 16 open reading frame 71
13758	LOC90784	-0.4471	0.9996	uncharacterized LOC90784
13759	ZNF572	-0.4475	0.0816	zinc finger protein 572
13760	WDR74	-0.4475	0.2441	WD repeat domain 74
13761	TMEM158	-0.4475	0.1539	transmembrane protein 158 (gene/pseudogene)
13762	SLAMF9	-0.4475	0.3694	SLAM family member 9
13763	RRP8	-0.4475	0.2184	ribosomal RNA processing 8, methyltransferase, homolog (yeast)
13764	PRKCG	-0.4475	0.4084	protein kinase C, gamma
13765	PPP5C	-0.4475	0.1953	protein phosphatase 5, catalytic subunit
13766	NDUFB2-AS1	-0.4475	0.2458	NDUFB2 antisense RNA 1 (non-protein coding)
13767	LOC730101	-0.4475	0.0925	uncharacterized LOC730101
13768	LOC439949	-0.4475	0.1289	uncharacterized LOC439949
13769	FAM43B	-0.4475	0.3443	family with sequence similarity 43, member B
13770	CRIP3	-0.4475	0.1712	cysteine-rich protein 3
13771	CLASP1	-0.4475	0.2347	cytoplasmic linker associated protein 1
13772	CHMP6	-0.4475	0.2177	charged multivesicular body protein 6
13773	CCDC65	-0.4475	0.1097	coiled-coil domain containing 65
13774	CCDC130	-0.4475	0.1887	coiled-coil domain containing 130

13775	PMAIP1	-0.4483	0.2385	phorbol-12-myristate-13-acetate-induced protein 1
13776	HRH3	-0.4483	0.4323	histamine receptor H3
13777	FN3KRP	-0.4483	0.2249	fructosamine 3 kinase related protein
13778	WDR24	-0.4487	0.2129	WD repeat domain 24
13779	LOC728743	-0.4487	0.2894	zinc finger protein pseudogene
13780	LOC148709	-0.4487	0.0681	actin pseudogene
13781	GSX1	-0.4487	0.3231	GS homeobox 1
13782	SYNCRIP	-0.4492	0.3709	synaptotagmin binding, cytoplasmic RNA interacting protein
13783	SEMA7A	-0.4492	0.2776	semaphorin 7A, GPI membrane anchor (John Milton Hagen blood group)
13784	OMP	-0.4492	0.3025	olfactory marker protein
13785	MIF	-0.4492	0.2694	macrophage migration inhibitory factor (glycosylation-inhibiting factor)
13786	COL11A2	-0.4492	0.4242	collagen, type XI, alpha 2
13787	ADD2	-0.4492	0.2589	adducin 2 (beta)
13788	TOMM5	-0.45	0.3306	translocase of outer mitochondrial membrane 5 homolog (yeast)
13789	RPL37A	-0.45	0.2294	ribosomal protein L37a
13790	RFWD3	-0.45	0.2794	ring finger and WD repeat domain 3
13791	PHC2	-0.45	0.1738	polyhomeotic homolog 2 (Drosophila)
13792	LOC415056	-0.45	0.319	uncharacterized LOC415056
13793	LINC00515	-0.45	0.0547	long intergenic non-protein coding RNA 515
13794	LCORL	-0.45	0.2827	ligand dependent nuclear receptor corepressor-like
13795	HHIP	-0.45	0.0763	hedgehog interacting protein
13796	CYMP	-0.45	0.9996	chymosin pseudogene
13797	USP13	-0.4508	0.1726	ubiquitin specific peptidase 13 (isopeptidase T-3)
13798	GUCA1B	-0.4508	0.3557	guanylate cyclase activator 1B (retina)
13799	EIF3G	-0.4508	0.2436	eukaryotic translation initiation factor 3, subunit G
13800	ZNF488	-0.4512	0.2449	zinc finger protein 488
13801	WDR16	-0.4512	0.1743	WD repeat domain 16
13802	SUFU	-0.4512	0.1079	suppressor of fused homolog (Drosophila)
13803	SNHG15	-0.4512	0.1927	small nucleolar RNA host gene 15 (non-protein coding)
13804	SFT2D3	-0.4512	0.1076	SFT2 domain containing 3
13805	MYLK2	-0.4512	0.3756	myosin light chain kinase 2
13806	MEX3B	-0.4512	0.085	mex-3 homolog B (C. elegans)
13807	BARHL1	-0.4512	0.4135	BarH-like homeobox 1
13808	VSTM1	-0.4514	0.0742	V-set and transmembrane domain containing 1
13809	LOC340094	-0.4514	0.9996	uncharacterized LOC340094
13810	C2orf82	-0.4514	0.2812	chromosome 2 open reading frame 82
13811	C20orf173	-0.4514	0.3757	chromosome 20 open reading frame 173
13812	UMPS	-0.4517	0.3017	uridine monophosphate synthetase
13813	TTLL12	-0.4517	0.2288	tubulin tyrosine ligase-like family, member 12
13814	GEMIN7	-0.4517	0.1533	gem (nuclear organelle) associated protein 7
13815	SNRNP48	-0.4525	0.2909	small nuclear ribonucleoprotein 48kDa (U11/U12)
13816	RGS22	-0.4525	0.1222	regulator of G-protein signaling 22
13817	RBM46	-0.4525	0.0539	RNA binding motif protein 46
13818	LINC00338	-0.4525	0.1373	long intergenic non-protein coding RNA 338
13819	EYS	-0.4525	0.0706	eyes shut homolog (Drosophila)
13820	DCTN3	-0.4525	0.2102	dynactin 3 (p22)
13821	CTCF	-0.4525	0.345	CCCTC-binding factor (zinc finger protein)
13822	CABP5	-0.4525	0.4022	calcium binding protein 5
13823	ALOX5AP	-0.4525	0.176	arachidonate 5-lipoxygenase-activating protein
13824	AADAT	-0.4525	0.0891	aminoadipate aminotransferase
13825	SHISA9	-0.4529	0.3511	shisa homolog 9 (Xenopus laevis)
13826	ONECUT3	-0.4529	0.325	one cut homeobox 3
13827	LINC00305	-0.4529	0.1271	long intergenic non-protein coding RNA 305
13828	CBX3P2	-0.4529	0.0614	chromobox homolog 3 pseudogene 2
13829	UBE2M	-0.4533	0.2598	ubiquitin-conjugating enzyme E2M
13830	SDAD1	-0.4533	0.3253	SDA1 domain containing 1
13831	OTUB1	-0.4533	0.2359	OTU domain, ubiquitin aldehyde binding 1
13832	MGC15705	-0.4533	0.9996	uncharacterized protein MGC15705
13833	WFIKKN2	-0.4538	0.3929	WAP, follistatin/kazal, immunoglobulin, kunitz and netrin domain containing 2
13834	TTC7B	-0.4538	0.1231	tetratricopeptide repeat domain 7B
13835	ITGAD	-0.4538	0.2918	integrin, alpha D
13836	ASXL3	-0.4538	0.2237	additional sex combs like 3 (Drosophila)
13837	TIMM44	-0.4542	0.2335	translocase of inner mitochondrial membrane 44 homolog (yeast)
13838	GTF2F1	-0.4542	0.1956	general transcription factor IIF, polypeptide 1, 74kDa
13839	EMC9	-0.4542	0.1845	ER membrane protein complex subunit 9
13840	DEF6	-0.4542	0.2065	differentially expressed in FDCP 6 homolog (mouse)
13841	CHST2	-0.4542	0.0821	carbohydrate (N-acetylglucosamine-6-O) sulfotransferase 2
13842	CCDC90B	-0.4542	0.3496	coiled-coil domain containing 90B
13843	C22orf31	-0.4542	0.3353	chromosome 22 open reading frame 31
13844	LOC728095	-0.4543	0.9996	uncharacterized LOC728095
13845	ZNF266	-0.455	0.2365	zinc finger protein 266
13846	VASH1	-0.455	0.1248	vasohibin 1
13847	SNAP25	-0.455	0.1198	synaptosomal-associated protein, 25kDa
13848	PPY	-0.455	0.2745	pancreatic polypeptide
13849	POU5F2	-0.455	0.3708	POU domain class 5, transcription factor 2
13850	PMCHL1	-0.455	0.0899	pro-melanin-concentrating hormone-like 1, pseudogene
13851	L3MBTL2	-0.455	0.1597	l(3)mbt-like 2 (Drosophila)
13852	JPH1	-0.455	0.089	junctophilin 1
13853	HNRNPA2B1	-0.455	0.3562	heterogeneous nuclear ribonucleoprotein A2/B1
13854	FFAR2	-0.455	0.1572	free fatty acid receptor 2
13855	DDX47	-0.455	0.3307	DEAD (Asp-Glu-Ala-Asp) box polypeptide 47
13856	C11orf40	-0.455	0.1566	chromosome 11 open reading frame 40
13857	ATAD3B	-0.455	0.2754	ATPase family, AAA domain containing 3B
13858	ANKRD45	-0.455	0.036	ankyrin repeat domain 45

13859	PARK7	-0.4558	0.3227	parkinson protein 7
13860	C14orf162	-0.4558	0.3237	chromosome 14 open reading frame 162
13861	ZDHHC19	-0.4563	0.4044	zinc finger, DHHC-type containing 19
13862	SYT6	-0.4563	0.2126	synaptotagmin VI
13863	SLC22A9	-0.4563	0.2832	solute carrier family 22 (organic anion transporter), member 9
13864	SEPT7P2	-0.4563	0.1685	septin 7 pseudogene 2
13865	RGS7BP	-0.4563	0.0409	regulator of G-protein signaling 7 binding protein
13866	PKD1L2	-0.4563	0.3907	polycystic kidney disease 1-like 2
13867	LRRC37B	-0.4563	0.2353	leucine rich repeat containing 37B
13868	LOC126536	-0.4563	0.3564	uncharacterized LOC126536
13869	KIAA1731	-0.4563	0.2578	KIAA1731
13870	IQCD	-0.4563	0.1253	IQ motif containing D
13871	DMRTC2	-0.4563	0.1733	DMRT-like family C2
13872	LYRM2	-0.4564	0.2746	LYR motif containing 2
13873	SULT4A1	-0.4567	0.2465	sulfotransferase family 4A, member 1
13874	STAM	-0.4567	0.3396	signal transducing adaptor molecule (SH3 domain and ITAM motif) 1
13875	SPOP	-0.4567	0.2515	speckle-type POZ protein
13876	SMARCD1	-0.4567	0.1667	SWI/SNF related, matrix associated, actin dependent regulator of chromatin, subfamily d, member 1
13877	SIK3	-0.4567	0.1713	SIK family kinase 3
13878	PRPS1	-0.4567	0.2875	phosphoribosyl pyrophosphate synthetase 1
13879	GPR162	-0.4567	0.2845	G protein-coupled receptor 162
13880	GAPDHS	-0.4567	0.3363	glyceraldehyde-3-phosphate dehydrogenase, spermatogenic
13881	C16orf57	-0.4567	0.1615	chromosome 16 open reading frame 57
13882	ADAM18	-0.4567	0.2928	ADAM metallopeptidase domain 18
13883	TMEM229B	-0.4575	0.0982	transmembrane protein 229B
13884	TESK2	-0.4575	0.1036	testis-specific kinase 2
13885	SP5	-0.4575	0.1682	Sp5 transcription factor
13886	SCAF4	-0.4575	0.1768	SR-related CTD-associated factor 4
13887	MGC45922	-0.4575	0.4406	uncharacterized LOC284365
13888	LIG4	-0.4575	0.2898	ligase IV, DNA, ATP-dependent
13889	CUEDC2	-0.4575	0.2322	CUE domain containing 2
13890	CRYBA2	-0.4575	0.2732	crystallin, beta A2
13891	SPEN	-0.4583	0.2158	spen homolog, transcriptional regulator (Drosophila)
13892	RNF216	-0.4583	0.1169	ring finger protein 216
13893	MBD4	-0.4583	0.3621	methyl-CpG binding domain protein 4
13894	LINC00337	-0.4583	0.9999	long intergenic non-protein coding RNA 337
13895	KCNE1L	-0.4583	0.2471	KCNE1-like
13896	ALDOC	-0.4583	0.0709	aldolase C, fructose-bisphosphate
13897	PCA3	-0.4586	0.9996	prostate cancer antigen 3 (non-protein coding)
13898	HMGB3P30	-0.4586	0.9999	high mobility group box 3 pseudogene 30
13899	LOC440900	-0.4588	0.2787	uncharacterized LOC440900
13900	DIRC1	-0.4588	0.3132	disrupted in renal carcinoma 1
13901	CARNS1	-0.4588	0.3084	carnosine synthase 1
13902	C20orf96	-0.4588	0.107	chromosome 20 open reading frame 96
13903	C19orf55	-0.4588	0.1414	chromosome 19 open reading frame 55
13904	TIMM13	-0.4592	0.2502	translocase of inner mitochondrial membrane 13 homolog (yeast)
13905	THOC6	-0.4592	0.2251	THO complex 6 homolog (Drosophila)
13906	STRAP	-0.4592	0.3388	serine/threonine kinase receptor associated protein
13907	PNMA2	-0.4592	0.0603	paraneoplastic Ma antigen 2
13908	PAGE4	-0.4592	0.2346	P antigen family, member 4 (prostate associated)
13909	DYNLL1	-0.4592	0.3159	dynein, light chain, LC8-type 1
13910	TMEFF2	-0.46	0.0444	transmembrane protein with EGF-like and two follistatin-like domains 2
13911	SIN3B	-0.46	0.1507	SIN3 transcription regulator homolog B (yeast)
13912	P2RY13	-0.46	0.1773	purinergic receptor P2Y, G-protein coupled, 13
13913	NUP210P1	-0.46	0.9999	nucleoporin 210kDa pseudogene 1
13914	MTMR9	-0.46	0.2375	myotubularin related protein 9
13915	GPN1	-0.46	0.3039	GPN-loop GTPase 1
13916	GPBP1	-0.46	0.3372	GC-rich promoter binding protein 1
13917	FAIM2	-0.46	0.3245	Fas apoptotic inhibitory molecule 2
13918	ENOX2	-0.46	0.1954	ecto-NOX disulfide-thiol exchanger 2
13919	CEP152	-0.46	0.2861	centrosomal protein 152kDa
13920	CCDC155	-0.46	0.3518	coiled-coil domain containing 155
13921	ATXN2L	-0.46	0.2092	ataxin 2-like
13922	POU3F3	-0.4608	0.35	POU class 3 homeobox 3
13923	MST4	-0.4608	0.3134	serine/threonine protein kinase MST4
13924	LYRM1	-0.4608	0.2531	LYR motif containing 1
13925	FRAT1	-0.4608	0.1221	frequently rearranged in advanced T-cell lymphomas
13926	TMIE	-0.4613	0.3162	transmembrane inner ear
13927	SYCP3	-0.4613	0.0797	synaptonemal complex protein 3
13928	KIAA1919	-0.4613	0.0741	KIAA1919
13929	C17orf66	-0.4613	0.1916	chromosome 17 open reading frame 66
13930	THEG	-0.4617	0.3903	theg spermatid protein
13931	PMM1	-0.4617	0.1696	phosphomannomutase 1
13932	IL12RB1	-0.4617	0.2711	interleukin 12 receptor, beta 1
13933	CASC3	-0.4617	0.1932	cancer susceptibility candidate 3
13934	SSBP4	-0.4625	0.2258	single stranded DNA binding protein 4
13935	SLC38A9	-0.4625	0.3135	solute carrier family 38, member 9
13936	GPR128	-0.4625	0.0526	G protein-coupled receptor 128
13937	FAM83D	-0.4625	0.2496	family with sequence similarity 83, member D
13938	EIF2B3	-0.4625	0.2791	eukaryotic translation initiation factor 2B, subunit 3 gamma, 58kDa
13939	TMEFF1	-0.4629	0.1686	transmembrane protein with EGF-like and two follistatin-like domains 1
13940	ZNF8	-0.4633	0.0843	zinc finger protein 8
13941	ZMYM3	-0.4633	0.1374	zinc finger, MYM-type 3
13942	WDR91	-0.4633	0.0711	WD repeat domain 91

13943	TRMU	-0.4633	0.1913	tRNA 5-methylaminomethyl-2-thiouridylate methyltransferase
13944	RPS2	-0.4633	0.2292	ribosomal protein S2
13945	MCM3AP-AS1	-0.4633	0.1494	MCM3AP antisense RNA 1 (non-protein coding)
13946	TAAR8	-0.4638	0.2932	trace amine associated receptor 8
13947	NLRP8	-0.4638	0.3582	NLR family, pyrin domain containing 8
13948	LRRC45	-0.4638	0.2678	leucine rich repeat containing 45
13949	IL17F	-0.4638	0.3059	interleukin 17F
13950	FLCN	-0.4638	0.0558	folliculin
13951	C2CD4C	-0.4638	0.3828	C2 calcium-dependent domain containing 4C
13952	TFDP2	-0.4642	0.1342	transcription factor Dp-2 (E2F dimerization partner 2)
13953	PLAGL2	-0.4642	0.1084	pleiomorphic adenoma gene-like 2
13954	NKX2-8	-0.4642	0.3676	NK2 homeobox 8
13955	ASNA1	-0.4642	0.2255	arsA arsenite transporter, ATP-binding, homolog 1 (bacterial)
13956	TRIM78P	-0.4643	0.9999	tripartite motif containing 78, pseudogene
13957	ZNF642	-0.465	0.226	zinc finger protein 642
13958	SLC35G2	-0.465	0.1213	solute carrier family 35, member G2
13959	SELL	-0.465	0.1522	selectin L
13960	SEC61G	-0.465	0.3028	Sec61 gamma subunit
13961	POLE3	-0.465	0.3356	polymerase (DNA directed), epsilon 3, accessory subunit
13962	PNMAL1	-0.465	0.074	paraneoplastic Ma antigen family-like 1
13963	MCM3AP	-0.465	0.1967	minichromosome maintenance complex component 3 associated protein
13964	LOXHD1	-0.465	0.2872	lipoxygenase homology domains 1
13965	LOC729173	-0.465	0.9996	uncharacterized LOC729173
13966	ERVFRD-1	-0.465	0.2207	endogenous retrovirus group FRD, member 1
13967	DDX28	-0.465	0.1983	DEAD (Asp-Glu-Ala-Asp) box polypeptide 28
13968	DCTN2	-0.465	0.1391	dynactin 2 (p50)
13969	CRYM	-0.465	0.091	crystallin, mu
13970	C21orf56	-0.465	0.1673	chromosome 21 open reading frame 56
13971	ATAT1	-0.465	0.1534	alpha tubulin acetyltransferase 1
13972	SBNO1	-0.4657	0.2803	strawberry notch homolog 1 (Drosophila)
13973	TCEA2	-0.4658	0.1759	transcription elongation factor A (SII), 2
13974	RABGGTB	-0.4658	0.3644	Rab geranylgeranyltransferase, beta subunit
13975	HMOX2	-0.4658	0.1956	heme oxygenase (decycling) 2
13976	DENND1C	-0.4658	0.2652	DENN/MADD domain containing 1C
13977	AGRP	-0.4658	0.3571	agouti related protein homolog (mouse)
13978	ADNP2	-0.4658	0.2266	ADNP homeobox 2
13979	STRBP	-0.4663	0.2857	spermatid perinuclear RNA binding protein
13980	GPRC6A	-0.4663	0.2596	G protein-coupled receptor, family C, group 6, member A
13981	FIBCD1	-0.4663	0.3914	fibrinogen C domain containing 1
13982	FAM100A	-0.4663	0.2231	family with sequence similarity 100, member A
13983	DNAJB8	-0.4663	0.2992	DnaJ (Hsp40) homolog, subfamily B, member 8
13984	TRAPPC2L	-0.4667	0.2318	trafficking protein particle complex 2-like
13985	THAP1	-0.4667	0.3505	THAP domain containing, apoptosis associated protein 1
13986	TARDBP	-0.4667	0.3133	TAR DNA binding protein
13987	SMCR7L	-0.4667	0.2308	Smith-Magenis syndrome chromosome region, candidate 7-like
13988	PTPRA	-0.4667	0.1552	protein tyrosine phosphatase, receptor type, A
13989	PPM1H	-0.4667	0.07	protein phosphatase, Mg2+/Mn2+ dependent, 1H
13990	PDHB	-0.4667	0.3349	pyruvate dehydrogenase (lipoamide) beta
13991	PAOX	-0.4667	0.1774	polyamine oxidase (exo-N4-amino)
13992	MRPL20	-0.4667	0.3169	mitochondrial ribosomal protein L20
13993	CAD	-0.4667	0.2518	carbamoyl-phosphate synthetase 2, aspartate transcarbamylase, and dihydroorotase
13994	ATXN2	-0.4667	0.1939	ataxin 2
13995	OR1J2	-0.4671	0.2726	olfactory receptor, family 1, subfamily J, member 2
13996	LOC93622	-0.4671	0.221	Morf4 family associated protein 1-like 1 pseudogene
13997	LOC151162	-0.4671	0.1379	hypothetical LOC151162
13998	ZNF485	-0.4675	0.2051	zinc finger protein 485
13999	UBLCP1	-0.4675	0.3206	ubiquitin-like domain containing CTD phosphatase 1
14000	TREML3P	-0.4675	0.3741	triggering receptor expressed on myeloid cells-like 3, pseudogene
14001	TARBP2	-0.4675	0.2392	TAR (HIV-1) RNA binding protein 2
14002	SS18L1	-0.4675	0.2868	synovial sarcoma translocation gene on chromosome 18-like 1
14003	SMCP	-0.4675	0.3775	sperm mitochondria-associated cysteine-rich protein
14004	NEUROD1	-0.4675	0.1519	neuronal differentiation 1
14005	HNRNPK	-0.4675	0.3425	heterogeneous nuclear ribonucleoprotein K
14006	HDGFRP3	-0.4675	0.2485	hepatoma-derived growth factor, related protein 3
14007	CACNG2	-0.4675	0.3776	calcium channel, voltage-dependent, gamma subunit 2
14008	C9orf11	-0.4675	0.1074	chromosome 9 open reading frame 11
14009	ACOT8	-0.4675	0.1845	acyl-CoA thioesterase 8
14010	TMOD1	-0.4683	0.049	tropomodulin 1
14011	SMPD3	-0.4683	0.132	sphingomyelin phosphodiesterase 3, neutral membrane (neutral sphingomyelinase II)
14012	RRAGC	-0.4683	0.1601	Ras-related GTP binding C
14013	DOK2	-0.4683	0.2591	docking protein 2, 56kDa
14014	BAI1	-0.4683	0.3995	brain-specific angiogenesis inhibitor 1
14015	YBEY	-0.4688	0.1004	ybeY metallopeptidase (putative)
14016	TSEN15	-0.4688	0.2993	tRNA splicing endonuclease 15 homolog (S. cerevisiae)
14017	SPHKAP	-0.4688	0.2005	SPHK1 interactor, AKAP domain containing
14018	SIGLEC10	-0.4688	0.2107	sialic acid binding Ig-like lectin 10
14019	PARP15	-0.4688	0.1021	poly (ADP-ribose) polymerase family, member 15
14020	C19orf44	-0.4688	0.1107	chromosome 19 open reading frame 44
14021	ARMC4	-0.4688	0.0502	armadillo repeat containing 4
14022	TTLL7	-0.4692	0.071	tubulin tyrosine ligase-like family, member 7
14023	SLC30A3	-0.4692	0.327	solute carrier family 30 (zinc transporter), member 3
14024	MRPL3	-0.4692	0.3859	mitochondrial ribosomal protein L3
14025	HIST1H4F	-0.4692	0.044	histone cluster 1, H4f
14026	HBXIP	-0.4692	0.2862	hepatitis B virus x interacting protein

14027	ERI2	-0.4692	0.2589	ERI1 exoribonuclease family member 2
14028	ASPHD1	-0.4692	0.1841	aspartate beta-hydroxylase domain containing 1
14029	ZNF416	-0.47	0.0717	zinc finger protein 416
14030	VAMP2	-0.47	0.1741	vesicle-associated membrane protein 2 (synaptobrevin 2)
14031	STAC3	-0.47	0.1851	SH3 and cysteine rich domain 3
14032	SEMA4F	-0.47	0.1179	sema domain, immunoglobulin domain (Ig), transmembrane domain (TM) and short cytoplasmic domain, (semaphorin) 4F
14033	RP1L1	-0.47	0.2953	retinitis pigmentosa 1-like 1
14034	PDE4C	-0.47	0.4068	phosphodiesterase 4C, cAMP-specific
14035	NLK	-0.47	0.2307	nemo-like kinase
14036	LPCAT1	-0.47	0.1511	lysophosphatidylcholine acyltransferase 1
14037	LOC731779	-0.47	0.9996	uncharacterized LOC731779
14038	KIRREL2	-0.47	0.3203	kin of IRRE like 2 (Drosophila)
14039	KCTD2	-0.47	0.1191	potassium channel tetramerisation domain containing 2
14040	IL16	-0.47	0.2245	interleukin 16
14041	GABRG2	-0.47	0.2667	gamma-aminobutyric acid (GABA) A receptor, gamma 2
14042	FIG4	-0.47	0.145	FIG4 homolog, SAC1 lipid phosphatase domain containing (S. cerevisiae)
14043	C9orf142	-0.47	0.2504	chromosome 9 open reading frame 142
14044	C6orf164	-0.47	0.0695	chromosome 6 open reading frame 164
14045	ARHGAP39	-0.47	0.1559	Rho GTPase activating protein 39
14046	ALKBH1	-0.47	0.1703	alkB, alkylation repair homolog 1 (E. coli)
14047	ZNF238	-0.4708	0.1775	zinc finger protein 238
14048	RMND5A	-0.4708	0.1996	required for meiotic nuclear division 5 homolog A (S. cerevisiae)
14049	ODF1	-0.4708	0.373	outer dense fiber of sperm tails 1
14050	MTL5	-0.4708	0.0674	metallothionein-like 5, testis-specific (tesmin)
14051	KCNA6	-0.4708	0.3685	potassium voltage-gated channel, shaker-related subfamily, member 6
14052	GUCY1B3	-0.4708	0.1041	guanylate cyclase 1, soluble, beta 3
14053	ZNF628	-0.4712	0.2569	zinc finger protein 628
14054	USP31	-0.4712	0.1684	ubiquitin specific peptidase 31
14055	SNX32	-0.4712	0.2372	sorting nexin 32
14056	MS4A14	-0.4712	0.1085	membrane-spanning 4-domains, subfamily A, member 14
14057	GEMIN5	-0.4712	0.2852	gem (nuclear organelle) associated protein 5
14058	FAM133A	-0.4712	0.0638	family with sequence similarity 133, member A
14059	EIF1AD	-0.4712	0.1551	eukaryotic translation initiation factor 1A domain containing
14060	UPF3B	-0.4717	0.3396	UPF3 regulator of nonsense transcripts homolog B (yeast)
14061	SRR	-0.4717	0.1742	serine racemase
14062	SETDB1	-0.4717	0.1529	SET domain, bifurcated 1
14063	NHLH2	-0.4717	0.139	nescient helix loop helix 2
14064	NFE2	-0.4717	0.0874	nuclear factor (erythroid-derived 2), 45kDa
14065	LOC100132815	-0.4717	0.9999	importin 5 pseudogene
14066	GTF2A2	-0.4717	0.3322	general transcription factor IIA, 2, 12kDa
14067	DYRK1A	-0.4717	0.1696	dual-specificity tyrosine-(Y)-phosphorylation regulated kinase 1A
14068	C6orf130	-0.4717	0.3292	chromosome 6 open reading frame 130
14069	ATP13A1	-0.4717	0.1925	ATPase type 13A1
14070	ADCYAP1	-0.4717	0.2249	adenylate cyclase activating polypeptide 1 (pituitary)
14071	UBE2L3	-0.4725	0.2442	ubiquitin-conjugating enzyme E2L 3
14072	TRIM35	-0.4725	0.059	tripartite motif containing 35
14073	RNF7	-0.4725	0.2802	ring finger protein 7
14074	RCHY1	-0.4725	0.3583	ring finger and CHY zinc finger domain containing 1, E3 ubiquitin protein ligase
14075	MS4A15	-0.4725	0.2596	membrane-spanning 4-domains, subfamily A, member 15
14076	MAGOHB	-0.4725	0.2857	mago-nashi homolog B (Drosophila)
14077	INTS1	-0.4725	0.2366	integrator complex subunit 1
14078	GPX4	-0.4725	0.193	glutathione peroxidase 4
14079	GPRC5B	-0.4725	0.0412	G protein-coupled receptor, family C, group 5, member B
14080	FAM71D	-0.4725	0.2333	family with sequence similarity 71, member D
14081	CUL2	-0.4725	0.3419	cullin 2
14082	C2orf80	-0.4725	0.111	chromosome 2 open reading frame 80
14083	C17orf98	-0.4725	0.0339	chromosome 17 open reading frame 98
14084	ATP1B2	-0.4725	0.291	ATPase, Na+/K+ transporting, beta 2 polypeptide
14085	ALDH5A1	-0.4725	0.1512	aldehyde dehydrogenase 5 family, member A1
14086	POTEKP	-0.4727	0.0258	POTE ankyrin domain family, member K, pseudogene
14087	VAC14	-0.4733	0.1722	Vac14 homolog (S. cerevisiae)
14088	TBL3	-0.4733	0.2256	transducin (beta)-like 3
14089	SUPT4H1	-0.4733	0.1894	suppressor of Ty 4 homolog 1 (S. cerevisiae)
14090	RHBDD3	-0.4733	0.2412	rhomboid domain containing 3
14091	PRR14	-0.4733	0.2094	proline rich 14
14092	LILRA2	-0.4733	0.244	leukocyte immunoglobulin-like receptor, subfamily A (with TM domain), member 2
14093	IKBKAP	-0.4733	0.3276	inhibitor of kappa light polypeptide gene enhancer in B-cells, kinase complex-associated protein
14094	HCK	-0.4733	0.2224	hemopoietic cell kinase
14095	GTF2B	-0.4733	0.3134	general transcription factor IIB
14096	FAM53B	-0.4733	0.1268	family with sequence similarity 53, member B
14097	ZNF697	-0.4737	0.0994	zinc finger protein 697
14098	TSPYL6	-0.4737	0.1392	TSPY-like 6
14099	TRIM11	-0.4737	0.1813	tripartite motif containing 11
14100	SYT16	-0.4737	0.0781	synaptotagmin XVI
14101	C9orf57	-0.4737	0.0855	chromosome 9 open reading frame 57
14102	ZNF771	-0.4742	0.2992	zinc finger protein 771
14103	RPP38	-0.4742	0.1981	ribonuclease P/MRP 38kDa subunit
14104	OAZ1	-0.4742	0.248	ornithine decarboxylase antizyme 1
14105	GPATCH3	-0.4742	0.1587	G patch domain containing 3
14106	GABRG3	-0.4742	0.0877	gamma-aminobutyric acid (GABA) A receptor, gamma 3
14107	DCUN1D2	-0.4742	0.1126	DCN1, defective in cullin neddylation 1, domain containing 2 (S. cerevisiae)
14108	MDGA2	-0.4743	0.0727	MAM domain containing glycosylphosphatidylinositol anchor 2
14109	LOC401442	-0.4743	0.9996	uncharacterized LOC401442
14110	ZCCHC9	-0.475	0.3256	zinc finger, CCHC domain containing 9

14111	TRUB2	-0.475	0.226	TruB pseudouridine (psi) synthase homolog 2 (E. coli)
14112	TAPT1	-0.475	0.2101	transmembrane anterior posterior transformation 1
14113	SRSF9	-0.475	0.3026	serine/arginine-rich splicing factor 9
14114	SNX20	-0.475	0.2309	sorting nexin 20
14115	SMYD4	-0.475	0.1006	SET and MYND domain containing 4
14116	PLCXD2	-0.475	0.0925	phosphatidylinositol-specific phospholipase C, X domain containing 2
14117	MZT1	-0.475	0.3356	mitotic spindle organizing protein 1
14118	COQ3	-0.475	0.2619	coenzyme Q3 homolog, methyltransferase (S. cerevisiae)
14119	CLEC2D	-0.475	0.1521	C-type lectin domain family 2, member D
14120	SUPV3L1	-0.4758	0.22	suppressor of var1, 3-like 1 (S. cerevisiae)
14121	STX18	-0.4758	0.1473	syntaxin 18
14122	UTP15	-0.4762	0.3304	UTP15, U3 small nucleolar ribonucleoprotein, homolog (S. cerevisiae)
14123	TAS1R1	-0.4762	0.3849	taste receptor, type 1, member 1
14124	SLC25A2	-0.4762	0.1417	solute carrier family 25 (mitochondrial carrier; ornithine transporter) member 2
14125	NTNG2	-0.4762	0.1401	netrin G2
14126	GBP5	-0.4762	0.1565	guanylate binding protein 5
14127	CARD16	-0.4762	0.1672	caspase recruitment domain family, member 16
14128	C10orf35	-0.4762	0.1153	chromosome 10 open reading frame 35
14129	BCAR4	-0.4762	0.1108	breast cancer anti-estrogen resistance 4 (non-protein coding)
14130	TMEM39B	-0.4767	0.1789	transmembrane protein 39B
14131	TMEM110	-0.4767	0.0876	transmembrane protein 110
14132	KIF22	-0.4767	0.2624	kinesin family member 22
14133	INTS9	-0.4767	0.2008	integrator complex subunit 9
14134	CNNM1	-0.4767	0.16	cyclin M1
14135	ADAM11	-0.4767	0.3671	ADAM metallopeptidase domain 11
14136	PIRT	-0.4771	0.3986	phosphoinositide-interacting regulator of transient receptor potential channels
14137	C10orf85	-0.4771	0.3133	chromosome 10 open reading frame 85
14138	ZSCAN1	-0.4775	0.3448	zinc finger and SCAN domain containing 1
14139	USP36	-0.4775	0.131	ubiquitin specific peptidase 36
14140	SPHK2	-0.4775	0.2263	sphingosine kinase 2
14141	SLC26A1	-0.4775	0.3946	solute carrier family 26 (sulfate transporter), member 1
14142	SEMA6B	-0.4775	0.3546	sema domain, transmembrane domain (TM), and cytoplasmic domain, (semaphorin) 6B
14143	PSMC3	-0.4775	0.2831	proteasome (prosome, macropain) 26S subunit, ATPase, 3
14144	MAP3K10	-0.4775	0.4019	mitogen-activated protein kinase kinase kinase 10
14145	SPAST	-0.4783	0.3632	spastin
14146	SCGN	-0.4783	0.2125	secretagogin, EF-hand calcium binding protein
14147	SART1	-0.4783	0.2114	squamous cell carcinoma antigen recognized by T cells
14148	RCOR1	-0.4783	0.3151	REST corepressor 1
14149	RCL1	-0.4783	0.1548	RNA terminal phosphate cyclase-like 1
14150	PPP2R2D	-0.4783	0.0687	protein phosphatase 2, regulatory subunit B, delta
14151	KPTN	-0.4783	0.1384	kaptin (actin binding protein)
14152	INSL6	-0.4783	0.1774	insulin-like 6
14153	DAGLA	-0.4783	0.1756	diacylglycerol lipase, alpha
14154	CLTA	-0.4783	0.2488	clathrin, light chain A
14155	CACNA1G	-0.4783	0.3998	calcium channel, voltage-dependent, T type, alpha 1G subunit
14156	BNIP3	-0.4783	0.2019	BCL2/adenovirus E1B 19kDa interacting protein 3
14157	AK4	-0.4783	0.9999	adenylate kinase 4
14158	TMCO5A	-0.4787	0.2444	transmembrane and coiled-coil domains 5A
14159	PREX2	-0.4787	0.1275	phosphatidylinositol-3,4,5-trisphosphate-dependent Rac exchange factor 2
14160	NKAPP1	-0.4787	0.0636	NFKB activating protein pseudogene 1
14161	C1orf127	-0.4787	0.3965	chromosome 1 open reading frame 127
14162	ASB2	-0.4787	0.1503	ankyrin repeat and SOCS box containing 2
14163	TUBB2A	-0.4792	0.1262	tubulin, beta 2A class IIa
14164	RPS20	-0.4792	0.158	ribosomal protein S20
14165	NLGN4X	-0.4792	0.0479	neuroligin 4, X-linked
14166	NEUROD6	-0.4792	0.3485	neuronal differentiation 6
14167	MTERFD1	-0.4792	0.3561	MTERF domain containing 1
14168	ITM2C	-0.4792	0.0846	integral membrane protein 2C
14169	DDX4	-0.4792	0.1128	DEAD (Asp-Glu-Ala-Asp) box polypeptide 4
14170	BRIP1	-0.4792	0.2479	BRCA1 interacting protein C-terminal helicase 1
14171	SLC4A3	-0.48	0.1971	solute carrier family 4, anion exchanger, member 3
14172	RAB11FIP4	-0.48	0.1065	RAB11 family interacting protein 4 (class II)
14173	PPIH	-0.48	0.3024	peptidylprolyl isomerase H (cyclophilin H)
14174	NRCAM	-0.48	0.0593	neuronal cell adhesion molecule
14175	MTPAP	-0.48	0.3354	mitochondrial poly(A) polymerase
14176	CDC25B	-0.48	0.1582	cell division cycle 25 homolog B (S. pombe)
14177	C7orf13	-0.48	0.0267	chromosome 7 open reading frame 13
14178	C18orf62	-0.48	0.2549	chromosome 18 open reading frame 62
14179	APBB1IP	-0.48	0.1407	amyloid beta (A4) precursor protein-binding, family B, member 1 interacting protein
14180	SRSF3	-0.4808	0.3718	serine/arginine-rich splicing factor 3
14181	SNCB	-0.4808	0.3529	synuclein, beta
14182	PTPRZ1	-0.4808	0.062	protein tyrosine phosphatase, receptor-type, Z polypeptide 1
14183	PHF21A	-0.4808	0.1683	PHD finger protein 21A
14184	PACS2	-0.4808	0.2024	phosphofurin acidic cluster sorting protein 2
14185	NEURL	-0.4808	0.3108	neuralized homolog (Drosophila)
14186	GSK3A	-0.4808	0.2351	glycogen synthase kinase 3 alpha
14187	DDT	-0.4808	0.2195	D-dopachrome tautomerase
14188	ANKRD27	-0.4808	0.2803	ankyrin repeat domain 27 (VPS9 domain)
14189	ADAM7	-0.4808	0.3123	ADAM metallopeptidase domain 7
14190	STARD9	-0.4812	0.1249	StAR-related lipid transfer (START) domain containing 9
14191	MBOAT1	-0.4812	0.1043	membrane bound O-acyltransferase domain containing 1
14192	LINC00202	-0.4812	0.228	long intergenic non-protein coding RNA 202
14193	DACH2	-0.4812	0.0752	dachshund homolog 2 (Drosophila)
14194	ACOT12	-0.4812	0.2111	acyl-CoA thioesterase 12

14195	LOC144742	-0.4814	0.9999	uncharacterized LOC144742
14196	MKKS	-0.4817	0.3145	McKusick-Kaufman syndrome
14197	METAP2	-0.4817	0.3617	methionyl aminopeptidase 2
14198	IL23A	-0.4817	0.1309	interleukin 23, alpha subunit p19
14199	FAM178A	-0.4817	0.2858	family with sequence similarity 178, member A
14200	FAM173A	-0.4817	0.2327	family with sequence similarity 173, member A
14201	DFFB	-0.4817	0.191	DNA fragmentation factor, 40kDa, beta polypeptide (caspase-activated DNase)
14202	TBCD	-0.4825	0.1666	tubulin folding cofactor D
14203	NOXRED1	-0.4825	0.1115	NADP-dependent oxidoreductase domain containing 1
14204	FAM168B	-0.4825	0.1703	family with sequence similarity 168, member B
14205	EXO1	-0.4825	0.2454	exonuclease 1
14206	CCER1	-0.4825	0.2543	coiled-coil glutamate-rich protein 1
14207	BOLA3	-0.4825	0.2756	bolA homolog 3 (E. coli)
14208	OR51M1	-0.4829	0.358	olfactory receptor, family 51, subfamily M, member 1
14209	TBKBP1	-0.4833	0.3346	TBK1 binding protein 1
14210	TAF1C	-0.4833	0.1853	TATA box binding protein (TBP)-associated factor, RNA polymerase I, C, 110kDa
14211	SAG	-0.4833	0.3199	S-antigen; retina and pineal gland (arrestin)
14212	PAGE1	-0.4833	0.1962	P antigen family, member 1 (prostate associated)
14213	MPHOSPH6	-0.4833	0.2487	M-phase phosphoprotein 6
14214	GRM2	-0.4833	0.3664	glutamate receptor, metabotropic 2
14215	EIF2C1	-0.4833	0.088	eukaryotic translation initiation factor 2C, 1
14216	CPLX2	-0.4833	0.3614	complexin 2
14217	APOA1	-0.4833	0.3293	apolipoprotein A-I
14218	ADAM30	-0.4833	0.3339	ADAM metallopeptidase domain 30
14219	LINC00261	-0.4837	0.1053	long intergenic non-protein coding RNA 261
14220	FLJ25328	-0.4837	0.2941	uncharacterized LOC148231
14221	FAM159A	-0.4837	0.1527	family with sequence similarity 159, member A
14222	C2orf29	-0.4837	0.2314	chromosome 2 open reading frame 29
14223	C19orf48	-0.4837	0.2417	chromosome 19 open reading frame 48
14224	C16orf93	-0.4837	0.1253	chromosome 16 open reading frame 93
14225	TNPO3	-0.4842	0.1772	transportin 3
14226	TMEM132A	-0.4842	0.2214	transmembrane protein 132A
14227	STK10	-0.4842	0.1773	serine/threonine kinase 10
14228	SART3	-0.4842	0.3127	squamous cell carcinoma antigen recognized by T cells 3
14229	SAMM50	-0.4842	0.2931	sorting and assembly machinery component 50 homolog (S. cerevisiae)
14230	RDBP	-0.4842	0.2295	RD RNA binding protein
14231	RASGRF1	-0.4842	0.2883	Ras protein-specific guanine nucleotide-releasing factor 1
14232	NGB	-0.4842	0.3503	neuroglobin
14233	DIABLO	-0.4842	0.2218	diablo, IAP-binding mitochondrial protein
14234	CORO1C	-0.4842	0.2445	coronin, actin binding protein, 1C
14235	PARD6G-AS1	-0.4843	0.9999	PARD6G antisense RNA 1 (non-protein coding)
14236	UPF1	-0.485	0.155	UPF1 regulator of nonsense transcripts homolog (yeast)
14237	SNRNP200	-0.485	0.2704	small nuclear ribonucleoprotein 200kDa (U5)
14238	SLC29A4	-0.485	0.1472	solute carrier family 29 (nucleoside transporters), member 4
14239	RFPL1	-0.485	0.0917	ret finger protein-like 1
14240	RBFOX1	-0.485	0.3592	RNA binding protein, fox-1 homolog (C. elegans) 1
14241	PRR18	-0.485	0.2396	proline rich 18
14242	LIM2	-0.485	0.3402	lens intrinsic membrane protein 2, 19kDa
14243	HBA2	-0.485	0.0391	hemoglobin, alpha 2
14244	GLUD2	-0.485	0.0533	glutamate dehydrogenase 2
14245	DPH3	-0.485	0.294	DPH3, KTI11 homolog (S. cerevisiae)
14246	ATXN10	-0.485	0.2968	ataxin 10
14247	ANKRD36BP2	-0.485	0.0673	ankyrin repeat domain 36B pseudogene 2
14248	LOC283731	-0.4857	0.9996	uncharacterized LOC283731
14249	SLC6A1	-0.4858	0.1274	solute carrier family 6 (neurotransmitter transporter, GABA), member 1
14250	RASL10A	-0.4858	0.3082	RAS-like, family 10, member A
14251	PLXNC1	-0.4858	0.1272	plexin C1
14252	FAM131A	-0.4858	0.077	family with sequence similarity 131, member A
14253	DR1	-0.4858	0.3311	down-regulator of transcription 1, TBP-binding (negative cofactor 2)
14254	CRYZL1	-0.4858	0.3306	crystallin, zeta (quinone reductase)-like 1
14255	CHMP7	-0.4858	0.1055	charged multivesicular body protein 7
14256	ATG3	-0.4858	0.3321	autophagy related 3
14257	YDJC	-0.4863	0.2245	YdjC homolog (bacterial)
14258	TTC25	-0.4863	0.0699	tetratricopeptide repeat domain 25
14259	TMCO7	-0.4863	0.0716	transmembrane and coiled-coil domains 7
14260	PALD1	-0.4863	0.0813	phosphatase domain containing, paladin 1
14261	LLPH	-0.4863	0.2922	LLP homolog, long-term synaptic facilitation (Aplysia)
14262	GCET2	-0.4863	0.0917	germinal center expressed transcript 2
14263	FLVCR1-AS1	-0.4863	0.0498	FLVCR1 antisense RNA 1 (non-protein coding)
14264	CXorf22	-0.4863	0.0425	chromosome X open reading frame 22
14265	FAM205B	-0.4864	0.3532	transmembrane protein C9orf144B pseudogene
14266	PRCD	-0.4867	0.9996	progressive rod-cone degeneration
14267	HIPK1	-0.4867	0.1638	homeodomain interacting protein kinase 1
14268	GPR17	-0.4867	0.3321	G protein-coupled receptor 17
14269	GAR1	-0.4867	0.3098	GAR1 ribonucleoprotein homolog (yeast)
14270	DSCC1	-0.4867	0.2509	defective in sister chromatid cohesion 1 homolog (S. cerevisiae)
14271	CBX2	-0.4867	0.258	chromobox homolog 2
14272	LOC147646	-0.4871	0.9999	uncharacterized LOC147646
14273	C19orf81	-0.4871	0.2448	chromosome 19 open reading frame 81
14274	ZNF706	-0.4873	0.2415	zinc finger protein 706
14275	TRMT1L	-0.4875	0.3357	tRNA methyltransferase 1 homolog (S. cerevisiae)-like
14276	SLC9B2	-0.4875	0.0664	solute carrier family 9, subfamily B (NHA2, cation proton antiporter 2), member 2
14277	RNF38	-0.4875	0.2691	ring finger protein 38
14278	RNF19B	-0.4875	0.1235	ring finger protein 19B

14279	GHRLOS	-0.4875	0.202	ghrelin opposite strand/antisense RNA (non-protein coding)
14280	FNTA	-0.4875	0.338	farnesyltransferase, CAAX box, alpha
14281	ATP6V0B	-0.4875	0.2036	ATPase, H+ transporting, lysosomal 21kDa, V0 subunit b
14282	SH2D1A	-0.4883	0.1678	SH2 domain containing 1A
14283	PWP1	-0.4883	0.3326	PWP1 homolog (S. cerevisiae)
14284	PPP3CC	-0.4883	0.2012	protein phosphatase 3, catalytic subunit, gamma isozyme
14285	HMGA1	-0.4883	0.2038	high mobility group AT-hook 1
14286	ASIC2	-0.4883	0.3124	acid-sensing (proton-gated) ion channel 2
14287	ST7-OT4	-0.4886	0.9996	ST7 overlapping transcript 4 (non-protein coding)
14288	GAB4	-0.4886	0.412	GRB2-associated binding protein family, member 4
14289	CLNK	-0.4886	0.2464	cytokine-dependent hematopoietic cell linker
14290	ZNF687	-0.4888	0.124	zinc finger protein 687
14291	MRPS23	-0.4888	0.3146	mitochondrial ribosomal protein S23
14292	FIZ1	-0.4888	0.2723	FLT3-interacting zinc finger 1
14293	BRD7	-0.4891	0.296	bromodomain containing 7
14294	TLX3	-0.4892	0.2238	T-cell leukemia homeobox 3
14295	TIAL1	-0.4892	0.1879	TIA1 cytotoxic granule-associated RNA binding protein-like 1
14296	SLC5A6	-0.4892	0.1693	solute carrier family 5 (sodium-dependent vitamin transporter), member 6
14297	ZNF281	-0.49	0.2183	zinc finger protein 281
14298	TCF20	-0.49	0.0999	transcription factor 20 (AR1)
14299	SSBP3	-0.49	0.0694	single stranded DNA binding protein 3
14300	SNRPD3	-0.49	0.2928	small nuclear ribonucleoprotein D3 polypeptide 18kDa
14301	RSRC1	-0.49	0.2662	arginine/serine-rich coiled-coil 1
14302	NUDT17	-0.49	0.2158	nudix (nucleoside diphosphate linked moiety X)-type motif 17
14303	MTMR7	-0.49	0.0441	myotubularin related protein 7
14304	LEPROTL1	-0.49	0.2965	leptin receptor overlapping transcript-like 1
14305	C20orf78	-0.49	0.3595	chromosome 20 open reading frame 78
14306	BARD1	-0.49	0.2603	BRCA1 associated RING domain 1
14307	AQP10	-0.49	0.217	aquaporin 10
14308	AIF1	-0.49	0.2214	allograft inflammatory factor 1
14309	USP14	-0.4908	0.3214	ubiquitin specific peptidase 14 (tRNA-guanine transglycosylase)
14310	UBAP2	-0.4908	0.1892	ubiquitin associated protein 2
14311	TRNAU1AP	-0.4908	0.0875	tRNA selenocysteine 1 associated protein 1
14312	SNAP29	-0.4908	0.1203	synaptosomal-associated protein, 29kDa
14313	PTP4A3	-0.4908	0.1144	protein tyrosine phosphatase type IVA, member 3
14314	PRKAR1B	-0.4908	0.2449	protein kinase, cAMP-dependent, regulatory, type I, beta
14315	POLR2G	-0.4908	0.2799	polymerase (RNA) II (DNA directed) polypeptide G
14316	PLP1	-0.4908	0.0663	proteolipid protein 1
14317	NRXN2	-0.4908	0.2528	neurexin 2
14318	IAPP	-0.4908	0.1163	islet amyloid polypeptide
14319	DNA2	-0.4908	0.2501	DNA replication helicase 2 homolog (yeast)
14320	CD300A	-0.4908	0.1912	CD300a molecule
14321	LSMD1	-0.4913	0.2092	LSM domain containing 1
14322	GPC2	-0.4913	0.1997	glypican 2
14323	HBZ	-0.4914	0.0756	hemoglobin, zeta
14324	CEBPG	-0.4917	0.2432	CCAAT/enhancer binding protein (C/EBP), gamma
14325	CDC40	-0.4917	0.2848	cell division cycle 40 homolog (S. cerevisiae)
14326	C19orf53	-0.4917	0.2314	chromosome 19 open reading frame 53
14327	C16orf53	-0.4917	0.1648	chromosome 16 open reading frame 53
14328	ATP5S	-0.4917	0.1669	ATP synthase, H+ transporting, mitochondrial Fo complex, subunit s (factor B)
14329	ADCY5	-0.4917	0.1334	adenylate cyclase 5
14330	ZNF383	-0.4925	0.2349	zinc finger protein 383
14331	SLC16A8	-0.4925	0.3631	solute carrier family 16, member 8 (monocarboxylic acid transporter 3)
14332	QKI	-0.4925	0.2401	QKI, KH domain containing, RNA binding
14333	PSMC4	-0.4925	0.2506	proteasome (prosome, macropain) 26S subunit, ATPase, 4
14334	MYT1	-0.4925	0.3192	myelin transcription factor 1
14335	MRGPRX4	-0.4925	0.2827	MAS-related GPR, member X4
14336	MFNG	-0.4925	0.1302	MFNG O-fucosylpeptide 3-beta-N-acetylglucosaminyltransferase
14337	MAFG	-0.4925	0.1053	v-maf musculoaponeurotic fibrosarcoma oncogene homolog G (avian)
14338	LRGUK	-0.4925	0.0531	leucine-rich repeats and guanylate kinase domain containing
14339	KRTAP7-1	-0.4925	0.2129	keratin associated protein 7-1 (gene/pseudogene)
14340	ISY1	-0.4925	0.1429	ISY1 splicing factor homolog (S. cerevisiae)
14341	FRRS1L	-0.4925	0.1633	ferric-chelate reductase 1-like
14342	FAM9B	-0.4925	0.0346	family with sequence similarity 9, member B
14343	CHKA	-0.4925	0.0706	choline kinase alpha
14344	AGAP3	-0.4925	0.1911	ArfGAP with GTPase domain, ankyrin repeat and PH domain 3
14345	LOC100129361	-0.4927	0.3003	chromosome X open reading frame 69 pseudogene
14346	C10orf113	-0.4929	0.0236	chromosome 10 open reading frame 113
14347	TSSC1	-0.4933	0.2021	tumor suppressing subtransferable candidate 1
14348	SOX11	-0.4933	0.1293	SRY (sex determining region Y)-box 11
14349	RABL6	-0.4933	0.2039	RAB, member RAS oncogene family-like 6
14350	HAUS3	-0.4933	0.3174	HAUS augmin-like complex, subunit 3
14351	GPSM2	-0.4933	0.2315	G-protein signaling modulator 2
14352	CD2BP2	-0.4933	0.1715	CD2 (cytoplasmic tail) binding protein 2
14353	B3GAT3	-0.4933	0.1726	beta-1,3-glucuronyltransferase 3 (glucuronosyltransferase I)
14354	ACTL7A	-0.4933	0.3439	actin-like 7A
14355	TRMT44	-0.4938	0.0422	tRNA methyltransferase 44 homolog (S. cerevisiae)
14356	TMEM31	-0.4938	0.1227	transmembrane protein 31
14357	PIGX	-0.4938	0.2454	phosphatidylinositol glycan anchor biosynthesis, class X
14358	OR10A4	-0.4938	0.207	olfactory receptor, family 10, subfamily A, member 4
14359	IL18BP	-0.4938	0.1665	interleukin 18 binding protein
14360	IGSF21	-0.4938	0.1068	immunoglobin superfamily, member 21
14361	HES4	-0.4938	0.2215	hairy and enhancer of split 4 (Drosophila)
14362	GPR98	-0.4938	0.1054	G protein-coupled receptor 98

14363	PDIA2	-0.4942	0.2503	protein disulfide isomerase family A, member 2
14364	LBX1	-0.4942	0.3101	ladybird homeobox 1
14365	BATF3	-0.4942	0.1303	basic leucine zipper transcription factor, ATF-like 3
14366	LINC00520	-0.4943	0.2474	long intergenic non-protein coding RNA 520
14367	SEC31B	-0.495	0.175	SEC31 homolog B (S. cerevisiae)
14368	POMC	-0.495	0.2765	proopiomelanocortin
14369	ORC5	-0.495	0.3205	origin recognition complex, subunit 5
14370	NUDT10	-0.495	0.121	nudix (nucleoside diphosphate linked moiety X)-type motif 10
14371	NANOS3	-0.495	0.349	nanos homolog 3 (Drosophila)
14372	MED30	-0.495	0.2315	mediator complex subunit 30
14373	HNRNPUL1	-0.495	0.185	heterogeneous nuclear ribonucleoprotein U-like 1
14374	HMGN3	-0.495	0.2841	high mobility group nucleosomal binding domain 3
14375	CNGA4	-0.495	0.1204	cyclic nucleotide gated channel alpha 4
14376	CLEC2L	-0.495	0.1534	C-type lectin domain family 2, member L
14377	ARIH2	-0.495	0.137	ariadne homolog 2 (Drosophila)
14378	ANP32A	-0.495	0.3004	acidic (leucine-rich) nuclear phosphoprotein 32 family, member A
14379	LOC401463	-0.4957	0.9996	uncharacterized LOC401463
14380	GNB1	-0.4958	0.2137	guanine nucleotide binding protein (G protein), beta polypeptide 1
14381	TRAPPC6B	-0.4963	0.3141	trafficking protein particle complex 6B
14382	TAS2R39	-0.4963	0.1725	taste receptor, type 2, member 39
14383	SNHG12	-0.4963	0.192	small nucleolar RNA host gene 12 (non-protein coding)
14384	NAA30	-0.4963	0.279	N(alpha)-acetyltransferase 30, NatC catalytic subunit
14385	LOC729059	-0.4963	0.1437	uncharacterized LOC729059
14386	COL24A1	-0.4963	0.0612	collagen, type XXIV, alpha 1
14387	C16orf78	-0.4963	0.3566	chromosome 16 open reading frame 78
14388	ACTL9	-0.4963	0.2233	actin-like 9
14389	UCK2	-0.4967	0.235	uridine-cytidine kinase 2
14390	TRPM8	-0.4967	0.1025	transient receptor potential cation channel, subfamily M, member 8
14391	EWSR1	-0.4967	0.1818	Ewing sarcoma breakpoint region 1
14392	ANKRD40	-0.4967	0.1093	ankyrin repeat domain 40
14393	PWRN2	-0.4971	0.9996	Prader-Willi region non-protein coding RNA 2
14394	ZNF577	-0.4975	0.1057	zinc finger protein 577
14395	SLC17A6	-0.4975	0.1163	solute carrier family 17 (sodium-dependent inorganic phosphate cotransporter), member 6
14396	RASD2	-0.4975	0.1283	RASD family, member 2
14397	PSMB1	-0.4975	0.3208	proteasome (prosome, macropain) subunit, beta type, 1
14398	PRPF38A	-0.4975	0.2886	PRP38 pre-mRNA processing factor 38 (yeast) domain containing A
14399	HAVCR2	-0.4975	0.1618	hepatitis A virus cellular receptor 2
14400	GCAT	-0.4975	0.2108	glycine C-acetyltransferase
14401	GAD2	-0.4975	0.3087	glutamate decarboxylase 2 (pancreatic islets and brain, 65kDa)
14402	CGRRF1	-0.4975	0.2503	cell growth regulator with ring finger domain 1
14403	CEL	-0.4975	0.1447	carboxyl ester lipase (bile salt-stimulated lipase)
14404	CDC14C	-0.4975	0.0145	CDC14 cell division cycle 14 homolog C (S. cerevisiae)
14405	C17orf85	-0.4975	0.2372	chromosome 17 open reading frame 85
14406	C14orf178	-0.4975	0.2851	chromosome 14 open reading frame 178
14407	ADCK5	-0.4975	0.2403	aarF domain containing kinase 5
14408	ACTR5	-0.4975	0.1367	ARP5 actin-related protein 5 homolog (yeast)
14409	NT5M	-0.4983	0.239	5',3'-nucleotidase, mitochondrial
14410	INSL3	-0.4983	0.3493	insulin-like 3 (Leydig cell)
14411	GLT25D2	-0.4983	0.0349	glycosyltransferase 25 domain containing 2
14412	CHD7	-0.4983	0.1283	chromodomain helicase DNA binding protein 7
14413	BUB3	-0.4983	0.3648	budding uninhibited by benzimidazoles 3 homolog (yeast)
14414	ACSBG1	-0.4983	0.0504	acyl-CoA synthetase bubblegum family member 1
14415	SBF2-AS1	-0.4986	0.9996	SBF2 antisense RNA 1 (non-protein coding)
14416	TBC1D21	-0.4988	0.3314	TBC1 domain family, member 21
14417	GUCA1C	-0.4988	0.1107	guanylate cyclase activator 1C
14418	FAM71E1	-0.4988	0.201	family with sequence similarity 71, member E1
14419	URM1	-0.4992	0.1818	ubiquitin related modifier 1
14420	TOP3B	-0.4992	0.1183	topoisomerase (DNA) III beta
14421	MAPRE2	-0.4992	0.0899	microtubule-associated protein, RP/EB family, member 2
14422	HIST1H4L	-0.4992	0.0838	histone cluster 1, H4l
14423	FBXO2	-0.4992	0.173	F-box protein 2
14424	CADM3	-0.4992	0.3746	cell adhesion molecule 3
14425	ZNF607	-0.5	0.1311	zinc finger protein 607
14426	ZNF213	-0.5	0.2216	zinc finger protein 213
14427	TMCC2	-0.5	0.2563	transmembrane and coiled-coil domain family 2
14428	RBBP7	-0.5	0.3231	retinoblastoma binding protein 7
14429	POM121L12	-0.5	0.4153	POM121 transmembrane nucleoporin-like 12
14430	LOC100133612	-0.5	0.9999	uncharacterized LOC100133612
14431	KCNT1	-0.5	0.2047	potassium channel, subfamily T, member 1
14432	GATS	-0.5	0.1136	GATS, stromal antigen 3 opposite strand
14433	CAMTA1	-0.5	0.047	calmodulin binding transcription activator 1
14434	ZNF688	-0.5008	0.2535	zinc finger protein 688
14435	TCEAL2	-0.5008	0.0496	transcription elongation factor A (SII)-like 2
14436	GAS2	-0.5008	0.0672	growth arrest-specific 2
14437	FARP2	-0.5008	0.074	FERM, RhoGEF and pleckstrin domain protein 2
14438	CLNS1A	-0.5008	0.3122	chloride channel, nucleotide-sensitive, 1A
14439	PHF13	-0.5013	0.0873	PHD finger protein 13
14440	GJD3	-0.5013	0.3756	gap junction protein, delta 3, 31.9kDa
14441	DSCAML1	-0.5013	0.2703	Down syndrome cell adhesion molecule like 1
14442	C10orf82	-0.5013	0.2039	chromosome 10 open reading frame 82
14443	C10orf53	-0.5013	0.3379	chromosome 10 open reading frame 53
14444	ANKRD13B	-0.5013	0.1977	ankyrin repeat domain 13B
14445	LINC00301	-0.5014	0.2673	long intergenic non-protein coding RNA 301
14446	HYALP1	-0.5014	0.9999	hyaluronoglucosaminidase pseudogene 1

14447	FOLH1B	-0.5014	0.0126	folate hydrolase 1B
14448	SPEF1	-0.5017	0.3552	sperm flagellar 1
14449	RMI1	-0.5017	0.3346	RMI1, RecQ mediated genome instability 1, homolog (S. cerevisiae)
14450	PRM2	-0.5017	0.3011	protamine 2
14451	NLGN3	-0.5017	0.3484	neuroligin 3
14452	LRRC40	-0.5017	0.3655	leucine rich repeat containing 40
14453	LARGE	-0.5017	0.0572	like-glycosyltransferase
14454	WDFY4	-0.5025	0.2336	WDFY family member 4
14455	SPOCK2	-0.5025	0.1668	sparc/osteonectin, cwcv and kazal-like domains proteoglycan (testican) 2
14456	PTPRN2	-0.5025	0.0818	protein tyrosine phosphatase, receptor type, N polypeptide 2
14457	PCNXL3	-0.5025	0.1786	pecanex-like 3 (Drosophila)
14458	MED10	-0.5025	0.15	mediator complex subunit 10
14459	CEP135	-0.5025	0.2664	centrosomal protein 135kDa
14460	C9orf131	-0.5025	0.3125	chromosome 9 open reading frame 131
14461	ACTRT2	-0.5025	0.3649	actin-related protein T2
14462	COX8C	-0.5029	0.051	cytochrome c oxidase subunit VIIIC
14463	PAK7	-0.5033	0.1853	p21 protein (Cdc42/Rac)-activated kinase 7
14464	HDAC5	-0.5033	0.2002	histone deacetylase 5
14465	FCHSD2	-0.5033	0.2129	FCH and double SH3 domains 2
14466	BCORL1	-0.5033	0.1777	BCL6 corepressor-like 1
14467	ATP5F1	-0.5033	0.336	ATP synthase, H+ transporting, mitochondrial Fo complex, subunit B1
14468	ASUN	-0.5033	0.3523	asunder, spermatogenesis regulator homolog (Drosphila)
14469	MRPL35	-0.5036	0.3009	mitochondrial ribosomal protein L35
14470	HAR1A	-0.5038	0.089	highly accelerated region 1A (non-protein coding)
14471	BTLA	-0.5038	0.1051	B and T lymphocyte associated
14472	ALYREF	-0.5038	0.2498	Aly/REF export factor
14473	ADAMTS18	-0.5038	0.2	ADAM metallopeptidase with thrombospondin type 1 motif, 18
14474	RPA1	-0.5042	0.3034	replication protein A1, 70kDa
14475	PAXIP1	-0.5042	0.3072	PAX interacting (with transcription-activation domain) protein 1
14476	HIST1H3C	-0.5042	0.0651	histone cluster 1, H3c
14477	HCFC1	-0.5042	0.1689	host cell factor C1 (VP16-accessory protein)
14478	GABRD	-0.5042	0.3026	gamma-aminobutyric acid (GABA) A receptor, delta
14479	CASP8AP2	-0.5042	0.3509	caspase 8 associated protein 2
14480	LOC388882	-0.5043	0.3488	uncharacterized LOC388882
14481	SPATA24	-0.505	0.0791	spermatogenesis associated 24
14482	KIAA1210	-0.505	0.0977	KIAA1210
14483	HOXC12	-0.505	0.262	homeobox C12
14484	FATE1	-0.505	0.2666	fetal and adult testis expressed 1
14485	EFCAB12	-0.505	0.1208	EF-hand calcium binding domain 12
14486	DHX34	-0.505	0.222	DEAH (Asp-Glu-Ala-His) box polypeptide 34
14487	AFG3L2	-0.505	0.1891	AFG3 ATPase family gene 3-like 2 (S. cerevisiae)
14488	TOMM22	-0.5058	0.2138	translocase of outer mitochondrial membrane 22 homolog (yeast)
14489	GMEB1	-0.5058	0.0777	glucocorticoid modulatory element binding protein 1
14490	GABARAPL2	-0.5058	0.2261	GABA(A) receptor-associated protein-like 2
14491	FXN	-0.5058	0.1958	frataxin
14492	C22orf43	-0.5058	0.0586	chromosome 22 open reading frame 43
14493	LOC100126784	-0.5063	0.205	uncharacterized LOC100126784
14494	KLK9	-0.5063	0.3702	kallikrein-related peptidase 9
14495	FLJ25758	-0.5063	0.2399	MAP/microtubule affinity-regulating kinase 1 pseudogene
14496	DEFB129	-0.5063	0.1344	defensin, beta 129
14497	SASH3	-0.5067	0.2223	SAM and SH3 domain containing 3
14498	PRG2	-0.5067	0.1304	proteoglycan 2, bone marrow (natural killer cell activator, eosinophil granule major basic protein)
14499	NDUFA6	-0.5067	0.2675	NADH dehydrogenase (ubiquinone) 1 alpha subcomplex, 6, 14kDa
14500	NCAPD2	-0.5067	0.2291	non-SMC condensin I complex, subunit D2
14501	CD37	-0.5067	0.1919	CD37 molecule
14502	FLJ41350	-0.5071	0.3138	uncharacterized LOC399806
14503	ZNF385B	-0.5075	0.0399	zinc finger protein 385B
14504	TMEM180	-0.5075	0.0911	transmembrane protein 180
14505	SLC7A6OS	-0.5075	0.053	solute carrier family 7, member 6 opposite strand
14506	S100B	-0.5075	0.0381	S100 calcium binding protein B
14507	PTPN7	-0.5075	0.1612	protein tyrosine phosphatase, non-receptor type 7
14508	NRTN	-0.5075	0.1059	neurturin
14509	NDST3	-0.5075	0.0791	N-deacetylase/N-sulfotransferase (heparan glucosaminyl) 3
14510	MESP1	-0.5075	0.1651	mesoderm posterior 1 homolog (mouse)
14511	KLHL7	-0.5075	0.307	kelch-like 7 (Drosophila)
14512	ENOPH1	-0.5075	0.3533	enolase-phosphatase 1
14513	CISD1	-0.5075	0.2386	CDGSH iron sulfur domain 1
14514	CDYL2	-0.5075	0.1173	chromodomain protein, Y-like 2
14515	ASB10	-0.5075	0.3889	ankyrin repeat and SOCS box containing 10
14516	ZNF764	-0.5083	0.1154	zinc finger protein 764
14517	PRR3	-0.5083	0.09	proline rich 3
14518	PGS1	-0.5083	0.1029	phosphatidylglycerophosphate synthase 1
14519	NACAD	-0.5083	0.1945	NAC alpha domain containing
14520	KANSL2	-0.5083	0.2906	KAT8 regulatory NSL complex subunit 2
14521	DNM2	-0.5083	0.1814	dynamin 2
14522	DLL3	-0.5083	0.2311	delta-like 3 (Drosophila)
14523	OR52D1	-0.5086	0.3056	olfactory receptor, family 52, subfamily D, member 1
14524	LINC00488	-0.5086	0.9996	long intergenic non-protein coding RNA 488
14525	THAP7-AS1	-0.5088	0.1512	THAP7 antisense RNA 1 (non-protein coding)
14526	TEX35	-0.5088	0.2167	testis expressed 35
14527	LOC84989	-0.5088	0.1216	uncharacterized LOC84989
14528	IQCF1	-0.5088	0.1902	IQ motif containing F1
14529	CREB3L3	-0.5088	0.1471	cAMP responsive element binding protein 3-like 3
14530	WDYHV1	-0.5092	0.252	WDYHV motif containing 1

14531	TNIK	-0.5092	0.049	TRAF2 and NCK interacting kinase
14532	SCD	-0.5092	0.0561	stearoyl-CoA desaturase (delta-9-desaturase)
14533	MEN1	-0.5092	0.1898	multiple endocrine neoplasia I
14534	FKBP4	-0.5092	0.1722	FK506 binding protein 4, 59kDa
14535	EPB49	-0.5092	0.1615	erythrocyte membrane protein band 4.9 (dematin)
14536	CYP46A1	-0.5092	0.2498	cytochrome P450, family 46, subfamily A, polypeptide 1
14537	C2CD2L	-0.5092	0.0951	C2CD2-like
14538	ZBTB11	-0.51	0.3452	zinc finger and BTB domain containing 11
14539	VHL	-0.51	0.0557	von Hippel-Lindau tumor suppressor, E3 ubiquitin protein ligase
14540	USH1G	-0.51	0.3623	Usher syndrome 1G (autosomal recessive)
14541	TTTY12	-0.51	0.1843	testis-specific transcript, Y-linked 12 (non-protein coding)
14542	TRMT12	-0.51	0.187	tRNA methyltransferase 12 homolog (S. cerevisiae)
14543	TMEM199	-0.51	0.1656	transmembrane protein 199
14544	PP13	-0.51	0.9999	uncharacterized LOC100129503
14545	POLR3C	-0.51	0.248	polymerase (RNA) III (DNA directed) polypeptide C (62kD)
14546	NTAN1	-0.51	0.1364	N-terminal asparagine amidase
14547	MVD	-0.51	0.1981	mevalonate (diphospho) decarboxylase
14548	MTRF1	-0.51	0.257	mitochondrial translational release factor 1
14549	MPND	-0.51	0.1789	MPN domain containing
14550	LSG1	-0.51	0.2242	large subunit GTPase 1 homolog (S. cerevisiae)
14551	LINC00543	-0.51	0.1433	long intergenic non-protein coding RNA 543
14552	IL27RA	-0.51	0.1045	interleukin 27 receptor, alpha
14553	GPSM3	-0.51	0.3074	G-protein signaling modulator 3
14554	FAM90A1	-0.51	0.0488	family with sequence similarity 90, member A1
14555	PSMB4	-0.5108	0.285	proteasome (prosome, macropain) subunit, beta type, 4
14556	PFKL	-0.5108	0.2011	phosphofructokinase, liver
14557	NRL	-0.5108	0.3066	neural retina leucine zipper
14558	COPS8	-0.5108	0.2973	COP9 constitutive photomorphogenic homolog subunit 8 (Arabidopsis)
14559	ZNF569	-0.5113	0.2212	zinc finger protein 569
14560	WFDC3	-0.5113	0.2115	WAP four-disulfide core domain 3
14561	LRTM2	-0.5113	0.2808	leucine-rich repeats and transmembrane domains 2
14562	ISLR2	-0.5113	0.2409	immunoglobulin superfamily containing leucine-rich repeat 2
14563	CARD11	-0.5113	0.1419	caspase recruitment domain family, member 11
14564	C7orf62	-0.5113	0.2716	chromosome 7 open reading frame 62
14565	C10orf90	-0.5113	0.192	chromosome 10 open reading frame 90
14566	PSORS1C3	-0.5114	0.0289	psoriasis susceptibility 1 candidate 3 (non-protein coding)
14567	PCGEM1	-0.5114	0.9996	prostate-specific transcript 1 (non-protein coding)
14568	REEP2	-0.5117	0.2931	receptor accessory protein 2
14569	HIST1H4A	-0.5117	0.0398	histone cluster 1, H4a
14570	TSPYL4	-0.5125	0.235	TSPY-like 4
14571	TOP2B	-0.5125	0.3391	topoisomerase (DNA) II beta 180kDa
14572	THOC5	-0.5125	0.1568	THO complex 5
14573	SMCHD1	-0.5125	0.3133	structural maintenance of chromosomes flexible hinge domain containing 1
14574	PNN	-0.5125	0.3311	pinin, desmosome associated protein
14575	NTN3	-0.5125	0.3106	netrin 3
14576	MOG	-0.5125	0.3015	myelin oligodendrocyte glycoprotein
14577	GPS1	-0.5125	0.2379	G protein pathway suppressor 1
14578	CEP63	-0.5125	0.2985	centrosomal protein 63kDa
14579	CCDC63	-0.5125	0.1999	coiled-coil domain containing 63
14580	ZMYND10	-0.5133	0.32	zinc finger, MYND-type containing 10
14581	RNGTT	-0.5133	0.2469	RNA guanylyltransferase and 5'-phosphatase
14582	MED28	-0.5133	0.2765	mediator complex subunit 28
14583	LILRP2	-0.5133	0.2699	leukocyte immunoglobulin-like receptor pseudogene 2
14584	INTS7	-0.5133	0.283	integrator complex subunit 7
14585	HNRNPAB	-0.5133	0.3077	heterogeneous nuclear ribonucleoprotein A/B
14586	DYRK1B	-0.5133	0.3089	dual-specificity tyrosine-(Y)-phosphorylation regulated kinase 1B
14587	CC2D1A	-0.5133	0.2274	coiled-coil and C2 domain containing 1A
14588	BAZ1B	-0.5133	0.2328	bromodomain adjacent to zinc finger domain, 1B
14589	ZNF57	-0.5138	0.1251	zinc finger protein 57
14590	ZNF546	-0.5138	0.0798	zinc finger protein 546
14591	VN1R2	-0.5138	0.2231	vomeronasal 1 receptor 2
14592	SPERT	-0.5138	0.2329	spermatid associated
14593	SLFNL1	-0.5138	0.3129	schlafen-like 1
14594	NPW	-0.5138	0.1302	neuropeptide W
14595	GAB3	-0.5138	0.109	GRB2-associated binding protein 3
14596	FAM118B	-0.5138	0.1872	family with sequence similarity 118, member B
14597	ASB12	-0.5138	0.1737	ankyrin repeat and SOCS box containing 12
14598	ZMYND8	-0.5142	0.1305	zinc finger, MYND-type containing 8
14599	TRAPPC4	-0.5142	0.2596	trafficking protein particle complex 4
14600	PRDM10	-0.5142	0.1511	PR domain containing 10
14601	PAAF1	-0.5142	0.2052	proteasomal ATPase-associated factor 1
14602	NUP188	-0.5142	0.1638	nucleoporin 188kDa
14603	MAPKAPK5-AS1	-0.5142	0.1399	MAPKAPK5 antisense RNA 1 (non-protein coding)
14604	INPP5E	-0.5142	0.1074	inositol polyphosphate-5-phosphatase, 72 kDa
14605	GMPR	-0.5142	0.0406	guanosine monophosphate reductase
14606	CINP	-0.5142	0.1973	cyclin-dependent kinase 2 interacting protein
14607	RNF5	-0.5143	0.0942	ring finger protein 5, E3 ubiquitin protein ligase
14608	GMNC	-0.5143	0.9996	geminin coiled-coil domain containing
14609	TUFM	-0.515	0.2329	Tu translation elongation factor, mitochondrial
14610	TMOD4	-0.515	0.1111	tropomodulin 4 (muscle)
14611	TLX2	-0.515	0.3572	T-cell leukemia homeobox 2
14612	SPATA2L	-0.515	0.1585	spermatogenesis associated 2-like
14613	RALYL	-0.515	0.1337	RALY RNA binding protein-like
14614	LOC150197	-0.515	0.3276	uncharacterized LOC150197

14615	LOC100131496	-0.515	0.1318	uncharacterized LOC100131496
14616	GNRH2	-0.515	0.3795	gonadotropin-releasing hormone 2
14617	GLYCTK	-0.515	0.2244	glycerate kinase
14618	GLS2	-0.515	0.0815	glutaminase 2 (liver, mitochondrial)
14619	GAMT	-0.515	0.2305	guanidinoacetate N-methyltransferase
14620	FOXJ3	-0.515	0.2244	forkhead box J3
14621	FBXL12	-0.515	0.0719	F-box and leucine-rich repeat protein 12
14622	DMRT2	-0.515	0.0359	doublesex and mab-3 related transcription factor 2
14623	ASCC1	-0.515	0.2357	activating signal cointegrator 1 complex subunit 1
14624	ACRBP	-0.515	0.1614	acrosin binding protein
14625	SHISA8	-0.5157	0.0643	shisa homolog 8 (Xenopus laevis)
14626	C20orf203	-0.5157	0.3379	chromosome 20 open reading frame 203
14627	SLC1A3	-0.5158	0.0432	solute carrier family 1 (glial high affinity glutamate transporter), member 3
14628	PRPS2	-0.5158	0.3029	phosphoribosyl pyrophosphate synthetase 2
14629	NUP155	-0.5158	0.3048	nucleoporin 155kDa
14630	CSNK2A2	-0.5158	0.099	casein kinase 2, alpha prime polypeptide
14631	ACRV1	-0.5158	0.3318	acrosomal vesicle protein 1
14632	SYTL5	-0.5163	0.0202	synaptotagmin-like 5
14633	MEX3A	-0.5163	0.2015	mex-3 homolog A (C. elegans)
14634	LRRC18	-0.5163	0.1397	leucine rich repeat containing 18
14635	BRMS1L	-0.5163	0.2766	breast cancer metastasis-suppressor 1-like
14636	SUN2	-0.5167	0.1297	Sad1 and UNC84 domain containing 2
14637	RSBN1	-0.5167	0.2837	round spermatid basic protein 1
14638	ODZ1	-0.5167	0.0224	odz, odd Oz/ten-m homolog 1 (Drosophila)
14639	NR6A1	-0.5167	0.3101	nuclear receptor subfamily 6, group A, member 1
14640	NPC1	-0.5167	0.1432	Niemann-Pick disease, type C1
14641	AKIRIN1	-0.5167	0.1625	akirin 1
14642	WDR46	-0.5175	0.217	WD repeat domain 46
14643	RAD51	-0.5175	0.2	RAD51 homolog (S. cerevisiae)
14644	RAC3	-0.5175	0.1812	ras-related C3 botulinum toxin substrate 3 (rho family, small GTP binding protein Rac3)
14645	PARD6A	-0.5175	0.2071	par-6 partitioning defective 6 homolog alpha (C. elegans)
14646	NYAP1	-0.5175	0.3204	neuronal tyrosine-phosphorylated phosphoinositide-3-kinase adaptor 1
14647	LCP2	-0.5175	0.1676	lymphocyte cytosolic protein 2 (SH2 domain containing leukocyte protein of 76kDa)
14648	CSRNP2	-0.5175	0.128	cysteine-serine-rich nuclear protein 2
14649	ANKLE1	-0.5175	0.1069	ankyrin repeat and LEM domain containing 1
14650	HNRNPC	-0.5182	0.3351	heterogeneous nuclear ribonucleoprotein C (C1/C2)
14651	PPP4R4	-0.5183	0.0471	protein phosphatase 4, regulatory subunit 4
14652	PNOC	-0.5183	0.0984	prepronociceptin
14653	METTL22	-0.5183	0.0749	methyltransferase like 22
14654	LPPR1	-0.5183	0.0926	lipid phosphate phosphatase-related protein type 1
14655	CA14	-0.5183	0.227	carbonic anhydrase XIV
14656	ACP1	-0.5183	0.2973	acid phosphatase 1, soluble
14657	ZFYVE27	-0.5188	0.0876	zinc finger, FYVE domain containing 27
14658	TRABD2A	-0.5188	0.0591	TraB domain containing 2A
14659	TMEM151A	-0.5188	0.2856	transmembrane protein 151A
14660	CCL26	-0.5188	0.0751	chemokine (C-C motif) ligand 26
14661	C5orf47	-0.5188	0.0979	chromosome 5 open reading frame 47
14662	C4orf48	-0.5188	0.1358	chromosome 4 open reading frame 48
14663	ZIM2	-0.5192	0.2583	zinc finger, imprinted 2
14664	C21orf91	-0.5192	0.1763	chromosome 21 open reading frame 91
14665	C14orf166	-0.5192	0.3132	chromosome 14 open reading frame 166
14666	AKAP6	-0.5192	0.1815	A kinase (PRKA) anchor protein 6
14667	ZNF536	-0.52	0.0968	zinc finger protein 536
14668	UCK1	-0.52	0.1036	uridine-cytidine kinase 1
14669	SLC35D3	-0.52	0.0221	solute carrier family 35, member D3
14670	SCAF1	-0.52	0.2676	SR-related CTD-associated factor 1
14671	PRM3	-0.52	0.342	protamine 3
14672	PAGE5	-0.52	0.1913	P antigen family, member 5 (prostate associated)
14673	NSUN5	-0.52	0.1905	NOP2/Sun domain family, member 5
14674	KRT222	-0.52	0.0347	keratin 222
14675	KIAA1383	-0.52	0.0374	KIAA1383
14676	GIGYF1	-0.52	0.2474	GRB10 interacting GYF protein 1
14677	DICER1-AS1	-0.52	0.1164	DICER1 antisense RNA 1 (non-protein coding)
14678	CHST10	-0.52	0.0758	carbohydrate sulfotransferase 10
14679	APBB1	-0.52	0.1761	amyloid beta (A4) precursor protein-binding, family B, member 1 (Fe65)
14680	AMIGO3	-0.52	0.233	adhesion molecule with Ig-like domain 3
14681	TK1	-0.5208	0.2166	thymidine kinase 1, soluble
14682	SLC16A1	-0.5208	0.1519	solute carrier family 16, member 1 (monocarboxylic acid transporter 1)
14683	HMBS	-0.5208	0.1994	hydroxymethylbilane synthase
14684	CDH22	-0.5208	0.3068	cadherin 22, type 2
14685	LOC152024	-0.5213	0.084	uncharacterized LOC152024
14686	IGSF5	-0.5213	0.0732	immunoglobulin superfamily, member 5
14687	LOC339568	-0.5214	0.9996	uncharacterized LOC339568
14688	LINC00460	-0.5214	0.9996	long intergenic non-protein coding RNA 460
14689	C10orf112	-0.5214	0.9996	chromosome 10 open reading frame 112
14690	WDR48	-0.5217	0.1523	WD repeat domain 48
14691	USE1	-0.5217	0.1395	unconventional SNARE in the ER 1 homolog (S. cerevisiae)
14692	PTPN2	-0.5217	0.2507	protein tyrosine phosphatase, non-receptor type 2
14693	LRRC14	-0.5217	0.1126	leucine rich repeat containing 14
14694	CPSF7	-0.5217	0.1251	cleavage and polyadenylation specific factor 7, 59kDa
14695	TMPRSS12	-0.5225	0.1272	transmembrane (C-terminal) protease, serine 12
14696	SOLH	-0.5225	0.1905	small optic lobes homolog (Drosophila)
14697	SF3A3	-0.5225	0.239	splicing factor 3a, subunit 3, 60kDa
14698	RNF26	-0.5225	0.1641	ring finger protein 26

14699	PAQR6	-0.5225	0.1231	progestin and adipoQ receptor family member VI
14700	LSM14B	-0.5225	0.0651	LSM14B, SCD6 homolog B (S. cerevisiae)
14701	KLHL26	-0.5225	0.0645	kelch-like 26 (Drosophila)
14702	CEBPZ	-0.5225	0.311	CCAAT/enhancer binding protein (C/EBP), zeta
14703	BEND2	-0.5225	0.037	BEN domain containing 2
14704	TIPARP-AS1	-0.5229	0.9999	TIPARP antisense RNA 1 (non-protein coding)
14705	PP2672	-0.5233	0.9999	uncharacterized LOC100130249
14706	MAPK6	-0.5233	0.2467	mitogen-activated protein kinase 6
14707	KCNB2	-0.5233	0.3274	potassium voltage-gated channel, Shab-related subfamily, member 2
14708	ITGAL	-0.5233	0.1625	integrin, alpha L (antigen CD11A (p180), lymphocyte function-associated antigen 1; alpha polypeptide)
14709	DPP6	-0.5233	0.1771	dipeptidyl-peptidase 6
14710	CSK	-0.5233	0.1603	c-src tyrosine kinase
14711	ANAPC5	-0.5233	0.2439	anaphase promoting complex subunit 5
14712	TRIM42	-0.5238	0.2051	tripartite motif containing 42
14713	TDH	-0.5238	0.1865	L-threonine dehydrogenase
14714	RIIAD1	-0.5238	0.1285	regulatory subunit of type II PKA R-subunit (RIIa) domain containing 1
14715	DYDC2	-0.5238	0.0406	DPY30 domain containing 2
14716	CLEC4G	-0.5238	0.1306	C-type lectin domain family 4, member G
14717	ZWINT	-0.5242	0.2989	ZW10 interactor
14718	TUBB4B	-0.5242	0.2071	tubulin, beta 4B class IVb
14719	C18orf1	-0.5242	0.074	chromosome 18 open reading frame 1
14720	ARMC1	-0.5242	0.3483	armadillo repeat containing 1
14721	UBAP1L	-0.525	0.9996	ubiquitin associated protein 1-like
14722	TULP2	-0.525	0.3176	tubby like protein 2
14723	TCFL5	-0.525	0.1891	transcription factor-like 5 (basic helix-loop-helix)
14724	TAGLN3	-0.525	0.0844	transgelin 3
14725	RPA3	-0.525	0.2669	replication protein A3, 14kDa
14726	PYY2	-0.525	0.3903	peptide YY, 2 (pseudogene)
14727	POLR3H	-0.525	0.1574	polymerase (RNA) III (DNA directed) polypeptide H (22.9kD)
14728	P2RX7	-0.525	0.073	purinergic receptor P2X, ligand-gated ion channel, 7
14729	OR8D1	-0.525	0.1482	olfactory receptor, family 8, subfamily D, member 1
14730	NEUROD2	-0.525	0.3157	neuronal differentiation 2
14731	MARCH4	-0.525	0.1243	membrane-associated ring finger (C3HC4) 4, E3 ubiquitin protein ligase
14732	LGI3	-0.525	0.2742	leucine-rich repeat LGI family, member 3
14733	DCAF12	-0.525	0.0715	DDB1 and CUL4 associated factor 12
14734	CELF6	-0.525	0.1324	CUGBP, Elav-like family member 6
14735	CCDC57	-0.525	0.2168	coiled-coil domain containing 57
14736	MFHAS1	-0.5258	0.0513	malignant fibrous histiocytoma amplified sequence 1
14737	LIN7B	-0.5258	0.1049	lin-7 homolog B (C. elegans)
14738	ISCA1	-0.5258	0.2158	iron-sulfur cluster assembly 1 homolog (S. cerevisiae)
14739	GRAP2	-0.5258	0.1474	GRB2-related adaptor protein 2
14740	FAM64A	-0.5258	0.1897	family with sequence similarity 64, member A
14741	CRBN	-0.5258	0.251	cereblon
14742	CNR1	-0.5258	0.1014	cannabinoid receptor 1 (brain)
14743	C21orf59	-0.5258	0.2543	chromosome 21 open reading frame 59
14744	ATP2B2	-0.5258	0.3202	ATPase, Ca++ transporting, plasma membrane 2
14745	WDR64	-0.5263	0.1304	WD repeat domain 64
14746	UBL7	-0.5263	0.1584	ubiquitin-like 7 (bone marrow stromal cell-derived)
14747	SLC6A17	-0.5263	0.3225	solute carrier family 6, member 17
14748	RCSD1	-0.5263	0.1266	RCSD domain containing 1
14749	OR8D2	-0.5263	0.2332	olfactory receptor, family 8, subfamily D, member 2
14750	MMP21	-0.5263	0.0943	matrix metallopeptidase 21
14751	LINC00608	-0.5263	0.2549	long intergenic non-protein coding RNA 608
14752	GPHA2	-0.5263	0.2874	glycoprotein hormone alpha 2
14753	DOCK8	-0.5263	0.1386	dedicator of cytokinesis 8
14754	SRSF7	-0.5267	0.325	serine/arginine-rich splicing factor 7
14755	SELPLG	-0.5267	0.2166	selectin P ligand
14756	FXYD7	-0.5267	0.2604	FXYD domain containing ion transport regulator 7
14757	ELAVL3	-0.5267	0.3338	ELAV (embryonic lethal, abnormal vision, Drosophila)-like 3 (Hu antigen C)
14758	NT5DC4	-0.5271	0.1555	5'-nucleotidase domain containing 4
14759	LOC648987	-0.5271	0.0488	uncharacterized LOC648987
14760	WBP2	-0.5275	0.1422	WW domain binding protein 2
14761	SPINK2	-0.5275	0.0465	serine peptidase inhibitor, Kazal type 2 (acrosin-trypsin inhibitor)
14762	SGTB	-0.5275	0.1877	small glutamine-rich tetratricopeptide repeat (TPR)-containing, beta
14763	SF3B3	-0.5275	0.2011	splicing factor 3b, subunit 3, 130kDa
14764	SCAP	-0.5275	0.1754	SREBF chaperone
14765	NUP93	-0.5275	0.2338	nucleoporin 93kDa
14766	MPEG1	-0.5275	0.1425	macrophage expressed 1
14767	MAPT	-0.5275	0.269	microtubule-associated protein tau
14768	MAP7D1	-0.5275	0.1821	MAP7 domain containing 1
14769	GPBAR1	-0.5275	0.3001	G protein-coupled bile acid receptor 1
14770	GGH	-0.5275	0.2425	gamma-glutamyl hydrolase (conjugase, folylpolygammaglutamyl hydrolase)
14771	GABRB1	-0.5275	0.0632	gamma-aminobutyric acid (GABA) A receptor, beta 1
14772	FAM117A	-0.5275	0.0858	family with sequence similarity 117, member A
14773	ENDOG	-0.5275	0.194	endonuclease G
14774	CMTM2	-0.5275	0.107	CKLF-like MARVEL transmembrane domain containing 2
14775	CACNA2D4	-0.5275	0.1515	calcium channel, voltage-dependent, alpha 2/delta subunit 4
14776	C7orf34	-0.5275	0.2853	chromosome 7 open reading frame 34
14777	RRP7A	-0.5283	0.1792	ribosomal RNA processing 7 homolog A (S. cerevisiae)
14778	LOC100128993	-0.5283	0.9999	uncharacterized LOC100128993
14779	KIAA1467	-0.5283	0.0382	KIAA1467
14780	GDF9	-0.5283	0.1913	growth differentiation factor 9
14781	ARL6IP1	-0.5283	0.2982	ADP-ribosylation factor-like 6 interacting protein 1
14782	LINC00523	-0.5286	0.3847	long intergenic non-protein coding RNA 523

14783	ZACN	-0.5288	0.0756	zinc activated ligand-gated ion channel
14784	UCN3	-0.5288	0.3351	urocortin 3 (stresscopin)
14785	NFATC2	-0.5288	0.0625	nuclear factor of activated T-cells, cytoplasmic, calcineurin-dependent 2
14786	NEU4	-0.5288	0.3447	sialidase 4
14787	LRRC25	-0.5288	0.2331	leucine rich repeat containing 25
14788	CSTL1	-0.5288	0.0801	cystatin-like 1
14789	CCDC158	-0.5288	0.0717	coiled-coil domain containing 158
14790	C20orf196	-0.5288	0.0425	chromosome 20 open reading frame 196
14791	C20orf152	-0.5288	0.206	chromosome 20 open reading frame 152
14792	ADH4	-0.5288	0.0365	alcohol dehydrogenase 4 (class II), pi polypeptide
14793	SYP	-0.5292	0.2305	synaptophysin
14794	SEZ6L2	-0.5292	0.1517	seizure related 6 homolog (mouse)-like 2
14795	PANK2	-0.5292	0.1967	pantothenate kinase 2
14796	CHCHD2	-0.5292	0.2063	coiled-coil-helix-coiled-coil-helix domain containing 2
14797	STAU2-AS1	-0.53	0.9999	STAU2 antisense RNA 1 (non-protein coding)
14798	SLC25A53	-0.53	0.0763	solute carrier family 25, member 53
14799	RPL38	-0.53	0.1472	ribosomal protein L38
14800	PTPN23	-0.53	0.2042	protein tyrosine phosphatase, non-receptor type 23
14801	PTGDR2	-0.53	0.3008	prostaglandin D2 receptor 2
14802	P2RX3	-0.53	0.1818	purinergic receptor P2X, ligand-gated ion channel, 3
14803	MFSD6L	-0.53	0.2249	major facilitator superfamily domain containing 6-like
14804	LOC100130458	-0.53	0.9999	uncharacterized LOC100130458
14805	C18orf34	-0.53	0.0958	chromosome 18 open reading frame 34
14806	APC2	-0.53	0.3577	adenomatosis polyposis coli 2
14807	TSSK2	-0.5308	0.2568	testis-specific serine kinase 2
14808	TATDN2	-0.5308	0.058	TatD DNase domain containing 2
14809	SLC25A44	-0.5308	0.0939	solute carrier family 25, member 44
14810	SCRIB	-0.5308	0.1493	scribbled homolog (Drosophila)
14811	SCG5	-0.5308	0.0276	secretogranin V (7B2 protein)
14812	MGAT3	-0.5308	0.2819	mannosyl (beta-1,4-)-glycoprotein beta-1,4-N-acetylglucosaminyltransferase
14813	IGFLR1	-0.5308	0.1342	IGF-like family receptor 1
14814	GLTSCR1	-0.5308	0.1856	glioma tumor suppressor candidate region gene 1
14815	ELMO1	-0.5308	0.0523	engulfment and cell motility 1
14816	TRMT5	-0.5309	0.2519	tRNA methyltransferase 5 homolog (S. cerevisiae)
14817	SLCO6A1	-0.5313	0.073	solute carrier organic anion transporter family, member 6A1
14818	PCDHAC1	-0.5313	0.125	protocadherin alpha subfamily C, 1
14819	MON1A	-0.5313	0.1196	MON1 homolog A (yeast)
14820	COL25A1	-0.5313	0.1326	collagen, type XXV, alpha 1
14821	C15orf59	-0.5313	0.1281	chromosome 15 open reading frame 59
14822	OR2L2	-0.5314	0.2078	olfactory receptor, family 2, subfamily L, member 2
14823	LOC284798	-0.5314	0.9996	uncharacterized LOC284798
14824	PDHX	-0.5317	0.3252	pyruvate dehydrogenase complex, component X
14825	NELL2	-0.5317	0.0454	NEL-like 2 (chicken)
14826	HIST1H4G	-0.5317	0.1165	histone cluster 1, H4g
14827	RBBP4	-0.5318	0.236	retinoblastoma binding protein 4
14828	SSNA1	-0.5325	0.2033	Sjogren syndrome nuclear autoantigen 1
14829	SNTG1	-0.5325	0.2047	syntrophin, gamma 1
14830	SMCR5	-0.5325	0.2207	Smith-Magenis syndrome chromosome region, candidate 5 (non-protein coding)
14831	PLA2G12B	-0.5325	0.2265	phospholipase A2, group XIIB
14832	MCOLN2	-0.5325	0.054	mucolipin 2
14833	FLJ35390	-0.5325	0.2334	uncharacterized LOC255031
14834	ERAL1	-0.5325	0.171	Era G-protein-like 1 (E. coli)
14835	CRTC2	-0.5325	0.1389	CREB regulated transcription coactivator 2
14836	C12orf76	-0.5325	0.0824	chromosome 12 open reading frame 76
14837	ANGEL1	-0.5325	0.0518	angel homolog 1 (Drosophila)
14838	RD3	-0.5329	0.2887	retinal degeneration 3
14839	OR14J1	-0.5329	0.2368	olfactory receptor, family 14, subfamily J, member 1
14840	LOC147727	-0.5329	0.0989	uncharacterized LOC147727
14841	INHBA-AS1	-0.5329	0.9996	INHBA antisense RNA 1 (non-protein coding)
14842	HTA	-0.5329	0.9996	uncharacterized LOC283902
14843	C5orf48	-0.5329	0.116	chromosome 5 open reading frame 48
14844	C10orf40	-0.5329	0.9996	chromosome 10 open reading frame 40
14845	WDHD1	-0.5333	0.2599	WD repeat and HMG-box DNA binding protein 1
14846	TOE1	-0.5333	0.1393	target of EGR1, member 1 (nuclear)
14847	TIMELESS	-0.5333	0.1747	timeless homolog (Drosophila)
14848	TCP11L1	-0.5333	0.0471	t-complex 11 (mouse)-like 1
14849	PPRC1	-0.5333	0.1948	peroxisome proliferator-activated receptor gamma, coactivator-related 1
14850	PCYT1B	-0.5333	0.1991	phosphate cytidylyltransferase 1, choline, beta
14851	NCAPD3	-0.5333	0.227	non-SMC condensin II complex, subunit D3
14852	MNT	-0.5333	0.1158	MAX binding protein
14853	CHRNA6	-0.5333	0.1637	cholinergic receptor, nicotinic, alpha 6 (neuronal)
14854	TMED6	-0.5337	0.0164	transmembrane emp24 protein transport domain containing 6
14855	SYT9	-0.5337	0.2267	synaptotagmin IX
14856	SLC7A14	-0.5337	0.1847	solute carrier family 7 (orphan transporter), member 14
14857	MCHR2	-0.5337	0.2818	melanin-concentrating hormone receptor 2
14858	FAM57B	-0.5337	0.3187	family with sequence similarity 57, member B
14859	CCDC114	-0.5337	0.2071	coiled-coil domain containing 114
14860	CATSPERD	-0.5337	0.0463	catsper channel auxiliary subunit delta
14861	C1orf194	-0.5337	0.1907	chromosome 1 open reading frame 194
14862	TSKS	-0.5342	0.3064	testis-specific serine kinase substrate
14863	SENP2	-0.5342	0.1182	SUMO1/sentrin/SMT3 specific peptidase 2
14864	PSMB6	-0.5342	0.2363	proteasome (prosome, macropain) subunit, beta type, 6
14865	MED24	-0.5342	0.1776	mediator complex subunit 24
14866	LRRC20	-0.5342	0.1191	leucine rich repeat containing 20

14867	FANCG	-0.5342	0.1973	Fanconi anemia, complementation group G
14868	PSMC5	-0.535	0.2607	proteasome (prosome, macropain) 26S subunit, ATPase, 5
14869	NOP9	-0.535	0.0642	NOP9 nucleolar protein homolog (yeast)
14870	NOC4L	-0.535	0.2018	nucleolar complex associated 4 homolog (S. cerevisiae)
14871	LCA5L	-0.535	0.0889	Leber congenital amaurosis 5-like
14872	GPR113	-0.535	0.258	G protein-coupled receptor 113
14873	ELFN1	-0.535	0.108	extracellular leucine-rich repeat and fibronectin type III domain containing 1
14874	ANKRD33	-0.535	0.2643	ankyrin repeat domain 33
14875	TRIM67	-0.5357	0.3632	tripartite motif containing 67
14876	LOC728819	-0.5357	0.0344	hCG1645220
14877	LOC285768	-0.5357	0.2228	uncharacterized LOC285768
14878	FLJ44342	-0.5357	0.1166	uncharacterized LOC645460
14879	TRAFD1	-0.5358	0.0577	TRAF-type zinc finger domain containing 1
14880	RIMBP2	-0.5358	0.1033	RIMS binding protein 2
14881	MCAT	-0.5358	0.1711	malonyl CoA:ACP acyltransferase (mitochondrial)
14882	ZNF295-AS1	-0.5362	0.2523	ZNF295 antisense RNA 1 (non-protein coding)
14883	NAPSB	-0.5362	0.1568	napsin B aspartic peptidase pseudogene
14884	IQCF5	-0.5362	0.1478	IQ motif containing F5
14885	CCDC172	-0.5362	0.178	coiled-coil domain containing 172
14886	TUBA1C	-0.5367	0.2071	tubulin, alpha 1c
14887	RAD54B	-0.5367	0.2018	RAD54 homolog B (S. cerevisiae)
14888	NCKAP1L	-0.5367	0.137	NCK-associated protein 1-like
14889	CYLC1	-0.5367	0.1699	cylicin, basic protein of sperm head cytoskeleton 1
14890	APBA2	-0.5367	0.0541	amyloid beta (A4) precursor protein-binding, family A, member 2
14891	THEMIS	-0.5371	0.0941	thymocyte selection associated
14892	PWRN1	-0.5371	0.9996	Prader-Willi region non-protein coding RNA 1
14893	MYH16	-0.5371	0.2238	myosin, heavy chain 16 pseudogene
14894	TRMT10A	-0.5375	0.2134	tRNA methyltransferase 10 homolog A (S. cerevisiae)
14895	RUVBL1	-0.5375	0.2791	RuvB-like 1 (E. coli)
14896	PRMT8	-0.5375	0.2942	protein arginine methyltransferase 8
14897	POLR2A	-0.5375	0.0829	polymerase (RNA) II (DNA directed) polypeptide A, 220kDa
14898	MRGPRX2	-0.5375	0.1507	MAS-related GPR, member X2
14899	MGC34800	-0.5375	0.3722	uncharacterized protein MGC34800
14900	MCTS1	-0.5375	0.2251	malignant T cell amplified sequence 1
14901	MASTL	-0.5375	0.2633	microtubule associated serine/threonine kinase-like
14902	IMPA1	-0.5375	0.3111	inositol(myo)-1(or 4)-monophosphatase 1
14903	FLVCR1	-0.5375	0.2059	feline leukemia virus subgroup C cellular receptor 1
14904	ZNF408	-0.5383	0.1795	zinc finger protein 408
14905	PSMB5	-0.5383	0.2322	proteasome (prosome, macropain) subunit, beta type, 5
14906	AARS	-0.5383	0.1293	alanyl-tRNA synthetase
14907	ZNF789	-0.5387	0.1254	zinc finger protein 789
14908	TMEM143	-0.5387	0.1057	transmembrane protein 143
14909	PYCR2	-0.5387	0.1304	pyrroline-5-carboxylate reductase family, member 2
14910	LELP1	-0.5387	0.2795	late cornified envelope-like proline-rich 1
14911	FOXA3	-0.5387	0.0838	forkhead box A3
14912	ENTHD2	-0.5387	0.1502	ENTH domain containing 2
14913	CCDC60	-0.5387	0.2958	coiled-coil domain containing 60
14914	ASH1L-AS1	-0.5387	0.0703	ASH1L antisense RNA 1 (non-protein coding)
14915	CES3	-0.5391	0.1512	carboxylesterase 3
14916	SCRN1	-0.5392	0.0693	secernin 1
14917	RAB3A	-0.5392	0.1773	RAB3A, member RAS oncogene family
14918	MIR600HG	-0.5392	0.1168	MIR600 host gene (non-protein coding)
14919	DOCK2	-0.5392	0.1288	dedicator of cytokinesis 2
14920	CTDP1	-0.5392	0.1456	CTD (carboxy-terminal domain, RNA polymerase II, polypeptide A) phosphatase, subunit 1
14921	CLGN	-0.5392	0.0431	calmegin
14922	XK	-0.54	0.047	X-linked Kx blood group (McLeod syndrome)
14923	SLITRK1	-0.54	0.1384	SLIT and NTRK-like family, member 1
14924	SLC25A14	-0.54	0.0739	solute carrier family 25 (mitochondrial carrier, brain), member 14
14925	RHOG	-0.54	0.1959	ras homolog family member G
14926	PRSS54	-0.54	0.2258	protease, serine, 54
14927	NAP1L1	-0.54	0.3066	nucleosome assembly protein 1-like 1
14928	H2BFM	-0.54	0.2698	H2B histone family, member M
14929	GEN1	-0.54	0.2222	Gen endonuclease homolog 1 (Drosophila)
14930	FAM179A	-0.54	0.128	family with sequence similarity 179, member A
14931	ACTR3B	-0.54	0.1381	ARP3 actin-related protein 3 homolog B (yeast)
14932	PASK	-0.5408	0.1072	PAS domain containing serine/threonine kinase
14933	NOL4	-0.5408	0.059	nucleolar protein 4
14934	DNAJC6	-0.5408	0.0434	DnaJ (Hsp40) homolog, subfamily C, member 6
14935	DEAF1	-0.5408	0.1476	deformed epidermal autoregulatory factor 1 (Drosophila)
14936	ANP32B	-0.5408	0.2463	acidic (leucine-rich) nuclear phosphoprotein 32 family, member B
14937	SLC4A1AP	-0.5412	0.1971	solute carrier family 4 (anion exchanger), member 1, adaptor protein
14938	MARCH8	-0.5412	0.0366	membrane-associated ring finger (C3HC4) 8, E3 ubiquitin protein ligase
14939	FBXL16	-0.5412	0.0729	F-box and leucine-rich repeat protein 16
14940	FAM40A	-0.5412	0.0662	family with sequence similarity 40, member A
14941	DNAH12	-0.5412	0.1024	dynein, axonemal, heavy chain 12
14942	C20orf202	-0.5412	0.197	chromosome 20 open reading frame 202
14943	BTBD9	-0.5412	0.0732	BTB (POZ) domain containing 9
14944	ADAT2	-0.5412	0.1478	adenosine deaminase, tRNA-specific 2
14945	LOC100287598	-0.5414	0.9999	uncharacterized LOC100287598
14946	C7orf71	-0.5414	0.2475	chromosome 7 open reading frame 71
14947	UBE2C	-0.5417	0.2186	ubiquitin-conjugating enzyme E2C
14948	RALGDS	-0.5417	0.1153	ral guanine nucleotide dissociation stimulator
14949	NDOR1	-0.5417	0.3583	NADPH dependent diflavin oxidoreductase 1
14950	LRRC61	-0.5417	0.1016	leucine rich repeat containing 61

14951	LRFN3	-0.5417	0.0885	leucine rich repeat and fibronectin type III domain containing 3
14952	SPATA8	-0.5425	0.2603	spermatogenesis associated 8
14953	SERGEF	-0.5425	0.0928	secretion regulating guanine nucleotide exchange factor
14954	RAP1GAP2	-0.5425	0.0257	RAP1 GTPase activating protein 2
14955	PHF6	-0.5425	0.2401	PHD finger protein 6
14956	NUDCD2	-0.5425	0.3048	NudC domain containing 2
14957	MS4A6E	-0.5425	0.0394	membrane-spanning 4-domains, subfamily A, member 6E
14958	MRPL51	-0.5425	0.2311	mitochondrial ribosomal protein L51
14959	MNF1	-0.5425	0.2026	mitochondrial nucleoid factor 1
14960	LINC00052	-0.5425	0.1657	long intergenic non-protein coding RNA 52
14961	ITPKA	-0.5425	0.1401	inositol-trisphosphate 3-kinase A
14962	C11orf85	-0.5425	0.0645	chromosome 11 open reading frame 85
14963	ZNF883	-0.5429	0.032	zinc finger protein 883
14964	SPAG6	-0.5433	0.0616	sperm associated antigen 6
14965	SELRC1	-0.5433	0.1265	Sel1 repeat containing 1
14966	KCNJ4	-0.5433	0.2913	potassium inwardly-rectifying channel, subfamily J, member 4
14967	C2orf44	-0.5433	0.1307	chromosome 2 open reading frame 44
14968	ACHE	-0.5433	0.2772	acetylcholinesterase
14969	MS4A8B	-0.5437	0.0899	membrane-spanning 4-domains, subfamily A, member 8B
14970	HCN3	-0.5437	0.1451	hyperpolarization activated cyclic nucleotide-gated potassium channel 3
14971	GPR26	-0.5437	0.2948	G protein-coupled receptor 26
14972	GABRG1	-0.5437	0.0548	gamma-aminobutyric acid (GABA) A receptor, gamma 1
14973	FBRSL1	-0.5437	0.1555	fibrosin-like 1
14974	CCDC42	-0.5437	0.2315	coiled-coil domain containing 42
14975	AFG3L1P	-0.5437	0.1118	AFG3 ATPase family gene 3-like 1 (S. cerevisiae), pseudogene
14976	ST18	-0.5442	0.0318	suppression of tumorigenicity 18 (breast carcinoma) (zinc finger protein)
14977	SOD1	-0.5442	0.2461	superoxide dismutase 1, soluble
14978	GHSR	-0.5442	0.3396	growth hormone secretagogue receptor
14979	CCR10	-0.5442	0.102	chemokine (C-C motif) receptor 10
14980	RCC1	-0.5443	0.1791	regulator of chromosome condensation 1
14981	ZNF444	-0.545	0.2468	zinc finger protein 444
14982	USP20	-0.545	0.1164	ubiquitin specific peptidase 20
14983	POLR1B	-0.545	0.2789	polymerase (RNA) I polypeptide B, 128kDa
14984	PMFBP1	-0.545	0.1402	polyamine modulated factor 1 binding protein 1
14985	NRGN	-0.545	0.0953	neurogranin (protein kinase C substrate, RC3)
14986	MSL1	-0.545	0.1364	male-specific lethal 1 homolog (Drosophila)
14987	MBD3L1	-0.545	0.2447	methyl-CpG binding domain protein 3-like 1
14988	LYRM7	-0.545	0.2812	Lyrm7 homolog (mouse)
14989	GSG1L	-0.545	0.2773	GSG1-like
14990	FAM105B	-0.545	0.0598	family with sequence similarity 105, member B
14991	C16orf46	-0.545	0.025	chromosome 16 open reading frame 46
14992	BCCIP	-0.545	0.3173	BRCA2 and CDKN1A interacting protein
14993	ADAMTSL2	-0.545	0.2525	ADAMTS-like 2
14994	ZRANB2-AS1	-0.5457	0.9999	ZRANB2 antisense RNA 1 (non-protein coding)
14995	PNPLA6	-0.5458	0.1747	patatin-like phospholipase domain containing 6
14996	GCFC1	-0.5458	0.2913	GC-rich sequence DNA-binding factor 1
14997	ELOVL2	-0.5458	0.0302	ELOVL fatty acid elongase 2
14998	HBG2	-0.546	0.0476	hemoglobin, gamma G
14999	TRNT1	-0.5462	0.2928	tRNA nucleotidyl transferase, CCA-adding, 1
15000	TPTE2P6	-0.5462	0.0209	transmembrane phosphoinositide 3-phosphatase and tensin homolog 2 pseudogene 6
15001	RBM33	-0.5462	0.1004	RNA binding motif protein 33
15002	C1orf226	-0.5462	0.0244	chromosome 1 open reading frame 226
15003	ANKRD60	-0.5462	0.3285	ankyrin repeat domain 60
15004	OPA1	-0.5467	0.2923	optic atrophy 1 (autosomal dominant)
15005	LINC00599	-0.5467	0.3112	long intergenic non-protein coding RNA 599
15006	GABRA1	-0.5467	0.2777	gamma-aminobutyric acid (GABA) A receptor, alpha 1
15007	C1orf159	-0.5467	0.207	chromosome 1 open reading frame 159
15008	PMCHL2	-0.5471	0.1023	pro-melanin-concentrating hormone-like 2, pseudogene
15009	C1RL-AS1	-0.5471	0.3365	C1RL antisense RNA 1 (non-protein coding)
15010	UCN	-0.5475	0.1608	urocortin
15011	TTC16	-0.5475	0.1695	tetratricopeptide repeat domain 16
15012	PTCD3	-0.5475	0.2392	pentatricopeptide repeat domain 3
15013	PIK3CD	-0.5475	0.1167	phosphatidylinositol-4,5-bisphosphate 3-kinase, catalytic subunit delta
15014	NDUFA3	-0.5475	0.1732	NADH dehydrogenase (ubiquinone) 1 alpha subcomplex, 3, 9kDa
15015	MEGF11	-0.5475	0.2292	multiple EGF-like-domains 11
15016	HNRNPH3	-0.5475	0.2912	heterogeneous nuclear ribonucleoprotein H3 (2H9)
15017	CREG2	-0.5475	0.0537	cellular repressor of E1A-stimulated genes 2
15018	C22orf39	-0.5475	0.0442	chromosome 22 open reading frame 39
15019	C19orf25	-0.5475	0.21	chromosome 19 open reading frame 25
15020	BRAF	-0.5475	0.083	v-raf murine sarcoma viral oncogene homolog B1
15021	ADIPOR2	-0.5475	0.0883	adiponectin receptor 2
15022	SUMO3	-0.5483	0.2007	SMT3 suppressor of mif two 3 homolog 3 (S. cerevisiae)
15023	SUGP2	-0.5483	0.1256	SURP and G patch domain containing 2
15024	SMG6	-0.5483	0.1196	smg-6 homolog, nonsense mediated mRNA decay factor (C. elegans)
15025	RNF4	-0.5483	0.1655	ring finger protein 4
15026	PCCB	-0.5483	0.2173	propionyl CoA carboxylase, beta polypeptide
15027	PAQR4	-0.5483	0.1379	progestin and adipoQ receptor family member IV
15028	MUM1	-0.5483	0.1038	melanoma associated antigen (mutated) 1
15029	ICAM4	-0.5483	0.0418	intercellular adhesion molecule 4 (Landsteiner-Wiener blood group)
15030	ZNF491	-0.5487	0.0674	zinc finger protein 491
15031	TSPAN10	-0.5487	0.1903	tetraspanin 10
15032	USP48	-0.5492	0.1996	ubiquitin specific peptidase 48
15033	TAF10	-0.5492	0.1795	TAF10 RNA polymerase II, TATA box binding protein (TBP)-associated factor, 30kDa
15034	SLC25A38	-0.5492	0.1027	solute carrier family 25, member 38

15035	NKIRAS2	-0.5492	0.111	NFKB inhibitor interacting Ras-like 2
15036	FYN	-0.5492	0.09	FYN oncogene related to SRC, FGR, YES
15037	COBRA1	-0.5492	0.1985	cofactor of BRCA1
15038	CENPQ	-0.5492	0.2893	centromere protein Q
15039	ADRBK1	-0.5492	0.1192	adrenergic, beta, receptor kinase 1
15040	RALY	-0.55	0.189	RNA binding protein, autoantigenic (hnRNP-associated with lethal yellow homolog (mouse))
15041	PFAS	-0.55	0.2275	phosphoribosylformylglycinamidine synthase
15042	OR2L1P	-0.55	0.9996	olfactory receptor, family 2, subfamily L, member 1 pseudogene
15043	OLFM3	-0.55	0.0349	olfactomedin 3
15044	LINC00620	-0.55	0.9996	long intergenic non-protein coding RNA 620
15045	LINC00479	-0.55	0.2746	long intergenic non-protein coding RNA 479
15046	ELMOD1	-0.55	0.0209	ELMO/CED-12 domain containing 1
15047	C1QTNF4	-0.55	0.1041	C1q and tumor necrosis factor related protein 4
15048	RRS1	-0.5508	0.2079	RRS1 ribosome biogenesis regulator homolog (S. cerevisiae)
15049	FXYD6	-0.5508	0.0683	FXYD domain containing ion transport regulator 6
15050	ZNF570	-0.5512	0.0443	zinc finger protein 570
15051	ZNF296	-0.5512	0.1623	zinc finger protein 296
15052	TCTE1	-0.5512	0.2768	t-complex-associated-testis-expressed 1
15053	NAA25	-0.5512	0.2465	N(alpha)-acetyltransferase 25, NatB auxiliary subunit
15054	C8orf46	-0.5512	0.0332	chromosome 8 open reading frame 46
15055	C15orf43	-0.5512	0.024	chromosome 15 open reading frame 43
15056	BRWD1-IT2	-0.5512	0.2176	BRWD1 intronic transcript 2 (non-protein coding)
15057	AMICA1	-0.5512	0.1239	adhesion molecule, interacts with CXADR antigen 1
15058	TRAF3IP3	-0.5517	0.104	TRAF3 interacting protein 3
15059	SLC2A6	-0.5517	0.1203	solute carrier family 2 (facilitated glucose transporter), member 6
15060	SLC1A2	-0.5517	0.1135	solute carrier family 1 (glial high affinity glutamate transporter), member 2
15061	PPP2R2B	-0.5517	0.0363	protein phosphatase 2, regulatory subunit B, beta
15062	EVI2A	-0.5517	0.1558	ecotropic viral integration site 2A
15063	C6orf106	-0.5517	0.0609	chromosome 6 open reading frame 106
15064	BEST1	-0.5517	0.2743	bestrophin 1
15065	AATF	-0.5517	0.1788	apoptosis antagonizing transcription factor
15066	THYN1	-0.5525	0.1892	thymocyte nuclear protein 1
15067	SNIP1	-0.5525	0.0913	Smad nuclear interacting protein 1
15068	SCN3B	-0.5525	0.2172	sodium channel, voltage-gated, type III, beta subunit
15069	OR51B2	-0.5525	0.1944	olfactory receptor, family 51, subfamily B, member 2
15070	HAPLN4	-0.5525	0.2428	hyaluronan and proteoglycan link protein 4
15071	EIF2S1	-0.5525	0.3024	eukaryotic translation initiation factor 2, subunit 1 alpha, 35kDa
15072	LUC7L	-0.5533	0.1125	LUC7-like (S. cerevisiae)
15073	LEF1	-0.5533	0.0223	lymphoid enhancer-binding factor 1
15074	EGR4	-0.5533	0.2305	early growth response 4
15075	ROPN1L	-0.5537	0.0701	rhophilin associated tail protein 1-like
15076	HORMAD2	-0.5537	0.1109	HORMA domain containing 2
15077	FBXW9	-0.5537	0.1917	F-box and WD repeat domain containing 9
15078	CD300LF	-0.5537	0.1654	CD300 molecule-like family member f
15079	BHLHE23	-0.5537	0.197	basic helix-loop-helix family, member e23
15080	SMG7	-0.5542	0.0683	smg-7 homolog, nonsense mediated mRNA decay factor (C. elegans)
15081	SLAMF1	-0.5542	0.1036	signaling lymphocytic activation molecule family member 1
15082	GFRA3	-0.5542	0.2294	GDNF family receptor alpha 3
15083	LOC645249	-0.5543	0.1217	uncharacterized LOC645249
15084	LOC286177	-0.5543	0.9996	uncharacterized LOC286177
15085	ZKSCAN4	-0.555	0.0615	zinc finger with KRAB and SCAN domains 4
15086	THAP7	-0.555	0.1575	THAP domain containing 7
15087	SACS	-0.555	0.2763	spastic ataxia of Charlevoix-Saguenay (sacsin)
15088	NUFIP1	-0.555	0.1877	nuclear fragile X mental retardation protein interacting protein 1
15089	HHIP-AS1	-0.555	0.0335	HHIP antisense RNA 1 (non-protein coding)
15090	CYFIP2	-0.555	0.0628	cytoplasmic FMR1 interacting protein 2
15091	CCM2	-0.555	0.1188	cerebral cavernous malformation 2
15092	C2orf53	-0.555	0.2999	chromosome 2 open reading frame 53
15093	ZWILCH	-0.5558	0.3044	Zwilch, kinetochore associated, homolog (Drosophila)
15094	SMARCA5	-0.5558	0.3355	SWI/SNF related, matrix associated, actin dependent regulator of chromatin, subfamily a, member 5
15095	SCRG1	-0.5558	0.0727	stimulator of chondrogenesis 1
15096	MAG	-0.5558	0.2001	myelin associated glycoprotein
15097	KLHDC4	-0.5558	0.1104	kelch domain containing 4
15098	ZNF341	-0.5562	0.1283	zinc finger protein 341
15099	SYCE1L	-0.5562	0.1535	synaptonemal complex central element protein 1-like
15100	NPM2	-0.5562	0.2942	nucleophosmin/nucleoplasmin 2
15101	LIN9	-0.5562	0.2626	lin-9 homolog (C. elegans)
15102	HMGB4	-0.5562	0.2034	high mobility group box 4
15103	GPR78	-0.5562	0.2564	G protein-coupled receptor 78
15104	DLGAP3	-0.5562	0.3196	discs, large (Drosophila) homolog-associated protein 3
15105	DEPDC7	-0.5562	0.0518	DEP domain containing 7
15106	C16orf11	-0.5562	0.3374	chromosome 16 open reading frame 11
15107	RGS7	-0.5567	0.0389	regulator of G-protein signaling 7
15108	NEDD8	-0.5567	0.1939	neural precursor cell expressed, developmentally down-regulated 8
15109	MAP2K1	-0.5567	0.1736	mitogen-activated protein kinase kinase 1
15110	CSNK1E	-0.5567	0.09	casein kinase 1, epsilon
15111	CAMK2B	-0.5567	0.332	calcium/calmodulin-dependent protein kinase II beta
15112	CAMK1	-0.5567	0.0736	calcium/calmodulin-dependent protein kinase I
15113	ACTR1A	-0.5567	0.1521	ARP1 actin-related protein 1 homolog A, centractin alpha (yeast)
15114	LINC00112	-0.5571	0.9996	long intergenic non-protein coding RNA 112
15115	TRIML1	-0.5575	0.1435	tripartite motif family-like 1
15116	TRAP1	-0.5575	0.2123	TNF receptor-associated protein 1
15117	SLITRK2	-0.5575	0.1088	SLIT and NTRK-like family, member 2
15118	RAD21	-0.5575	0.2977	RAD21 homolog (S. pombe)

15119	PSPN	-0.5575	0.2569	persephin
15120	NUP210	-0.5575	0.1204	nucleoporin 210kDa
15121	NDUFA7	-0.5575	0.1905	NADH dehydrogenase (ubiquinone) 1 alpha subcomplex, 7, 14.5kDa
15122	LINC00304	-0.5575	0.2972	long intergenic non-protein coding RNA 304
15123	IRGC	-0.5575	0.2779	immunity-related GTPase family, cinema
15124	C19orf54	-0.5582	0.0949	chromosome 19 open reading frame 54
15125	SSSCA1	-0.5583	0.1972	Sjogren syndrome/scleroderma autoantigen 1
15126	RAB28	-0.5583	0.2919	RAB28, member RAS oncogene family
15127	MIR17HG	-0.5583	0.1501	miR-17-92 cluster host gene (non-protein coding)
15128	FASTKD5	-0.5583	0.1507	FAST kinase domains 5
15129	EXOSC4	-0.5583	0.2137	exosome component 4
15130	DCAF7	-0.5583	0.0929	DDB1 and CUL4 associated factor 7
15131	ANP32E	-0.5583	0.2956	acidic (leucine-rich) nuclear phosphoprotein 32 family, member E
15132	LOC255167	-0.5586	0.9999	uncharacterized LOC255167
15133	TTC21A	-0.5587	0.1254	tetratricopeptide repeat domain 21A
15134	TMEM74	-0.5587	0.041	transmembrane protein 74
15135	TBATA	-0.5587	0.2596	thymus, brain and testes associated
15136	NDUFA12	-0.5587	0.2108	NADH dehydrogenase (ubiquinone) 1 alpha subcomplex, 12
15137	MAMSTR	-0.5587	0.199	MEF2 activating motif and SAP domain containing transcriptional regulator
15138	LSM10	-0.5587	0.1488	LSM10, U7 small nuclear RNA associated
15139	LHFPL5	-0.5587	0.1777	lipoma HMGIC fusion partner-like 5
15140	FAM24B	-0.5587	0.0254	family with sequence similarity 24, member B
15141	DKFZp434L192	-0.5587	0.0173	uncharacterized protein DKFZp434L192
15142	DDX53	-0.5587	0.0107	DEAD (Asp-Glu-Ala-Asp) box polypeptide 53
15143	RGS20	-0.5592	0.0366	regulator of G-protein signaling 20
15144	MATK	-0.5592	0.1563	megakaryocyte-associated tyrosine kinase
15145	MAPK8IP1	-0.5592	0.0515	mitogen-activated protein kinase 8 interacting protein 1
15146	DDX41	-0.5592	0.1904	DEAD (Asp-Glu-Ala-Asp) box polypeptide 41
15147	CORO1A	-0.5592	0.1389	coronin, actin binding protein, 1A
15148	OR51I1	-0.56	0.2791	olfactory receptor, family 51, subfamily I, member 1
15149	IMMP1L	-0.56	0.2423	IMP1 inner mitochondrial membrane peptidase-like (S. cerevisiae)
15150	FCRL3	-0.56	0.1149	Fc receptor-like 3
15151	DPPA2	-0.56	0.033	developmental pluripotency associated 2
15152	DNAAF3	-0.56	0.1273	dynein, axonemal, assembly factor 3
15153	DEF8	-0.56	0.088	differentially expressed in FDCP 8 homolog (mouse)
15154	ACTL7B	-0.56	0.2379	actin-like 7B
15155	USF2	-0.5608	0.2173	upstream transcription factor 2, c-fos interacting
15156	MRPL12	-0.5608	0.2534	mitochondrial ribosomal protein L12
15157	APBA3	-0.5608	0.1084	amyloid beta (A4) precursor protein-binding, family A, member 3
15158	UNC13C	-0.5612	0.0303	unc-13 homolog C (C. elegans)
15159	SLC30A8	-0.5612	0.17	solute carrier family 30 (zinc transporter), member 8
15160	COX19	-0.5612	0.0739	COX19 cytochrome c oxidase assembly homolog (S. cerevisiae)
15161	VRK3	-0.5617	0.0806	vaccinia related kinase 3
15162	SNRNP40	-0.5617	0.1637	small nuclear ribonucleoprotein 40kDa (U5)
15163	IMP4	-0.5617	0.2132	IMP4, U3 small nucleolar ribonucleoprotein, homolog (yeast)
15164	HIGD1B	-0.5617	0.0906	HIG1 hypoxia inducible domain family, member 1B
15165	HAGH	-0.5617	0.1266	hydroxyacylglutathione hydrolase
15166	FANCE	-0.5617	0.1027	Fanconi anemia, complementation group E
15167	SLC24A4	-0.5625	0.0751	solute carrier family 24 (sodium/potassium/calcium exchanger), member 4
15168	SH3GL2	-0.5625	0.0355	SH3-domain GRB2-like 2
15169	SAMD15	-0.5625	0.0719	sterile alpha motif domain containing 15
15170	RABEPK	-0.5625	0.1587	Rab9 effector protein with kelch motifs
15171	DENND5A	-0.5625	0.1249	DENN/MADD domain containing 5A
15172	LOC338588	-0.5629	0.4169	uncharacterized LOC338588
15173	PCDHB6	-0.5633	0.0289	protocadherin beta 6
15174	NKX2-2	-0.5633	0.0196	NK2 homeobox 2
15175	GTPBP2	-0.5633	0.0657	GTP binding protein 2
15176	GRM5	-0.5633	0.2692	glutamate receptor, metabotropic 5
15177	CNPY3	-0.5633	0.1316	canopy 3 homolog (zebrafish)
15178	ZNF30	-0.5638	0.1409	zinc finger protein 30
15179	SUSD3	-0.5638	0.0671	sushi domain containing 3
15180	PNMAL2	-0.5638	0.2694	paraneoplastic Ma antigen family-like 2
15181	FBXO27	-0.5638	0.0763	F-box protein 27
15182	RPL39L	-0.5642	0.1157	ribosomal protein L39-like
15183	PGK2	-0.5642	0.1756	phosphoglycerate kinase 2
15184	LYL1	-0.5642	0.2221	lymphoblastic leukemia derived sequence 1
15185	ASPM	-0.5642	0.2115	asp (abnormal spindle) homolog, microcephaly associated (Drosophila)
15186	STK31	-0.565	0.0293	serine/threonine kinase 31
15187	sept-01	-0.565	0.1113	septin 1
15188	PPFIA4	-0.565	0.045	protein tyrosine phosphatase, receptor type, f polypeptide (PTPRF), interacting protein (liprin), alpha 4
15189	NUP85	-0.565	0.2682	nucleoporin 85kDa
15190	NAT6	-0.565	0.139	N-acetyltransferase 6 (GCN5-related)
15191	LINC00518	-0.565	0.0354	long intergenic non-protein coding RNA 518
15192	HMGB3	-0.565	0.1074	high mobility group box 3
15193	GOT1L1	-0.565	0.0877	glutamic-oxaloacetic transaminase 1-like 1
15194	CLEC12B	-0.565	0.0496	C-type lectin domain family 12, member B
15195	CHTOP	-0.565	0.1326	chromatin target of PRMT1
15196	ANKRD33B	-0.565	0.9996	ankyrin repeat domain 33B
15197	ANKMY1	-0.565	0.0531	ankyrin repeat and MYND domain containing 1
15198	UBR7	-0.5658	0.285	ubiquitin protein ligase E3 component n-recognin 7 (putative)
15199	CNP	-0.5658	0.0583	2',3'-cyclic nucleotide 3' phosphodiesterase
15200	PPP1R9B	-0.5663	0.1336	protein phosphatase 1, regulatory subunit 9B
15201	NPFFR2	-0.5663	0.0362	neuropeptide FF receptor 2
15202	HBM	-0.5663	0.1519	hemoglobin, mu

15203	FBXO25	-0.5663	0.0424	F-box protein 25
15204	CNTNAP4	-0.5663	0.1682	contactin associated protein-like 4
15205	AMN1	-0.5663	0.1266	antagonist of mitotic exit network 1 homolog (S. cerevisiae)
15206	STX1B	-0.5667	0.1515	syntaxin 1B
15207	POLD2	-0.5667	0.2122	polymerase (DNA directed), delta 2, accessory subunit
15208	NPRL3	-0.5667	0.1386	nitrogen permease regulator-like 3 (S. cerevisiae)
15209	COPS4	-0.5667	0.3049	COP9 constitutive photomorphogenic homolog subunit 4 (Arabidopsis)
15210	C19orf40	-0.5667	0.071	chromosome 19 open reading frame 40
15211	VCX	-0.5671	0.0301	variable charge, X-linked
15212	RASAL3	-0.5675	0.1659	RAS protein activator like 3
15213	PRIMA1	-0.5675	0.0847	proline rich membrane anchor 1
15214	HBE1	-0.5675	0.1237	hemoglobin, epsilon 1
15215	H1FNT	-0.5675	0.3859	H1 histone family, member N, testis-specific
15216	CXXC4	-0.5675	0.0304	CXXC finger protein 4
15217	CELA1	-0.5675	0.2506	chymotrypsin-like elastase family, member 1
15218	C9orf91	-0.5675	0.0529	chromosome 9 open reading frame 91
15219	ASPDH	-0.5675	0.283	aspartate dehydrogenase domain containing
15220	PPP2R5D	-0.5683	0.107	protein phosphatase 2, regulatory subunit B', delta
15221	MKI67	-0.5683	0.1726	antigen identified by monoclonal antibody Ki-67
15222	LRPPRC	-0.5683	0.3098	leucine-rich pentatricopeptide repeat containing
15223	KIF11	-0.5683	0.2853	kinesin family member 11
15224	CHORDC1	-0.5683	0.2816	cysteine and histidine-rich domain (CHORD) containing 1
15225	LOC284788	-0.5686	0.2719	uncharacterized LOC284788
15226	BREA2	-0.5686	0.1147	breast cancer estrogen-induced apoptosis 2
15227	MDN1	-0.5688	0.0438	MDN1, midasin homolog (yeast)
15228	SIAH2	-0.5692	0.0635	siah E3 ubiquitin protein ligase 2
15229	SEMA4D	-0.5692	0.0729	sema domain, immunoglobulin domain (Ig), transmembrane domain (TM) and short cytoplasmic domain, (semaphorin) 4D
15230	PPM1D	-0.5692	0.2125	protein phosphatase, Mg2+/Mn2+ dependent, 1D
15231	NDUFAF4	-0.5692	0.2566	NADH dehydrogenase (ubiquinone) complex I, assembly factor 4
15232	NDUFA2	-0.5692	0.1884	NADH dehydrogenase (ubiquinone) 1 alpha subcomplex, 2, 8kDa
15233	NCAN	-0.5692	0.1669	neurocan
15234	MRPL16	-0.5692	0.2389	mitochondrial ribosomal protein L16
15235	TBRG4	-0.57	0.1977	transforming growth factor beta regulator 4
15236	POLA2	-0.57	0.1735	polymerase (DNA directed), alpha 2, accessory subunit
15237	NT5DC3	-0.57	0.0248	5'-nucleotidase domain containing 3
15238	LOC100287576	-0.57	0.9999	uncharacterized LOC100287576
15239	LOC100130872	-0.57	0.2964	uncharacterized LOC100130872
15240	KIF2B	-0.57	0.2316	kinesin family member 2B
15241	DYNC1I1	-0.57	0.0414	dynein, cytoplasmic 1, intermediate chain 1
15242	CSNK2A1	-0.57	0.187	casein kinase 2, alpha 1 polypeptide
15243	CS	-0.57	0.108	citrate synthase
15244	CMTM5	-0.57	0.1243	CKLF-like MARVEL transmembrane domain containing 5
15245	C2orf42	-0.57	0.1032	chromosome 2 open reading frame 42
15246	C17orf89	-0.57	0.1616	chromosome 17 open reading frame 89
15247	ARHGAP19	-0.57	0.1766	Rho GTPase activating protein 19
15248	RRP12	-0.5708	0.1447	ribosomal RNA processing 12 homolog (S. cerevisiae)
15249	KHDRBS1	-0.5708	0.274	KH domain containing, RNA binding, signal transduction associated 1
15250	TAB3	-0.5713	0.1638	TGF-beta activated kinase 1/MAP3K7 binding protein 3
15251	KNDC1	-0.5713	0.2179	kinase non-catalytic C-lobe domain (KIND) containing 1
15252	KNCN	-0.5713	0.3207	kinocilin
15253	DHX36	-0.5713	0.2481	DEAH (Asp-Glu-Ala-His) box polypeptide 36
15254	CCDC36	-0.5713	0.1969	coiled-coil domain containing 36
15255	SEBOX	-0.5714	0.3152	SEBOX homeobox
15256	ZNF23	-0.5717	0.1855	zinc finger protein 23
15257	SREBF2	-0.5717	0.0676	sterol regulatory element binding transcription factor 2
15258	PTTG3P	-0.5717	0.137	pituitary tumor-transforming 3, pseudogene
15259	MSH5	-0.5717	0.1225	mutS homolog 5 (E. coli)
15260	METTL5	-0.5717	0.2947	methyltransferase like 5
15261	GARS	-0.5717	0.1569	glycyl-tRNA synthetase
15262	DNAJC2	-0.5717	0.2322	DnaJ (Hsp40) homolog, subfamily C, member 2
15263	ZNF318	-0.5725	0.1348	zinc finger protein 318
15264	UIMC1	-0.5725	0.1138	ubiquitin interaction motif containing 1
15265	TTF1	-0.5725	0.134	transcription termination factor, RNA polymerase I
15266	RTTN	-0.5725	0.105	rotatin
15267	RNF40	-0.5725	0.1398	ring finger protein 40, E3 ubiquitin protein ligase
15268	PTPN5	-0.5725	0.196	protein tyrosine phosphatase, non-receptor type 5 (striatum-enriched)
15269	PLA2G6	-0.5725	0.2434	phospholipase A2, group VI (cytosolic, calcium-independent)
15270	PDCD10	-0.5725	0.2935	programmed cell death 10
15271	LMNB1	-0.5725	0.1977	lamin B1
15272	KIF23	-0.5725	0.2412	kinesin family member 23
15273	GAS7	-0.5725	0.1097	growth arrest-specific 7
15274	FAM129C	-0.5725	0.1196	family with sequence similarity 129, member C
15275	EIF2B1	-0.5725	0.2593	eukaryotic translation initiation factor 2B, subunit 1 alpha, 26kDa
15276	C15orf37	-0.5725	0.0376	chromosome 15 open reading frame 37
15277	C12orf50	-0.5725	0.0247	chromosome 12 open reading frame 50
15278	APLP1	-0.5725	0.0921	amyloid beta (A4) precursor-like protein 1
15279	ADORA2A	-0.5725	0.0533	adenosine A2a receptor
15280	QDPR	-0.5733	0.1452	quinoid dihydropteridine reductase
15281	PRKRA	-0.5733	0.2749	protein kinase, interferon-inducible double stranded RNA dependent activator
15282	KLHL15	-0.5733	0.1278	kelch-like 15 (Drosophila)
15283	FIBP	-0.5733	0.2013	fibroblast growth factor (acidic) intracellular binding protein
15284	CCDC59	-0.5733	0.2462	coiled-coil domain containing 59
15285	C19orf57	-0.5733	0.2478	chromosome 19 open reading frame 57
15286	TPPP2	-0.5738	0.1757	tubulin polymerization-promoting protein family member 2

15287	SMC1B	-0.5738	0.0234	structural maintenance of chromosomes 1B
15288	LOC731789	-0.5738	0.0831	uncharacterized LOC731789
15289	IGF2BP1	-0.5738	0.106	insulin-like growth factor 2 mRNA binding protein 1
15290	FAM9C	-0.5738	0.082	family with sequence similarity 9, member C
15291	FAM122B	-0.5738	0.2123	family with sequence similarity 122B
15292	DUS3L	-0.5738	0.175	dihydrouridine synthase 3-like (S. cerevisiae)
15293	CRB2	-0.5738	0.1313	crumbs homolog 2 (Drosophila)
15294	C9orf84	-0.5738	0.0302	chromosome 9 open reading frame 84
15295	ATG9B	-0.5738	0.1266	autophagy related 9B
15296	TF	-0.5742	0.0322	transferrin
15297	NXF3	-0.5742	0.0166	nuclear RNA export factor 3
15298	SCNM1	-0.5743	0.0854	sodium channel modifier 1
15299	LOC285593	-0.5743	0.9996	uncharacterized LOC285593
15300	TAOK3	-0.575	0.1581	TAO kinase 3
15301	STK11IP	-0.575	0.111	serine/threonine kinase 11 interacting protein
15302	POLR2B	-0.575	0.3084	polymerase (RNA) II (DNA directed) polypeptide B, 140kDa
15303	PHLDB3	-0.575	0.1614	pleckstrin homology-like domain, family B, member 3
15304	MRPS18A	-0.575	0.1654	mitochondrial ribosomal protein S18A
15305	LINC00467	-0.575	0.0588	long intergenic non-protein coding RNA 467
15306	KCTD19	-0.575	0.1927	potassium channel tetramerisation domain containing 19
15307	HINT1	-0.575	0.2162	histidine triad nucleotide binding protein 1
15308	GPS2	-0.575	0.1084	G protein pathway suppressor 2
15309	GINS4	-0.575	0.126	GINS complex subunit 4 (Sld5 homolog)
15310	FKBPL	-0.575	0.0949	FK506 binding protein like
15311	CUL1	-0.575	0.2335	cullin 1
15312	C20orf20	-0.575	0.136	chromosome 20 open reading frame 20
15313	C12orf56	-0.575	0.0212	chromosome 12 open reading frame 56
15314	TEX13B	-0.5758	0.3265	testis expressed 13B
15315	SLMO1	-0.5758	0.0515	slowmo homolog 1 (Drosophila)
15316	POU4F3	-0.5758	0.0699	POU class 4 homeobox 3
15317	CBX6	-0.5758	0.0537	chromobox homolog 6
15318	SVIP	-0.5763	0.0992	small VCP/p97-interacting protein
15319	SIGLECL1	-0.5763	0.0862	SIGLEC family like 1
15320	LINC00167	-0.5763	0.2347	long intergenic non-protein coding RNA 167
15321	IPMK	-0.5763	0.0216	inositol polyphosphate multikinase
15322	C2orf57	-0.5763	0.2388	chromosome 2 open reading frame 57
15323	C1orf162	-0.5763	0.1109	chromosome 1 open reading frame 162
15324	AKNA	-0.5763	0.1104	AT-hook transcription factor
15325	ZNF696	-0.5767	0.1434	zinc finger protein 696
15326	PRPS1L1	-0.5767	0.0652	phosphoribosyl pyrophosphate synthetase 1-like 1
15327	INTS8	-0.5767	0.2137	integrator complex subunit 8
15328	LRRC37A5P	-0.5771	0.1654	leucine rich repeat containing 37, member A5, pseudogene
15329	ZNF579	-0.5775	0.2668	zinc finger protein 579
15330	ZFP42	-0.5775	0.0544	zinc finger protein 42 homolog (mouse)
15331	SIGLEC17P	-0.5775	0.057	sialic acid binding Ig-like lectin 17, pseudogene
15332	RSL1D1	-0.5775	0.1734	ribosomal L1 domain containing 1
15333	RNF187	-0.5775	0.1214	ring finger protein 187
15334	RAB3IP	-0.5775	0.0619	RAB3A interacting protein (rabin3)
15335	PTPMT1	-0.5775	0.1574	protein tyrosine phosphatase, mitochondrial 1
15336	NLRC3	-0.5775	0.0952	NLR family, CARD domain containing 3
15337	MYO16	-0.5775	0.1742	myosin XVI
15338	MAP3K9	-0.5775	0.1312	mitogen-activated protein kinase kinase kinase 9
15339	FNDC7	-0.5775	0.2055	fibronectin type III domain containing 7
15340	CPLX1	-0.5775	0.0621	complexin 1
15341	C3orf75	-0.5775	0.1599	chromosome 3 open reading frame 75
15342	GDAP1L1	-0.5783	0.1399	ganglioside induced differentiation associated protein 1-like 1
15343	ZNF70	-0.5788	0.017	zinc finger protein 70
15344	TLR9	-0.5788	0.1593	toll-like receptor 9
15345	SRG7	-0.5788	0.0239	spermatogenesis-related protein 7
15346	SCAND3	-0.5788	0.0347	SCAN domain containing 3
15347	RNF151	-0.5788	0.2587	ring finger protein 151
15348	C16orf86	-0.5788	0.258	chromosome 16 open reading frame 86
15349	APOA5	-0.5788	0.3313	apolipoprotein A-V
15350	TADA3	-0.5792	0.1155	transcriptional adaptor 3
15351	SCRT1	-0.5792	0.2835	scratch homolog 1, zinc finger protein (Drosophila)
15352	PAK2	-0.5792	0.2068	p21 protein (Cdc42/Rac)-activated kinase 2
15353	EYA3	-0.5792	0.0274	eyes absent homolog 3 (Drosophila)
15354	TMCO2	-0.58	0.1279	transmembrane and coiled-coil domains 2
15355	SKIV2L	-0.58	0.1352	superkiller viralicidic activity 2-like (S. cerevisiae)
15356	SCLY	-0.58	0.0667	selenocysteine lyase
15357	RPIA	-0.58	0.1542	ribose 5-phosphate isomerase A
15358	PPP1CC	-0.58	0.3039	protein phosphatase 1, catalytic subunit, gamma isozyme
15359	ODC1	-0.58	0.1718	ornithine decarboxylase 1
15360	NINJ2	-0.58	0.0508	ninjurin 2
15361	MRPL52	-0.58	0.1685	mitochondrial ribosomal protein L52
15362	LINC00320	-0.58	0.1216	long intergenic non-protein coding RNA 320
15363	IFI27L1	-0.58	0.0668	interferon, alpha-inducible protein 27-like 1
15364	HOXB4	-0.58	0.0298	homeobox B4
15365	GAL3ST2	-0.58	0.1997	galactose-3-O-sulfotransferase 2
15366	ARX	-0.58	0.0516	aristaless related homeobox
15367	PDSS1	-0.5808	0.2234	prenyl (decaprenyl) diphosphate synthase, subunit 1
15368	KALRN	-0.5808	0.1056	kalirin, RhoGEF kinase
15369	ATAD3A	-0.5808	0.2159	ATPase family, AAA domain containing 3A
15370	ANKS1B	-0.5808	0.05	ankyrin repeat and sterile alpha motif domain containing 1B

15371	ZNF710	-0.5811	0.0553	zinc finger protein 710
15372	TDRD5	-0.5813	0.0368	tudor domain containing 5
15373	SLC32A1	-0.5813	0.1078	solute carrier family 32 (GABA vesicular transporter), member 1
15374	RPL13AP17	-0.5813	0.1615	ribosomal protein L13a pseudogene 17
15375	LOC285548	-0.5813	0.0289	uncharacterized LOC285548
15376	ESPN	-0.5813	0.2054	espin
15377	CES5A	-0.5813	0.144	carboxylesterase 5A
15378	C22orf13	-0.5813	0.0765	chromosome 22 open reading frame 13
15379	AGPAT4-IT1	-0.5813	0.0508	AGPAT4 intronic transcript 1 (non-protein coding)
15380	LOC151760	-0.5814	0.2504	uncharacterized LOC151760
15381	HSPA4	-0.5817	0.2706	heat shock 70kDa protein 4
15382	CDK5	-0.5817	0.1342	cyclin-dependent kinase 5
15383	ZNF233	-0.5825	0.0257	zinc finger protein 233
15384	TPRN	-0.5825	0.1467	taperin
15385	TEX30	-0.5825	0.2001	testis expressed 30
15386	STRN4	-0.5825	0.142	striatin, calmodulin binding protein 4
15387	NPAS1	-0.5825	0.2671	neuronal PAS domain protein 1
15388	MS4A7	-0.5825	0.1045	membrane-spanning 4-domains, subfamily A, member 7
15389	ING1	-0.5825	0.0459	inhibitor of growth family, member 1
15390	GSC	-0.5825	0.0906	goosecoid homeobox
15391	DSCR8	-0.5825	0.0103	Down syndrome critical region gene 8
15392	CADM4	-0.5825	0.1065	cell adhesion molecule 4
15393	ZNF675	-0.5833	0.1026	zinc finger protein 675
15394	ZKSCAN5	-0.5833	0.0544	zinc finger with KRAB and SCAN domains 5
15395	RBM10	-0.5833	0.1722	RNA binding motif protein 10
15396	DNAJA1	-0.5833	0.2245	DnaJ (Hsp40) homolog, subfamily A, member 1
15397	DAXX	-0.5833	0.1036	death-domain associated protein
15398	TEKT3	-0.5838	0.0132	tektin 3
15399	SOX8	-0.5838	0.072	SRY (sex determining region Y)-box 8
15400	RTN4IP1	-0.5838	0.1973	reticulon 4 interacting protein 1
15401	UQCR10	-0.5842	0.1858	ubiquinol-cytochrome c reductase, complex III subunit X
15402	FAF1	-0.5842	0.1341	Fas (TNFRSF6) associated factor 1
15403	ZAR1	-0.585	0.2034	zygote arrest 1
15404	SPATA19	-0.585	0.1552	spermatogenesis associated 19
15405	PPP2R2A	-0.585	0.1299	protein phosphatase 2, regulatory subunit B, alpha
15406	MAZ	-0.585	0.1625	MYC-associated zinc finger protein (purine-binding transcription factor)
15407	ERI1	-0.585	0.232	exoribonuclease 1
15408	EIF1	-0.585	0.0901	eukaryotic translation initiation factor 1
15409	E2F3	-0.585	0.0779	E2F transcription factor 3
15410	DNAH10	-0.585	0.3062	dynein, axonemal, heavy chain 10
15411	CSNK1G2-AS1	-0.585	0.3325	CSNK1G2 antisense RNA 1 (non-protein coding)
15412	C1orf216	-0.585	0.0593	chromosome 1 open reading frame 216
15413	C10orf122	-0.585	0.0579	chromosome 10 open reading frame 122
15414	LOC148824	-0.5857	0.9999	uncharacterized LOC148824
15415	CMPK2	-0.5857	0.0697	cytidine monophosphate (UMP-CMP) kinase 2, mitochondrial
15416	PITPNB	-0.5858	0.1718	phosphatidylinositol transfer protein, beta
15417	MRPL28	-0.5858	0.1549	mitochondrial ribosomal protein L28
15418	B3GAT1	-0.5858	0.0458	beta-1,3-glucuronyltransferase 1 (glucuronosyltransferase P)
15419	ARNT2	-0.5858	0.0225	aryl-hydrocarbon receptor nuclear translocator 2
15420	ABCC5	-0.5858	0.0535	ATP-binding cassette, sub-family C (CFTR/MRP), member 5
15421	PCDH19	-0.5863	0.0403	protocadherin 19
15422	LOXL3	-0.5863	0.0546	lysyl oxidase-like 3
15423	KCNQ1OT1	-0.5863	0.1414	KCNQ1 opposite strand/antisense transcript 1 (non-protein coding)
15424	HEXIM2	-0.5863	0.0809	hexamethylene bis-acetamide inducible 2
15425	GRAMD1A	-0.5863	0.1007	GRAM domain containing 1A
15426	C1orf173	-0.5863	0.0444	chromosome 1 open reading frame 173
15427	C16orf82	-0.5863	0.2941	chromosome 16 open reading frame 82
15428	BRS3	-0.5863	0.0371	bombesin-like receptor 3
15429	RNF126P1	-0.5864	0.2327	ring finger protein 126 pseudogene 1
15430	ZNF222	-0.5867	0.071	zinc finger protein 222
15431	SLC18A2	-0.5867	0.0219	solute carrier family 18 (vesicular monoamine), member 2
15432	PCNA	-0.5867	0.2922	proliferating cell nuclear antigen
15433	CHST11	-0.5867	0.05	carbohydrate (chondroitin 4) sulfotransferase 11
15434	CASP3	-0.5867	0.1972	caspase 3, apoptosis-related cysteine peptidase
15435	LOC100286922	-0.5871	0.2275	DnaJ (Hsp40) homolog, subfamily B, member 3 pseudogene
15436	ZNF529	-0.5875	0.1573	zinc finger protein 529
15437	U2AF2	-0.5875	0.1236	U2 small nuclear RNA auxiliary factor 2
15438	TMEM169	-0.5875	0.0427	transmembrane protein 169
15439	SLC17A7	-0.5875	0.3272	solute carrier family 17 (sodium-dependent inorganic phosphate cotransporter), member 7
15440	SIRPB2	-0.5875	0.1035	signal-regulatory protein beta 2
15441	MORN3	-0.5875	0.035	MORN repeat containing 3
15442	CALY	-0.5875	0.1587	calcyon neuron-specific vesicular protein
15443	C3orf80	-0.5875	0.0203	chromosome 3 open reading frame 80
15444	C19orf43	-0.5875	0.1344	chromosome 19 open reading frame 43
15445	C14orf80	-0.5875	0.2094	chromosome 14 open reading frame 80
15446	RWDD2A	-0.5883	0.0719	RWD domain containing 2A
15447	KIF4A	-0.5883	0.1884	kinesin family member 4A
15448	JPH3	-0.5883	0.1369	junctophilin 3
15449	GDF11	-0.5883	0.0396	growth differentiation factor 11
15450	DHX8	-0.5883	0.0931	DEAH (Asp-Glu-Ala-His) box polypeptide 8
15451	ADSL	-0.5883	0.2586	adenylosuccinate lyase
15452	PABPC1L2B	-0.5886	0.0351	poly(A) binding protein, cytoplasmic 1-like 2B
15453	CCDC168	-0.5886	0.1621	coiled-coil domain containing 168
15454	ASB15	-0.5886	0.1848	ankyrin repeat and SOCS box containing 15

15455	RCC2	-0.5888	0.1468	regulator of chromosome condensation 2
15456	PSMA8	-0.5888	0.0111	proteasome (prosome, macropain) subunit, alpha type, 8
15457	HILS1	-0.5888	0.1809	histone linker H1 domain, spermatid-specific 1, pseudogene
15458	FAM71C	-0.5888	0.1057	family with sequence similarity 71, member C
15459	FAM222A-AS1	-0.5888	0.0556	FAM222A antisense RNA 1 (non-protein coding)
15460	MARCKSL1	-0.5892	0.0608	MARCKS-like 1
15461	MAP2K7	-0.5892	0.1735	mitogen-activated protein kinase kinase 7
15462	VAV1	-0.59	0.0853	vav 1 guanine nucleotide exchange factor
15463	TLK2	-0.59	0.0573	tousled-like kinase 2
15464	PRRT1	-0.59	0.2314	proline-rich transmembrane protein 1
15465	PGLYRP3	-0.59	0.1928	peptidoglycan recognition protein 3
15466	LYG2	-0.59	0.0098	lysozyme G-like 2
15467	LOC100128292	-0.59	0.3434	uncharacterized LOC100128292
15468	LBX2-AS1	-0.59	0.1881	LBX2 antisense RNA 1 (non-protein coding)
15469	H1FX	-0.59	0.1265	H1 histone family, member X
15470	GLIPR1L1	-0.59	0.0553	GLI pathogenesis-related 1 like 1
15471	FGFR1OP	-0.59	0.1871	FGFR1 oncogene partner
15472	CCNB2	-0.59	0.2047	cyclin B2
15473	CARS	-0.59	0.0622	cysteinyl-tRNA synthetase
15474	MAP4K1	-0.5908	0.1471	mitogen-activated protein kinase kinase kinase kinase 1
15475	ICAM5	-0.5908	0.1467	intercellular adhesion molecule 5, telencephalin
15476	FGF3	-0.5908	0.2676	fibroblast growth factor 3
15477	SLC5A11	-0.5913	0.1796	solute carrier family 5 (sodium/glucose cotransporter), member 11
15478	SGSM1	-0.5913	0.2296	small G protein signaling modulator 1
15479	LINC00238	-0.5913	0.0087	long intergenic non-protein coding RNA 238
15480	LIN54	-0.5913	0.0734	lin-54 homolog (C. elegans)
15481	KLF14	-0.5913	0.3331	Kruppel-like factor 14
15482	FNDC9	-0.5913	0.1311	fibronectin type III domain containing 9
15483	C19orf47	-0.5913	0.1214	chromosome 19 open reading frame 47
15484	ABCC12	-0.5913	0.1503	ATP-binding cassette, sub-family C (CFTR/MRP), member 12
15485	LOC100288123	-0.5914	0.9999	uncharacterized LOC100288123
15486	HSPA8	-0.5917	0.2256	heat shock 70kDa protein 8
15487	GRAP	-0.5917	0.0825	GRB2-related adaptor protein
15488	FAM217B	-0.5917	0.9996	family with sequence similarity 217, member B
15489	TIGD4	-0.5925	0.0412	tigger transposable element derived 4
15490	RACGAP1P	-0.5925	0.0234	Rac GTPase activating protein 1 pseudogene
15491	PUSL1	-0.5925	0.1599	pseudouridylate synthase-like 1
15492	KLHL6	-0.5925	0.0792	kelch-like 6 (Drosophila)
15493	FSIP2	-0.5925	0.0226	fibrous sheath interacting protein 2
15494	FNBP1	-0.5925	0.1025	formin binding protein 1
15495	EFNA2	-0.5925	0.2125	ephrin-A2
15496	ATCAY	-0.5925	0.268	ataxia, cerebellar, Cayman type
15497	ALKBH7	-0.5925	0.1433	alkB, alkylation repair homolog 7 (E. coli)
15498	KRTAP10-11	-0.5929	0.343	keratin associated protein 10-11
15499	TERT	-0.5933	0.27	telomerase reverse transcriptase
15500	TELO2	-0.5933	0.2059	TEL2, telomere maintenance 2, homolog (S. cerevisiae)
15501	SSRP1	-0.5933	0.2305	structure specific recognition protein 1
15502	PREP	-0.5933	0.092	prolyl endopeptidase
15503	HIST1H1E	-0.5933	0.0333	histone cluster 1, H1e
15504	CCT5	-0.5933	0.2391	chaperonin containing TCP1, subunit 5 (epsilon)
15505	C16orf88	-0.5933	0.0879	chromosome 16 open reading frame 88
15506	RBFOX3	-0.5938	0.1962	RNA binding protein, fox-1 homolog (C. elegans) 3
15507	NLRP6	-0.5938	0.0582	NLR family, pyrin domain containing 6
15508	LOC100130557	-0.5938	0.1066	uncharacterized LOC100130557
15509	IL4I1	-0.5938	0.14	interleukin 4 induced 1
15510	DNAH14	-0.5938	0.0422	dynein, axonemal, heavy chain 14
15511	CABP7	-0.5938	0.0866	calcium binding protein 7
15512	AK7	-0.5938	0.0245	adenylate kinase 7
15513	ZNF259	-0.5942	0.1403	zinc finger protein 259
15514	NSF	-0.5942	0.1811	N-ethylmaleimide-sensitive factor
15515	NELF	-0.5942	0.0741	nasal embryonic LHRH factor
15516	CKAP2	-0.5942	0.2881	cytoskeleton associated protein 2
15517	SNX30	-0.5943	0.1528	sorting nexin family member 30
15518	TTI1	-0.595	0.1932	TELO2 interacting protein 1
15519	SYN1	-0.595	0.3294	synapsin I
15520	PPP3R1	-0.595	0.0822	protein phosphatase 3, regulatory subunit B, alpha
15521	LMTK3	-0.595	0.2082	lemur tyrosine kinase 3
15522	C20orf141	-0.595	0.3235	chromosome 20 open reading frame 141
15523	C1orf187	-0.595	0.0428	chromosome 1 open reading frame 187
15524	RPS13	-0.5957	0.0931	ribosomal protein S13
15525	ARPP19	-0.5958	0.2542	cAMP-regulated phosphoprotein, 19kDa
15526	ZNF713	-0.5962	0.0245	zinc finger protein 713
15527	VWA3A	-0.5962	0.3303	von Willebrand factor A domain containing 3A
15528	LRRC4B	-0.5962	0.1169	leucine rich repeat containing 4B
15529	LOC150622	-0.5962	0.0897	uncharacterized LOC150622
15530	GRIN3A	-0.5962	0.125	glutamate receptor, ionotropic, N-methyl-D-aspartate 3A
15531	DPP9	-0.5962	0.1179	dipeptidyl-peptidase 9
15532	DCST1	-0.5962	0.2552	DC-STAMP domain containing 1
15533	SLC8A2	-0.5967	0.2675	solute carrier family 8 (sodium/calcium exchanger), member 2
15534	OXLD1	-0.5967	0.1511	oxidoreductase-like domain containing 1
15535	GFI1B	-0.5967	0.1382	growth factor independent 1B transcription repressor
15536	CATSPERG	-0.5967	0.2662	catsper channel auxiliary subunit gamma
15537	CA5BP1	-0.5967	0.1102	carbonic anhydrase VB pseudogene 1
15538	ZC3H3	-0.5975	0.0987	zinc finger CCCH-type containing 3

15539	TRPM2	-0.5975	0.1901	transient receptor potential cation channel, subfamily M, member 2
15540	TMIGD2	-0.5975	0.1479	transmembrane and immunoglobulin domain containing 2
15541	sept-06	-0.5975	0.0634	septin 6
15542	PRR7	-0.5975	0.1534	proline rich 7 (synaptic)
15543	NOTUM	-0.5975	0.2019	notum pectinacetylesterase homolog (Drosophila)
15544	LAMB2P1	-0.5975	0.1094	laminin, beta 2 pseudogene 1
15545	KNTC1	-0.5975	0.2085	kinetochore associated 1
15546	JMJD6	-0.5975	0.0658	jumonji domain containing 6
15547	LOC100268168	-0.5986	0.1283	uncharacterized LOC100268168
15548	TAGAP	-0.5987	0.1133	T-cell activation RhoGTPase activating protein
15549	SH2D1B	-0.5987	0.0314	SH2 domain containing 1B
15550	RELL2	-0.5987	0.1051	RELT-like 2
15551	PNCK	-0.5987	0.1726	pregnancy up-regulated non-ubiquitously expressed CaM kinase
15552	KATNAL1	-0.5987	0.0903	katanin p60 subunit A-like 1
15553	FDX1L	-0.5987	0.1506	ferredoxin 1-like
15554	FAM211A	-0.5987	0.017	family with sequence similarity 211, member A
15555	DNAH8	-0.5987	0.0572	dynein, axonemal, heavy chain 8
15556	CCDC14	-0.5987	0.1987	coiled-coil domain containing 14
15557	ANKRD34A	-0.5987	0.0913	ankyrin repeat domain 34A
15558	WDR7	-0.5992	0.1415	WD repeat domain 7
15559	TTI2	-0.5992	0.0739	TELO2 interacting protein 2
15560	SYN2	-0.5992	0.2116	synapsin II
15561	PSMD12	-0.5992	0.2736	proteasome (prosome, macropain) 26S subunit, non-ATPase, 12
15562	HIST1H4B	-0.5992	0.039	histone cluster 1, H4b
15563	C7orf43	-0.5992	0.1257	chromosome 7 open reading frame 43
15564	AKAP8	-0.5992	0.0857	A kinase (PRKA) anchor protein 8
15565	UNC13D	-0.6	0.2086	unc-13 homolog D (C. elegans)
15566	TMOD2	-0.6	0.0428	tropomodulin 2 (neuronal)
15567	TMEM121	-0.6	0.1113	transmembrane protein 121
15568	SURF6	-0.6	0.1129	surfeit 6
15569	RBM19	-0.6	0.0598	RNA binding motif protein 19
15570	PRDM7	-0.6	0.0165	PR domain containing 7
15571	POLR3K	-0.6	0.2046	polymerase (RNA) III (DNA directed) polypeptide K, 12.3 kDa
15572	GOLGA7B	-0.6	0.242	golgin A7 family, member B
15573	CRKL	-0.6	0.0905	v-crk sarcoma virus CT10 oncogene homolog (avian)-like
15574	CNTROB	-0.6	0.139	centrobin, centrosomal BRCA2 interacting protein
15575	PPM1E	-0.6008	0.0542	protein phosphatase, Mg2+/Mn2+ dependent, 1E
15576	MRPL2	-0.6008	0.1197	mitochondrial ribosomal protein L2
15577	INPP5F	-0.6008	0.0609	inositol polyphosphate-5-phosphatase F
15578	C17orf80	-0.6008	0.0705	chromosome 17 open reading frame 80
15579	TOX2	-0.6012	0.0281	TOX high mobility group box family member 2
15580	PRRT3	-0.6012	0.0363	proline-rich transmembrane protein 3
15581	PAQR9	-0.6012	0.022	progestin and adipoQ receptor family member IX
15582	MGC12916	-0.6012	0.0537	uncharacterized protein MGC12916
15583	MAGEE2	-0.6012	0.0244	melanoma antigen family E, 2
15584	GTDC2	-0.6012	0.0565	glycosyltransferase-like domain containing 2
15585	CLECL1	-0.6012	0.0444	C-type lectin-like 1
15586	C3orf24	-0.6012	0.2519	chromosome 3 open reading frame 24
15587	C11orf65	-0.6012	0.016	chromosome 11 open reading frame 65
15588	ALKBH6	-0.6012	0.0592	alkB, alkylation repair homolog 6 (E. coli)
15589	YWHAE	-0.6025	0.1025	tyrosine 3-monooxygenase/tryptophan 5-monooxygenase activation protein, epsilon polypeptide
15590	SP8	-0.6025	0.0651	Sp8 transcription factor
15591	SLC9B1	-0.6025	0.0056	solute carrier family 9, subfamily B (NHA1, cation proton antiporter 1), member 1
15592	PPP1R7	-0.6025	0.1908	protein phosphatase 1, regulatory subunit 7
15593	OSTalpha	-0.6025	0.0397	organic solute transporter alpha
15594	MPV17L2	-0.6025	0.1182	MPV17 mitochondrial membrane protein-like 2
15595	LZTS1	-0.6025	0.251	leucine zipper, putative tumor suppressor 1
15596	HS3ST5	-0.6025	0.051	heparan sulfate (glucosamine) 3-O-sulfotransferase 5
15597	AURKC	-0.6025	0.1344	aurora kinase C
15598	ANAPC2	-0.6025	0.139	anaphase promoting complex subunit 2
15599	YWHAQ	-0.6029	0.2299	tyrosine 3-monooxygenase/tryptophan 5-monooxygenase activation protein, theta polypeptide
15600	RPL26L1	-0.6029	0.1595	ribosomal protein L26-like 1
15601	MRVI1-AS1	-0.6029	0.3289	MRVI1 antisense RNA 1 (non-protein coding)
15602	LRRC52	-0.6029	0.2689	leucine rich repeat containing 52
15603	BMI1	-0.6029	0.2049	BMI1 polycomb ring finger oncogene
15604	XAB2	-0.6033	0.0838	XPA binding protein 2
15605	TAF7L	-0.6033	0.0302	TAF7-like RNA polymerase II, TATA box binding protein (TBP)-associated factor, 50kDa
15606	PTOV1	-0.6033	0.1602	prostate tumor overexpressed 1
15607	PSMD10	-0.6033	0.2834	proteasome (prosome, macropain) 26S subunit, non-ATPase, 10
15608	NOL11	-0.6033	0.2957	nucleolar protein 11
15609	NDUFB4	-0.6033	0.137	NADH dehydrogenase (ubiquinone) 1 beta subcomplex, 4, 15kDa
15610	TEX101	-0.6037	0.1079	testis expressed 101
15611	WDR67	-0.6042	0.2132	WD repeat domain 67
15612	STK11	-0.6042	0.177	serine/threonine kinase 11
15613	PCYT2	-0.6042	0.1287	phosphate cytidylyltransferase 2, ethanolamine
15614	PARPBP	-0.6042	0.2257	PARP1 binding protein
15615	KRI1	-0.6042	0.1299	KRI1 homolog (S. cerevisiae)
15616	GPI	-0.6042	0.1375	glucose-6-phosphate isomerase
15617	TTLL9	-0.6043	0.2787	tubulin tyrosine ligase-like family, member 9
15618	RBMY3AP	-0.6043	0.9996	RNA binding motif protein, Y-linked, family 3, member A pseudogene
15619	LOC100287628	-0.6043	0.0292	uncharacterized LOC100287628
15620	TIMM23	-0.6045	0.2496	translocase of inner mitochondrial membrane 23 homolog (yeast)
15621	ZRANB3	-0.605	0.1101	zinc finger, RAN-binding domain containing 3
15622	ZDHHC23	-0.605	0.0438	zinc finger, DHHC-type containing 23

15623	SPATS1	-0.605	0.064	spermatogenesis associated, serine-rich 1
15624	SH2D4B	-0.605	0.0858	SH2 domain containing 4B
15625	RTP3	-0.605	0.1996	receptor (chemosensory) transporter protein 3
15626	PP2D1	-0.605	0.9999	protein phosphatase 2C-like domain containing 1
15627	NLRP4	-0.605	0.0689	NLR family, pyrin domain containing 4
15628	MRPS5	-0.605	0.1078	mitochondrial ribosomal protein S5
15629	LINC00526	-0.605	0.0434	long intergenic non-protein coding RNA 526
15630	CST9	-0.605	0.0736	cystatin 9 (testatin)
15631	USP32	-0.6058	0.0453	ubiquitin specific peptidase 32
15632	UBIAD1	-0.6058	0.0729	UbiA prenyltransferase domain containing 1
15633	SYNGR4	-0.6058	0.2771	synaptogyrin 4
15634	HJURP	-0.6058	0.1522	Holliday junction recognition protein
15635	ZFAND4	-0.6062	0.0661	zinc finger, AN1-type domain 4
15636	ADCK1	-0.6062	0.0516	aarF domain containing kinase 1
15637	SYMPK	-0.6067	0.1199	symplekin
15638	SF3B2	-0.6067	0.1507	splicing factor 3b, subunit 2, 145kDa
15639	HNRNPD	-0.6067	0.2831	heterogeneous nuclear ribonucleoprotein D (AU-rich element RNA binding protein 1, 37kDa)
15640	ASPSCR1	-0.6067	0.1521	alveolar soft part sarcoma chromosome region, candidate 1
15641	C3orf65	-0.6071	0.0533	chromosome 3 open reading frame 65
15642	ZNF446	-0.6075	0.2555	zinc finger protein 446
15643	OPALIN	-0.6075	0.1573	oligodendrocytic myelin paranodal and inner loop protein
15644	NUDT9P1	-0.6075	0.0184	nudix (nucleoside diphosphate linked moiety X)-type motif 9 pseudogene 1
15645	JOSD1	-0.6075	0.0805	Josephin domain containing 1
15646	FAM123B	-0.6075	0.0315	family with sequence similarity 123B
15647	EFCAB11	-0.6075	0.1083	EF-hand calcium binding domain 11
15648	DACT3	-0.6075	0.1677	dapper, antagonist of beta-catenin, homolog 3 (Xenopus laevis)
15649	CCNA1	-0.6075	0.014	cyclin A1
15650	C4orf17	-0.6075	0.2177	chromosome 4 open reading frame 17
15651	VGF	-0.6083	0.1198	VGF nerve growth factor inducible
15652	LIN37	-0.6083	0.1045	lin-37 homolog (C. elegans)
15653	IKZF1	-0.6083	0.0926	IKAROS family zinc finger 1 (Ikaros)
15654	GART	-0.6083	0.2357	phosphoribosylglycinamide formyltransferase, phosphoribosylglycinamide synthetase, phosphoribosylaminoimidazole synthetase
15655	SEC1P	-0.6086	0.3397	secretory blood group 1, pseudogene
15656	ZNF138	-0.6087	0.1658	zinc finger protein 138
15657	TGM7	-0.6087	0.2382	transglutaminase 7
15658	EFTUD2	-0.6087	0.1579	elongation factor Tu GTP binding domain containing 2
15659	CBLN4	-0.6087	0.0538	cerebellin 4 precursor
15660	TCERG1	-0.6092	0.2976	transcription elongation regulator 1
15661	RPF1	-0.6092	0.2892	ribosome production factor 1 homolog (S. cerevisiae)
15662	ADO	-0.6092	0.2052	2-aminoethanethiol (cysteamine) dioxygenase
15663	WDR93	-0.61	0.2621	WD repeat domain 93
15664	SUN5	-0.61	0.2928	Sad1 and UNC84 domain containing 5
15665	POLR3D	-0.61	0.0462	polymerase (RNA) III (DNA directed) polypeptide D, 44kDa
15666	OR1I1	-0.61	0.3316	olfactory receptor, family 1, subfamily I, member 1
15667	HENMT1	-0.61	0.031	HEN1 methyltransferase homolog 1 (Arabidopsis)
15668	ACYP1	-0.61	0.1755	acylphosphatase 1, erythrocyte (common) type
15669	TFAM	-0.6108	0.2029	transcription factor A, mitochondrial
15670	MAP6D1	-0.6108	0.0629	MAP6 domain containing 1
15671	ISYNA1	-0.6108	0.0697	inositol-3-phosphate synthase 1
15672	FAM222B	-0.6108	0.039	family with sequence similarity 222, member B
15673	ERC2	-0.6108	0.024	ELKS/RAB6-interacting/CAST family member 2
15674	C11orf48	-0.6108	0.1878	chromosome 11 open reading frame 48
15675	ANAPC15	-0.6108	0.1144	anaphase promoting complex subunit 15
15676	NOM1	-0.6112	0.1567	nucleolar protein with MIF4G domain 1
15677	HEMGN	-0.6112	0.0266	hemogen
15678	C5orf64	-0.6112	0.0704	chromosome 5 open reading frame 64
15679	ZC2HC1B	-0.6114	0.0584	zinc finger, C2HC-type containing 1B
15680	SLC12A5	-0.6117	0.1334	solute carrier family 12 (potassium/chloride transporter), member 5
15681	S100PBP	-0.6117	0.1838	S100P binding protein
15682	NCDN	-0.6117	0.1161	neurochondrin
15683	YTHDF2	-0.6118	0.2046	YTH domain family, member 2
15684	SMG5	-0.6125	0.1049	smg-5 homolog, nonsense mediated mRNA decay factor (C. elegans)
15685	OOEP	-0.6125	0.0065	oocyte expressed protein
15686	OIP5	-0.6125	0.2229	Opa interacting protein 5
15687	NCKIPSD	-0.6125	0.0557	NCK interacting protein with SH3 domain
15688	LY6H	-0.6125	0.0562	lymphocyte antigen 6 complex, locus H
15689	HMP19	-0.6125	0.0905	HMP19 protein
15690	GPN2	-0.6125	0.0998	GPN-loop GTPase 2
15691	EXTL3	-0.6125	0.0563	exostoses (multiple)-like 3
15692	CPNE9	-0.6125	0.1382	copine family member IX
15693	CALM2	-0.6125	0.235	calmodulin 2 (phosphorylase kinase, delta)
15694	C12orf65	-0.6125	0.0662	chromosome 12 open reading frame 65
15695	OR5K1	-0.6129	0.0288	olfactory receptor, family 5, subfamily K, member 1
15696	OR5J2	-0.6129	0.0618	olfactory receptor, family 5, subfamily J, member 2
15697	SAMSN1	-0.6133	0.1085	SAM domain, SH3 domain and nuclear localization signals 1
15698	POLR3G	-0.6133	0.0946	polymerase (RNA) III (DNA directed) polypeptide G (32kD)
15699	AMBRA1	-0.6133	0.0361	autophagy/beclin-1 regulator 1
15700	ZFHX2	-0.6137	0.1302	zinc finger homeobox 2
15701	XAGE3	-0.6137	0.0298	X antigen family, member 3
15702	PGBD4	-0.6137	0.025	piggyBac transposable element derived 4
15703	MMD2	-0.6137	0.2134	monocyte to macrophage differentiation-associated 2
15704	LINC00032	-0.6137	0.0724	long intergenic non-protein coding RNA 32
15705	CMTM1	-0.6137	0.0098	CKLF-like MARVEL transmembrane domain containing 1
15706	C6orf99	-0.6137	0.0259	chromosome 6 open reading frame 99

15707	SFSWAP	-0.6142	0.0845	splicing factor, suppressor of white-apricot homolog (Drosophila)
15708	MLH1	-0.6142	0.189	mutL homolog 1, colon cancer, nonpolyposis type 2 (E. coli)
15709	ECSIT	-0.6142	0.1488	ECSIT homolog (Drosophila)
15710	AP3B2	-0.6142	0.0509	adaptor-related protein complex 3, beta 2 subunit
15711	LINC00494	-0.6143	0.9996	long intergenic non-protein coding RNA 494
15712	TRIM46	-0.615	0.1974	tripartite motif containing 46
15713	LOC400657	-0.615	0.1266	uncharacterized LOC400657
15714	KCNRG	-0.615	0.0084	potassium channel regulator
15715	GPR34	-0.615	0.0692	G protein-coupled receptor 34
15716	COTL1	-0.615	0.1134	coactosin-like 1 (Dictyostelium)
15717	C1QL1	-0.615	0.194	complement component 1, q subcomponent-like 1
15718	BUD13	-0.615	0.0867	BUD13 homolog (S. cerevisiae)
15719	YWHAH	-0.6158	0.1206	tyrosine 3-monooxygenase/tryptophan 5-monooxygenase activation protein, eta polypeptide
15720	NUP107	-0.6158	0.2932	nucleoporin 107kDa
15721	NDUFAB1	-0.6158	0.244	NADH dehydrogenase (ubiquinone) 1, alpha/beta subcomplex, 1, 8kDa
15722	CHEK1	-0.6158	0.2171	checkpoint kinase 1
15723	CERK	-0.6158	0.0313	ceramide kinase
15724	ZNRD1	-0.6162	0.1326	zinc ribbon domain containing 1
15725	TBC1D25	-0.6162	0.074	TBC1 domain family, member 25
15726	SHISA7	-0.6162	0.3656	shisa homolog 7 (Xenopus laevis)
15727	PRNT	-0.6162	0.1455	prion protein (testis specific)
15728	PDZD9	-0.6162	0.0107	PDZ domain containing 9
15729	OR2L13	-0.6162	0.0069	olfactory receptor, family 2, subfamily L, member 13
15730	MAP7D2	-0.6162	0.011	MAP7 domain containing 2
15731	KIAA1324L	-0.6162	0.017	KIAA1324-like
15732	HSH2D	-0.6162	0.0555	hematopoietic SH2 domain containing
15733	FAM209B	-0.6162	0.0158	family with sequence similarity 209, member B
15734	CNDP1	-0.6162	0.0751	carnosine dipeptidase 1 (metallopeptidase M20 family)
15735	CASKIN1	-0.6162	0.2155	CASK interacting protein 1
15736	WNT6	-0.6167	0.211	wingless-type MMTV integration site family, member 6
15737	NUSAP1	-0.6167	0.2028	nucleolar and spindle associated protein 1
15738	MRPL18	-0.6167	0.225	mitochondrial ribosomal protein L18
15739	IQSEC1	-0.6167	0.0788	IQ motif and Sec7 domain 1
15740	EIF2B4	-0.6167	0.1378	eukaryotic translation initiation factor 2B, subunit 4 delta, 67kDa
15741	AMPH	-0.6167	0.0444	amphiphysin
15742	TBC1D30	-0.6171	0.9996	TBC1 domain family, member 30
15743	LOC285740	-0.6171	0.1707	uncharacterized LOC285740
15744	LOC283856	-0.6171	0.9996	uncharacterized LOC283856
15745	WNK3	-0.6175	0.0492	WNK lysine deficient protein kinase 3
15746	WBSCR28	-0.6175	0.0845	Williams-Beuren syndrome chromosome region 28
15747	PNPT1	-0.6175	0.1985	polyribonucleotide nucleotidyltransferase 1
15748	MAGOH	-0.6175	0.2477	mago-nashi homolog, proliferation-associated (Drosophila)
15749	IGSF11	-0.6175	0.0249	immunoglobulin superfamily, member 11
15750	FAM169A	-0.6175	0.0574	family with sequence similarity 169, member A
15751	COX5A	-0.6175	0.2092	cytochrome c oxidase subunit Va
15752	SPECC1L	-0.6183	0.0573	sperm antigen with calponin homology and coiled-coil domains 1-like
15753	SLC25A4	-0.6183	0.0442	solute carrier family 25 (mitochondrial carrier; adenine nucleotide translocator), member 4
15754	MPHOSPH9	-0.6183	0.2093	M-phase phosphoprotein 9
15755	C3orf79	-0.6186	0.0104	chromosome 3 open reading frame 79
15756	PRAM1	-0.6187	0.1679	PML-RARA regulated adaptor molecule 1
15757	CCDC83	-0.6187	0.0084	coiled-coil domain containing 83
15758	TUBB4A	-0.6192	0.0651	tubulin, beta 4A class IVa
15759	PRPF8	-0.6192	0.1307	PRP8 pre-mRNA processing factor 8 homolog (S. cerevisiae)
15760	MAD1L1	-0.6192	0.1125	MAD1 mitotic arrest deficient-like 1 (yeast)
15761	HNRNPR	-0.6192	0.2885	heterogeneous nuclear ribonucleoprotein R
15762	ZNF143	-0.62	0.219	zinc finger protein 143
15763	UBE2QL1	-0.62	0.0152	ubiquitin-conjugating enzyme E2Q family-like 1
15764	STAR	-0.62	0.0194	steroidogenic acute regulatory protein
15765	SNRPB2	-0.62	0.2102	small nuclear ribonucleoprotein polypeptide B
15766	SLC20A1	-0.62	0.1002	solute carrier family 20 (phosphate transporter), member 1
15767	PSMA1	-0.62	0.2603	proteasome (prosome, macropain) subunit, alpha type, 1
15768	NXF5	-0.62	0.0999	nuclear RNA export factor 5
15769	NAA60	-0.62	0.0802	N(alpha)-acetyltransferase 60, NatF catalytic subunit
15770	MPP6	-0.62	0.1048	membrane protein, palmitoylated 6 (MAGUK p55 subfamily member 6)
15771	KARS	-0.62	0.2379	lysyl-tRNA synthetase
15772	HYLS1	-0.62	0.0997	hydrolethalus syndrome 1
15773	GPR174	-0.62	0.0222	G protein-coupled receptor 174
15774	CCKBR	-0.62	0.1888	cholecystokinin B receptor
15775	CCDC67	-0.62	0.0243	coiled-coil domain containing 67
15776	C18orf12	-0.62	0.2597	chromosome 18 open reading frame 12
15777	C17orf62	-0.62	0.1147	chromosome 17 open reading frame 62
15778	ASNS	-0.62	0.0569	asparagine synthetase (glutamine-hydrolyzing)
15779	TPRKB	-0.6208	0.2769	TP53RK binding protein
15780	TERF2	-0.6208	0.0378	telomeric repeat binding factor 2
15781	NHP2	-0.6208	0.2231	NHP2 ribonucleoprotein homolog (yeast)
15782	NAE1	-0.6208	0.3022	NEDD8 activating enzyme E1 subunit 1
15783	GLO1	-0.6208	0.2257	glyoxalase I
15784	AASDHPPT	-0.6208	0.2948	aminoadipate-semialdehyde dehydrogenase-phosphopantetheinyl transferase
15785	TTC29	-0.6212	0.0094	tetratricopeptide repeat domain 29
15786	MAGEB6	-0.6212	0.005	melanoma antigen family B, 6
15787	LINC00174	-0.6212	0.1444	long intergenic non-protein coding RNA 174
15788	CCDC116	-0.6212	0.2231	coiled-coil domain containing 116
15789	CABS1	-0.6212	0.0235	calcium-binding protein, spermatid-specific 1
15790	FXR1	-0.6217	0.2089	fragile X mental retardation, autosomal homolog 1

15791	RFESD	-0.6225	0.0464	Rieske (Fe-S) domain containing
15792	PPM1B	-0.6225	0.2498	protein phosphatase, Mg2+/Mn2+ dependent, 1B
15793	PCYOX1L	-0.6225	0.0352	prenylcysteine oxidase 1 like
15794	MRPS25	-0.6225	0.1109	mitochondrial ribosomal protein S25
15795	GPR158	-0.6225	0.02	G protein-coupled receptor 158
15796	EEFSEC	-0.6225	0.0758	eukaryotic elongation factor, selenocysteine-tRNA-specific
15797	CIRH1A	-0.6225	0.1797	cirrhosis, autosomal recessive 1A (cirhin)
15798	CELSR3	-0.6225	0.0832	cadherin, EGF LAG seven-pass G-type receptor 3 (flamingo homolog, Drosophila)
15799	CCDC54	-0.6225	0.0276	coiled-coil domain containing 54
15800	C11orf71	-0.6225	0.0369	chromosome 11 open reading frame 71
15801	C11orf42	-0.6225	0.2245	chromosome 11 open reading frame 42
15802	PAM16	-0.6229	0.1352	presequence translocase-associated motor 16 homolog (S. cerevisiae)
15803	KLHL18	-0.6233	0.0295	kelch-like 18 (Drosophila)
15804	ELAC2	-0.6233	0.151	elaC homolog 2 (E. coli)
15805	ATP6V1B2	-0.6233	0.1279	ATPase, H+ transporting, lysosomal 56/58kDa, V1 subunit B2
15806	ARRB2	-0.6233	0.0747	arrestin, beta 2
15807	FAM24A	-0.6237	0.0313	family with sequence similarity 24, member A
15808	TNK2	-0.6242	0.2451	tyrosine kinase, non-receptor, 2
15809	SMC2	-0.6242	0.2827	structural maintenance of chromosomes 2
15810	MT3	-0.6242	0.0564	metallothionein 3
15811	FUT9	-0.6242	0.018	fucosyltransferase 9 (alpha (1,3) fucosyltransferase)
15812	ATP8A2	-0.6242	0.0124	ATPase, aminophospholipid transporter, class I, type 8A, member 2
15813	SNORA71B	-0.6243	0.1497	small nucleolar RNA, H/ACA box 71B
15814	ZNF92	-0.625	0.1287	zinc finger protein 92
15815	PHF5A	-0.625	0.1334	PHD finger protein 5A
15816	LETM2	-0.625	0.0103	leucine zipper-EF-hand containing transmembrane protein 2
15817	EXOSC8	-0.625	0.2686	exosome component 8
15818	CST9L	-0.625	0.0637	cystatin 9-like
15819	CNOT3	-0.625	0.1173	CCR4-NOT transcription complex, subunit 3
15820	CARHSP1	-0.625	0.0563	calcium regulated heat stable protein 1, 24kDa
15821	C20orf201	-0.625	0.2128	chromosome 20 open reading frame 201
15822	C17orf47	-0.625	0.1006	chromosome 17 open reading frame 47
15823	C15orf55	-0.625	0.3227	chromosome 15 open reading frame 55
15824	NFKBIE	-0.6258	0.071	nuclear factor of kappa light polypeptide gene enhancer in B-cells inhibitor, epsilon
15825	ZIC5	-0.6263	0.0866	Zic family member 5
15826	TAS1R2	-0.6263	0.1669	taste receptor, type 1, member 2
15827	PRSS36	-0.6263	0.2828	protease, serine, 36
15828	LOC732275	-0.6263	0.0451	uncharacterized LOC732275
15829	GPR112	-0.6263	0.0341	G protein-coupled receptor 112
15830	FLJ40292	-0.6263	0.1498	uncharacterized LOC643210
15831	CTCFL	-0.6263	0.0201	CCCTC-binding factor (zinc finger protein)-like
15832	COX6B2	-0.6263	0.2406	cytochrome c oxidase subunit VIb polypeptide 2 (testis)
15833	C6orf223	-0.6263	0.0836	chromosome 6 open reading frame 223
15834	C1orf131	-0.6263	0.1957	chromosome 1 open reading frame 131
15835	C17orf67	-0.6263	0.0347	chromosome 17 open reading frame 67
15836	C10orf62	-0.6263	0.1898	chromosome 10 open reading frame 62
15837	UQCR11	-0.6267	0.1075	ubiquinol-cytochrome c reductase, complex III subunit XI
15838	TMEM11	-0.6267	0.0963	transmembrane protein 11
15839	TEX14	-0.6267	0.0174	testis expressed 14
15840	PACRG	-0.6267	0.0792	PARK2 co-regulated
15841	CCDC94	-0.6267	0.1157	coiled-coil domain containing 94
15842	CAMTA2	-0.6267	0.0752	calmodulin binding transcription activator 2
15843	PRR19	-0.6271	0.1499	proline rich 19
15844	ZCCHC12	-0.6275	0.0527	zinc finger, CCHC domain containing 12
15845	ZCCHC10	-0.6275	0.223	zinc finger, CCHC domain containing 10
15846	SEC14L1	-0.6275	0.0529	SEC14-like 1 (S. cerevisiae)
15847	SAP30BP	-0.6275	0.061	SAP30 binding protein
15848	RPRD1A	-0.6275	0.2586	regulation of nuclear pre-mRNA domain containing 1A
15849	POP4	-0.6275	0.1865	processing of precursor 4, ribonuclease P/MRP subunit (S. cerevisiae)
15850	MARCH10	-0.6275	0.0515	membrane-associated ring finger (C3HC4) 10, E3 ubiquitin protein ligase
15851	KLB	-0.6275	0.0365	klotho beta
15852	INSM1	-0.6275	0.0297	insulinoma-associated 1
15853	USP16	-0.6283	0.2602	ubiquitin specific peptidase 16
15854	SPTB	-0.6283	0.2786	spectrin, beta, erythrocytic
15855	SAFB2	-0.6283	0.0772	scaffold attachment factor B2
15856	PAX6	-0.6283	0.01	paired box 6
15857	NEUROG2	-0.6283	0.0376	neurogenin 2
15858	CLCN6	-0.6283	0.0516	chloride channel, voltage-sensitive 6
15859	CELF2	-0.6283	0.078	CUGBP, Elav-like family member 2
15860	PLAC8L1	-0.6286	0.0658	PLAC8-like 1
15861	LINC00051	-0.6286	0.9996	long intergenic non-protein coding RNA 51
15862	ZNF829	-0.6288	0.0152	zinc finger protein 829
15863	SPATA3	-0.6288	0.2877	spermatogenesis associated 3
15864	QSOX2	-0.6288	0.0579	quiescin Q6 sulfhydryl oxidase 2
15865	C15orf32	-0.6288	0.0252	chromosome 15 open reading frame 32
15866	SRSF2	-0.6292	0.2426	serine/arginine-rich splicing factor 2
15867	HPCAL4	-0.6292	0.0182	hippocalcin like 4
15868	HMGXB4	-0.6292	0.1361	HMG box domain containing 4
15869	TESK1	-0.63	0.0709	testis-specific kinase 1
15870	OR51B4	-0.63	0.0545	olfactory receptor, family 51, subfamily B, member 4
15871	KIAA1875	-0.63	0.1318	KIAA1875
15872	FAM40B	-0.63	0.0215	family with sequence similarity 40, member B
15873	BOLL	-0.63	0.0937	bol, boule-like (Drosophila)
15874	MRPL46	-0.6308	0.1826	mitochondrial ribosomal protein L46

15875	ZNF71	-0.6313	0.0253	zinc finger protein 71
15876	FAM228A	-0.6313	0.0169	family with sequence similarity 228, member A
15877	C2CD3	-0.6313	0.015	C2 calcium-dependent domain containing 3
15878	BPIFB6	-0.6313	0.1112	BPI fold containing family B, member 6
15879	C19orf45	-0.6314	0.2887	chromosome 19 open reading frame 45
15880	BTF3L4	-0.6314	0.1213	basic transcription factor 3-like 4
15881	PPIG	-0.6317	0.1752	peptidylprolyl isomerase G (cyclophilin G)
15882	PGP	-0.6317	0.1402	phosphoglycolate phosphatase
15883	PEG10	-0.6317	0.014	paternally expressed 10
15884	ORC3	-0.6317	0.1951	origin recognition complex, subunit 3
15885	LST1	-0.6317	0.1551	leukocyte specific transcript 1
15886	PDCD11	-0.6318	0.1288	programmed cell death 11
15887	TRAPPC10	-0.6325	0.128	trafficking protein particle complex 10
15888	MAK16	-0.6325	0.2113	MAK16 homolog (S. cerevisiae)
15889	LARS2	-0.6325	0.121	leucyl-tRNA synthetase 2, mitochondrial
15890	ISM2	-0.6325	0.1763	isthmin 2 homolog (zebrafish)
15891	FEM1A	-0.6325	0.0582	fem-1 homolog a (C. elegans)
15892	FAM49B	-0.6325	0.1578	family with sequence similarity 49, member B
15893	B4GALNT4	-0.6325	0.0926	beta-1,4-N-acetyl-galactosaminyl transferase 4
15894	ATP2B3	-0.6325	0.2745	ATPase, Ca++ transporting, plasma membrane 3
15895	TOP2A	-0.6333	0.196	topoisomerase (DNA) II alpha 170kDa
15896	PTTG1	-0.6333	0.1448	pituitary tumor-transforming 1
15897	NDUFA8	-0.6333	0.1781	NADH dehydrogenase (ubiquinone) 1 alpha subcomplex, 8, 19kDa
15898	GPN3	-0.6333	0.2498	GPN-loop GTPase 3
15899	STK33	-0.6338	0.0143	serine/threonine kinase 33
15900	LINC00521	-0.6338	0.2665	long intergenic non-protein coding RNA 521
15901	KCNK16	-0.6338	0.2608	potassium channel, subfamily K, member 16
15902	FAM117B	-0.6338	0.0763	family with sequence similarity 117, member B
15903	EAF1	-0.6338	0.1278	ELL associated factor 1
15904	A1BG	-0.6338	0.0697	alpha-1-B glycoprotein
15905	SNRNP27	-0.6342	0.1849	small nuclear ribonucleoprotein 27kDa (U4/U6.U5)
15906	MAGEB1	-0.6342	0.0193	melanoma antigen family B, 1
15907	HGS	-0.6342	0.1503	hepatocyte growth factor-regulated tyrosine kinase substrate
15908	ATP5J2	-0.6342	0.1835	ATP synthase, H+ transporting, mitochondrial Fo complex, subunit F2
15909	OR6B1	-0.6343	0.2076	olfactory receptor, family 6, subfamily B, member 1
15910	C3orf67	-0.6343	0.0182	chromosome 3 open reading frame 67
15911	VPS72	-0.635	0.1194	vacuolar protein sorting 72 homolog (S. cerevisiae)
15912	TPD52L3	-0.635	0.1876	tumor protein D52-like 3
15913	SIRPB1	-0.635	0.0804	signal-regulatory protein beta 1
15914	RNF208	-0.635	0.1568	ring finger protein 208
15915	PPM1N	-0.635	0.0267	protein phosphatase, Mg2+/Mn2+ dependent, 1N (putative)
15916	MLL4	-0.635	0.1255	myeloid/lymphoid or mixed-lineage leukemia 4
15917	KLHL32	-0.635	0.0091	kelch-like 32 (Drosophila)
15918	DCUN1D1	-0.635	0.0949	DCN1, defective in cullin neddylation 1, domain containing 1 (S. cerevisiae)
15919	CCDC66	-0.635	0.1536	coiled-coil domain containing 66
15920	C17orf99	-0.635	0.062	chromosome 17 open reading frame 99
15921	C1orf126	-0.6357	0.0859	chromosome 1 open reading frame 126
15922	TUBA3C	-0.6358	0.0469	tubulin, alpha 3c
15923	SSBP1	-0.6358	0.2775	single-stranded DNA binding protein 1, mitochondrial
15924	DCLRE1A	-0.6358	0.0721	DNA cross-link repair 1A
15925	RGMA	-0.6363	0.0682	RGM domain family, member A
15926	REXO1	-0.6363	0.1292	REX1, RNA exonuclease 1 homolog (S. cerevisiae)
15927	NKAIN3	-0.6363	0.0391	Na+/K+ transporting ATPase interacting 3
15928	GPATCH4	-0.6363	0.1709	G patch domain containing 4
15929	CLEC4D	-0.6363	0.0529	C-type lectin domain family 4, member D
15930	ASB16	-0.6363	0.2953	ankyrin repeat and SOCS box containing 16
15931	CIAPIN1	-0.6367	0.1637	cytokine induced apoptosis inhibitor 1
15932	ZNF497	-0.6375	0.0612	zinc finger protein 497
15933	WDR47	-0.6375	0.1996	WD repeat domain 47
15934	STIP1	-0.6375	0.1541	stress-induced-phosphoprotein 1
15935	SNX22	-0.6375	0.1626	sorting nexin 22
15936	PROK2	-0.6375	0.0252	prokineticin 2
15937	MYEF2	-0.6375	0.0571	myelin expression factor 2
15938	INTS2	-0.6375	0.2367	integrator complex subunit 2
15939	DTX1	-0.6375	0.0537	deltex homolog 1 (Drosophila)
15940	CATSPER1	-0.6375	0.0374	cation channel, sperm associated 1
15941	TCF3	-0.6383	0.093	transcription factor 3 (E2A immunoglobulin enhancer binding factors E12/E47)
15942	PRKAR2B	-0.6383	0.0361	protein kinase, cAMP-dependent, regulatory, type II, beta
15943	PRAME	-0.6383	0.0066	preferentially expressed antigen in melanoma
15944	NIF3L1	-0.6383	0.2363	NIF3 NGG1 interacting factor 3-like 1 (S. cerevisiae)
15945	ELL	-0.6383	0.1115	elongation factor RNA polymerase II
15946	CUL9	-0.6383	0.1025	cullin 9
15947	LOC285692	-0.6386	0.9996	uncharacterized LOC285692
15948	WDPCP	-0.6388	0.0374	WD repeat containing planar cell polarity effector
15949	SLAMF6	-0.6388	0.0738	SLAM family member 6
15950	PPIL1	-0.6388	0.2029	peptidylprolyl isomerase (cyclophilin)-like 1
15951	PAIP2	-0.6388	0.1379	poly(A) binding protein interacting protein 2
15952	MGC23284	-0.6388	0.102	uncharacterized LOC197187
15953	KLF17	-0.6388	0.1535	Kruppel-like factor 17
15954	KCNK9	-0.6388	0.146	potassium channel, subfamily K, member 9
15955	ZNF821	-0.6392	0.0989	zinc finger protein 821
15956	USP11	-0.6392	0.0604	ubiquitin specific peptidase 11
15957	LYRM4	-0.6392	0.1013	LYR motif containing 4
15958	GTPBP8	-0.6392	0.1413	GTP-binding protein 8 (putative)

15959	ZNF778	-0.64	0.0087	zinc finger protein 778
15960	SMC4	-0.64	0.282	structural maintenance of chromosomes 4
15961	SHD	-0.64	0.0926	Src homology 2 domain containing transforming protein D
15962	RRP9	-0.64	0.1566	ribosomal RNA processing 9, small subunit (SSU) processome component, homolog (yeast)
15963	PRSS33	-0.64	0.2276	protease, serine, 33
15964	LINC00242	-0.64	0.0736	long intergenic non-protein coding RNA 242
15965	ILF2	-0.64	0.2418	interleukin enhancer binding factor 2, 45kDa
15966	HMGN1	-0.64	0.1648	high mobility group nucleosome binding domain 1
15967	GFAP	-0.64	0.2457	glial fibrillary acidic protein
15968	DDX39B	-0.64	0.1486	DEAD (Asp-Glu-Ala-Asp) box polypeptide 39B
15969	COX7B2	-0.64	0.0037	cytochrome c oxidase subunit VIIb2
15970	MAGEL2	-0.6408	0.0475	MAGE-like 2
15971	APOO	-0.6408	0.1189	apolipoprotein O
15972	SP7	-0.6413	0.0922	Sp7 transcription factor
15973	CCDC77	-0.6413	0.1055	coiled-coil domain containing 77
15974	OR51I2	-0.6414	0.2133	olfactory receptor, family 51, subfamily I, member 2
15975	TMEM177	-0.6425	0.1279	transmembrane protein 177
15976	NECAP1	-0.6425	0.074	NECAP endocytosis associated 1
15977	MCPH1	-0.6425	0.0096	microcephalin 1
15978	MAPK1	-0.6425	0.1347	mitogen-activated protein kinase 1
15979	HIST1H4D	-0.6425	0.0142	histone cluster 1, H4d
15980	CCT3	-0.6425	0.2323	chaperonin containing TCP1, subunit 3 (gamma)
15981	P2RY11	-0.6433	0.0938	purinergic receptor P2Y, G-protein coupled, 11
15982	METTL13	-0.6433	0.1333	methyltransferase like 13
15983	HSPA13	-0.6433	0.244	heat shock protein 70kDa family, member 13
15984	ZCCHC3	-0.6438	0.0438	zinc finger, CCHC domain containing 3
15985	SRRM4	-0.6438	0.2507	serine/arginine repetitive matrix 4
15986	SLC35B4	-0.6438	0.0522	solute carrier family 35, member B4
15987	PRIC285	-0.6438	0.1795	peroxisomal proliferator-activated receptor A interacting complex 285
15988	HSF5	-0.6438	0.0265	heat shock transcription factor family member 5
15989	TSN	-0.6442	0.265	translin
15990	YJEFN3	-0.6443	0.1524	YjeF N-terminal domain containing 3
15991	C6orf147	-0.6443	0.9996	chromosome 6 open reading frame 147
15992	UNC79	-0.645	0.0771	unc-79 homolog (C. elegans)
15993	TEX33	-0.645	0.0174	testis expressed 33
15994	RTN4RL1	-0.645	0.0223	reticulon 4 receptor-like 1
15995	PIDD	-0.645	0.1477	p53-induced death domain protein
15996	GRIK5	-0.645	0.2872	glutamate receptor, ionotropic, kainate 5
15997	ZNF324B	-0.6455	0.101	zinc finger protein 324B
15998	SAE1	-0.6458	0.1643	SUMO1 activating enzyme subunit 1
15999	KLC1	-0.6458	0.0998	kinesin light chain 1
16000	DDX11	-0.6458	0.1037	DEAD/H (Asp-Glu-Ala-Asp/His) box helicase 11
16001	ZNF382	-0.6463	0.026	zinc finger protein 382
16002	OTP	-0.6463	0.0174	orthopedia homeobox
16003	SOX1	-0.6467	0.1712	SRY (sex determining region Y)-box 1
16004	PEX5	-0.6467	0.0211	peroxisomal biogenesis factor 5
16005	NDUFS7	-0.6467	0.153	NADH dehydrogenase (ubiquinone) Fe-S protein 7, 20kDa (NADH-coenzyme Q reductase)
16006	EIF4A3	-0.6467	0.1353	eukaryotic translation initiation factor 4A3
16007	DUSP8	-0.6467	0.1066	dual specificity phosphatase 8
16008	SPATA12	-0.6475	0.0488	spermatogenesis associated 12
16009	RNF219	-0.6475	0.2667	ring finger protein 219
16010	NUP88	-0.6475	0.2322	nucleoporin 88kDa
16011	KIFC2	-0.6475	0.1452	kinesin family member C2
16012	KCNK4	-0.6475	0.1631	potassium channel, subfamily K, member 4
16013	ST8SIA3	-0.6483	0.1504	ST8 alpha-N-acetyl-neuraminide alpha-2,8-sialyltransferase 3
16014	RNF144A	-0.6483	0.0202	ring finger protein 144A
16015	SATB2-AS1	-0.6488	0.1069	SATB2 antisense RNA 1 (non-protein coding)
16016	MARCH11	-0.6488	0.0222	membrane-associated ring finger (C3HC4) 11
16017	LINC00162	-0.6488	0.1116	long intergenic non-protein coding RNA 162
16018	CYB5RL	-0.6488	0.0453	cytochrome b5 reductase-like
16019	C9orf37	-0.6488	0.0722	chromosome 9 open reading frame 37
16020	C17orf105	-0.6488	0.0225	chromosome 17 open reading frame 105
16021	ANKRD54	-0.6488	0.0594	ankyrin repeat domain 54
16022	USP7	-0.6492	0.1029	ubiquitin specific peptidase 7 (herpes virus-associated)
16023	POU2F1	-0.6492	0.0483	POU class 2 homeobox 1
16024	HTRA2	-0.6492	0.0892	HtrA serine peptidase 2
16025	TPH1	-0.65	0.0041	tryptophan hydroxylase 1
16026	SRPK1	-0.65	0.1467	SRSF protein kinase 1
16027	RPRML	-0.65	0.0773	reprimo-like
16028	LOC619207	-0.65	0.2489	scavenger receptor protein family member
16029	HTATSF1	-0.65	0.1915	HIV-1 Tat specific factor 1
16030	ZSCAN5A	-0.6508	0.015	zinc finger and SCAN domain containing 5A
16031	SF3B5	-0.6508	0.1572	splicing factor 3b, subunit 5, 10kDa
16032	SF3A1	-0.6508	0.0824	splicing factor 3a, subunit 1, 120kDa
16033	CHGB	-0.6508	0.0101	chromogranin B (secretogranin 1)
16034	SYCE1	-0.6513	0.0166	synaptonemal complex central element protein 1
16035	PHF19	-0.6513	0.1021	PHD finger protein 19
16036	FAM71A	-0.6513	0.2229	family with sequence similarity 71, member A
16037	ZNF184	-0.6517	0.1973	zinc finger protein 184
16038	SV2A	-0.6517	0.0314	synaptic vesicle glycoprotein 2A
16039	SHCBP1	-0.6517	0.1487	SHC SH2-domain binding protein 1
16040	AKAP3	-0.6517	0.0185	A kinase (PRKA) anchor protein 3
16041	UBXN11	-0.6525	0.0655	UBX domain protein 11
16042	TRIM40	-0.6525	0.0389	tripartite motif containing 40

16043	SEZ6L	-0.6525	0.2296	seizure related 6 homolog (mouse)-like
16044	RLN3	-0.6525	0.1057	relaxin 3
16045	OR4N4	-0.6525	0.0051	olfactory receptor, family 4, subfamily N, member 4
16046	HES5	-0.6525	0.0653	hairy and enhancer of split 5 (Drosophila)
16047	C20orf61	-0.6525	0.2544	chromosome 20 open reading frame 61
16048	TTC4	-0.6529	0.081	tetratricopeptide repeat domain 4
16049	YEATS2	-0.6533	0.0724	YEATS domain containing 2
16050	TRIP13	-0.6533	0.1763	thyroid hormone receptor interactor 13
16051	TOX	-0.6533	0.0087	thymocyte selection-associated high mobility group box
16052	JAKMIP2	-0.6533	0.0081	janus kinase and microtubule interacting protein 2
16053	ZCCHC13	-0.6538	0.0228	zinc finger, CCHC domain containing 13
16054	MIAT	-0.6538	0.0561	myocardial infarction associated transcript (non-protein coding)
16055	LINC00612	-0.6538	0.108	long intergenic non-protein coding RNA 612
16056	GALNTL5	-0.6538	0.0199	UDP-N-acetyl-alpha-D-galactosamine:polypeptide N-acetylgalactosaminyltransferase-like 5
16057	ZNHIT3	-0.6542	0.2043	zinc finger, HIT-type containing 3
16058	SV2B	-0.6542	0.0509	synaptic vesicle glycoprotein 2B
16059	SLA	-0.6542	0.109	Src-like-adaptor
16060	KCNN1	-0.6542	0.2629	potassium intermediate/small conductance calcium-activated channel, subfamily N, member 1
16061	CDCA4	-0.6542	0.1117	cell division cycle associated 4
16062	LOC151174	-0.6543	0.154	uncharacterized LOC151174
16063	KCNU1	-0.6543	0.9999	potassium channel, subfamily U, member 1
16064	ZBTB9	-0.655	0.0462	zinc finger and BTB domain containing 9
16065	RAD9B	-0.655	0.004	RAD9 homolog B (S. pombe)
16066	NGLY1	-0.655	0.1418	N-glycanase 1
16067	GRPEL1	-0.655	0.1474	GrpE-like 1, mitochondrial (E. coli)
16068	FBXO10	-0.655	0.0134	F-box protein 10
16069	FAM205A	-0.655	0.076	family with sequence similarity 205, member A
16070	CLPB	-0.655	0.0766	ClpB caseinolytic peptidase B homolog (E. coli)
16071	ADAT3	-0.655	0.1698	adenosine deaminase, tRNA-specific 3
16072	NANOS2	-0.6557	0.2191	nanos homolog 2 (Drosophila)
16073	TTLL4	-0.6558	0.0437	tubulin tyrosine ligase-like family, member 4
16074	SYT5	-0.6558	0.248	synaptotagmin V
16075	GPR63	-0.6558	0.0246	G protein-coupled receptor 63
16076	TMEM132C	-0.6563	0.0419	transmembrane protein 132C
16077	PPP3R2	-0.6563	0.0916	protein phosphatase 3, regulatory subunit B, beta
16078	NECAB1	-0.6563	0.0091	N-terminal EF-hand calcium binding protein 1
16079	FLJ38379	-0.6563	0.0195	uncharacterized FLJ38379
16080	FBXO31	-0.6563	0.0435	F-box protein 31
16081	DBIL5P	-0.6563	0.0176	diazepam binding inhibitor-like 5, pseudogene
16082	SOX3	-0.6567	0.1413	SRY (sex determining region Y)-box 3
16083	RNF165	-0.6571	0.0358	ring finger protein 165
16084	LOC283070	-0.6571	0.1579	uncharacterized LOC283070
16085	WDR12	-0.6575	0.2277	WD repeat domain 12
16086	TUBB3	-0.6575	0.0671	tubulin, beta 3 class III
16087	SYNPR	-0.6575	0.0462	synaptoporin
16088	SLC25A41	-0.6575	0.0378	solute carrier family 25, member 41
16089	IQCF2	-0.6575	0.0644	IQ motif containing F2
16090	INA	-0.6575	0.021	internexin neuronal intermediate filament protein, alpha
16091	GRM3	-0.6575	0.0743	glutamate receptor, metabotropic 3
16092	DIP2B	-0.6575	0.0901	DIP2 disco-interacting protein 2 homolog B (Drosophila)
16093	CSE1L	-0.6575	0.282	CSE1 chromosome segregation 1-like (yeast)
16094	BANP	-0.6575	0.0385	BTG3 associated nuclear protein
16095	B4GALT5	-0.6575	0.0339	UDP-Gal:betaGlcNAc beta 1,4- galactosyltransferase, polypeptide 5
16096	ARHGEF18	-0.6575	0.0528	Rho/Rac guanine nucleotide exchange factor (GEF) 18
16097	AKIRIN2	-0.6575	0.0426	akirin 2
16098	THOC7	-0.6583	0.2309	THO complex 7 homolog (Drosophila)
16099	RBM28	-0.6583	0.1583	RNA binding motif protein 28
16100	SLC7A3	-0.6587	0.0317	solute carrier family 7 (cationic amino acid transporter, y+ system), member 3
16101	SYNJ1	-0.6592	0.1138	synaptojanin 1
16102	GLMN	-0.6592	0.1638	glomulin, FKBP associated protein
16103	VWA3B	-0.66	0.156	von Willebrand factor A domain containing 3B
16104	USP30-AS1	-0.66	0.0431	USP30 antisense RNA 1 (non-protein coding)
16105	UROC1	-0.66	0.063	urocanase domain containing 1
16106	UBA2	-0.66	0.2397	ubiquitin-like modifier activating enzyme 2
16107	RAD54L2	-0.66	0.053	RAD54-like 2 (S. cerevisiae)
16108	LOC100133985	-0.66	0.9999	uncharacterized LOC100133985
16109	KIAA0586	-0.66	0.0556	KIAA0586
16110	GNG3	-0.66	0.0636	guanine nucleotide binding protein (G protein), gamma 3
16111	FAM32A	-0.66	0.0735	family with sequence similarity 32, member A
16112	DMRT1	-0.66	0.0149	doublesex and mab-3 related transcription factor 1
16113	CNTRL	-0.66	0.0872	centriolin
16114	C20orf132	-0.66	0.046	chromosome 20 open reading frame 132
16115	ZBTB32	-0.6608	0.1201	zinc finger and BTB domain containing 32
16116	SFI1	-0.6608	0.0908	Sfi1 homolog, spindle assembly associated (yeast)
16117	PCM1	-0.6608	0.1713	pericentriolar material 1
16118	FAM193A	-0.6608	0.0897	family with sequence similarity 193, member A
16119	FADS2	-0.6608	0.0152	fatty acid desaturase 2
16120	ERCC6L	-0.6608	0.132	excision repair cross-complementing rodent repair deficiency, complementation group 6-like
16121	ZNF598	-0.6612	0.16	zinc finger protein 598
16122	PHF21B	-0.6612	0.0543	PHD finger protein 21B
16123	LYPD4	-0.6612	0.21	LY6/PLAUR domain containing 4
16124	RP1-177G6.2	-0.6614	0.0383	uncharacterized LOC286411
16125	LINC00421	-0.6614	0.0889	long intergenic non-protein coding RNA 421
16126	C18orf42	-0.6614	0.5353	chromosome 18 open reading frame 42

16127	QRICH1	-0.6617	0.0538	glutamine-rich 1
16128	GEMIN6	-0.6617	0.1736	gem (nuclear organelle) associated protein 6
16129	CUTC	-0.6617	0.1138	cutC copper transporter homolog (E. coli)
16130	C16orf59	-0.6617	0.158	chromosome 16 open reading frame 59
16131	ZNF777	-0.6625	0.0401	zinc finger protein 777
16132	TMEM160	-0.6625	0.1465	transmembrane protein 160
16133	PSD2	-0.6625	0.105	pleckstrin and Sec7 domain containing 2
16134	PLCB2	-0.6625	0.1417	phospholipase C, beta 2
16135	PFKFB4	-0.6625	0.0283	6-phosphofructo-2-kinase/fructose-2,6-biphosphatase 4
16136	ILKAP	-0.6625	0.1044	integrin-linked kinase-associated serine/threonine phosphatase
16137	ADPRHL2	-0.6625	0.0656	ADP-ribosylhydrolase like 2
16138	SRGAP2	-0.6633	0.0207	SLIT-ROBO Rho GTPase activating protein 2
16139	CENPE	-0.6633	0.1359	centromere protein E, 312kDa
16140	NPBWR1	-0.6637	0.0597	neuropeptides B/W receptor 1
16141	LOC100130155	-0.6637	0.0749	uncharacterized LOC100130155
16142	IL20	-0.6637	0.0582	interleukin 20
16143	TPI1	-0.6642	0.1287	triosephosphate isomerase 1
16144	SNRNP25	-0.6642	0.1548	small nuclear ribonucleoprotein 25kDa (U11/U12)
16145	PPP2CA	-0.6642	0.2065	protein phosphatase 2, catalytic subunit, alpha isozyme
16146	PPP1R16B	-0.6642	0.0361	protein phosphatase 1, regulatory subunit 16B
16147	KIF14	-0.6642	0.1527	kinesin family member 14
16148	IDI1	-0.6642	0.0415	isopentenyl-diphosphate delta isomerase 1
16149	TMEM151B	-0.6645	0.2694	transmembrane protein 151B
16150	TUSC2	-0.665	0.0747	tumor suppressor candidate 2
16151	IFLTD1	-0.665	0.0678	intermediate filament tail domain containing 1
16152	CAPN13	-0.665	0.0647	calpain 13
16153	C17orf74	-0.665	0.0883	chromosome 17 open reading frame 74
16154	C10orf88	-0.665	0.104	chromosome 10 open reading frame 88
16155	LIPJ	-0.6657	0.0337	lipase, family member J
16156	C16orf91	-0.6657	0.108	chromosome 16 open reading frame 91
16157	ZNF280B	-0.6658	0.0285	zinc finger protein 280B
16158	NCOA1	-0.6658	0.0848	nuclear receptor coactivator 1
16159	HINFP	-0.6658	0.0263	histone H4 transcription factor
16160	GAD1	-0.6658	0.0091	glutamate decarboxylase 1 (brain, 67kDa)
16161	CPNE6	-0.6658	0.2661	copine VI (neuronal)
16162	C10orf137	-0.6658	0.2111	chromosome 10 open reading frame 137
16163	BNIP1	-0.6658	0.0446	BCL2/adenovirus E1B 19kDa interacting protein 1
16164	AMZ2	-0.6658	0.1089	archaelysin family metallopeptidase 2
16165	ZNF619	-0.6662	0.0198	zinc finger protein 619
16166	ZNF507	-0.6662	0.143	zinc finger protein 507
16167	ZNF317	-0.6662	0.0423	zinc finger protein 317
16168	WDR65	-0.6662	0.0314	WD repeat domain 65
16169	TMEM108	-0.6662	0.0262	transmembrane protein 108
16170	ICA1L	-0.6662	0.0199	islet cell autoantigen 1,69kDa-like
16171	ANKRD23	-0.6662	0.067	ankyrin repeat domain 23
16172	ADAMTS14	-0.6662	0.1325	ADAM metallopeptidase with thrombospondin type 1 motif, 14
16173	NME1	-0.6667	0.1982	NME/NM23 nucleoside diphosphate kinase 1
16174	DPEP3	-0.6667	0.1541	dipeptidase 3
16175	LOC100129726	-0.6671	0.0945	uncharacterized LOC100129726
16176	TAF3	-0.6675	0.0185	TAF3 RNA polymerase II, TATA box binding protein (TBP)-associated factor, 140kDa
16177	RXFP2	-0.6675	0.0329	relaxin/insulin-like family peptide receptor 2
16178	PCDHB9	-0.6675	0.0392	protocadherin beta 9
16179	LOC729970	-0.6675	0.0113	hCG2028352-like
16180	C5orf34	-0.6675	0.1362	chromosome 5 open reading frame 34
16181	ARHGAP30	-0.6675	0.1055	Rho GTPase activating protein 30
16182	STRADA	-0.6683	0.0313	STE20-related kinase adaptor alpha
16183	RFC5	-0.6683	0.2162	replication factor C (activator 1) 5, 36.5kDa
16184	NOP2	-0.6683	0.1224	NOP2 nucleolar protein homolog (yeast)
16185	DNAJC12	-0.6683	0.0167	DnaJ (Hsp40) homolog, subfamily C, member 12
16186	CCNA2	-0.6683	0.1908	cyclin A2
16187	RAB4B	-0.6686	0.0944	RAB4B, member RAS oncogene family
16188	MTBP	-0.6687	0.0884	Mdm2, transformed 3T3 cell double minute 2, p53 binding protein (mouse) binding protein, 104kDa
16189	FSTL5	-0.6687	0.0075	follistatin-like 5
16190	CALHM1	-0.6687	0.1809	calcium homeostasis modulator 1
16191	RIMS2	-0.6692	0.0277	regulating synaptic membrane exocytosis 2
16192	MRPS22	-0.6692	0.1237	mitochondrial ribosomal protein S22
16193	DCLRE1B	-0.6692	0.0524	DNA cross-link repair 1B
16194	CDK5R2	-0.6692	0.2004	cyclin-dependent kinase 5, regulatory subunit 2 (p39)
16195	CCDC70	-0.6692	0.2359	coiled-coil domain containing 70
16196	ZNF541	-0.67	0.0513	zinc finger protein 541
16197	ZAN	-0.67	0.2573	zonadhesin
16198	URB2	-0.67	0.1003	URB2 ribosome biogenesis 2 homolog (S. cerevisiae)
16199	TRPV3	-0.67	0.1331	transient receptor potential cation channel, subfamily V, member 3
16200	RGS9	-0.67	0.0125	regulator of G-protein signaling 9
16201	PDCL2	-0.67	0.0047	phosducin-like 2
16202	NIN	-0.67	0.069	ninein (GSK3B interacting protein)
16203	GNB1L	-0.67	0.1156	guanine nucleotide binding protein (G protein), beta polypeptide 1-like
16204	CYP8B1	-0.67	0.0608	cytochrome P450, family 8, subfamily B, polypeptide 1
16205	ANGPTL6	-0.67	0.0681	angiopoietin-like 6
16206	SNAPC2	-0.6708	0.1146	small nuclear RNA activating complex, polypeptide 2, 45kDa
16207	EIF4A2	-0.6708	0.1521	eukaryotic translation initiation factor 4A2
16208	ZNF527	-0.6712	0.0153	zinc finger protein 527
16209	DDX55	-0.6712	0.1333	DEAD (Asp-Glu-Ala-Asp) box polypeptide 55
16210	C5orf20	-0.6712	0.0539	chromosome 5 open reading frame 20

16211	C21orf67	-0.6712	0.2255	chromosome 21 open reading frame 67
16212	R3HCC1	-0.6717	0.0655	R3H domain and coiled-coil containing 1
16213	HAUS6	-0.6717	0.2197	HAUS augmin-like complex, subunit 6
16214	SEZ6	-0.6725	0.2146	seizure related 6 homolog (mouse)
16215	LOC100130264	-0.6725	0.1594	uncharacterized LOC100130264
16216	IQCF3	-0.6725	0.0882	IQ motif containing F3
16217	GJA10	-0.6725	0.0548	gap junction protein, alpha 10, 62kDa
16218	GFOD1	-0.6725	0.0088	glucose-fructose oxidoreductase domain containing 1
16219	FERD3L	-0.6725	0.2068	Fer3-like (Drosophila)
16220	DZIP3	-0.6725	0.0923	DAZ interacting protein 3, zinc finger
16221	COPS5	-0.6725	0.2477	COP9 constitutive photomorphogenic homolog subunit 5 (Arabidopsis)
16222	C1orf158	-0.6725	0.112	chromosome 1 open reading frame 158
16223	TMEM235	-0.6729	0.9996	transmembrane protein 235
16224	DENND4B	-0.6733	0.0814	DENN/MADD domain containing 4B
16225	PXT1	-0.6737	0.0112	peroxisomal, testis specific 1
16226	IBA57	-0.6737	0.0253	IBA57, iron-sulfur cluster assembly homolog (S. cerevisiae)
16227	DEFB125	-0.6737	0.0187	defensin, beta 125
16228	CCDC108	-0.6737	0.1601	coiled-coil domain containing 108
16229	ZBTB48	-0.6742	0.0956	zinc finger and BTB domain containing 48
16230	NUDT2	-0.6742	0.0335	nudix (nucleoside diphosphate linked moiety X)-type motif 2
16231	ARHGDIG	-0.6742	0.1118	Rho GDP dissociation inhibitor (GDI) gamma
16232	PSKH2	-0.675	0.0805	protein serine kinase H2
16233	NKRF	-0.675	0.0852	NFKB repressing factor
16234	FLJ30679	-0.675	0.0527	uncharacterized protein FLJ30679
16235	C3orf37	-0.675	0.0358	chromosome 3 open reading frame 37
16236	C3orf30	-0.675	0.0244	chromosome 3 open reading frame 30
16237	C1QBP	-0.675	0.2394	complement component 1, q subcomponent binding protein
16238	PPFIA3	-0.6758	0.0802	protein tyrosine phosphatase, receptor type, f polypeptide (PTPRF), interacting protein (liprin), alpha 3
16239	GCDH	-0.6758	0.1087	glutaryl-CoA dehydrogenase
16240	ACSL6	-0.6758	0.011	acyl-CoA synthetase long-chain family member 6
16241	LHFPL3	-0.6762	0.0397	lipoma HMGIC fusion partner-like 3
16242	FAM47A	-0.6762	0.0125	family with sequence similarity 47, member A
16243	DCAF4L2	-0.6762	0.0336	DDB1 and CUL4 associated factor 4-like 2
16244	ZNF165	-0.6767	0.0254	zinc finger protein 165
16245	GNL3	-0.6767	0.1922	guanine nucleotide binding protein-like 3 (nucleolar)
16246	ADRM1	-0.6767	0.1621	adhesion regulating molecule 1
16247	PRKRIP1	-0.6771	0.0423	PRKR interacting protein 1 (IL11 inducible)
16248	ZFP1	-0.6775	0.1355	zinc finger protein 1 homolog (mouse)
16249	SNRPA	-0.6775	0.1653	small nuclear ribonucleoprotein polypeptide A
16250	RGL4	-0.6775	0.0452	ral guanine nucleotide dissociation stimulator-like 4
16251	LACE1	-0.6775	0.0074	lactation elevated 1
16252	INSM2	-0.6775	0.0985	insulinoma-associated 2
16253	FAM75E1	-0.6775	0.2111	family with sequence similarity 75, member E1
16254	CSPG5	-0.6775	0.0153	chondroitin sulfate proteoglycan 5 (neuroglycan C)
16255	C1orf61	-0.6775	0.0835	chromosome 1 open reading frame 61
16256	AP3D1	-0.6775	0.0678	adaptor-related protein complex 3, delta 1 subunit
16257	SYT11	-0.6783	0.0314	synaptotagmin XI
16258	LSM3	-0.6783	0.184	LSM3 homolog, U6 small nuclear RNA associated (S. cerevisiae)
16259	EDC3	-0.6783	0.0461	enhancer of mRNA decapping 3 homolog (S. cerevisiae)
16260	DPH2	-0.6783	0.1049	DPH2 homolog (S. cerevisiae)
16261	TMEM194B	-0.6786	0.0177	transmembrane protein 194B
16262	LOC283761	-0.6786	0.9996	uncharacterized LOC283761
16263	RRM1	-0.6792	0.2258	ribonucleotide reductase M1
16264	KIF18A	-0.6792	0.1442	kinesin family member 18A
16265	IZUMO4	-0.6792	0.1222	IZUMO family member 4
16266	CAPRIN1	-0.6792	0.2418	cell cycle associated protein 1
16267	ZNF625	-0.68	0.0113	zinc finger protein 625
16268	ZNF142	-0.68	0.0516	zinc finger protein 142
16269	SSR4P1	-0.68	0.027	signal sequence receptor, delta pseudogene 1
16270	RIMKLA	-0.68	0.0649	ribosomal modification protein rimK-like family member A
16271	ODF4	-0.68	0.1869	outer dense fiber of sperm tails 4
16272	LOC401068	-0.68	0.9996	uncharacterized LOC401068
16273	KCNH3	-0.68	0.2094	potassium voltage-gated channel, subfamily H (eag-related), member 3
16274	DPF2	-0.68	0.0334	D4, zinc and double PHD fingers family 2
16275	BCL2L12	-0.68	0.1358	BCL2-like 12 (proline rich)
16276	ZNF200	-0.6808	0.0765	zinc finger protein 200
16277	TAF11	-0.6808	0.1442	TAF11 RNA polymerase II, TATA box binding protein (TBP)-associated factor, 28kDa
16278	REEP1	-0.6808	0.0257	receptor accessory protein 1
16279	PPID	-0.6817	0.1725	peptidylprolyl isomerase D
16280	GTF3C5	-0.6817	0.1111	general transcription factor IIIC, polypeptide 5, 63kDa
16281	ACTR6	-0.6817	0.274	ARP6 actin-related protein 6 homolog (yeast)
16282	VWA7	-0.6825	0.2324	von Willebrand factor A domain containing 7
16283	TXNDC2	-0.6825	0.1653	thioredoxin domain containing 2 (spermatozoa)
16284	MGC16142	-0.6825	0.0145	uncharacterized protein MGC16142
16285	HMGB2	-0.6825	0.1777	high mobility group box 2
16286	EMC6	-0.6825	0.1029	ER membrane protein complex subunit 6
16287	C19orf73	-0.6825	0.0831	chromosome 19 open reading frame 73
16288	LSAMP-AS3	-0.6829	0.9996	LSAMP antisense RNA 3 (non-protein coding)
16289	SLC25A32	-0.6833	0.2113	solute carrier family 25 (mitochondrial folate carrier) , member 32
16290	PSMD1	-0.6833	0.1537	proteasome (prosome, macropain) 26S subunit, non-ATPase, 1
16291	POLR2E	-0.6833	0.157	polymerase (RNA) II (DNA directed) polypeptide E, 25kDa
16292	LSM2	-0.6833	0.1501	LSM2 homolog, U6 small nuclear RNA associated (S. cerevisiae)
16293	EPS15L1	-0.6833	0.0215	epidermal growth factor receptor pathway substrate 15-like 1
16294	CEP55	-0.6833	0.1584	centrosomal protein 55kDa

16295	CCDC101	-0.6833	0.0384	coiled-coil domain containing 101
16296	OR8G1	-0.6837	0.0424	olfactory receptor, family 8, subfamily G, member 1
16297	UBP1	-0.6842	0.1416	upstream binding protein 1 (LBP-1a)
16298	C1orf50	-0.6842	0.034	chromosome 1 open reading frame 50
16299	BRCA1	-0.6842	0.0954	breast cancer 1, early onset
16300	MESP2	-0.6843	0.3271	mesoderm posterior 2 homolog (mouse)
16301	TAAR9	-0.685	0.0381	trace amine associated receptor 9 (gene/pseudogene)
16302	CITED1	-0.685	0.0163	Cbp/p300-interacting transactivator, with Glu/Asp-rich carboxy-terminal domain, 1
16303	BUD31	-0.685	0.1124	BUD31 homolog (S. cerevisiae)
16304	ANKRD24	-0.685	0.2352	ankyrin repeat domain 24
16305	ZNF839	-0.6858	0.0424	zinc finger protein 839
16306	SURF2	-0.6858	0.112	surfeit 2
16307	TAKR	-0.6862	0.0351	aldo-keto reductase, truncated
16308	C2orf77	-0.6862	0.0224	chromosome 2 open reading frame 77
16309	C11orf31	-0.6862	0.0697	chromosome 11 open reading frame 31
16310	NRXN1	-0.6867	0.1152	neurexin 1
16311	MORC2	-0.6867	0.062	MORC family CW-type zinc finger 2
16312	LDHC	-0.6867	0.0101	lactate dehydrogenase C
16313	HERC5	-0.6867	0.022	HECT and RLD domain containing E3 ubiquitin protein ligase 5
16314	SRRM5	-0.6871	0.9999	serine/arginine repetitive matrix 5
16315	SIRT6	-0.6875	0.1285	sirtuin 6
16316	POLR1A	-0.6875	0.0422	polymerase (RNA) I polypeptide A, 194kDa
16317	ODF3L2	-0.6875	0.2627	outer dense fiber of sperm tails 3-like 2
16318	NNAT	-0.6875	0.0754	neuronatin
16319	NIM1	-0.6875	0.0138	serine/threonine-protein kinase NIM1
16320	ARL6IP6	-0.6875	0.0927	ADP-ribosylation-like factor 6 interacting protein 6
16321	TMEM97	-0.6883	0.103	transmembrane protein 97
16322	GGA1	-0.6883	0.0441	golgi-associated, gamma adaptin ear containing, ARF binding protein 1
16323	DEPDC5	-0.6883	0.024	DEP domain containing 5
16324	CTNNA2	-0.6883	0.0109	catenin (cadherin-associated protein), alpha 2
16325	CNOT7	-0.6883	0.2128	CCR4-NOT transcription complex, subunit 7
16326	LINC00282	-0.6888	0.0113	long intergenic non-protein coding RNA 282
16327	UBE2E1	-0.6892	0.139	ubiquitin-conjugating enzyme E2E 1
16328	RUNDC3A	-0.6892	0.1241	RUN domain containing 3A
16329	HIST1H3D	-0.6892	0.0064	histone cluster 1, H3d
16330	ADCY1	-0.6892	0.0814	adenylate cyclase 1 (brain)
16331	ACD	-0.6892	0.1003	adrenocortical dysplasia homolog (mouse)
16332	ZNF700	-0.69	0.1267	zinc finger protein 700
16333	PROKR2	-0.69	0.0408	prokineticin receptor 2
16334	NKD1	-0.69	0.1132	naked cuticle homolog 1 (Drosophila)
16335	MAST3	-0.69	0.0661	microtubule associated serine/threonine kinase 3
16336	LTV1	-0.69	0.1387	LTV1 homolog (S. cerevisiae)
16337	HIST1H1T	-0.69	0.0118	histone cluster 1, H1t
16338	HEATR4	-0.69	0.0073	HEAT repeat containing 4
16339	C4orf45	-0.69	0.0105	chromosome 4 open reading frame 45
16340	ALKBH2	-0.69	0.078	alkB, alkylation repair homolog 2 (E. coli)
16341	TUBB	-0.6908	0.1364	tubulin, beta class I
16342	MYL6B	-0.6908	0.0673	myosin, light chain 6B, alkali, smooth muscle and non-muscle
16343	TRIM69	-0.6913	0.0239	tripartite motif containing 69
16344	NXNL1	-0.6913	0.2291	nucleoredoxin-like 1
16345	FAM187B	-0.6913	0.0908	family with sequence similarity 187, member B
16346	GNN	-0.6914	0.1555	Grp94 neighboring nucleotidase pseudogene
16347	NCAPG2	-0.6917	0.1591	non-SMC condensin II complex, subunit G2
16348	YSK4	-0.6925	0.0093	YSK4 Sps1/Ste20-related kinase homolog (S. cerevisiae)
16349	TTR	-0.6925	0.0464	transthyretin
16350	TTLL11	-0.6925	0.0145	tubulin tyrosine ligase-like family, member 11
16351	SOX2	-0.6925	0.0155	SRY (sex determining region Y)-box 2
16352	MPP2	-0.6925	0.0958	membrane protein, palmitoylated 2 (MAGUK p55 subfamily member 2)
16353	LPPR5	-0.6925	0.0317	lipid phosphate phosphatase-related protein type 5
16354	LOC284801	-0.6925	0.0045	uncharacterized LOC284801
16355	LINC00626	-0.6925	0.089	long intergenic non-protein coding RNA 626
16356	BAIAP3	-0.6925	0.16	BAI1-associated protein 3
16357	TCF19	-0.6938	0.0649	transcription factor 19
16358	SPACA3	-0.6938	0.0503	sperm acrosome associated 3
16359	CCDC88C	-0.6938	0.0372	coiled-coil domain containing 88C
16360	NPTXR	-0.6942	0.0171	neuronal pentraxin receptor
16361	HOXB-AS5	-0.6943	0.9996	HOXB cluster antisense RNA 5 (non-protein coding)
16362	USP1	-0.695	0.2811	ubiquitin specific peptidase 1
16363	NAIF1	-0.695	0.0124	nuclear apoptosis inducing factor 1
16364	MED25	-0.695	0.0628	mediator complex subunit 25
16365	ENTHD1	-0.695	0.0058	ENTH domain containing 1
16366	CSRP2BP	-0.695	0.0337	CSRP2 binding protein
16367	CACNA1E	-0.695	0.1787	calcium channel, voltage-dependent, R type, alpha 1E subunit
16368	CABIN1	-0.695	0.1036	calcineurin binding protein 1
16369	BRPF1	-0.695	0.0446	bromodomain and PHD finger containing, 1
16370	AURKAIP1	-0.695	0.168	aurora kinase A interacting protein 1
16371	IQSEC3	-0.6956	0.1925	IQ motif and Sec7 domain 3
16372	MED9	-0.6958	0.0227	mediator complex subunit 9
16373	MAPK8IP2	-0.6958	0.1925	mitogen-activated protein kinase 8 interacting protein 2
16374	GPR173	-0.6958	0.1247	G protein-coupled receptor 173
16375	ZNF275	-0.6963	0.0259	zinc finger protein 275
16376	RDM1	-0.6963	0.0379	RAD52 motif 1
16377	FAM69B	-0.6963	0.0648	family with sequence similarity 69, member B
16378	DNAJC5G	-0.6963	0.1281	DnaJ (Hsp40) homolog, subfamily C, member 5 gamma

16379	VBP1	-0.6967	0.2692	von Hippel-Lindau binding protein 1
16380	USP39	-0.6967	0.0908	ubiquitin specific peptidase 39
16381	CPSF1	-0.6967	0.1104	cleavage and polyadenylation specific factor 1, 160kDa
16382	TMEM170A	-0.6975	0.0355	transmembrane protein 170A
16383	FAM212A	-0.6975	0.062	family with sequence similarity 212, member A
16384	ERMN	-0.6975	0.0115	ermin, ERM-like protein
16385	DOK3	-0.6975	0.0781	docking protein 3
16386	CCHCR1	-0.6975	0.0709	coiled-coil alpha-helical rod protein 1
16387	CALN1	-0.6975	0.0544	calneuron 1
16388	REXO4	-0.6983	0.0788	REX4, RNA exonuclease 4 homolog (S. cerevisiae)
16389	PIN1P1	-0.6983	0.1763	peptidylprolyl cis/trans isomerase, NIMA-interacting 1 pseudogene 1
16390	KPNB1	-0.6983	0.1188	karyopherin (importin) beta 1
16391	GOLGA7	-0.6983	0.0965	golgin A7
16392	COMMD3	-0.6983	0.0525	COMM domain containing 3
16393	OPN5	-0.6986	0.2271	opsin 5
16394	UBE2DNL	-0.6988	0.2186	ubiquitin-conjugating enzyme E2D N-terminal like (pseudogene)
16395	RBM17	-0.6988	0.059	RNA binding motif protein 17
16396	CCDC97	-0.6988	0.0308	coiled-coil domain containing 97
16397	ZIC2	-0.7	0.0106	Zic family member 2
16398	TTLL6	-0.7	0.0617	tubulin tyrosine ligase-like family, member 6
16399	PINX1	-0.7	0.0541	PIN2/TERF1 interacting, telomerase inhibitor 1
16400	OR2C3	-0.7	0.0103	olfactory receptor, family 2, subfamily C, member 3
16401	NEURL4	-0.7	0.0805	neuralized homolog 4 (Drosophila)
16402	KRTAP8-1	-0.7	0.1797	keratin associated protein 8-1
16403	IWS1	-0.7	0.0333	IWS1 homolog (S. cerevisiae)
16404	HSD11B1L	-0.7	0.1557	hydroxysteroid (11-beta) dehydrogenase 1-like
16405	CDC5L	-0.7	0.1809	CDC5 cell division cycle 5-like (S. pombe)
16406	C1orf94	-0.7013	0.126	chromosome 1 open reading frame 94
16407	ASB17	-0.7013	0.0114	ankyrin repeat and SOCS box containing 17
16408	PRC1	-0.7017	0.1395	protein regulator of cytokinesis 1
16409	CYB5R4	-0.7017	0.1204	cytochrome b5 reductase 4
16410	CHD5	-0.7017	0.1843	chromodomain helicase DNA binding protein 5
16411	ATP6V1E1	-0.7017	0.0624	ATPase, H+ transporting, lysosomal 31kDa, V1 subunit E1
16412	ARID3A	-0.7017	0.0237	AT rich interactive domain 3A (BRIGHT-like)
16413	TTL	-0.7025	0.0406	tubulin tyrosine ligase
16414	SUN3	-0.7025	0.0105	Sad1 and UNC84 domain containing 3
16415	LRP3	-0.7025	0.1297	low density lipoprotein receptor-related protein 3
16416	C11orf94	-0.7029	0.0247	chromosome 11 open reading frame 94
16417	SOBP	-0.7033	0.0202	sine oculis binding protein homolog (Drosophila)
16418	IMMT	-0.7033	0.1425	inner membrane protein, mitochondrial
16419	COIL	-0.7033	0.1561	coilin
16420	TMC8	-0.7038	0.0933	transmembrane channel-like 8
16421	DHX37	-0.7038	0.1237	DEAH (Asp-Glu-Ala-His) box polypeptide 37
16422	CMC1	-0.7038	0.1116	COX assembly mitochondrial protein 1 homolog (S. cerevisiae)
16423	RNMT	-0.7042	0.0739	RNA (guanine-7-) methyltransferase
16424	LOC255025	-0.7043	0.9996	uncharacterized LOC255025
16425	C8orf74	-0.7043	0.2971	chromosome 8 open reading frame 74
16426	NYAP2	-0.705	0.0347	neuronal tyrosine-phosphorylated phosphoinositide-3-kinase adaptor 2
16427	HSPA14	-0.705	0.1889	heat shock 70kDa protein 14
16428	FLJ37201	-0.705	0.02	tigger transposable element derived 2 pseudogene
16429	FDXACB1	-0.705	0.0083	ferredoxin-fold anticodon binding domain containing 1
16430	DDN	-0.705	0.1426	dendrin
16431	CDCA7	-0.705	0.1156	cell division cycle associated 7
16432	C1orf212	-0.705	0.0448	chromosome 1 open reading frame 212
16433	SMNDC1	-0.7058	0.2414	survival motor neuron domain containing 1
16434	PRKCB	-0.7058	0.0412	protein kinase C, beta
16435	HSPBP1	-0.7058	0.1509	HSPA (heat shock 70kDa) binding protein, cytoplasmic cochaperone 1
16436	TTBK1	-0.7063	0.2403	tau tubulin kinase 1
16437	MRPL1	-0.7063	0.1715	mitochondrial ribosomal protein L1
16438	MIXL1	-0.7063	0.1014	Mix paired-like homeobox
16439	GDPD1	-0.7063	0.0188	glycerophosphodiester phosphodiesterase domain containing 1
16440	EPHA10	-0.7063	0.2324	EPH receptor A10
16441	CD300LB	-0.7063	0.1872	CD300 molecule-like family member b
16442	RRM2	-0.7067	0.1409	ribonucleotide reductase M2
16443	IP6K1	-0.7067	0.0286	inositol hexakisphosphate kinase 1
16444	COPS7B	-0.7067	0.0439	COP9 constitutive photomorphogenic homolog subunit 7B (Arabidopsis)
16445	CENPO	-0.7067	0.0718	centromere protein O
16446	CDC6	-0.7067	0.1313	cell division cycle 6 homolog (S. cerevisiae)
16447	XRCC1	-0.7075	0.0448	X-ray repair complementing defective repair in Chinese hamster cells 1
16448	SUV39H1	-0.7075	0.0969	suppressor of variegation 3-9 homolog 1 (Drosophila)
16449	POLQ	-0.7075	0.103	polymerase (DNA directed), theta
16450	GPR6	-0.7075	0.1111	G protein-coupled receptor 6
16451	CECR5	-0.7075	0.0867	cat eye syndrome chromosome region, candidate 5
16452	CECR2	-0.7075	0.0762	cat eye syndrome chromosome region, candidate 2
16453	CCNB1	-0.7075	0.1782	cyclin B1
16454	CACNA1A	-0.7083	0.1477	calcium channel, voltage-dependent, P/Q type, alpha 1A subunit
16455	ZC3H8	-0.7088	0.1011	zinc finger CCCH-type containing 8
16456	TBC1D26	-0.7088	0.1012	TBC1 domain family, member 26
16457	PPP1R1C	-0.7088	0.0083	protein phosphatase 1, regulatory (inhibitor) subunit 1C
16458	FAM46D	-0.7088	0.0069	family with sequence similarity 46, member D
16459	DCLK3	-0.7088	0.0083	doublecortin-like kinase 3
16460	SHANK1	-0.7092	0.1399	SH3 and multiple ankyrin repeat domains 1
16461	NCBP2	-0.7092	0.1865	nuclear cap binding protein subunit 2, 20kDa
16462	GADD45GIP1	-0.7092	0.1545	growth arrest and DNA-damage-inducible, gamma interacting protein 1

16463	ZC3H12B	-0.71	0.045	zinc finger CCCH-type containing 12B
16464	FAXC	-0.71	0.0145	failed axon connections homolog (Drosophila)
16465	DNAH2	-0.71	0.0458	dynein, axonemal, heavy chain 2
16466	BRD4	-0.71	0.0733	bromodomain containing 4
16467	SFPQ	-0.7108	0.1598	splicing factor proline/glutamine-rich
16468	MICAL3	-0.7108	0.0186	microtubule associated monoxygenase, calponin and LIM domain containing 3
16469	SOX5	-0.7117	0.0043	SRY (sex determining region Y)-box 5
16470	RFC3	-0.7117	0.1756	replication factor C (activator 1) 3, 38kDa
16471	RAB6B	-0.7117	0.042	RAB6B, member RAS oncogene family
16472	MCM10	-0.7117	0.1465	minichromosome maintenance complex component 10
16473	UCA1	-0.7125	0.0213	urothelial cancer associated 1 (non-protein coding)
16474	MAPK8IP3	-0.7125	0.1849	mitogen-activated protein kinase 8 interacting protein 3
16475	LOC728537	-0.7125	0.0269	uncharacterized LOC728537
16476	LOC150568	-0.7125	0.0047	uncharacterized LOC150568
16477	GRIN1	-0.7125	0.3265	glutamate receptor, ionotropic, N-methyl D-aspartate 1
16478	EIF4ENIF1	-0.7125	0.0538	eukaryotic translation initiation factor 4E nuclear import factor 1
16479	CWC27	-0.7125	0.1247	CWC27 spliceosome-associated protein homolog (S. cerevisiae)
16480	CCDC110	-0.7125	0.006	coiled-coil domain containing 110
16481	C6orf201	-0.7125	0.0143	chromosome 6 open reading frame 201
16482	C14orf183	-0.7125	0.0375	chromosome 14 open reading frame 183
16483	RIMS3	-0.7133	0.0176	regulating synaptic membrane exocytosis 3
16484	RAD23A	-0.7133	0.0888	RAD23 homolog A (S. cerevisiae)
16485	PLCL2	-0.7133	0.0212	phospholipase C-like 2
16486	C12orf43	-0.7133	0.0423	chromosome 12 open reading frame 43
16487	UBE2J2	-0.7138	0.065	ubiquitin-conjugating enzyme E2, J2
16488	SNAI3	-0.7138	0.042	snail homolog 3 (Drosophila)
16489	MEI1	-0.7138	0.0953	meiosis inhibitor 1
16490	LINC00593	-0.7138	0.1887	long intergenic non-protein coding RNA 593
16491	NEK4	-0.7142	0.2177	NIMA (never in mitosis gene a)-related kinase 4
16492	MSH2	-0.7142	0.2366	mutS homolog 2, colon cancer, nonpolyposis type 1 (E. coli)
16493	CR1L	-0.7143	0.0175	complement component (3b/4b) receptor 1-like
16494	ZNF775	-0.715	0.0952	zinc finger protein 775
16495	OSBPL6	-0.715	0.0072	oxysterol binding protein-like 6
16496	NDUFB6	-0.715	0.125	NADH dehydrogenase (ubiquinone) 1 beta subcomplex, 6, 17kDa
16497	ITGB3BP	-0.715	0.1436	integrin beta 3 binding protein (beta3-endonexin)
16498	JPH4	-0.7157	0.1605	junctophilin 4
16499	SNRPF	-0.7158	0.1875	small nuclear ribonucleoprotein polypeptide F
16500	MAGEF1	-0.7158	0.0295	melanoma antigen family F, 1
16501	CCNH	-0.7158	0.102	cyclin H
16502	TCEANC2	-0.7163	0.0148	transcription elongation factor A (SII) N-terminal and central domain containing 2
16503	GPR62	-0.7163	0.1889	G protein-coupled receptor 62
16504	C2orf51	-0.7163	0.1881	chromosome 2 open reading frame 51
16505	ANKRD39	-0.7163	0.0699	ankyrin repeat domain 39
16506	RLF	-0.7167	0.0629	rearranged L-myc fusion
16507	GINS1	-0.7167	0.1429	GINS complex subunit 1 (Psf1 homolog)
16508	CACNG3	-0.7167	0.11	calcium channel, voltage-dependent, gamma subunit 3
16509	ATP5J	-0.7167	0.1105	ATP synthase, H+ transporting, mitochondrial Fo complex, subunit F6
16510	VPS13A-AS1	-0.7171	0.9999	VPS13A antisense RNA 1 (non-protein coding)
16511	PHF2P1	-0.7171	0.9996	PHD finger protein 2 pseudogene 1
16512	LOC284889	-0.7171	0.0167	uncharacterized LOC284889
16513	SAPCD2	-0.7175	0.0993	suppressor APC domain containing 2
16514	NGDN	-0.7175	0.1261	neuroguidin, EIF4E binding protein
16515	MSH6	-0.7175	0.1978	mutS homolog 6 (E. coli)
16516	FOXM1	-0.7175	0.1181	forkhead box M1
16517	C9orf43	-0.7175	0.0029	chromosome 9 open reading frame 43
16518	C12orf40	-0.7175	0.003	chromosome 12 open reading frame 40
16519	ATP5O	-0.7175	0.1639	ATP synthase, H+ transporting, mitochondrial F1 complex, O subunit
16520	TPTE	-0.7183	0.0064	transmembrane phosphatase with tensin homology
16521	C4orf27	-0.7183	0.1132	chromosome 4 open reading frame 27
16522	CAGE1	-0.7186	0.0083	cancer antigen 1
16523	TNFAIP8L2	-0.7188	0.0693	tumor necrosis factor, alpha-induced protein 8-like 2
16524	PRSS58	-0.7188	0.031	protease, serine, 58
16525	DEPDC4	-0.7188	0.0135	DEP domain containing 4
16526	BHLHE22	-0.7188	0.047	basic helix-loop-helix family, member e22
16527	ATXN7L3	-0.7188	0.0386	ataxin 7-like 3
16528	ANKRD31	-0.7188	0.0065	ankyrin repeat domain 31
16529	KATNB1	-0.7192	0.0712	katanin p80 (WD repeat containing) subunit B 1
16530	ESPL1	-0.7192	0.1024	extra spindle pole bodies homolog 1 (S. cerevisiae)
16531	D4S234E	-0.7192	0.0087	DNA segment on chromosome 4 (unique) 234 expressed sequence
16532	ARMC8	-0.7192	0.1379	armadillo repeat containing 8
16533	PRSS37	-0.72	0.1002	protease, serine, 37
16534	LRIF1	-0.72	0.1653	ligand dependent nuclear receptor interacting factor 1
16535	FAT3	-0.72	0.0274	FAT tumor suppressor homolog 3 (Drosophila)
16536	FAM161A	-0.72	0.0658	family with sequence similarity 161, member A
16537	EIF4E	-0.72	0.1295	eukaryotic translation initiation factor 4E
16538	DDX20	-0.72	0.133	DEAD (Asp-Glu-Ala-Asp) box polypeptide 20
16539	SFMBT1	-0.7208	0.0129	Scm-like with four mbt domains 1
16540	CCDC28B	-0.7208	0.0412	coiled-coil domain containing 28B
16541	TCAM1P	-0.7212	0.006	testicular cell adhesion molecule 1 homolog (mouse), pseudogene
16542	SENP1	-0.7212	0.0754	SUMO1/sentrin specific peptidase 1
16543	PCDH10	-0.7212	0.0154	protocadherin 10
16544	FAM104A	-0.7212	0.0073	family with sequence similarity 104, member A
16545	SOGA3	-0.7214	0.0366	SOGA family member 3
16546	SIVA1	-0.7217	0.1305	SIVA1, apoptosis-inducing factor

16547	THEMIS2	-0.7225	0.0587	thymocyte selection associated family member 2
16548	FAM209A	-0.7225	0.0106	family with sequence similarity 209, member A
16549	DDX25	-0.7225	0.0224	DEAD (Asp-Glu-Ala-Asp) box helicase 25
16550	ATP8B5P	-0.7225	0.0098	ATPase, class I, type 8B, member 5, pseudogene
16551	ARMC6	-0.7225	0.1336	armadillo repeat containing 6
16552	AMD1	-0.7225	0.1068	adenosylmethionine decarboxylase 1
16553	GNL2	-0.7233	0.1725	guanine nucleotide binding protein-like 2 (nucleolar)
16554	ERI3	-0.7233	0.0765	ERI1 exoribonuclease family member 3
16555	CTNNBL1	-0.7233	0.0427	catenin, beta like 1
16556	CETN3	-0.7233	0.2264	centrin, EF-hand protein, 3
16557	CACNA2D2	-0.7233	0.028	calcium channel, voltage-dependent, alpha 2/delta subunit 2
16558	AP2S1	-0.7233	0.1275	adaptor-related protein complex 2, sigma 1 subunit
16559	C1orf52	-0.7237	0.0682	chromosome 1 open reading frame 52
16560	NEK2	-0.7242	0.0733	NIMA (never in mitosis gene a)-related kinase 2
16561	NDUFS3	-0.7242	0.1468	NADH dehydrogenase (ubiquinone) Fe-S protein 3, 30kDa (NADH-coenzyme Q reductase)
16562	BUB1B	-0.7242	0.1449	budding uninhibited by benzimidazoles 1 homolog beta (yeast)
16563	USP37	-0.725	0.1159	ubiquitin specific peptidase 37
16564	CNTNAP2	-0.725	0.0168	contactin associated protein-like 2
16565	ANAPC11	-0.725	0.0763	anaphase promoting complex subunit 11
16566	RNFT2	-0.7258	0.0291	ring finger protein, transmembrane 2
16567	PPM1G	-0.7258	0.1365	protein phosphatase, Mg2+/Mn2+ dependent, 1G
16568	LIMD2	-0.7258	0.0885	LIM domain containing 2
16569	LAGE3	-0.7258	0.0909	L antigen family, member 3
16570	CDC42	-0.7258	0.0214	cell division cycle 42 (GTP binding protein, 25kDa)
16571	OR10A5	-0.7262	0.0462	olfactory receptor, family 10, subfamily A, member 5
16572	NUP210L	-0.7262	0.0071	nucleoporin 210kDa-like
16573	NR2C2AP	-0.7262	0.0713	nuclear receptor 2C2-associated protein
16574	HRASLS5	-0.7262	0.051	HRAS-like suppressor family, member 5
16575	COL20A1	-0.7262	0.1988	collagen, type XX, alpha 1
16576	NUBP2	-0.7267	0.0966	nucleotide binding protein 2
16577	KATNA1	-0.7267	0.0999	katanin p60 (ATPase containing) subunit A 1
16578	FANCI	-0.7267	0.1361	Fanconi anemia, complementation group I
16579	ATAD2	-0.7267	0.1745	ATPase family, AAA domain containing 2
16580	TONSL	-0.7275	0.1551	tonsoku-like, DNA repair protein
16581	SNRPD2	-0.7275	0.1282	small nuclear ribonucleoprotein D2 polypeptide 16.5kDa
16582	MAB21L3	-0.7275	0.0038	mab-21-like 3 (C. elegans)
16583	LANCL2	-0.7275	0.0132	LanC lantibiotic synthetase component C-like 2 (bacterial)
16584	CTXN1	-0.7275	0.0962	cortexin 1
16585	CAMSAP1	-0.7275	0.0253	calmodulin regulated spectrin-associated protein 1
16586	PSMD8	-0.7283	0.1301	proteasome (prosome, macropain) 26S subunit, non-ATPase, 8
16587	DZANK1-AS1	-0.7286	0.2333	DZANK1 antisense RNA 1 (non-protein coding)
16588	PPARGC1B	-0.7287	0.02	peroxisome proliferator-activated receptor gamma, coactivator 1 beta
16589	NACC1	-0.7287	0.0591	nucleus accumbens associated 1, BEN and BTB (POZ) domain containing
16590	FAM181B	-0.7287	0.063	family with sequence similarity 181, member B
16591	TTLL3	-0.73	0.0891	tubulin tyrosine ligase-like family, member 3
16592	TDRG1	-0.73	0.1702	testis development related 1 (non-protein coding)
16593	SYNGAP1	-0.73	0.0961	synaptic Ras GTPase activating protein 1
16594	SLC22A16	-0.73	0.0131	solute carrier family 22 (organic cation/carnitine transporter), member 16
16595	HPS4	-0.73	0.0277	Hermansky-Pudlak syndrome 4
16596	GRIN2D	-0.73	0.1479	glutamate receptor, ionotropic, N-methyl D-aspartate 2D
16597	FAM181A-AS1	-0.73	0.9996	FAM181A antisense RNA 1 (non-protein coding)
16598	ACPT	-0.73	0.1994	acid phosphatase, testicular
16599	USP6	-0.7308	0.0418	ubiquitin specific peptidase 6 (Tre-2 oncogene)
16600	NOC2L	-0.7308	0.1091	nucleolar complex associated 2 homolog (S. cerevisiae)
16601	MRPL39	-0.7308	0.1491	mitochondrial ribosomal protein L39
16602	PITPNM2	-0.7312	0.0222	phosphatidylinositol transfer protein, membrane-associated 2
16603	CXXC11	-0.7312	0.1299	CXXC finger protein 11
16604	CENPH	-0.7312	0.1203	centromere protein H
16605	LOC100289361	-0.7314	0.0169	uncharacterized LOC100289361
16606	FAM170B	-0.7314	0.0694	family with sequence similarity 170, member B
16607	LRP2BP	-0.7317	0.0092	LRP2 binding protein
16608	INTS4L1	-0.7317	0.9996	integrator complex subunit 4-like 1
16609	ZNRF1	-0.7325	0.0096	zinc and ring finger 1, E3 ubiquitin protein ligase
16610	ZNF526	-0.7325	0.0268	zinc finger protein 526
16611	TTC9C	-0.7325	0.0182	tetratricopeptide repeat domain 9C
16612	OSBPL11	-0.7325	0.135	oxysterol binding protein-like 11
16613	MTX2	-0.7325	0.1507	metaxin 2
16614	MCM2	-0.7325	0.1377	minichromosome maintenance complex component 2
16615	GLRX2	-0.7325	0.1006	glutaredoxin 2
16616	DGKZ	-0.7325	0.056	diacylglycerol kinase, zeta
16617	CDC123	-0.7325	0.1924	cell division cycle 123 homolog (S. cerevisiae)
16618	CCDC171	-0.7325	0.007	coiled-coil domain containing 171
16619	CATSPER3	-0.7325	0.0059	cation channel, sperm associated 3
16620	RASSF2	-0.7333	0.0362	Ras association (RalGDS/AF-6) domain family member 2
16621	MTA1	-0.7333	0.074	metastasis associated 1
16622	CKS2	-0.7333	0.1116	CDC28 protein kinase regulatory subunit 2
16623	ZSCAN12P1	-0.7337	0.0419	zinc finger and SCAN domain containing 12 pseudogene 1
16624	SIRPD	-0.7337	0.0601	signal-regulatory protein delta
16625	CC2D2B	-0.7337	0.052	coiled-coil and C2 domain containing 2B
16626	TBC1D10B	-0.7342	0.0567	TBC1 domain family, member 10B
16627	POLE2	-0.7342	0.1287	polymerase (DNA directed), epsilon 2, accessory subunit
16628	DNAJB13	-0.7343	0.1094	DnaJ (Hsp40) homolog, subfamily B, member 13
16629	ARRDC3-AS1	-0.7343	0.9999	ARRDC3 antisense RNA 1 (non-protein coding)
16630	RAP1GDS1	-0.735	0.0785	RAP1, GTP-GDP dissociation stimulator 1

16631	NODAL	-0.735	0.0948	nodal homolog (mouse)
16632	LOC338651	-0.735	0.0113	uncharacterized LOC338651
16633	LOC257358	-0.735	0.1032	uncharacterized LOC257358
16634	CXXC1	-0.735	0.0762	CXXC finger protein 1
16635	CCDC124	-0.735	0.1185	coiled-coil domain containing 124
16636	CAMKV	-0.735	0.123	CaM kinase-like vesicle-associated
16637	C1orf228	-0.7357	0.0212	chromosome 1 open reading frame 228
16638	WBP11	-0.7358	0.081	WW domain binding protein 11
16639	VWA5B1	-0.7362	0.1227	von Willebrand factor A domain containing 5B1
16640	PP12613	-0.7362	0.0242	uncharacterized LOC100192379
16641	FBN3	-0.7362	0.1418	fibrillin 3
16642	DAND5	-0.7362	0.0373	DAN domain family, member 5
16643	RAB33A	-0.7367	0.0103	RAB33A, member RAS oncogene family
16644	OLA1	-0.7367	0.0405	Obg-like ATPase 1
16645	EIF2B5	-0.7367	0.0387	eukaryotic translation initiation factor 2B, subunit 5 epsilon, 82kDa
16646	LOC100288079	-0.7371	0.9999	microtubule-associated protein 1 light chain 3 beta pseudogene
16647	USP26	-0.7375	0.0067	ubiquitin specific peptidase 26
16648	SPAG5	-0.7375	0.0971	sperm associated antigen 5
16649	RNF133	-0.7375	0.0157	ring finger protein 133
16650	DKFZP434K028	-0.7375	0.1379	uncharacterized LOC26070
16651	BRSK1	-0.7375	0.0544	BR serine/threonine kinase 1
16652	SSX2IP	-0.7383	0.0464	synovial sarcoma, X breakpoint 2 interacting protein
16653	PAFAH1B3	-0.7383	0.098	platelet-activating factor acetylhydrolase 1b, catalytic subunit 3 (29kDa)
16654	CPSF4	-0.7383	0.05	cleavage and polyadenylation specific factor 4, 30kDa
16655	CDC25C	-0.7383	0.0739	cell division cycle 25 homolog C (S. pombe)
16656	FER1L5	-0.7387	0.0266	fer-1-like 5 (C. elegans)
16657	CLRN1	-0.7387	0.1558	clarin 1
16658	SBF1	-0.7392	0.0842	SET binding factor 1
16659	RNASEH2A	-0.7392	0.147	ribonuclease H2, subunit A
16660	PHF7	-0.7392	0.0081	PHD finger protein 7
16661	ZNF554	-0.74	0.0369	zinc finger protein 554
16662	TACC3	-0.74	0.1085	transforming, acidic coiled-coil containing protein 3
16663	NPSR1-AS1	-0.74	0.1364	NPSR1 antisense RNA 1 (non-protein coding)
16664	LOC100129195	-0.74	0.9999	uncharacterized LOC100129195
16665	LMX1A	-0.74	0.1412	LIM homeobox transcription factor 1, alpha
16666	ELOVL5	-0.74	0.0289	ELOVL fatty acid elongase 5
16667	SYNGR3	-0.7408	0.0088	synaptogyrin 3
16668	FBXO5	-0.7408	0.1819	F-box protein 5
16669	SPATA25	-0.7412	0.041	spermatogenesis associated 25
16670	RNASE11	-0.7412	0.0046	ribonuclease, RNase A family, 11 (non-active)
16671	MFSD9	-0.7412	0.0085	major facilitator superfamily domain containing 9
16672	KIAA2022	-0.7412	0.0134	KIAA2022
16673	MLC1	-0.7417	0.0047	megalencephalic leukoencephalopathy with subcortical cysts 1
16674	SRRT	-0.7425	0.1047	serrate RNA effector molecule homolog (Arabidopsis)
16675	SLC1A4	-0.7425	0.0042	solute carrier family 1 (glutamate/neutral amino acid transporter), member 4
16676	SET	-0.7425	0.1677	SET nuclear oncogene
16677	NTMT1	-0.7425	0.1037	N-terminal Xaa-Pro-Lys N-methyltransferase 1
16678	NOL7	-0.7425	0.1367	nucleolar protein 7, 27kDa
16679	LRRC33	-0.7425	0.023	leucine rich repeat containing 33
16680	FAM123C	-0.7425	0.2301	family with sequence similarity 123C
16681	AGBL3	-0.7425	0.0051	ATP/GTP binding protein-like 3
16682	GRK6	-0.7433	0.082	G protein-coupled receptor kinase 6
16683	DNM1L	-0.7433	0.147	dynamin 1-like
16684	CHST13	-0.7437	0.0251	carbohydrate (chondroitin 4) sulfotransferase 13
16685	WRAP53	-0.7442	0.0982	WD repeat containing, antisense to TP53
16686	TKTL1	-0.7442	0.0205	transketolase-like 1
16687	PEX14	-0.7442	0.012	peroxisomal biogenesis factor 14
16688	MRPL4	-0.7442	0.1439	mitochondrial ribosomal protein L4
16689	KCNN2	-0.7442	0.0037	potassium intermediate/small conductance calcium-activated channel, subfamily N, member 2
16690	ADA	-0.7442	0.0124	adenosine deaminase
16691	RPF2	-0.745	0.1084	ribosome production factor 2 homolog (S. cerevisiae)
16692	PRMT1	-0.745	0.1393	protein arginine methyltransferase 1
16693	LINC00461	-0.745	0.0089	long intergenic non-protein coding RNA 461
16694	CCDC99	-0.745	0.1425	coiled-coil domain containing 99
16695	C14orf102	-0.745	0.0079	chromosome 14 open reading frame 102
16696	KLC2	-0.7458	0.035	kinesin light chain 2
16697	CEP250	-0.7458	0.0565	centrosomal protein 250kDa
16698	C1orf112	-0.7458	0.0982	chromosome 1 open reading frame 112
16699	PQLC2	-0.7462	0.0265	PQ loop repeat containing 2
16700	MYO1G	-0.7462	0.0487	myosin IG
16701	mars-02	-0.7462	0.0478	methionyl-tRNA synthetase 2, mitochondrial
16702	LY6G5B	-0.7462	0.0127	lymphocyte antigen 6 complex, locus G5B
16703	E2F7	-0.7462	0.0478	E2F transcription factor 7
16704	TSSC4	-0.7467	0.1017	tumor suppressing subtransferable candidate 4
16705	NUDT3	-0.7471	0.0107	nudix (nucleoside diphosphate linked moiety X)-type motif 3
16706	WIZ	-0.7475	0.0945	widely interspaced zinc finger motifs
16707	TDP1	-0.7475	0.0395	tyrosyl-DNA phosphodiesterase 1
16708	SLA2	-0.7475	0.0523	Src-like-adaptor 2
16709	PPP1R3E	-0.7475	0.0398	protein phosphatase 1, regulatory subunit 3E
16710	AMH	-0.7475	0.0586	anti-Mullerian hormone
16711	PHKG2	-0.7483	0.0452	phosphorylase kinase, gamma 2 (testis)
16712	C17orf53	-0.7483	0.1042	chromosome 17 open reading frame 53
16713	C22orf42	-0.7486	0.0216	chromosome 22 open reading frame 42
16714	THUMPD3	-0.7487	0.1166	THUMP domain containing 3

16715	SNHG1	-0.7487	0.0886	small nucleolar RNA host gene 1 (non-protein coding)
16716	SAMD10	-0.7487	0.1043	sterile alpha motif domain containing 10
16717	C7orf45	-0.7487	0.0158	chromosome 7 open reading frame 45
16718	MEAF6	-0.7492	0.0258	MYST/Esa1-associated factor 6
16719	ZGLP1	-0.75	0.9999	zinc finger, GATA-like protein 1
16720	XPO1	-0.75	0.2448	exportin 1 (CRM1 homolog, yeast)
16721	PAQR8	-0.75	0.0114	progestin and adipoQ receptor family member VIII
16722	MTFP1	-0.75	0.0846	mitochondrial fission process 1
16723	C3orf20	-0.75	0.0545	chromosome 3 open reading frame 20
16724	GRIA2	-0.7508	0.0089	glutamate receptor, ionotropic, AMPA 2
16725	ELAVL4	-0.7508	0.0085	ELAV (embryonic lethal, abnormal vision, Drosophila)-like 4
16726	CADPS	-0.7508	0.0069	Ca++-dependent secretion activator
16727	PACSIN1	-0.7513	0.0413	protein kinase C and casein kinase substrate in neurons 1
16728	GPR84	-0.7513	0.0471	G protein-coupled receptor 84
16729	RFC2	-0.7517	0.1421	replication factor C (activator 1) 2, 40kDa
16730	CDK9	-0.7517	0.0138	cyclin-dependent kinase 9
16731	WRB	-0.7525	0.1291	tryptophan rich basic protein
16732	MTMR14	-0.7525	0.0279	myotubularin related protein 14
16733	EHMT2	-0.7525	0.085	euchromatic histone-lysine N-methyltransferase 2
16734	DOC2A	-0.7525	0.0282	double C2-like domains, alpha
16735	DNMT3B	-0.7525	0.0254	DNA (cytosine-5-)-methyltransferase 3 beta
16736	C17orf100	-0.7525	0.0069	chromosome 17 open reading frame 100
16737	ANLN	-0.7525	0.0873	anillin, actin binding protein
16738	RSPO4	-0.7529	0.15	R-spondin 4
16739	LOC100288432	-0.7529	0.027	uncharacterized LOC100288432
16740	C1orf200	-0.7529	0.2968	chromosome 1 open reading frame 200
16741	EVL	-0.7533	0.0462	Enah/Vasp-like
16742	PARVG	-0.7538	0.062	parvin, gamma
16743	FBLL1	-0.7538	0.0138	fibrillarin-like 1
16744	PHACTR1	-0.7542	0.0063	phosphatase and actin regulator 1
16745	DHX16	-0.7542	0.0371	DEAH (Asp-Glu-Ala-His) box polypeptide 16
16746	LGALS8-AS1	-0.7543	0.1443	LGALS8 antisense RNA 1 (non-protein coding)
16747	ZNF681	-0.755	0.0249	zinc finger protein 681
16748	ZFP41	-0.755	0.0111	zinc finger protein 41 homolog (mouse)
16749	R3HDML	-0.755	0.036	R3H domain containing-like
16750	MTF2	-0.755	0.1727	metal response element binding transcription factor 2
16751	MCM3	-0.755	0.1407	minichromosome maintenance complex component 3
16752	HIST1H4C	-0.755	0.0387	histone cluster 1, H4c
16753	DNAJC18	-0.755	0.0124	DnaJ (Hsp40) homolog, subfamily C, member 18
16754	CAMK4	-0.7558	0.0108	calcium/calmodulin-dependent protein kinase IV
16755	ZNF708	-0.7563	0.0613	zinc finger protein 708
16756	TRIM39	-0.7563	0.0176	tripartite motif containing 39
16757	TCP10L	-0.7563	0.008	t-complex 10 (mouse)-like
16758	NLRP14	-0.7563	0.006	NLR family, pyrin domain containing 14
16759	C20orf144	-0.7563	0.0851	chromosome 20 open reading frame 144
16760	C12orf73	-0.7563	0.053	chromosome 12 open reading frame 73
16761	WDR82	-0.7567	0.0339	WD repeat domain 82
16762	VPS33A	-0.7567	0.0086	vacuolar protein sorting 33 homolog A (S. cerevisiae)
16763	SMG9	-0.7567	0.0313	smg-9 homolog, nonsense mediated mRNA decay factor (C. elegans)
16764	ORC1	-0.7567	0.0863	origin recognition complex, subunit 1
16765	COPE	-0.7567	0.1146	coatomer protein complex, subunit epsilon
16766	CLDND1	-0.7567	0.0928	claudin domain containing 1
16767	BSN	-0.7567	0.055	bassoon (presynaptic cytomatrix protein)
16768	LOC340074	-0.7571	0.9996	uncharacterized LOC340074
16769	RGAG1	-0.7575	0.0177	retrotransposon gag domain containing 1
16770	RASGRP2	-0.7575	0.0416	RAS guanyl releasing protein 2 (calcium and DAG-regulated)
16771	DDX24	-0.7575	0.0143	DEAD (Asp-Glu-Ala-Asp) box polypeptide 24
16772	CCDC11	-0.7575	0.0123	coiled-coil domain containing 11
16773	C20orf197	-0.7575	0.0054	chromosome 20 open reading frame 197
16774	UTS2D	-0.7588	0.003	urotensin 2 domain containing
16775	RFX4	-0.7588	0.0126	regulatory factor X, 4 (influences HLA class II expression)
16776	DPPA5	-0.7588	0.0261	developmental pluripotency associated 5
16777	CCDC23	-0.7588	0.0087	coiled-coil domain containing 23
16778	C1orf111	-0.7588	0.0867	chromosome 1 open reading frame 111
16779	RFPL3-AS1	-0.7592	0.0167	RFPL3 antisense RNA 1 (non-protein coding)
16780	RABIF	-0.7592	0.0175	RAB interacting factor
16781	COX6C	-0.7592	0.0457	cytochrome c oxidase subunit VIc
16782	TAF4	-0.76	0.0345	TAF4 RNA polymerase II, TATA box binding protein (TBP)-associated factor, 135kDa
16783	MGRN1	-0.7608	0.0434	mahogunin ring finger 1, E3 ubiquitin protein ligase
16784	CDK1	-0.7608	0.1317	cyclin-dependent kinase 1
16785	SNHG10	-0.7613	0.0271	small nucleolar RNA host gene 10 (non-protein coding)
16786	LDHAL6A	-0.7613	0.0132	lactate dehydrogenase A-like 6A
16787	CLEC4C	-0.7613	0.0053	C-type lectin domain family 4, member C
16788	CALR3	-0.7613	0.0061	calreticulin 3
16789	PAK1IP1	-0.7617	0.1352	PAK1 interacting protein 1
16790	PAICS	-0.7617	0.1894	phosphoribosylaminoimidazole carboxylase, phosphoribosylaminoimidazole succinocarboxamide synthetase
16791	EXOSC10	-0.7617	0.0795	exosome component 10
16792	PIK3R6	-0.7625	0.0264	phosphoinositide-3-kinase, regulatory subunit 6
16793	LSM6	-0.7625	0.108	LSM6 homolog, U6 small nuclear RNA associated (S. cerevisiae)
16794	LCN15	-0.7625	0.0194	lipocalin 15
16795	WDR18	-0.7633	0.1078	WD repeat domain 18
16796	RBMX	-0.7633	0.0635	RNA binding motif protein, X-linked
16797	MED16	-0.7633	0.0733	mediator complex subunit 16
16798	EPS15	-0.7633	0.1709	epidermal growth factor receptor pathway substrate 15

16799	AP1S2	-0.7633	0.0332	adaptor-related protein complex 1, sigma 2 subunit
16800	TMEM132D	-0.7638	0.0179	transmembrane protein 132D
16801	NKIRAS1	-0.7638	0.0233	NFKB inhibitor interacting Ras-like 1
16802	MSI2	-0.7638	0.0044	musashi homolog 2 (Drosophila)
16803	LSM11	-0.7638	0.0061	LSM11, U7 small nuclear RNA associated
16804	LINGO1	-0.7638	0.0124	leucine rich repeat and Ig domain containing 1
16805	C10orf25	-0.7638	0.0053	chromosome 10 open reading frame 25
16806	NUP153	-0.7642	0.1808	nucleoporin 153kDa
16807	LOC285847	-0.7643	0.9996	uncharacterized LOC285847
16808	ZP1	-0.765	0.011	zona pellucida glycoprotein 1 (sperm receptor)
16809	TXNL4A	-0.765	0.0636	thioredoxin-like 4A
16810	TMED8	-0.765	0.0058	transmembrane emp24 protein transport domain containing 8
16811	SLC35F1	-0.765	0.024	solute carrier family 35, member F1
16812	PSMD7	-0.765	0.1083	proteasome (prosome, macropain) 26S subunit, non-ATPase, 7
16813	MBNL3	-0.765	0.0064	muscleblind-like splicing regulator 3
16814	DRG1	-0.765	0.1055	developmentally regulated GTP binding protein 1
16815	C4orf26	-0.765	0.0062	chromosome 4 open reading frame 26
16816	POP1	-0.7657	0.045	processing of precursor 1, ribonuclease P/MRP subunit (S. cerevisiae)
16817	LOC149086	-0.7657	0.9999	ornithine decarboxylase 1 pseudogene
16818	UBAC1	-0.7658	0.0518	UBA domain containing 1
16819	RPL32P3	-0.7663	0.0236	ribosomal protein L32 pseudogene 3
16820	PASD1	-0.7663	0.0123	PAS domain containing 1
16821	TRAF3	-0.7667	0.0089	TNF receptor-associated factor 3
16822	RPA2	-0.7667	0.0861	replication protein A2, 32kDa
16823	NAT10	-0.7667	0.0893	N-acetyltransferase 10 (GCN5-related)
16824	ZBTB8B	-0.7671	0.0497	zinc finger and BTB domain containing 8B
16825	LINC00293	-0.7671	0.9996	long intergenic non-protein coding RNA 293
16826	NAPB	-0.7675	0.0178	N-ethylmaleimide-sensitive factor attachment protein, beta
16827	CNTD1	-0.7675	0.0197	cyclin N-terminal domain containing 1
16828	CENPL	-0.7675	0.0866	centromere protein L
16829	C22orf46	-0.7675	0.0089	chromosome 22 open reading frame 46
16830	ABCF1	-0.7675	0.0621	ATP-binding cassette, sub-family F (GCN20), member 1
16831	SRRD	-0.7683	0.0232	SRR1 domain containing
16832	SEH1L	-0.7683	0.1929	SEH1-like (S. cerevisiae)
16833	PRMT7	-0.7683	0.0507	protein arginine methyltransferase 7
16834	CLASP2	-0.7683	0.1518	cytoplasmic linker associated protein 2
16835	LOC221122	-0.7686	0.9996	uncharacterized LOC221122
16836	C17orf104	-0.7686	0.0033	chromosome 17 open reading frame 104
16837	TAF1L	-0.7688	0.0349	TAF1 RNA polymerase II, TATA box binding protein (TBP)-associated factor, 210kDa-like
16838	SGK196	-0.7688	0.0112	protein kinase-like protein SgK196
16839	DEFB123	-0.7688	0.0115	defensin, beta 123
16840	ADAMTS20	-0.7688	0.0117	ADAM metallopeptidase with thrombospondin type 1 motif, 20
16841	CEP76	-0.7692	0.1419	centrosomal protein 76kDa
16842	CBX3	-0.7692	0.208	chromobox homolog 3
16843	TUBB2B	-0.77	0.0074	tubulin, beta 2B class IIb
16844	TJAP1	-0.77	0.0313	tight junction associated protein 1 (peripheral)
16845	NETO1	-0.77	0.0037	neuropilin (NRP) and tolloid (TLL)-like 1
16846	LOC339352	-0.77	0.0424	cytosolic thiouridylase subunit 1 homolog (S. pombe) pseudogene
16847	ERH	-0.77	0.0943	enhancer of rudimentary homolog (Drosophila)
16848	TSR1	-0.7708	0.1209	TSR1, 20S rRNA accumulation, homolog (S. cerevisiae)
16849	RAE1	-0.7708	0.0574	RAE1 RNA export 1 homolog (S. pombe)
16850	PGBD5	-0.7708	0.0046	piggyBac transposable element derived 5
16851	LOC81691	-0.7708	0.0235	exonuclease NEF-sp
16852	KCNK12	-0.7708	0.0034	potassium channel, subfamily K, member 12
16853	RGS9BP	-0.7713	0.0042	regulator of G protein signaling 9 binding protein
16854	PREX1	-0.7713	0.0196	phosphatidylinositol-3,4,5-trisphosphate-dependent Rac exchange factor 1
16855	FRMPD3	-0.7713	0.0674	FERM and PDZ domain containing 3
16856	C18orf54	-0.7713	0.0469	chromosome 18 open reading frame 54
16857	LINC00486	-0.7714	0.9996	long intergenic non-protein coding RNA 486
16858	MRPS28	-0.7717	0.1492	mitochondrial ribosomal protein S28
16859	C1orf114	-0.7717	0.0052	chromosome 1 open reading frame 114
16860	WFDC11	-0.7725	0.0071	WAP four-disulfide core domain 11
16861	U2AF1	-0.7725	0.0503	U2 small nuclear RNA auxiliary factor 1
16862	TMEM198	-0.7725	0.1098	transmembrane protein 198
16863	PLK4	-0.7725	0.1008	polo-like kinase 4
16864	OXNAD1	-0.7725	0.0221	oxidoreductase NAD-binding domain containing 1
16865	IL27	-0.7725	0.0629	interleukin 27
16866	GNG4	-0.7725	0.0262	guanine nucleotide binding protein (G protein), gamma 4
16867	VPRBP	-0.7733	0.022	Vpr (HIV-1) binding protein
16868	SLC4A8	-0.7733	0.0053	solute carrier family 4, sodium bicarbonate cotransporter, member 8
16869	POLE	-0.7733	0.0676	polymerase (DNA directed), epsilon, catalytic subunit
16870	ZNF843	-0.7738	0.2236	zinc finger protein 843
16871	CCNB3	-0.7738	0.0088	cyclin B3
16872	C2orf65	-0.7738	0.0042	chromosome 2 open reading frame 65
16873	TTC1	-0.7742	0.0297	tetratricopeptide repeat domain 1
16874	KIF1A	-0.7742	0.0513	kinesin family member 1A
16875	LNP1	-0.7743	0.0152	leukemia NUP98 fusion partner 1
16876	FAM18A	-0.7743	0.0096	family with sequence similarity 18, member A
16877	TIAM1	-0.775	0.0044	T-cell lymphoma invasion and metastasis 1
16878	GRID1	-0.775	0.0805	glutamate receptor, ionotropic, delta 1
16879	C21orf128	-0.7757	0.0385	chromosome 21 open reading frame 128
16880	SRP19	-0.7758	0.0585	signal recognition particle 19kDa
16881	LCMT1	-0.7758	0.0415	leucine carboxyl methyltransferase 1
16882	GIT1	-0.7758	0.0304	G protein-coupled receptor kinase interacting ArfGAP 1

16883	YPEL4	-0.7763	0.016	yippee-like 4 (Drosophila)
16884	OR6W1P	-0.7763	0.0567	olfactory receptor, family 6, subfamily W, member 1 pseudogene
16885	LINC00470	-0.7763	0.004	long intergenic non-protein coding RNA 470
16886	DEFB119	-0.7763	0.0986	defensin, beta 119
16887	APOBEC3D	-0.7763	0.0057	apolipoprotein B mRNA editing enzyme, catalytic polypeptide-like 3D
16888	LOC646214	-0.7771	0.0189	p21 protein (Cdc42/Rac)-activated kinase 2 pseudogene
16889	PSMB2	-0.7783	0.0988	proteasome (prosome, macropain) subunit, beta type, 2
16890	KIFC1	-0.7783	0.0808	kinesin family member C1
16891	CPOX	-0.7783	0.0453	coproporphyrinogen oxidase
16892	ZNF295	-0.7788	0.0611	zinc finger protein 295
16893	TIMM50	-0.7788	0.0982	translocase of inner mitochondrial membrane 50 homolog (S. cerevisiae)
16894	FAM154A	-0.7788	0.0262	family with sequence similarity 154, member A
16895	EPHA6	-0.7788	0.0056	EPH receptor A6
16896	PFDN4	-0.7792	0.0455	prefoldin subunit 4
16897	CACNA1B	-0.7792	0.0988	calcium channel, voltage-dependent, N type, alpha 1B subunit
16898	C20orf27	-0.7792	0.0879	chromosome 20 open reading frame 27
16899	TMEM70	-0.78	0.049	transmembrane protein 70
16900	RYBP	-0.78	0.0364	RING1 and YY1 binding protein
16901	LOC286467	-0.78	0.0107	family with sequence similarity 195, member A pseudogene
16902	GZF1	-0.78	0.0084	GDNF-inducible zinc finger protein 1
16903	GRPEL2	-0.78	0.031	GrpE-like 2, mitochondrial (E. coli)
16904	FAM210A	-0.78	0.0486	family with sequence similarity 210, member A
16905	EFCAB5	-0.78	0.0735	EF-hand calcium binding domain 5
16906	DGUOK	-0.78	0.0606	deoxyguanosine kinase
16907	STAG3	-0.7808	0.0051	stromal antigen 3
16908	ATIC	-0.7808	0.1271	5-aminoimidazole-4-carboxamide ribonucleotide formyltransferase/IMP cyclohydrolase
16909	TREML1	-0.7813	0.0402	triggering receptor expressed on myeloid cells-like 1
16910	SPSB4	-0.7813	0.0645	splA/ryanodine receptor domain and SOCS box containing 4
16911	MTHFD1L	-0.7813	0.0241	methylenetetrahydrofolate dehydrogenase (NADP+ dependent) 1-like
16912	ARHGAP9	-0.7813	0.0597	Rho GTPase activating protein 9
16913	SMG8	-0.7817	0.0274	smg-8 homolog, nonsense mediated mRNA decay factor (C. elegans)
16914	KHDRBS3	-0.7817	0.0053	KH domain containing, RNA binding, signal transduction associated 3
16915	ZC3HC1	-0.7825	0.0356	zinc finger, C3HC-type containing 1
16916	TRA2B	-0.7825	0.152	transformer 2 beta homolog (Drosophila)
16917	TBCB	-0.7825	0.0631	tubulin folding cofactor B
16918	MKI67IP	-0.7825	0.1208	MKI67 (FHA domain) interacting nucleolar phosphoprotein
16919	BID	-0.7825	0.0213	BH3 interacting domain death agonist
16920	AMPD2	-0.7825	0.0184	adenosine monophosphate deaminase 2
16921	C12orf77	-0.7829	0.9996	chromosome 12 open reading frame 77
16922	PPFIA2	-0.7833	0.0261	protein tyrosine phosphatase, receptor type, f polypeptide (PTPRF), interacting protein (liprin), alpha 2
16923	FIP1L1	-0.7842	0.0528	FIP1 like 1 (S. cerevisiae)
16924	ZNF584	-0.785	0.0068	zinc finger protein 584
16925	ZBTB49	-0.785	0.0077	zinc finger and BTB domain containing 49
16926	VARS	-0.785	0.0961	valyl-tRNA synthetase
16927	GAB2	-0.785	0.0173	GRB2-associated binding protein 2
16928	DPYSL4	-0.785	0.0094	dihydropyrimidinase-like 4
16929	CHD1L	-0.785	0.0108	chromodomain helicase DNA binding protein 1-like
16930	C12orf42	-0.785	0.0037	chromosome 12 open reading frame 42
16931	C1orf227	-0.7857	0.0056	chromosome 1 open reading frame 227
16932	CACTIN	-0.7858	0.0829	cactin, spliceosome C complex subunit
16933	FAM75D1	-0.7871	0.24	family with sequence similarity 75, member D1
16934	TUBA3FP	-0.7875	0.0077	tubulin, alpha 3f, pseudogene
16935	TRMT6	-0.7875	0.057	tRNA methyltransferase 6 homolog (S. cerevisiae)
16936	SNAP91	-0.7875	0.0127	synaptosomal-associated protein, 91kDa homolog (mouse)
16937	LINC00158	-0.7875	0.004	long intergenic non-protein coding RNA 158
16938	GNG7	-0.7875	0.0162	guanine nucleotide binding protein (G protein), gamma 7
16939	DIAPH3	-0.7875	0.0485	diaphanous homolog 3 (Drosophila)
16940	AKAP8L	-0.7875	0.0406	A kinase (PRKA) anchor protein 8-like
16941	USP22	-0.7883	0.0459	ubiquitin specific peptidase 22
16942	POU3F2	-0.7883	0.0158	POU class 3 homeobox 2
16943	ACTL6B	-0.7883	0.1624	actin-like 6B
16944	ZNF707	-0.7887	0.0211	zinc finger protein 707
16945	WDR83	-0.7887	0.0279	WD repeat domain 83
16946	LYSMD2	-0.7887	0.0185	LysM, putative peptidoglycan-binding, domain containing 2
16947	KLHL17	-0.7887	0.0561	kelch-like 17 (Drosophila)
16948	ITPRIPL1	-0.7887	0.0087	inositol 1,4,5-trisphosphate receptor interacting protein-like 1
16949	CDH23	-0.7887	0.0608	cadherin-related 23
16950	ADAD2	-0.7887	0.1339	adenosine deaminase domain containing 2
16951	MYBL2	-0.7892	0.0891	v-myb myeloblastosis viral oncogene homolog (avian)-like 2
16952	GTPBP4	-0.7892	0.1551	GTP binding protein 4
16953	STIL	-0.79	0.0816	SCL/TAL1 interrupting locus
16954	KIAA1257	-0.79	0.0041	KIAA1257
16955	INPP4A	-0.79	0.0123	inositol polyphosphate-4-phosphatase, type I, 107kDa
16956	SNN	-0.7908	0.0072	stannin
16957	NAP1L4	-0.7908	0.0326	nucleosome assembly protein 1-like 4
16958	CFDP1	-0.7908	0.0415	craniofacial development protein 1
16959	FAM219A	-0.7912	0.0183	family with sequence similarity 219, member A
16960	CENPK	-0.7912	0.1081	centromere protein K
16961	IFFO1	-0.7917	0.0461	intermediate filament family orphan 1
16962	SLC39A12	-0.7925	0.0044	solute carrier family 39 (zinc transporter), member 12
16963	GPRIN1	-0.7925	0.0816	G protein regulated inducer of neurite outgrowth 1
16964	ELP3	-0.7925	0.0101	elongation protein 3 homolog (S. cerevisiae)
16965	CCT6A	-0.7933	0.1961	chaperonin containing TCP1, subunit 6A (zeta 1)
16966	LOC440356	-0.7937	0.0251	uncharacterized LOC440356

16967	DIRAS1	-0.7937	0.0218	DIRAS family, GTP-binding RAS-like 1
16968	RBM38	-0.7942	0.0303	RNA binding motif protein 38
16969	FKBP3	-0.7942	0.1279	FK506 binding protein 3, 25kDa
16970	LOC254312	-0.7943	0.9996	uncharacterized LOC254312
16971	STK40	-0.795	0.0147	serine/threonine kinase 40
16972	OXER1	-0.795	0.1299	oxoeicosanoid (OXE) receptor 1
16973	FAM135B	-0.795	0.0476	family with sequence similarity 135, member B
16974	TXNL4B	-0.7958	0.0069	thioredoxin-like 4B
16975	SPPL2C	-0.7962	0.0957	signal peptide peptidase like 2C
16976	SALL3	-0.7962	0.0178	sal-like 3 (Drosophila)
16977	RHBDL3	-0.7962	0.0388	rhomboid, veinlet-like 3 (Drosophila)
16978	GPR61	-0.7962	0.015	G protein-coupled receptor 61
16979	ATPAF1	-0.7962	0.0574	ATP synthase mitochondrial F1 complex assembly factor 1
16980	LIG1	-0.7967	0.0722	ligase I, DNA, ATP-dependent
16981	HSP90AA1	-0.7967	0.0758	heat shock protein 90kDa alpha (cytosolic), class A member 1
16982	FDFT1	-0.7967	0.0109	farnesyl-diphosphate farnesyltransferase 1
16983	SPZ1	-0.7975	0.0033	spermatogenic leucine zipper 1
16984	RAB39A	-0.7975	0.011	RAB39A, member RAS oncogene family
16985	ABT1	-0.7983	0.0206	activator of basal transcription 1
16986	LOC143188	-0.7986	0.0968	uncharacterized LOC143188
16987	TNFRSF13C	-0.7987	0.0599	tumor necrosis factor receptor superfamily, member 13C
16988	DDHD1	-0.7987	0.0157	DDHD domain containing 1
16989	C16orf55	-0.7987	0.0123	chromosome 16 open reading frame 55
16990	RNASEH1	-0.8	0.0419	ribonuclease H1
16991	POLR2I	-0.8	0.0965	polymerase (RNA) II (DNA directed) polypeptide I, 14.5kDa
16992	PANK4	-0.8	0.0201	pantothenate kinase 4
16993	NDUFV3	-0.8	0.0188	NADH dehydrogenase (ubiquinone) flavoprotein 3, 10kDa
16994	LHFPL4	-0.8	0.0522	lipoma HMGIC fusion partner-like 4
16995	C17orf50	-0.8	0.0601	chromosome 17 open reading frame 50
16996	ASCL1	-0.8	0.0361	achaete-scute complex homolog 1 (Drosophila)
16997	AP3M2	-0.8	0.0101	adaptor-related protein complex 3, mu 2 subunit
16998	UTP11L	-0.8008	0.115	UTP11-like, U3 small nucleolar ribonucleoprotein, (yeast)
16999	MKRN2	-0.8008	0.0069	makorin ring finger protein 2
17000	RCOR2	-0.8012	0.0496	REST corepressor 2
17001	MRPS9	-0.8012	0.0717	mitochondrial ribosomal protein S9
17002	C12orf53	-0.8012	0.099	chromosome 12 open reading frame 53
17003	LOC284688	-0.8014	0.9996	uncharacterized LOC284688
17004	TRIM24	-0.8017	0.0232	tripartite motif containing 24
17005	PI4KA	-0.8017	0.0223	phosphatidylinositol 4-kinase, catalytic, alpha
17006	HSF2	-0.8017	0.0832	heat shock transcription factor 2
17007	GTPBP1	-0.8017	0.0356	GTP binding protein 1
17008	EXOSC5	-0.8017	0.0725	exosome component 5
17009	RANBP1	-0.8018	0.0819	RAN binding protein 1
17010	UHRF1BP1	-0.8025	0.0055	UHRF1 binding protein 1
17011	KCNH7	-0.8025	0.0175	potassium voltage-gated channel, subfamily H (eag-related), member 7
17012	CIRBP-AS1	-0.8025	0.1098	CIRBP antisense RNA 1 (non-protein coding)
17013	C17orf81	-0.8025	0.0205	chromosome 17 open reading frame 81
17014	LOC100289509	-0.8029	0.9999	uncharacterized LOC100289509
17015	BAP1	-0.8033	0.0366	BRCA1 associated protein-1 (ubiquitin carboxy-terminal hydrolase)
17016	ZPBP2	-0.8037	0.0034	zona pellucida binding protein 2
17017	FAM120B	-0.8037	0.0079	family with sequence similarity 120B
17018	HEY1	-0.8042	0.0071	hairy/enhancer-of-split related with YRPW motif 1
17019	CBFA2T3	-0.8042	0.0086	core-binding factor, runt domain, alpha subunit 2; translocated to, 3
17020	AURKB	-0.8042	0.0965	aurora kinase B
17021	CERS1	-0.8043	0.0515	ceramide synthase 1
17022	TSSK4	-0.805	0.0046	testis-specific serine kinase 4
17023	SRM	-0.805	0.1151	spermidine synthase
17024	SKA2	-0.805	0.0351	spindle and kinetochore associated complex subunit 2
17025	SKA1	-0.805	0.0497	spindle and kinetochore associated complex subunit 1
17026	RAB37	-0.805	0.0137	RAB37, member RAS oncogene family
17027	PAF1	-0.805	0.0209	Paf1, RNA polymerase II associated factor, homolog (S. cerevisiae)
17028	ELFN2	-0.805	0.0097	extracellular leucine-rich repeat and fibronectin type III domain containing 2
17029	ZBED4	-0.8058	0.0407	zinc finger, BED-type containing 4
17030	PSMD2	-0.8058	0.0728	proteasome (prosome, macropain) 26S subunit, non-ATPase, 2
17031	HTT	-0.8058	0.0249	huntingtin
17032	FOXG1	-0.8058	0.0095	forkhead box G1
17033	AANAT	-0.8058	0.0226	aralkylamine N-acetyltransferase
17034	SLC26A8	-0.8062	0.0121	solute carrier family 26, member 8
17035	LOC100130700	-0.8062	0.0345	uncharacterized LOC100130700
17036	C2orf69	-0.8062	0.0633	chromosome 2 open reading frame 69
17037	TDRD7	-0.8067	0.0184	tudor domain containing 7
17038	LOC100128071	-0.8071	0.9999	uncharacterized LOC100128071
17039	THAP8	-0.8075	0.0243	THAP domain containing 8
17040	SPECC1	-0.8075	0.0041	sperm antigen with calponin homology and coiled-coil domains 1
17041	SMARCB1	-0.8075	0.036	SWI/SNF related, matrix associated, actin dependent regulator of chromatin, subfamily b, member 1
17042	NAT8L	-0.8075	0.0113	N-acetyltransferase 8-like (GCN5-related, putative)
17043	HLTF	-0.8075	0.1169	helicase-like transcription factor
17044	DNAJC8	-0.8075	0.0129	DnaJ (Hsp40) homolog, subfamily C, member 8
17045	CNTNAP5	-0.8075	0.0576	contactin associated protein-like 5
17046	COX7A2	-0.8083	0.0429	cytochrome c oxidase subunit VIIa polypeptide 2 (liver)
17047	JAKMIP1	-0.8087	0.0056	janus kinase and microtubule interacting protein 1
17048	WASF1	-0.8092	0.0097	WAS protein family, member 1
17049	VDAC3	-0.8092	0.0835	voltage-dependent anion channel 3
17050	RMI2	-0.81	0.0233	RMI2, RecQ mediated genome instability 2, homolog (S. cerevisiae)

17051	RASGRP4	-0.81	0.0408	RAS guanyl releasing protein 4
17052	OPTC	-0.81	0.0784	opticin
17053	OLIG2	-0.81	0.0095	oligodendrocyte lineage transcription factor 2
17054	LSM4	-0.81	0.134	LSM4 homolog, U6 small nuclear RNA associated (S. cerevisiae)
17055	FGF4	-0.81	0.0211	fibroblast growth factor 4
17056	ESF1	-0.81	0.0691	ESF1, nucleolar pre-rRNA processing protein, homolog (S. cerevisiae)
17057	CN5H6.4	-0.81	0.9999	uncharacterized LOC150384
17058	CMIP	-0.81	0.0175	c-Maf inducing protein
17059	BCAS2	-0.8108	0.0892	breast carcinoma amplified sequence 2
17060	TESPA1	-0.8112	0.0073	thymocyte expressed, positive selection associated 1
17061	NLRP5	-0.8112	0.0254	NLR family, pyrin domain containing 5
17062	MGC16025	-0.8112	0.005	uncharacterized LOC85009
17063	FIGNL1	-0.8112	0.0886	fidgetin-like 1
17064	MAD2L1	-0.8117	0.1554	MAD2 mitotic arrest deficient-like 1 (yeast)
17065	C9orf96	-0.8129	0.0972	chromosome 9 open reading frame 96
17066	SRPK2	-0.8133	0.0181	SRSF protein kinase 2
17067	PSMC3IP	-0.8133	0.0358	PSMC3 interacting protein
17068	TSEN54	-0.8138	0.0519	tRNA splicing endonuclease 54 homolog (S. cerevisiae)
17069	TNRC6C	-0.8138	0.0143	trinucleotide repeat containing 6C
17070	KIF6	-0.8138	0.0056	kinesin family member 6
17071	FSD1L	-0.8138	0.0045	fibronectin type III and SPRY domain containing 1-like
17072	CXorf58	-0.8138	0.0028	chromosome X open reading frame 58
17073	RPP21	-0.8143	0.0409	ribonuclease P/MRP 21kDa subunit
17074	STH	-0.815	0.0107	saitohin
17075	SLBP	-0.815	0.1085	stem-loop binding protein
17076	RTN4RL2	-0.815	0.0148	reticulon 4 receptor-like 2
17077	LOC100169752	-0.815	0.0047	uncharacterized LOC100169752
17078	KDM1A	-0.815	0.0317	lysine (K)-specific demethylase 1A
17079	CCT2	-0.815	0.1677	chaperonin containing TCP1, subunit 2 (beta)
17080	MCM5	-0.8158	0.1023	minichromosome maintenance complex component 5
17081	CLEC16A	-0.8158	0.0098	C-type lectin domain family 16, member A
17082	C16orf80	-0.8158	0.0516	chromosome 16 open reading frame 80
17083	TRMT10C	-0.8163	0.0933	tRNA methyltransferase 10 homolog C (S. cerevisiae)
17084	LZIC	-0.8163	0.021	leucine zipper and CTNNBIP1 domain containing
17085	TRIM28	-0.8167	0.0919	tripartite motif containing 28
17086	PSMD14	-0.8167	0.1561	proteasome (prosome, macropain) 26S subunit, non-ATPase, 14
17087	C10orf2	-0.8167	0.0464	chromosome 10 open reading frame 2
17088	TRIM9	-0.8175	0.0054	tripartite motif containing 9
17089	HAUS1	-0.8175	0.0994	HAUS augmin-like complex, subunit 1
17090	GTPBP3	-0.8175	0.0343	GTP binding protein 3 (mitochondrial)
17091	DTL	-0.8175	0.0795	denticleless E3 ubiquitin protein ligase homolog (Drosophila)
17092	TTYH1	-0.8183	0.0056	tweety homolog 1 (Drosophila)
17093	NUDT1	-0.8183	0.0767	nudix (nucleoside diphosphate linked moiety X)-type motif 1
17094	GFI1	-0.8183	0.0072	growth factor independent 1 transcription repressor
17095	LY6G6F	-0.8188	0.0138	lymphocyte antigen 6 complex, locus G6F
17096	IL1RAPL1	-0.8188	0.0038	interleukin 1 receptor accessory protein-like 1
17097	DHH	-0.8188	0.0356	desert hedgehog
17098	CIB3	-0.8188	0.0797	calcium and integrin binding family member 3
17099	EXOSC9	-0.8192	0.0437	exosome component 9
17100	NLRP7	-0.82	0.0042	NLR family, pyrin domain containing 7
17101	FAM69C	-0.82	0.0066	family with sequence similarity 69, member C
17102	CEP19	-0.82	0.0052	centrosomal protein 19kDa
17103	CENPF	-0.82	0.0654	centromere protein F, 350/400kDa (mitosin)
17104	UBE2V2	-0.8208	0.1657	ubiquitin-conjugating enzyme E2 variant 2
17105	CDC25A	-0.8208	0.08	cell division cycle 25 homolog A (S. pombe)
17106	HUS1B	-0.8213	0.0089	HUS1 checkpoint homolog b (S. pombe)
17107	TCOF1	-0.8217	0.0805	Treacher Collins-Franceschetti syndrome 1
17108	PITHD1	-0.8238	0.0077	PITH (C-terminal proteasome-interacting domain of thioredoxin-like) domain containing 1
17109	CTU2	-0.8238	0.052	cytosolic thiouridylase subunit 2 homolog (S. pombe)
17110	TOP3A	-0.8242	0.0271	topoisomerase (DNA) III alpha
17111	ASF1B	-0.8242	0.0577	ASF1 anti-silencing function 1 homolog B (S. cerevisiae)
17112	LOC100287482	-0.8243	0.0052	uncharacterized LOC100287482
17113	TAF5	-0.825	0.0518	TAF5 RNA polymerase II, TATA box binding protein (TBP)-associated factor, 100kDa
17114	SLC22A23	-0.825	0.0126	solute carrier family 22, member 23
17115	METTL6	-0.825	0.0094	methyltransferase like 6
17116	C9orf163	-0.825	0.1159	chromosome 9 open reading frame 163
17117	ATP6V1E2	-0.825	0.0036	ATPase, H+ transporting, lysosomal 31kDa, V1 subunit E2
17118	CNBD1	-0.8263	0.0052	cyclic nucleotide binding domain containing 1
17119	CCDC79	-0.8263	0.0042	coiled-coil domain containing 79
17120	SNW1	-0.8267	0.0751	SNW domain containing 1
17121	SAFB	-0.8275	0.0376	scaffold attachment factor B
17122	MAP2K2	-0.8275	0.0845	mitogen-activated protein kinase kinase 2
17123	TROAP	-0.8283	0.0819	trophinin associated protein (tastin)
17124	MIIP	-0.8283	0.0676	migration and invasion inhibitory protein
17125	LOC100144604	-0.8286	0.9999	uncharacterized LOC100144604
17126	SLC8A3	-0.8288	0.037	solute carrier family 8 (sodium/calcium exchanger), member 3
17127	RNF148	-0.8288	0.0028	ring finger protein 148
17128	ARMC12	-0.8288	0.0067	armadillo repeat containing 12
17129	KLHDC3	-0.8292	0.0206	kelch domain containing 3
17130	FAM125B	-0.8292	0.0075	family with sequence similarity 125, member B
17131	BEX1	-0.8292	0.0037	brain expressed, X-linked 1
17132	ZBTB8OS	-0.83	0.0128	zinc finger and BTB domain containing 8 opposite strand
17133	UTF1	-0.83	0.0553	undifferentiated embryonic cell transcription factor 1
17134	SSH2	-0.83	0.008	slingshot homolog 2 (Drosophila)

17135	POLR3A	-0.83	0.0146	polymerase (RNA) III (DNA directed) polypeptide A, 155kDa
17136	NUDC	-0.83	0.0632	nuclear distribution C homolog (A. nidulans)
17137	KXD1	-0.83	0.0297	KxDL motif containing 1
17138	CSMD1	-0.83	0.0207	CUB and Sushi multiple domains 1
17139	ERCC3	-0.8308	0.0453	excision repair cross-complementing rodent repair deficiency, complementation group 3
17140	EBNA1BP2	-0.8308	0.1214	EBNA1 binding protein 2
17141	SRSF12	-0.8313	0.0075	serine/arginine-rich splicing factor 12
17142	OR8G2	-0.8313	0.0033	olfactory receptor, family 8, subfamily G, member 2
17143	CELF5	-0.8313	0.0263	CUGBP, Elav-like family member 5
17144	GTF2E1	-0.8317	0.0345	general transcription factor IIE, polypeptide 1, alpha 56kDa
17145	VTA1	-0.8325	0.1013	Vps20-associated 1 homolog (S. cerevisiae)
17146	UTP18	-0.8325	0.1397	UTP18 small subunit (SSU) processome component homolog (yeast)
17147	UNC5A	-0.8325	0.0076	unc-5 homolog A (C. elegans)
17148	RANBP9	-0.8325	0.0413	RAN binding protein 9
17149	HIST1H2AB	-0.8325	0.0049	histone cluster 1, H2ab
17150	MAP4K4	-0.8333	0.0281	mitogen-activated protein kinase kinase kinase kinase 4
17151	BYSL	-0.8333	0.0741	bystin-like
17152	RHOXF1	-0.8338	0.004	Rhox homeobox family, member 1
17153	LOC647946	-0.8338	0.003	uncharacterized LOC647946
17154	HEPACAM	-0.8338	0.0186	hepatic and glial cell adhesion molecule
17155	TPX2	-0.8342	0.065	TPX2, microtubule-associated, homolog (Xenopus laevis)
17156	LSS	-0.835	0.0041	lanosterol synthase (2,3-oxidosqualene-lanosterol cyclase)
17157	KCNQ2	-0.835	0.0809	potassium voltage-gated channel, KQT-like subfamily, member 2
17158	FRMD5	-0.835	0.0042	FERM domain containing 5
17159	WRAP73	-0.8358	0.018	WD repeat containing, antisense to TP73
17160	SLC25A22	-0.8358	0.0294	solute carrier family 25 (mitochondrial carrier: glutamate), member 22
17161	KDM4D	-0.8358	0.0045	lysine (K)-specific demethylase 4D
17162	CMAS	-0.8358	0.043	cytidine monophosphate N-acetylneuraminic acid synthetase
17163	TFAP2E	-0.8363	0.0143	transcription factor AP-2 epsilon (activating enhancer binding protein 2 epsilon)
17164	SYT4	-0.8363	0.0073	synaptotagmin IV
17165	SH2D5	-0.8363	0.0401	SH2 domain containing 5
17166	RNF214	-0.8363	0.0044	ring finger protein 214
17167	HCG27	-0.8363	0.0101	HLA complex group 27 (non-protein coding)
17168	HARBI1	-0.8363	0.003	harbinger transposase derived 1
17169	EEPD1	-0.8363	0.0034	endonuclease/exonuclease/phosphatase family domain containing 1
17170	DPH3P1	-0.8363	0.0124	DPH3, KTI11 homolog (S. cerevisiae) pseudogene 1
17171	MCM6	-0.8367	0.0901	minichromosome maintenance complex component 6
17172	RECQL4	-0.8375	0.101	RecQ protein-like 4
17173	KCNJ11	-0.8375	0.0039	potassium inwardly-rectifying channel, subfamily J, member 11
17174	HNRNPM	-0.8375	0.0869	heterogeneous nuclear ribonucleoprotein M
17175	C3orf38	-0.8375	0.0901	chromosome 3 open reading frame 38
17176	PDCD5	-0.8383	0.035	programmed cell death 5
17177	BRF1	-0.8383	0.0815	BRF1 homolog, subunit of RNA polymerase III transcription initiation factor IIIB (S. cerevisiae)
17178	ACAT2	-0.8383	0.0078	acetyl-CoA acetyltransferase 2
17179	TCTE3	-0.8388	0.0233	t-complex-associated-testis-expressed 3
17180	C15orf27	-0.8388	0.0038	chromosome 15 open reading frame 27
17181	KIF20A	-0.8392	0.0451	kinesin family member 20A
17182	MYO9B	-0.84	0.0638	myosin IXB
17183	GINS3	-0.84	0.0374	GINS complex subunit 3 (Psf3 homolog)
17184	EID2	-0.84	0.0154	EP300 interacting inhibitor of differentiation 2
17185	CCDC75	-0.84	0.0038	coiled-coil domain containing 75
17186	CCDC137	-0.84	0.0338	coiled-coil domain containing 137
17187	YRDC	-0.8408	0.0214	yrdC domain containing (E. coli)
17188	WHSC1	-0.8408	0.0346	Wolf-Hirschhorn syndrome candidate 1
17189	TEX19	-0.8413	0.0059	testis expressed 19
17190	SAMD1	-0.8413	0.0273	sterile alpha motif domain containing 1
17191	CASC5	-0.8413	0.0315	cancer susceptibility candidate 5
17192	C3orf56	-0.8414	0.1252	chromosome 3 open reading frame 56
17193	PCDHGC4	-0.8425	0.0073	protocadherin gamma subfamily C, 4
17194	PARP2	-0.8425	0.048	poly (ADP-ribose) polymerase 2
17195	MARS	-0.8425	0.0183	methionyl-tRNA synthetase
17196	CCDC135	-0.8425	0.005	coiled-coil domain containing 135
17197	ABHD16B	-0.8425	0.0209	abhydrolase domain containing 16B
17198	ZNF48	-0.8433	0.0097	zinc finger protein 48
17199	MRTO4	-0.8433	0.0587	mRNA turnover 4 homolog (S. cerevisiae)
17200	EDC4	-0.8433	0.0287	enhancer of mRNA decapping 4
17201	OPN4	-0.8438	0.0695	opsin 4
17202	HN1	-0.8442	0.0292	hematological and neurological expressed 1
17203	CSNK2B	-0.8442	0.0485	casein kinase 2, beta polypeptide
17204	CPSF3L	-0.8442	0.0403	cleavage and polyadenylation specific factor 3-like
17205	PPAN	-0.845	0.083	peter pan homolog (Drosophila)
17206	MDH1	-0.845	0.0625	malate dehydrogenase 1, NAD (soluble)
17207	IZUMO1	-0.845	0.0036	izumo sperm-egg fusion 1
17208	C8orf56	-0.845	0.0036	chromosome 8 open reading frame 56
17209	WDR62	-0.8458	0.0694	WD repeat domain 62
17210	PRPSAP2	-0.8458	0.0319	phosphoribosyl pyrophosphate synthetase-associated protein 2
17211	TMEM145	-0.8462	0.0199	transmembrane protein 145
17212	PTCHD2	-0.8462	0.0573	patched domain containing 2
17213	FAM207A	-0.8462	0.0201	family with sequence similarity 207, member A
17214	CEP57L1	-0.8462	0.0073	centrosomal protein 57kDa-like 1
17215	GMNN	-0.8467	0.0762	geminin, DNA replication inhibitor
17216	HEATR7B2	-0.8475	0.0701	HEAT repeat family member 7B2
17217	PSMD4	-0.8483	0.0452	proteasome (prosome, macropain) 26S subunit, non-ATPase, 4
17218	HMMR	-0.8483	0.0587	hyaluronan-mediated motility receptor (RHAMM)

17219	DCK	-0.8483	0.0987	deoxycytidine kinase
17220	FAM181A	-0.8487	0.0567	family with sequence similarity 181, member A
17221	LRRC47	-0.8492	0.0337	leucine rich repeat containing 47
17222	HRK	-0.8492	0.005	harakiri, BCL2 interacting protein (contains only BH3 domain)
17223	PHLPP1	-0.85	0.0076	PH domain and leucine rich repeat protein phosphatase 1
17224	DNAH1	-0.85	0.059	dynein, axonemal, heavy chain 1
17225	CCNE2	-0.85	0.0279	cyclin E2
17226	MAST1	-0.8508	0.0338	microtubule associated serine/threonine kinase 1
17227	CORO7	-0.8508	0.0301	coronin 7
17228	CLPP	-0.8517	0.0744	ClpP caseinolytic peptidase, ATP-dependent, proteolytic subunit homolog (E. coli)
17229	PHOSPHO1	-0.8525	0.0039	phosphatase, orphan 1
17230	FAM123A	-0.8525	0.017	family with sequence similarity 123A
17231	WDR5	-0.8529	0.0411	WD repeat domain 5
17232	KLLN	-0.8529	0.9999	killin, p53-regulated DNA replication inhibitor
17233	SLC22A31	-0.8533	0.9999	solute carrier family 22, member 31
17234	CROCCP2	-0.8533	0.0147	ciliary rootlet coiled-coil, rootletin pseudogene 2
17235	KCNH8	-0.8537	0.0048	potassium voltage-gated channel, subfamily H (eag-related), member 8
17236	ZDHHC14	-0.8542	0.0022	zinc finger, DHHC-type containing 14
17237	FLJ22184	-0.8542	0.0967	putative uncharacterized protein FLJ22184
17238	SLC25A48	-0.855	0.0131	solute carrier family 25, member 48
17239	MEA1	-0.855	0.0371	male-enhanced antigen 1
17240	CCDC86	-0.855	0.0519	coiled-coil domain containing 86
17241	NFKBIB	-0.8558	0.0297	nuclear factor of kappa light polypeptide gene enhancer in B-cells inhibitor, beta
17242	MTOR	-0.8558	0.0093	mechanistic target of rapamycin (serine/threonine kinase)
17243	CCNF	-0.8558	0.0541	cyclin F
17244	LOH12CR1	-0.8562	0.0028	loss of heterozygosity, 12, chromosomal region 1
17245	SCAMP5	-0.8575	0.0068	secretory carrier membrane protein 5
17246	LRFN2	-0.8575	0.0045	leucine rich repeat and fibronectin type III domain containing 2
17247	PDE6D	-0.8583	0.009	phosphodiesterase 6D, cGMP-specific, rod, delta
17248	FTSJD2	-0.8583	0.0106	FtsJ methyltransferase domain containing 2
17249	RRP36	-0.8587	0.0246	ribosomal RNA processing 36 homolog (S. cerevisiae)
17250	NDUFAF5	-0.8589	0.0082	NADH dehydrogenase (ubiquinone) complex I, assembly factor 5
17251	RUVBL2	-0.8592	0.0931	RuvB-like 2 (E. coli)
17252	CCDC88A	-0.8592	0.0167	coiled-coil domain containing 88A
17253	ARHGAP33	-0.8592	0.1548	Rho GTPase activating protein 33
17254	VWA5B2	-0.86	0.1486	von Willebrand factor A domain containing 5B2
17255	TADA1	-0.86	0.0562	transcriptional adaptor 1
17256	ATP1A4	-0.86	0.0061	ATPase, Na+/K+ transporting, alpha 4 polypeptide
17257	UBA52	-0.8608	0.0087	ubiquitin A-52 residue ribosomal protein fusion product 1
17258	HAGHL	-0.8612	0.052	hydroxyacylglutathione hydrolase-like
17259	RNF8	-0.8633	0.0042	ring finger protein 8, E3 ubiquitin protein ligase
17260	POLR2F	-0.8633	0.0456	polymerase (RNA) II (DNA directed) polypeptide F
17261	POLB	-0.8633	0.0039	polymerase (DNA directed), beta
17262	ME2	-0.8633	0.0257	malic enzyme 2, NAD(+)-dependent, mitochondrial
17263	MADD	-0.8633	0.023	MAP-kinase activating death domain
17264	ZNF645	-0.8637	0.0108	zinc finger protein 645
17265	TOMM40L	-0.8637	0.0048	translocase of outer mitochondrial membrane 40 homolog (yeast)-like
17266	ADAMTS17	-0.8637	0.0031	ADAM metallopeptidase with thrombospondin type 1 motif, 17
17267	DHPS	-0.8642	0.0288	deoxyhypusine synthase
17268	SLC7A5	-0.865	0.0115	solute carrier family 7 (amino acid transporter light chain, L system), member 5
17269	PFDN6	-0.865	0.0142	prefoldin subunit 6
17270	FANCA	-0.865	0.0383	Fanconi anemia, complementation group A
17271	C7orf61	-0.865	0.0078	chromosome 7 open reading frame 61
17272	C4orf21	-0.8657	0.0154	chromosome 4 open reading frame 21
17273	ZNF738	-0.8662	0.018	zinc finger protein 738
17274	TEX34	-0.8662	0.1075	testis expressed 34
17275	ERICH1	-0.8662	0.0037	glutamate-rich 1
17276	PSMG1	-0.8667	0.086	proteasome (prosome, macropain) assembly chaperone 1
17277	NUP50	-0.8667	0.0102	nucleoporin 50kDa
17278	DOHH	-0.8667	0.0488	deoxyhypusine hydroxylase/monooxygenase
17279	TTK	-0.8675	0.0638	TTK protein kinase
17280	RANGRF	-0.8675	0.0125	RAN guanine nucleotide release factor
17281	LRWD1	-0.8675	0.0152	leucine-rich repeats and WD repeat domain containing 1
17282	CCDC62	-0.8675	0.0071	coiled-coil domain containing 62
17283	ARPC5L	-0.8683	0.0231	actin related protein 2/3 complex, subunit 5-like
17284	TMEM86B	-0.8687	0.0143	transmembrane protein 86B
17285	PHRF1	-0.8687	0.0188	PHD and ring finger domains 1
17286	LRR1	-0.8687	0.0606	leucine rich repeat protein 1
17287	FAM122C	-0.8687	0.0043	family with sequence similarity 122C
17288	PODXL2	-0.87	0.0112	podocalyxin-like 2
17289	PELP1	-0.87	0.0381	proline, glutamate and leucine rich protein 1
17290	GNAZ	-0.87	0.0057	guanine nucleotide binding protein (G protein), alpha z polypeptide
17291	ZSCAN10	-0.8712	0.0433	zinc finger and SCAN domain containing 10
17292	ILF3	-0.8717	0.0805	interleukin enhancer binding factor 3, 90kDa
17293	GLRX5	-0.8725	0.0161	glutaredoxin 5
17294	FADS1	-0.8733	0.003	fatty acid desaturase 1
17295	MYPOP	-0.8737	0.0196	Myb-related transcription factor, partner of profilin
17296	IGDCC3	-0.8737	0.0423	immunoglobulin superfamily, DCC subclass, member 3
17297	CSMD3	-0.8737	0.0035	CUB and Sushi multiple domains 3
17298	CDCA3	-0.8742	0.0509	cell division cycle associated 3
17299	TFPT	-0.875	0.0137	TCF3 (E2A) fusion partner (in childhood Leukemia)
17300	MIR7-3HG	-0.875	0.0091	MIR7-3 host gene (non-protein coding)
17301	LOC400794	-0.875	0.0163	uncharacterized LOC400794
17302	CPA5	-0.875	0.0039	carboxypeptidase A5

17303	CIT	-0.875	0.0103	citron (rho-interacting, serine/threonine kinase 21)
17304	MTMR4	-0.8758	0.0111	myotubularin related protein 4
17305	AZI1	-0.8758	0.028	5-azacytidine induced 1
17306	E2F1	-0.8767	0.0565	E2F transcription factor 1
17307	TSGA13	-0.8775	0.0104	testis specific, 13
17308	TAF9	-0.8775	0.0771	TAF9 RNA polymerase II, TATA box binding protein (TBP)-associated factor, 32kDa
17309	SH3GL3	-0.8775	0.0093	SH3-domain GRB2-like 3
17310	PCSK1N	-0.8775	0.0091	proprotein convertase subtilisin/kexin type 1 inhibitor
17311	CEP128	-0.8775	0.0084	centrosomal protein 128kDa
17312	C6orf136	-0.8775	0.0126	chromosome 6 open reading frame 136
17313	ZNF670	-0.8788	0.0113	zinc finger protein 670
17314	EID2B	-0.8788	0.0106	EP300 interacting inhibitor of differentiation 2B
17315	GPR19	-0.8792	0.0102	G protein-coupled receptor 19
17316	XRCC3	-0.88	0.0441	X-ray repair complementing defective repair in Chinese hamster cells 3
17317	SNRPE	-0.88	0.0074	small nuclear ribonucleoprotein polypeptide E
17318	SLIT1	-0.88	0.0187	slit homolog 1 (Drosophila)
17319	GKAP1	-0.88	0.0239	G kinase anchoring protein 1
17320	C1orf135	-0.88	0.0378	chromosome 1 open reading frame 135
17321	BUB1	-0.8808	0.045	budding uninhibited by benzimidazoles 1 homolog (yeast)
17322	TECTB	-0.8813	0.0038	tectorin beta
17323	CHCHD4	-0.8813	0.0142	coiled-coil-helix-coiled-coil-helix domain containing 4
17324	ACSF3	-0.8813	0.0061	acyl-CoA synthetase family member 3
17325	CCDC34	-0.8825	0.0154	coiled-coil domain containing 34
17326	TTLL13	-0.8829	0.0051	tubulin tyrosine ligase-like family, member 13
17327	PABPN1	-0.8829	0.0078	poly(A) binding protein, nuclear 1
17328	TEX10	-0.8833	0.0228	testis expressed 10
17329	BRSK2	-0.8833	0.0072	BR serine/threonine kinase 2
17330	BRAP	-0.8833	0.0089	BRCA1 associated protein
17331	AGTPBP1	-0.8833	0.015	ATP/GTP binding protein 1
17332	POC5	-0.8838	0.0078	POC5 centriolar protein homolog (Chlamydomonas)
17333	NDUFAF6	-0.8838	0.0063	NADH dehydrogenase (ubiquinone) complex I, assembly factor 6
17334	MMD	-0.8842	0.0044	monocyte to macrophage differentiation-associated
17335	XPO5	-0.885	0.0257	exportin 5
17336	PPAT	-0.885	0.0417	phosphoribosyl pyrophosphate amidotransferase
17337	RAD51AP1	-0.8858	0.0681	RAD51 associated protein 1
17338	SOHLH1	-0.8863	0.0165	spermatogenesis and oogenesis specific basic helix-loop-helix 1
17339	GPANK1	-0.8863	0.004	G patch domain and ankyrin repeats 1
17340	DHX33	-0.8863	0.016	DEAH (Asp-Glu-Ala-His) box polypeptide 33
17341	C5orf25	-0.8863	0.0033	chromosome 5 open reading frame 25
17342	FOXK2	-0.8867	0.0099	forkhead box K2
17343	PGAM5	-0.8875	0.021	phosphoglycerate mutase family member 5
17344	HIST3H3	-0.8875	0.0028	histone cluster 3, H3
17345	C19orf76	-0.8875	0.0079	chromosome 19 open reading frame 76
17346	TMEM63C	-0.8886	0.0049	transmembrane protein 63C
17347	PSIP1	-0.8892	0.0785	PC4 and SFRS1 interacting protein 1
17348	VWCE	-0.89	0.0054	von Willebrand factor C and EGF domains
17349	OR2B3	-0.89	0.0318	olfactory receptor, family 2, subfamily B, member 3
17350	GPR137C	-0.8913	0.0046	G protein-coupled receptor 137C
17351	KHDC3L	-0.8914	0.0575	KH domain containing 3-like, subcortical maternal complex member
17352	SF3A2	-0.8917	0.0258	splicing factor 3a, subunit 2, 66kDa
17353	PUF60	-0.8917	0.053	poly-U binding splicing factor 60KDa
17354	TCEB1	-0.8925	0.051	transcription elongation factor B (SIII), polypeptide 1 (15kDa, elongin C)
17355	TBP	-0.8925	0.0061	TATA box binding protein
17356	NOP58	-0.8925	0.044	NOP58 ribonucleoprotein homolog (yeast)
17357	NLRC4	-0.8925	0.0101	NLR family, CARD domain containing 4
17358	DNAJC5B	-0.8925	0.0104	DnaJ (Hsp40) homolog, subfamily C, member 5 beta
17359	WDR3	-0.8933	0.0442	WD repeat domain 3
17360	TAL2	-0.8938	0.0117	T-cell acute lymphocytic leukemia 2
17361	CCDC38	-0.8938	0.0035	coiled-coil domain containing 38
17362	MLF1IP	-0.8942	0.0249	MLF1 interacting protein
17363	HDAC2	-0.8942	0.0346	histone deacetylase 2
17364	GDAP1	-0.8942	0.004	ganglioside induced differentiation associated protein 1
17365	ATP1A3	-0.8942	0.0037	ATPase, Na+/K+ transporting, alpha 3 polypeptide
17366	ZNF473	-0.895	0.006	zinc finger protein 473
17367	PRRC2A	-0.895	0.0429	proline-rich coiled-coil 2A
17368	HIST1H3B	-0.895	0.0039	histone cluster 1, H3b
17369	ZNF428	-0.8958	0.0104	zinc finger protein 428
17370	PPP1R8	-0.8958	0.0303	protein phosphatase 1, regulatory subunit 8
17371	PES1	-0.8958	0.0331	pescadillo ribosomal biogenesis factor 1
17372	LOC100130357	-0.8967	0.9999	uncharacterized LOC100130357
17373	CSNK1G2	-0.8967	0.0129	casein kinase 1, gamma 2
17374	CCT4	-0.8967	0.0799	chaperonin containing TCP1, subunit 4 (delta)
17375	ADRBK2	-0.8967	0.0046	adrenergic, beta, receptor kinase 2
17376	TIPIN	-0.8975	0.0464	TIMELESS interacting protein
17377	HIC2	-0.8983	0.01	hypermethylated in cancer 2
17378	ZNF684	-0.8988	0.0103	zinc finger protein 684
17379	SLC17A8	-0.8988	0.0031	solute carrier family 17 (sodium-dependent inorganic phosphate cotransporter), member 8
17380	LOC100129250	-0.8991	0.0133	uncharacterized LOC100129250
17381	RRP1B	-0.9	0.0142	ribosomal RNA processing 1 homolog B (S. cerevisiae)
17382	NSL1	-0.9	0.0134	NSL1, MIND kinetochore complex component, homolog (S. cerevisiae)
17383	MBLAC1	-0.9	0.0099	metallo-beta-lactamase domain containing 1
17384	HELLS	-0.9	0.0383	helicase, lymphoid-specific
17385	GINS2	-0.9008	0.048	GINS complex subunit 2 (Psf2 homolog)
17386	IQCB1	-0.9017	0.0096	IQ motif containing B1

17387	RNASEH2C	-0.9025	0.0076	ribonuclease H2, subunit C
17388	PLEKHM2	-0.9025	0.0228	pleckstrin homology domain containing, family M (with RUN domain) member 2
17389	KIAA1211	-0.9025	0.0039	KIAA1211
17390	KCTD13	-0.9033	0.0062	potassium channel tetramerisation domain containing 13
17391	SEC61A2	-0.9042	0.0041	Sec61 alpha 2 subunit (S. cerevisiae)
17392	MGC57346	-0.9043	0.0032	uncharacterized LOC401884
17393	SLC16A10	-0.905	0.0038	solute carrier family 16, member 10 (aromatic amino acid transporter)
17394	CDK5R1	-0.9067	0.0045	cyclin-dependent kinase 5, regulatory subunit 1 (p35)
17395	MID1IP1	-0.9075	0.0029	MID1 interacting protein 1
17396	DLGAP5	-0.9075	0.0374	discs, large (Drosophila) homolog-associated protein 5
17397	ADAD1	-0.9075	0.0052	adenosine deaminase domain containing 1 (testis-specific)
17398	CDC20	-0.9083	0.0473	cell division cycle 20 homolog (S. cerevisiae)
17399	GPSM1	-0.9087	0.0132	G-protein signaling modulator 1
17400	C16orf79	-0.9087	0.0193	chromosome 16 open reading frame 79
17401	ANO8	-0.91	0.0058	anoctamin 8
17402	SUPT5H	-0.9108	0.0171	suppressor of Ty 5 homolog (S. cerevisiae)
17403	KHDC1	-0.9112	0.0035	KH homology domain containing 1
17404	ZC3H18	-0.9125	0.011	zinc finger CCCH-type containing 18
17405	LIN52	-0.9125	0.0037	lin-52 homolog (C. elegans)
17406	MAD2L1BP	-0.9133	0.0065	MAD2L1 binding protein
17407	TAMM41	-0.9137	0.0032	TAM41, mitochondrial translocator assembly and maintenance protein, homolog (S. cerevisiae)
17408	C16orf92	-0.9137	0.0045	chromosome 16 open reading frame 92
17409	MCM7	-0.915	0.0536	minichromosome maintenance complex component 7
17410	TRMT1	-0.9158	0.0282	tRNA methyltransferase 1 homolog (S. cerevisiae)
17411	ZNF496	-0.9175	0.0048	zinc finger protein 496
17412	MRPS17	-0.9175	0.033	mitochondrial ribosomal protein S17
17413	MARCH9	-0.9175	0.0056	membrane-associated ring finger (C3HC4) 9
17414	LINC00471	-0.9187	0.0039	long intergenic non-protein coding RNA 471
17415	MBD3	-0.9192	0.0528	methyl-CpG binding domain protein 3
17416	C14orf2	-0.9192	0.0191	chromosome 14 open reading frame 2
17417	BIRC5	-0.9192	0.044	baculoviral IAP repeat containing 5
17418	TBCC	-0.92	0.0068	tubulin folding cofactor C
17419	PSMB7	-0.92	0.0359	proteasome (prosome, macropain) subunit, beta type, 7
17420	PARS2	-0.92	0.0044	prolyl-tRNA synthetase 2, mitochondrial (putative)
17421	LMNB2	-0.92	0.0414	lamin B2
17422	KIAA1586	-0.92	0.01	KIAA1586
17423	DCST2	-0.92	0.0047	DC-STAMP domain containing 2
17424	ENHO	-0.9212	0.0109	energy homeostasis associated
17425	TFIP11	-0.9217	0.0054	tuftelin interacting protein 11
17426	ACVR2B	-0.9217	0.0111	activin A receptor, type IIB
17427	MCM4	-0.9225	0.0356	minichromosome maintenance complex component 4
17428	DHRS13	-0.9225	0.0045	dehydrogenase/reductase (SDR family) member 13
17429	CENPA	-0.9225	0.0255	centromere protein A
17430	ZNF100	-0.9237	0.006	zinc finger protein 100
17431	SASS6	-0.9237	0.0405	spindle assembly 6 homolog (C. elegans)
17432	LINC00326	-0.9237	0.0026	long intergenic non-protein coding RNA 326
17433	CNIH2	-0.9237	0.0157	cornichon homolog 2 (Drosophila)
17434	MED27	-0.925	0.0054	mediator complex subunit 27
17435	KSR2	-0.925	0.003	kinase suppressor of ras 2
17436	OR51B6	-0.9257	0.0236	olfactory receptor, family 51, subfamily B, member 6
17437	FAM59B	-0.9257	0.0052	family with sequence similarity 59, member B
17438	TNPO2	-0.9258	0.011	transportin 2
17439	THOP1	-0.9258	0.0286	thimet oligopeptidase 1
17440	PSMD6	-0.9258	0.032	proteasome (prosome, macropain) 26S subunit, non-ATPase, 6
17441	TXNDC16	-0.9262	0.0138	thioredoxin domain containing 16
17442	RNF166	-0.9275	0.0057	ring finger protein 166
17443	GPHB5	-0.9275	0.0091	glycoprotein hormone beta 5
17444	FAM222A	-0.9275	0.0046	family with sequence similarity 222, member A
17445	BAG6	-0.9275	0.0254	BCL2-associated athanogene 6
17446	ENO2	-0.9283	0.0035	enolase 2 (gamma, neuronal)
17447	DAZAP1	-0.9283	0.0145	DAZ associated protein 1
17448	TOPBP1	-0.9292	0.0815	topoisomerase (DNA) II binding protein 1
17449	LOC285401	-0.93	0.9996	uncharacterized LOC285401
17450	DYNLL2	-0.93	0.0041	dynein, light chain, LC8-type 2
17451	DBF4	-0.93	0.0348	DBF4 homolog (S. cerevisiae)
17452	CENPM	-0.9308	0.0336	centromere protein M
17453	KIAA1967	-0.9312	0.0026	KIAA1967
17454	ELOF1	-0.9312	0.0078	elongation factor 1 homolog (S. cerevisiae)
17455	CCDC58	-0.9325	0.0065	coiled-coil domain containing 58
17456	CARM1	-0.9327	0.0114	coactivator-associated arginine methyltransferase 1
17457	PGBD1	-0.9337	0.0031	piggyBac transposable element derived 1
17458	EXOSC3	-0.9337	0.0255	exosome component 3
17459	CDC45	-0.935	0.0333	cell division cycle 45 homolog (S. cerevisiae)
17460	C1orf182	-0.935	0.0079	chromosome 1 open reading frame 182
17461	S100Z	-0.9362	0.0023	S100 calcium binding protein Z
17462	OR51B5	-0.9362	0.0108	olfactory receptor, family 51, subfamily B, member 5
17463	C3orf22	-0.9362	0.0084	chromosome 3 open reading frame 22
17464	MELK	-0.9375	0.0223	maternal embryonic leucine zipper kinase
17465	HS3ST4	-0.9383	0.007	heparan sulfate (glucosamine) 3-O-sulfotransferase 4
17466	FBXO41	-0.9383	0.0064	F-box protein 41
17467	FANCM	-0.9388	0.0095	Fanconi anemia, complementation group M
17468	CHAC2	-0.9388	0.023	ChaC, cation transport regulator homolog 2 (E. coli)
17469	RBM15	-0.9392	0.0095	RNA binding motif protein 15
17470	PPP6R1	-0.9392	0.0097	protein phosphatase 6, regulatory subunit 1

17471	SRRM2-AS1	-0.94	0.9999	SRRM2 antisense RNA 1 (non-protein coding)
17472	GPR55	-0.94	0.0059	G protein-coupled receptor 55
17473	SMARCA4	-0.9408	0.0231	SWI/SNF related, matrix associated, actin dependent regulator of chromatin, subfamily a, member 4
17474	CDKN3	-0.9408	0.0175	cyclin-dependent kinase inhibitor 3
17475	CCT8	-0.9408	0.0477	chaperonin containing TCP1, subunit 8 (theta)
17476	SYNE3	-0.9413	0.0039	spectrin repeat containing, nuclear envelope family member 3
17477	MGAT5B	-0.9413	0.0075	mannosyl (alpha-1,6-)-glycoprotein beta-1,6-N-acetyl-glucosaminyltransferase, isozyme B
17478	UBE2O	-0.9417	0.0084	ubiquitin-conjugating enzyme E2O
17479	DDX39A	-0.9417	0.0325	DEAD (Asp-Glu-Ala-Asp) box polypeptide 39A
17480	LOC441204	-0.9425	0.0112	uncharacterized LOC441204
17481	CELF4	-0.9425	0.0127	CUGBP, Elav-like family member 4
17482	CECR6	-0.9425	0.0037	cat eye syndrome chromosome region, candidate 6
17483	NOSIP	-0.9433	0.0106	nitric oxide synthase interacting protein
17484	GTSE1	-0.9433	0.0235	G-2 and S-phase expressed 1
17485	ZMYND19	-0.9438	0.0228	zinc finger, MYND-type containing 19
17486	TIGD3	-0.9438	0.0045	tigger transposable element derived 3
17487	SAP30	-0.9442	0.0031	Sin3A-associated protein, 30kDa
17488	KLF16	-0.945	0.0151	Kruppel-like factor 16
17489	EMC8	-0.945	0.0122	ER membrane protein complex subunit 8
17490	TUBG1	-0.9458	0.0303	tubulin, gamma 1
17491	ZSWIM3	-0.9463	0.0027	zinc finger, SWIM-type containing 3
17492	KIF2C	-0.9467	0.0203	kinesin family member 2C
17493	MCM8	-0.9488	0.0091	minichromosome maintenance complex component 8
17494	PLEKHO1	-0.9492	0.0062	pleckstrin homology domain containing, family O member 1
17495	WDR85	-0.95	0.0055	WD repeat domain 85
17496	TSSK3	-0.95	0.007	testis-specific serine kinase 3
17497	RPTOR	-0.95	0.0031	regulatory associated protein of MTOR, complex 1
17498	PTRH2	-0.95	0.0062	peptidyl-tRNA hydrolase 2
17499	LOC100271832	-0.95	0.9999	uncharacterized LOC100271832
17500	LINC00528	-0.95	0.9996	long intergenic non-protein coding RNA 528
17501	GNAO1	-0.95	0.0265	guanine nucleotide binding protein (G protein), alpha activating activity polypeptide O
17502	FAM100B	-0.95	0.0037	family with sequence similarity 100, member B
17503	C11orf73	-0.9508	0.0041	chromosome 11 open reading frame 73
17504	SVOP	-0.9513	0.0033	SV2 related protein homolog (rat)
17505	NSUN6	-0.9513	0.0036	NOP2/Sun domain family, member 6
17506	C19orf26	-0.9513	0.0391	chromosome 19 open reading frame 26
17507	ZCCHC18	-0.9525	0.0028	zinc finger, CCHC domain containing 18
17508	SAP130	-0.9525	0.0038	Sin3A-associated protein, 130kDa
17509	NDC80	-0.9525	0.0146	NDC80 kinetochore complex component homolog (S. cerevisiae)
17510	PRPF19	-0.9533	0.0061	PRP19/PSO4 pre-mRNA processing factor 19 homolog (S. cerevisiae)
17511	C12orf60	-0.9538	0.0035	chromosome 12 open reading frame 60
17512	YPEL1	-0.9542	0.0038	yippee-like 1 (Drosophila)
17513	LONP1	-0.9542	0.0089	lon peptidase 1, mitochondrial
17514	NHP2L1	-0.955	0.0134	NHP2 non-histone chromosome protein 2-like 1 (S. cerevisiae)
17515	CKAP2L	-0.955	0.0079	cytoskeleton associated protein 2-like
17516	C16orf87	-0.955	0.0046	chromosome 16 open reading frame 87
17517	XPO6	-0.9558	0.004	exportin 6
17518	RIOK1	-0.9563	0.0116	RIO kinase 1 (yeast)
17519	DBF4B	-0.9564	0.0049	DBF4 homolog B (S. cerevisiae)
17520	AACSP1	-0.9571	0.9986	acetoacetyl-CoA synthetase pseudogene 1
17521	GRB2	-0.9575	0.0047	growth factor receptor-bound protein 2
17522	FCHO1	-0.9575	0.0039	FCH domain only 1
17523	ASPHD2	-0.9575	0.0027	aspartate beta-hydroxylase domain containing 2
17524	PDRG1	-0.9588	0.0042	p53 and DNA-damage regulated 1
17525	PRIM1	-0.9592	0.017	primase, DNA, polypeptide 1 (49kDa)
17526	MYT1L	-0.96	0.0023	myelin transcription factor 1-like
17527	BEND3	-0.96	0.0049	BEN domain containing 3
17528	ABHD8	-0.96	0.0038	abhydrolase domain containing 8
17529	UNC80	-0.9613	0.0025	unc-80 homolog (C. elegans)
17530	NLRP11	-0.9613	0.0038	NLR family, pyrin domain containing 11
17531	MED12L	-0.9613	0.0056	mediator complex subunit 12-like
17532	AARS2	-0.9625	0.0042	alanyl-tRNA synthetase 2, mitochondrial (putative)
17533	DGCR14	-0.9642	0.0031	DiGeorge syndrome critical region gene 14
17534	C7orf26	-0.9642	0.0051	chromosome 7 open reading frame 26
17535	YEATS4	-0.965	0.0116	YEATS domain containing 4
17536	CDCA8	-0.9658	0.009	cell division cycle associated 8
17537	RPS6KL1	-0.9663	0.0028	ribosomal protein S6 kinase-like 1
17538	NCAPG	-0.9667	0.0148	non-SMC condensin I complex, subunit G
17539	RNF138	-0.9675	0.0191	ring finger protein 138, E3 ubiquitin protein ligase
17540	CCNE1	-0.9675	0.0037	cyclin E1
17541	KHSRP	-0.9683	0.0075	KH-type splicing regulatory protein
17542	LOC148696	-0.9686	0.0093	uncharacterized LOC148696
17543	RNF220	-0.9692	0.0036	ring finger protein 220
17544	KIF15	-0.97	0.0079	kinesin family member 15
17545	IGLON5	-0.97	0.0035	IgLON family member 5
17546	RACGAP1	-0.9708	0.007	Rac GTPase activating protein 1
17547	ZNF689	-0.9712	0.0039	zinc finger protein 689
17548	MRPL14	-0.9712	0.0049	mitochondrial ribosomal protein L14
17549	MEIG1	-0.9712	0.0026	meiosis expressed gene 1 homolog (mouse)
17550	KCNH2	-0.9717	0.0029	potassium voltage-gated channel, subfamily H (eag-related), member 2
17551	SCGB2B2	-0.9725	0.0027	secretoglobin, family 2B, member 2
17552	RNPS1	-0.9733	0.0041	RNA binding protein S1, serine-rich domain
17553	LINC00176	-0.9737	0.0038	long intergenic non-protein coding RNA 176
17554	NOP56	-0.9742	0.0117	NOP56 ribonucleoprotein homolog (yeast)

17555	SLAIN1	-0.975	0.003	SLAIN motif family, member 1
17556	LOC643201	-0.9767	0.9996	centrosomal protein 192kDa pseudogene
17557	TRIM36	-0.9775	0.0018	tripartite motif containing 36
17558	TMEM163	-0.9775	0.0018	transmembrane protein 163
17559	UBE2N	-0.9783	0.0067	ubiquitin-conjugating enzyme E2N
17560	PSTK	-0.9787	0.0036	phosphoseryl-tRNA kinase
17561	ATXN7L2	-0.9787	0.004	ataxin 7-like 2
17562	SCG3	-0.9792	0.0036	secretogranin III
17563	ZUFSP	-0.98	0.0047	zinc finger with UFM1-specific peptidase domain
17564	PIP4K2A	-0.9808	0.003	phosphatidylinositol-5-phosphate 4-kinase, type II, alpha
17565	DPF1	-0.9808	0.0049	D4, zinc and double PHD fingers family 1
17566	LOC284661	-0.9814	0.9996	uncharacterized LOC284661
17567	DYNC1LI1	-0.9833	0.0041	dynein, cytoplasmic 1, light intermediate chain 1
17568	ZNF445	-0.9837	0.0039	zinc finger protein 445
17569	RNF182	-0.9837	0.0031	ring finger protein 182
17570	TMEM206	-0.985	0.0035	transmembrane protein 206
17571	RNMTL1	-0.9858	0.0045	RNA methyltransferase like 1
17572	TTC9B	-0.9862	0.0045	tetratricopeptide repeat domain 9B
17573	SLC25A33	-0.9862	0.0034	solute carrier family 25 (pyrimidine nucleotide carrier), member 33
17574	FAM186B	-0.9862	0.0028	family with sequence similarity 186, member B
17575	ESCO1	-0.9862	0.0041	establishment of cohesion 1 homolog 1 (S. cerevisiae)
17576	ATG4D	-0.9862	0.0039	autophagy related 4D, cysteine peptidase
17577	TERF2IP	-0.9875	0.003	telomeric repeat binding factor 2, interacting protein
17578	RTBDN	-0.9875	0.006	retbindin
17579	CHTF18	-0.9875	0.0084	CTF18, chromosome transmission fidelity factor 18 homolog (S. cerevisiae)
17580	CENPV	-0.9875	0.0033	centromere protein V
17581	LOC144486	-0.9887	0.0035	uncharacterized LOC144486
17582	TBPL1	-0.99	0.0012	TBP-like 1
17583	CALM3	-0.99	0.003	calmodulin 3 (phosphorylase kinase, delta)
17584	SPC25	-0.9917	0.0036	SPC25, NDC80 kinetochore complex component, homolog (S. cerevisiae)
17585	MIS18A	-0.9917	0.0039	MIS18 kinetochore protein homolog A (S. pombe)
17586	SENP5	-0.9925	0.0028	SUMO1/sentrin specific peptidase 5
17587	POP7	-0.9942	0.0063	processing of precursor 7, ribonuclease P/MRP subunit (S. cerevisiae)
17588	SUGP1	-0.9958	0.0028	SURP and G patch domain containing 1
17589	VARS2	-0.9962	0.0041	valyl-tRNA synthetase 2, mitochondrial (putative)
17590	RBX1	-0.9967	0.0012	ring-box 1, E3 ubiquitin protein ligase
17591	FRS3	-0.9967	0.0034	fibroblast growth factor receptor substrate 3
17592	DONSON	-0.9967	0.0028	downstream neighbor of SON
17593	NCAPH2	-0.9992	0.006	non-SMC condensin II complex, subunit H2
17594	CDC7	-0.9992	0.0035	cell division cycle 7 homolog (S. cerevisiae)
17595	USF1	-1.0025	0.0035	upstream transcription factor 1
17596	SLIRP	-1.0025	0.0032	SRA stem-loop interacting RNA binding protein
17597	RASGEF1C	-1.0025	0.003	RasGEF domain family, member 1C
17598	NRSN1	-1.0025	0.0035	neurensin 1
17599	ODF2	-1.0033	0.002	outer dense fiber of sperm tails 2
17600	XRCC6BP1	-1.0037	0.0019	XRCC6 binding protein 1
17601	KTI12	-1.0037	0.003	KTI12 homolog, chromatin associated (S. cerevisiae)
17602	FARSB	-1.0037	0.0035	phenylalanyl-tRNA synthetase, beta subunit
17603	EZH2	-1.0042	0.0025	enhancer of zeste homolog 2 (Drosophila)
17604	FANCB	-1.0087	0.0024	Fanconi anemia, complementation group B
17605	ATRIP	-1.0087	0.0027	ATR interacting protein
17606	TOR2A	-1.01	0.0026	torsin family 2, member A
17607	INCENP	-1.01	0.0026	inner centromere protein antigens 135/155kDa
17608	DNHD1	-1.01	0.0032	dynein heavy chain domain 1
17609	LOC728485	-1.0125	0.0033	uncharacterized LOC728485
17610	CDKN2D	-1.0133	0.0042	cyclin-dependent kinase inhibitor 2D (p19, inhibits CDK4)
17611	ZSCAN22	-1.0137	0.0025	zinc finger and SCAN domain containing 22
17612	CDK19	-1.015	0.0025	cyclin-dependent kinase 19
17613	AP4B1	-1.015	0.0037	adaptor-related protein complex 4, beta 1 subunit
17614	SS18L2	-1.0158	0.0028	synovial sarcoma translocation gene on chromosome 18-like 2
17615	RAD54L	-1.0158	0.002	RAD54-like (S. cerevisiae)
17616	C1orf100	-1.0163	0.0033	chromosome 1 open reading frame 100
17617	PIF1	-1.0175	0.0037	PIF1 5'-to-3' DNA helicase homolog (S. cerevisiae)
17618	LRFN1	-1.0175	0.0025	leucine rich repeat and fibronectin type III domain containing 1
17619	UBE2D2	-1.0192	0.0027	ubiquitin-conjugating enzyme E2D 2
17620	LOC150051	-1.02	0.9999	uncharacterized LOC150051
17621	CNOT10	-1.02	0.0027	CCR4-NOT transcription complex, subunit 10
17622	CENPN	-1.02	0.003	centromere protein N
17623	TRAIP	-1.0208	0.0036	TRAF interacting protein
17624	RPAIN	-1.0213	0.0024	RPA interacting protein
17625	FAM178B	-1.0213	0.0025	family with sequence similarity 178, member B
17626	GEMIN2	-1.0217	0.0015	gem (nuclear organelle) associated protein 2
17627	SLX4	-1.0225	0.0045	SLX4 structure-specific endonuclease subunit homolog (S. cerevisiae)
17628	TBCA	-1.0229	0.0027	tubulin folding cofactor A
17629	TRIM37	-1.0233	0.0031	tripartite motif containing 37
17630	SAC3D1	-1.0242	0.0028	SAC3 domain containing 1
17631	ORC6	-1.0242	0.0036	origin recognition complex, subunit 6
17632	CLVS2	-1.0243	0.0041	clavesin 2
17633	MDC1	-1.025	0	mediator of DNA-damage checkpoint 1
17634	SPANXA2-OT1	-1.0263	0.0041	SPANXA2 overlapping transcript 1 (non-protein coding)
17635	NCAPH	-1.0267	0.0029	non-SMC condensin I complex, subunit H
17636	SLC19A1	-1.0283	0.0027	solute carrier family 19 (folate transporter), member 1
17637	CHERP	-1.0283	0.0032	calcium homeostasis endoplasmic reticulum protein
17638	CDT1	-1.03	0.0033	chromatin licensing and DNA replication factor 1

17639	CCT7	-1.03	0.0032	chaperonin containing TCP1, subunit 7 (eta)
17640	FZR1	-1.0308	0.0027	fizzy/cell division cycle 20 related 1 (Drosophila)
17641	E2F2	-1.0308	0.0019	E2F transcription factor 2
17642	PKMYT1	-1.0317	0.0006	protein kinase, membrane associated tyrosine/threonine 1
17643	PIAS4	-1.0325	0.0032	protein inhibitor of activated STAT, 4
17644	ANKRD26P3	-1.0329	0.9999	ankyrin repeat domain 26 pseudogene 3
17645	GNG2	-1.0338	0.0037	guanine nucleotide binding protein (G protein), gamma 2
17646	LOC100128361	-1.0362	0.0043	uncharacterized LOC100128361
17647	PBK	-1.0383	0.0023	PDZ binding kinase
17648	JARID2	-1.04	0.0023	jumonji, AT rich interactive domain 2
17649	CCSAP	-1.04	0.0033	centriole, cilia and spindle-associated protein
17650	RBL1	-1.0412	0.0028	retinoblastoma-like 1 (p107)
17651	POLD1	-1.0417	0.0024	polymerase (DNA directed), delta 1, catalytic subunit
17652	sept-03	-1.0425	0.0023	septin 3
17653	DIP2A	-1.0438	0.0025	DIP2 disco-interacting protein 2 homolog A (Drosophila)
17654	TBC1D7	-1.045	0.0028	TBC1 domain family, member 7
17655	AHSA1	-1.045	0.0029	AHA1, activator of heat shock 90kDa protein ATPase homolog 1 (yeast)
17656	CCP110	-1.0458	0	centriolar coiled coil protein 110kDa
17657	FBXL19	-1.0462	0.0033	F-box and leucine-rich repeat protein 19
17658	CABLES2	-1.0475	0.0035	Cdk5 and Abl enzyme substrate 2
17659	CEP78	-1.0488	0.0021	centrosomal protein 78kDa
17660	KIF21B	-1.0492	0.0032	kinesin family member 21B
17661	ZCCHC17	-1.05	0.0026	zinc finger, CCHC domain containing 17
17662	NPB	-1.05	0.0018	neuropeptide B
17663	CACYBP	-1.05	0.0033	calcyclin binding protein
17664	E2F8	-1.0508	0.0023	E2F transcription factor 8
17665	PHYHIPL	-1.0512	0.002	phytanoyl-CoA 2-hydroxylase interacting protein-like
17666	RRP1	-1.0525	0.0014	ribosomal RNA processing 1 homolog (S. cerevisiae)
17667	C18orf21	-1.0525	0.0035	chromosome 18 open reading frame 21
17668	PSRC1	-1.0533	0.0014	proline/serine-rich coiled-coil 1
17669	MRPL47	-1.0538	0.0027	mitochondrial ribosomal protein L47
17670	TYMS	-1.055	0.004	thymidylate synthetase
17671	REC8	-1.055	0	REC8 homolog (yeast)
17672	C11orf84	-1.055	0.0031	chromosome 11 open reading frame 84
17673	RANBP3	-1.0558	0.0009	RAN binding protein 3
17674	FOXD2-AS1	-1.0562	0.0012	FOXD2 antisense RNA 1 (non-protein coding)
17675	RAD18	-1.0575	0.0022	RAD18 homolog (S. cerevisiae)
17676	FARSA	-1.0583	0.0032	phenylalanyl-tRNA synthetase, alpha subunit
17677	C18orf8	-1.0583	0.0023	chromosome 18 open reading frame 8
17678	IL23R	-1.0588	0.0032	interleukin 23 receptor
17679	UBB	-1.0608	0.0025	ubiquitin B
17680	LSM7	-1.0617	0.0029	LSM7 homolog, U6 small nuclear RNA associated (S. cerevisiae)
17681	UBE2T	-1.0625	0.003	ubiquitin-conjugating enzyme E2T (putative)
17682	TIMM10	-1.0633	0.0029	translocase of inner mitochondrial membrane 10 homolog (yeast)
17683	ZNF101	-1.0638	0	zinc finger protein 101
17684	RPL27A	-1.0638	0.0026	ribosomal protein L27a
17685	LOC152217	-1.0638	0.0023	uncharacterized LOC152217
17686	LIN28B	-1.065	0.0027	lin-28 homolog B (C. elegans)
17687	ZDHHC22	-1.0675	0.0027	zinc finger, DHHC-type containing 22
17688	GTSF1	-1.0688	0.0039	gametocyte specific factor 1
17689	DDA1	-1.07	0.0033	DET1 and DDB1 associated 1
17690	KPNA2	-1.0725	0.0024	karyopherin alpha 2 (RAG cohort 1, importin alpha 1)
17691	CHAF1B	-1.0758	0.0019	chromatin assembly factor 1, subunit B (p60)
17692	CCDC136	-1.0762	0.0025	coiled-coil domain containing 136
17693	RLTPR	-1.0775	0.0031	RGD motif, leucine rich repeats, tropomodulin domain and proline-rich containing
17694	PFDN2	-1.0775	0.0029	prefoldin subunit 2
17695	MANEAL	-1.0775	0.0025	mannosidase, endo-alpha-like
17696	FAM216A	-1.0783	0.0032	family with sequence similarity 216, member A
17697	FOXRED1	-1.0788	0.0032	FAD-dependent oxidoreductase domain containing 1
17698	C19orf52	-1.0788	0	chromosome 19 open reading frame 52
17699	EME1	-1.08	0.0041	essential meiotic endonuclease 1 homolog 1 (S. pombe)
17700	EIF1B	-1.0808	0.0032	eukaryotic translation initiation factor 1B
17701	PLK1	-1.0825	0.0018	polo-like kinase 1
17702	DCAF15	-1.0825	0	DDB1 and CUL4 associated factor 15
17703	LOC100128881	-1.0829	0.9999	uncharacterized LOC100128881
17704	PPP2R3C	-1.0842	0.0038	protein phosphatase 2, regulatory subunit B'', gamma
17705	TNFAIP8L1	-1.085	0.003	tumor necrosis factor, alpha-induced protein 8-like 1
17706	SMAP2	-1.085	0.0021	small ArfGAP2
17707	PRDM15	-1.085	0.0028	PR domain containing 15
17708	LOC254559	-1.085	0.003	uncharacterized LOC254559
17709	HIRIP3	-1.085	0.0001	HIRA interacting protein 3
17710	C3orf62	-1.085	0.0036	chromosome 3 open reading frame 62
17711	ACOT7	-1.085	0.0035	acyl-CoA thioesterase 7
17712	REM2	-1.0875	0.0025	RAS (RAD and GEM)-like GTP binding 2
17713	LOC643837	-1.0875	0.0039	uncharacterized LOC643837
17714	IQGAP3	-1.0875	0.0027	IQ motif containing GTPase activating protein 3
17715	ZNF367	-1.0888	0.0026	zinc finger protein 367
17716	PIN1	-1.0892	0.0023	peptidylprolyl cis/trans isomerase, NIMA-interacting 1
17717	PLD6	-1.09	0.0036	phospholipase D family, member 6
17718	NASP	-1.09	0.0031	nuclear autoantigenic sperm protein (histone-binding)
17719	TTC28-AS1	-1.0912	0.0034	TTC28 antisense RNA 1 (non-protein coding)
17720	WDR53	-1.0938	0.0018	WD repeat domain 53
17721	C9orf100	-1.0938	0	chromosome 9 open reading frame 100
17722	FSD1	-1.0958	0.0037	fibronectin type III and SPRY domain containing 1

17723	EEF1E1	-1.0967	0	eukaryotic translation elongation factor 1 epsilon 1
17724	CCDC18	-1.0971	0.0035	coiled-coil domain containing 18
17725	BEND4	-1.0975	0.0017	BEN domain containing 4
17726	DOT1L	-1.0992	0	DOT1-like, histone H3 methyltransferase (S. cerevisiae)
17727	C3orf26	-1.1	0	chromosome 3 open reading frame 26
17728	HPRT1	-1.1017	0.0032	hypoxanthine phosphoribosyltransferase 1
17729	VRK1	-1.1025	0.0034	vaccinia related kinase 1
17730	CPSF3	-1.1025	0.0029	cleavage and polyadenylation specific factor 3, 73kDa
17731	C6orf52	-1.1037	0.0023	chromosome 6 open reading frame 52
17732	RPP40	-1.1042	0.0042	ribonuclease P/MRP 40kDa subunit
17733	R3HDM1	-1.1067	0.0025	R3H domain containing 1
17734	SNRPC	-1.1075	0.003	small nuclear ribonucleoprotein polypeptide C
17735	DPYSL5	-1.1075	0.0024	dihydropyrimidinase-like 5
17736	CDC34	-1.1108	0	cell division cycle 34 homolog (S. cerevisiae)
17737	KCNG3	-1.1113	0.0026	potassium voltage-gated channel, subfamily G, member 3
17738	POLRMT	-1.1114	0.0033	polymerase (RNA) mitochondrial (DNA directed)
17739	CCDC112	-1.1125	0.0032	coiled-coil domain containing 112
17740	C6orf228	-1.1125	0.0032	chromosome 6 open reading frame 228
17741	SLC39A3	-1.1137	0.0028	solute carrier family 39 (zinc transporter), member 3
17742	RAVER1	-1.1163	0.0021	ribonucleoprotein, PTB-binding 1
17743	SNX10	-1.1167	0.0024	sorting nexin 10
17744	GLT1D1	-1.1187	0.004	glycosyltransferase 1 domain containing 1
17745	H2AFZ	-1.1192	0.0014	H2A histone family, member Z
17746	SGOL2	-1.12	0.0027	shugoshin-like 2 (S. pombe)
17747	KIF5C	-1.1208	0.003	kinesin family member 5C
17748	TCP1	-1.1225	0.0038	t-complex 1
17749	RPUSD3	-1.1263	0.0031	RNA pseudouridylate synthase domain containing 3
17750	OLIG1	-1.1263	0.0029	oligodendrocyte transcription factor 1
17751	HDGFRP2	-1.1263	0	hepatoma-derived growth factor-related protein 2
17752	LOC100288525	-1.1267	0.9999	hypothetical protein LOC100288525
17753	KIAA0226	-1.1283	0	KIAA0226
17754	C9orf40	-1.1283	0.0024	chromosome 9 open reading frame 40
17755	AP1M1	-1.13	0	adaptor-related protein complex 1, mu 1 subunit
17756	KISS1R	-1.1337	0	KISS1 receptor
17757	LOC100144603	-1.1343	0.9999	uncharacterized transcript
17758	ASRGL1	-1.1375	0.0002	asparaginase like 1
17759	PSMA6	-1.1383	0.0037	proteasome (prosome, macropain) subunit, alpha type, 6
17760	DHX30	-1.1383	0.0023	DEAH (Asp-Glu-Ala-His) box polypeptide 30
17761	LOC442075	-1.1387	0.003	uncharacterized LOC442075
17762	RNF126	-1.1392	0.0037	ring finger protein 126
17763	PCMT1	-1.1408	0.0034	protein-L-isoaspartate (D-aspartate) O-methyltransferase
17764	DLEU2	-1.1425	0	deleted in lymphocytic leukemia 2 (non-protein coding)
17765	RNF168	-1.1437	0.002	ring finger protein 168, E3 ubiquitin protein ligase
17766	PCNT	-1.1525	0.0023	pericentrin
17767	AGPAT5	-1.1525	0.0017	1-acylglycerol-3-phosphate O-acyltransferase 5 (lysophosphatidic acid acyltransferase, epsilon)
17768	RFC4	-1.1533	0.0029	replication factor C (activator 1) 4, 37kDa
17769	ELAVL1	-1.1542	0.0031	ELAV (embryonic lethal, abnormal vision, Drosophila)-like 1 (Hu antigen R)
17770	CAMK2N2	-1.1563	0	calcium/calmodulin-dependent protein kinase II inhibitor 2
17771	FAM47C	-1.1571	0.0034	family with sequence similarity 47, member C
17772	SNAPC4	-1.1575	0.0026	small nuclear RNA activating complex, polypeptide 4, 190kDa
17773	PKN1	-1.1583	0.0023	protein kinase N1
17774	TMPO-AS1	-1.1586	0.9999	TMPO antisense RNA 1 (non-protein coding)
17775	LOC100287015	-1.1586	0.9999	uncharacterized LOC100287015
17776	ZNF653	-1.1588	0.0035	zinc finger protein 653
17777	ASH2L	-1.16	0	ash2 (absent, small, or homeotic)-like (Drosophila)
17778	POLR2D	-1.1608	0.0031	polymerase (RNA) II (DNA directed) polypeptide D
17779	OTUD7A	-1.1612	0.0031	OTU domain containing 7A
17780	FEN1	-1.1642	0.0021	flap structure-specific endonuclease 1
17781	MED26	-1.165	0	mediator complex subunit 26
17782	TUBA1B	-1.1658	0.0031	tubulin, alpha 1b
17783	NUP62	-1.1683	0.0044	nucleoporin 62kDa
17784	MAP1S	-1.1683	0.0031	microtubule-associated protein 1S
17785	TICRR	-1.1688	0	TOPBP1-interacting checkpoint and replication regulator
17786	PRR22	-1.1712	0.0031	proline rich 22
17787	FBXO43	-1.1729	0.003	F-box protein 43
17788	C14orf23	-1.1743	0.0039	chromosome 14 open reading frame 23
17789	SYCE2	-1.175	0.0036	synaptonemal complex central element protein 2
17790	CCDC150	-1.175	0.0027	coiled-coil domain containing 150
17791	YARS	-1.1792	0	tyrosyl-tRNA synthetase
17792	ZBTB17	-1.18	0.0004	zinc finger and BTB domain containing 17
17793	C18orf56	-1.1862	0.0028	chromosome 18 open reading frame 56
17794	SGTA	-1.1875	0.0029	small glutamine-rich tetratricopeptide repeat (TPR)-containing, alpha
17795	CLSPN	-1.1888	0.0026	claspin
17796	CCDC138	-1.1925	0.0035	coiled-coil domain containing 138
17797	RNF157	-1.1938	0.0029	ring finger protein 157
17798	DTNBP1	-1.1938	0	dystrobrevin binding protein 1
17799	ZNF77	-1.195	0.0033	zinc finger protein 77
17800	CCDC167	-1.1962	0.0036	coiled-coil domain containing 167
17801	CDCA5	-1.2	0.0033	cell division cycle associated 5
17802	CXorf65	-1.2062	0.0025	chromosome X open reading frame 65
17803	SBK1	-1.2125	0.0026	SH3-binding domain kinase 1
17804	RQCD1	-1.2162	0	RCD1 required for cell differentiation1 homolog (S. pombe)
17805	DDX49	-1.2175	0.0032	DEAD (Asp-Glu-Ala-Asp) box polypeptide 49
17806	CKAP5	-1.2175	0	cytoskeleton associated protein 5

17807	ZKSCAN2	-1.2225	0.0027	zinc finger with KRAB and SCAN domains 2
17808	KIF2A	-1.2225	0.0021	kinesin heavy chain member 2A
17809	DNMT1	-1.2225	0.0036	DNA (cytosine-5-)-methyltransferase 1
17810	TMEM201	-1.2287	0.0026	transmembrane protein 201
17811	GSG2	-1.2287	0	germ cell associated 2 (haspin)
17812	ZNF519	-1.2333	0.0026	zinc finger protein 519
17813	HES6	-1.2337	0.0027	hairy and enhancer of split 6 (Drosophila)
17814	BABAM1	-1.2358	0.0033	BRISC and BRCA1 A complex member 1
17815	FLJ37453	-1.2363	0.0029	uncharacterized LOC729614
17816	KBTBD8	-1.2387	0	kelch repeat and BTB (POZ) domain containing 8
17817	FAM78A	-1.24	0.0029	family with sequence similarity 78, member A
17818	C17orf96	-1.2413	0.0034	chromosome 17 open reading frame 96
17819	NARF	-1.2458	0.0014	nuclear prelamin A recognition factor
17820	CENPW	-1.2525	0.0029	centromere protein W
17821	ESCO2	-1.2575	0.0029	establishment of cohesion 1 homolog 2 (S. cerevisiae)
17822	RANGAP1	-1.2583	0.0036	Ran GTPase activating protein 1
17823	PDCD2L	-1.2613	0	programmed cell death 2-like
17824	SKA3	-1.2625	0.0033	spindle and kinetochore associated complex subunit 3
17825	LYAR	-1.2663	0.0018	Ly1 antibody reactive homolog (mouse)
17826	KDM2B	-1.27	0	lysine (K)-specific demethylase 2B
17827	RELT	-1.2713	0	RELT tumor necrosis factor receptor
17828	NUF2	-1.2713	0	NUF2, NDC80 kinetochore complex component, homolog (S. cerevisiae)
17829	FAM53C	-1.2767	0.0028	family with sequence similarity 53, member C
17830	CHAF1A	-1.2825	0.0041	chromatin assembly factor 1, subunit A (p150)
17831	RAD51C	-1.2833	0.0029	RAD51 homolog C (S. cerevisiae)
17832	MAD2L2	-1.2925	0.003	MAD2 mitotic arrest deficient-like 2 (yeast)
17833	MND1	-1.305	0	meiotic nuclear divisions 1 homolog (S. cerevisiae)
17834	COPS3	-1.3067	0	COP9 constitutive photomorphogenic homolog subunit 3 (Arabidopsis)
17835	KIAA1524	-1.3175	0.0029	KIAA1524
17836	POC1A	-1.3212	0.0033	POC1 centriolar protein homolog A (Chlamydomonas)
17837	H2AFX	-1.3217	0.003	H2A histone family, member X
17838	RHEBL1	-1.3288	0.0028	Ras homolog enriched in brain like 1
17839	FBXO45	-1.3362	0.0023	F-box protein 45
17840	PWP2	-1.345	0.0044	PWP2 periodic tryptophan protein homolog (yeast)
17841	PDXP	-1.3513	0.0032	pyridoxal (pyridoxine, vitamin B6) phosphatase
17842	CMC2	-1.3517	0.0031	COX assembly mitochondrial protein 2 homolog (S. cerevisiae)
17843	SAAL1	-1.3575	0.0026	serum amyloid A-like 1
17844	RNF34	-1.3575	0	ring finger protein 34, E3 ubiquitin protein ligase
17845	SLC25A19	-1.365	0.0031	solute carrier family 25 (mitochondrial thiamine pyrophosphate carrier), member 19
17846	SGOL1	-1.3725	0.0028	shugoshin-like 1 (S. pombe)
17847	PARP1	-1.3883	0.0026	poly (ADP-ribose) polymerase 1
17848	DEPDC1B	-1.3975	0.0026	DEP domain containing 1B
17849	CDCA2	-1.4075	0.0032	cell division cycle associated 2
17850	C21orf58	-1.4225	0.0033	chromosome 21 open reading frame 58
17851	FAM54A	-1.4262	0.0021	family with sequence similarity 54, member A
17852	SNRPD1	-1.4417	0.0028	small nuclear ribonucleoprotein D1 polypeptide 16kDa
17853	HAUS8	-1.4713	0	HAUS augmin-like complex, subunit 8
17854	STMN1	-1.5117	0.0033	stathmin 1
17855	FANCD2	-1.5175	0.0033	Fanconi anemia, complementation group D2
17856	C11orf82	-1.5412	0.0029	chromosome 11 open reading frame 82
17857	PRR11	-1.6233	0.0026	proline rich 11
