# Supplementary material for: PLA2R1 promotes DNA damage and inhibits spontaneous tumor formation during aging
Source: Cell Death Dis. 2021 Feb 16;12(2):190. doi: 10.1038/s41419-021-03468-3 (PMC7887270; doi:10.1038/s41419-021-03468-3)
Supplement: Supplementary file 3 — Supplementary Table 2 [file 41419_2021_3468_MOESM3_ESM.pdf]

Supplemental Table 2: correlation between PLA2R1 and DNA repair genes			
	**Gene**	**Coexpression Score**	**P-Value**
MMR	MSH2	-0.7142	0.2366
	MSH3	0.4575	0.2546
	MSH6	-0.7175	0.1978
	MLH1	-0.6142	0.189
	MLH3	0.1275	0.3535
	EXO1	-0.4825	0.2454
	RPA1	-0.5052	0.3034
	LIG1	-0.7967	0.0722
	POLQ	-0.7075	0.103
DNA damage bypass(translesion synhesis)	POLI	0.2492	0.3868
	REV1	-0.0608	0.5194
	POLH	-0.1158	0.4238
BER	APEX1	-0.0492	0.5021
	APEX2	-0.2783	0.3183
	POLB	-0.8633	0.0039
	OGG1	0.115	0.2737
	MPG	0.4592	0.1704
SSB repair	PARP1	-1.3883	0.0026
	XRCC1	-0.7075	0.0448
DSB repair	ATM	0.3483	0.2839
	BRCA1	-0.6842	0.0954
	CHEK2	-0.1257	0.4743
	RAD51	-0.5175	0.2
	BRCA2	-0.2725	0.3952
	PALB2	-0.1692	0.4442
NER	ERCC6L	-0.6608	0.132
	ERCC8	0.1892	0.3892
	XPA	-0.0192	0.5046
	XPC	0.4842	0.1362
Fanconi anemia pathway	FANCD2	-1.5175	0.0033
	FANCB	-1.0087	0.0024
	FANCM	-0.9388	0.0095
	FANCA	-0.865	0.0383
