# Supplementary figures and images for: PLA2R1 promotes DNA damage and inhibits spontaneous tumor formation during aging
Source: Cell Death Dis. 2021 Feb 16;12(2):190. doi: 10.1038/s41419-021-03468-3 (PMC7887270; doi:10.1038/s41419-021-03468-3)

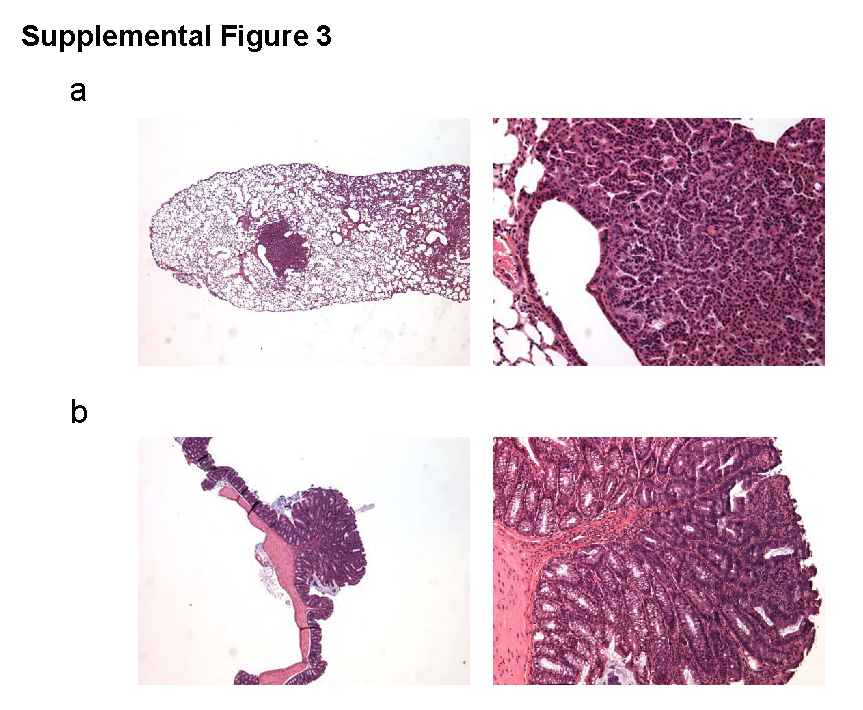

Supplement: Supplementary file 6 — Supplementary Figure 3 [file 41419_2021_3468_MOESM6_ESM.tif]

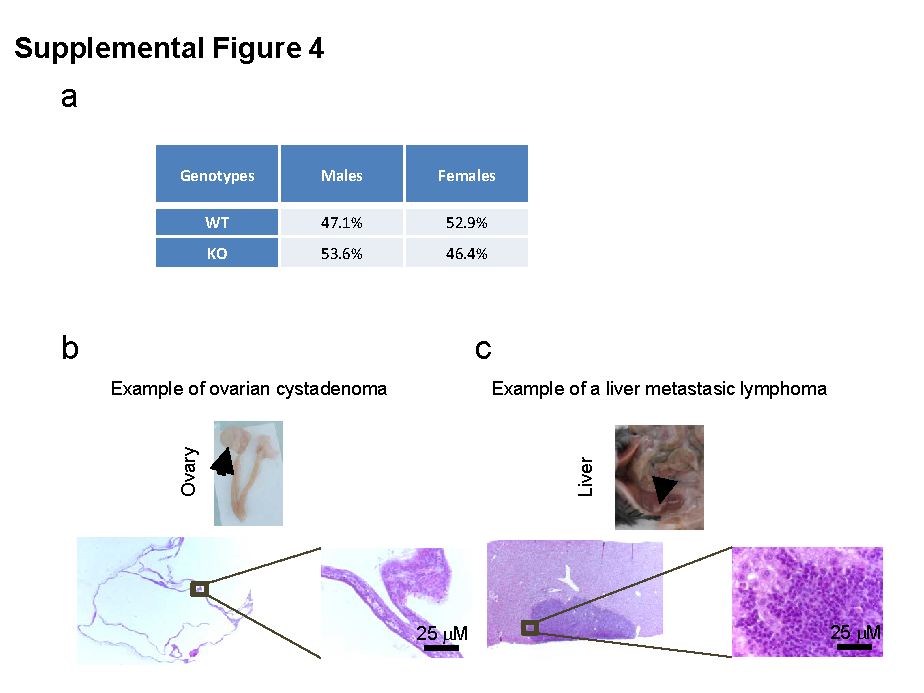

Supplement: Supplementary file 7 — Supplementary Figure 4 [file 41419_2021_3468_MOESM7_ESM.tif]

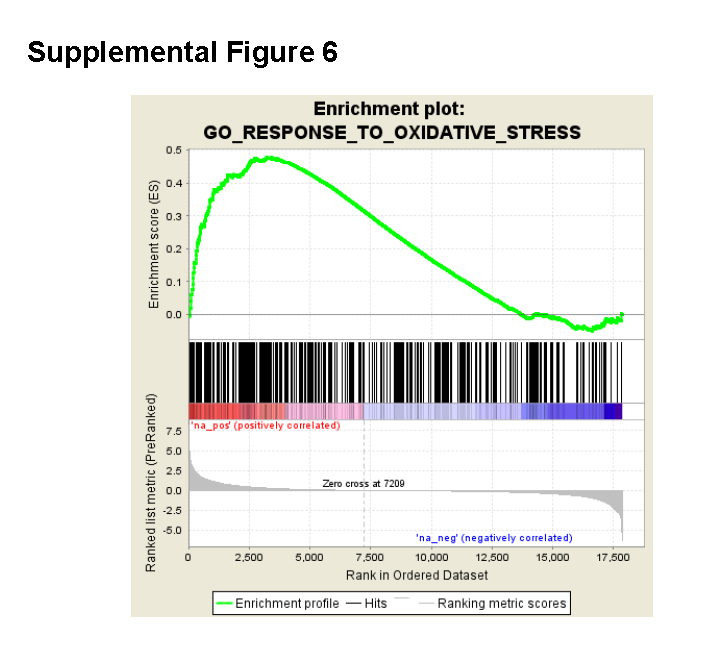

Supplement: Supplementary file 9 — Supplementary Figure 6 [file 41419_2021_3468_MOESM9_ESM.tif]
